# Development of novel InDel markers by whole-genome sequence comparison and genetic diversity assessment of Thailand rice blast fungus populations

**DOI:** 10.1007/s44154-025-00212-1

**Published:** 2025-04-27

**Authors:** Napassorn Thamkirati, Worrawit Suktrakul, Athipat Ngernmuen, Theerayut Toojinda, Sureeporn Katengam, Nonglak Parinthawong, Waree Laophermsuk, Pradipha Pradapphai, Watchareeporn Suksiri, Suphattra Janthasri, Chatchawan Jantasuriyarat

**Affiliations:** 1 Department of Genetics, Faculty of Science, Kasetsart University, Bangkok, 10900 Thailand; 2 Department of Zoology, Faculty of Science, Kasetsart University, Bangkok, 10900 Thailand; 3 Rice Science Center, Kasetsart University, Nakhon Pathom, 73140 Thailand; 4 Department of Agronomy, Faculty of Agriculture, Ubon Ratchathani University, Ubon Ratchathani, 34190 Thailand; 5 Department of Plant Production Technology, School of Agricultural Technology, King Mongkut’s Institute of Technology Ladkrabang, Bangkok, 10520 Thailand; 6 Center for Advanced Studies in Tropical Natural Resources, National Research University-Kasetsart (CASTNAR, NRU-KU), Kasetsart University, Bangkok, 10900 Thailand

**Keywords:** Genetic diversity, Rice blast fungus, Rice blast, Polymorphism

## Abstract

InDel markers are commonly used to assess genetic relationships among populations. In this study, we employed a whole-genome sequence comparison method to identify and develop InDel markers for the rice blast fungus *Pyricularia oryzae*. We analyzed 152 whole-genome sequences of *P. oryzae* isolates from diverse global regions, including Brazil, Burundi, China, Colombia, Côte d'Ivoire, France, Ghana, Hungary, India, Japan, Korea, Laos, Madagascar, Mali, Morocco, Nepal, the Philippines, Portugal, Spain, Suriname, Thailand, the UK, the USA, and Zambia. Our analysis identified a total of 233,595 InDel loci distributed across the seven chromosomes of *P. oryzae*. From these, 82 loci were selected based on their high polymorphism across the 152 genome sequences. The effectiveness of these 82 loci was assessed by analyzing the genetic diversity of 47 Thai rice blast isolates alongside two reference isolates, GUY11 (France) and KJ201 (Korea). Of the 82 InDel loci, 33 exhibited polymorphisms, with 2–4 alleles per locus and polymorphic information content (PIC) scores ranging from 0.04 to 0.67. Principal coordinate and structure analyses revealed two genetic subgroups among the Thai rice blast isolates, categorized according to host specificity. Genetic relationships highlighted disparities among rice blast populations based on their respective hosts: rice and grassy weeds. This finding suggests a correlation between genetic relatedness and the plant hosts susceptible to rice blast disease. The newly developed InDel markers provide a valuable resource for future research in this field.

**Supplementary Information:**

The online version contains supplementary material available at 10.1007/s44154-025-00212-1.

## Introduction

Rice blast is a destructive disease that affects rice production globally and is caused by the ascomycete fungus *Pyricularia oryzae* (telomorph: *Magnaporthe oryzae*). The pathogen exhibits multi-host behavior, infecting various Poaceae species including ryegrass (*Lolium multiflorum*), crabgrass (*Digitaria sanguinalis*), goose grass (*Eleusine indica*), rice (*Oryza sativa*), wheat (*Triticum aestivum*), barley (*Hordeum vulgare*), and foxtail millet (*Setaria italica*) (Qi et al. 2019; Thierry et al. 2020; Tsukiboshi 2022). This demonstrates that infection across various Poaceae hosts is mobile, suggesting the potential for host shifts and expansion (Chung et al. 2020). First reported in China in 1673, rice blast affects all stages of rice development, causing seedling, leaf, node, grain, and neck blasts (Khan et al. 2016; Younas et al. 2023). While cultivating resistant rice varieties is the most effective control method (Maciel 2011), single-gene resistance can be quickly lost due to pathogen adaptation (Miah et al. 2013). The presence of more virulent pathotypes further complicates breeding efforts (Tuan et al. 2020). Therefore, understanding the genetic diversity and mechanisms driving the pathogen's ability to overcome resistance is essential. Genetic alterations or shifts in frequency within pathogen populations require comprehensive genetic data to develop effective and sustainable disease management strategies (Levy et al. 1993).

Molecular markers are widely used to study inheritance patterns and gene identification. Hybridization and PCR-based techniques are commonly employed to detect polymorphisms, enabling the identification of variations among DNA fragments or genotypes under investigation. Commonly used molecular markers include Restriction Fragment Length Polymorphism (RFLP), Random Amplified Polymorphic DNA (RAPD), Simple Sequence Repeat (SSR), Single-Nucleotide Polymorphism (SNP), and Insertion and Deletion (InDel) markers, all of which play critical roles in genetic diversity studies (Gupta et al. 2001). Ideal markers display polymorphism, high-resolution genetic differences, codominance, ease of amplification, and cost-effectiveness (Agarwal et al. 2008). InDels, which represent insertions or deletions of nucleotide bases, are particularly valuable due to their stability and codominant inheritance, making them suitable for population studies (Yang et al. 2016). These markers are accurate, reproducible, and simple to genotype (Jain et al. 2019; Niihama et al. 2015), and have been extensively applied in research on plant pathogens, human health, and food science (Oliveira and Azevedo 2022).

Recently, whole-genome sequence analysis has become a powerful technique for developing molecular markers due to its ability to identify a large number of genetic variants and potential markers associated with specific functional elements or traits of interest (Xu et al. 2022; Khodaeiaminjan et al. 2018; Sorkheh et al. 2016). This approach has been used to develop various types of molecular markers, including SSRs, SNPs, and InDels, and has proven successful in validating and optimizing these markers (Bhattarai and Mehlenbacher 2017). For example, Ngernmuen et al. (2019) used whole-genome sequences of the rice blast fungus to develop SSR markers, which were subsequently applied to assess genetic relationships in *P. oryzae*. Similarly, Labbé et al. (2008) used whole-genome sequencing to construct a genetic linkage map for the ectomycorrhizal fungus *Laccaria bicolor*, developing SNP, RAPD, and SSR markers. However, no InDel markers have been reported for *P. oryzae* to date. This study aimed to develop polymorphic InDel markers by analyzing 152 whole-genome sequences of rice blast fungus collected from various regions worldwide, available in a public database, through whole-genome comparison. The newly developed markers were then used to assess the genetic relationships within a selected population of Thai rice blast fungus. The findings of this study highlight the efficacy of the whole-genome sequence comparison method as a successful strategy for generating new molecular markers. Furthermore, it underscores the effectiveness of InDel markers in evaluating genetic diversity within the rice blast population.

## Results

### Comparison of whole-genome sequences and identification of InDel markers

**Fig. 1:**
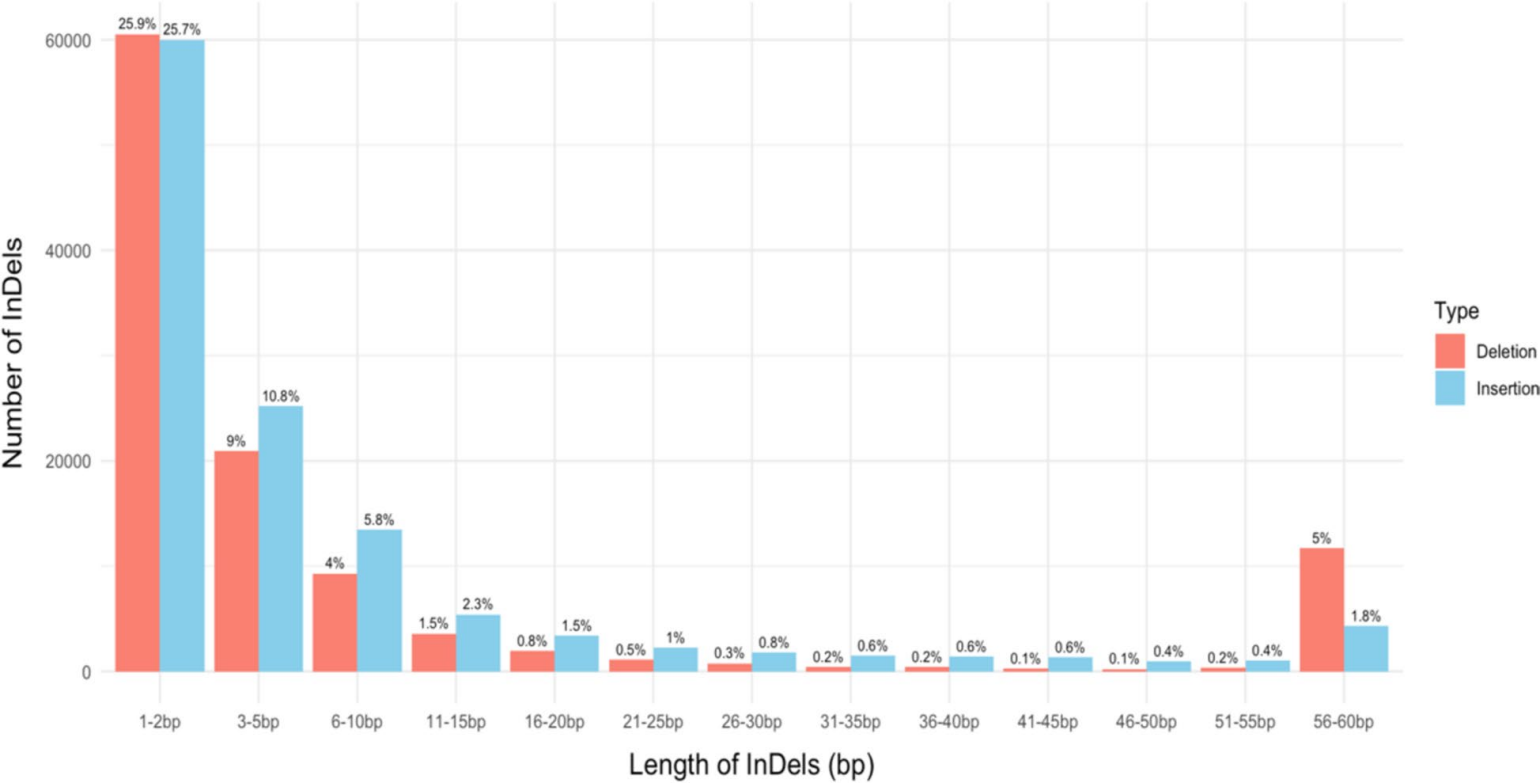
Distribution of the InDel length in 152 whole genome rice blast isolates

A total of 233,595 InDels were identified by comparing the whole-genome sequences of 152 isolates from global populations with the reference genome of *P. oryzae* strain 70–15. The analysis revealed variable numbers of InDel loci across the chromosomes of the rice blast fungus. The highest number of InDels was found on chromosome 1 (47,833 InDels), followed by chromosome 2 (44,409 InDels), chromosome 3 (36,377 InDels), chromosome 4 (30,845 InDels), chromosome 5 (26,499 InDels), chromosome 6 (25,657 InDels), and chromosome 7 (21,974 InDels). The InDel length distribution ranged from 1 to 60 bp, with the majority being 1–2 bp long (51.60%), followed by 3–5 bp (19.80%), 6–10 bp (9.80%), 11–15 bp (3.80%), 16–20 bp (2.30%), 21–25 bp (1.50%), 26–30 bp (1.10%), 31–35 bp (0.80%), 36–40 bp (0.80%), 41–45 bp (0.70%), 46–50 bp (0.50%), 51–55 bp (0.60%), and 56–60 bp (6.80%), as illustrated in Fig. 1.

**Fig. 2:**
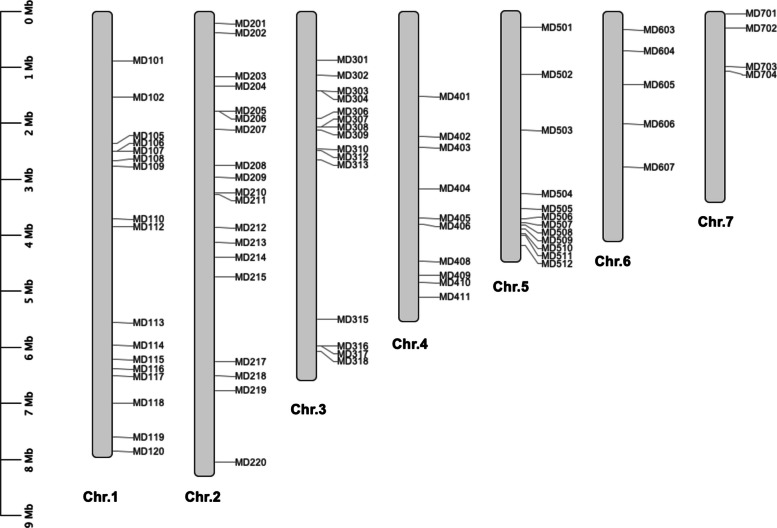
The genetic map of the rice blast fungus genome and highlights the locations of InDel markers. The scale bar indicates the length of each chromosome in million base pairs (Mb)

Each variation corresponded to a distinct allele at each InDel locus. From these, we developed polymorphic markers by selecting InDel loci with more than two alleles, each having a minimum allele frequency of 0.05. A total of 82 InDel loci were carefully selected, distributed across all seven chromosomes of the rice blast fungus. Specifically, 17 loci were located on chromosome 1, while chromosomes 2, 3, 4, 5, 6, and 7 contained 19, 15, 10, 12, 5, and 4 loci, respectively (Fig. 2).

These selected loci exhibited variability in the number of variants, ranging from 3 to 7 variants per locus, with an average of 5.84 variants. The Polymorphic Information Content (PIC) scores for these 82 InDel loci ranged from 0.06 to 0.82 (Table 1). Consequently, we used these 82 InDel markers to further investigate the genetic diversity of the Thai rice blast population.

**Table 1:** Development of 82 InDel marker from whole-genome comparison: Marker name, chromosomal position, number of alleles, variants value, and PIC scores

**Chr.**	**Name**	**Chromosomal position**	**No. of alleles**	**Variation and frequency from whole-genome comparison**^**a**^	**PIC scores**
1	MD101	882997	7	-23(12), -17(2), -16(2), 0(78), + 15(1), + 21(1), + 22(1)	0.34
	MD102	1532014	7	-18(5), -12(6), 0(37), + 6(74), + 18(4), + 48(9), + 54(12)	0.67
	MD105	2361505	7	-15(1), -8(6), 0(102), + 5(7), + 6(7), + 7(8), + 8(3)	0.41
	MD106	2495243	7	-22(11), -20(10), -18(17), -8(4), -6(6), -4(5), 0(88)	0.59
	MD107	2495261	6	-50(15), -12(8), -4(65), 0(30), + 15(32)	0.71
	MD108	2663882	5	-18(14), -15(12), -7(4), 0(82), + 10(30)	0.60
	MD109	2771158	7	-15(1), -13(8), -11(2), 0(103), + 2(7), + 3(6), + 4(2)	0.35
	MD110	3711451	7	-2(2), -1(81), 0(35), 1(7), 2(13), 10(13), + 20(1)	0.65
	MD112	3846322	7	-24(15), -5(2), 0(108), + 8(7), + 10(5), + 14(3), + 26(5)	0.43
	MD113	5557275	4	-24(53), -12(7), 0(88), + 36(1)	0.52
	MD114	5966005	7	-12(11), -9(47), -6(60), 0(16), + 9(4), + 12(5), + 18(4)	0.71
	MD115	6218659	7	-36(4), -32(4), -24(4), -22(23), -20(9), -18(7), 0(94)	0.55
	MD116	6383854	7	-8(7), -6(61), -2(9), 0(15), + 2(8), + 4(33), + 6(6)	0.73
	MD117	6501456	7	-8(21), -6(18), -2(40), 0(40), + 7(7), + 10(16), + 16(7)	0.81
	MD118	6990217	7	-8(3), -6(47), -4(3), -2(69), 0(5), + 2(14), + 18(6)	0.66
	MD119	7605832	7	-17(10), -7(10), -6(10), -5(6), 0(104), + 6(5), + 9(2)	0.48
	MD120	7852311	7	-30(13), -15(36), 0(85), + 15(1), + 30(1), + 45(3), + 60(1)	0.56
2	MD201	220876	7	-16(74), -14(9), -12(4), -10(1), -4(4), -2(3), 0(53)	0.62
	MD202	389841	7	-8(9), -7(2), -5(44), -4(23), -3(12), -2(24), 0(24)	0.80
	MD203	1162885	7	-11(11), -10(1), -8(3), -7(43), -6(26), -5(18), 0(40)	0.77
	MD204	1337169	7	-21(9), -18(1), -15(7), -12(76), -9(48), -6(3), 0(6)	0.63
	MD205	1780411	7	-14(12), -13(43), -11(7), -10(3), -6(9), -5(19), 0(51)	0.75
	MD206	1784234	7	-14(3), -12(7), -10(63), -8(61), -6(5), -4(2), 0(8)	0.65
	MD207	2107779	5	-18(16), -12(11), -11(5), -10(26), 0(86)	0.59
	MD208	2750543	7	-6(4), -5(51), -4(11), -3(7), -2(13), -1(54), 0(9)	0.73
	MD209	2965943	7	-30(6), -22(23), -20(27), -16(6), -4(9), 0(49), + 6(26)	0.79
	MD210	3236948	7	-30(6), -22(1), -18(7), -2(3), 0(34), + 2(19), + 8(83)	0.64
	MD211	3275446	6	-30(1), -24(13), -6(3), 0(27), + 6(10), + 12(98)	0.54
	MD212	3864621	7	-24(4), -15(5), -9(6), -6(14), -3(95), 0(14), + 9(9)	0.56
	MD213	4129283	7	-22(3), -11(9), 0(33), + 11(46), + 22(49), + 33(12), + 44(1)	0.76
	MD214	4394130	7	-33(12), -21(32), -18(57), -15(7), -6(12), -3(16), 0(12)	0.77
	MD215	4736781	7	-16(10), -8(15), -4(5), 0(72), + 12(40), + 16(3), + 20(4)	0.68
	MD217	6254110	5	-24(15), -12(8), 0(93), + 12(28), + 24(8)	0.58
	MD218	6510164	4	-20(14), -10(24), 0(50), + 2 (35)	0.71
	MD219	6766168	6	-27(64), -18(3), -9(69), 0(13), + 9(1), + 18(1)	0.60
	MD220	8042406	7	-28(6), -26(51), -24(7), -6(5), -4(22), -2(23), 0(30)	0.78
3	MD301	869112	6	-28(8), -26(2), -20(105), -18(20), -6(15), 0(2)	0.49
	MD302	1145485	3	-16(2), -8(142), 0(6)	0.10
	MD303	1422947	7	-24(62), -20(25), -18(17), -16(2), -14(4), -12(5), 0(32)	0.73
	MD304	1422968	3	-28(56), -20(11), 0(58)	0.58
	MD306	1911873	4	-16(8), -8(49), 0(79), + 8(14), + 36(1)	0.61
	MD307	2060125	7	-18(5), -14(8), -12(34), -10(18), -8(19), -6(33), 0(23)	0.82
	MD308	2063753	4	-26(9), -20(17), -16(22), 0(86)	0.54
	MD309	2120256	7	-10(2), -9(8), -6(9), -5(19), -4(14), -3(9), 0(76)	0.65
	MD310	2462713	7	-20(1), -19(1), -18(1), -15(3), -14(10), -11(11), 0(110)	0.34
	MD312	2491892	7	-12(54), -9(15), -6(13), -3(7), 0(53), + 3(3), + 9(3)	0.72
	MD313	2658725	7	-16(1), -6(1), -5(6), -4(5), -3(3), -2(3), 0(119)	0.25
	MD315	5495540	7	-18(9), -16(3), -6(29), -4(33), -2(16), 0(19)	0.78
	MD316	5971371	5	-20(21), -10(35), -8(9), -6(13), 0(71)	0.68
	MD317	5971378	4	-19(8), -15(45), 0(59), + 3(39)	0.69
	MD318	6072583	7	-45(11), -30(6), -15(87), 0(9), + 15(7), + 30(1), + 60(1)	0.47
4	MD401	1523192	7	-12(3), -11(16), -10(24), -9(22), -8(9), -6(2), 0(61)	0.73
	MD402	2236773	7	-34(3), -32(7), -26(31), -24(53), -22(28), -20(14), 0(5)	0.76
	MD403	2433428	5	-16(6), -13(18), -12(6), -11(21), 0(97)	0.53
	MD404	3166700	7	-10(1), -9(12), -4(43), -3(7), 0(45), + 5(2), + 24(1)	0.67
	MD405	3692872	5	-18(10), -15(23), -10(18), -9(23), 0(75)	0.68
	MD406	3806291	7	-26(10), 0(84), + 4(14), + 6(10), + 8(7), + 10(3), + 12(4)	0.57
	MD408	4467053	7	-22(31), -20(64), -18(10), -14(3), -6(8), -8(8), 0(18)	0.72
	MD409	4708378	4	-12(35), -5(12), -3(11), 0(85)	0.57
	MD410	4838239	7	-24(1), -22(9), -10(23), -8(53), -6(11), -4(10), 0(18),	0.75
	MD411	5100008	7	-11(9), -10(1), -9(2), -7(20), -6(3), 0(41), + 12(1)	0.63
5	MD501	283634	4	-18(24), -14(16), 0(56), + 18(22)	0.68
	MD502	1122549	3	-15(13), 0(106), + 18(23)	0.41
	MD503	2127209	3	-10(138), -2(11), 0(3)	0.17
	MD504	3257206	4	-20(13), -14(19), 0(87), + 2(11)	0.51
	MD505	3529911	4	-25(11), -20(23), -10(8), 0(67)	0.56
	MD506	3699895	4	-20(34), -10(19), 0(65), + 11(16)	0.67
	MD507	3773379	7	-48(2), -24(1), -46(9), -14(5), 0(115), + 6(6), + 8(7)	0.36
	MD508	3811891	4	-14(36), -2(2), -1(40), 0(68)	0.65
	MD509	3889001	3	-11(54), -6(14), 0(78)	0.57
	MD510	3968102	4	-15(30), -11(12), 0(89), + 14(9)	0.54
	MD511	4004261	6	-18(8), -9(6), 0(66), + 9(62), + 18(9), + 27(1)	0.64
	MD512	4180095	7	-14(2), -4(42), -2(10), 0(63), + 6(10), + 8(7), + 16(12)	0.71
6	MD603	335494	4	-38(14), -15(33), -10(8), 0(87)	0.56
	MD604	708168	4	-37(23), -20(9), 0(96), + 12(20)	0.53
	MD605	1307584	4	-27(36), -15(12), -10(2), 0(85)	0.48
	MD606	2015625	7	-9(37), 0(66), + 27(9), + 36(18), + 45(7), + 54(10), + 63(4)	0.72
	MD607	2780767	4	-50(34), -13(11), 0(96)	0.47
7	MD701	42844	3	-45(33), -12(26), 0(75)	0.59
	MD702	299103	4	-45(2), -36(1), 0(130), + 18(1)	0.06
	MD703	993765	4	-20(14), 0(52), + 5(21), + 8(17)	0.66
	MD704	1073944	7	-38(12), -30(1), -28(17), -20(5), -18(4), -12(7), 0(63)	0.62

^a^The number in () indicates the number of rice blast fungus isolates having the particular InDel variant obtained by comparing the whole-genome sequence with the reference genome of rice blast strain 70–15. + denotes insertion,—denotes deletion

### Genetic diversity assessment in Thai rice blast populations using developed InDel markers

**Fig. 3:**
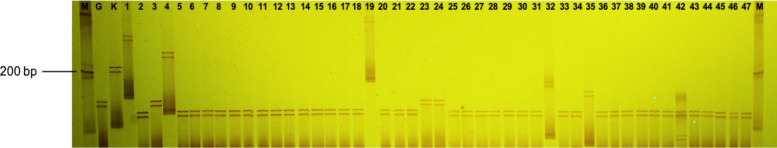
Genetic polymorphism in 47 Thai rice blast populations and two reference isolates (GUY11 and KJ201) detected by InDel marker MD606 on 6% polyacrylamide gel electrophoresis. In the labels, G represents Guy11, K represents KJ201 and numbers 1–47 represent Thai rice blast isolates, 1: PNB61008, 2: YST61001, 3: UTI61102, 4: RBR61109, 5: STI61004, 6: PYO61008, 7: PRE61014, 8: PYO61002, 9: PRE61021, 10: TAK61001, 11: SSK61003, 12: SRN61004, 13: RBR61001, 14: STI61005, 15: LPG61011, 16: PL61001, 17: PL61009, 18: KSN61003, 19: NST61103, 20: NKI61010, 21: NBP61001, 22: LPG61004, 23: CRI61007, 24: MSN61008, 25: KPT61002, 26: YST61004, 27: UBN61011, 28: KKN61005, 29: LRI61002, 30: PL61017, 31: CRI61002, 32: LRI61104, 33: SRN61007, 34: MSN61005, 35: PL61121, 36: NMA61004, 37: UDN61008, 38: NKI61013, 39: PL61006, 40: SKN61009, 41: RBR61003, 42: CMI61001, 43: BRM61010, 44: PCT61003, 45: UBN61017, 46: PL61019, 47: NPM61001

A total of 82 InDel markers were used to investigate the genetic diversity among 47 Thai rice blast isolates and two reference strains, GUY11 and KJ201 (Fig. 3). The PCR amplification products exhibited size variations, reflecting insertions and deletions ranging from -255 to 122 nucleotides. Of the 82 InDel markers, 33 (40.24%) showed polymorphism among the tested isolates, 35 markers yielded monomorphic bands, and 14 markers generated non-specific bands, as detailed in Table 2. Genetic diversity analysis based on the 33 polymorphic InDel markers revealed allelic diversity ranging from 2 to 4 alleles per marker. The Polymorphic Information Content (PIC) scores for these markers ranged from 0.04 to 0.67. MD508 and MD603 had the lowest PIC value of 0.04, while MD107 exhibited the highest PIC value of 0.67, as shown in Table 2.

**Table 2:** Genetic diversity of Thai rice blast population: Chromosomal position, Thai rice blast population variants, and PIC scores

**Chromosome**	**Name**	**Chromosomal position**	**The number of variants**	**PIC scores**
1	MD105	2361505	3	0.19
	MD107	2495261	4	0.67
	MD108	2663882	4	0.15
	MD109	2771158	2	0.42
	MD116	6383854	2	0.08
	MD119	7605832	3	0.52
2	MD201	220876	2	0.18
	MD204	1337169	3	0.58
	MD207	2107779	2	0.49
	MD209	2965943	3	0.15
	MD210	3236948	3	0.37
	MD211	3275446	2	0.24
	MD212	3864621	3	0.37
	MD213	4129283	3	0.36
	MD215	4736781	3	0.40
	MD220	8042406	3	0.19
3	MD303	1422947	2	0.08
	MD304	1422968	3	0.12
	MD306	1911873	2	0.15
	MD308	2063753	3	0.28
	MD312	2491892	2	0.18
4	MD405	3692872	3	0.15
	MD406	3806291	4	0.57
	MD408	4467053	3	0.40
5	MD503	2127209	3	0.22
	MD505	3529911	2	0.21
	MD506	3699895	2	0.11
	MD507	3773379	4	0.60
	MD508	3811891	2	0.04
	MD509	3889001	2	0.21
6	MD603	335494	2	0.04
	MD604	708168	3	0.22
	MD606	2015625	3	0.22
7	MD701	42844	2	0.47
	MD702	299103	2	0.11

### Genetic relationships of Thai rice blast populations

**Fig. 4:**
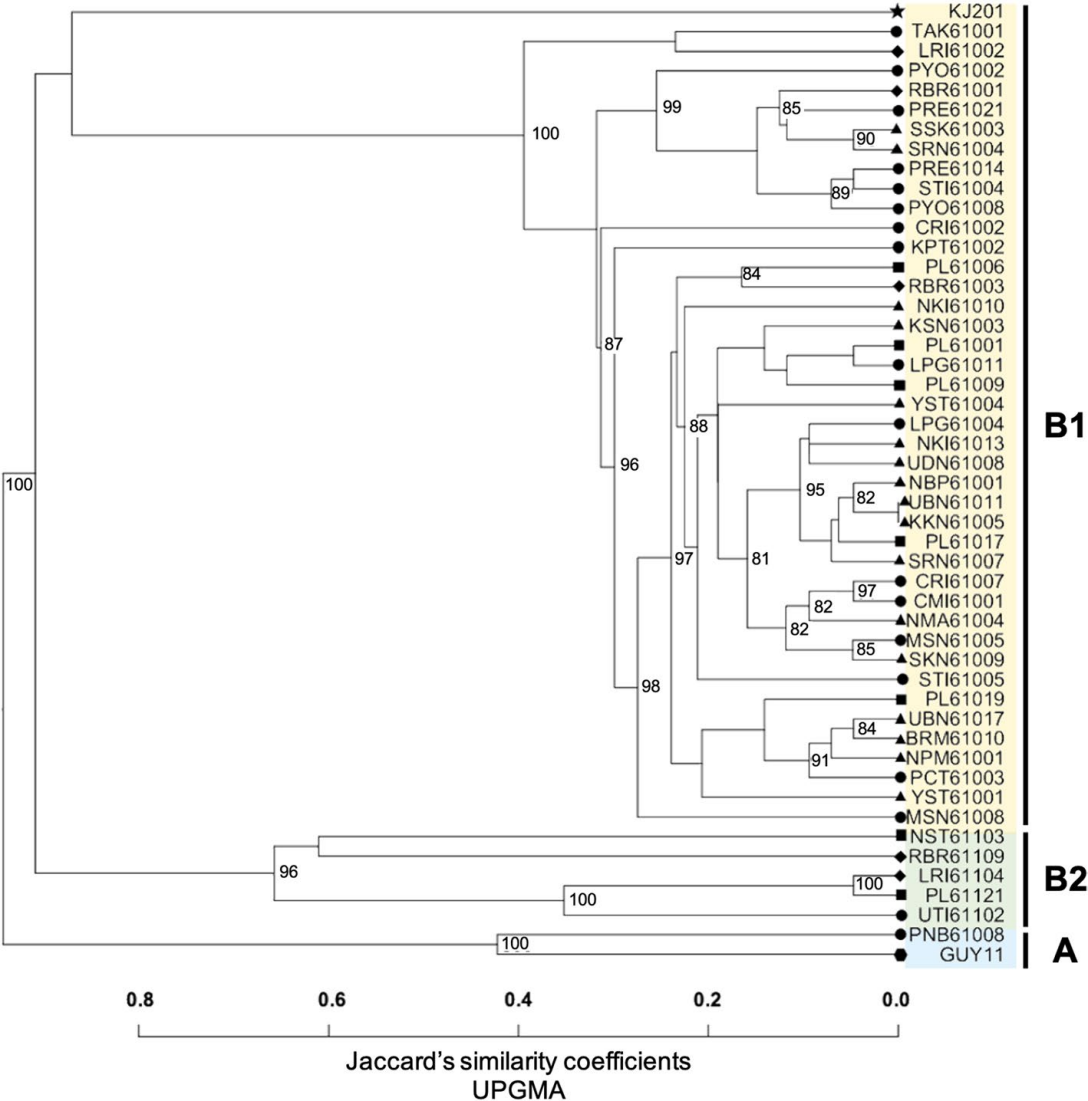
Phylogenetic tree of 49 rice blast isolates using 33 InDel markers, analyzed based on genetic distance with Jaccard similarity coefficient and UPGMA clustering. The shapes represent the region, with circle, triangle, diamond, and square indicating northern, northeastern, central, and southern Thailand, respectively. Star and hexagon shapes indicate the rice blast isolates obtained from Korea and France, respectively. Bootstrap values exceeding 80 are assigned to the corresponding nodes for each cluster

Among the 82 InDel markers, 33 exhibited polymorphisms in 47 Thai rice blast isolates, including the reference strains GUY11 and KJ201. Based on similarity coefficients, which ranged from 0 to 0.8, the 49 rice blast isolates were classified into two major clusters, A and B (Fig. 4). Cluster A comprised only two isolates, GUY11 and PNB61008. Cluster B was further divided into two sub-clusters. The first sub-cluster, B1, included 41 Thai rice blast isolates and one isolate from Korea, forming a distinct clade. These isolates included KJ201, MSN61008, STI61005, PL61009, LPG61011, PL61001, KSN61003, SRN61007, NBP61001, KKN61005, UBN61011, PL61017, LPG61004, NKI61013, UDN61008, NKI61010, YST61004, PCT61003, BRM61010, NPM61001, UBN61017, NMA61004, CMI61001, CRI61007, MSN61005, SKN61009, PL61019, YST61001, RBR61003, PL61006, KPT61002, CRI61002, PYO61008, STI61004, PRE61014, SRN61004, SSK61003, PRE61021, RBR61001, PYO61002, LRI61002, and TAK61001. All isolates in sub-cluster B1 were collected from rice hosts. The second sub-cluster, B2, included five isolates (UTI61102, PL61121, LRI61104, RBR61109, and NST61103), all of which were derived from grassy weeds. The InDel markers effectively differentiated the 47 Thai rice blast isolates, except for UBN61011 and KKN61005, which exhibited identical genetic profiles. Notably, InDel markers developed in this study, such as MD212, MD406, MD505, MD507, and MD509, located on chromosomes 2, 4, and 5, were effective in distinguishing between isolates from rice hosts and grassy weeds. These findings demonstrate the utility of the developed InDel markers for assessing genetic diversity within rice blast populations and highlight their potential application in future studies aimed at elucidating genetic diversity and understanding plant host interactions in rice blast populations.

### Population structure analysis

**Fig. 5:**
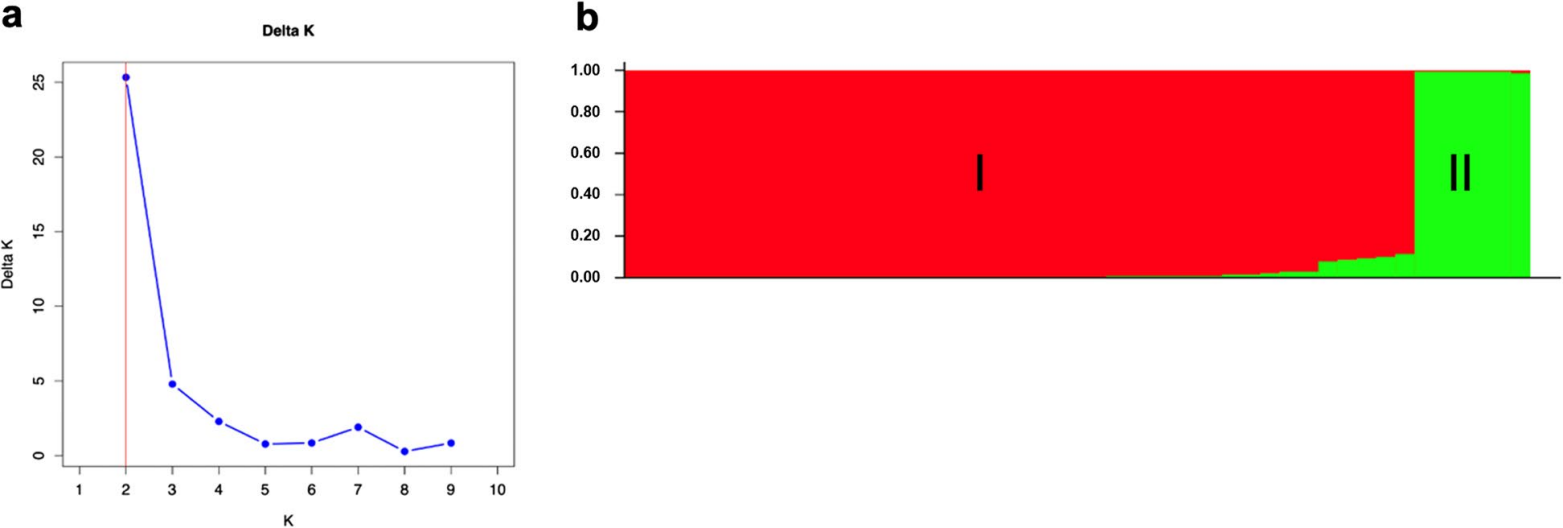
Population structure analysis of 47 Thai rice blast isolates based on 33 InDel markers. **a** The population structure was estimated using Δ*K* values, with a range of *K*-values from 1 to 10. **b** The 47 Thai rice blast isolates were classified into two groups (K = 2) using STRUCTURE 2.3.4. The color coding indicates the distribution of Thai rice blast isolates

The population distribution of 47 Thai rice blast isolates was analyzed using STRUCTURE 2.3.4. The results showed that as the K-value increased from 1 to 10, there was a corresponding rise in the value of inP(D) (Fig. 5a). A population structure distribution map was constructed based on the ΔK value, which classified the 47 isolates into two distinct subgroups (Fig. 5b). Subgroup I consisted of 39 isolates, all derived from rice, while Subgroup II included 6 isolates, all collected from grassy weeds, except for PNB61008, which was sourced from rice.

The genetic diversity statistics are presented in Table 3. The study of the Thai rice blast population revealed that the observed number of alleles (Na) ranged from 2 to 4, while the effective number of alleles (Ne) varied between 1.04 and 2.84. Nei's gene diversity (h) ranged from 0.04 to 0.65, with the highest value observed at locus MD107 and the lowest at loci MD116 and MD303. The mean expected heterozygosity across all loci was calculated to be 0.27. Furthermore, the mean Shannon diversity index (I) was 0.48, indicating a moderate level of genetic diversity within the population.

**Table 3:** Genetic diversity statistics of 47 rice blast population. Na; observed number of alleles, Ne; effective number of alleles, h; Nei's gene diversity, I Shannon’s information index

**Marker/locus**	**Na**	**Ne**	**I**	**h**
MD105	3	1.14	0.28	0.12
MD107	4	2.84	1.16	0.65
MD108	3	1.09	0.21	0.08
MD109	2	1.77	0.63	0.43
MD116	2	1.04	0.10	0.04
MD119	3	2.09	0.82	0.52
MD201	2	1.18	0.29	0.16
MD204	3	2.38	0.98	0.58
MD207	2	1.93	0.67	0.48
MD209	3	1.09	0.21	0.08
MD210	3	1.55	0.66	0.36
MD211	2	1.29	0.38	0.22
MD212	3	1.56	0.67	0.36
MD213	3	1.54	0.62	0.35
MD215	3	1.55	0.65	0.35
MD220	3	1.14	0.28	0.12
MD303	2	1.04	0.10	0.04
MD304	3	1.09	0.21	0.08
MD306	2	1.18	0.29	0.16
MD308	3	1.35	0.48	0.26
MD312	2	1.14	0.24	0.12
MD405	3	1.14	0.28	0.12
MD406	4	2.26	0.98	0.56
MD408	3	1.62	0.70	0.38
MD503	3	1.24	0.39	0.19
MD505	2	1.23	0.34	0.19
MD506	2	1.09	0.18	0.08
MD507	4	2.49	1.11	0.60
MD509	2	1.23	0.34	0.19
MD604	3	1.24	0.41	0.20
MD606	3	1.24	0.39	0.19
MD701	2	1.77	0.63	0.43
MD702	2	1.09	0.18	0.08
MEAN	2.70	1.47	0.48	0.27

**Fig. 6:**
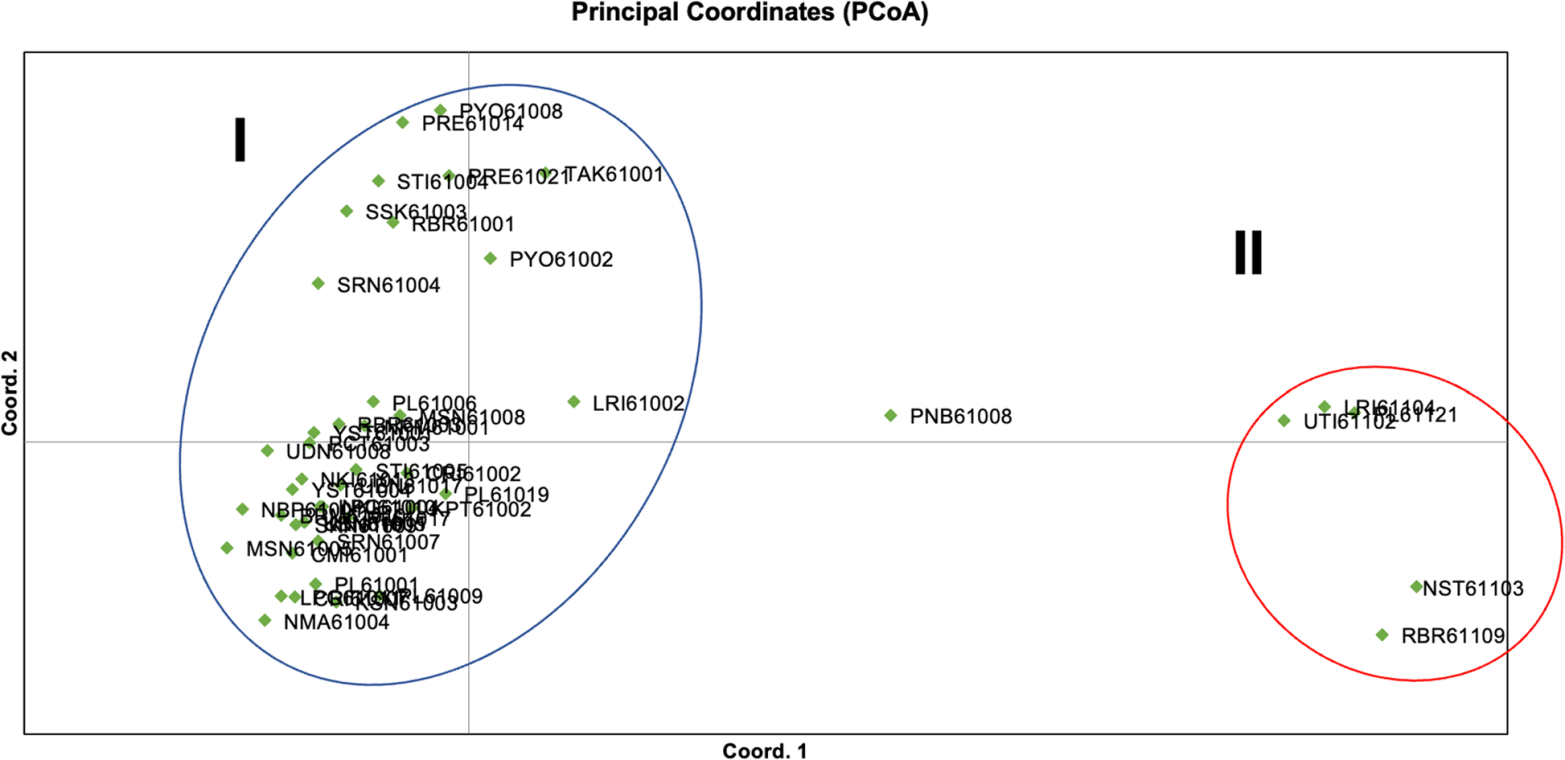
Principal coordinate analysis (PCoA) of the 47 Thai rice blast isolates with 33 InDel marker

The results of the Principal Coordinate Analysis (PCoA) demonstrated that the first and second coordinates accounted for 29.20% and 12.31% of the total variance, respectively. The rice blast population was distinctly divided into two primary groups in the PCoA plot, categorized based on the host from which the rice blast fungus was isolated. Group I comprised 41 isolates obtained from rice plants, while Group II included 5 isolates collected from grassy weeds, as illustrated in Fig. 6.

## Discussion

The development of InDel markers through whole-genome sequence comparison has been successfully applied to various organisms, including *Lupinus luteus* L. (Osorio et al. 2018), *Cucumis sativus* (Adedze et al. 2021), and *Solanum lycopersicum* (Yang et al. 2014). Notably, this approach had not been previously reported for the development of InDel markers in the rice blast fungus until our study. In this research, we utilized whole-genome sequences from 152 isolates of *Pyricularia oryzae*, including 131 sequences from Zhong et al. (2018) and Gladieux et al. (2018b), along with an additional 21 sequences obtained from diverse global regions. These datasets were used to facilitate the development of InDel markers. A total of 233,595 InDel loci were identified across the seven chromosomes of the rice blast fungus. From these, 82 InDel loci were selected for the genetic analysis of Thai rice blast populations, revealing an average of 2.67 variants per locus. Studies by Zhong et al. (2018) and Gladieux et al. (2018b) have shown that the population structure of *P. oryzae*, particularly in Asia, exhibits considerable diversity due to recombination and the acquisition of genetic material from multiple lineages. This suggests that the number of variants in markers developed through whole-genome sequence comparison is influenced by the genetic diversity present in the samples used for marker development. A critical factor in selecting markers for genetic studies is their ability to detect polymorphism. Polymorphic Information Content (PIC) has been widely used for assessing genetic variation (Serrote et al. 2020). In this study, the informativeness of the InDel markers was categorized based on their PIC scores into three groups: highly informative (PIC > 0.5), moderately informative (0.25 < PIC < 0.5), and slightly informative (PIC < 0.25) (Li et al. 2023). Whole-genome comparison and genetic analysis of the Thai rice blast populations revealed that the PIC scores of the InDel markers indicated low to moderate informativeness. These findings are consistent with previous work by Ngernmuen et al. (2019), who successfully developed SSR markers to detect polymorphisms in 58 Thai rice blast isolates, showing similar low to moderate informativeness. Thus, the InDel markers employed in this study proved effective in detecting polymorphisms within the Thai rice blast population.

InDel markers have been utilized in various fungal species through the whole-genome sequence comparison method. For instance, Paola et al. (2017) developed InDel markers based on the aflatoxin biosynthesis cluster in *Aspergillus* spp., enabling the differentiation of groups within section Flavi. Lv et al. (2013) developed 50 InDel markers for *Brassica oleracea* L. var. capitata L., which were associated with Fusarium wilt resistance. These findings highlight the considerable potential of InDels, developed via whole-genome sequence comparison, for investigating global genetic diversity. Additionally, the phylogenetic tree analysis revealed that rice blast isolates within cluster B1 originated exclusively from rice sources, while isolates in cluster B2 were derived from grassy weed sources. This finding suggests a correlation between genetic relationships and the specific plant hosts infected by the rice blast fungus. Among the 152 whole-genome sequences used for InDel marker development in this study, six were derived from rice blast isolates infecting grassy weed species: MZ5-1–6, EI9411, EI9604, LpKY97, UbJA110, and UbJA174 (Table S1). This allowed for the identification of several InDel markers capable of distinguishing between rice blast isolates that infect rice and those that infect grassy weeds. Further investigations are required to identify the genes associated with these markers, as they may play a crucial role in understanding the mechanisms underlying the host specificity of the rice blast fungus. In this study, we identified five InDel markers—MD212, MD406, MD505, MD507, and MD509—that effectively distinguish between rice blast isolates infecting rice and those infecting grassy weeds. Among these markers, MD406 is located within the first exon of the *HAL* protein kinase gene, a key regulator in protein kinase signaling pathways. This kinase plays an essential role in stabilizing nutrient transporters at the plasma membrane, crucial for maintaining cellular functions and nutrient balance (Antunes and Sá-Correia 2022). MD507 is associated with the mitotic control protein Dis3, which encodes a conserved exonuclease enzyme vital for RNA metabolism and mitotic regulation. This enzyme facilitates proper cellular replication and function by promoting the processing and degradation of various RNA species. Gladieux et al. (2018a) investigated historical gene flow between lineages within the rice blast fungus population by identifying SNPs and analyzing single-copy orthologous genes across the entire *P. oryzae* genome from cereal and grass species. Their findings revealed extensive haplotype reticulation among these single-copy orthologs, linking all lineages. These lineages could be classified into cereal and grass groups, encompassing isolates associated with rice, grasses, and wheat. Meanwhile, MD212, MD505, and MD509 are linked to proteins that remain uncharacterized. The discovery of these InDel markers, which distinguish isolates based on host preference, holds significant potential for advancing research into the genetic basis of host specificity. These markers could aid in identifying key genes involved in host–pathogen interactions, such as those governing host recognition, infection, and defense evasion. A deeper understanding of the genetic mechanisms underlying host specificity in *P. oryzae* could provide valuable insights into the evolutionary dynamics of this fungus and its adaptability to new hosts. Ultimately, this knowledge may inform strategies for controlling rice blast in agricultural systems.

Based on the results obtained from Principal Coordinate Analysis (PCoA) and STRUCTURE analysis, it is clear that the 47 Thai rice blast isolates could be categorized into two distinct subgroups, independent of their geographic origin. The first subgroup comprised isolates collected from rice, while the second subgroup consisted of isolates from grassy weeds. These findings suggest that the analysis effectively differentiated the isolates according to host specificity in rice blast infections, although it did not achieve resolution based on geographic origin.

Ngernmuen et al. (2019) developed SSR markers from whole-genome sequences of *P. oryzae* and assessed the genetic diversity of rice blast strains in Thailand, finding a high level of diversity with clustering according to geographic distribution. Similarly, Korinsak et al. (2019) classified 73 isolates from Thailand into eight subpopulations based on geographic origin. However, Yadav et al. (2019) demonstrated that PCoA could categorize 96 *P. oryzae* isolates into two distinct subgroups, based solely on the plant infection source, without regard to geographic location. This classification underscores that the differentiation of isolates is more strongly influenced by the source of infection rather than geographic factors. Sirithunya et al. (2008) also identified four distinct groups among 174 Thai rice blast isolates, revealing that isolates from barley and wild rice clustered together with other rice blast isolates, while those from weeds formed a separate group.

In our study, PCoA analysis indicated that the first two coordinates accounted for 41.51% of the total variation among the isolates. While the explanatory power of these coordinates was limited, they effectively distinguished isolates according to plant host specificity, clearly differentiating rice-associated isolates from those associated with grassy weeds. These results suggest that, although geographic variation was not fully addressed, host-specific differentiation is a prominent factor influencing the genetic structure of the rice blast fungus.

To further understand the genetic mechanisms underlying this differentiation, future research should explore genome-wide association studies (GWAS) or functional genomics approaches. These studies would provide deeper insights into how *P. oryzae* adapts to different hosts. Additionally, expanding the sample set to include isolates from a broader range of hosts and geographic regions would allow for a more comprehensive analysis of the interaction between host specificity and geographic factors in the population structure of *P. oryzae*.

The whole-genome sequence analysis of *P. oryzae* reveals the existence of several distinct lineages, each correlated with a specific host genus. This suggests the potential onset of speciation following host shifts or range expansion (Gladieux et al. 2018a). These findings support the notion that *P. oryzae* populations are closely tied to host interactions, with the pathogen adapting to new environments as it encounters different plant species. During the rice cultivation period, the primary source of infection is the rice crop itself. However, once the rice-growing season concludes, the infection source may shift to alternative hosts, namely the grassy weeds surrounding the rice fields. Given its widespread cultivation and prevalence, rice is particularly susceptible to hosting fungal shifts and may also serve as a conduit for the pathogen to infect co-existing weeds (Couch et al. 2005). When a pathogen successfully transitions to a new host, it undergoes specialization and genetic divergence on that host, completing the process of host shift (MacLeod et al. 2001; Talbot 2003). Such host shifts, coupled with genetic divergence, can lead to the emergence of specialized *P. oryzae* populations, potentially driving early speciation. Over time, these populations may adapt to specific host environments, resulting in genetically distinct strains (Baudin et al. 2024). This host-driven divergence has significant implications for both disease management and the evolutionary dynamics of *P. oryzae*. Ongoing gene flow between rice and weed populations may increase genetic diversity, complicating efforts to control rice blast. Therefore, it is crucial to monitor both rice and weed populations for the emergence of new fungal strains.

The identification of distinct lineages associated with specific hosts highlights the dynamic nature of host–pathogen interactions and the influence of environmental factors in shaping fungal evolution. In this context, the development of InDel markers that can distinguish between rice and grassy weed isolates offers valuable insights into the evolutionary dynamics and host specificity of the rice blast fungus. Further research aimed at elucidating the genetic basis of host adaptation and the mechanisms underlying host shifts will deepen our understanding of fungal pathogenesis and inform more effective strategies for disease management in agricultural settings.

## Conclusion

This study successfully utilized whole-genome sequence comparisons to develop InDel markers for *Pyricularia oryzae*, the rice blast fungus, representing a significant advancement in identifying genetic variations. A total of 233,595 InDel loci were identified, from which 82 loci were selected for the genetic evaluation of Thai rice blast populations. These markers exhibited moderate informativeness and effectively differentiated isolates based on host specificity, particularly between rice and grassy weeds. The findings underscore the role of genetic diversity in marker development and demonstrate the potential of InDel markers for studying global genetic diversity. Additionally, the study highlights the importance of host–pathogen interactions and evolutionary dynamics, suggesting that host shifts contribute to pathogen specialization. Further research is required to uncover the genetic basis of host adaptation and to improve disease management strategies in agriculture.

## Materials and methods

### Rice blast fungus whole-genome sequence and sequence preparation

This study utilized whole-genome sequence datasets of *Pyricularia oryzae* collected from various global locations. The datasets included 131 isolates from a global population, as reported by Gladieux et al. (2018b) and Zhong et al. (2018), and were supplemented with an additional 21 isolates obtained from the National Center for Biotechnology Information (NCBI). These rice blast populations originated from seven distinct hosts within the Poaceae family, including *Oryza sativa*, *Triticum aestivum*, *Eleusine indica*, *Eleusine coracana*, perennial ryegrass, and *Urochloa brizantha*. Detailed information about the isolates is provided in Table S1. Sequence datasets were retrieved from the Sequence Read Archive (SRA) at NCBI. Raw reads were evaluated using the FastQC algorithm, and low-quality reads were removed using Trimmomatic 0.39 (Bolger et al. 2014). The resulting high-quality reads were mapped to the whole-genome sequence of the *P. oryzae* 70–15 strain (accession number GCA_000002495.2) using Burrows-Wheeler Aligner (BWA) 0.7.17 (Li and Durbin 2010). Mapped reads were sorted with Samtools 1.11 (Danecek et al. 2021). The MarkDuplicates command was applied to eliminate duplicate reads, and the AddOrReplaceReadGroups command, using Picard 2.20.8 (https://broadinstitute.github.io/picard/), was employed to assign all reads to a single new read group.

### InDel locus and variant identification

The Genome Analysis Toolkit (GATK) version 4.1.3.0 was used to identify InDel loci and variants among 152 rice blast isolates, using default settings. Modules such as HaplotypeCaller, GenotypeGVCFs, and VariantFiltration were employed for genomic variant genotyping and filtering according to the following parameters: QUAL < 50, QD < 5.0, MQ < 20.0, ReadPosRankSum < -20.0, and MQRankSum > 2.0. The resulting InDel sites were visually inspected using the Integrative Genomics Viewer (IGV). Polymorphic InDel loci with at least two variants per locus, each with a frequency greater than 0.05, were selected for primer design. To determine the genomic locations of these marker sequences in *P. oryzae* strain 70–15, we first identified the positions of insertions and deletions (InDels) that met the marker selection criteria. We then examined the regions 2,000 base pairs upstream and downstream of these positions. The genome annotation file was analyzed directly using bedtools software version 2.30.0 to identify conserved regions suitable for primer design (Supplementary Material 1). Locus-specific primers were designed using the Primer3 program (http://bioinfo.ut.ee/primer3-0.4.0/) (Table S2), and Primer-BLAST (https://www.ncbi.nlm.nih.gov/tools/primer-blast/) was employed to verify primer uniqueness through sequence verification.

### Thai rice blast fungus populations

**Fig. 7:**
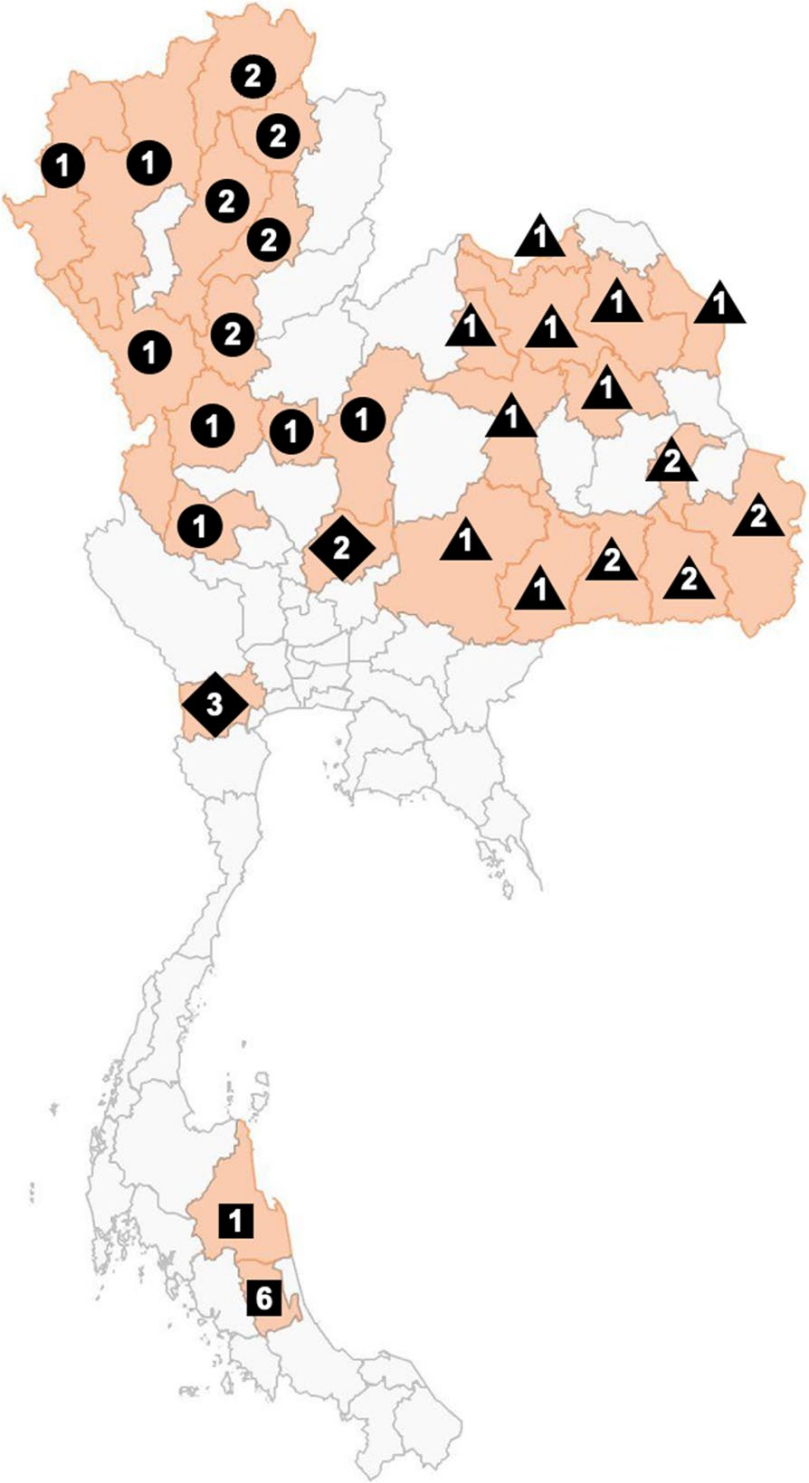
Geographic distribution of Thai rice blast fungal isolates collected for genetic diversity assessment. This figure displays the locations where rice blast isolates were collected for the study. The shapes represent the region, with circle, triangle, diamond, and square indicating northern, northeastern, central, and southern Thailand, respectively. The number inside each shape represents the total number of isolates collected from that province

Forty-seven rice blast fungal isolates were collected from the leaves of rice and grassy weeds across diverse regions of Thailand, including the central, northern, northeastern, and southern areas (Fig. 7). The sampling method, single-spore isolation, cultivation, and storage procedures were described by Sirisathaworn et al. (2017). Additionally, the reference isolates Guy11 (originally isolated from an infected rice plant in Guyana) and KJ201 (a Korean rice blast isolate obtained from the Center for Fungal Genetic Resources, Seoul National University) were used for genetic diversity analysis. Supplementary information regarding the isolates is provided in Table S3.

### DNA extraction and InDel analysis for genetic diversity

DNA samples were extracted from mycelium using the cetyltrimethylammonium bromide (CTAB) method, as described by Doyle and Doyle (1987). PCR reactions were performed in a total volume of 20 μL, containing 1 μL of 50 ng/μL DNA, 2 μL of 10X buffer A, 0.2 μL of 2.5 mM dNTPs, 0.6 μL of MgCl₂, 0.8 μL of 5 μM forward primer, 0.8 μL of 5 μM reverse primer (Table S3), 0.2 μL of 5 U/μL *Taq* polymerase (Vivantis, Shah Alam, Malaysia), and 14.4 μL of ddH₂O. PCR amplification was conducted under the following conditions: initial denaturation at 94 °C for 4 min, followed by 35 cycles of denaturation at 94 °C for 30 s, annealing at 55.0—59.0 °C for 30 s, and extension at 72 °C for 30 s. A final extension step was performed at 72 °C for 4 min. PCR products were visualized by electrophoresis on a 2% agarose gel in 0.5X TAE buffer and stained with a DNA staining solution containing 20,000X RedSafe™ (iNtRON, Korea). The resulting products were visualized using a Gel Documentation system (Axygen, CA, USA).

### Data analysis

To assess the genetic diversity within the 47 Thai rice blast isolates using the InDel markers developed in this study, the sizes of the amplified bands were scored to indicate the presence (1) or absence (0) of each variant. The Polymorphic Information Content (PIC) was calculated using the formula PIC = 1—∑xi^2^, where xi represents the frequency of the i^th^ allele at the InDel loci. To determine the genetic relationships among the isolates, genetic distance was calculated using the Jaccard similarity coefficient, and clustering was performed using the UPGMA algorithm. The R "pvclust" package (Version 1.3–2) was used to generate the phylogenetic tree. Confidence levels for the branches were determined by calculating multiscale bootstrap resampling p-values with 1,000 replications using the "pvclust" package in R (Suzuki et al. 2017). Genetic diversity parameters, including the number of alleles (Na), effective number of alleles (Ne), Shannon diversity index (I), Nei's gene diversity (h), and PCoA, were calculated using GenAlex V6.5 (Peakall and Smouse 2006, 2012). The genetic structure of the 49 rice blast isolates was analyzed using Structure 2.3.4 software. Subgroup numbers (ΔK) were evaluated for K values ranging from 1 to 10, with each K value assessed through 10,000 iterations of Markov Chain Monte Carlo (MCMC), repeated three times. The average value of lnP(D) was used for population estimation, and the optimal number of populations was determined using the maximum likelihood method. The corresponding K value was calculated, and the peak value of ΔK was identified using the StructureSelector program, following the method described by Li and Liu (2018).

## Supplementary Information

Additional file 1: Table S1. List of 152 rice blast isolates used for whole-genome sequence comparison with SRA ID, collection site and host. Table S2. Primer sequences of 82 developed rice blast InDel markers. Table S3. List of 47 Thai rice blast isolates and two reference isolates, GUY11 and KJ201 in the study including isolates name, location, host plant of collection and region of collected site in Thailand.

Additional file 2: Supplementary material 1. The genome sequence of *P. oryzae *for InDel marker development. Yellow highlight; Forward and reverse primer sequence, blue highlight; InDel position based on genome sequence of *P. oryzae *strain 70-15 (reference genome).

## Data Availability

All data generated or analyzed during this study are included in this published article and its Supplementary Information files. The whole-genome sequence datasets of *P. oryzae* used in InDel marker development are available in NCBI database using SRA numbers in Table S1.

## Abbreviations

InDel: Insertion and deletion
PIC: Polymorphic Information Content
PCoA: Principal coordinate analysis
UPGMA: Unweighted pair group method with arithmetic mean
