# Supplementary material for: Development of novel InDel markers by whole-genome sequence comparison and genetic diversity assessment of Thailand rice blast fungus populations
Source: Stress Biol. 2025 Apr 27;5(1):27. doi: 10.1007/s44154-025-00212-1 (PMC12034604; doi:10.1007/s44154-025-00212-1)
Supplement: Supplementary file 1 — Additional file 1: Table S1. List of 152 rice blast isolates used for whole-genome sequence comparison with SRA ID, collection site and host. Table S2. Primer sequences of 82 developed rice blast InDel markers. Table S3. List of 47 Thai rice blast isolates and two reference isolates, GUY11 and KJ201 in the study including isolates name, location, host plant of collection and region of collected site in Thailand. [file 44154_2025_212_MOESM1_ESM.docx]

Article: Development of novel InDel markers by whole-genome sequence comparison and genetic diversity assessment of Thailand rice blast fungus populations

**Journal:** Stress biology

Napassorn Thamkirati ^1^, Worrawit Suktrakul ^1^, Athipat Ngernmuen ^2^, Theerayut Toojinda ^3^, Sureeporn Kate-ngam ^4^, Nonglak Parinthawong ^5^, Waree Laophermsuk ^5^, Pradipha Pradapphai ^5^, Watchareeporn Suksiri ^5^, Suphattra Janthasri ^5^ and Chatchawan Jantasuriyarat ^1,6*^

^1^ Department of Genetics, Faculty of Science, Kasetsart University, Bangkok, 10900, Thailand.

[napassorn.thamk@ku.th](mailto:napassorn.thamk@ku.th); [worrawit.s@ku.th](mailto:worrawit.s@ku.th); [fscicwj@ku.ac.th](mailto:fscicwj@ku.ac.th)

^2^ Department of Zoology, Faculty of Science, Kasetsart University, Bangkok, 10900, Thailand.

[athipat.ng@ku.th](mailto:athipat.ng@ku.th)

^3^ National Center for Genetic Engineering and Biotechnology (BIOTEC), 113 Thailand Science Park, PathumThani, 12120, Thailand.

[theerayuttoojinda638@gmail.com](mailto:theerayuttoojinda638@gmail.com)

^4^ Agronomy Department, Faculty of Agriculture, Ubon Ratchathani University, Ubon Ratchathani, 34190, Thailand.

[sureeporn.k@ubu.ac.th](mailto:sureeporn.k@ubu.ac.th)

^5^ Department of Plant Production Technology, School of Agricultural Technology, King Mongkut’s Institute of Technology Ladkrabang, Bangkok, 10520

[nonglak.pa@kmitl.ac.th](mailto:nonglak.pa@kmitl.ac.th); [waree_pla@hotmail.com](mailto:waree_pla@hotmail.com); [jane3957@hotmail.com](mailto:jane3957@hotmail.com); [june_26709@hotmail.com](mailto:june_26709@hotmail.com); [nutty_suphattra@hotmail.com](mailto:nutty_suphattra@hotmail.com)

^6^ Center for Advanced Studies in Tropical Natural Resources, National Research University-Kasetsart (CASTNAR, NRU-KU), Kasetsart University, Bangkok, 10900, Thailand.

[fscicwj@ku.ac.th](mailto:fscicwj@ku.ac.th)

*Correspondence: fscicwj@ku.ac.th

**Table S1.** List of 152 rice blast isolates used for whole-genome sequence comparison with SRA ID, collection site and host

| **No.** | **Genome sequence name** | **SRA** | **Genbank/Reference** | **Collection site** | **Host** |
| --- | --- | --- | --- | --- | --- |
| 1 | 10100 | SRR7503877 | GCA_003991345.1 | Thailand | *Oryza sativa* |
| 2 | 13FM-16-1 | SRR6669189 | Zhong et al., 2018 | China | *Oryza sativa* |
| 3 | 13FM-24-1 | SRR6669190 | Zhong et al., 2018 | China | *Oryza sativa* |
| 4 | 13FM-3-2 | SRR6669187 | Zhong et al., 2018 | China | *Oryza sativa* |
| 5 | 13FM-5-1 | SRR6669188 | Zhong et al., 2018 | China | *Oryza sativa* |
| 6 | 13FM-9-1 | SRR6669193 | Zhong et al., 2018 | China | *Oryza sativa* |
| 7 | AV1-1-1 | SRR6669194 | Zhong et al., 2018 | China | *Oryza sativa* |
| 8 | B157 | SRR1747212 | N/A | India | *Oryza sativa* |
| 9 | B71 | SRR6232156 | GCA_946468025.1 | Brazil | *Triticum aestivum* |
| 10 | BD0024 | SRR6384793 | Gladieux et al., 2018 | Burundi | *Oryza sativa* |
| 11 | BJ-90-611 | SRR6669192 | Zhong et al., 2018 | China | *Oryza sativa* |
| 12 | BJ08-8-1 | SRR6669191 | Zhong et al., 2018 | China | *Oryza sativa* |
| 13 | BR0026 | SRR6384792 | Gladieux et al., 2018 | Brazil | *Oryza sativa* |
| 14 | CD0073 | SRR6384795 | Gladieux et al., 2018 | cote d'ivoire | *Oryza sativa* |
| 15 | CD0203 | SRR6384794 | Gladieux et al., 2018 | cote d'ivoire | *Oryza sativa* |
| 16 | CH0052 | SRR6384789 | Gladieux et al., 2018 | China | *Oryza sativa* |
| 17 | CH0063 | SRR6384788 | Gladieux et al., 2018 | China | *Oryza sativa* |
| 18 | CH0092 | SRR6384791 | Gladieux et al., 2018 | China | *Oryza sativa* |
| 19 | CH0328 | SRR6384790 | Gladieux et al., 2018 | China | *Oryza sativa* |
| 20 | CH0333 | SRR6384797 | Gladieux et al., 2018 | China | *Oryza sativa* |
| 21 | CH0532 | SRR6384796 | Gladieux et al., 2018 | China | *Oryza sativa* |
| 22 | CH0549 | SRR6384784 | Gladieux et al., 2018 | China | *Oryza sativa* |
| 23 | CH0595 | SRR6384783 | Gladieux et al., 2018 | China | *Oryza sativa* |
| 24 | CH0680 | SRR6384782 | Gladieux et al., 2018 | China | *Oryza sativa* |
| 25 | CH0689 | SRR6384781 | Gladieux et al., 2018 | China | *Oryza sativa* |
| 26 | CH0701 | SRR6384780 | Gladieux et al., 2018 | China | *Oryza sativa* |
| 27 | CH0860 | SRR6384779 | Gladieux et al., 2018 | China | *Oryza sativa* |
| 28 | CH0999 | SRR6384778 | Gladieux et al., 2018 | China | *Oryza sativa* |
| 29 | CH1016 | SRR6384777 | Gladieux et al., 2018 | China | *Oryza sativa* |
| 30 | CH1019 | SRR6384787 | Gladieux et al., 2018 | China | *Oryza sativa* |
| 31 | CL0026 | SRR6384786 | Gladieux et al., 2018 | Colombia | *Oryza sativa* |
| 32 | CL3-6-7 | SRR6384808 | Gladieux et al., 2018 | Colombia | *Oryza sativa* |
| 33 | DB11-621 | SRR6669195 | Zhong et al., 2018 | China | *Oryza sativa* |
| 34 | EI9411 | SRR11836441 | GCA_001548775.1 | China | *Eleusine indica* |
| 35 | EI9604 | SRR11836442 | GCA_001548785.1 | China | *Eleusine indica* |
| 36 | FJ0204804 | SRR6669196 | Zhong et al., 2018 | China | *Oryza sativa* |
| 37 | FJ11SH-527-1 | SRR6669217 | Zhong et al., 2018 | China | *Oryza sativa* |
| 38 | FJ11YD-673-1 | SRR6669218 | Zhong et al., 2018 | China | *Oryza sativa* |
| 39 | FJ12JN-084-3 | SRR6669215 | Zhong et al., 2018 | China | *Oryza sativa* |
| 40 | FJ13SH05-2 | SRR6669216 | Zhong et al., 2018 | China | *Oryza sativa* |
| 41 | FJ2000-62A | SRR6669221 | Zhong et al., 2018 | China | *Oryza sativa* |
| 42 | FJ2000-69A | SRR6669222 | Zhong et al., 2018 | China | *Oryza sativa* |
| 43 | FJ2001-112B | SRR6669219 | Zhong et al., 2018 | China | *Oryza sativa* |
| 44 | FJ2003-001A1 | SRR6669220 | Zhong et al., 2018 | China | *Oryza sativa* |
| 45 | FJ2005113B | SRR6669223 | Zhong et al., 2018 | China | *Oryza sativa* |
| 46 | FJ2006-60A3 | SRR6669224 | Zhong et al., 2018 | China | *Oryza sativa* |
| 47 | FJ43ZB15-68 | SRR6669182 | Zhong et al., 2018 | China | *Oryza sativa* |
| 48 | FJ72ZC7-77 | SRR6669181 | Zhong et al., 2018 | China | *Oryza sativa* |
| 49 | FJ78-JJ | SRR6669180 | Zhong et al., 2018 | China | *Oryza sativa* |
| 50 | FJ81-JY | SRR6669186 | Zhong et al., 2018 | China | *Oryza sativa* |
| 51 | FJ81-MH | SRR6669185 | Zhong et al., 2018 | China | *Oryza sativa* |
| 52 | FJ81-SW | SRR6669184 | Zhong et al., 2018 | China | *Oryza sativa* |
| 53 | FJ81-ZP | SRR6669183 | Zhong et al., 2018 | China | *Oryza sativa* |
| 54 | FJ81221ZB11-14 | SRR6669179 | Zhong et al., 2018 | China | *Oryza sativa* |
| 55 | FJ81278 | SRR16282279 | GCA_002368515.1 | China | *Oryza sativa* |
| 56 | FJ86-CT | SRR6669177 | Zhong et al., 2018 | China | *Oryza sativa* |
| 57 | FJ86061ZE3-39 | SRR6669178 | Zhong et al., 2018 | China | *Oryza sativa* |
| 58 | FJ95054B | SRR6669201 | Zhong et al., 2018 | China | *Oryza sativa* |
| 59 | FJ95085AZB13-116 | SRR6669202 | Zhong et al., 2018 | China | *Oryza sativa* |
| 60 | FJ98099 | SRR6669203 | Zhong et al., 2018 | China | *Oryza sativa* |
| 61 | FJ99138 | SRR6669204 | Zhong et al., 2018 | China | *Oryza sativa* |
| 62 | FJSH0703 | SRR6669197 | Zhong et al., 2018 | China | *Oryza sativa* |
| 63 | GD-05-029b | SRR6669198 | Zhong et al., 2018 | China | *Oryza sativa* |
| 64 | GD06-53 | SRR6669199 | Zhong et al., 2018 | China | *Oryza sativa* |
| 65 | GD08-2025 | SRR6669200 | Zhong et al., 2018 | China | *Oryza sativa* |
| 66 | Guy11 | SRR16282278 | GCA_002925095.1 | France | *Oryza sativa* |
| 67 | HB-14 | SRR6669205 | Zhong et al., 2018 | China | *Oryza sativa* |
| 68 | HB-LTH18 | SRR6669206 | Zhong et al., 2018 | China | *Oryza sativa* |
| 69 | HN-0812-3 | SRR6669164 | Zhong et al., 2018 | China | *Oryza sativa* |
| 70 | HN-158 | SRR6669166 | Zhong et al., 2018 | China | *Oryza sativa* |
| 71 | HN0001 | SRR6384809 | Gladieux et al., 2018 | Hungary | *Oryza sativa* |
| 72 | HN10-1604 | SRR6669163 | Zhong et al., 2018 | China | *Oryza sativa* |
| 73 | IN0072 | SRR6384806 | Gladieux et al., 2018 | India | *Oryza sativa* |
| 74 | IN0082 | SRR6384807 | Gladieux et al., 2018 | India | *Oryza sativa* |
| 75 | IN0094 | SRR6384804 | Gladieux et al., 2018 | India | *Oryza sativa* |
| 76 | JP0010 | SRR6384805 | Gladieux et al., 2018 | Japan | *Oryza sativa* |
| 77 | JRBC01 | SRR1561422 | GCA_000805855.1 | China | *Oryza sativa* |
| 78 | JS-10-6-1-2 | SRR6669159 | Zhong et al., 2018 | China | *Oryza sativa* |
| 79 | JS08-611 | SRR6669165 | Zhong et al., 2018 | China | *Oryza sativa* |
| 80 | JS09-138 | SRR6669160 | Zhong et al., 2018 | China | *Oryza sativa* |
| 81 | JUM1 | ERR2660287 | GCA_905109865.1 | The United Kingdom | *Oryza sativa* |
| 82 | JX-09Z116-1 | SRR6669162 | Zhong et al., 2018 | China | *Oryza sativa* |
| 83 | JX10-102 | SRR6669161 | Zhong et al., 2018 | China | *Oryza sativa* |
| 84 | JX11-141 | SRR6669158 | Zhong et al., 2018 | China | *Oryza sativa* |
| 85 | KA1-3-1 | SRR6669157 | Zhong et al., 2018 | Ghana | *Oryza sativa* |
| 86 | KA2-1-1 | SRR6669175 | Zhong et al., 2018 | Ghana | *Oryza sativa* |
| 87 | KJ201 | SRS4090462 | GCA_000376685.2 | Korea | *Oryza sativa* |
| 88 | LA0005 | SRR6384802 | Gladieux et al., 2018 | Laos | *Oryza sativa* |
| 89 | LA0021 | SRR6384803 | Gladieux et al., 2018 | Laos | *Oryza sativa* |
| 90 | LpKY97 | SRR11553671 | GCA_012272995.1 | America | *Perennial ryegrass* |
| 91 | MC0016 | SRR6384800 | Gladieux et al., 2018 | Morocco | *Oryza sativa* |
| 92 | MD0929 | SRR6384801 | Gladieux et al., 2018 | Madagascar | *Oryza sativa* |
| 93 | MG01 | SRR1745845 | GCA_000969745.1 | India | *Oryza sativa* |
| 94 | ML0025 | SRR6384774 | Gladieux et al., 2018 | Mali | *Oryza sativa* |
| 95 | MZ5-1-6 | SRR8258942 | GCA_004346965.1 | Japan | *Eleusine coracana* |
| 96 | Nich-2-3-2 | SRR6669176 | Zhong et al., 2018 | Suriname | *Oryza sativa* |
| 97 | Nich-2-7-4 | SRR6669173 | Zhong et al., 2018 | Suriname | *Oryza sativa* |
| 98 | NP0037 | SRR6384773 | Gladieux et al., 2018 | Nepal | *Oryza sativa* |
| 99 | NP0041 | SRR6384776 | Gladieux et al., 2018 | Nepal | *Oryza sativa* |
| 100 | NP0052 | SRR6384775 | Gladieux et al., 2018 | Nepal | *Oryza sativa* |
| 101 | NP0061 | SRR6384770 | Gladieux et al., 2018 | Nepal | *Oryza sativa* |
| 102 | P131 | SRR24890911 | GCA_000292605.2 | Japan | *Oryza sativa* |
| 103 | PH0103 | SRR6384769 | Gladieux et al., 2018 | Philippines | *Oryza sativa* |
| 104 | PH0118 | SRR6384772 | Gladieux et al., 2018 | Philippines | *Oryza sativa* |
| 105 | PR0009 | SRR6384771 | Gladieux et al., 2018 | Portugal | *Oryza sativa* |
| 106 | Sar-2-20-1 | SRR6669174 | Zhong et al., 2018 | Suriname | *Oryza sativa* |
| 107 | Sar-AD3-5 | SRR6669171 | Zhong et al., 2018 | Suriname | *Oryza sativa* |
| 108 | SC-10-120-65-2 | SRR6669172 | Zhong et al., 2018 | China | *Oryza sativa* |
| 109 | SC-10-25-44-1 | SRR6669169 | Zhong et al., 2018 | China | *Oryza sativa* |
| 110 | SP0005 | SRR6384768 | Gladieux et al., 2018 | Spain | *Oryza sativa* |
| 111 | T21 | SRR23388169 | GCA_033875275.1 | Brazil | *Triticum aestivum* |
| 112 | T29 | SRR23400923 | GCA_031208665.1 | Brazil | *Triticum aestivum* |
| 113 | TH0017 | SRR6384798 | Gladieux et al., 2018 | Thailand | *Oryza sativa* |
| 114 | TW-1-1-1-B-1 | SRR6669170 | Zhong et al., 2018 | Taiwan | *Oryza sativa* |
| 115 | TW-12CY-TB1-2 | SRR6669167 | Zhong et al., 2018 | Taiwan | *Oryza sativa* |
| 116 | TW-12HL-DF1-2 | SRR6669168 | Zhong et al., 2018 | Taiwan | *Oryza sativa* |
| 117 | TW-12HL-YL2-1 | SRR6669144 | Zhong et al., 2018 | Taiwan | *Oryza sativa* |
| 118 | TW-12TD-RH1-1 | SRR6669143 | Zhong et al., 2018 | Taiwan | *Oryza sativa* |
| 119 | TW-12TN-HB2-2 | SRR6669142 | Zhong et al., 2018 | Taiwan | *Oryza sativa* |
| 120 | TW-12YL-DL3-2 | SRR6669141 | Zhong et al., 2018 | Taiwan | *Oryza sativa* |
| 121 | TW-12YL-DP1-1 | SRR6669140 | Zhong et al., 2018 | Taiwan | *Oryza sativa* |
| 122 | TW-12YL-TT4-1 | SRR6669139 | Zhong et al., 2018 | Taiwan | *Oryza sativa* |
| 123 | TW-2-7-2-A-1 | SRR6669138 | Zhong et al., 2018 | Taiwan | *Oryza sativa* |
| 124 | TW-6-2-2-B-1 | SRR6669137 | Zhong et al., 2018 | Taiwan | *Oryza sativa* |
| 125 | TW-6-43-1 | SRR6669146 | Zhong et al., 2018 | Taiwan | *Oryza sativa* |
| 126 | TW-CYBP1-3 | SRR6669145 | Zhong et al., 2018 | Taiwan | *Oryza sativa* |
| 127 | TW-PT1-1 | SRR6669147 | Zhong et al., 2018 | Taiwan | *Oryza sativa* |
| 128 | TW-PT3-1 | SRR6669148 | Zhong et al., 2018 | Taiwan | *Oryza sativa* |
| 129 | TW-PT6-1 | SRR6669149 | Zhong et al., 2018 | Taiwan | *Oryza sativa* |
| 130 | TW-TN4-2 | SRR6669150 | Zhong et al., 2018 | Taiwan | *Oryza sativa* |
| 131 | UbJA110 | SRR23498602 | GCA_031466495.1 | Brazil | *Urochloa brizantha* |
| 132 | UbJA174 | SRR23404000 | GCA_033875295.1 | Brazil | *Urochloa brizantha* |
| 133 | UbJA92 | SRR14705986 | N/A | Brazil | *Triticum aestivum* |
| 134 | US0031 | SRR6384799 | Gladieux et al., 2018 | The United States | *Oryza sativa* |
| 135 | US0098 | SRR6384785 | Gladieux et al., 2018 | The United States | *Oryza sativa* |
| 136 | V86010 | SRR16280147 | GCA_002105295.1 | Philippines | *Oryza sativa* |
| 137 | WB127 | SRR14705989 | N/A | Brazil | *Triticum aestivum* |
| 138 | WD-3-1 | SRR6669151 | Zhong et al., 2018 | Ghana | *Oryza sativa* |
| 139 | YN07205e | SRR6669152 | Zhong et al., 2018 | China | *Oryza sativa* |
| 140 | YN072310 | SRR6669153 | Zhong et al., 2018 | China | *Oryza sativa* |
| 141 | YN072311 | SRR6669154 | Zhong et al., 2018 | China | *Oryza sativa* |
| 142 | YN072313 | SRR6669155 | Zhong et al., 2018 | China | *Oryza sativa* |
| 143 | YN08181e | SRR6669156 | Zhong et al., 2018 | China | *Oryza sativa* |
| 144 | YN08182c | SRR6669208 | Zhong et al., 2018 | China | *Oryza sativa* |
| 145 | YN126311 | SRR6669207 | Zhong et al., 2018 | China | *Oryza sativa* |
| 146 | YN126441 | SRR6669210 | Zhong et al., 2018 | China | *Oryza sativa* |
| 147 | YN8773-19 | SRR6669209 | Zhong et al., 2018 | China | *Oryza sativa* |
| 148 | YN8773R-27 | SRR6669212 | Zhong et al., 2018 | China | *Oryza sativa* |
| 149 | ZJ00-10 | SRR6669211 | Zhong et al., 2018 | China | *Oryza sativa* |
| 150 | ZJ08-41 | SRR6669214 | Zhong et al., 2018 | China | *Oryza sativa* |
| 151 | ZJ2011-7-1 | SRR6669213 | Zhong et al., 2018 | China | *Oryza sativa* |
| 152 | ZM1-2 | SRR19903160 | GCA_026261775.1 | Zambia | *Triticum aestivum* |

SRA; Sequence Read Archive data

**Table S2.** Primer sequences of 82 developed rice blast InDel markers

| No. | Chrom | Name | Primer Sequences | | Tm | Expected Size  (bp) |
| --- | --- | --- | --- | --- | --- | --- |
|  |  |  | Forward primer | Reverse primer |  |  |
| 1 | 1 | MD101 | GTTGAGCCAAGTTTCACGTC | GATGTGAGCGTTCTGAGGAT | 55 | 293 |
| 2 | 1 | MD102 | GGTATGCAGTGCAGAAACTG | ATACACCTCGACTGCATCTG | 55 | 292 |
| 3 | 1 | MD105 | GGCTTGGTAGTGAGATCGAG | AGTAAGGATGGTGGGGAAAC | 55 | 298 |
| 4 | 1 | MD106 | ACAAGGACAAGTGGGTGTTG | GTTCCTGTGATTCGGTTACG | 59 | 280 |
| 5 | 1 | MD107 | GCCTACGTTCTCTGGTTCTT | GGTTCCTGTGATTCGGTTAC | 55 | 198 |
| 6 | 1 | MD108 | CGTGTCTCTGAGTGGAGTTT | CGAGATAGATACCACGTTGC | 59 | 286 |
| 7 | 1 | MD109 | ATCTTAGCGCAGCATGTACC | CGGGTTTCTGGGTCTTAGAG | 55 | 236 |
| 8 | 1 | MD110 | AGTGACTGGACCAAGGAGAA | GGATATGGGCTTCAGGACTT | 59 | 202 |
| 9 | 1 | MD112 | CGCAAGTAGTGCAAGATTGG | GGGCTGTTTGTTAGCTTGGT | 59 | 181 |
| 10 | 1 | MD113 | AGAGGTCAACCTTCACTGGT | GATGAGGCAGCCCTATAGTT | 55 | 274 |
| 11 | 1 | MD114 | CCACCCGAGTCATAGAGATG | CTCTGATCTACGGCTGAAGG | 55 | 397 |
| 12 | 1 | MD115 | GTATGTTGTGATGGCCTTGC | TACTCAAATGAGGCGCAGTC | 55 | 196 |
| 13 | 1 | MD116 | CCTTGTCCCCTCCTGATTT | GTGGATGGGAGACCATTTG | 55 | 253 |
| 14 | 1 | MD117 | CGAGCAGTAAAGTCAGACCA | GCAGTGCAGACGAAGACTAC | 55 | 247 |
| 15 | 1 | MD118 | AGCTCTCCCTCGAAGTCATA | CAATCTGTACTCCGTGCCTA | 55 | 108 |
| 16 | 1 | MD119 | GATTTGCACCTGGGTATGAC | GCCAAGTCCTCCTGATATTG | 59 | 263 |
| 17 | 1 | MD120 | AATACACGTTGCCACCAGTC | GCGCCTGAGTTATGACATCT | 59 | 240 |
| 18 | 2 | MD201 | TGGGATCTTCGGTAAGACG | GCTCTTTACAACCACCGTCA | 55 | 253 |
| 19 | 2 | MD202 | CGATAGCAGTTGGGTAGGTC | AAGCCAAGCACAGAGAAGAG | 59 | 105 |
| 20 | 2 | MD203 | CGCTCCTTCAGTCCTGATAA | TAAGGCTGGTTACGGGAGTA | 55 | 242 |
| 21 | 2 | MD204 | TCCAAGCTTCTAGGGAAACC | CGCCTAAGGTGGCTTTTAGT | 55 | 291 |
| 22 | 2 | MD205 | TCGGACAGTAGATTGAGTGC | CTCGGCATCACACATTAGAG | 55 | 281 |
| 23 | 2 | MD206 | CCCTTGCTCCCTTCTTACAT | CGTGGAGATCACACACACAC | 59 | 97 |
| 24 | 2 | MD207 | GAGCTGTACGTGGTGAATGG | GAGCGGATCCAAAGTCAGTT | 55 | 236 |
| 25 | 2 | MD208 | CACAGGATGCAAGTGACTGA | AGGGGAAGAAGACTGGCTTA | 55 | 278 |
| 26 | 2 | MD209 | ACTCGGCTGCACATACTCAT | GTTGTTCGTCACACCCTTGT | 55 | 104 |
| 27 | 2 | MD210 | GAGTCGTCATCCACAGATTC | TGGTAATCTCTCCTGTCTGG | 55 | 273 |
| 28 | 2 | MD211 | CCGCAGTACTACCTGCTCTT | AGTCCTGGGACATGTTGTGT | 55 | 257 |
| 29 | 2 | MD212 | CTGCGTATCCATACCATCCT | AAGCCGTACAGACCAGACAG | 55 | 290 |
| 30 | 2 | MD213 | GGTGTCTCGACCTACGAAGT | GGTCCTGATTATGCGTACCT | 55 | 179 |
| 31 | 2 | MD214 | GCGTTCTTCGGTAGTAGGAG | GCGTTGCCTCTAGTACCTCT | 55 | 281 |
| 32 | 2 | MD215 | CCTGTACCAAAGGCACAATG | ACCCAGTCATCCGGAGTAAG | 55 | 157 |
| 33 | 2 | MD217 | AACCAACGACAGTCGACAAC | CGAGTAGCACAGAGCAAGGT | 55 | 297 |
| 34 | 2 | MD218 | AGTAGTCCGAGGGGTGTTGT | CAACTCCCAATACTCGGCTA | 55 | 289 |
| 35 | 2 | MD219 | CAACAGCAGCCATAGAGAAG | ATCGAGTCGGTAGAAAGGAG | 55 | 236 |
| 36 | 2 | MD220 | AAACAAGAACAGGGGTCGTC | CACTCATCCTCCTGGGACTT | 59 | 240 |
| 37 | 3 | MD301 | AAGTCCCCTTTCCTCCTTAC | ACTGGAGGGTTTCGGTTAG | 55 | 91 |
| 38 | 3 | MD302 | TCCCTTTCTGGACAGGACT | GCAATTAACGGGGTACACTC | 55 | 244 |
| 39 | 3 | MD303 | GTCTTGGCATGATCGTAAGG | ACTGCATCAAGGGTCCTGTA | 59 | 295 |
| 40 | 3 | MD304 | GTCTTGGCATGATCGTAAGG | ACTGCATCAAGGGTCCTGTA | 55 | 295 |
| 41 | 3 | MD306 | ACCGGTGAGTAACAAGAGTG | GCTTGAGTTGAAGTGTCAGG | 55 | 184 |
| 42 | 3 | MD307 | GGTCGTGAGGGTTCCTATCT | CGCTTCCTTCCTCCTATCTC | 55 | 196 |
| 43 | 3 | MD308 | TTACCCTTGTCCTTGTGCTC | GATTGCAGTGCAGTAAGCTG | 59 | 299 |
| 44 | 3 | MD309 | ATTCATAGGGCCAACTGGAG | GACTGGACTGGATTGCCTCT | 59 | 269 |
| 45 | 3 | MD310 | TCCCAGACTTGTTGACTGGT | TCCGACTAGGACGCAAGTT | 55 | 267 |
| 46 | 3 | MD312 | TGGATGGCCTCATCTACTTC | GACAAGGTTGGTGCTTCTTC | 59 | 286 |
| 47 | 3 | MD313 | GCTTCCCTGAGCGACTTTAT | CCTCAAGGTTCTGGTTGGAT | 55 | 158 |
| 48 | 3 | MD315 | GAGATGAGCCCAAGTCAAAC | ACTCAGCCCCTCCACTTTAT | 59 | 145 |
| 49 | 3 | MD316 | GCTAGCTCTGCCTTCTGTTT | GCACTGATCAACGTCTTGAG | 55 | 293 |
| 50 | 3 | MD317 | TAGCTCTGCCTTCTGTTTGG | GGTGCACTGATCAACGTCTT | 55 | 293 |
| 51 | 3 | MD318 | CTTCCTAGTCCCTTGAGCA | CCACTTGACCACTCAAAGAC | 55 | 254 |
| 52 | 4 | MD401 | ACAGCAGCAGGACTGATTAC | CTTTGACAGCTGAGATAGGC | 55 | 131 |
| 53 | 4 | MD402 | GGGTACACTAAGCTGCTACG | CAGCACAAGATGGCAGTT | 55 | 250 |
| 54 | 4 | MD403 | GTCTCCTTTTGTCTGCTCTG | GGGGTATGTATGTCCAGTTG | 55 | 277 |
| 55 | 4 | MD404 | CGAGTATGTTGTACGGAACC | CTAGGCCTCGCTAGAACAA | 55 | 192 |
| 56 | 4 | MD405 | CCGGTATACGTAATGCAGAG | GAGAGGTTGGTCTGTTACGA | 55 | 271 |
| 57 | 4 | MD406 | CAGCACCTGCTATTGTGTC | GAGGCTCCGTCTTTGTTT | 55 | 216 |
| 58 | 4 | MD408 | GTGGTTCGGTTTGAGAGAG | GGAGGGTCAGACTGAGTACA | 55 | 347 |
| 59 | 4 | MD409 | CGGACCTTGGATGAAGAAG | GTGAACTGCCCTATGACCTG | 59 | 220 |
| 60 | 4 | MD410 | GTGGGGGCACTGTTTATT | GAGAGTGGGATTGCTCATC | 55 | 263 |
| 61 | 4 | MD411 | CTTCTCTGTCGCTGTCTCTC | GGACCAAGCTCTATGTGGTA | 55 | 264 |
| 62 | 5 | MD501 | GAAGGGCAAACTCAAGGTAG | GATACGTCCATGGCTCAAG | 55 | 131 |
| 63 | 5 | MD502 | GTCCCAGTTGTCAACCTCCT | CGAGACTTCGACGTTTACGA | 55 | 282 |
| 64 | 5 | MD503 | GATAGACGAGTGAGGGGAAA | GGAGGTTGGGGTAAAAGAG | 55 | 257 |
| 65 | 5 | MD504 | CCACCTATGTAACCATCGAC | GGGCCTAATAAAGCCTCTC | 55 | 106 |
| 66 | 5 | MD505 | GATGGAAAGGGCTCAAGT | CTTGGCTCTCTTCTGTTCAC | 55 | 167 |
| 67 | 5 | MD506 | CCAGCAAGGATGGAGTTACA | ACGAACCTCCAAACCCATAC | 55 | 217 |
| 68 | 5 | MD507 | CCAGGGCGAGTCTAATACTG | TGTCTGTGATCGGTTAGTCG | 55 | 287 |
| 69 | 5 | MD508 | GTTACATGGTCGTCTTGTCC | GTCAGCCGCATTAGTCTTC | 55 | 296 |
| 70 | 5 | MD509 | GGATGATGAATGGCTGTG | AGGTGCTTCTTGGAGACTG | 55 | 166 |
| 71 | 5 | MD510 | CATGCTTGTCAGTCCCTAGT | GTCTGGCTTGTCTGAATCTG | 55 | 161 |
| 72 | 5 | MD511 | CCAGCTTGGATGGTGAGTT | CCCCCTATACGACCAGAGTT | 59 | 157 |
| 73 | 5 | MD512 | AAATCCCTACCAGGAGACAG | CCATGTTCTAAGGTGCTGAG | 55 | 289 |
| 74 | 6 | MD603 | GTGGACCTACATCGTTTCG | TCCTAGGCACTCTCCATTG | 59 | 170 |
| 75 | 6 | MD604 | TTTGCGATGGGTGTTACG | GCCAAGAGGAGGTGATCATT | 55 | 330 |
| 76 | 6 | MD605 | GCTTGAGGTCCTATGTGCT | GCTTAATACGGTCCTCCTGT | 55 | 240 |
| 77 | 6 | MD606 | GATCGTGGAGGGAAGGAGTA | TGGCAGCTTGCTGGATAA | 59 | 168 |
| 78 | 6 | MD607 | CGGGTGGATTGTGTTCAGT | CACTTTGAAGACCGCGAAC | 55 | 294 |
| 79 | 7 | MD701 | CCGTAAATGGACAGCGTTAC | TCAGCAAGCTTACACCACCT | 55 | 406 |
| 80 | 7 | MD702 | ATTCTTGGTCTTGGGTCTCG | CTAGTGCGAGCGCTGATATG | 55 | 198 |
| 81 | 7 | MD703 | CCGTAGTTATGATCCAGACG | AGTCTAAAAGGGACCACCTG | 59 | 276 |
| 82 | 7 | MD704 | ACGAGGTTCACCTCTTTTGC | CGCACATGGTCCTCACTATC | 59 | 176 |

**Table S3.** List of 47 Thai rice blast isolates and two reference isolates, GUY11 and KJ201 in the study including isolates name, location, host plant of collection and region of collected site in Thailand.

| No. | Isolate Name | Region of Thailand | Location of Collection | Elevation (m amsl) | Latitude | Longtitude | Host plant |
| --- | --- | --- | --- | --- | --- | --- | --- |
| 1 | PNB61008 | N | Phetchabun | 699 | 16.5661 | 100.901 | rice |
| 2 | YST61001 | NE | Yasothon | NA | 16.063800 | 104.361000 | rice |
| 3 | UTI61102 | N | Uthai Thani | 37 | 15.3402 | 99.9402 | grass |
| 4 | RBR61109 | C | Ratchaburi | 81 | 13.64261 | 99.506225 | grass |
| 5 | STI61004 | N | Sukhothai | 62 | 17.3324 | 99.8265 | rice |
| 6 | PYO61008 | N | Phayao | 303 | 19.148333 | 100.275 | rice |
| 7 | PRE61014 | N | Phrae | 192 | 18.191517 | 99.93403 | rice |
| 8 | PYO61002 | N | Phayao | 303 | 19.148333 | 100.275 | rice |
| 9 | PRE61021 | N | Phrae | 192 | 18.191517 | 99.93403 | rice |
| 10 | TAK61001 | N | Tak | 104 | 16.82336 | 99.15623 | rice |
| 11 | SSK61003 | NE | Sisaket | 154 | 14.686500 | 104.577000 | rice |
| 12 | SRN61004 | NE | Surin | 160 | 14.795300 | 103.450000 | rice |
| 13 | RBR61001 | C | Ratchaburi | 7 | 13.488812 | 99.790862 | rice |
| 14 | STI61005 | N | Sukhothai | 62 | 17.3324 | 99.8265 | rice |
| 15 | LPG61011 | N | Lampang | 234 | 18.28882 | 99.41045 | rice |
| 16 | PL61001 | S | Phatthalung | 14 | 7.566132 | 100.126538 | rice |
| 17 | PL61009 | S | Phatthalung | 14 | 7.566132 | 100.126538 | rice |
| 18 | KSN61003 | NE | Kalasin | 149 | 16.304800 | 103.438000 | rice |
| 19 | NST61103 | S | Nakhon Si Thammarat | 8 | 8.195634 | 100.11169 | grass |
| 20 | NKI61010 | NE | Nong Khai | 174 | 17.743300 | 102.685000 | rice |
| 21 | NBP61001 | NE | Nong Bua Lamphu | 249 | 17.321500 | 102.207000 | rice |
| 22 | LPG61004 | N | Lampang | 229 | 18.16096 | 99.43698 | rice |
| 23 | CRI61007 | N | Chiang Rai | 406 | 19.53095 | 99.740168 | rice |
| 24 | MSN61008 | N | Mae Hong Son | 195 | 19.3126 | 97.9589 | rice |
| 25 | KPT61002 | N | Kamphaeng Phet | 59 | 16.052 | 99.8494 | rice |
| 26 | YST61004 | NE | Yasothon | 168 | 16.304300 | 104.519000 | rice |
| 27 | UBN61011 | NE | Ubon Ratchathani | 124 | 15.341100 | 104.701000 | rice |
| 28 | KKN61005 | NE | Khon Kaen | 168 | 15.788300 | 102.607000 | rice |
| 29 | LRI61002 | C | Lopburi | 14 | 14.855 | 100.472 | rice |
| 30 | PL61017 | S | Phatthalung | 14 | 7.566132 | 100.126538 | rice |
| 31 | CRI61002 | N | Chiang Rai | NA | 19.884954 | 99.915992 | rice |
| 32 | LRI61104 | C | Lopburi | 14 | 14.855 | 100.472 | grass |
| 33 | SRN61007 | NE | Surin | 169 | 14.612200 | 103.353000 | rice |
| 34 | MSN61005 | N | Mae Hong Son | 195 | 19.3126 | 97.9589 | rice |
| 35 | PL61121 | S | Phatthalung | 14 | 7.566132 | 100.126538 | grass |
| 36 | NMA61004 | NE | Nakhon Ratchasima | 159 | 15.250800 | 102.420000 | rice |
| 37 | UDN61008 | NE | Udon Thani | 181 | 17.646200 | 102.442000 | rice |
| 38 | NKI61013 | NE | Nong Khai | 174 | 17.743300 | 102.685000 | rice |
| 39 | PL61006 | S | Phatthalung | 14 | 7.566132 | 100.126538 | rice |
| 40 | SKN61009 | NE | Sakon Nakhon | 156 | 17.180800 | 104.119000 | rice |
| 41 | RBR61003 | C | Ratchaburi | 7 | 13.488812 | 99.790862 | rice |
| 42 | CMI61001 | N | Chiang Mai | 362 | 18.745278 | 99.120278 | rice |
| 43 | BRM61010 | NE | Buriram | 195 | 14.715100 | 102.559000 | rice |
| 44 | PCT61003 | N | Phichit | 44 | 16.1463 | 100.545 | rice |
| 45 | UBN61017 | NE | Ubon Ratchathani | 135 | 15.148600 | 105.243000 | rice |
| 46 | PL61019 | S | Phatthalung | 14 | 7.566132 | 100.126538 | rice |
| 47 | NPM61001 | NE | Nakhon Ratchasima | 138 | 16.882100 | 104.682000 | rice |
| 48 | GUY11 | - | French | - | - | - | - |
| 49 | KJ201 | - | Korea | - | - | - | - |

C, Central; N, North; S, South; NE, Northeast
