# Supplementary material for: Development of novel InDel markers by whole-genome sequence comparison and genetic diversity assessment of Thailand rice blast fungus populations
Source: Stress Biol. 2025 Apr 27;5(1):27. doi: 10.1007/s44154-025-00212-1 (PMC12034604; doi:10.1007/s44154-025-00212-1)
Supplement: Supplementary file 2 — Additional file 2: Supplementary material 1. The genome sequence of P. oryzae for InDel marker development. Yellow highlight; Forward and reverse primer sequence, blue highlight; InDel position based on genome sequence of P. oryzae strain 70-15 (reference genome). [file 44154_2025_212_MOESM2_ESM.docx]

Article: Development of novel InDel markers by whole-genome sequence comparison and genetic diversity assessment of Thailand rice blast fungus populations

**Journal:** Stress biology

Napassorn Thamkirati ^1^, Worrawit Suktrakul ^1^, Athipat Ngernmuen ^2^, Theerayut Toojinda ^3^, Sureeporn Kate-ngam ^4^, Nonglak Parinthawong ^5^, Waree Laophermsuk ^5^, Pradipha Pradapphai ^5^, Watchareeporn Suksiri ^5^, Suphattra Janthasri ^5^ and Chatchawan Jantasuriyarat ^1,6*^

^1^ Department of Genetics, Faculty of Science, Kasetsart University, Bangkok, 10900, Thailand.

[napassorn.thamk@ku.th](mailto:napassorn.thamk@ku.th); [worrawit.s@ku.th](mailto:worrawit.s@ku.th); [fscicwj@ku.ac.th](mailto:fscicwj@ku.ac.th)

^2^ Department of Zoology, Faculty of Science, Kasetsart University, Bangkok, 10900, Thailand.

[athipat.ng@ku.th](mailto:athipat.ng@ku.th)

^3^ National Center for Genetic Engineering and Biotechnology (BIOTEC), 113 Thailand Science Park, PathumThani, 12120, Thailand.

[theerayuttoojinda638@gmail.com](mailto:theerayuttoojinda638@gmail.com)

^4^ Agronomy Department, Faculty of Agriculture, Ubon Ratchathani University, Ubon Ratchathani, 34190, Thailand.

[sureeporn.k@ubu.ac.th](mailto:sureeporn.k@ubu.ac.th)

^5^ Department of Plant Production Technology, School of Agricultural Technology, King Mongkut’s Institute of Technology Ladkrabang, Bangkok, 10520

[nonglak.pa@kmitl.ac.th](mailto:nonglak.pa@kmitl.ac.th); [waree_pla@hotmail.com](mailto:waree_pla@hotmail.com); [jane3957@hotmail.com](mailto:jane3957@hotmail.com); [june_26709@hotmail.com](mailto:june_26709@hotmail.com); [nutty_suphattra@hotmail.com](mailto:nutty_suphattra@hotmail.com)

^6^ Center for Advanced Studies in Tropical Natural Resources, National Research University-Kasetsart (CASTNAR, NRU-KU), Kasetsart University, Bangkok, 10900, Thailand.

[fscicwj@ku.ac.th](mailto:fscicwj@ku.ac.th)

*Correspondence: fscicwj@ku.ac.th

**Supplementary material 1.** The genome sequence of *P. oryzae* for InDel marker development.
**Yellow highlight**; Forward and reverse primer sequence, **blue highlight**; InDel position based on genome sequence of *P. oryzae* strain 70-15 (reference genome).

**> MD101_chr01:880997-884997**

ACCGTAAGTACCTGACTTTGTTTTGTCACCAAGCCAAGCCACTAACTCCTTCAAAGCTCCTGGTTTTTCGTTCAACCTGCCGAACTCGGCAATTCCGGGCGATCTTGCGGCGTCCCTGACGCATTCCTTGCTCAACCAGCCTCACCCGCTGCCCGTTCAGCCACACATGGTTCCTCTGCCAGCTTCCGAGATCAAGTCACGAAAGGAAACGCGCAAATTCAGCAATTTTGCTGCGCAGTGCTCACCAGCTATCCATTCGCCTTGCGCCGAGATTTTGGATAAGCGAAACTTCTCATCTTCTGCATTCACTGTTGCAGAAGTCGCTGCTGCTGTTGGCTCCAATATTAGCACACCTTCTCACCAAAGCTTTCAGATGCTGCCGCCATATGCAGTGCCAAGAGGCCCTCCAGAGGCAACCAACGACAGGGCTTTCTATTCATCTTCGGCTGGCTCTCGGGCCTGTTCATCGGGCGATCTTCTGACTTCTTTCTCTAATCTCTCGGAGGGAATCAATGCGCCTATGTTATCGGCAACGACGTCTGGTACAAACAACAACGTTACGCCTTTGGTAGAAGCTGCTCCTGACAGATGCAACAAGCGTCTACGCAACTTTACCCCTGCCAGCATCAAGGCTATCGATGAGGAAGACGAGCCTAGGCGTGTCAGCCCTAGATTGAGGGCTACTCCATTCGCGGGGCAGGACCGCTGCGAGAGCATCTGAACTTGGTGTATGGCATTCATGCCGAGCCTGGCAATAATTTGTTACAAGGTTTCTCTCGCATTGAATAGCGGTCTACTCTTGTCTTAGTGCTGGCCTGCTTTGGGCTCGAATCACCAATGAGTGTATGTTTTGAGTTAGGCCATTGATCAGATCCAATGATTTAATATTTTTAGTTCTCAGGCCATAATTATACTTGAAAGGGACAGGCTCAAGTCATTCTTCATCTCATTATTAGTGGGGTCTAATTCTCCACTAGGATTGTTAGTATGATGGTGTCTAATAGTTTGGAGGTCAGTCTTTGTGATACTCAAATTTATTCTTGGCACGATACAAGTTCTCGTTCTTTCGCGGCCCCTGCCTACTTTTGCTCTTTGCTTATAGCTATATGCAATCGTCAGGAATGATTAGGCGTCGTCATTCTTGGTCTTTAGCGTCATGATTTTCTCGGCCTTGTCTGCGGCTTCGGCTGCGGACGAGACGGGTTTCGCAGCGACACGGGCTTCCTAGACAGGAACGTTGGCCAGAGGTCCGGGGCAAATAAACGCGTGCCGAGTTTCTGGAGCGACACGACGGCATCTTAACTCACTCTCAGGGTCTCGTGCTCCTACGTGATATGAGGGAAATCATTGGCAAGGACTCCACACTTACACTCATTAGCGACAAGTTGGCACTCGATAATAGCTTTGGGGGAGGGCAGGGGAATGCTATTTCCCAGCCACTGCCTGCGAAGGAAAGGTGCTGCGCGGTCGTGGAATGCCCACCCACCTCGCTTACTTGGGGAGCGTCAATTTGAAGTCCAGCACTGAGACGTAGGGGAGCACCAATGTGCGATAAAAGAGTTAGCTGGCCATAAGCTGGTAGATTGCATTTATTTTCCGGAGCAGAGATGGTCAAACAGAATTCGATTGTGATGCTCCCTGAGGAACTGCAGATCAAATTTCGACGGGACAATCCACCAAACCCAATATGAACACCCCCATTCGTATTTCCCAATCACAAACGTACCAAGGGGATGTCTTCGGATAAATCAACAATGCTAGCCTACTACTTGGTCGATTCATCCATTTTTCGAGCTCCAGCCATCCGAACACCCAGTCGAAAAATACGGCGGCTGTCTTGCGGTAACCCAGAATGTGGTTTCCCAAAGTCAAAAT

TTCTTGGCGGGTTGAGCCAAGTTTCACGTCGGTTAAGCTTAGCTTGGCTGGGATCGAGCTTTTTGGGCAGGCTCCCTGGCGTATCCCCTGGGTGTGACACGGCGGCGCACCTTGGCAGGTTCGCCGGGGGGGGGGGGGGGGGGGGGGGGGGGGGCTGGTACGGAAGACTGGGATTTGGAGCGCTGCAGTTCAGGCAGTAAGGTGAACTAAATTGCATCGATGCTAAAAAAAAAAAAGAGAAAAAAAGAAAGAAAATCTCCAGCCACAGCACATTCAGCCCTCCATCCTCAGAACGCTCAC

ATCACCGCATCTGAACAAGACCTGCGTTGTGACGTCGTTAAAATCAGGGTACGCCCAAGGGTGGCGGCGGTCATG

CTGGCGATAATTCAGGGCAGCTTGTCGGGTGGGGAAAACAAAAGGTCCAAATGCCGCCCCGATTTCAATCTTGTACAAGCGTCATTTCTCCGCACGAGCTTGAGGTTGTCTGCCGCAAAGATACCCCTAAAAAAAAGACTACATGAGTTCCCAAGGTAAATGGGTAGGCCTTCTAGGTACCTCAGCACAAAGAGGTAAAAAGGGCGAGTGGGGACAAGGCCATCACGTGTGACGATTTGACAGCTTCGGGTGAGGTAGATCGAATGATAAGGTCGGATCGCCATAGGACCAACGGGAGGTACAGGAAGCTAAGGTACGGAGGAACTTTGGGGCAAGGCAGATAGACGTCTTATCTCTGTATAAGTAACGCTGTCTCTGCTTTATTTAAACTACTCCAAGAGCGGTCCGCCCTTTGCTGTCTGGTAATCCAATCCATTCATCATCAAAAATCAATATATTTACAATGGCGCAAAGCATTAGCGGTAACAACATCTTGAACCCCTTTGCCAAGGGTCCTGGGCCCACCAAGCCCGAGCACATGCAATATGGAGACCACTCGAGCAGCTACTGGCCAGAGGGCTGGAACTACCAGAGGTACAGCGACGCGACGGCAGAGGACATATTGTCGCTGCCCGACGGCGAGCTGGACAAGATGCAGGCGGGGCTGAGAGAGGTTCTGGGCGAGCACGGCGCGGCGGCCATGTCCAAGCACAACTTCGAGAGGAGGCTCTCGGGCGGCGCGACGATGCTGGATGCCTACACAAGCAACGCGCGGGACCGCAGGGCGAGCGTGAGCAGCAACAGCGCCGCCGACGCCGTGACGCCGAGCTGGCTCAAGCAGTGGAGGAAGCGACACGAGGGCCAGCCGTGGGGGTTCGTCGGCGTGCGGACGGGATGCTACGGCGGGGGTGGCGCCGACCAGAGGTGGCAGCAGTTCCGGGCGCAGTTTCAACGCATCCTCGAGGTGCCCTTCGATTGGGCCGTCGAGAATGGCAGAAGCGGCGGCGGCGGAAGCACCAGTGGCGGCAGTGGGGAAGCCGCGTCCAAAGCGCGGGAGATGTTTGAGGTGCGCTGGATTGAAGACCCCGCGCTCGATGGTGCGGATGTGCAGATCCTCAGGGGGTGAGTTTTTTTTTTTTTTTTTTTTTTTCTCCTATTCTTTCGAATGTTTCGGGTTGCTCCCCGTCCGTTCAAGTGTGGCTTGAACTTTTTTTTGTAGAGGGTAACAGAGTGTGTCCTTTTTCCGCCTGCACCCTTTCCGCCTGTTACTCCTTTCGGCATTTGGCGGGCAGAATCAACCAGAGCCTCAGGCAGTCATCAAAATGACGTAACTGACAGCTTGTGGGGGACATACGGAGCTAACCCCAATTCGATCAAACCCAAAAGTAAATTCCAAGAGCTTCAATCCACGCTTTCGCCAGGCTTCAGCCAACCGATCTTCCTCTCGGCAACGGATCAGGTCGTCGATTCAGTGCTCGCATTGGCACCCGAGGACCTCCCAACGACGGCCTCGCGGCGCGGCAGAAATCCCGCGCCCTGGATACACGTAGTCGCCGCCTCAGCGGACCGCGGCGTGCCGGACGAGCATGTCGAGGCGGCCTGGTTTCGCGACGTGTTCAAGGTCGCCATCGAGGTCCTTGCGGATGAATTGTGGTGGATCGTCGACTCGGGCACCATGCCGCTTCGACGCATCACCCGCGGCGTCAAGGGCGAGGGTGTGGCGGCGGACGCAGGGAACGACGATCTAGAG

**> MD102_chr01:1530014-1534014**

ATATAAACAATTATAATTATTTTGGGGTAATTACGGCTGTGCTGATTATTTTTTTGGCAATGATCGTGGTTATTCCTATTTTGGTAATCGGGTTAGGGTTTCTTATCTTATCACCACGTGACCTCGTGCTCCCTTTTTCTGTCTTTGGTTCGTGTTTCTTGGTTAGTTCTGGCTTGGGTTCCCGACACCTCTCTCATTCTTGCCCAATCGTGTCGGTACCAATCCTGGTCGTAACAGGAGGGTGGCGAACTGCATTTTGATTGAGTTGTTTTGTGAAAGATGCACGTGGCACTTTTGGGCGAGTGAAGAAGTTGTAGAGGAGAGAAAAGATTTGTAGGAGAGAAAAGATCTGTAAAGGAGAGACACGATTGAATGAGTGTGAAGTGATTGAATGAGTGTGAAGTGATTGAATGAGTGTGAAGTGATTGAATGAGTGTGAAGTGATTGAATGAGTGTGAAGTGATTGCTGATGATGGATGGGAATAATAATGTTCGATTATAGTTGGGAAGCACCTTCTTTTATACACAGTTCCTATGTTACACGGCTTCTCAACACCAGATGACGCAGTAAGTACCTGGTAGTACCCTTCCGTGTATGACACGGTCGATTATGGAACTCTTTGCATTCGCGATCGCCTCGAGGCGCACCTCGTAGCCGCCCGTGAGAGACCGACAAGGCCACCGCCGGATCTACCACATGGGTCTGCAGATCTTGGTCACGAATCATATGCGCATTATTGTTTGGCAAGATCGTAAATTATAACAATGCGTGCCTAGCTACGGGAAGGAGATTTATATGGAGAACGTATATGGATAGCTAAAAGAAATGGGCAGGCACACGAAATAGCCTATTCCGTGATCTCAATGGTGTACATAGTAAATCGCCACTCGTCCAGGGGGAGCCCTCCACAGTGCATTGCGCTGTTGGCAGTGTTGGCTAATGTCTATCAGGAAGGCCGGACATGAATTGGAATCCTCCAGCATTTGCAAGAAAGTGTGGTGCGATCAAGGTACACCCGGGATCACCGACTATTCCAATTGAGCTTTGGTCATTTGTTGTGACTGGCACCTACCCAGGTAGTACTAGACTAGAACTAGAACTGGTACCTAGGTAGGGGGATAAAAGGTAATGCATATAGGCGCCATCCCTGCAAGATCTAAATAAGCATGACTACGATGCTACATGCCTTCGCCCACCTTAAGTCAGCTATGATTGTCGGAACATACCCACAAGACGATCATAGTCTATGCATACAGTCAGTCAAGCTTGAATACATCAATGCCATATTTTGCGCCTTTTTCTTTCCCGTACTCCACTTGCCGTTCTATTACATAACGGTCTCTCCGTTAGTGCTGAATCCGAAAAAAGAATATACCCACCGCTGACTACAAAGGGAAACATTTACAAGTGGACTGGAATGCTGTGGATGTGGAGGCTGCAAGAGAAGATGTGCCCACCAAGGGTGACTGGCTCGGTGGCATATGGACGCAGTTTGAGCAAATGAAGATCAAGCATGATAAAAAGCATGAGAAAAAGCATGAAGGGCTTGGGTTAAATTGGAAGCGGGCCCGCCGGCCAGCGGGTCAAGTAAATTTCTCCCATCTTTAGCTTCATGTGTTGTTTACGGAATCTTTATTGACGAGTGCTAGCAAATGACTAATAGGCATCAACGATTGATTGTTTACCTACCAAGCTACCGACCTACCTATTCATGCACCAAAGGC

ATCCACTATGTGGTACTATTGTGTTTCGATTACGGTATGCAGTGCAGAAACTGCTGCGTGGATTCAAGTGGCACATGACGGTGTGAACGATCCAATTCTGGCTAAACGCCATATTGCATGGTCTGTACTAGATGTGGCGGCCGAGTTTCAACTTTCTGTGCAACCAGTGCTTCAACAATGACATACGCTCCGCTCATATTGTGGTGGTGCATCTTGCACGCAGCTTGCCAATTTCACATGGCGCTATAAAAAGCTATTATTGCCCAGATGCGGATCAGATGCAGATGCAGATGCAGATGC

AGATGCAGATGCAGTCGAGGTGTATCATATCATCCCCGTGGTGCCACGCACTATCGAAAGAGTCACCTTTCACCATGTATGATGTGACTCAATCTGCAGTCATATAACCTCATTAACGCACGCTAGTGGTACTTCAAGCTTTGAAAAGTATATGGTGGCGTTGGATTTGATTCTCGTGGTGAGCAACCAAGACCACAAAGGTTCTCGAGAGTGGCGAATGAGGAAAAAGCATATGTATCATGGTTTCCCTCCCAGCCACGCTTTTTACTACTGCTGCAAGAAACTTGTAGATTTCCGAGCCCATCATGCATTGGTCAATCTAGCCAGCCAAAAAATGTGACACGAAGAAGAGAATTTCCAGATATAAAAAGGGTTCTGATGCCCAGTCTTGATCCCAATCCCAAGAAAGCCAAGTCGTAATATCTCCAAAACATTTCAAACATCAAATTTTCGTTCGAGTCATCAAAGATGCGCTACACCACCTCGATCCCTCTGGCCCTGGCCGTCGGCGCCGCCAACGCGATGAACACCAAAGTGCCAACTGGTGCTCACTCGTCCGGCTTCCTCACCGTGACGACGGCCACCCTCGCCACCACCACCAGCACAACCCCTTACCAGATGCCCACCATCATCCCCTTCCCGGATGAAGAGCAGCCCTACCCGGACGACCCGTGGGGCACCACCTGGACCTGGATTGCAACCGCCAAGGTCCCCACCCCCACCGTCGAGCTCACCAAGACGTCGGGCGGCGTCCTGGAGGTGCCCCAGCCCACCGCGGTTACTCCCGTGCCGCTCAGCAACGAGAACGGCCCCATCATCGGCCCCTGGAAGATCCCCGCGGGCGGCCCGGCCATCAAGCTCGACGGCCTCCAAAACGACACGGACGCCAGGACCCCATTCAGCGACGCCCCCGGCCCCGTCATCCCGGACGAGGACGACTTTGAGATGCCAGAAGTCCCAGATGCCGACGACATTGTCGAGGATCTCATCCGCGAGGGCATGAGCCGGCAGGAGGCGGAGGCGATCAGAGCCCTCATGGGAGAGGGTTCCAAGGAGTCAAAGGCCGTCCAGGAGGACACCCGCAAGGTCGGCGATGCCATGAAGGCTCTAGCCAGGGATTTCCGCCGCTTGCGTCAAATGGCCAAGGCTCACTGAATGCAGGATAAAGGCGGGATTACGGAGCAATGTGCCAAAGAAGCAAATCTCGCATAAGGGGCCTGCACATGGAATGAGAGCCGAGGTTGTGTACAGAGCTGACATAGCCTGCTACTACAAAAGTTTTGAGTTTATAAGCTGTCCCAACTAAGAGGATAGTTATAACGGAGTATGATTGCCCAAGAAAAACAAAAAGAAACATTTTGAACTGTGCGTAGATGTGAGGAAGCAAGGTTGGAGCTAGTCCTTGTTCGGGGCTAGCCTCACCTGGATCGTGCGTAGAGAAGTTATCAAGTGAAGTGAGGCTTGATGGGAATGGTGGGTGTGTGCACTCGCCGCTCGCATGGCTCGGGGAACAAAAATGTTCTCTTCCGGTCAGGCTTGAACTGACAACCTCTCGATGTCGGGTCGGAAAACTAACAGTCGAACGCGCTAGCCAATTGCGCCACGGAAGATTGGCTTCAATCGATTTGAATCGCTTGATATCAAGGGTATGGTGCGCTAGAAGGCGAGATGATTCGTTTATATCACGTACTAGACCACGTTCAGCGTCAAGGTCACTGCCTGTATTGCAGGCTCAACTGAGGGCTTCAGCTGCCCGCCTGAGCCGGGATTATCATCTGCAAAATACTTTTTGTGGGGGAAGATGGCGATGGGATCCACCATCCAGGTAAACTCGGAAATGGGGAGATGTGGTGATATTTTTGGCTATACCCACCAGCTTGGACGCGGAGAATTTGATTATCAGAAAGGCCAGAAACTTTTTCTCGCGACTGCAGAGGCATAGATAAAGTTAAAACACGGGGCTATCCTCACGGGC

**> MD105_chr01:2359505-2363505**

CTTGTACACAATTTGATCAAATCTCAGGCCTTTGAGCCACTGCCCAAACGCTTCTCAGCAGCTGCCCGTATTCCCCCCCCCCCCCCCCCCCCAAAAAAAAAAAAAAAAAAAAAAGGAAAAAAAAAGAAGAAAAAATAAAATTCATACACATAATAGTATAGTCAGTCAGGCAATCAAATGGAGTCTCATACGATCACCGTGTTGTTGTTGGGAGATGACAGGACTGGAAAATCCACCTTCCTATCGTAAGCATCATCAATATCTACAACAGCCCCCATTGAATTGTTTCCAAGTTGGTTGAGAGAAAACGCACCATCATTTCCTAACAAGAAAATAAAAATACATCAACTGCCGTAGTCGCGTAGCCCAGGGCAAAGACAGGCTGACCGGACGAGCCGAGATCACGCTTTTGCGCGACTCGGACCAGCCCTTCTTCTTTGAACTGCGGTCCAGAAAAGGGGTCTATCGCATCGAGTTCCACGACACTTCTGGTCCCACCAATTGGCGCCTTCTTCAGCCAGATCTCATCGTGATATGTTATGATATAAGCCAACGCCTCAGCCTCATAAATATGCAGAGAGTTGTGAGTCGAGTTGCCCAACCGCCCCCAATAAAACCATCCGGTACCCATTCATAAGATAGAGGCTCACACAGTTGCCCGCGATTCTCGCACGCCCAACATAAAAACAACAGTGGATTAAGGAGTTGCGCCAGACATTTCCAGCCGGCGACTCGATCCCCATCGCAGTCCTAGGTCTCAAGCGAGACCTGAGGTCCGAGGAAGACCCCAACGGCATCATCTACCCGCAGGAAGGATATCGCATATCGCAGGAGATGAGGGCGGATCGATACATGGAGTGCTCGGCCGTGACGGGCGAGCTACTGAGTGAGACTTTCGAGGATCTATACCTCATGGCCGTCAAGACCACGACCGCCGAGGGAGGGCAGAGCGAAGGGGCGTGTGCGATCATGTAGAGCGCGTGTGGGTGCTGGTGGTGACTAGGTAGTTCTTGTCGTAAATCATTTCGCAATGTTCATTCTGACACGAAATAATAAATTTACCCCCTCAATCTCACAATCGCGCCTTAGCGTCACGTCATGGCGCATTGCCGTTCTCAATCGAATCGGTCACCTCTTCGTCGCCATACTCCTCCGCCTGCTCCTCTGAGACCGGAAAGGCACCCAGGACGTCGACAACGGCCTCGAGGATCTGCGTCTGCTGCTCCTCGGTGAAGCGCTCGGCAATCTCCTCGATGCAGACGTGCAGACCGGGAAGCTCGGTCGGGCGCAGGTTGGCGAGCATCAGAACCTCACCCTTGGCGAGGCCGAACTCGGCCAGCCTGCCGAGGAGCTGCGCGAGGCTGTCCTTGGTGTAGGTGAGCGGTTTTTGGCTCAGAGGGTTGGGGTAAGTCCCGCAGTAGTTGACGACCTTTTTGGGATGAGAGAGTCAGCGGCTTTTTTTCTTCTCTTTCTTCTCTTTTCTGTCACCCCTCTCTTCCTTGGTTGCAGATCGCGTTCGTTTGGTTGTCTTCTGGATGAATTTGCGCCCATTTTAACATACATCTTTGGTCACTGCCTGATAATTCTTGGGCGGTCGTCTATGCTCCTTTTTGTGCCGTGCAGAGAAACGGGTCTCGGACTCAATAAGATGAGTGTAAACCTCGTAATTTGTCAGAACTGCATTCTGTGCTTCAAGAATCTTCATTTCGACAGATACTTGTTTGCCGGTTTGATTGAATGGTGCTCTGATATTTGCAAGGGGACAAATGAACAAGAATGAAAGGGGTGCAATCGCTAGTT

CTGGTAGGTACCGTAGACAGTGTCTCGGTATGTTACTGTTTAATCTTGGCCTCGAGCAATGTTTCAAAACTTGGCTTGGTAGTGAGATCGAGTTTAGCTCCGCTGTCCGCTTTGAGCTGCTCGCAGGATGAGCACCGCACGGCAAACGAAGGTGGTGGGGGTGGTGTGCAACCTGCGATGCCCCAAATTGCTACTTGGAGTTTTTTTTTTTTTTTTTTTTTTTTTTTTTATCACCTTAATCCAAGCAGGCAGGTACTGTACTGTACATACTGTAGGCAAATCACATGGAGCTCAACCCGA

TTGATTCAGTCTTTTCTAGGTGCAGGATCGTGTGCGTAAACCCCCACTCTGTTTCCCCACCATCCTTACTCCAACAGCATTCTGATTCAAAGGTGCAATCTTCTGTTTTTCCGTCTGTACTGTCGCTTGCCCGAGCAAATCACAAGATGAAATTCAATAAAAGGCCAGTCCTGGACCCTCTGTTCAAGAGCTCCAGTGTAGAGGAGCATCAGGACTTGCCTAACCTTGTTTTTTTTTTTTTTTTTTTTTTTTTTTCACAACGCTGCGTCGTCTGCCGCTTCAGCAATACGCACTTGACTTGACTTGACTCTTTTTTCCCCGCAAAAAAAACCTACCTAGCTTGCGGAGCCCGAAACTCGAGGCCTCCAGGCTCACAACTTCTTCGCCTTGACCAAAACTTTCTTACACCCATTTTTTCCGCATCCCAGTTAGCTTTTAGCCAGGCACTTGGTGAGCGGAAAACATCTTTGATCTCTTTTGAAAAAAAACCAAAAAAAAAAAACAAATATCGAAATTCATCATCCACCAGGTGACCGTGCAGTCTGTCTTCGTTTGCCTGTTGAGGAGCCGATTTCGGCATTGCTCCGGGATCAGGTAAAGAGAGCCGGCACCACCCCCAATCGTGAAAATTCGGGTGTGTGTACCTTGATCGCCAACAAGGGAGAAAAGCAAACAGAAAAAAAAGTGAGCAAAGCTTTTGTTCGGTATGCACCCCTGCATCTAGAACCACTCGCTTGTGGTTTACTACTAGGTCTACCAAAAAAAAAAACTGCCTATCACCATGAATTTCAAAGCAAAGGACTGACGAGAGACATTCTCCTTTTCTTTTTCTTTTTTCAATACAGTCTCGGCCTTCAATTGCGAAGTGTATCTAAAACTATCGGCCCCGGCCACCGAGGAGAAAAAGAAAAAGAAAGAAAACGCCACAATCACACACCCAGTCGCGTCGAGTATTTTTTTTTTTTTTTTTTTTTTTTTGGCACGACATCTGCCCCGCATCACAACATTGATTGACCAAGACCTCATTGCTTTCTCTCAAACTCACTCAACGCCCCAGACACTCACCTAACCCGGCAACCAGCCCCGCCGGCCATACGCTGCTGTCATGGTCCGCAATATTGTCGTCCTCGGTGGCAACTCCCACCCGGAGTTGGTTGACGAGGTCTGCGGCATCCTCTCGATTCCACCATGCGACCGCATCCTGTCCAAGTTCGAGGTGGGAGAGAGCCGCTGCGAGATCAAGGACTCGGTTCGTGGCAAGGACGTGTACATTATCCAGTCTGGAGGCGGGAAGTCCAACGATCACTTCATTGAACTTTGTATCAGTGGGTGCTCCCTGACTTGACCTTGTCCATCCGCACTACGGCTGCTCAGTTTGTCTGATCTGACTTCCATGAACAGTGATCTCGGCCTGCAAGACAGGGCAAGTATAATTCCTCCCTTTTACGGTCAATTGACGGCCTGTCGTCGCCATTGCCTGATATTCGGGGCTCTCGTGGCATCATATTCTCGCATTTGTTGAGGAATCCTCAACTAATATTTGGTTCTCACCCTACAGCTCCGCTAAGCGTGTTACTGCCGTACTCCCTCTGTTCCCCTACTCGCGGCAACCAGATCTGCCATACAAGAAGTCCGGCGCCCCGCTGTCCCAGGCCGCCGGCAGCTCCAACAAGGAATACACATTCGAGAGCGTTCCAGCAACACCGGCTCCCGGCATCCCCAGGACTGCTGGGCTTACCAATGGCGTCGATATCACGCATATGCTGCGCGACAGCTCCTTCACAAATGGCAACGCCCCAGCCTCTCCCCGGCCAGCGCCTCCCTCGGAACAATTCTCTGGCAAGAATGATGCAAATGGTGTGAACGGCAGCCGGTCGGGCAAGCCAAAAGCCCACAATTC

**> MD106_chr01:2493243-2497243**

AGGTCGCCCCCGTTTCCGCTTAGGTTGGGATTCAGCTATAGCTGGCGATTGAACAACTGCTGCCGCAGAAGAGGTTCTGGGCGGGCGGCCTCTTTTCTTGCTTAGAGCTGGGACTTCGGCATTTACCTCTCCAGCCGAGCGCGATGGCCTGCCTCGTCTCTTTCGTGGTTCTGTGCGACCGCCACCAGTGTGCACCCCGCTCTCGTCAGCCGCCTCACTGTCATTTTGATTTAGACACCGGCGCTTGCTGCTTCTCTGCGGCGGCAGGGTTTCGCTTCGAGATGCTGGATTCTCACTTTCAGGTCGGGAGACTTCAGCTGTGGGTCGCTTTCGAGGTGTTGGCGCGTCTGCTTCAAATTGCGACGCGCCTTGGGCTTCGCGCCGGCGCTTTTGCCGAGACTCCGGAGCCTATGGATATTGAGTTAGCATAATAAAAAGTATAATTTAATAAAATAATAACTGACCATTTTGAGCAGTGCGCAGGTGATAATGCTAACAAAAAGGGCAGAAGTCAACCATGTTGTCGCAAGCTGCCCTCCGCCATTGAGCAATTAATTAGGCAGGTAGGTAGCTACCTAAACTCGTTGATGTCACCAACGCGTCGGGCATTTAATTACTTCATGTACCCAGCAGGGGACTTTTCCACCCCAGTTCTGTGGATACCTCTGTAGAAGCTATGACCATACTACAACAACGGTCAGGCTTCTGATTCGATAAACCCTTGATGTGTGTTCCCACCGCGCTTAACCCAGTTTGCTCTTTACCCGGCGGCATGGACGGACTGCACGCAACATTAACAAGGATTCGTAAGAGTGTCCTATTTCCCAAGACCAGAAGTCTAGACATCATTAAAATTCCGGAAGGGTCTTTTTACAACTTCCCGAAGAAATTGCTCCTGCGATCACGACAGGTGGCTTTGAGACCATTTTCAAGATTGCCCTTGTCACTCCTTCTTTCTTCTTGTACTCTTTTTGCATCAAACCCAGCTCCTTGGCGATATAAACTAAGGTAGAGCTTCCTATCCGTTAGTCTCTTGGCGGCCGACCCCTCGCAGTCCCTAACATCATTGCCGACGCGCCACTGGGCAAACGGCTCGGTTATCCCAAGTCACCTACACCTCATTTCTAACCATTAGATCACGAACAAATCAAAGATACCTGCGATGCGCTTGTCCACTCCCCTTCTGCTGGCCTTGCCAGTACTCGGTGTCTCCGCCGAGGGCGCCTACGAGCAATACTATGCCAAATTCCAAAACTTTCTTGGCCAATTTGGCGCAACGCCCCCTAGCGCAACGCCCGAGGCCGACAAGGCAAAGAGCTCCGATGCCAGCACCGCAACCGGTAGTCAAGCGCCGGCCAAGCCCATCGAGGTGCTTACCTTGGATAACTGGAAGGATCTGCTCTATGGGCCTGTGAAGTCGACTTCCACGGAGCCTGAGGAGTGGTGGGTTATGATCACCGGTGGCAACAAGACATGCTGGGGTACGTATGGATGGAAGACCTGATCGTGTCGATGAGATGTGTAACGAACAACCAGAAGCGCCGTTTGCTAACTTCATTTCCGTCTAGGCCGCTGTGCCATGCCTGAAGCTGCTTGGAACGCATCGACACCCAAGATCGCTGCCCTTCCAAACTCCCCGCACTTAGGCTACCTCAACTGCGAGGCCAACCCAGTTCTTTGCAACGTTTGGTCAGCCGGCGCACCTTCCATCTGGTCCCTGAAGATGAAGCCAGCTCCCAGTGAGGTTGAGATTGTCATCAAGCCATTGTCGCGCAACGAGACTACGGCTCAAACCATTAT

GGACATCTACGAGGAGGGCTTCGTCAAGGGCAACAAGGACAAGTGGGTGTTGAACGACAGCGTCTTCCATCCGTTCAACGGCTTCTTTGCCAAGAACAGTCTTAGCGTTCCCGTTGCCTACGTTCTCTGGTTCTTCAGCGTCGTTCCCAACTGGGCCGTGATGCTTTGCGTGTCTTTCATGAGCCGCAGCATGATGTAAGCACACACACACACACACACACACACACACACACACACACACACTCTCTCTCTCTCTCTCTCTCTCTCTCTCTCTCTCTCTCTCTGATATTAACGTAACCG

AATCACAGGAACCGCCGCATGGGAGTCGACCAGGCTGGTGCCGGGCAGGCTGCTCGTCGCGGCGCACCCCCTGGCGATGCTAGGTAACGCACTGCCAAAGTTTGAGGGGCTGGTACAGTTCAAGGATTGGGTTGCATACCTCACTTCTGTTATCGGATCACCTGCCGGTTACTTTTTTGGATACAAGCATGGGCAGTGGGGGGGGGGGGGGGGGATATAGTATGAATCTCCTTTGTCAATGATGTGTCTGGGTTGCAACTCTATTTGATGGCGCACTGCTCTATTTTACTATGGATTTATAATATCCCGCAGGTATATCTTGGTTGCACAAGAGTTTATTTTGTATTTAAGCCTTTGCTTGTGTCGCAATCTCACGGATTTTTATTCTCGCATATATCCTGTAGGTTTTTCAAACCATCCAAGACCTGCTGCGGCAAGCTCGCATTGGATAAACGACCGTAACGCAGGTCGCTCAAGTCTCCAACAGTTTTCTTCACCAATTCGAGAAGTCTGGTCTCTTCAGTATGCAGATCCTTAATCTGGCTCTCGACTGCGGTTAAAGAGGTTTCCATACTTTGCACCACAATACTCAGGTTTGTGAGAGGATCGTTGCCAACTGGAGTAGAGCTCGAGGCAACCAGCTGGTGACGCTATCAGTATTCATCTGGTTGGTTTCGGTGGAATTGAATATCGAGAGTAACAAAGTTGGGGATTCTCACCGCTTTCTCAACTTCATCTTCTGCGTCGTACTCGTCCGCCAAAGTCCCTATCCTATCCGCCCTAGTGACCTGTCTTCGAATAACGTTTGCGTGCCGGGATGTCTCCTCTGCGATGTTTTCGGAGACTACTTCTACGACATTACTCCTTTGGCGTTGAAGATCCCTATACAGGAATCGTACTTGAGCCGATGATCGGTGCTGTAGAGGAAATAAGGCTTTGAATTCATCCATGGAGATGATTGTAGGGAGCTGTGCTGGGGCTAACAAGAAGTTGCTCAGGATGGTCGATTCTGTTGGCGCCATGGTCAGAGCCGGCCTGATTGTACGAGATAGAACATCTCTGTTTCTCAATACAGTGACATTCGAGATGTTAATTAAAAGGCGTGCTCAAAAATTATTTCACTGCTCCCACCCATCTTGGCCTGAAATTGACATAAAGGCAGCGGAGTGGGCGTTTGTCATTTAATGATCGACGCGTCGAAGTAGCTTCTTGACACGTCTAGAACGCGTCGCCACTCGCATAGCGGTGGGCCTGTCAATGGTCGTGCAAAGCAAACTCCCGTGATCACGGGATCACCCTCAACCATCCTTTTCCGCGTTCACTCCCTCTGTCCCTTTCCATCTTGCCCAAATCGGCCTGCATCGGTCTAATTCTATCATCACCAAATTGCCTTGCCAGCAAATTGCCTTATTCACCAGAGATTTGTGCAAGTCTCTTTGTTTGTATTGGCGCATCCGAACCACTAGTAATTGATTGGCATCAGACGTCGGGTACTCTTTACACAAAGCCTGATCCGACGTGTTTGGTTTGCAACAAGGGAGGCACTAGCGCGTTCTGGAAGCTCAAAACACCATCAGCAGAGCTGTCATAAAAAAAAAAAAAGAAAAAGAAAAAAAACCAAGACAAACCCAAAGGACAAGGCGGGTTAATTGTCCAGGTCTCTGAATCGACAATAAATTCATCCTTCGCCGCATCAAAGACTTGCAAAGATGCCGCCAAAGCAATCAGCACCGCCCTTCAAGGCGGACGAGAGGGTATTTTGCTTCCACATGGACATGCTTTACGAGGCACGCATCCTCGAGATCCAGAGCGAGGACAACGACCTGTTCTACAAGATCCATTACAAAGGATGGAAGAACACGTGGGACGACTGGGTGCGGCAGGACAGGCTGCGCAAGTT

**> MD107_chr01:2493261-2497261**

CTTAGGTTGGGATTCAGCTATAGCTGGCGATTGAACAACTGCTGCCGCAGAAGAGGTTCTGGGCGGGCGGCCTCTTTTCTTGCTTAGAGCTGGGACTTCGGCATTTACCTCTCCAGCCGAGCGCGATGGCCTGCCTCGTCTCTTTCGTGGTTCTGTGCGACCGCCACCAGTGTGCACCCCGCTCTCGTCAGCCGCCTCACTGTCATTTTGATTTAGACACCGGCGCTTGCTGCTTCTCTGCGGCGGCAGGGTTTCGCTTCGAGATGCTGGATTCTCACTTTCAGGTCGGGAGACTTCAGCTGTGGGTCGCTTTCGAGGTGTTGGCGCGTCTGCTTCAAATTGCGACGCGCCTTGGGCTTCGCGCCGGCGCTTTTGCCGAGACTCCGGAGCCTATGGATATTGAGTTAGCATAATAAAAAGTATAATTTAATAAAATAATAACTGACCATTTTGAGCAGTGCGCAGGTGATAATGCTAACAAAAAGGGCAGAAGTCAACCATGTTGTCGCAAGCTGCCCTCCGCCATTGAGCAATTAATTAGGCAGGTAGGTAGCTACCTAAACTCGTTGATGTCACCAACGCGTCGGGCATTTAATTACTTCATGTACCCAGCAGGGGACTTTTCCACCCCAGTTCTGTGGATACCTCTGTAGAAGCTATGACCATACTACAACAACGGTCAGGCTTCTGATTCGATAAACCCTTGATGTGTGTTCCCACCGCGCTTAACCCAGTTTGCTCTTTACCCGGCGGCATGGACGGACTGCACGCAACATTAACAAGGATTCGTAAGAGTGTCCTATTTCCCAAGACCAGAAGTCTAGACATCATTAAAATTCCGGAAGGGTCTTTTTACAACTTCCCGAAGAAATTGCTCCTGCGATCACGACAGGTGGCTTTGAGACCATTTTCAAGATTGCCCTTGTCACTCCTTCTTTCTTCTTGTACTCTTTTTGCATCAAACCCAGCTCCTTGGCGATATAAACTAAGGTAGAGCTTCCTATCCGTTAGTCTCTTGGCGGCCGACCCCTCGCAGTCCCTAACATCATTGCCGACGCGCCACTGGGCAAACGGCTCGGTTATCCCAAGTCACCTACACCTCATTTCTAACCATTAGATCACGAACAAATCAAAGATACCTGCGATGCGCTTGTCCACTCCCCTTCTGCTGGCCTTGCCAGTACTCGGTGTCTCCGCCGAGGGCGCCTACGAGCAATACTATGCCAAATTCCAAAACTTTCTTGGCCAATTTGGCGCAACGCCCCCTAGCGCAACGCCCGAGGCCGACAAGGCAAAGAGCTCCGATGCCAGCACCGCAACCGGTAGTCAAGCGCCGGCCAAGCCCATCGAGGTGCTTACCTTGGATAACTGGAAGGATCTGCTCTATGGGCCTGTGAAGTCGACTTCCACGGAGCCTGAGGAGTGGTGGGTTATGATCACCGGTGGCAACAAGACATGCTGGGGTACGTATGGATGGAAGACCTGATCGTGTCGATGAGATGTGTAACGAACAACCAGAAGCGCCGTTTGCTAACTTCATTTCCGTCTAGGCCGCTGTGCCATGCCTGAAGCTGCTTGGAACGCATCGACACCCAAGATCGCTGCCCTTCCAAACTCCCCGCACTTAGGCTACCTCAACTGCGAGGCCAACCCAGTTCTTTGCAACGTTTGGTCAGCCGGCGCACCTTCCATCTGGTCCCTGAAGATGAAGCCAGCTCCCAGTGAGGTTGAGATTGTCATCAAGCCATTGTCGCGCAACGAGACTACGGCTCAAACCATTATGGACATCTACGAGGAGGGCTTCGTCAAGGGCAACAAGGACAAGTGGGTGTTGAACGACAGCGTCTTCCATCCGTTCAACGGCTTCTTTGCCAA

GAACAGTCTTAGCGTTCCCGTTGCCTACGTTCTCTGGTTCTTCAGCGTCGTTCCCAACTGGGCCGTGATGCTTTGCGTGTCTTTCATGAGCCGCAGCATGATGTAAGCACACACACACACACACACACACACACACACACACACACACAC

TCTCTCTCTCTCTCTCTCTCTCTCTCTCTCTCTCTCTCTCTGATATTAACGTAACCGAATCACAGGAACCGCCGCATGGGAGTCGACCAGGCTGGTGCCGGGCAGGCTGCTCGTCGCGGCGCACCCCCTGGCGATGCTAGGTAACGCACTGCCAAAGTTTGAGGGGCTGGTACAGTTCAAGGATTGGGTTGCATACCTCACTTCTGTTATCGGATCACCTGCCGGTTACTTTTTTGGATACAAGCATGGGCAGTGGGGGGGGGGGGGGGGGATATAGTATGAATCTCCTTTGTCAATGATGTGTCTGGGTTGCAACTCTATTTGATGGCGCACTGCTCTATTTTACTATGGATTTATAATATCCCGCAGGTATATCTTGGTTGCACAAGAGTTTATTTTGTATTTAAGCCTTTGCTTGTGTCGCAATCTCACGGATTTTTATTCTCGCATATATCCTGTAGGTTTTTCAAACCATCCAAGACCTGCTGCGGCAAGCTCGCATTGGATAAACGACCGTAACGCAGGTCGCTCAAGTCTCCAACAGTTTTCTTCACCAATTCGAGAAGTCTGGTCTCTTCAGTATGCAGATCCTTAATCTGGCTCTCGACTGCGGTTAAAGAGGTTTCCATACTTTGCACCACAATACTCAGGTTTGTGAGAGGATCGTTGCCAACTGGAGTAGAGCTCGAGGCAACCAGCTGGTGACGCTATCAGTATTCATCTGGTTGGTTTCGGTGGAATTGAATATCGAGAGTAACAAAGTTGGGGATTCTCACCGCTTTCTCAACTTCATCTTCTGCGTCGTACTCGTCCGCCAAAGTCCCTATCCTATCCGCCCTAGTGACCTGTCTTCGAATAACGTTTGCGTGCCGGGATGTCTCCTCTGCGATGTTTTCGGAGACTACTTCTACGACATTACTCCTTTGGCGTTGAAGATCCCTATACAGGAATCGTACTTGAGCCGATGATCGGTGCTGTAGAGGAAATAAGGCTTTGAATTCATCCATGGAGATGATTGTAGGGAGCTGTGCTGGGGCTAACAAGAAGTTGCTCAGGATGGTCGATTCTGTTGGCGCCATGGTCAGAGCCGGCCTGATTGTACGAGATAGAACATCTCTGTTTCTCAATACAGTGACATTCGAGATGTTAATTAAAAGGCGTGCTCAAAAATTATTTCACTGCTCCCACCCATCTTGGCCTGAAATTGACATAAAGGCAGCGGAGTGGGCGTTTGTCATTTAATGATCGACGCGTCGAAGTAGCTTCTTGACACGTCTAGAACGCGTCGCCACTCGCATAGCGGTGGGCCTGTCAATGGTCGTGCAAAGCAAACTCCCGTGATCACGGGATCACCCTCAACCATCCTTTTCCGCGTTCACTCCCTCTGTCCCTTTCCATCTTGCCCAAATCGGCCTGCATCGGTCTAATTCTATCATCACCAAATTGCCTTGCCAGCAAATTGCCTTATTCACCAGAGATTTGTGCAAGTCTCTTTGTTTGTATTGGCGCATCCGAACCACTAGTAATTGATTGGCATCAGACGTCGGGTACTCTTTACACAAAGCCTGATCCGACGTGTTTGGTTTGCAACAAGGGAGGCACTAGCGCGTTCTGGAAGCTCAAAACACCATCAGCAGAGCTGTCATAAAAAAAAAAAAAGAAAAAGAAAAAAAACCAAGACAAACCCAAAGGACAAGGCGGGTTAATTGTCCAGGTCTCTGAATCGACAATAAATTCATCCTTCGCCGCATCAAAGACTTGCAAAGATGCCGCCAAAGCAATCAGCACCGCCCTTCAAGGCGGACGAGAGGGTATTTTGCTTCCACATGGACATGCTTTACGAGGCACGCATCCTCGAGATCCAGAGCGAGGACAACGACCTGTTCTACAAGATCCATTACAAAGGATGGAAGAACACGTGGGACGACTGGGTGCGGCAGGACAGGCTGCGCAAGTTCAACGACGAGAACAAGGA

**> MD108_chr01:2661882-2665882**

GATATCACGTACGGATAGCACAAGATCGCTGGTGTACAGAAGGTATTTGGAATATTGTTCTAGGGTTTGCTTGAAGAGTGCAGGAAACCCATGGCAAGCTCTTGATATTTACGAGCTCACTAATACTGGGACAGAGCCTTAATATGGTGCATACTCAAAGGTATTCAGAGGCATGCATGCCAGTCAACGTTGCAGGACAGGGTTGTCTGCTTGAACGGTTTGTTGAAGCCACGGGCAGGTTGTTGATACTCATAACGATTTCAAGACCACCAGATGTATTCATTAATATATACAAATACTGTTTCACGTATCTAGAAGCTAGTGCCTCCAACCTCCGTAGGAACCAGCACAGTCTACAGTGACATAAACCCATACCAGCTTGTCTACGTCCAGCGGCAGCTGTTACTGGGCAGAGGGTGAATGGCTGCACAAACGAAGGACTGTGATGCTCTCTGCAGTTTGAGATTATTCCTTTTTGAAGCAAAATGTCTCCTGTCTAGCCACGCCACGAGCACAGTGACACAATCATAACCATCTAGTTTCATGTGTGTGCCACCCTCCCAGACCAAAGGGGGTACGTTGTTGAGGCTTCTCATTGATGCCGAGCTACCGTTTTGAGGACGAATAGACTTTCCGAGACGTGGTTTGTGACCTGCCATACCATAGTGTCTGGGAAAAAGTGACTGCGCCTTATAGAAGGGTCGCGTTGTCACGAGAAGGGGGGGCGCTTGGAAGGCTCCATGGATGACTGGGGCATGACTGGCGCTTAGGAAGCATTTTTTTTTTATGTTGTTCATGTACGTCCGGCAAAAGGGCGGCTGTTCCCTGGCAAAAAGCTCCCTATTCCTTTTTGTTTGGTTTGTTTGTTTTGATTTCGAGTGAGGGGGAAACTGTGTGAAGCACAGTAGAAACCCACAGCATATTGATATCGACGACTCAGGTTTCGGGTATATTAACATTGCACCGATATTCCCTTCCCGGGGACGCCATATTACTGAGGCAATCTATCTAACGCTTTCATCTCGACGAAGCTCCAGGTTGGCAAAGATTGCGAAGCTTTTTGAGCCCATTGTATCATAACTCCAATACGTAGGTAGTTCTAGAACTGCATAACAAAGGCCGAGTCGTTCGCTGTGCCACCTGATCATTGCATAGCATAAACTTTTAAACCAAAAGAACACGATTTGTGCCTTGAGCAGCAAATTTTCACACAGACCGTAGTGGTGAACAATTTCTATTACCTTTCGCTACCTCTTTGAAGAGAAAAAAAAAGAAAAAAAAGAAAAAAAAGGCTTCTGAGGCGACAAGCAGGCTTCATTCTGGCGCCACGCGCATGTGTTGATTCGTGCAAGAGATAGTTAGGCTTTGGTATGGCATGGCTTGCATGGCCGGTAAGAGCGAAACTAAAAGAAAAAAAATAGAGCAAAAACGAGGACATCGTGTCGTCTTGCGGATAAATAATAAATGCACATAACACAAAAGAACGGATGATTTATGGGCACGCCTCTCTCAGCCACAACCACCGCACCCATATTTGCGACAGTCATGGCTTTGGGTTTACCGGGCTGGGTAGGCGGCTTTTTTTTTTTTTTTGTTTCTGTTTGCTGGAAGCAGGGGGGGACCAAAGTGAGTAACCCTGCTAAGCAGAAGAATCTAGATTCACGAGAGTACCACACCCCCAGTAGTCCTGTCAGGCCATTCTCAAATTTGCTGCAGACAGATGTCGAAGATCAGCCGGCATGTTTTTCGCGACACGTCTTACGCTGTTTATTTCGGGCGTGGGGTTCGTCAAGTCTCTGAGACTCGTACACATGTCAAGTCGCTTGGTCACCTGGCTTTGTCCTTTTTCGTCCCATCTTGGCTTTATAAGCTTCA

GCGCCGTCTAGGGGAAGGGAAGCTTGTTCGGTTTCCACCGTGTCTCTGAGTGGAGTTTTGTCACTATTCATAGTATTTTTTCCGGCGATCGAAAAAAAAAAGAAATAGAAAAAAAAAAGAAATAGAAAAAAAAAAGAAATAGAAAAAAAAAGAACAAAGAAAAAAATGAAGAAGCTCTGGAGCATCACTGGCGTCGTCGTGACTTGGGTTGAAAGTTCCGACGGCGACAACATCCGGCGGCACCGGGGCTTTGATAGGGGATGGCCTCCACCGCCGTAAATTTTGATGTGCGGCGGAAAA

AGATGCAACGTGGTATCTATCTCGGTTCTTTTCACTTGTTTCTGTCAACTTTATAGCCGAGTAGATTAATCACAACGCTGTTTACCTCAAACTGATGACCACTGGCAGCTGTTGGTCAAATACCACAATAGGGTTGTAACCGAGATTGCCACGGATAATAAAGTGCGATTGTCGAGCAAGAGATAGATTGACCACAGCTCGAGCAGATGGATCAGGGTCCTCGGAGACGTTGTATCGCAGGAGCCGATCAAGCCCTTTGAAGTTCTAACCCCTTGGAGTCTCCATACACACCGGTACTGGGCCCCGTCTTTGGAGGGATGGTTGGTATTCATTGCAGACTGTTTGTCTATCAGGCCCCCGCAACCCGCCCGACAAACTTACGCTCTGCAGGAGTGCCGGCCCACGATGCCACTGGAGAGCATCGATCCCTTGACAAGGGGGACGGAAACAAAGACAGGTGTGCCGGAAAGCTCAAGCGTACGGATCTCCCCCACGCATCGAAGAATGCAACTGACTGACTAGGGACTTTTTTTTCTCTTTTTTTTTTCCCCAGCTTTGCATATCACCGCTGAAGAGGGTACATCATGTGGGCAGGATCTGGCACGGTTTGGAAACGAGTCAGGCCCAACACAGCTCCCCACACGTTGAACCCAAAGATAGTAGTATCGGTCGCTTGAATAGGGAAGGCAGATCGCGCGCGGCCCGCGCGCCTGTTTCAGAATAAACGTGAGAGATGAGACGACTCCGCATGTGGAGAGGCGCCAGCCGCGTACCGCAAGTGTATTCTGAGCGGGTGAGGTGGGTTTGGGTGCGATCTCGCAAAGTCCGAGTCGGGCAAACGTTGGAAGGGCGACACTTTTGTGCTGTCGCATTGTTCATCCCAACTCGTCCGAGGATAGAAACTTGTGGCTGAAGTGCTGGACCCTGCACTTGACTTGATTTGTCTTTTTTTTTGTAAGTGTTTATGTTATTTTTTCTTCTTTCTTTTTTTGTATCATCTCTGATCGCTTATGCTCCCCTGCCCCAATGTGAGTGAGATTTACCTCCTATGGTTCTTCTAATATCATACAACCAAAAAAGGGAGCAGCAGCTGCCATAGCCCCGTCATCCCATCCTCTGGGACACCTGGCATAGATGACAAGACCCGCGCATTTCCAGGTCTAGGTCAACCGGTAGCTATGCAGATAAACAGCTTATTTGTCGTGGATAAGGAGGATCGGTGGGTACTCTTTTTTTTCACATAAACATAATTGAGGGAAACGGTTTGATAGTTTTTAAAACGGCGACTAGGAGACAAGGAAAAAGGGTACCGTTTTCCGCACATCGGAATAAAACAATTGAGTGCGATATCGTGGGAAGCGATAATGCATTAACAAAGATGATAATTAAAGGGGTTGGTCCGCAAGGACGACAAGCCCAACAGAGAGTTTGTTGGAGGGGCCAAGACCAACAGACAAGAGATGATTTGGAAAGATCGTGGAAATTTCCCAAAGGGTCTGTCTGTCTCTTTCTTGGTCGGATGGAATGCCCGGGCATGGCATTCAACCAGTTGGGCTTGACCAAATCCCGTAGCCAACGTAGCGGCTCCGCAATTTCCCGTCATGGCCACGGGCGCGGGGATTTTCCCCCTCAATAATCGGACAGGAAAAAAAAAAAAATCTCGGTGTTACTTTTTTGTCACCTCAACCCCGTGATAGATTAATTCGGCCTCGGGAAGTTGCAAGCTTGGGAATATGCTTTTTGCACCAGAACAGGTGGTGTGATTACATCCCATATTCAATCAACAAACCCCTGCCAAGCCTACCTCGGTTCAACATACCTACCT

**> MD109_chr01:2769158-2773158**

GCACGGGGTCGCGTCAGTGAACTAACCCCCTAAGCAGGACCGGGCCCGAACCCGGTCAGGCACGATCCGCCTCTGCCCTCCTTGTTTTCCCCCTGTGTAAATAAAGAAGATAGAACGCGCGCCGAGATACCCCTCGGGAGGTTGCTAACGGCCGGCTAACAAGCCGGGCCGAGCCCGGCGTTAACTAATACTACTACTACTACTACTACTACTGTTCTTTTGCCAGCCCCCATGACATTTATGCCCAGCTTACTCCATACGTACCTGCCCAAAGTCGGGCTCACTAGCGTTGCCCACTCACAAAGCCGAGTGACCTAGCCACATCTGCGGCTGCCTCATCGAGAAAAAAAAGCGCCGTGCGTTGCGGCGTGGCTAGGAATGGGACCTGCTGCTCACCTGTGGAGGCCTGCGCAAACACGAGAATAAAAAAAAAACATGTCAAGGCTGGAGGTCAACCTTGCTTGCATTAAAAAGTCGTTTTGTCGGTGAACATGACTGGTGAAGGATCTTCCTGGCAGATGGCCATGGGCCCGGACAGTTGTCAAGGCGTCTTGGCAATCGCCATTCGGCCGAAGAAAGAAGGCAACTATCGGTTTTTTTTTTTTTTTTTTTTTTTTTCTTTGCACCCAGGGCCGTTGTGGCGAAACAAAAAAAACCTCAAAGCCATGCAAACAGACCTTTTGGGGGCCAAAAAAGAATAGCTTGGTCAGAGCGAGCCGAAAAAAAAAGACAGCGGTCGGGCGGACGAACCCGAGGACCAACACCTTCACCGGGTACCCGGGACTGTTCACCGCAAAAAAGGTCCAACGCATCGGTTCCCACGCGGAAGCAGGACACCCATTCCCCGCCCTTGTTTCCCTGGAACTGTCTCGGCATTGCATGTCTTTTTTGCAACATGGGAAACAACGGGGGAGAATGTGATGGCATACCGGAAGACCAAAAGGGGGTGGTTCCCCCATGCATCATCCAACTGTGGAAACGTTGCACAAATCTCCAAAAAAGAAGTTGGTATCGCGATGAGCCGGGGCCCAGGGAAACGGGTTGCCACAGCTTCGTGCGTATCTTGGGATATTTCGCCCGTACCACCAAAAACCCCTCAAGATTCTTTTTCTTTTTCTTTTTTTTTCCTGCAGCCTGGAGCCATGTGTGCACCCCCTCTTGTTTTTTTCTTTCTCCGGAATGAAGGTCAAAGACAGCGGATACAATCGGCAGGCACAGAGTAATTCCATCCCATCGGCAAGCTTGCTCCCGTTCTTTTGGTGCCTCCCCGGCAACTCGAACGTCAAGTGTTTTTTTTATGTGCGTTAATCATGCAGTGGTTAGGTGCGTGTACCTGGTGGTTCAAAACTCAACCAAAGACAATGCGTTGGTCTATTGTTTTATTTCTTGTAGACTTATCTTTTTGGACCAGGGCGATTCCTCAATTTACAAAGACATTATAGCAGGTGATGTAAACTCAACTTGGTTCTGCTCAAGCCCAGGTGCGGATCAACATGCCTAGCTGCACGCTAAGCGCTGTAGCGTGGGGAAAGATCTACGTGAATTTCCTGTCCAAGGTGGGAATCTATACCCTAATGTAAGATTTGGCCGAAAATGGAAGGCGTGGCTTGGATTGATGCACCACCGTAGGCGGGGTGACCTGACCTTGGAAGAAAAATACATGTACACATTGATTGGAAGTATATGTTGGCCTGGAAAAATAAAGCCTTTTTTTTCTTTGGCAGCTCTCGCCGTCTCCCCCGGCATGAAAAAGCAACAGAACAACCTAAATTGAAGAATGATCTAGGCCAAGGCCCAGAC

TTTTTTTTTTGGTTGAAAGCTACAAGCGACCTAGAGGCGCGGCCCCGCCCCCATCTTAGCGCAGCATGTACCTGTATACTACACCTCGACACATAAAAAAAAATGTACTCACCTTTTTGCAGACAAGCACAGACAAGCGATGGCCCGCAAGTCCAGACCCGGCAGGACCTGACAGAGAAAACCGCCGCGATGGCCGATGTAAAAAAAAAAAAAAAAAAAAAAAAA

GACCACAAAAAAGAGATGCAGATTAAGATTCACCGGTCGGCTGCTCTAAGACCCAGAAACCCGGACCTTTTATTCCCGTTCCTTTCCACCCCTCATTTTTTTTAAAAACACGCTGCACGCATGGGGGAGCTACCCAAAACCATGGAACAGGGTCGACGGCATGCTGAAGAATCTCGACTCGCACAAAGAAGGGTATTTTTTGTGGCGCACAGATGAGAGTAACAGAAGAAATAAATCACAAAGAGATAGAAAAGAAGAAGAAAAAAAGCAAGGCGAAAGAGTGTTGGATACGGGGTACGAACCTAGATTAAATCAAGCCTTGTTTGTTCTCCCTTTTTCTGCTTTTGCTGCTGGCTTCCCTAGTCGCACCCCCCTTCACCTAGACCCCACGGCTGGGAGAGATCAAGCCGTTGCCCGGATGTGCAGAATGATGTAAATGAGAAAAAAAAAGTGTCTTGACAAATTTCACGGGAACGGGGAAAGCTTGCGCACGAGAGGATCAAGGAAAAAAAAAAGACGCGGAAGGGTAGTGTGCATTGTCTTTTTTTTTCTCCTTCGTTTTTTTTTCCTGGGGGGCCATGCCGTGTCTTCAGAAGTCTTGTCTCTCGGACAACTTGGGACCTGCACCAACGGAGTGGACCGGGGGTGGCGGTTGGTGTAAGAAAAAAAAAAGACAAGAAGAAGAGACAACCTACTCGCACGTCTTTGTACAGAGTATATGTGTGCTGCCACGTCAGTACCTGTTGAGTATTGTTTTTTCCCCATTCCCCATGTTCACCTCTTCTTGACCTCTTTAATCTCTTTGCTTTTGTAGCATACGAGTCTGTTGATCTACCTTGATCTACCTGATCTACCCAGTAGTCCACGGTGACCGAAGCCACCTTTTATTTTCCCCTTTATTCCGGTAGGCCCAGCGAGAGAACAAAGAAACCCAAAACAATATTTTTTCCCTCCCTCTCTCTCTCTCACCTTTACGAAAAAAAAGATGGCTACACTTATTCCACCAAACGGCGCTGGAGACGCGGAAAAGGAGTCATGTACGAGCCCTTCAAGTTTGAGGCCCTCGAGCTGGGTGCACAGTATACAAGGAAGCCAAACAGACCTGGACTCGACCAGTCCATCCTTAAGAAAACAAGAGGGTGCAGCGAGCCCGAGGATATCGAGCGATCTCGCCGGCACAGATACAGACACAGACACGGACGAGCATGAAGAGCCGCCGCTGCCTTACTCAAAAGCCAGGTGTGTCGCGCTGGTCGTGACCACCACGGGGGCTGCCTTCACAAACGTGAGTACTCCCCATAACCCGCCAAGGTACTTTTGACCATCCACTCACTCGCAATATTGCACTCTTTTGTCACAGATTCTATCAATCCAAGCAGTCGTGATTGCCCTGCCGACCATCGGCCGCGATCTCAACATACCAGACACGAGACTCCAGTGGGTCGTGTCTTCGTATGCTCTCTCCTTTGGCTGCTTCCTCCTCCTCTGGGGTCGCATTGCCGACCTCTACGGCATGAAAAAGATCTTTGTGCTCGGTTCCGCATGGGTCGCTGTCACTACGGCGCTGAACCCCCTCATCAAAAACGAGATTGCCTTTGACCTCTTTAGGGGCCTCCAAGGTTTGGTAGGAGCTGCCACGAAAAAAAAATCCTGCCAAGTGGCCCCTGAACCAGAAGCTGACTTTTTCGTTTCTACAACAGGGAGCTGCGGCCAATGTCCCCACAGCCCTCGGCATTCTAGGAACTACATTTCGGCCCGGCAAGGCCAAGACGAATGCATTTAGCGCATACGGTAAGCTTCCACGCCGTTGCTGTCAATTGTCCTGTCCACATGCAAGCATGAGCTAACCAAAACCCCTCATTTAAACCAAACCATAGGCGCCGGTGCTCCGTTAGGTAGCATATTCGGCACCCTGATGGCCGGCTTCATCACCGCCAACGTATCTTGGGTCTGGGTTTTTGGCGTCACGGCCATC

**> MD110_chr01:3709451-3713451**

TTTCTCCTTCGTCTCCAGCATTGCCTACCTAGAGGGTGTCGAGCTTTGGGGTGCCGTTGTTCTTGTTTTTTTTTTCCTTTCTTTCTTTTCTCCATTCCTTTTTGCTTCAGCCAGCAACCGCGCATTGGTTGTGACTTGAATGTTTGGTTCTTTTTCTTCTTCATCTTTTATACCTCGATTCATACTATCATTACATGCGCGTATCTGCCCGGGTGGTGATCGTTGCTTAGTACACTAATACTCTAGCATTGACTGCACATTAATCACCCACCCTCTCATCACTCAATCGAACGACAAAGAACTCGTGCGCGCTGTTGGACGGCAAGCACAACACATCACCTATAATCGACTCAACTTGGCCAAAGGTTATCTCTAGGGTCTTGGCTACACATGTTCACCTGAAGTCCGAAGTTGACTGCTTGAACAGAGCAGCGCTGCTGCGGGAAGCGACGAGGTTCACCCCGAGTTTGCCGGGACGGACAGTGCCGACTTTTTGCTCTTCTCCACAGGTGTTGAATCAACTTGAATGACACCGTCCCCGGTGGCTCGAGAAGATATGCAGACGGTGAACCACTCATCCGCCATTTTTGGAAGATATAATTTCTTCTCAAGGATGGACGCAACTATGGAAGGCCCCGCCGGGGATCAAATCGGGACGCAATTTCACCACCTCAGCTACTATATCGCGCTGGCATGCATATTATTGGGCGCATACTTTTTCAAACTGCAGAAAAATGCCAAGAGGAGTCAGGTTGATGCGCCTTATTACAAGGCCTCGATATGGAAATGGATGTTTGACGCCGAGACCCAGATCATCGAAAGCTATAACAAGGTCAGCCACCTCTGCATGCTCCTTACGTGAGGAGCCATACGCGTTCGATATAGCCTTCTCGAAGCTTTGACACCTACAGGATTCTTACTTTCTCGTTGTTGTTACTAACCGCGGCTTTTGTGTTGGCAGTTTTATGGCAAAGTCTACCAAATAAAAGCAACAGAAGGCATGCAACTGGTACTACCACCGGCGTTTGTGGCAGAGCTGAAGAGCCTGCCTGAAGAGACGCTGAGCGCAACCGCAGCGATGTGTGAGGTACGAATACCATGTCTCTGGATCCGACTTGACAAGAACAGAACCTCCTAGTGGACCATTGTCAACATAGATATGCTCACTAACAGATTCGCGCAGGCCATGTTGAGCGAATACACCCACTTCCACTTCGCCAACAACGGCGATCTGTTGGCGGCTTTGGTGAAAACCAAACTTGCCCAGAACGCAACCAACTCCACTATCGTGCCGCGATTAAAAGAGGAGATCGATGTCCTGGTGGCTGAAGAATTCCCCGAGTGTCGCGACTGGACGACCTTCAAGATACAGCCCTTTACGCTCCGTGCGGTAACTCGCATGGCCGGGCGCATCTTTGTGGGGCCGGAAATCAACCGACGCGAGGACTGGGTCGAGACCAACATCAACTTTGTCGTGCACGTCTTCATGACCGTGGCCAAGCTGCAGTTCATGCCGCAGTTTCTGCGCCCGATAGGACAGTACCTCGTGCCCGACGTGCGACGCACTGCTGCCGATCTCGAAAAGGCCGGGAACTTGCTGAAGCCTATCATTGACCAGCGGATGCGCGACAAGGAGAACTTTCCAGGCTATGAGGCGCCCGCAGACATGATTTCCTGGCTGCTCGAAACTCTCTCCGACAAAGACCGGGCTGACATTGCTGTCCATGCCCAGCTGCAACTCATGCTTGCGATGGCCAGTGGCCACGGCACCAACAACATCATTGTCGACTGCATCTTCGATCTTGCCGATGACCCTGACCTGCAGGACGAACTGCGAGAGGAGGTGGAGGCTGCGTATGCTACCGGGGCACCCGG

GAGTGACTGGACCAAGGAGAAGCTTCTGATGCTGAAGAAGATGGATAGCCTCATGAAGGAGGTGCAGCGGTTTCGAGGCAATCTCAGTAAGTTCTCCTTGTCTGAAAATGAGCAAAACGGGATGGAAAAAAAAAAAAGAAAAAACACTAA

CCGGTTGACAACCAACAGTCGGCTTCCACCGCAAAGTCCTGAAGCCCATATCCCTCTCCGACGGCACCTACCTCCCTCCAGGAACAAAGCTGCTCGTCCCACAGGCGGGCATCTCCCGCGACGGGGCCTTTTACCAGTACCCCGACCACTTTGACCCGATGCGCTTCTACAAGCTCCGGCAACAGCAGCAACAAAGCAGCCGAGACTCGAGCGGGTCCGGGGACGAGGACTCGGCCAGGCACCACCAGTTCACGGCCATTGTTGACACCAACGTCAACTTTGGCGGCGGCAAGCACGCCTGCCCGGGCCGGTTTTTTGCCTGCACCGAGATCAAGCTGCTTCTGGCGTACTTTTTGAGAAACTACGAGGTCCGCCTCAAGCCGGGCGAGGGGAGGCCCAAGCCCTTCACCATGATGATGTCAAAAGTCCCGAACCCAGACGGCGAGGTGCAGTTCCGCAGGAGGGAGTGTGCCGAATACTGAAAGACGGGGTTTTTTTTGGCTGTTTTGTAGTTTAGAGGTCAATGAAAGAACCAGAAATTGAGTACATCGAGCGGCAAGAAAAAACTGTATTTGGATTGTGACATGTCGTTGTGTTGTCGAAGTGGCCGTCCTGGTGGTCAGTGGTCGTAGGTGGGAACGCCTGTCTGTGTCGCTATACTAAGCGAGCAGGTTCACTTGCAGCTTTGAATCCTAGATTAGATGCGCGCCGTGGGTTACTACCGTAATTACACAGTCGAGGCTAACTACCAAAGTCTCAAAAGGTCATTGGGCAAAATTAACAGATTTATTATTGTGTATTATGTATAAGCTTTTGTCTTGTCGTCTCAAGGTCTTGAATCACATAACCTATTCCCGGGGGAATGCATTATAGCGCTTTCCAACAAAGCTGCCGTTTCCGGCCTTGCCATCGGTGTACAGTGTCATCGTATCCGTCGAGTTCTGGTAAGACACACCGCACTTTTCCGAGAATCCCTTGAACCGCTCGGTCCTCTCCTTGATGAACTGCTCGAATGCAGCCTTGGAGCAGCTCGTGCCCGGTCCATCGTTGCAACCGGGAATCAGCTGAGGTGAGCTGTTGACCAAGACGCGATGGTACTGGCCGTTGTCGAAGCCATAGCTGCCGGAGCACTCGAGACGCTCGAGGGCGATGTTGGTCAGGAAGGGGACGATGTGCGAGGAGCGCCAGGCGCGGTGTGGGTTCTCGGCATCGGAGGGCATGGTGGCGTTGACGGTGGCTTCTGATCCGCCGAATTGCGAGTTGTTGAACAGGCCCATGGCGACCATGACCATGGGCGGAAGCTCGCGGTGCGTAAAGCTGATGTATAGGTCCTCAGCCTCAGGGCCGCCGTCGCGCGTCATCAGGTCGGCAGTCGCCTGCAACCAAGGCATGCCAATGTATCCGGCGGCGGGGAAGCCGTAGCCGACATTGTAGTGGTAGCGGACGTCCTCCGAGTACTCCCAACCGAGCCAGTCGTCCGGGCCAAAGAGGTCGAGGTCGCAAAACTTGGAGCGGCCTCGAATGAGGGACTCGTAGCCGCAAAGCTGCATCATGCCAAAGACGTCCTTGGTGGTGAAGTTGAACCCCGGGGCTGCAGCGTTGAAGCGGGCCTTGATGGGTTTGGTAAACTTCTCCTGGTACACCTGGCTCTGCTCGCTCCCGGTTGAGCCCGAGTAGGCCGGGCAGGCCTTGTAGGGGGTCAGGCTATTGGAACCAGACTCCTTGCCCTCGTAGATACTGACAACGCGGATGGTGTTATCCTCGCGCTGAAAGCCGCGCACGAGGGACTTGGCGGACTCAAAGGTGCGTTCAGCGGACGAGGTCCACACGTGCTGGGGTAAGCTGAGGTTTGGGTAGCGGAAGCTGAGGTCAACACCGAGCCTCGAGGCCTCGAGCTTGCCCATCCGGGTCAGGAGAGATGCCTCGGCCTCGGTGACGGGCGGCTCCCAGTGAGCTAGGAAACTGAGCGAGGGGA

**> MD112_chr01:3844322-3848322**

ACAGTCCGTATTCCTCAGCAACGCTGTGTATATATACGAAGTGTCTAGCCAATGGAGTCAGGCAAGTGTCCGTGGGATAATAAATCCTTCTGGTTGGGCTTGACGCTTCGTTCATGACACTGTATATTCGCTCTACCGCAGCCGAATATTTATCCACAGCGGATTTGTTCATGTGGTATGTAACCATGTAAGGGGTGAATGAGGAAATTTAGTATCATCCATTTTGGACGCAAGAGACCGAACTAATATGGTTCTTTTGATTGCTACAGTAAAAGCCCCATGTCAATGCCAGTCAGGGGCCAGGGCAGCAGCTCTGTCAACTTGACTCAGGGTCCACCTCTAGGGATCGTCAGTATGAAATTTTTTTTGGGAGAGTTGGGGCTTTTTTTATACCGTTCAAATAGATCCGTACGGTTACTATTGTTGGAACCTAACCAGAAGGTTGCTCTCAATCATACCTAGATAGTTAGTGGCACGTTTCTTCTATGGTACCTTGGTAGGGATGGCACCGCAAAATTGTTTACCTCCCAGGGGGCCCATCAGGGGTATCAGGCGACTTGCTTGCCAAATATGACCCAGAAAGAACCTGCCAAGCACGATATGTGGAGGCAAGGCGGAGCTCTGTTTAGAATCAGAATTGTTGTTCAGGATACATAACAGGATGAGCACGTTCTATTTTGCTTCCACCTGTGCTGGTCCAGGAGCGTCTTGGGACTCCGACAAGAACCTGCTGCTCAAACGGTTCCACCACAAAGAGCCTTGCCGACAAAAATGATTCGCCTCAACCTGGCTCTGGGCCTCTGTGCTGCCGCTGCATATACGCCTGCAGCATCAGCAGCGTCCGTCGGCACAGTAGGGAAGCGCGAGGTTGATTGCACCGGTGTCAAAGCAATCAGCCCGCTCTGCAAATCCAATGAGGCCGCTTTCCACCGAGACGCCTTTTACGTCGGGGGTCGCGACCTGAAAACTGACCGGGGAACCAGCACGGCCGACCAGCTCTATGTCGAAAAGCTAACACCGACAGGCGGCGTGACACAAGCCAACCCGCTGGTCTTGTTCCATGGAGGGGGTTTCTCTGGGATCACATGGCTGAACACGCCCGACAACCGTAAAGGGTGGGCGTCGCACTTCATCGAGCAGGGTTACGCCGTCTACATTGTCGACTCGATTGCCGTCGGCCGGTCGACACAGATTGAAGCGGCGGGATATGCCCCCGTGGGAGCCGGTAGCCCCGTCGAGACGCTGCGAGATATCTTCACAGCGCCTCAAGACGTCAACCAGTACCCGCAGTCGCAGCTTCACACTCAATGGCCTGGGACTGGGAGGGAGGGCGACCCGGTGTTTGAGCAGTTTCGCCGATCGGCCCTGCCGCTGAGCGTCAACATGACCCGACACGAGACTGGCATGCGCGCGGCAGGGTGTGAGCTGCTTTCGCTGCTCGGAACACCATCCTTTCTGATCTCGCACTCTTTTGGCGCCGTGACACCGTTCCTGCTCACCAACGACTGCCCGCAGCTCGTGGCAGGCAACATTAACCTAGAACCGTCAGCCGGGCCTTTTTACCAATTCAACCTGGACGGAAGCGGGCAGGAAGCCTTCCTCTGGGGCATGTCAAACACCCCCATCGACTACGACCCGCCCGTGACGGAGGCATCCGGGCTGAAGACCGAGTTTGTGGGTAATGCCACCGCAGTTCAAGGCCGCTGTCGGTTGTTGGCCGAGCCCGCCCCTAAACTGCCCAAGGTTGCGAGCGTGCCCTATCTGGCTGTTGCGGGCGAGGCGAGCATCCACATACTCTGGGACCACTGCGCGGCCATGTTCATCAGGCAGGTTGGTGGCACAGCAGACTACGTCAAGCTGGCCGATGTGGGGGTT

CGGGGGAACGGGCACTTTTCGCACATGGAGAAGAACAATCTGGAGGTGGCGCAAGTAGTGCAAGATTGGATTCGAAAGCGTGCTGCGCCACGCGTGTGAGACAAAGGACGCGTTGGCGTTTTGTTTCTCTCTCTCTCTCTCTCTCTCTCTCTCTCTCTCTCTCTCTCTGAATTTCGGATCACAACCGTGCCGCTGAAATGATAGCACATTACCAAGCTAACAAAC

AGCCCGCCTACCCGGTACCTACCCTAGGCAGGAAGATAAGGGCTCAAATTAAGCGAAACTAAGTGCCAATAGTATGGGGCACGTGACGGTGCTGTAGCTACTGATTTCAACGTGAGGAAGGAAGGAGACAAGATCACAGTGAATGCATCTTTTATCGCGGACGCGGGATTCGGACAGATGCTTGCTGCTCTTTACTTCTCTGATCTCGATCGAGCATTTCAAATGCAAGACATTGTTTCTGTTCGCATCAACTTTCCCGTCTCCGCACCTTGGCCGCATCAATCACACGTCAATGTCATATGGACGCCCAGACGGACGCCTTGAAACACCCCCGCCTGGCTCCAAATTATATCGCCCCCCACCTTTTGGGGGTCAGCTGCGTACGAGAGAATCACAAACTTTCTCCCAAGGTCGTATAACAAACCTTCAATCTTGGTCATCTTGAACGACCAACTTGGCAGATAATCCTTGCCCGATGAATCTATGAGTCGTTCATTTTTTTGAATGCAGGGGCAATAACAGGAGATGTTGCTACGCGTCGCGTCTCTGGTATCTATCTAGAACTAGGTGCTTACCTACAACTTGACAGGTCAGCCGGCGCTGGCTTGCACTAAAAAGCCATCCCGAACAGTTCGTTCCTGTCTATAGAACGATAGCATTAACAATGGTTCCAAAAACCGTCCTGTCGACATTACGGGCCACACAAATATTGATGATATTTGGCCGATATCGGTGACTCTTGTTCGAAAACGATGCTCTTAGGCCATGTTCAGTCAAGAAGTCACTCAGCCGGTACTTGTGTTGCTGAACGCTAAAAACCTACAAAGAAAAGAAACAGCAAAAAAAAAACCAAAAAAAACCGAAAGAGTGTCGACAAAAGAACCTCCAAAAAGCATCGATAGCTCTGGTGCGGCCTGGCGGCCGCATAATGCAAGGTACGCGGCGACAAGATCGACAGGGAACATGTATACATCTTGGTGCATCTACCTGCCGCTGCAGCCTGGCGATATTGGTGGACTGCAGCCGTGGATTAGCCCGACCCTTCCGTCCTTCTCACGTATCTTTTTTTTTTTTCACAAGTGTGTGATACGAGAACATCCCGGGACTCATAGTCTATTATCAGCCTCCCACCCACCATATCAAACCGGTAGCGTCGGGTTTAGACAATATCCACTAGAGAATATATACACTAAACAATCGGCAGTGGTGTGGTGTCCACAGTCACCTGTCAACGGCCTTCATATCTCCTGGCTGATCAATCCAAGGCAAGAACTATTCGTGCCCGGCTAGCTACACTCCATCACTGTCTTAGCCCCGTCACGAGGACGACAACATCACCCGAATTCCTCCATGCTAACGGCCCGGTTTTAGATGCCAAAAACCCAGCTTTTGCTAAACCCAGAGAAACGCGACTGAAGAGGCGCCGTCGCAATCTGCACCAAGCCCAAGCCCCGCACGATGATTCACATGTATGCAAGCCTACCACTTTAGCACGCCGCGACTAAATCCAACAGATGGCGCGTCTTTGCATCCTTGGCGTGCGCCATAAACAGTTTAGCTCCGGCAGAGCTCCTCTTGGTTCTTTTGCCAGGCCAGGCAAGCCAGGGAGGCACCTCTGAACCCCACACAGCCTTCAGCCGTCGTGTGAATCACAATCCAACCCCCAACGTCTACTTATCCGGTTTCTGCTATCTACTTGGCTGGCAGAAGGACTCGGGTTTACGCACTCACACGAATGTGCCCCCCGTCGTCTCGTCTTGCCCGTAGAGTATTATGTGACACCCAAGCTGAGAATGACCATTCTCCACCAGCACGAGACAGCGACGACTTCCCCGCCGGTGACTTACATGCTAGCCCGGGACTATGGTGCTTTTTTTTTGTTTCTGGCATTTTCTTGGTC

**> MD113_chr01:5555275-5559275**

CATCACCCTGCTCGTGGGCGCTTGTGTCTGCGTGACGCAGAGGCATGATATGCTGGCCGACCTGCCCGCGTCCAGGAAATGAGGCTCTTGATCGTTCCTGGCGTAGGGGTGACCTTCATACTTGCGGTCCCATAGCTCAGTTGGTTAGAGCGTCGTACTAATATGTTGTCCAGAGATGGCATATCAAGTTATTGAGTGAATGCGAAGGCCGAGAGTTCGACCCTCTCTGGGACCATGATGGCCAGCAGGCTTTTCTTTTGGCGCGTCAATCAAATCCCTTTTGTGGTTTGTGCTTGGAGCTCGCAGGCTCTGCTCTCTTTTTATCTTGGGGCCCTCTTGTTGTTGTTGCCTAGTTGCCTTTACTATATGCTCACTTGAACCTCTGGGCCATCCTTTTGCTAGTGAATGGGCTCTTCCTGGGTAGGAAACCAGATACAAAGGACGCCGGAAACTGGGCCGATAACTGAACCTGGGTAATGGATACGGCCCCACCGAGTACTCCATCATCGTCCGTAATGCCCCGCCTAACGAGGTGATCGAGGCACCCGACGTTGGCCATGCCTTGGGCTGCATTCATCGGCTGGTAGGACCCGCATTGGAGCCCCGAGCTTGTCCTTTAAGGCTCTCCACTCTCGCGTGGCTACGTGAGCGACAGGATGCATTGGGACATTCCGCAGCGATGGACAGTACTAAGAATACGCACAGCGTGAGACTGTCATTTGTATTAGCCGTCGCCGCCTCGCTTCTGAGCGTCGATGATCATCCACTTCCCCGTCACGGGCTCCACCGGCTTCTCAGGCGCCAGACGGTCAAGCAACTCCTACCCCGAAGTGAGGCGACAATGGTGGTCTTTCTCGTCCGCGCAAACTACGACATGCAGGCTTAGGAACAAATGGATGTTGCCCTCAATGCTCATTGGAAACACAGCGATGTAGTAGGGGTGGGCAAGCATGTCTTGGAGGACAGTGGCATCTGAACCAAAGTGGTGAGCAGGATGGAACGCATAAATATGGCTTGCAGCCAGATCCCAAAAGTCATTCAGCTGCGGGACCTGGACTCGCGCAATTCGAGCTTCCCTGGAAAGGATACAAAGCGGGCGAGGCGTCAATGGTTTATGGCCCTGGGAAATCTTGACAGTTTACCGACAAAGTGGCCAGACACAAGACCACGTGTCGCTTTGTGAAGGTTGAAAGGATGAGAAAATAGCGGATTCACAGAGTCAGAACACAGGAGACGTCAACAAGACTTGTCGCCCAGCGACGTCGTGCGGGGAGTTTGCGGGGCGATGCTCACATCTCACGGGTCCAGGGGGATCAGACGAACTCTCCGAGCCCGAGCAAGCATCGGTTGAACACCACGACGTTGGTTAGCCCCTGTATCCTGCTGACCCGGTGTTTTGTCGCGTATGAATCATTATTCATTACACGAGGTACTCGGGAACCTTGACTTGGTAGACGGCTAGATTTCCCAGATCGGATCATCAGAGTGGCTTTCTGCCAGCATCTCAGGCCGAGCCCGATCTGTAGGCGCTCTTGGCCACTTCAACAAGCAGGGTCAGGGGCTCCACCTTGGAGGGCTAGAACCAACAAACGCTTCCCATTATAGACTTATACTCAACAACCCTGCAGCGCCCATCTAGATTTGTCGCAAATCCAGATTACCTTTGGGCTTATCAAACCCGCGAATCAGACCTCATGTTACAGGTCGATCTAGTACAGGCGCCTTTTTACCAATAAAATTCCGGTTTCAGCCGCAGGAAAAAGCATGAAATCATGAATTGAGGTGATTGACAGAGCGTAC

GATTGGACTTGGAATAAATAGAGGACGGTTGCCCAAAGAGGTCAACCTTCACTGGTTAGATCCAGGCCAACAAAATAGCCAGCAGATATACAAAGTCATTGTGCTGTGCGCAACACTATTCTCTGCCAACTCCTTTCTGCTTTTTGTCCCGTGTATAAAACAGCTCTCGACGCCGGAATGCACTTTTCTTCGCTGCTCCTTGTCGTCGCCTCTGTCGTCGCCTCTGTCGTCGCCTCTGTCGTCGACCTCTCAGGGGTTGCCGCACTCTCAGCTACAGAGCCCGGAATCTCAACTATAGGG

CTGCCTCATCTATTATTCTCAAGACCAAAGAATGCGTTTTCCGACGAGACAGACTCAGAAGGGGCTACCTGTCTGAAGAAAAGAGAAATGCCCCTTTTTACCAAGATAATAATAATACAAGTTACTGTCGGTGCGAAAGCCAGAAATACCACGGGTATTGGTGCAGGCCCGGCGGGTTTATTTGCCCCGTCGTTGTTGGCAAATGCGTAAGTAGTACTTGTGCCTCTTATCCTTGGGGTTCTGGAAGAAAGCCATGCTAACTTTGAGTTCCCACTACAACAGAATGCCGGCGCGTGTTGCTTCAACAGGATTTCCAATCCCACAAAAGGCTGCCCCTTCGCCTAAACAAAGATAAGGAAGCCATTAGTGTGAGGTCTGGGGTACCGTTGGGCCCTTGATAGGACCATACTGCCCCGGGCCGCCAGGTCATTCGAGTTGTCCCGTCTCAGTACAGAGCTCAACCAGATACCACTCTATAGAAATAATATTTTTTTCAATCCACGACAAAGCACCAAATCGTGAGAGTCGACGCAGCTACATTACTTGGTCGGCGGCGGGCCACAACCCAAATAACGCTTCCATGACATGAGGCAGCCTGCCCGCCAGCAGTTCTCCCATAGCCTCTTTGATTTACGGGCCAGTCGCTACTTTCGCCAAAATGCAGCCTCGCACGGCGAGTGGCCTTCTTACCTCCCATCGTCTAATTGACCGGTGCGGTCAGCACAATTTTTGTCGCTTCCATCTGCCAGCCTGTATTTTCTGTGTCAAGTGCGGGATGGCTGCTTGGTGAACCGGCTCTCTGCTTTCGTTTGCGCGTCGTCCATCCGGGCGCATCTCAGGCTTGCTGTGCTTGCTACGGCCGAAGAATCCCAAGGACAACAGCAGAGACAAGAACCGGCCGAGGCAGAGGAGACTGGAGAGAGCAGGGTTGCAAGGAGAGCAACAACACAAGGGAAAGACGGTCAAGCTCACGTTTGGCATTCGACCTACATGGAACCCGCATGGCTATATTGGGAGTCCAAGATTGATTGCTAGCCACTAACTGTACACCGAGCAGACATAGTTTGATATACTATGCTAGGAGTCCCAGCTAAACCATCGTTTGATTTGATCTCTCAGATTTAACTTTATTTTAGCTTTGAGCTTTACCCTTCTAGTCAAGTCGAGTCAAACAATGTAGCACGACAAAGTTGGTGGCAATCCTAGCAGTCTTATGATTTTAACTTTGTGCCATCATCACCTTGCCTTCTTCTCTTCCGCTGGCAATGTATTCATCCTCTTGTGCCTGTCGGAAATTTCCCCCAGGTATTTATGTCCTCAATATCCCTGCTGCGCGGTACCAGAGACAAGGCGTGTTGGCCTCCCCCAATGGGATGTTTACCCTTCCCTCACCCATCAGATCAAAAATTACCATGCTACACACATGGTGGCGTTCGATGTCAGCGGAATACCTCTTTGCCATCAGTTTGAAAGATGTGGCCACCTCGACCAAGTTCGGCCGTAACTCTTGAGGTTGACGAGGGATATATCTTGGAAATGTGGGTAAGGACTTAACGACACCCCCGAGCTTAAAAAGGTGGATATGGATACTTACCGGGCTCACCACAAGCATCCTCCACCATTCATGGGTCAGACCCACCTTTGTTAGCGATTCACACCCAAACACACCAATAAGCTTAACAGCTGAAGAATTTTCGCCCTCCGGGCGCTGGTCGTCATGGCCTCACCAGAGGTCTCAAACCGCCGCGCGCGCCCGCAGAGCACGCCGCAGACTTTGCCGCAGGGCCAGGCCGAGTCGGGACACGTATCTCTCGACACGCGCACCGCTAACGCGCCTGAGCCGGCTGGCCGCGGAAGGTACTCCAGCCTCCCGGCCCTGGCCATCAAAACACTGCAGCAG

**> MD114_chr01:5964005-5968005**

GTCTGACAATAAATATACATGTGCAGTTGATTGCTGATCGCACTCTCACGGCAGACTGTGACGAAGACTACGGCTATCTCTGACTAAAGTAGACTTAAATATACGAAGAGCTGTCTCTTCCAGACAGTACTTGCTGCCTGTGCAAAGTATCTGGCACTTTTCTTTCCTCTCTTTTTCCGTCTCCTCACATGTCGACAACAACTACAAGCACTCGCAGCTGCGAGTCTGATGATAGCCTCACCGCATCGAAGACGAGTGGTGCTCCGTGAGTGCAGACGAGCTCGAGGACCTTGAACAGTCCCGTATGAAGCTACTGTTTGTTGAATGCTGTGGTCCAATAGCTTGATTATCAACTAACATAAGGCTCCGTTGGAAGAAACCTCTGTATCCCAAGAAGGACTTGTTCCATTACAAACATCCCACAAAGGGAGCGTCAAGAAGCCCGCACATCACAAGAACCCCAATGTCGGCGGCTCATGGACAGTCGTTACCCTCCAAAGCGCCCGAAGCGACTGCGTACACGACATGGATCGCTCCGACGTCGAGCTCACCCCCAAGGAACGCCTTCAAATCGTCGCCGGCGCGTCGGCAAAACTAGCAGTGGCGCCGGTACAGGGGCTGATGGCCCTGAGCAGACGGCACGGAAACTGGACGTTCTTCATCATTGAAACCACCACCGCGAGGCAGAGACAATGAGACAATTGGAACGTTCACTACGTTTACGAGACAATAAGGCAAAAAAAAACCCCCGGAGCAAAACCGGTTTCTGCACGAAGGATACGCTTATGAGACGGGCTGCGATGGCCCAGGAGTTAATGTCTAATGACTGATAAGACTCCATGACGGTATGAATAGAAACAAAAGAAAAGCAATAATGTGGAGGGTTGTTTCATATAAGTTAATGGGGGCGAGCTGCCAGCAGGGTAAACAAGATGTTTTTTTTCTTCTTTTTTTCTTTCCCAATGCAGTGAGCGATTGAGCTCAGACCTAGAGAGTAAATGAAAGGAATGATGTCGACGGGAAATAAAATTAATGGAGTAATATGGTGATATCAGCCCGATTACTTCTATCCAGAATATGATGCGCAGTTCAGATTCACTCCTTACAACAAAAGAGCCATGATGTACGCAGGGCTCTTGTTAGGGATATCAGTGAATCACAGAAAAACAGCACCTCAAAAACAGCCTGTAATGAATGTTGACGCAGTAAAACACAATGATCGTTGTTCGCCTATCCGACAACATGCATTCTATCACAAGCAACATATTCGACCAGAAAGATATGAATGCCCGATCAAGCCATAGCCTGCATCATGGCAGAGATATCGTTCGCCCAGAGTATTATCGTAGTCACACCATCAGCCACCGGGATGGACCTGTCCGCTCGAATGTCCATACTACTAAACAATCGCCGTGCCAGCATCAGGAGCAGGAAGGCGAGCGACCGCGGGGAGGAGACAGAGTCACTGGTGCAAGCCAATACAACGGCCGCCGTCAAGATGAACGATCCTTTTTACATGTGGTACGTGACCGAGAATCTGCTACCAAACAGCCATTATGCATACATCTTCGTAATATGAACCACAAAGTGCACAAGAGCCTCACGAATGACGCCCGCCTCGAACGAACTGTAGCATAAAATATACACAAAAAAAAAACCAAAAAACCCCAGCACCTAATTCCAAGCTTCACAAGCCACTAACCGACACGGCCCTGTACGTCATCG

ACCGCAGGTTCCTCACGGGCGGCACAGCCCACAACATACCCGCCAACACCACCCGAGTCATAGAGATGGCCCGGGAGCGCAACGACGCCGCCGCCGCGGCCAAAGAATACGCGACGGCCCTCAAGGGGGCCAAAGCGGAGCTGAAGTCCAGCAAAAAGGAGCTCAAGGCAGGGTTCGAGTCGACCAGCAAGGAGCTCGACGCAGCTCACAAGGCCGAGTGGGGGAGCAAGCCCAAGGACAAGCGTAAATGGCTCAATGCCCGGCGTCAACAACAACAGCAGCAGCAGCAGCAGCAGCAGCAGCAGCAAATGGATATCGATATCACCGCCGAGGCGGCGGTGGCGCAGGAAAGTCGGCTGCTGTCCCGGGAGCCTC

CAGCAACAGCAGCAACAACATCAGCTGATGACGGCACCGTCCCGGAGGTGCCTTCAGCCGTAGATCAGAGAGCGGCAGAACAGGCACGTCTAGACATGCGGCTGAGCGAGCTTTATGAGCGCGCCACCCAGGCCATGGCAGATTTGCGTGCCATCCTTGACTCTCCAGAAATTGTCCAGAGAGGTACCCAAGCAAGTGCCTGACTGAAGCCTCTACCGCGACGGGGGAAAGATTGCTTCAGGGCCTTGTCTCGGTAACGTCTCCTGAGCAACCGCTAATGAGCATGCTCACAAAGGCTGGTTGATCGCCCCGACTGAATTGTCCAGGCAACGTACAACAAATATCAAAGAAGAAACACCTCTCCGCTACTTGCCGGCTCGTCCATTTCCACCTCTTCACTAGCATGTCAACTTCTTGGGACGATCACGACAAGCATATTGAGCTTCTATGCACGAAACCCAGACAGAGCTAGCACCAAGGGCTGATTACCCGACATCAACCGGTGCTGTCACGATGGTAAAGTAGATTAGGACAAGACGTTAAGAACAGTGGCAATTTTCAGAAGTGTACCCTTTTCTCCATAGAACCCAACCATGCGCCCAAAAATCATTAAACGAGACGCCCATCAGAACTTAGACCAACACATCCAGCGACCAGTATATACACACAACCTCCTCCGAACACTTTTCTTGGCCGGGGAACCGTAGCATCTAATCGTTGCTACGGTTGAATCATGGCTCCAAAAAAGGCTTCTGATCTGACGCATAATGTAACTCTGAAAGCCGTTGGCCCGATCTCCCCCTTAATCCTTCATTATTGTGTTCTCCTCCAATATCGAGCCCAAGGAATCATGAATACAAAAAACAAAAGATAAAACTCAGAAGTGCTAACAACAGTGCAAAAGCATGGGCACCCCATGCCGGAACGTGTGAATCTCTGACAAACGCGGTAGTGCATCAATGAACAATGCAAATGAAACAAGAATAGAAGAGAGAAATAAAAAGATAGACCCCCAGAGCAGCCCCCATAAAAAAAAACTTGAGGCTTGGGCGGCTTGCCATAATATCAAGCAAAGAAGACGCACACGCTCGACGTAGAACAATGAGTCTTCAGCCTGGAAATCAAATGGCTACAGACGCCATTCGCCGTCGCAATGCCATGTGTGCGAAAACTCCTGCAATGAAATTCGATAAACGGTGGGAAAGAATAACGAGGGTCGAGAAAAGAAATAAGCCGCACGACGGCAATCTTAGGAGCAAAAAGCCCAACGAATGACACCCAGTTGTCATGCGATAATATGCCCGGAGAGGCTACTTGCTGATACATGAATGGTGTAATGACCTCCGTGAATGACATGTAGGTCCAAAACTTCCCATCCAAAGGGTATCCTGGGAAAGGTGGCAATCTGGCACAGAAGACATGATGAAATGTCGAATTGATGTTTTTTTTCGAGGGAAATCTCCTACAGCTGGTCGATAGCTACGTCTGTTTGGTCAATGCCAAAGAGATAACCATTCTCCTCGACCCAGTTGCGTGACAGGATGGATGCTGGAAAGCTAGTTAGCGATTATGCAGTGGGCGTGATTGACGAGACGGATAACATACCCTTGAAGAATTTCTTGCTGGCATATTTCTTGAAGAATGCCTCATGAATAACGGGGCGAGAGAGGTAATCAACCATGAGGTTTACAACGGGCTCAACGTCATCCTCGTTGTAACCCGAGTAGTATGAGATGGTCTTGTCCTGCACCAATTAACATAAATTGTTAGTTCAAAAAACGCAGCACTCCGAGAGGGGACTCATGAAACAAAGAAAAACACTCGTACCCATTCACCGCGGTCAAGAATAATACGGGAGAGAGCCATAGCAGATGCGGCAACAAGACTGGGGCGGTACTGC

**> MD115_chr01:6216659-6220659**

ATTCATTGTGAACAGAAACATCGCCTAACACATGGAAGAGCGTACTAAATTTTGGGGGCGGGTGTCCAAAAGTATTGGGGAACCACCCATTTGATTCAGCGCCGAACAAGGGCCGCGCCGGGGCAAGTCGAGATTGGGGAAGTGGGGAAGCTTGCTTCCCATCCAGTAGGCATGGCGTGGAGATTACTGCTTTCAACCCACGCACAAAGCAATACCACCCAAGCTCAAAGGCAAGAAGAAGAAGGGTGGGACCCCGAACCACAGCCGCCGGGAGGAAGGTTCAAGGTTGGCCGGGGTCTGCTTCTTTGGCCTTCTGAGGCTTTGGGAATTGGGTGTGCGTGCTTGGCTTGGGTTTCGCCTGCCTCCTCCACACATGAATGCTATGCTACCACGACGTGCGTGGACAGCTGAGAGCCCAGGTCGTTGCCAGGTGAGGTGAGGTGCGCCTCAGGGTGCATCGTGCATTTGTACGTTTCCAGTTCCAGACCAGTAGTAATCCCAAAGCCCCATATGAAAGATGCTGCACGCGAGGTACGATCTATTAGTTCTAGTTTTAAATCTAGCTCGTCTGTTTACCTACCTACCTAGGCTAGCTGAGGTACTCGATGCGAGCGAGGTCAGCAAGGGTGTCTTATTAGAGACTATTCACATTGCATTTCCACCGTACTTTGAGGGGAGGGGGGAACCAACCACCAATAGTCAATCTTACCCAACTAACCTACCTAAGGCACTTAAAACACACTGGAGGAACCTTCATCCGCGCAATTCCGCATAAGCGAGCATCTCAGAAAATGGTCTCTCCTATGACTCCTTTGCTTGTTCTCAGGCGAGGCAAGTATTATAGTTTGTTAGCTATAACCCAGCATACCGCATATTCCCTTCTTGCAAAGACACCCTGACGGTTCTTCGAGGAGTTTACGGAAATGAACGTTTCGAGCTGACGGCATAGACGGCCGAAAATACCTGAGGGTGGTGACATGATGCTATAGGCCATGTGGAACGACCATCATCAAAGTTTCCAGGCGAGACAGTCAGTGCTACGGTACGGTACAGTAGTAACCTTTGCGAGTCTACAGCCCCCCAGTCGGTCAATTGATTCTGGTATCAATCACCACCTAATTAGGTGTCAAGTCAATCAGCCTTGACAATCCACCACCTGGATCGACCTGAAAGCGTCGGGTAAAAGCAAGCGGGCGGCACGCGGACCCCAATTCGTCCCAAGAAACAAAGCAATAACCATGGATACCATGGATCAAGAGTTGGACCTACCCCCACTGTTTATCGTCTATCATTTACCGCCCGCCCCGTCCTTTCCACCCGCGTCTTCCATACCATCCTTGTCAACTCACGTCAATCCATGCCGAGGTCTGTGCCGTCTTGACCGTTGAACGACGCACTGCACTGCACTGCGCAGTACCCGCACCGCACCTGCAACCATGCTTGCTGCGTTGCGCCCATCTAAACCCATCACAAAAAAAGGAGAAAAAAAAAAAACACCCCGAGAGAGGCAGTTCCGGCATGCGGGCTACCACACCGTCGGCTTATTCACTCACCTGCTTTTGGTGATAATGGTCTGGATTATCTGTTTTTGACCCTTAAGGACTTTTGATGCTCGTTCTTTGGTTTTAACTCCTTTCCTGTCGTGTCTGTCCTTCCTACTTTGTGAATTCTTTGGGGATCGATAATTACATACTATGATATCCAGCAGCCCTCTCGACAATCTTTTCCCCGATGCAACCCTACCCCTCCCTTCCAACCCATCAACGTGCCCACTGACCGCGTCCGGTGATTACAGTTTTCTACCGCGAGCTGCTACCCGCTTTGTAAGTAGTACTTCGTACTTTATCTATACTGCAGCGCCTTGAAAACTTACCG

TTCTCTGCATGGTACTCGTGCCTACTGCCCACGTATGTTGTGATGGCCTTGCAAGGGATGGCATGCAAAATGTTGGTTGCGCTTATTTCTGTCCACCCCTCTAGAACTAGACGATCTCTCCCTTATCTCTCTCTCTCTCTCTCTCTCTCTCTCTCTCTCTCTCTCTCTCTCTCTCTCTCTGCCTTGCATGCTGCTGCCACCTACTACGGACTGCGCCTCATTTGA

GTAGGTACTTCGTATGCTCTTAAGCATGAATTTAATGCCTCTCTATACAGTATCCAAATCATCTGCCTCTCTTGTCTGTGTCGAAATGCAACAGCTGACTGACGGGTTTCCAGGTGACAGGGGCGGGCGATGATCGTTCGCCCAATTACCCTGCCTTGCCCTGCCCATCCATGCACGCACCGTCCCAGGTAACCCCCAACTGCCTCGCCTTATTATTCGATGAACACATCCCTGGAAAGCCGTTGCCCTCATCGACCAATAACCAGCTCTGTATTAAGTGCGTGCAGGGACCGAGGCCCACGGCAAAAGTCGAGTCGACAATATCGGATATTATCACCAGATAATCTCAACTTTGGATAGACGAACGGAGGGGTGGGCAGGTAGACAGGCGGGCTGCGAATTAAGGAGAGAAAGGGTCTCGCTGGGCTTATCTAAGGTACCCAAGGTATGTTAAGTATTAGGTGCATATCTCATATTGTGAATGCTCAAGTCCGCTTATGCGACGTCGCGCTGCTGTCGGTTACGCAGTGCTTATCCACACCGGTAATTTTCTTTCTCATCTGCAACACCCCAAATAGGATAGGGCGAATAAAACATGATATTAACACTAGACAGTTTTGGGTCGAGAGGAAATCTTCATGGTAGGTACTACGTAACTTGTCAGCTCACCACCCTTTTTCAGCTGCGAACCTTGCTCGCTACGAAGTACGTTGGTACAGGTCGTGGGTGCCCTAGCTGGTCACAGAGCACCCAGATCCACCAAGCTAAGCCTGCACGCTAAATCGCTTAAAAGCAGCCGACGCACAAAGCTCTGGCCAGGCAAAGCCCACCTGTACGACCTGACGCGATTCAGGTTGAGTTGCATTAGGCACATGGGCATGGGAACCCAGACTTCGAAATGGCCGTTGAACATTCTCTCGAAATTCTACCGTACCGACGGGGAACACATTACTCACTGACGTCGTGGAGTGGAGTGAGGAGACTTTTTTGGCGGGAGCCGAGCTGGCTGCTCGAGGCGAGTACTATGTATACAACGTGGCAATGACGACTAACGGGCAAGGTACGGTACAGTAGTATTCCCAGCGTACTATGTACGTTTTCCTGTGTAGGTACACTCCCTGGGAAAAGGGTTGCCCAGACTGGCAGACAGGTATTGCGACAGGGCGGGACATTGCCTAGGTAGGTAGGTAGGTACGTAGCCAAGGAGCCAGCCACATTATATATGTACAGTAACTTTCGGGGAAATCCAAGGTGCAAAAATACCGATATACTCGGGCGGCAACAACAATCGTTATAGATTACCTGCTCCTTTTCCTTTCACAAGCCCCTGTCTTGTCCTGCTCTCGGTTTTTTAAGCTACCATGGCTTGAGTCAAGACATCACAAGGCAGGCGGGCGATGATCGGCCAAAAAAGCAAAGCACCGGCCGAGAACACACACACAAACGCAACACAGACACACACCAGAAAAGGCGGAAAACCTAACAACCGTGATGTAGTATCAACTGTAACCCCGTGGGGCGCTGATCCCGTTGGCGTCTTGGTAGGGTGTTCCGCCGAGCAGCGGAAAGCATAGCACAAGACCAAACCCGGGAATGCCGGCTTTTGCAAGCACGTTTGGCCGTTGTTTCTGTTTGCTTTACTCTCTTCACACACGGCCCGCCAACCAGTAAGCATCTGTTCCGCCTCCAAGAGCTGCATCCAAACCTGGATTTGTATGTTCATTTCGCGCTCCAGCCACACAACGCGGGGGGGGGGGGGGGGGGTGCAAGGCAAGCAGACATATGCACATTCTTTTTGTGTGTGGATTAAAGCCTCTGGTTTTTTTTTGTCCTTTCTCTCCGTCATCTGCCCTTTCTTTTTTTTTATTTTCCTATTCTCTCCCCGTGTAACTTGTATAGT

**> MD116_chr01:6381854-6385854**

TCTTACTCAGGTTTGCCCTTGTCAATATCTATCACTTACTCACAGACTGTCATGTCCGTGCTCCCAATATCGATGATGCTTTCAATTGTCAATCCATCGCGTGATATTTGTTCAGGTCTGTTGGAGATAGAGGATTTTGAGTCATTTATGAATCTTAGCGACGAACCCAAATGTCATACTTTGTAATTATGTAACTGCCAGATTGGTCAATTTTTGTTTTGTTTATTCATTTCGTATACCGTACAAATGCCACGGCCTGGACAACTTGTTCAGCGAATTAATTAGGTCTCGATCTAGTTCTAGTTCTATCTAGACCTAGACTACCGTAGACCAGCCGCAGACAAGACCGGTCGATATCCGTCTGTCTAGGGCGAATTACACAACCAACGAACTCCACAATAAAAATCCGTCGGAACTAGTAAGATGTAAACAAACGTCAAGGCCTGTAGATACCAATCTGGCTATGATTTTCGGATAATTGCGTAACATCATGACCGCCGTCGGAAAGCCAGGAGCGATCCAAAAGCTCGTCAAAGAAGGTCCTGCAATGGGGTGTTCTCAACACTGCTGGGAGCCAACCCAAACAGCGACGTTGGCGTCATGATAACGCCGTCCGGCAACCCAATCCTTGCCGTACATGTCCGGGAACGAGCAGTCTTGAACTACGATCTCGGCAGCGTTGCCGATCCAGCCAAACGAGTTGACCGTGTAGCGGTCATTCCTCTGTCGTAAGCGAAACCAAACATGTCAGTGTGGGAAGAAACTAGATAAAGCTCTCGTGTGCAGGTTTAGGGGTGGAGGGTAGAAACTCACATAGCTACACCATCCGATACCACCACCGCTGCCGCAGCTGACACGGCCACAGGTGCCGCCGTCAATGATGGGCTCGCCGTTGACCTTTCGGAGGTACTCAATGCCTCTGCTGATGGACTCGGCCGATGCCGGGCGGTAGTCGCCGTTGCACTCGTTGTGGTCACGCTTGCTCAGCGTGCTGGTCTGGTGCTGCTGCTCCCTCGCGCGCCTCGCTGCCGTCTTCTCAGCCATGTCCTTTTCAAAGTCCGGGTTGAGCTGCAGCATTTTCGCGTAGACCGACTCGACGGTGCCGTTTAGCTGGATCTTCTCGCCCGGCCGGATCTCAACCATCCACTGCGTGTCTACCAAGGTGAAGCCGGGTGGTGGGAAAGTCTGCTGGTGGAAATATGATTTGGGCGGGTAGCTGTGAGTTAAATTTTGTCTATTTCACAGTCCAGGGGTGTCTTTCTTCCCTTACCATGGAATCGTTCATGTGAACGCGAACAGTCGACCCGGCAGTGGCAGCAGGATGGATGGCAGCGGCCTCGACGGCGACCATAGCCAGAGAAGCAAAGGCCAAGAACTTGGTCACGGCAGTCATGATTTGGCTAGTATCTATGCCCTGTATTGATGTGGGTGCAGTGGTAAAGAGATCCTGAGTTGATAATAATAGTAGTAGTAAGGATAGGGTTGGGCAAGAGAACTAAACTGGGAGAGAAACGGAAGAAGCAGAACCAAATGCTCGATTCTAGATATTTGTATCTTTCCCCGACTAAAAGAAACATGCACGACCAACAACCTCAAGCAGTCTGGCCAACGGTGACTGTGGGGTCCTAGGCTGTGATTCTCTTTGAACTCAAGGTAACGCACTTTCCGCTTGGTCTCGTTGGCTTTCATCTGATGCCTTTTGGGCACCAGAAGCCCATGCGGGAGGTTAGCAACTTATCCTTATTGCGTGGGCAAGGTATTTTGGTCACTTATCCGGTCACTTGGTTTAGGTATGTGCTTCATGCGGTAGGATAGACGTTAAGGTTCTCTTTTCTACCAGACTAGCAGTTAGTTTCAATACTTGAACACACATTT

GCACCTCATTGCCAAGAAATGGACGAAATCAATGTAGTTTCTCATGTATCCCCTTGTCCCCTCCTGATTTCCGTTTTGGAAGCCATCGATAAAAACCACAAAAAAAGCATCGTCCAGGTTATCATTATATATATATATATATATCTAAGAAACAGTCATAGAAGTGAATGAATAAAACCAGTGCACAAGTATGTAGAAAAACAAGGAAGTAGAGTTGCTCAATAAGTAAGAGCAATGCGAAACCAATCTACCCATCAACGCCCGCAAACCTGAAGCACGCATCCTCAAATGGTCTCCCAT

CCACCGTTGTTGACATCGCTCTCCAGCCTCCTCGCGATCAGGCTTGGAATGAGGTGGGACAGCCACAGGTTTCTCAACATTGTGTCGTACCGCTTTTGATGAAGGAAAAGCTGCCGCCCATGCCTGAAACCCATGTCTGCCTCGCCGTACTTGTCAATGAAAGGCGCCTGCATCCACGAGCCCACGATCCCATGAAGCACGAAGACGGAACACTTGCGGATGTTGATGAAAAGCCCAACGTTTCCTTGACACCTGATTTGTGGAGAAATCTTTTGTTAGCCATGCTACTTGAAGGTCAAACCAATGGAACGAACTGCTGGTTAGAATGGATAAGGCGACATACTTCATCCTGTGCTGTTGACAACCCCCGATCTTCCGCTTGACACTCTTGCCTCGCCCCGGCTGGTCCTGTCGGACAGTCTGGAGGCAGCAGATGGACTGCGAGCAAAACACCTCACCGCAGTGGAGACACAAGGCAGGATCCGAGACCTCCCTGCCCGTCGTTGGACATTTGCACCTTGTAACCTCGTCGATCAGCGAGTCGTAATTCATCGGCAGGCCTACGAGCTCAAAGATACCCGGGTGGCTGACAAGGGCAGTGGTGTTGTCGTTGCTGATTCCGTCCTTGCCGGGCTTTCCGTCCCCGTATATGACATCATGCTGAATCCAGCCTCGCATGACCTGGAACGTTCGGTCATCTTGCGATATCGAGGCACACATGTCGTCAAACGATGGAAGTCTCAGAGCTTCCGTAAGCCGCTCAATCTCCTCGAGCTCCGGCGAAGGAGATACATGGTTCACCAAGTCGACACCAAACCTGACGTGAAGCAGCACCGCACACTTGCGGAGGAAGACGAGAGCATACCGCCTGACGAATTGATAATGCATCGCAAGACCTCCCTCGTTAAACAAAGGTTGCTCAAACCCACGATTTTCACCCAGGCTGCTGTCAGATTTGTCCAGTATGGAGACGTCAGGAATGGAGCTGGCGCCACCGCTCGTCCTAGTGCGGATCAACATGTTCTGCTCCATGACGCCCAGGCAAAAAGACGCGAAGCAGCGAAGAGGGTCCCCTTCCTGCGTGTGCTTAGCCAACAGTTGCGGCAACCACGTCTGCGTCGGCATGTTCCGACCCATATGATAGACCACTTTGGTAATCTCAGCCAGGTAGCACAGCCTTACCAAGTTGGCTATCTCTGCCCCTCTGACGCGCATGATGCCAAATACGGCCTCACAGAGGAATACAAACGGGTCTAGTCGAAGAAGCGGTTTGTAGTCCTGCATCGGAGAGCGCGAGTTCTCCGTCTCTGCGTTTGCATACGACTCGATAAAGAGCTGGCAGTGTTGCCGCTCACAGTCGCGACGAAGCTCTCTGGCGATTCGGTTGTCGCCGCCATCCTTCTGTCCGCCAATGGAAATGTACGAGTCCACAGTCTCGGCGAAGACACGCAGATGCGTCAGGACCTGCTCCGGAATCTTTCCCAAGAATGACACGCCATACTCGGCATCCGTTCCACGCTGTTGAATCTCTGCCGCAGAGATCGAGAACCCTACTGCCCTCGCAAGGGTGTCAGTGTGACTGAGCTCGCCGCCTTTGCCCTCAAAATTATCGATTGGATGATGCGACGGTATGCGGTTGATTTTGAGTGTGCTAGTGAGCCGGCGGTAAACTTCCACCAGCTCCTTCATGGGGTTTCCGCTTTCGGACACCGGGTTCGGTCGCCGCGGGGTCTCAACCACGACTGTCGGACGGCCGAACATGGAACCATATCGAGCAGAAGTAGACATGCGCGGGTTGGGTACCAAAGGCCCTTCGGGGTATTCC

**> MD117_chr01:6499456-6503456**

AACGGAAAAACTCGCCACGGTGCCACCGAGTATAAAATGTCAAAAAGGAAATACGCATAAACATCCGTCACTTCCTCCCCTTTGCCCAAACCTCAACCTTGCCAATCAAGCTCTTCAATCCGACGCCCCTCTCGCAGTTGCCCTCCAGCCAGGTCTCCATTCGCTCTCTGACGTTCTTCATGCCCCTGAGAACGGGCATCTTTGCTCCGTCCTCGGCCTCGATGTGCGCCCCCGGGTTGAGCAGCAGGTCGAAGCAGTCGCTCCAGATGTCGGTCTGCCAGTAGCGCTTGAGGCTCTCGCGGACCTTGTAGCGCCTGCCGCCGGTCCCGGGCATGTTGGCGTCGCAGCGGACCGTCTCGATGTACTTTCCAAACAGGAGGCAGTGGATGGTTCCGGCCAGCCCGTGACAGTCGATCTGCCAGGTCCAGGGCCTGCCCTCGCGCATCTCGGCGCAGTCCTGGGGTGACGTCTTCCAGTCGGCGACGAACTGTACGTCTGGCCGGAATGCGCGCATGTCGATCCCGCGGCCAAAGTCAATGAGGGTGACACCGCGCGACGACCAGCCGCCGCTGCCGTCGGCGCGCCACTGCGAGCTGAGTTGGGCGCCCGCGCTGGGGTCCGAGGCGGTCGAGGGCGCGTCGAGACGGAGCAGGCAGTTGTCGGCCTTGAGGTCGCCGTGCAGCAGGCCCTTGGAGTGCAGGGCCTCGGCGGTGCGGAGGAGCTCGATGCTGAAGAACATGGCCAGCGTTTCGTCCATGACGCCCGAGGGCTCGGCGCGGAAGAAGTTGACCACGTCGAGGAGCGTGCCGTGCGGGTGGAAGGGGAGCATGAGGAAACCCTCGTCCTGGTACAAGTGGAACTCGAGCGCTGCCGAGAGCGAGGCCACGGCGCGGTGCTGCGGGCCCAGGCGGTCGTGGGCAAGACGCATCATGTGGAATTCCCAGGCCGAAGGGGGAGTTTCCATCTTTAGCGCCTCGAGGTGGCTTCGTTGGCTGTGCGCTGTTGCAAAGGCACCCTTGCCCATCTCGATTGCGTTTTCATCCTGAGGGTCTGCAGCGTCGGGGGCTGAGTTCTCTACCAGGAAGACAGGGGCAAAGGCGCCCTTCCCGAGCTCCTTTTTGACTGTATACTTCATTTCGCAGCTGGGGAACTCGATAGTTGGCTGTAGTGGTAGGTTGCTTGTCTTGTCTCCGCTTGCTTTGGCTTTGGTGAGAGCCTTGGCGAACTTGCGGATCTCGGAGCCCTTTTCATATTTGCCAGGCCTGTGGTCGTAGTATCGTGGTTGTGAGGTGAGCGCCGGGCTGGCTTTGGATAGGATCTCGGCCCTGACAGACTCCTCTACTGGGTTGCATTGTAGATCCGCAATGATGGGCCCTTTGGGTGGTATGCTCTTTGGTTGAAGTGCAGTCCTTCCCACAACCTTGGGCACAACCGGCTGCAACGCCTTCCTCGGCGGTGCAGGCTCATCCTCCACAATGTCTCGTAGAGGACTGCTGGGGGGCTCAGCACTCCCTTCGTCTTCGTCTTCGTCTTCGTCTTCGCTGGTTGGGAAAGTACGCCGACACCTACTAGGCGTCTTTGTAAACGCAGAACGACTTCCGCCGCCCGAGCCCATGTTGATATCAGTCTCTGTCCGCTCTGTGATGGGCGTCATAAACGGCAGACGGTTGTTAGCCATCTCGACAGGATCGCGATATGTGCGGCGCTCTGGGACATAGTCTTCTGGTGGGATGGGGACAAAGGTCGTCCTGGTTCGAGGATCGTCATCATCTATTGGCGTTTCCGGTTCCAGGTTTTCGTCATCTTCCGGATTGAGTTCCGGGCTGGGTTGCTCTGTGAAGTTGGATGCCCTAGTGACATCTTGGTCTTCCGCAC

CGATGTCATCCAGATTAGGTATATGTTTTCGAGCAGTAAAGTCAGACCATTCGCTGACGCTCTTGTCATCGGCTGTGTCATCGGCGTCTATGGCCTCATCTTCAGCGTTATCATCATCATCATCATCTTCTTCTTCTTCTTCTTCTTCTTCTTCTTCTTCTTCTTCATCTTCATCTTCATCTTCATCCCCTGCTTCACTAATCGCTCTCGTGGTGTTTCCACTCT

CTGCTCCGCTGGTATAGTCTCCATCGGTATCGTAGTCTTCGTCTGCACTGCCTTCTTCCTGAGTAGCGGGCTTGATTGGCGCGTTGAATATGTCATAAATATCGTCCGTTGCAGCCCGGGTATGCATGGTCATCGTCGGCTCAGAGGTACCTTTCCTCTTGAGCAGCTTTGGTCGTGATGGCGAGTCCAGCTTCGCCTTGACTGTAGAGTCGTTAGATCTGAGAGTGTGAGCGAAGCAGACATTACTTACTTATTTGTGTTTCATTAACCTCCATTACTTTCTTTTTTCTTGGTTTCCCTTCTTTGGGAATTGGCTTGATGGGTGCACCATTCTCATCCAGGGGTATTGTGTCGCGATGGATGACTAGCTTTTGCGGCGCATTCTCGTCAAGAAGATCAACTTGTGAGATTACGGGTGGAGATTGGTTATTTTCATCCTTTAGGGGCACCTTTCTTTCAACCGGAGAGGGTCTCCTGTCACGATGAATGACAAGCTTCTGTGGAGCGTTCTCGAGGTCAACTTGAGAGGGCACAGGCGAAGGACGGTTTTCGTCCCTCAGAGGCACCATTCTTTCCACAGGGGTCTCGGGCCTTTCATCCAACCATCCAGCCCTCGCCAATAGGACTTCCTCAAAACTCAACTCAGATCCTGGAATCTCAGGGGTGGGGTACAAAACTCGAAGGTCGAACACTATGCGCTGCCTCTTGCCAGCTCGAGGGTCCACGGTCACTTGATGTTCAGATGCATCAATTGGAATATGGGATTTCATCAATAACGACTGAAGTACAAAAATCATAACCGGTCGGATCGCCGCCAAAATTAGTCCTGTATTCATCAATATGTGGACGGCCACTAAGGGGACGAGAGGGCAACGGGTGCTTACCGTATCCCTGAACACAGCCATCTTGGGCCCGCTGGGCTTCCTCGACCCCGTCTTGAGCGTCTCTCCTACCCATGACTTGGGCTCCATCACGTTCTCCTTCTTGCGGTGAGCCAACGAGTCGATGGTATCCCATCCCTGGGGTCCTGCCTCACGCGAACCCAGCGCGGACGCTGGTGCGGCGTCGGCATCTGAGAAGATTGCCAGCTTGGACTTGGCCGGCTTGGACCGACTGCTGCTTGCTGTGGCGGCCTGCCTCTGTGCTGCTTGAGGGTCTGTAGGTGCCGAAGAGGCAAACGGGTCCATCTTGGCGGCAAGTGCGGGCCGCATGGTTGGCAATGCAGGGGAGGAGGGTGCGTCAGCCTCGTCGCCAGCCTGTTGCGCGCGGCGCTGCTCAAACTCTCCCAGCTTGCGCACCAGGCGCTGTTGAGGACGGGCGTCCCGCTCGATGCCAAGCCTGTACACCTCCTCTGCTTGGTTCCAGCGTCCTGCACCCTCTAGCCAGGCAGCATACTCCTCGTAAAACAGGGCGAGTCCCTCACCGATGCCGTGGCGCGACACGTACAGGAAGGTCTCGCGGGGCGCGTCGGAGAAGAATTTGATGTAGTAGAGCCACAGCTTCAAGTAGCGGGGGTCGTTTTTGTACTGTGATGAGCCGATAAAGGCCTTGGTGGCGCGCTCGAGGAGCACATGCAGCTGCGACTGGGCCGTCGCTTGAGCCGAGGGGTATGCGTCTAGCGTCCAACGGACATATCGATCGTAGACATCGAGCGGGTCGTCGAGGTCGGCGGCGTTGGCGAGCTCGGCCTCGTATTCTGCGCGGATGCAGTCGTGGATGTTGTTTGTGTCGTTGGGCGTCGGCGGCTGGCTCTTACCCATCGGCGAGGGCGCAAACAGACCGGCGAGCTTCTTTGCGGAGCGGCCACCGGGGAGGGACTGGATGTTCTCCTTGTGGCCCTCGATGACGTCGAAGTTGATGAGGTCTTCCGAGTCCCCCATAGTGGCGGTGAGGGGGAGGG

**> MD118_chr01:6988217-6992217**

GTATGCTTCAGCAGCTCCCACCCGTGCACTTTTGCCTGTGCGAGTATGACATGCTTCTCGCCGAGGGCCTGACGTTCACAGAGAGACTTAAGAGTCACGGTAGGATTGTCGAGACTCGTGTCGTCAAGGGCGAGAAGCACGCGTGGGACAAGCCGCCGCTGTGGGCGCCCAAGGAGAACGCGGCGGTCGAGTACGAGGCTGCGATTGAGTCGCTGAAGACCTGGGTTGGGTTGGATTAGCTAACTGGATTGTTCTGCGCCATATAGAATGTATATACCTATACATGTATATATCTCATGTCACTCGAGCAGTTTTTTAGATCTGAATGCGGACTTTACTGTTGACGGACTGGAGTGTGGCGATGGGGTTCAATCTCTATGAATTTGTTGCTGTTTTTAAACTTTTATTAAATGCTAATACTTTGCCTTGTTGATCACTTTTTATCCTTCCATCACGGCTATTTTTAGTGTTGTTCGTATTAGCTAAAGTTAAGGTACGCCACGAAACTACCGTGGTATGCATATTCTTTTCGCTTCCTCCCAACTATCGAGCCATGTTATGATTCCTTTCCATTGCCAGCGGAGCAGCCGTCGCTCAACGGGTCGATGCCATGAGCTTTACAGAGCTCGTGTTGGAGATCCAGACGGTCAAGTCTGGTCTGAACTTTCTTCGTCTTTGAAACCGGGCTTCTAGGAAACCCCGCCCCCTTTCCCATGAATGGCTGAACAGGTTGATGAGAAGCCATCCCAGGAGCCCGCTCTTGAGAAAACCCCGCGATGGACTCGACTGTTACACTCGATTTTTTTTTCTTTTTTTTTTTTGACCGAAGTCGGTCTCGGAAATTTCTCAGACGAAAAACAAAGGGGTCCTATAGCGAAGAAAACCCCAAACACGGCAAAAAAAGGCCCGCTTGGCGTGATCGTTGTATTCACGGAAGTCTGTGGCTGTAAAACCTCGAAACTCCCGACTTTTGTAATTCTTTTGTGTTTATTCTCCTCTTCTCTTTTGCTCTATCTGGACCGCAACCATGAGAGGAAGACCTGTATGCGAGATCCGCTGGGGTTTGATCAGTTTGCGTTCAGAGTAAAAGGGAGGCGGCGCCATGTTCTCCGGGTGACTCTGGGGGGGACGGGGTTGTGCCGGCGCCTGTTGTGGACGCGTGTTGGGTATCCAGCTCGAAAACCCGGAATCCAGCGGGTCGCAACAGAAAACAACCAGTCTATCGAGGTTTTCTCTTCAGTAGGAGGTCGACTTTGTTTTTGTCTGATTTATATAAAAAGTGACTTTTGGTTGTTTCAACATTCTTCGTCCGAAAAAGGAGGTACTCCTACTAGCGGGAGCAACTAGGTCAGGGTAGGCTTTCGCTCTGCACAGGCGCAGGGCTTGCCCAGCATCATCTGACCGAGCGGTATGGCCATCAAAACGAACAAAGTCAATGATCTCTCGGGTCAAATAAGCCCTTAAACAGGTTCGAATAGCTTTGCTGACTCCCTTCCAGTGGCAGAGGATGGGCCCGGAACAAGTCACGGAAGGTAAGTGATCCTTGTTGTTGCTCGGTCCAGTATCTCGCCAGTCTCGTGGGTACCGCAGCCGCTGTACAAAGGGAGGATGGGACGATCACAGTCTCTAGGAGAATGGTGTATCGCTCTATGTTTTGACCTTTTCTTATTGTATAGCCCATTCTATATACAGGCGGGTGTGACGGTGAGGCAGCATGTTCTCAATATTGTGCTTCGTATAACGAGGGTGAGCCCTGAGACTGCATTTCCAGCCCATCGACTCGAACTCCTTCAGCGAAGCTTTGGTATTATCACCGATATCCGAAGCGATCAGGGGTGAGCCCTGAGACTATGGTTAGTATAGGATCGCGGCGAAAAAAGGTGGTTTGCTCCGCTTACAATGGTTCGCATTTGCGATGGCCATTTCGGCAGTTCCAACACTTGGTTTTGC

CGTTTTTGGCAAGCTCTCCCTCGAAGTCATAACATGCAGGCTCGCTATGAATATATATATATATATATATATATA

ATCCCTCTGACCCTGGGTGCGGACTAGGCACGGAGTACAGATTGGCAGCCACGAAAAAGGCAAAGGCAAGCAGTTGAGCGGGTGGTGTGGTGGTAGTGGTGATGGGGACAACCTTCCCGCACTGCAGTCGCCTGTTGCGCCTCGGCAGTCGAGCAGAGAGCTGAGCAGGTAAAGAACAAGTATGTTTATATAACTGAGTATGCACTCAGATACCCGGTCGATCGCAGGTCTCCTCGATACATCCAAATATATCCGCTGCCAAAGAGCACCCAGGCGTAGGGGCCCAAGCAGAGTTGCGGCCTCGGACCTGGTTCAGTCGCACAAAGTATATGAGGCCCAGCCACGCTCTCATATTCTGCAGTATCTGCGAGGTCCTCGTACTCTCTTTCCCGTTGAGAAGCAGCTCTCTCGAGTTCAAGGCCCAGGGCTCATTTCAGATTGTCTGAGCTCTAGAGTGAGGAATCCATGATCTCGTCTTCCAGCCAACTTTCAAGGTCGCGGCCAAACGCGCCTGAAATGTGCGTGCAGCGGTGCAGCCTATCGCGTCGGACGAGCATATGTGCACATACATGCCCGTACTCACGCAGATATGTTTATGGTGATGATGGGCGGTCGAGTGACTTGAGAGCAATTCCTCGCAAAGTCTAAGGCTTTACCCCAGTGACAGCGGAAGAGAACATAATCCTGGCCGGTGGGCGAATTGAGGCATTGGCTCTCTCTCGTACTGGTCTCCTCTGAATACTAGTAATCAATCTCACACAAAGAGTCTGGTGCGAGACTCAATGCCCTGACAAGAGGTGCCCAGGTCACATCCCCCGCCTGATCGAGTGTCTATGACACTTTGCGCGAGGGAGCCTGTGGTCTGGGCCGCGACGAGGTGATTTGCGGCGCCCGCGATGGCGGAGGAGGCGAGTTGCTCGACATTCAGCTCTGCCACACATTCATGGCTGAACTGCGAGGTGGTGGCTTAGCACGAGATGGGGCCGCATGTGCCAACGATGCTTCTACTGAAAGGAAGCGGCCGCAAGTTGAACGAGAGTCAGAGCCTCCTCGGCAAAAACGAGGACTCGGAGCTTGTACCTCTGGGGTCTCATGGAAGTTGTCTCCAAAATGAGCAAAAGAGCCCCGTGCTTCGTTCTTGTGAGAAGTATATATAAAAGATCGTTCGTGGACTCGGTATTCGTCATCACATCTCCTCGGACTGCTTTGCAAGGCGGTCATCCTCGCCATGGTTCCTCCGCGGGAGGATGAGGAGAGTGTGAGGTACCGGTAACCTTGACCATCTACGTCGCCCATGGCTATGAGGCGGATGAGAGGGCACTCTTGTCAAAACCCACATGGACAGACCGAGCTGGACTCTTATCTTCGTGCCAGCTCCATCAACTCACAACCGCGACATGGGTTAGAAAACCAGTGTAGAGGTACGAATAATTTCCGAATTCAAGCTTGCCCCCCAGCTGATTGGCCTCGACTCGTTCGATCGGTGAGTGCATGCTGGGGTTCACGATGTCCAGCCCCTTTTCGTGCTCTCATACGCAAAAGAAAATGTATGTATACGAGTCCTGTAGCTCGCCTGAAAACAAGCCGCCTTCATCTCCCATGAGAATGAGGGTGCGGCCCTCTCGGTCAAATCAACAGCGAGGAGCTGGTATTCGCGATGGTGACCCCGTGGTTGAGTCCGCCGGGGTTAATGGAGTGTGAATGGTGTACTTTTATCACCACCAGGGACGCCTCTCGCGCACATCTTGATGTCAGATGTTGTTGTGCATGACGTGCTATATATAAATGTGGAAAAAAAGAGCGAGATCTCCGATGACTGTTTCTTCCGCAGAGTGAGGCGCAAAAACACATGCATCCTTCTTTTGAGGCTGCTCGCCACTAGTCGGATTGATAGTACCACGGGGGACTGGTGTTGGTGAACAGACTTCAGGAGTTTGATCCCCTCCCATAGCTAGATACTACTCGTGCGTGACAC

**> MD119_chr01:7603832-7607832**

CGATGACTGACCAATCATAGAAAAGAATGAGCCCTGCACGTCATAGGAAGACCCTGCCGGAATATGAGCTGCCGTCTGGCGCCACTTCGGGCCCGGGGCAGAAAGGCATCCGGCGGTCAATCCGTGCAAGCCGTGGTTCGTATGTAGACTATGGAAACGAGATCTAGGTCTAGTGCTAGATTTAAACTTGATTCAGGTTCATGTCACATCTCATCATTAAAGTGTAGCTACCGCTCTACTATGTAGAAGAATACTGTAATTTTGGCTCCAGTGTTGTGGAATCTGGTCTCCCATACCAAAGCTACCAGCCCAGCTGTGCTCGCTTGATCCGTTGAGGCTTCTTTGCAGCATCGTTCATAATCTTTTCCAGAAGTTTGCTGTAGAATTTAGGTGTTAGCGTGGACCCCCGCAAAGACGTATGTAGTTAGCAAAAAAAAAAAACTTACCTGACTGGCAACCCATTCCGTTGTCCCTCCTCTCGGGCCTGCGCCGCCAACTCCATATCGTGTTTTGTGTGCTCTTTCATATGTTCTAGTACCTTCAACCTCTGTATTGACTCTTTTGCGGACCGCACTACATGCCCGCAGCCCGCTTCTGAGCAAACCCATGTATCCATTGGGCCAGTCGGCTTTTCCGTCTGCAGAGGGTACGAACGGAGCACGACCTTAGTCGCCGTACCGCCAATACCACTATCGGATTCATCACTGGACTCGTCATCGGTGTTTTCCGTGCTCGGGGTTTGTGGTGTTTGCGCCGCTTCGGCTTTGGACATTTTGGCCGGTCTGCCCCGGCGCTTAGGCATTGAACTTGGCCCTACCAGACTTTGCTCGGCAGCAATTTCATCGTCGTCATCGACATCCATTTCTTCCAATGGACGTTTTTTCGCAACAGACAGGCTTAGGCGTGGTGTTTTGCCAGCACCAGCATAAGGCCTATTCGGAACACGGCTAGGCGTGGCAACTTCCTCCACCGAGACGCTTGTCCGATGTTGACGACGCACAAGCCTTTCAGGTATCTCGTCAATGGATTGCAAGTGAACCAGCTTGGTGATGGGCTCCTTGGCTTCCTTTTGCAACCCCTCCCATAATACCGTCCCCTTCCATCGCTCCGGGTCCAAGTTTTTTAGTAGATCGGCTGCGTATAGTTGCAGAATTTCCTTGGGGATGGAATAAGCCGAGTTGGGAAGCTTGCATTTTTTGTACATCTCAGATGTAAATTTGCCATAGGTACTTCTGCAACCTGCATCTGATTCCGCGGCGATTTCTTCCAAAAGAGTAATAATGGTGGGTATGTTGGTCGCCGGGAGATTTGTTGCAAGGCCGCTTTTAACGACCGTGGGAGATATATGCACAGAAGTAGGTGCAGATTTGGGCGCCCGTTTAGTAGACGGAGGTCCAGAAGGTGGAAGTTTACTGGGCTCCGGCTCCGCACCTTTGGAAGATTCGGCGATGGTAGTAGTTCCGGACATTTCGATATCGACCGACAATCTTGTTCGCGAATTAGTGGCCGCCGCCTCAGTAGCATTTTTTATTTTTTGTGCTTCAAGTTTGGCGACAATATCGGGATGTCTTTTATGCATCCAATGGAAAAAGCTTGTCTTGCCCCAATCGAAGTCCGACTGTTGATCGAACTGGTCGAGAAGAAACAGAGCGTGCTTATCGCATCGTTTTGCCACCTCAGAGAACAAGACGCCGTCGCCTGCCTTTGTGGCGTAATGGTAGAAAATCTCGTCTAGTCGAGCATTCAGCAGTGCTTGACGAATGGCCGCTTTGGACTTCCTCTTTTTCTCCTCGACAATCTGGTATGCAGTCATGATTTCCCAGTAAAGGGTAATGGCCTCAGACATGTCGAAATAGGTTTCGCGGTAGACCTTTGCAGGCTTGATCTCCATCCATCCACCAATAGACCATGCCCACAAGCACAACGGCTCGTAACCCACCGAAAATATGC

TGCAGTTGGGTATTTGGACGTAAACGGATTTGCACCTGGGTATGACGACTAAAAAAAAAAAAAAAAAAAAAAAAAAAACCGTCAGCTGGGGACGGACATTGGTTAATTCGGGTTTGCCAGGTACCCACGTTGCTCTTCTGTATCCTCTTCCACAATCATTTTTCCTTTGACGATAAACGGACCGTCCAGTGTCACTTCGAGGAGATTCGCCATAGTCTTGCCATC

CTGTCGGAATACAGTCGCATCCTGTAGGTCAAACATGGGGTCGTCAATATCAGGAGGACTTGGCAACCCCGTCGTTGTTGCAGGGCGAAGGACCTGGGCGATATCATGGTAAACGGGTGGACCTCGCGGGTCTATCGGGGTGCCTGAAGAGCGCGTGCGTCCGGATGGGTTTCCGGGAGGCACGACCCGCGGTCTAGGTCTAGGACCACTACCTCGACCTCCCATGGTTGCAGATGTGAGCCTGGTTGAGGTTGGCGATCGGAATCATTTACGCAGTGCAAGCTAAGGAAAGTTTTTGTCGGAGAAAAAAAAGTGAAGAGGTCAATTTAGTAAGGTGATGGACTTATTGAGTAGTAAGAGTATGTGGTTGAAATCAGCGTAGACTGTCCAGGTGTTGATGTACCAGGTAAGTCAAGTAAGGTCTAAACAATGACGGTCGGATAGGACGCGTGATGTGATCCCTTACCCAACGCGACGGTCGGTGGGGTAAGCAATTAAGATGGTTGGAGAGAGCACATGCAGTTGGCAGTTTCGCGTAACCCAACCCCGCAAAGAAAGCTCGTCCCGCTGCGGACCCCTGCAGCTTGCCGCAATCGCCAGAAAAAAGTTTCAATTCGAACTTTTTTTTTCTTGCATGATGGTTAGCTAACCATTTTCCACTCCTTGCGATACTCTGCCAATTGTGCCCATCTACCACGCTGCCCTGCGCACGTAGCTTAGGTGTAACTTTCCAGGCTTGAATGGTCTGGCTTCCATACTCATATGAAAAGAAGTCTGTGGAAGCTCGCCGATATGGTCATACCATCAGTGACGGGCCTGGATGGCATCTTTGGTAAGCAACACCGAGGAACATCCCGGTACCGCACCTACTAACACCCAATCCCCCTAGCCATCAACAAGCCAATGGGCATGTCCTCGGCTCAGGTCATCCGCGACTGTCAAGCTTACTTTCAGCCCTCTCGACTATTTGCGCCAATGCTCGCGCGCGAAGCAGAGGAGGCCCGTCGCGACGCCGCGACGCAGGGCAAACATAACCGCAAATGGAAGAAGAGGAAGCAGGACCCCCGCGTCAAGATGGGTCACGGTGGTACGCTGGATCCGCTGGCGACGGGCGTGCTGATCCTGGGCGTCGGCCGCGCTACCAAGTCGCTCCAAAACTTTCTAGAGTGCACCAAGACCTACGAGACCATCGTGGTGTTTGGCGCCGCTACCGACACGTACGACCGCGTCGGGCGCATCCTGGCCAAGAAGTCATACGACGAAATCACGAGGGATAAGGTTGAGAAGCAGCTGGATGCGTTCAGGGGCAAGTACAAGCAAATGCCCCCGCTGTACTCGGCTCTCAAGATGAACGGCAAGCCGCTCTACGAGTACGCGCGTGAGGGCAAGCCGATCCCACGCGAGATAGAAACCCGCGAAGTCGAGGTTCTCGGTCTGGAGCTGTTGGAGTGGTATGAGCCGGGTACTCACAACCACCGATGGCCGACCGAGGAGGCTGGCATTGCCGAGAAGAGCCTTGCTGAGAGCGTGTGGAGGGTGGAGCACGAGCAGGCGACTGGCAAGAAGCTGACGCCTGAGCAGGGAGCGGAGGAGGACCGGGCATTTGCCGCCCACCAGTCGTTCAAGGAGCAAGCCGAGGAGCGTCAGGACAAGCTCGTCTTTGAGCGGGGTCAGAAGCGCAAGGCCGAAGAGAGCAATGGCAGGCCAAACAAGAAGCAGCAGGGGAAGCAAGGCCGAGAACAGCTCATGTCAGGAGCACTAGGAGAGCTGCCACCGGGAGGCCCATCTAAGGGACGCGGGTCAGATTTGGTGCCACCAGCTCCTA

**> MD120_chr01:7850311-7854311**

TCTTTGTCATTGGACCATTGTTTTCCTTTTTGCCGTTCAACACCACTTAGACTATATCCTTTAGATAGCATCGTACCTGTGTGAAATATAACATTTCCTGAGTGATTCTGGAGATTGCCAAACTACGGACCTTTCCTTTCTTTGCTTTTGAATATCGATTTTTTTGCGCTTTGGGAAAGTTTGCCCCAGCATCAGGTAACACTAGTTATGAAATGCTTACAAATATTATCTCGAGACTGCCATTTACAGCTGCTCCAAAAACATCTCAAACAATCCTCTGCTATTTTAATGGATTTTACCCCTCCCGCTGTTTGTGTTATCGAGTTCTTTTCTCACAATGGCAAGCAACCTTGAATTGGCGACTATGCGGCTAAGTCTAGGACTCGGACGCAACTTTTCTGACTGGATCTTACCGCTCGCTTTGTAAAGCAAGCAATAGGCCCGGCCGACGTTTTCAATCGTCTCTTCAAAAGGCAGAGTGAAAAACAATGGTGTGGCCACGTCATTTTCTGATTTGTGTATGTTTGCTACAGTTTTCGAACGAACTGCTAGTTTGACGGACCGAAGAGTGTTTCCCATGGCAGTTGGATATTTCTGTCTAAAGAAGGGCTGTATCAGATACCTGGTTTCAAATCAACAAATGAGATCTTTTGTCTGTAAATGAGCAGGCTGAACTATTGAAAATCGAAGGTCGAGAAAAATCAAATAGGAATACTGAAAAGCGTTGTTTTATTAGAGCTTATATGGCAAGGCCGAAATATCCACCTGGCTGACATATTCCCGATTTGGAATCTGAATTGTCCAAAAACCCGTCTGGGGCTTTGGTAAAGTCTGCTGGTTTCTCCGTATTTGTTCCTTCTCTTTTTTCTTCGGAGAAAAAATCAAAAATAAAAGAAAGAATGTATGACCAAGCTTTCTATCTGCCTGCAAAGCCTCGCGATGTGTTAATTTACTCGAGGGAATATCTGCACGACAGTGTTTTTGACCTTTGTGGTATCGTGCTATTGAAAAAGCTTTTCGATAAGGGTTGTAGTGCAGAAAACGTACACTCCAAATTGAACGAAAAACAACATGGTAAGGTATTGGACCCTAGCGTTACGTTTACCCACCCGAAACTAGGCTTTTTCAGCGCTGATCTTTGGTACCTTCCAAAGTTCACATAATGGTCTACTTAACTATCTTTGGCTTCGGCTTACGCTTCAGGGCCAAAATTCTGTCTGAACAGGAATAAATTCCTCAAACTGACCATCGACGGGCGAAAAGTTGGCAGCTTAATCACTTTTATTTTTCCCCTGTTCGGCGTCCGGTTAATTTAACCTACCAATGCCACGGCTTAAGCGGGGCGCTGGTCAAAGCCCAAAACTTGCCTACCAAGAAACTGTTTGACGAAACTTTGTCCCACTTACTTTGTGCTCAATCGCGCTCCAACCGATGATTTAGACAAAACGCTACTGTCAAATTTGTTGATAACAGTTCGGGGAAATACTTCTGTGACGCCTTCGCAGCCACCGGAACACAATAAAACCCATTGTTGCTTGGGTGCTTTGCCGTATGGATACTACGGCGCTGATTCATTGCAAGCCCCTTGATCGTAAAAACGCATAGGGAAGCTGGGTATTTTGTTTTAGACATAAATGTCGCGATAAGACTGCTGGATGCAATGAAGGATGGAAGCTGGAAGCTATTTAGTCGTACTGCTGCCTTTGTAAACTTTGCTTTCTATAGAGAACATGAGAGCACCTTGTCAGGAAAGAAAAACAGTAAGATTACAAATGAGAATAAAAATTGGGAGATCAAACGGGTCCGAATATCCATCTACACCGGGGGATAAAAGTATAAATAATCAATACCCGCATGAATCTCGGCTGGATAATG

TCCAATCTTGACAACTCTTCACCGAATTAAACACACTGTTACGGCCATTTTGATCGCAATACACGTTGCCACCAGTCGCGGTTGTCAGCATAACAGTTATGCGTGTCTTTACATCGCTATTCAGCCGAGAGAGAAACAGTCGAGAGAGAAACAGTCGAGAGAGAAACAGTCGAGAGAGAAAAAGGAAGGCAAAAGACCGAATTTCTGCGCCGTTCAACTTTCAAC

ATCTGAAACATTTAGAAAAGGAAGACGTGCCAGGTTTGGAAAGGGTGAGAAAAGATGTCATAACTCAGGCGCTCCATTGCGCACACCACTGCCATCAGGTGGCTCGTTAATTATGAGCTAGATTCGTCAGCTAAGCAGCCTTTTTATGTGAGCAGTCTTCACACCTCGCGGAAAACATTGAGGGTATGGCAGCGCCTTCAGCAGTTATAACGACCGGGATAGCATCAGTGACGTCCACTGGGTCCCGGGAAGCCGTCGCGACACCCACCCCCTGCCCACCGACGGAGGCAAAGGCAGCTCTACCACCGTTAATGCCATGTTCATCTGATGGGGAGCCTACCACCACTACAGGGGAAAAGTCACGGGACTGTGACATAACGCTACAGGCTCTGAAAGAAGCTGCCCCCCAAAAGGCTTTGCTTTGAGAGAGGATACAACCTCTAGGTTTCCCCTAGCAGGTAAAGCTTTGCTGCGACCTCTCAGTATGGATTCAGAGACGCTTGGACAATTGGATGGTTCACAACCTGTGACTCGCTACCCAGTTGTTCCTCAAGATACCGGGTCACTTGGCAATTTGGACAAGAGCAGCTGGGCGGACGACGTCGACTACATCTATGAACATGGAGCAGAGGCCAATTGCAATTATCCGTGGCACCGCCCATCCTTCGACGAGCCCCTACAAGCCTTGATGGCCTGAGCGAAAGGTGTCTGTCTAAAAGGGTCGCTTTAGATTAAGCTATGATGAGAAAAAGGTCAGCCGTGGACTTTGTGTAATTTAAAGACTTAGCAAGGGCAGGAGCTGTGAAGGTATATTGGCCTCACATACTACATGCTGATGCGGGATTGCTAAACATATTGCAAATAGTTGCTGGATGGCAAGTGCGCACGTCGTGCGCAGAACTGCATAACGCAAAATTTATATTGTTCGAAAGTTATAGTTTTTTTTCGTTTCCAAGATTCAAAACCAAAATTGGAGTAAACAAAACATATCACTGCTAGTCCCGGTGGATGAGGACTACGAGGAGTAAAAATTTTATACCAGAAATACGATTTTCAAACCACGTTACAAAGCAGACGTTTATGAAGTGTACCCAAGGGCCACGATCAAGCTAAAGTTTGGCCTCAGAAAACCTCTGCGAAAACGTGTACAGCACTGCTTATAACTGCCCCAAACAGTGCACAACATGTGCATCCTATCAGAAGCTCGAACACGTCACCATTTTCAACCTCGATCCATGGCGGCAACTCATCGAGCTTGGACCGGACCATAGAAAGTGACAAGGTGGGGATTATGTACGGAACGCAAAGCAGTAGTCCAATGAGCAGCCTCAATATAAAGACAATGGGCTTCGGTGAGAATAAAATCTCCTTCGACTTTTTAAGCCAGGTAAAACGGCCATAAGCCGACATGACGACAAGGAGAGTAGCGATACCAGTTAGCACTACGCCGGCTATGAGGGTATGGCGGAAATATGGCGTTGTGATTTGCGTAAGAGTAGCGTCGACATTTTCAATGTCTTGAAAGTGCTGTTTCAACACTTTCACAATTTCCGGCTCGATGAAAGTAGAAAGGTCAAGAGGCAGGGCGTCACATGAGTTTTTGTCACCGATTTGAATACAGAACTCTCCGGTCCCCAGGGAGCAGTTTTTGGGAACTAGTTCCGAAATAAGCTGGGGCGCTTGGCTGATGACTTTGGGTATATTCTTGATAATTTCTGGGACCGATGTAGCAACTGCTCCTGCTGCACTCTGGAGATTGTTCAAGATGCTGGAAACACGAGATCCGGGTCTTATGCCGTCTAAAAGACCTTCTTGCTGCGGGAAACGCCGTTCCAGCGCATCGAGTTTTAAGATTTGTGTGACATCAAACTTTTGAATTTAGCAGCTTAAAAAGCCAGGG

**Chr.2**

**> MD201_chr02:218876-222876**

AAAAAGCCAGACCGGATACCAGAATGGCAACGCGATCAATTCGTGTCAGGTAGCGTATCATCTGTGCGGGAAGCTGAGTTGAGTACCAAGCTTAGCATTGTCTGTTGCATCAAAGCGTGGAGGACAGCTTGTCGTAGTTCCAAAGAGAAAGAAACAAAAAAAGAGGTGGTTCTGCCCAGCGAAGCCGATGGTGCTTTCTTTTTCCTTGTCTTGTCTCGGCATGTCATCGGAGAAAAGGCACATCTCCTGAAGGACCTGCATCAAAAAAGCCAAAAAAAACGCGTTTGCCTTGCGGCATTCTACGGGTGACGTGCAACCAGTCAAGGGGAATTTTTGTTTTCTGGATTGCCAAAGAGTAGGCAGCTCGGACTAGAGTAGAAATGCAGGTACCCTTGTCGCGTCAATCATAAGCACTTAGTGTAGACACCAAAAGGCAACTAACTCATCTGTGTACATACGTACGTATGGCATGCAAAGAGGGGCCGTTTAATCAATGTCAAACTGCCTTCAGCCGGAGCGGGCCGTGTCAATCGCAATCTGGATCTGGATCTGGATCGGACGTTGATGTGCACACGTGCCAGACACAAAGGTCTCGGACATGTCGGGATTAGAGTTAGGGCTGTCTTTGCAAGGCACGTTTTTTTTTCGCATGTCTTGGATTTGTAGATGGAACTTCGTGGTAATTAGGTATGGGTGAAATGTTGACTTGGGGGTATAGACTCAAAATACGTAATTGCTGCATGTCTTGGCAGCCCTGGAGTACTTGCTCCGTAAAAAAGTCGCGTCGAGAGTAAAAAAGGCAAGCTAATGCAGGGGACGAGGCCAAGGGAGCACTCGGCTAGTCCTAAAATATCCTTCAGCTGGAATGTCAGTGCATCCATCAGCTGCCGAACCATGGATGCAAAATGGATGGTGTGCTCATCTCAAGGTGCAGCTCATGTGATGCGGGGGGATGCGAGAATGGCATGATGACGGGATGAATCTTGGCTGCCTTTTGCAGAAGATCGTCCAATGATTTGCTTTTTTTATACTGGGGTATTCTGTGTTTTTTTTTTTTTTTTTTTTTTTCGTTCTCGTCGGATTTTGGACACAACCCACAACAAAACGGACAGAAAAAAAGCCGTGTAAAAGACTAAAACTATCAACAGCAAGAGTGGCCCAGTGTTTATCAGATATGAAATAGTCGCAAACCCAAAGCCCGACCAAGGAATGAGTAGAATTTACCTAGATCAACGCTAGACGACAAGATGGACGGTGCCTAAACTTGGTAGTCTAGCTGCCACCAACAAGCGAACTATTACCCCTGGTGCGCAGAACGGGAGATTGCAGACGTCGTAATTGGTCAATCGCTCGATCGCCTAATTTTTTGTGTTCGTTCTGCACACTGTGGTCTTTCAGCCGTGGGCTTTTTTGCCATTGCTTGTTGGCCGCCGCCTCTGTAGCATCCTACTCCATGAGTATGGTCTGCCCTGAATTGCTGCTCGACCCTTTGAGGACCGAGACAACCGAGACAAGGAAGGCTTGCTAGGCACGCCTGAACAAAAGAGAAGAAAATAAAAAGAAAAAAAATAAAATAAAACCAAGCAGGGCAAGCTTGGTTATCGTTTATTTTGCAAGCCTTGTTCTTGGCGCCAGTGTTCCGGGTTCCCGAAAGTGCACAGCAGCTGAACTGAACCAAAAACGTTTTTGTTTGCTCTTTTCGCTAAGCGACTCGGTGTGTCTCTCCCAAGCCCGACGGGGGAGGAGAAAAAAATATGGTTGTGTTTTTCGCTCCCTTCCTCCTGCCCCCAGATCCTTCCGAGTCGTGACTCGAATTTTTTTTTTTCTATTTTATTTCATCAACCCTCCTTCCCTTTCTTTTTTTGGTCCTCCACA

ACCCACCCCCGCATCGCCCACCGCGTCATTTGGGATCTTCGGTAAGACGTCCGCAGAGGAGGCGCAGTCTTCCGGTCCCGTCCATTCGTTCTCTTTTTGTACTTAATTTTGTCCTTTCCGACTCGCTCTCTCTCTCTCTCTCTCTCTCTCTCTCTCAATAATGCATAGTCACTCATTCAGTGTCTTGTCGGCCCTGCTGCTGGCCTCCACAGCCGCCGCCTCGGG

CAGCTACGGTCAACCCGGCAAGGTCGAGGTTCCAGTCGTGACGGTGGTTGTAAAGAGCACAATCACGACATGCACGCTGCCCAAGACACCTCCTCCGCCATGCTCCAGCAAAGGTAGCTCGCAGGGCGGGTCTAATAGTGTTGCCACACCGTCTGGAGGCAGCAACGGCAGCCCGTACGGCGGTTCCAGCGCGGCGGCCACATTGACGTCAAAGTCAACTGGACCCTCCAGCATCCCGACCTACTCGGTCCCTGGCAGCAACTCAACCATCAGGACGAGCCCTGGTTATGGTAACGGGACCACCAGTAGTACCAGGACAGCGACTGGCACATCTACCTCGGCGTCGGCCACGTCGAGCTCGACCAGCTCGCCACTGACGGGCTGCGGCAGTGACTCGTACTGGCTGGCCAATATCAAGCACCAAGGTTTTGCCCCTTATGCGCCGAACCCGTCAAACTACACCGTGTTCCGCAGCGTCAAGGAATTTGGCGCCAAGGGTGATGGCAAGGCAGACGACACAGTAGCCATCAACCGCGCCATCAGCGAGGGAGGTCGCTGCGGTCCGGGATCGTGCAACTCCACCACCACCACTCCTGGCCTGATCTACTTCCCCCCTGGTACCTACCGCATCACGTCTCCGATCATCAACTACTACTTCACCCAGATTTATGGCAGCCCGCTCTGCATGCCCGTCATCAAGCCGAGCTCCAACTTTACCGGCGCATGGGTCCTCGACGCCAACCAGTACCAGGCCGGCGGCAAGCTGGGATGGGGCTCTACCAACGTCTTTTGGAGGCAGGTTCGCAATCTGGTCATTGACATGACGGATCTGCCTGTGGCTGCCGATGTCGCCGGAATCCATTGGCCGTCCTCGCAGGCTACCAGCCTGCAGAACATCCATGTCAAGATGGCCAAGGGCACGCAGACCAAGCACTTTGGAATGTTTATCGAGGAGGGATCTGGAGGCTACATTGGCGACATGGTGTTTGAGGGTGGCCTGGATGGTATCCGGGTCGGAAACCAGCAGTTCACCATGCGCAACCTGACTTTTATTGGCGCCCAGACTGCCATCAAGCAGCTGTGGAGCTGGGGATGGACCTACTCGGGCATCACCATCAAGGATTGCAAGGTCGGACTGGACATTTCCAACGTTGATACCAGCACTGGCCGCAACCCCGTGGCATCAATCGTGTTTATCGACTCGGAAATCAGCAACACCCCGATTGGAGTCGTGACCAGCCGCAAGCCCCCGGGACAGCAAAACAATCCCGCACAGGCAAACACGCTCATCCTCGAGAACATCCGCCTGTTCAACGTTCCCACTGCAATCAGCGGCCCCAGCAACAGTGTCCTCCTTGCCGGCTCAGCATCGTTCACAACAATCACCGGATGGGGCCAGGGTAACCGCTACACTGCTTCTGGCGGCCCGACTGCTTTCCAGGGTCCAATCACTCCTTTTGCCCGTCCTGCAGCCCTGACTGCGGGAACCAACTTTTATGAGCGCTCGCGGCCTTCATACGCCGAGGTGCCTGCCTCGCAGTTCTTGTCGGTCAAGTCCGAGGGTGCCAAGGGAGACGGCAAGACTGACGACACGGCAGCCATCGCCGCCGCTGTCAAGAAGGCCAGCTCCTCCAACAAGGTCCTCTTCTTTGACTACGGCATGTATGTCGTCAGGTCAACCATCTACATCCCCGCCGGCATGCGCATCACCGGCGAGGCCCTCCCTGTCATCATCTCCGAGGGTGAGTTCTTCAACTCGATGGCTTCACCCAAGCCTGTTGTGCAGGTCGGCAAGCCAGGTGAGACGGGAGCGGCGGTCGAGTGGTCCGACATGGTGCTGTCTACGCGCGGACAGCAGATGGGCGCCATCCTGATCCAGTGGAACCTCGCATCCACCACC

**> MD202_chr02:387841-391841**

GGCAAGTTTGGGGCTAGCAAATGAATCCAGCAACGACCGTGGCGGAGAAAACAAGGTGAAGGGGACGGGTCCATGCGCTTGCGAAGAAGACGAGGAGGAGGAGGAGGAGGAAGAAGAAGAAGAAGAAGAGGAAAAATATGAAAGGAGGCTGTAAGGCATCGCCACGGGGGCTTGATGACATTCTTATCAGCCCGAGGTTGAAAGAGAGTATAATAGCAAGTGGGGCGCTCTTATTTTGCTCAACTAGCCACCTCCCAGCGTTTGAGGAACACGTACGTTCATGGGCACGTGGGACTATGATGGGTCAAGAGGAGAAACAGAAATGCATGCATGCATAATGTGGTGGGTTTTGCTCACATGACTGAGGATATAATATATCTGAGGTCAACCATCTCTGGTAGCAACAGCTGCCGGTTTGACAGGACTGTCATCCTAGTCATGTCAGTATACGATACGCTATGATCTGCAATAAAGTGGTTCCAGACCTCGTACAGATCACGACATGCCGACCGATCGAGAGCACAACCTGGATGATGATGATGACAAGGTTGGTGATCATAGCACCCAAATACGTCAAAGAAGGCCCGAGGTAGCATTGACCACAAGTATTACCACGTATCACCACGTATGATGCGGTTATCTACACCTCGCCCCGAACCGTCGGGAAGCTTGCCTTGCAGCGATCGAGTAGGTTTGGGAGCAATTTGTGTTGTTTGTCTGCTGCAGCGGCGGCCACCGAAGCACCCAAAAGCATGGTTCGGGATGCGGAAGCGTACGCGGCCGGACCCGTGGTTCATGTCTGGGGCGATGCCCCACTCCTGATAAACTGTGCAATGGTGGATTGCTTTGCCTGCCTGGGATGAGGCCAGTCTTGAAGAACAAGGAAGTAACAAGAGAGGTCGATGGAATGTAGGCATCGGCTTCGCGTGGGAACACGATTGCGTTGGGTTTTTGCTTGCATCCGACACGCACAGACTAGGAATTCTGGCCTCCTCGGACTGGGTAGATCAGATCAGTTCGACTGGTGCGGTGAGGTAGATGAAGAAAGAAGAAAAGATGGGATGGGTTTTGGTGGCCATGATTCGGGCCAGGCGAAAACGAATTAACTTGCCGTTTTTGGCCACGAGGAGAAGCTCGGCGTTCACGAAACGCTTGGGTTGAGAGGAGAGGATGCTATTGCGTATGTGACTCGATGCTCTCTTTTTTTATCGATTGCAGGGTGGCTTGACAAGAAAAAGTGAAAAAAAAAAAATCATCACTTGATACCTACCAGTAAATTAATTACTCCCATCACAACTAGTATTTCCTCCCACCCAATCTCTGAAAATAGCATCTGTCAAAGCCATGAGCACAGCACCACCACCCTTTTATGTGGCAGGTTCATTTGAAGAAAAGAAAGGAATTGCAAAAGACGAGAAGAAGAGAGAAAAAAAGGTACATCAGCACAGCCAAAAGTTGGAAACTCTTTTTCCAAAACATTTCTGCCGCACTTTTGCTGTTGGTCCAACTGGGACAGGTCCTAAAACCAAACCAACATCGGACGGTCGTCCTACAGCCACCATTCAGTACGTACCAGTGTTGGTCCATCCAGGGTCGCGACACGCCACTCAACGCGCAGACACATCCCTGGCAGCCGATCCGAGCATATCATATCCCCGATCGTTTATCAGCCAAATTTTGACTCCAAGTGATTATCGCCCAGGATCCACGACGTACCTTGGATTGCACGCCTGGGAAAGAAACAAAAGGTCGCCAGGTCACGTAGCCCAAAATCGTCCCAAAAAAAAGTTGCAAACACCCAGGTAGTACTCCTTTTGCAGCCTTGCTTGGGATTGTCCGGCAGTCGGAGCCCCGCCGTCCAAGCTTGCGACTCCAAAACAGTTTTGCGACTATTTGGGGAACCTGCACAAAAGGCGACACTTGAACAAGCTGGGACCTAAGCACTTGGCT

TGCTACGATAGCAGTTGGGTAGGTCAATATACTTTGGGACGACTGTGACAGGGGGGGGGGGGGGCCAGAGGGCAT

GCCCGTCCATCCCGTCTCTTCTCTGTGCTTGGCTTCAGGGCCTCGATACAAGGTGCCGTCATTCGCCGACTTGGGGGCCTCAATAGGCTTTTTTCCGAGGCTTGTTTCTTTCTTCTTGTCCCGAGACTGGCATCTCTTTTTTTTTGTGTTTCTTATTTTTTTCTCTAATTACTTCTTTTGGTGTTTTCCTTTGAACCTTTTTAGGCCATCATACCCTTTACCCTGGATGTTTGACAATGACCCCTGGTGCTCAGTAAAGGACTTGGTCAAAAAGGACTTGGGGTCACTACAACCAGTACCAAATGAGAAGAAAAAGATCAAGCACAAATGCTCTTTCGCACGCCGTGCTTGTGGCCGCCACCGGCCTGCTAGTCCTGACTCTTCCGACGGGGGTTTTGAGCAGGAGGCTCTTTGAGGGGAACGGTGTCGCCAAGATCCCAGCTGACTCCGGGGAGCTGACGGAACCTGCGCTCTTGGATGCGGCGGCGGCAGCGCTCATCACCCCGGCACCAGTGGCAGTGATAGCAAAGGTGCGACGGGACGATGTCGACTCCCTGGACGTCGACTACGGTCAGGCAGCATTTGGGCCGGGGCCTGGGCCTGGGCCTGGCCCTCCGCGACCACGACCACCATCACAGCAGCAGCCGCAACAACCGCCGCCGCCTCTACGGGGCCCGGAGGACCAGATAGTCATGTCGCTGTCCAGCGCGCTGCTGGCCGCCCAGAGGAACCTCAGCCAGGCGTCCCAGACGTTTGAGCAGTCCATCAGCGAGCTTCAGGGGTCCTTGTCCTCGGCGACGTCGGCCGCCCAGGCTGCCAGTTCCAACGCGGCGGAAGCGTCCACCTCGGCGGCCTCTGTTGCTGCGTCCGCGTCCACCGCTGTGGCTGTCGCGAGGGCGTCCATCAGCAACATTCAGGCGGCCGCATCCCAGTCCCTGGCGGATGCCGAGTCGAGGGCCAAGTTGGCTCAGGCCAGCGCCAATAGTGCCGAGGCGGATGCCAGGCAGGCGAGGGTAAGTCAAACCTTGGTTGTTTTCTTTTCTCTTACTTTTCTAGTCTGTCTCGGCAACACGAAATGAAAAACATGCACACAATGCCAAATACTCACATTCCAAACCCCCCTAGGCCGAGGCGGATAGCCGTGTATCGCAAGCCCAGGGCGCCTCCGTGTCCATCACGCAGGCCGCCATCGCCGTGGTCGCGTCCGTGCTGGGCTCGGCTCTCATAACGCTGATAGTGCTCTGGCTTCTCATGCGGTACCGGCGCAAAAAGCGGTTGGAGAAGGTCGCCGAGGAGGAGGAAAAGGAGCAGATAGCGCTGGCGGCGGAGAAGCAGCGCCTGGCGGCCGAGGAGGCCAGGAGACGCGGACTGGAGCGCGGGGCGGCGGCCATGCTGATGCACTCCCGGCAACGGAACACGGGCTCGTTTCAGTCTCGGAATAGGCCCAGCAGCACCGGCGCAGGGGGGCCGAGCCGCAACTCGGTTGCATCGCGCAGGGGCAGCGTGGTCGGGCAGCGTGGCCCGGGGTCTGGGAATGGCAAGGGCGGACCCTCGACCAGGCCCCAATCGGCGTCGACCATGGCCAGCAGCAGCAGCCGCACTTACGTGAGCGACCATCCCATCAGCTTTCCGAGGGGGGCCGTATTGTTATTTCCGCCGGGCTCTCGGAACAGAGATTCGTTCAGCAGCGAGCAGGGCATGGTTGGCTATGCGATGAGCACCAAGAGCGATCACACGTCGTCGAGGCCTGTCACGGCTGACATGTCGGCCGCGCAGGGCGCCGGACGTTTCAACCTCGCCTTGGGGTCGCACCCCGTGGGCGGTGCGCCATCGTCCAACTCGGATATGAACCGGACATCGGTCTACAGCCCAAACCTGGGTTCAGACCGGAGCCGTCCTACGTCGAGAGCGCCATTCTTCCAGCAGCGACTCGGAAGGTTGGATGAGGAAGACCTGCCTAGAACCAGTCTGGACC

**> MD203_chr02:1160885-1164885**

GCCATTTCCGCAGCGGAGAACCCTTGAGGAAATGTCTCTTTTTGTGCCTAGTCTGCTAGCTTCATGCGCGTGTTTGCGCCTCCACTGGTTACGCTTGAACTTGAACAGGGTTTCGGCATCCAGAATAAACGGCACGATTGACAGTGGGCTATCTTCTTTTGCGTAGTGCTAGGCTCTAGCTGCTTGCTTCTTTTATTCGACAAGGTTTCCGTCTGTCCCCCGGCAGGGGAAAGCGGATTCTTTGCTTGTGGCATGTGATGTTTGGATAAAATTGCCCGTCCAGGGCAAACGCTTGGCTTGCGCCTCTCTCGTCACAGCTCTGGGCTCAGTGGAAGGGGGATGGGACATTTCTTTGCCCATCCCATCCCATCCACTTTTATTTTATTATTATTTTATCTTTTTGGTTCTATATCTTTGTCTTTTATCTATTTTATCGTTTTATTTTTTATTTACTTTCGTTACCAGTCAGTCTAGCCGGCCGCCCGCACCTTAAGATGAATGTCACTCAAGGGGACAACTCTAGCAAGTCAGCTGAGAGTACCATCCCCTTGTCATGACATCCCTCTTTGGGAAGTCTACTGTCCAAGACCTGTTGCATAAAGAGCTGCCATCTCATCCCACTTCCCTTCCCTCGTTCTCTTGTCTGTCTACATTCTCAAACCACGCTTCTCATTCTTGCTCGCCTACCTTCTTATCTCACAGGTCATCATCAGTCTGGACATCAAATACCACCACTCGTCTTGACAACCCCGCCAATATATACGGGAGTCTACAAGGAAATATAGCCACAACAAAGAAAGCACACACACACACACGCACGCTCACACATATCATCAAGATGTCTTTCGCAAACACCAGCCCTCTTACCGGCTTCCTTCAGCTGGCCCCGGCTACCTCCAACTCATACCCTCAAGTTCAGAGGTCACAGCAGCCCAAGACTGAGGCCGAGCCCGCAGCCGCCGCCGCCGCCGTCCCGGCAGAGTCCAACGCCGCCACAGCCAGCCCTCAGCCTGTCCAGCGCCGCAGCTCGAGCCTGAGCAGCAGTAGCAGCAACGACGGTATGCGCTTCCTCAAGCTCGGTCCCGTCCACTGGGGAGAGCACATGGAGGACCACCAGGGTGACTTTGCTGTCGAGTAAAGTCTACATCTGAAGCATACCTGACGCTTCTGTTGTAATGAATTCTCTGGGTCTATCTATTTTTTTATTTTTTTCACTTTCCTCTTTTTTATCTTCCTTGGTACACTGGTGTTGTGCAGGCGCTCTATCTTGGTCAAAAAGCGGAACAAGACAGAGGAATGGATTGGATTGGATGGACGAATCTTTTGTTTGGAAATACCACCACAGCTACGATTTACAATCATGACGAGAGCAAATCTCAAAAAGAAATTCAAAAAATACCCTCTTAATGATGCCTTTTTTTCGTCACCATCAGTCCCCCACAGCATGCCACGTCGGAAAGGAACAAAAAACGCCAAAAATCCCACTCTGCATGCTTACGGCAGTACACCGCTGTCCTCATGACAAGCGAATATGTGCATTGCATTTGCCCAAGATCGCGATTTATAAAACTGCCCAATGCCTATTACGGGTTTCCGCACAAGCAACCACACACGCACGCACACACACACACACACACACAAAAATAAATATGTACCCGAATCTCTTGAACTTCTTTTTTGTTTTTTTCTCTTTTGTTTCTCTGACCGGCTTCCGGAATTGTCGGCCATACACCCTCAGAGAATGATCTAGTGTGGGGGGAAAGAGAAATAGTCAAGGAGGGGTTTATACCGTAATCCCCTAAGCCCGCCATGTTATCTGATAGGGGCGTCAAGAAACAAACAAGAAGCGCGAATGTGGCTTGCTCGGAAAAGGGT

CCGTGTCGCGCTCCTTCAGTCCTGATAAAATGCTGCATAGTAGCTATGAATTCTCACTCCATATCAATTGTTATTATTATTATTATTATTATTTTATCTTTTCATTGCGCAACTTTCTCTGTGTTCCCCCCCCCCCCCCCCATTATGTTTTCTTTTTTTAAACGAATGCCTTGCCCTTCCTTCATGGATGGGCCTGTTTTCTCCGGCAAAAGTCCATAAACGCCG

TACATTACTCCCGTAACCAGCCTTAGCCCCCAAATACACTTCTTCTCCGTAGCAACCATCACCTTGTTTATAACACCGTGGGAGTGAAAAAAAAAGATGGGATGATAAACTGACGACTTCCCGGGCTGGACGGAAAGGACCCGGAGGACATCTTTCTCATCCGTACGTCCGGCGCCGATGATACTTTATGAGGAGACAAATGAAATATGTATAGGCCGAGGCAAGCCGATCAGCAGTTTTTTTCGTTCCTCAATCTGCATCAGGCGACCAACCATACCAAAAGGGGTGGCATCGCCAAAAGGGAAACAATTGAGGGGTCCCCCCCTTTAACCTGCGATGCGTGCATGAGGGTGGACGTACGGAAAGTCAAATCTAGATAACTACAGTACGTACTATATAGTATACCAAGTAGTAACCGCATGGTAGAGTACCTATCTGCATGTGCCTAGAGGTTGGGTACGGCCTTGGCAGTAAGCGGCAGCAAAAGGACCAAACCTTCGGAGCTTGGGACGTATAATCTGACATGTAACCAAACAGAAAATAATAAAAGAAGCAAGGTCCACCCCTTTCCCCCTTCAATTCTCAGGGCCAAGGCGTGTCTCAAAACGAGAAAACGTCATCTTTTTTTCAGGATTACCATAACCGGAATCCGTGAGAGAAAGCATTGGACAGGATGGCATTTTGCGAGGCCGCGTCTATGGGTTGTAAAAATGGTTAGCCGACCTTTCCCTGCATATTCCCAATAACGGCCTTTCCGGTATGTCCCGCGAACATCCATTCTGGGTTGTAGTTCGCCTTGGTATAGGCACGCGACAAAATACATGGCACTGAAACAGACAATGCCGAGGCAGAGAGATCTTTTGAAGATCTCGATGTTCTTTGCTTGGGCATGGGTTCCCCTCTTTTCCACCATTTCCGCTCGCTTCTATTGTAGGTCCCTGGTTGCGAAAAAAAAAGCTCTGTGATCTCGTCAAGTTTGGTTCCTTGGTCAGTGATCGTATATCATGAAGTATCGATTCGTAATAAAACCCGGATCCGAGGATGCCTCTGCATAGCTGCCTTAAATGACATTGACCTAGACCGGGTTGTCAATCATCAAGTACAGTATGAACGAACAATACGTAGTCATGATATATCTGAAATTCCGCAGGTTTATCCTGCCTGCCGCCCTCCATTGAGACTTTGATGGTTCCAGGAAAAACAAAAATCTGCAATTAGATGTTCCACCATTTCGCATCTTCGCCTTTGAACTTGCGCAGCATGACATTGAGATTCAGCTCAGTCCAGCCAGGAGCTGTGAAAGCAACAGTCGCAGGCGATCCTTTGCTTGAGTGACACGAGATATCGTCGTGCAAATCATCATGGCAGGTCGTATCGTCATCCCGCACACACCCACTACCAGGCTTGTGGGCTTGGCTATCGTGAAACTGACACCAGAGAGGCGAATGCTCCTTCTGCGGAACGCTAGCACGGGCGACGGTGAGGTCAGGATCATTGAGCAGCCTGAGAACTGCAAGAAAAGCCTCATTGCTACCGAGGATCTTCTCACTCCTCTTCAAGCTTTTCTGCACAGGACCAGCACTGCCCATCGCAACGCTGCTCGACTGCCATGATACGGATGGACTAGGAATCATATTTCGAGAAACCTGATCGTTGAACCTCACCTCGAGCCAGCGCAGCGACCCCCGTAGAATAAGATCCTGAAGCGCGGGGACGACCTCGTCCCCAAGAGACCCTCCGAACCTCTGCACGATCAGAACCAACTTCCGGATCTTGAAGCGCGCCTCGCTGCCCATCAGGTTACGGCAAGGCGTCCGGGTGCTGATTGAGCGGCAGTCGACGTCATCGTCGCTGTCGCTCGGGTAGCCGTGGTTGCGGCCCAGCTCTTTTGGTTCCGGCT

**> MD204_chr02:1335169-1339169**

GATGCCACAGCGCTATACAGAGAGTTAGTTGATTCCCAGGACGAAGGGCCATGTAAGAAGTGGTGGCTGGTGAGGACTGAGGCCTGATCTGAAAAGGGCACAAACCTCATGTTGGCAGACTGAGGAGTCGGGAGCGAGGTGGACGAATTCGAATTCTGAGGCACTTCGTCCACGGGGTCGACCTTTAAAAGAGGAGGACCTTGATCTGACGGCTGCGACGTTTGGACGTTGAACCAAGCTGCGTTTAAAATATCCTCGGAGAGCTGCGGAAAGGGTGCATGTGAAGTCTGTGATGCAGGGGTCGGTAGGAAGCCCTGGATGTGCCGGACGGCGAGCTCGGGGCTAATATAAGGTGAGGATGTTGTTCTTTGCGAAGATGCCGATGAAGACTGTGTGCCACTGAGGTCGAGTGGTTGCGCTCCTCGTGGTGAGCTAGACAGTTGAAGTGTGTTGAGCAAACTTCTTGTTGTCGAGACACCGCTGCTGGCTCTGGACCTATGGTTCAGTGAGTGACTGTCCTCGTGAAAATGTTCTGAGGGGTCCATCGCGAAAGACAAAGGAAAAGAAGAAAAAGAAATAGCTGGCTGGTAGCAGTCCAGGCGGCACATACGGTGCTTGTCGTCGAATGAGTCCGTGGTGGGGCGTGGTGTGGTATGTAAGGTATCTTTTCTACTTTGGTGCTATGGGAAATAGCTCAGATAAACATAAATGAGGCTTCTTTTCGGAGCAGATTTAGGTTGGATGCGTGATTCAGGGTCGAGATGATTTTCGAGCTACGTCCCAGCAGCGAAGCTGAAGGTGCGGCGGTAGGGCTCAGATATAATATAAAGGGGCGTGCAAGGCCCAGGCACCGGTGAAACAGCGATGGATGTCCCACGAAGAGTGATATGAGGACAGTCGAGGAGTAGTAAGGTTGGGTTTGGCGGATGGATGCTAGCCTTGGCCTCAGCCACTCTGCAAGAGACGAGTGCGAACAAGCCGGGACCGGGGCAAGGTTATATCCAAGTGCAGAGAGGTGAAGAGGTGTAAAGACGTTGTAACCTAATTCCAGTTGATGCGCACTACATCTTTTTTTTTTCTTTTTTTTTTCGTGGCGGCAGGGAATTTCGATTTAAAAGATATCCAACCTAGATGAGCAGCAGCTCCTGGGGTTGTGAGGGGTGGCGTGGTTCTAGGGCAATACCGTAGCTCCATTTGGTCTTTACCCTGAGCAGAGGACGGGAGATGACGGGGGTGTAGGTACACACCTTTGACCGGGGAGGGGGCGCAAGGGGGCGTACATCGCGTCTATCAGACCAAGTCTCCTTGTACGCGCGATGGAGAAAGGTGTGAGTGGATGATACCAGGGTTGATTTCAAGGCAAGTTCTTTCATCTAGTACTGCGTACTGCACTTTATTGCTTAATGTAAAGGAAGACCATGTACGTGTGTAGTAGGTTGAATAGACACAAGGCCACCGCATTGCAATCTCGCGAGGCATGGGCAGAAGCGAAGCAGAAGCTTTGGACAATTGGTTACTTGTTGCAAACCAGAGAGTCCCATCCACCCCATCTTACTGTGCGTTGTCTCTTTTTTCCCCCTTGGGACTGGACTTGTGCGAGAGGAGCAAGTGGTGGAGGGTGGTGTCTGTTTGTCTTTGCTAGGGGAGAAGAATAATTAGCGAATAGCTGCCAATCGAATCCGGGGGAGCCCACAGTTACCCTGTTTGTTGTCTGCCTCTGTTTTTTTTTTTTTCTGTACATTTTACGTTAAGGGAGGAGGGGTGAAAGAAACCCCCAAGGGAGTGAGCGCAGTCTAGCCGCGGGTCTTTGGGGCGCGGGTCTTGTTAGTAGTACTTGTAGTAATTTTGGCTGGGTGGAACTAACGCATCGTTTCC

CCTGCACAGACGCCGACTTGAGATGAACGAAGAGTGTACCCAAGTAAGCCGGATCGTCCAAGCTTCTAGGGAAACCCCAGCCCCTAGTCGTTTTACTGTACCATACTAGACCTACCGGTAGCAATTTGTTGTTGTTGTTGTTGTTGTTGTTGTTGTTGTTGTTGTTGTTGCCTGAGGCGCCGGGACCGAGAGGTTTGTCCAACTCCCGTCAGCTAATCTTGGCCTTTGTGGCGGTGCAGCGACAAAGTCGAAGACTAGGCTACCGAGATTAATCATGTTGTAGAAATTGATTACAGTACT

TTGAGGCAAGCAAGTAGTAGGTAGGTAACTAAAAGCCACCTTAGGCGACGGTAGAAACTAATGGGAAAGGAGTAGTCGGAATAGGCAGCTTTGAAGTCGTTGAATTTTGTTGTTCAGCAATTGCTAAGATACTGCGGGCATGGACAAACGGGGCAAACATGGCGGTGATGCTGACAAGGCAAGGTGTACCTACCCTACCACGTTTGAACGACGTCCAGTATTGGAAACAGGCTCCACTTCAGCTGCTTCTTTTGGTTGTTCTGTGTGCCTTCCGTTGTTACCGAGTGGAGTATCGGCGACTTGGTGGATTCAGTGGGGGAACAGGTTGGTGAGAAACTAAACCCCTTCCAATCGGCGCGTTAATTGAGCAAATCAAGAGTTGCAAGCCCATCCCTGCCGTCAGATTTGTCCCGCTCCACTCCACTGTCGGAGGCGCGAAGTGCGGTATATGCTATGTACCGTACGCCGAACCTGCCTTGTGTAATGTTGACGTGGTTCTAGCGCAGCTTTGTGGTGGATGTCTTTGCGGGTAGACTTGCCATAGCAGCATCGGAATCTGAGATTTCCATGTTATAATATTGAAAGACGCTTAATATCCATAATGTCTCGTCCTACGAAAGACCCTGATGAGCTTGATCAATTCTCATCCCATCGCTTGGGTACAGCGGGCATTAGGCACGTGTTGGAAAGTGGGGGGGATTTGATAGGTACCTTAAACAGGTAGGTACTTACTTTGTATTGTAATACACCCCAGCGTCGCTCGGTATTTTGCTGCACGGTAGGTCAGAAGCCCAGAGATAGAGTGGAAATCATTTTCTAACTCGGGTGAGCTACAACGGTAATGCTGACCTTGACATCCATTCAAAGTGGGGAACCAAATACGATACAACGTGGTTGAGAAAAGATGCAGGGTACTTTCAGAGGTACTCAAAAAGAAGACAGTGGCACGTGCACTCAAACGGCCAACGCGCACATATGAATGGCGATCCATGGCAATCGATTTTGACGACATGGGACCCACCATGTTGGGACAGGAGGGAAAAAACGACACCAAACATGAGTTCGTTGACAGTGGGTGAGGGTTTTTTCTCTCTCTTTCCCCCGAACACCATTCCACATGGCACCGCACAAACACCACAATCCAGTTACAACACACCCAAATGGCTCGCTCATACAACTTCTGTGGGCTCCAGCACTTAAAGGATTCGAATCCCTGTCGATTTCTCTAATAAGCGGGCCCGACAGCAGCATTGTCGCGGCAAGGCTAGTGGCCTTTGTCGTGCCCCTTCAATCATGATTTCTGGAGACGGCGAGCGGCAACTAGCTGACGGCGGATCACGCAACTGGGCAACTGGGAAGCTGCAGTCGCACCATGCCCTACCCACCGACCGCTTGCTTGCTACAGGCCCCGTCACCAGCATGAGATAACCTGGAACGAAAGCACATGCAATATGCTGCCTAGAGGTATCACCTACGTACCTACTGTACCTACCCACCTTACATTAGGAACATACATGCCTTATTTACTATACTGCCCTACGGGGAGTAGCCGGAACGTGCACCTCGCCCTACAGTCGTTCGTTTTATCACAGACAATCGCCAGACCTAGTCCTAGAACTAGGTAGAATCCATGCACACAGCGGGACCGCCTCTCTGTTTTATTACTACCTATGATGATTGTTTCGTTACTGATTCAAATTCCGAATTTCCGGGACCGCTTTATATGTTTAGCCACTATGATTATCATTTTATTACTCATTCAGGTTCCGAAATTCCGGCTACAGGTCTTGAAAATGGTTCGAAATGGCAATTCACAAAGTCGCTG

**> MD205_chr02:1778411-1782411**

AGCTTTCGCTGCTGCCATTGCTCGAAGGCAGGCTGGCGGTGTTGGTGTTGCCGCCATGTGAAGGAGCCAAACGACTCTGTGTCTGGGGAAAATCAGGACCCTTCTGGCCAGTCATTTCTCCAGACTGGCCGGCACCCTGTGGGGTAAAGGGTCGACGGTATTCGGCAGAATCCAGGGCAGGCATGCTGCGGCGTCTGAGGCGCTTTTGCTCGTCGGGAAGCTGATGGCGGTATGCCTGGAAGTCATCTGTCGAAGAAGCCGTCGTTGAGCTGTAGAAACTGTTGGGTCGGTTGGTGTTCGACGGAGTCCTGTTCCTGGGGACAGCAAGCCTCTGGTCGCCAAACTGTGGGGGAGGTAGTTGCTGTCGACCTGCCGCTCCGGCAGGAACGTCTACAATTCGAGGAGCGGGCCTCCTGTAACGTTCGGGAGAGAGTCGGACCGGAGTTGCCTGTGTGAAGGTCACCTGGGTTGCGGTTGCCGTCGTAGCGCCAATAAGATGAGCCGACTGGGGTCTTGGAGCGGAGGGATACCTTCGACCGCCGACGGCTGGCAGGCTGGAATCATCCCTCGAGCCAGCGCCATAATCTGCATAAGAACTGCTGGGTAGGTTCGTCATCGACACACTGCCGGGGTACCGACCACGTTGTTGTTGTTGTTGTTGAGAACCAGAGCCACTGAACGCCCGCATCGTAGGAACTGCCGGAGAGGAAGCGGTTCTGTATGTCGCAGGATGTTGCCATTGGTTGTTCGAGTGGAGGGTCGGTGTGCTGGTGAACGCATACGGCTGTATCGGCCCAGGATTCATCCGATAGACTTGTTGGCCACCTGACCCGCCATAATAGCTGTTTCGTTGCGGCTGATTCCCTCCATACGGTTGATTCTGCCCTTGCATCCCTGACGCTGAGCCCGGGCGTGTATGAAGCATCGTGATAGATCCGGACTGCTGAGGATATGGTTGCACCAAGGCAGCCATAGCGGGACACACAAGTGCCTAGTGTTGAGTACAGAGTATTCTGTGCAAATACAGAGGTTACCCGGTTTGAGTAAATCGGGTTTCGATGAGTAAAACGAGCGGCGAGTTTCCCTTCGATTAGAAGAGAAGCGGGAATGCAGGTAACGTAAGGCTGTTGGCACCAGGAGGATTGGTTAGCAAGAGAGTCACGGACCGGCTTTTGAATCCCAATATTGACAAGACGGAGGCGATGTGCCAGTACAAGACTTGCGAGTCAGGGCAGTACATTAATAGGCTGTCTGACGCTTCCAGCTGGGTTTAACTTGCCAATTATAAAAAGCCAATGATGAAGTTGTCAGGGTTTCTCTCGCTGGGAAATCAAACAAGCAAGGTGATTTAAATCCAAGTTGAACGGCCGGGAAAAGAAAAAAAGCAGGCAGATAGGCGATGTGATGTAGTAACCCTCGGCATACAAAGGGGCGAGTTCTCAAATTCAACGCAAGCAAGAAACGAATGTTCAAAACGAGCAAAACGTAGAGGGACGCCTGCGGCCAGCTGGGAAAGATGAAATGAACGAGTGTTTGGTGTCCAGGGAGCTGCTCACTTTTACCGTGAACGTTGACCCATGTGAAGCCGTCTGTTGCTACGACGAGTTGTTTTGTTTCTTCCTGGAAATCGTAGTTCGAAATTACCACGCTACAGGAGGCAGTAGTAAAAGAGGCTCGGATCGCGTGGGATTCTGGTTCGATCTTCGATGGTGAAAACAGATGAGAACAACGATGGACTCGGGACGAGCGCAGGTACGGTACGCAAAGATGCTTCAGAGCCTGTGGTGGCAAGATGTCGAG

ATGGAGTCGGACGGAGTAGGCAGGACTTTTGTCGGATCGGACAGTAGATTGAGTGCCCCGAGACAGGAACACGGCACGGAAAGTGTACCTAGAAATGCACGTCGGGCGGCCTGCGGACCACCAACCACCAAAAGAGTCCCAAATGGGGAAGAAAAGAGACAAGATAAAAGGGGGCAAGGAGGACAGAGAAAGAAGAAAACAAAAAAAAAAAAAAAAAAAAAAACACAGCAAAACAGACACTCAAGGAACGGAGCGGGTAATAAAAACGAAAGACAACAAAAAAGACACAGAAAGCAACTC

TAATGTGTGATGCCGAGCTCCAATTGTCGATTTGGCAGAATAAAACAAGATAAAAAAAATGCGTAAACGATGGGCAGGAAGGAAGAGACGGGAATAGGGGCAAGGGCAGTGGAAGGTGAGGAGAGGGGCAGAAAGGAAAGGAGATACCAGGAAGCCGGTCAAATTGCTGGAAGCGGAAGTTGAGATGAAAAGCTCCAGAAGGTGATGTTCTCACCAAGCGGAAATTCTGCCACGGAAGGGTTCACCTTTGCTTGGAATACCCACAGTTGTACTGGGGCAAGTAATTGCTTGATTGGCCAGGGGTCCTGTCTCCCTCGGGCCTTTTCCCCTCTCTTTTTCGCTGTCTCTCTTGTTCCTTTCCTTTTGTCTCCCTGCAGCAAAGAACTGGAACTGTACGGGTACAAAAGAATGTTACGGCGCAACCTTTCACATACATTCTCTCTCTCTCTCTCCCGGCAGGAGGAAAAAAGCGCCCCCCTCCATAGAAAAATAATATCCCAGCTCACTCGTTGCACCCAGAAACACGATAATTACAGGGCACACCCCTCCCCAGATGGGCTTGACGGGCGGCGTGTCGACGCTAACAAGCAGCGCACGCACTGCATGTCAGTTCGCCGCCTACTGGCGGGCACGCTAGGTACAGAGAGAGTACGCCCGTTTTTTTCTCTCTATTTCTCTTTTTTGATCGGGATTGGGGCCAGCCGGGGTTGCTTGGAGGGCCCCCCCTTGCTGGCACCACACTTGGGTGGAACATCGATTATTTTGAAAATGGCATCCTGTTCCTTGGGTGGACTAACGCTCAGGCAGCCCCCGACCATGGTTTGCCAGGTACTTGTACCGGATATGAACGTCTCAGCCATACCAAAAGTACCCCCAAAACACATCGCCTCACTACGCCCCTCCGTTGGAATTCCCCACCCCATCTCGCATTATCAAGCAAATATCCACCACGTTCGTTTCCCCAGGACGTGAGCCGGTCTGTGATTGGATCGACGAGATTCACGTTGTCGCGATTTCGCTCCGCTGCACCCCGACAAACTCGAGGGTAGCTTGCCCACCTCCCTCCCTGCGGGTCACGCATTGAAATCCAAACAATACGCAAACAGAAAAAACCAACCTTACGTGGTACGGTACACACGTAGTAATAAGGCAAGACTCAGTGATGTGCAGGGGTGGTGGAGGTGGGTGCACGGGGCAGAAGAAAAGGAGTGATGGAGAAAGCTGCCGGATACCATTAGCAGATTGCATTTTTGTTTGTTTTGTCCCGAACGCGCCATGTAAAGGACTTTGAAAATATATTTCCTTTGCTTGACAAAATGACAAGGTCTCTTTGACCAATGCTACGCTGATCAATCATTCAAAATGCCAACTTCTACGTAATAACCTGACGACCTACTCAAGCTACTGAAAACCAATTCATTCACCTGAAGCTTTGTTTTACTTGTGGAGCTGTATTCGTCCGGCGAGCGTTTTTTTTTTTCCCCGCTAGGTCGGCAGGGACGGTTCAACGACGTGAGGCTCCATGCCATTCTTGCCCCTGAGTCCGTGCCGTTTGACAAAAAAGCACGCTTACCTAGGTGCTTGCCAAGCCACCGGGGATTTGAGAGTGACGCATGTGCATTTTACAGTGCTATACAGTAATTATCATTACCAGACTAGGTACGTATCGTAGATTAGGCGCTTTGTACATAGAATTAGGCAGGCAGGTAGGTACCTACCTAGATATTCAAATTGCCCACTGGTTAATACACGTTGCCTGTACAAAGCAAGCAGTTGAATTTGTGCTTGCTGCTTGCTTGTCTAAGGCATTCATTCATCCATAAAAAGCATTAGAATTACAAGCAACAAGAAGAAAAAGGGTTTCTGCTTTACATTGTTTTTTCTTTCCTGAGGTGGAAAT

**> MD206_chr02:1782234-1786234**

TATTCAAATTGCCCACTGGTTAATACACGTTGCCTGTACAAAGCAAGCAGTTGAATTTGTGCTTGCTGCTTGCTTGTCTAAGGCATTCATTCATCCATAAAAAGCATTAGAATTACAAGCAACAAGAAGAAAAAGGGTTTCTGCTTTACATTGTTTTTTCTTTCCTGAGGTGGAAATACAACATGCACAAGCCTCCCGAACTTACCGCCTGCCCAAGATAGCAATTGATCTGTGAACCATCAAGTATGGTAGTATTCGACAATAAGGTATTGTTGGATTTTTCAAAGTGCATGACAGCAACAGCGCTTGCTTCAGCTGAGGAATCTCACCTACCTACAGTACATACTACGTAAAATAGAGCCGACCGTTTGTTTCTGGTGGTGGTAGGTGTGGGCATTCGGCAGGCCGGGTGTGCGTGTGTCGAGGCTGTGTGACTTGGTTGGCCCCATCCATTTTGCTCGTGTCTGTGTCTTTTTTTTTTCTTCCTTGTCCGCTCATATGTTCCAGTTGATGACATGATTCGTATTCTCTACATCAGACGGTCTCTGTTTGGTAAGGTGCCATCCATCGATCATCACTCGTACGGGACGCTTCAAAACACAAAGCCGATCACTGCCCTGCAACATGCCAACTTTTTCCCAATCGGTCGCCTTGCGCACAAACTCTTGTACAAAAAAAAAAGATTGAGATGAAAGGGGTGATGTACATTATTAATGAAATTGCCATTTGCACCAGATTTTGTGTCGCTCGCTCGTGGGTAAAATCGCCACGAAGGCTGTGGAAGATAACAAGGAAGCAATAATCAATCGCCCATAGAAACAGACAAAAAAAAAAAAAAAAAAAAAAGACCCTACCAGGACCAGAGTCCATCAAGGGTCTGCAGCCTATAAGACCCCTCATCCTTGCTCTTTCGTCCGATCTCTGTTGCCTTCACGCGGCATGTCTCGGAATTTTCTAGGTATTCATCTCGTAATTATTGGATTTGGGCTTTTCAGCAGATATGCCACCACTAGGTTAGCTCTGACCTCTGACGCAACGCTTTTCAGTCTCTTTGCAATTTTTTGCTGAAGTCATGAACCTTGATTTTTTTAAAGAAAAAAAAATCTATTTTATTTATGGGGATGACTTGGCAGATTAGTCTTGGTGCATTGCCGCACCAAAATGATACTACTAAGCAATTCATCTTGGGAGGGGAGCTTGGGGAGATCATCTCTAAAGACTCTATGTGGCTCCAGCGCCCTTAGATGGAATATTCCATTTCCCTCGCCTCGGCACAGATTGGGATTGGACGTTTATGCGGAACCCAACATGAACATCCACATTACGGAGTATACAACACGGACGTCGAGAGAATTGAAAAGAAGCAACCTTGTCAAGATTTTGTTACAAGTAAGTAGGAAAAAGCCGCCGACGCCGACAAGCTTAACCATGCCTAACCGTACCCTCTTTTGGTTCGCTTGATGTTGCTTTTGGCTGGTGTTTATCGTCGATCTTATTTGGGGTTCGGAGCCGCAGCTGAAGTGTTGCTTTCTCAGCTCAACGGGTAGGTCGGTACTTTACCTACCATTGAACCTACAAGAAACTACGGAGTATCCGTAGTCTCTCCTTGCCTACTGTAATACAGAAAGGTACTGTACTGACTGATGCATACCAAGGGAAAAAATTTCAGTGCTATTAATAATAAGTACAATCCAACCATACAGTCTAATTAGTACATGACTTACTTTCGGACGCGACGCGTCTTTGTTTGCCAAAACAGAGCTAAAGAAAAAAGCAACCTTCGTTCCTTAGGGGGTGTGCCTGGTTTAACATGCAAGCCCGGGACGGCTGGCATGCTTGTGGACGTACGGTAGTGAGGCGCCCAATCTGACAAGGCTTTGCACGCCACCGTTTTCCCGTTCCGCCATGCATGCAATGGCGTCGTCTGGTAATACCATGTACTTTCTTTTT

TCCCTTGCTCCCTTCTTACATGACGGTGGCCCAATTAGCGAAGCATGCGAGTGTGTGTGTGTGTGTGTGTGTGTG

TGTGTGTGTGTGTGATCTCCACGGCACTACCGTAATGTACAATTATTAAATCAAATTCCACATTTATTCCGTAGTCGAATTTATTTTGTTACCCCGGTTATCAAATTGTCACGGCGCAACCTTTGATTCTGCCCAGGTTGATTGCGTACCTATATTTTCAACCCAATTTTCCCCGGAGTTGTATCTCCTGTCACTCGATCTTTTTTTTTTCTCTAGCGACATGATACTTACGAGATTTCGAGGCTGTGCTATTTTGCTCGTGCGACGTCAGCTGCAATGTTTTTCAATGTCTGAACGCAACGCTAGTCAAGACAAGCCAGACAGGCGGGCAGGTAAAGTGTATGTAGGCAGGCAAAGCATTTCAAAGGTGCGGTACCTTGGCACTCTAAAGATCCGATCTCGCCTGAGTTCCCAGCTTTTGTTAGTCAGGTGGAAGAGAGAAAAAAAAATAAGGATGCAAACTAGTCTAGACTCTAGACATGGCCCAATGGTTTGGCGTCCCGCCGGCCTCACCGCCGATGTCTCTCTAGACTCCTTTGCCGATCGATTCTCTTCAAAAACCAAGAAATCAAGGGAAACAACAGACGTTAGATAAATTGAGGATTGGGACGTAGCATTGGCTTTTGGGACTAGTGGGGTTATATTATGATAGTACCTCGTACTCTACTGCAAATAGTAGGTTTTTTTTTGTTTGCTCGTTGGGTGAGCTTTTTACTCAGTTACAATCTTGCAATGAACCAAGGCTCCGGTGCATCTTGCATCTTGCACCCTCCACTTGAAAGCGGAGCGGTACCATTCTTTCTTCTTTTTTTCCGAACCTCGTAATCGGTCAGGGTGTTGACCTGCATGCAACACTCTTACTTTAGACCCCTCCATGTGGGCATCATCCAATCACCAATCCCATATGCAGATAATGACTTCTTGGAGATATCGTCATTAAGGACAGCCGCCATGCATGGCGAGAGGGGGGAAAGGAGGAAGGAAAAATAAAAAGAAGTTGCGCAGGGAAATGACCTGCACATTCTCGCTTCTTCTTGACAAATCCGTAGAAAACGGTCCCGGCATCGGGGCGATGGTCGGAGGTTGCCGTTCGGGCGCAGTCAATCTGGAACAAATCAATGCATTGTTATTTAGGGGAACAGTTTTGTGTTGCAAAAAAAAAAAAAAAAAAAAAAAAAAAAACCGCCACCGCAAAAATTTATTTGGGTATCGGCACTAAGCATAGTAAGAAACCCACCTTATGCGATATAGCGAATAGCAAGAAGAAGAAGAAGAAGAAAATATATAAAAACAAAAACAAAGAGAGAGAACATCATTGGTTTTCAAGCAAGTATGATGATCCCTTTCGCGACCCTCCCCGGGGAATGCCCCCGGAGCGGGTTGAGCGGGGAGCAAGTATCGCCCGCCGATACCCACGATATGGGGGTTGCACCAAGTCCAGACATTGTCAACAGGAAAGAGACCTTCCTTTCCACCTTGATCTCCCAATCATTTTCAAGTCAGGGTACGACTGGTCATTGAGGGCCTTGTGATACTCAAAAGGGTTTCACCACTTTCCTGCTGTGAAATGCCGAGGACATATAGGTCCCGCCGGGGCTGAATGAATCTACCATGTACAATAGTTCCGGGTTGCAAACAGATACAGAAAGGAGAGCGAAGAAGAAACAAGAAGCAAAAAGAAATATATGGTACCTGTAGAAGCCTCAGGGCCAAGAACACACGATGCATCATGACCCGGTGCTACTGCTTTAACATAACATGCTAATTCACCCAATCACAGCTTTCCCGGGGGCTCAACAAAACCCAAAGACCCCGCGCCGAGGAATGTTCGCTTCCATATCCCTCCGTCAGAACTAACTACAATATGCAAAAAAGTCTTCATGCAAGGGATAACTGACTGTACTGGGTGCATCATGCTTCCGTGTATCTGGTGTGTTATGATGAACGAACCGGCGGCGGTAAGCGCTCCATTAGT

**> MD207_chr02:2105779-2109779**

GCAAGGAGAAGAAAAAAAAAGAGGAGACCGGGTGATAAGGGGGCAAGAGGGGCTGTATATACTGTACTGACTGGCTTGACTGGATGCCTAATTGAATCTTGTTCGACAAGACATTTGAGATATTATGTCTTGTCTGGCCGACGCTTTAGATGTGCCAAGAGGTGCTGTTGCGCACAAAAAGTTCCAGGTTTCTTTTTTAACGAGGCAAGTGGTACTCTCCGCTTGCGGGCCACAGGAAATTCCTCTTTGAGCGTAGATGCACCCAGTTGTGGTGTTTTAAAAAAAAGGCCAATTAAAGCCCGATGGAACCCAACCTTGCCAACCCCCATCGTCTGTTGTATTGGTCCTTTTCTTTTTTTTCTTTTCTTGTCGGATTGTTTCGTTTCGGGTGTGGTTTGACTCGATAAATTTCTCACACAGCATTTGATTTTTTCTTTCCCCCTGCAGCTTTTAACCTTACTTCGTACCTTACCTTACCTTACCTAGCTTACTCGCATTGGTTCCCCGGATTTTTCGACAAAGGAACGAGGGACAAAAACAAAGAAAGAAAAAAAAAAGAAAAAGAAAAAAAGAGAACTTAAAAAAAAGAAATTGATTAAAAACTTGACCAAGGTAGGTCAAGGGGTACGTACTTGATACCTTTTTCTGACAAAGTAGGCAGCCATGGATGTATTCGATCCGAGCGTCGCGCGACTTCTTGCAACCACCAGCGGGCAGCAGTCCACCTCTCCGTTGGAGTCAGAAGCAGAAGTGGGGTTGCAGTCAGGTACGGGCGAGGGGCCCGAAGCCTCGTTGAGTGGTGAATCGTCGACATCGCAGGGCGGCGCGCCATCGGTGCCCGCGGCTTGTCTAGCGTGTGTAAGTCGCTTCATTTTTATTTTATGCTCGAGGTACCCTTTGCAAAAAAGTCATCGCAACCGACTGTGCCATTCATTCCGGCTAATCTGGGATTTTGTTTTGCCGTGTGTGGTCCGGGTTCGCCCCATGACCACCACCAAATCCACGTCGCAACAAAAATATAGCGCGGTAAACATTTGAAATGTGACGGCGCAACCCCGTGCTCCCGCTGCGTGGCTTCTGCCTCGGAGTGTGTCTACATCGCGTCGCGGCGTGGGTACAAGGGCCCTCGGAAGAGTAATGTGGCACACAATCCAAACAAGCGCTTCGCCTCGTCGCCGACCCCAGGCTCCGCACCAGACGACGACTCGTGCCCAATGCTCCTCGGCGCGAGCCCATCTGGCAGCACGGTCACGACTGGCTCCTATACCCAGGCGCCGGTGCTGTCGCAGTTCGTCGCGCCGCCGGTGGGGGCCAACATGCAGCTGTACAGGAACCCCTTCCTCATGGCCGGCGGCGGCAGCAGCAGCAGCAGCGCCAACACCAACACCAACCACGCGGGCTCGTCGCACATGCGGAGCCAGCTGAGCATGCCCGAGCGCTGCCTGACGTCCTTTTACCAAAACTTCCACGCCTCGCACCCTTGCGTGCTGCCCAGAGATGCGCTCGAGAGGGTCATCAGGGAAGGGCCGGCCAAGCTCGAACACCTCCTGGCGGCCATGCGGTACCTTGGCTCCCTGTACCTTGACGCGGGCCCCGCCAGGGCCCAGTACTTTGACGAGGCCGTCAGCCTCGCCTACCACCCCGGCTGCCCGAGGGATGGGTTCCTTGCCCAGACCCTGCTGCTGCTGTCCATCGGCCAGGACGGCAGCTGCAACCAGGCGCGCGCCAACCAGTTCATGGCCGACGCGGAGAGGCTCGCCGTCGAGATTGGCCTCAACACGCGCCAGTTCGCCACCGCCA

ACGGGCGCGGCAGCTCTGTCGTCGAGGAGAGCTGGCGACGCACCTGGTGGGAGCTGTACGTGGTGAATGGCATGATCGCCGGCGTTCATCGCATCACCAATTTCGCGCTTTATGACGTCGCCGCCGACGTCGGTCTCCCGTGCGAAGAGCACCAGTTCCTGTCGGGCGTAAGTTGATTTGAGAATATCCGGCTCTTTTTGTTCTTTTTTTTTTTTTTTTTTTTTT

TTTCTCCATGAATTCATTTGCTTTGACAAAAAGTGTCCAACAACTGACTTTGGATCCGCTCATTCACGACACACAAACCAGAATATTCCCATGCCAATGTACATGGAAGATTTTGACGATCAGCTCTTCTCGGGCGAGGATCGCGAGTTCTCGTCCTTTGCGTATCGGATCGCGTCGGCTCGAAACCTCGGCAGGATGATGAGGACCCCGCCGATGCTCTCGCCTGACGATGACAACCTAGCCAAGCTGGAGGCCTTGCTCACCAACTGGAAAATGCATCTTCCGCGCAGCAGACAGGATGCGCTGAACAAAAAATGCCAGCTGGACGAGATGATGTTCCAGGCTCTGTTTATCAACCATGCGTAAGTTTTTTTTTCTTTTCTTTTTTGTTTTTGTCAGCTTACTCGCAGCTTAGTCTTTTCGTTCCATGGTTTTGCTTTTCAGCTTTATTCCGAGTCGGCGATCTCCAGGCTCTAGCCCGCTGACCAGGAGAGGGTCCCCCCTGCCTAATACCCCGAATTCCAAAACGCTAATTCCCGATTCGATAGCTGCACCATAATGCTCCACCAGCCTCATTCCCAGCTGGACTCGTCCCCGTCCCGCAGCGTCACATCGTGCGCGCCGCACATGCCCGTGTCGAGCGGCGACGCCTTCAACACCCACACGCGGCACACGGTGACGGCAGCCTGCGAGGTGTCGAGGATGGTGACGCACGCGGTCCCGCTCCTTTCGCACACGCACTTCTTCACGTGCGTGCTGACCCTCTCGTCGATTGTGCACCTCAACAAGTGGGCGCTCTACTTCCTGCAGGACGAGGACGACCTCCGCCAGCAGATCCGCCTCAACATCGGCGCCCTGCAGCGCATCAGCGCCGTCTGGCAAAACGCCGCCAAGGCCGGCGGTCAGGTCAGGGGCGTCGCCCAGGAGATCTACCGCGCAAAGAAGGCCCAGCAGATCAGCCCGGCCTTTTGGGTCGCCTTCACGCAGGAGCAGATGCTCACTAGTCTGAATGCCGATGAGGGCATCATGAACGACTTTGACTCCATCTTGCCGCCGCAGGTCACTCGGCCTTGAGGAGGGCTCTGTGATGACTGATGAGAAGTAGCAGGTGGTGGGGTTTTGGTGTTTTTTGTGTTTTGCTATGCTGGACTGGGGAGCTCAGGCTGTTTTTTTATTTATTTTTTTATTTTTACTTTTTGTGTATTCTTTTTTTTTTCCTTGAATGATCTCCTGATCTACTCATCTGTGTTTACTATCACTCACTAATACCACCATGTGCGTCTTGGAGACAATTCGGCCTCGACTTGACAGATGCATGCATGCCATCTACAGAAAAATTTCCCGATGAGCCTGTTTTAGTGACAGGAACGGGACAAAGACTTGCCACTCGCCCTATAGGGAGTGCAATCACGAGTCGATGTAGCATTGTTTCCCCTTCTTCCTCCCCCTGGACGTAGTATTCAGGCAAGGTATGCATTCTATCGTCTACCCCTACACTTGTCAAATCTGCATGTCGGAATAACATTTTAGCCCCCATTTTACTAGGTACCTATATATACAGGCTTCCCCACTGTCATCGGCGACTTCCAGGCACGGCTGCAAAAATATGGATACCCCGACTGCAGTGGGGCATTGCTCATTAGTCTAGGTCTAGACCTGGTGGTAAAGATGGAGAAGCCCGCCAGGCCCGCCAAGTCGCCGATTTGGGGTGTTTTTTTTTTTTTTTTTTTTTTTTTTTTTTTGGGTGGCCTTGTTCACTAGTATGTGCTTATTTTGCCAACCAGGGTACAGGTTAATTATTGATTGCGTCTCTGCGGACCGGCGCTCGGAGCCCTCTCCTCCTATAGGGCTTTTCCGTGCCTTCATTGGTCCCTCGACGTTACACCCTACCTCTATCTCGTTCGGACTTTGTGAACCCCTCCTATACGGGTTGACCTGCCTAATACTACCTTGGCAACCCTGTTGCTTTTTTTGC

**> MD208_chr02:2748543-2752543**

ATGCAAGGATGTTCGACAGGCAATGGCAGCAACTCCGTCTCGCAGCACTGGCCCTTTTTTGGCGTCAAATCGCAAGATTTCTTTCTTGATTTCTCTTCACCTTTTTTTTTCTTCTCTTTTTCTTCTCATTATCTTGGGGTTTACTTTCACTTCCCCCCTGTCTTAGAACTGCTTGCACAGCACCTTGCTCAGGGGAATTAATTCATGGGATCTGACCCCCAGAAAAAAAAAGGTCCAGAAAATACTACTTACTGCGTACTAGGTAGTTTTAGTACGTAGTACTACAACGATATCTACCACGAACTAGACTAGCAATCCAGGCTCCACACACAATTCAGCATGAGAAAGATGATCATATGCTGGTTGAGGAACAAAGCCGAATACCGTAGTGAGCTGATGCGTCGTTGTTTGAAACGCTCTGTTGTGATATTGCAAAACCACCTGAGTGCTAAGAATGCACCCCCGTGACGAGAAACAAACCCCGGCCGGCTAGGACCTCCCTTTTCCTATATCCAAAAAAGTGAAAGAAAGAAAGGTAAAGACAGTAGCCCCATCGCAACAAAAAAAAAAAAAAACCAGTCAACCAGCAATGGGAAAAAAAATCTCGGATTCAATTGCGTGGCACGGACTGGCGACTTGGGATACTCGGGTGGGACTGGGCGGTGGTCGGAGTGGATGCCGGGTAGCGTCCGTAATCTAATTATTGACCCCATCCGAGAAGCCTAGGCCATTTGTTCCGTCCAGGGCAACTTGCAAACCCGCTCAAAAGCAGGTGGATCTCGATTGATTGATTGATTACATTCGTCCCAGTAGCCGCCGACCAGTCATTTCAAACGGGATGCATTGTGATGCAAGCCTTTTCTTCTGTTTTTGTGGACATGAGGTGGAGAAGGAGGCTTGTCGGTTGTCAAAAGAGCGAAGCAGCGCCGGTCAAAGAAACAGTCAAAGTCAGATCATTGTTCCTGCTACAGTGTGCCATCCCTCCTTTCTTTTGTTTTGTGATATAATCAAGGTACACACGAGATTGGTAGACAAGAATTGAGGTGATGGGCCTTTTTCGCTGCAACCCCGTAACCACTCCGCCTTGAAGCATTCCCTTCAATAATTAGATAAGACTAAAAAGCTGAAAAATGAGAACTTGCTATTAGCTTAAACCCACTGTCAGGCTCATCCCGCAGTTTTTTTTTTTTTTTTTTGCCCTTGAGCACATAACCAGCTTGCCAGCCCAGTATGCAGGCTAGATACAATCGGTAATTAACAATGCACGAGCCTTCTGAACACAGATCGGCAACACGAGACATCCACTTTTGACAAAACGTATGGTGGACCTCTTACAAAAGAGAAAAAAGAGACAGCACACAGAAAACCCCGAACAGCCAAGGGGTCTAGACGTGATCCCTAAAACCTGCGTCACAGCTATAAGCCCCCAAAAATGACCAGACTTTTAGCTAATATCGTAGACATAAAGCTGTACTCAGCATTCCAGCCGTGATTCCGCTTCGGCAAACCATGCCATGCTTGCACCTAGAAAACCGCCTATCCTGTCCCCTTACGCGGTTAGGCATGCTAGTTCCAGAGTATGCCGGCACTGGAAAAGACCATGTACCGGGCCACAAGGTTTGAACCACTCAAAGTGAGAGCGTTGGTGGAGCCGAAATCATTGTTCCAATCTTCTGCAGGCCGCCCGTTGAAAGTTCAGGGTCCCAGCATCCTTGTTGGCTGGTTTGGCCTTTTTTTCTCTCTTTTTACTTTTTTTTCCTTTTTGTTCCCTCAGGCTCTGGTGGAGCCCAATGCGAAAAGGGGATCGTTCTCGACACTCACACACGTACCACCCTTCTTTTTCCTGCGTGGGACCCACATATGCCCCTACCTTGC

AGCACATGATGACTTACGGCAGAGCGGGTTGGCGCTGCCGCCCACAGGATGCAAGTGACTGACTACCTACATACTATAGTAGGTAGTACAGTATGACTGGACACTGCCGCATGGAAAGAGGATTTAAAAAAAAAAAAAAAAAGATAGACGAGAGCCCTGCAGGAGGACAACGCCACAAACCCCTGTCCCTTTTTGGTTTGGCACGCTACGGATGCAATTTCGCGAAATAGAGCGCACGAGCCAGCTGTGCCCTGGCAACAACAGGCGGCTGTCCGATGAGATTTTCGGTTTTTTTTTTGT

TAAGCCAGTCTTCTTCCCCTGATGTTATCAAACGGGTCGATACTGTACAATGGGGAAAAGTAAACACGGTGTGGGAGTCGAATGAAGAACACGGCCCGGCTTGATTCACAGAGAGAATGAGTTCCCGCTTTCATGGCAAGTCAAGGTGTAATTATTTGCTGGGAGGGAAGAAAAAGCAAAGAAATCAAAAGATTGGAGCACGGCGAAATTTTGTCTGCATGTAGATCCGCTGCGCAAGACAAGTTTGGAGCTCCCGTCCTGGCAGCGACGGCGTTGACAGCCCAAGCCTAAAGCTGGGACAGGGCCGCTACAGGCATTGGGTCAGGTCGAGGGGGCCAGGCCAGGCCAGGCATAATTCCTCACAGACACTCGGTTGGATGGCCGTACTGTGCGAAAATGCGTTTCCATTTGCCATACCCCCATTGACGGAGGTACCCCTTGGTGAGGTACCCTACCGTGCAATACCATACCTCTCCTTCCCTGTCTCGACCCGCTCCACGCTGTGTTGCAAACAAATAAGTACTTGTCCCCTAGAAATAAATCCAGACTCAATCTCCCCTCCTGTTCTTTCCTTCCTCCATCTGTCTAATTCTATCCTCACAGAGATCAAAAGAGGACTTTCAGTTGACCTTCGACATTAAACCGTCCTTGAGTCCCTGACATATACAAGCTTTAAGATACCCTTAATTTTAAAGGCATACTTTTTTCTGCCTTTTCTTTCTCTTTCTTTTTCTTGTGATACCCAAGTTTCTTTGATACCCCAAAACATTTTTCACAATGTCGGACGCACAGAAGCCCGTTGAGGTCCCGAAGGACGCCGCCGCTGCCCCGGCAGTTGAGCCAATTGTTGCTGCCCCCGAGGCGACCCCCGCTGTTGTCGAGGCCCCCAAAGAGGCCGAGCCCGTTGTCGCTGCCACCACTGCCGAGCCCGAGACCGCCGCTACGACGACCGCCGAGACCCCCGCCGCCGCTGCTACGACTGAGGCTGCTACCGAGACCGCGGCTGCCGAGACCGAGGGGGCTAAGAAGGAGGAGAAGGAGGTTACCCCCATCGAGGAGGGCTACCTTGAGTCAAAGGGCACCAAGTTTCCTCAGTATGTTTGACCAGGAGAACGCCGACAAGATTTGTAGCGCCATTACTAACCTATGAATAGGAACTTCATCTACACCAAGCGCTTCTTCTGGTTCGGTACTGATGCCGTCGAGCCCAAGAACTTGGCCTCTTTCCTCAAGTCAGAGAAGTCAACTGAGAGCGCGCAGAACATTGCCGCATGGGCTAGCCACACTGGCGAGGGTCTCCTTTTCTTCACCGAGAAGGGCACTGACAAGACCGCTCCTGGTGGCGCCATCCAACTGGTAAGTCACACCTGACGCGATCAAGTATGAAGAAAAAACTGGTCCAATGTTCTTGGCACTGACCCGCTTCCGACCCATGCAGAGCGAGGCTTCTGAGCCAACTGCCGACGGCCCCACTAAGTTCCACTTTACTGCCAAGGGCCACAAGCACACGTTCAAGGCTGCTACTGCTGCCGACCGCGACAACTGGGTCGCCCAAATCAAGGCCAAGTCCGCTGCCGCCAAGGAGCTCGCCGCTTCTGTGACTGAGAGCGAGACCTACAAGAAGACCTTGGAGAGTTTCAAGCCTGCTGCCCCTGTCGCTCCCGCCAAGAAGGGGGAGGCTAAGGCTGAGGAGCCTGCCGCTGAGGTTGCCGCCGCTACCGAGGCCGAGCCTGCCACTGAGGCCAAGGAGACTGAGGTCCCCAAGATTGAGGAGCCCGCCGAGGCCAAGGAGGCTGCTGCCGCCAAGGACGAGAAGAAGGAGGAG

**> MD209_chr02:2963943-2967943**

CGGCAGCCACGTTACAGTGCTTGTGCGCAAGGACAAGCTGCGCGCCAGCAGCATCATGGCCAAGCGCTTGCTGTCGCACCCCAAGGTTACAGTCAAGTTCAATTCGGTTGGAGTCGAGGTCAAGGGTGGGGAGGACGGTCTTATGAGCCACATGGTCATCAAGGACGTCGTTACTGGCAAGGAGGAGACGCTCGAAGCCAACGGTCTATTTTATGCCATCGGACACGACCCGGCCACTCAGCTCGTCAAGGGGCAGCTCGAGACCGATGAGGAGGGTTATATCGTCACTAAGCCGGGTACCCCCCTGACGAGTGTCGAGGGTGTTTTTGCTTGCGGCGATGTGCAAGACAAGAGATACAGACAGGCTATTACAAGTGCCGGTAAGTTTTCCGCCCTGCCGACCTTGCTCTCCTAGTGAAACATCAAAGCACATGACAGGTCAGTCAGGATCAGACTAACTCTTAGACTCATAGGTTCCGGTTGCATGGCCGCCATGGATGCTGAAAAGTTCCTGGCTGAGCAAGAGGACGTCGAGCCCGATCGAGAGGCGCGCATGTAAGCGTGTCACATATTTTGATACTTCTTATATCTCAAATCGGCCAGCGTCTGCCGAGGCTTGTGATTGCGTTCCGAGAACGATTCAGCGATTTATACGGGGCTAAACTTTCAACTGGCTGTTTGGACTATGATAGATGGAAGAAGCTAAAGAAAATAAATATATACATATGTATAGATTCTATAGAGAAGGCACACCCTCAGGGGTGTGTTGCGTGCATCTAATCGTCCGTTTGTGCCAGCATGGGTATATTCGGTACACCATCCACTATCTGTAATATCTAACCACTGGAGCAACCCCGCTCGACTGCTTAGAAAAGTGGTGAGGCTTCTGGAGACGTCCTGTGGCCCTTTTTAACAGCCTCTTCACACCAAAGTGGTGAGAACCCGGTTTCACAAGGCTTGTGTGGAGTTACAGGGCAGCCTTGCAGAGTCTCGGAGCAAATCTACAGAAAATGCCTATATTTGACTGAGAAGTCCGATTCTAGTCAGCCAAGCTCAAGCTACCCGTAAGTGGATGCTCACCATCGAGGTACACGGGAACATCAGTGCTCGCATGTATATGCTAGTCTTGGCAAGGCTTAAGCTTGGCTTCTAACACCTTAGTTCTGGCTATGTATGGCGAGAGGGATAACGCTGTCCGCATCACATTAACTTTTCAAAAGTGGGCCTAAAGGCATTGTCGCCTGTGTGTCGTTGACAAAGCTCACGATATCAGGTGCTGGAGAGCAAGCACAAGCTTAGTTTCATATGCTGATGTGCCATGCCGAAAGGCTACGTTGCTCAACCTGGCCCTTCTTTTCAGTATCCGGTATCAAGACTCGACTGCTTTGTACCATTTCCATTCCAACAAAACAGCGGCTTAAAGGCACAAGTCCACCCACACCACATCCGCAGCGTCAGCAACAGCCCTATTCCAGACGATCATAAAGCTGTGCGACTCAGTTCTCCCGGCGCAAGAAGGTTTTTTGAAGGCGAATATGCCGTCTTTGCATTGCATCGAGCGGTCAAAAAGCGCTAGACTAGTATGAACCATGTACAAATGCAGTTATAACTTATCCCCTTTCTACATGACGCTAAAAACACACACTCGCTACGTTCCGCGCCGGGACCGGCTTACGGGCCACCAAAGGCGGCCTTTGTCGAGGGGGAGGGGCCTCTGCTGCTTAATTGCAGCCATCTTTTTTGACGTATCCCAAGACATACAGTACATAAGCATGACGGGTCAGCGCTGTCCAACCCTCACTGCTCAATACGGCCTTCCACCTGGCCGACCATGCACCTAAATACGGCAAGAAACAGGAAGAGGAAGAAGAAGAAAAAGGAGAGAGAAAACAACGAGTAAGAGGTTGGCAAGGGTTGGTAAGCGTGAAAAGATATCGGCAGGCCGTTGCA

GCAACAAGGCTGACCACCCACCCACCCACTCGGCTGCACATACTCATACCCACACACACACACACACACACACAC

ACACACACACACACACACACACACACCCCAAAGCCAACAAGGGTGTGACGAACAACCGTTTATCAGGTACCTGGTCTTGTCGTGTCTCTTTGACATTACGATTAATCTAAAACAAATGAGCAATGTGCCCATGCATCATGCAGGTTGCTGACGTCTCGGGATCAATAAATGGCACATCACAACCACGCCGAAAACCAAACGAATGAAAAGGCTTCGATCGAAGGCCGACAGGGCACACTGGAAAATCAAATCTCACAATCGTCCATCCAGACCGTGTTGGTCATTTTGCCCTCTTTGATTTGATCTATCATCAAACTGGCGTAGATCAACATTTTTCAATGCAACCAAAGACGTGGAGGGTGACAGGTAATCGAAGGAAACAACGCAGGTGTGAGGCGAGGTAGCGGCCTTGGCGGCACAGTAAAATATGCGAATGGGATAAGCATGGCATGTAGTATAATGTGTCTCTGTTCAAATGTATCATGATTTATGTCTGAATCTTTCGATATGTCTCGTCGATGATATGTCGAGGGGTGTCCGCTGGACAAGCCCAACCAAGAACGAACGCCTTGAACCATGCTTTCGCTGCATATAACCTATATGCCCAAAGTAAGGTAACGTATGTATCGTAATGATAAAATGTCCAGCGCTTCATAGCTCCGTTTTGCCTCTTTTGTCTCATTAAAAAAGTAACTCAAAAGCAAAAACAATAAATTCATCATCCCTCCGTGATTTCGAGGTCCAAATTTCCCACCCAACGTTTCGGGGCATATCAGACAAAGGAAACAAGATGATAGAAAAAAAAAGGTGAGATAAAAACAACAACAAGCGGTCATACAGATCGTGAAGAAAAGCAGATCATAAAAAGGTGTGATGCTAAAGAGTGTCGGTAAAGTTGGGTGCAGATAACATGTGGTCATGAAGTTGAGGTGCTCCAGGCGCATTGATTTGTGTCGTGAGTTCTCGTAGCGTCTTTGTGTCGTACGTGTGGTGGCCGACAGTGCCGCCGTCATTTCCAGTCGAAGGTGAAAGGCAAATAAAAATAATAATATGCGGGAAGGGAGAAGGAAGAGACGCCGTCGCTCAATGATCCAATCCAACCGAATGTAACTGACAAAGGAAATCAAATAAACAATCTAAGCCAAATGTAAACTGCCGATCATCAATGGATCGTACAGGTCTCAGATGGCAAGCAAGACATGAACAGAATGGTATCATGTAACACCAGCAAGGGCTGGTGTCGACACTGATAATCGGACAAGTGTCTCAATTGGCACGAATGACCAACTTGGAAAGGAACTTGTTGGATAGAGAGTCGAAGTCGTAGCGGTCGAACTTGGCATCGGCACGGCCCCTCAGGTATTGTTCTGTTGAGGTATGTTAGCGTTTTGATCTTTGCATGTGAAATACCTAACACGGCTGGAACAAAAAGATCAAGTTGGGCTATAGGATACATACCATGCTTGGTCACATGTTGGCGCACTTCTGTCATGGCCTCACACACAGCCGGGTTGTTGTTGAGCAGCTGGCCATGCCTGCGTGTGTCCCACTCGGTGAATGCGGGAAGCTTCTGGGAGATGGCGTGGAAAAGACCGACAATGACACCAACGGGCGTCGACTCCATGTCGTTGCAGAATTTGTTGACGGCGATGGGGTCGGGTACGCGGTAGTGGCAGTCGAACTGCATCTCCTCCCAAACACACAGAAGAATGGCGGCGAGCATGAAAATGGTCGGCCAGTTCTTGAGCCTGTCGCCGCTGTAAACGCTCGAGTAGAGAGCAGATAACTCCTCGAGGATCTCCTTTTGCAGCTCGCGCCACATGTCGGCCATGGCACACTTGATCTGGAAGTTGATCATGACGGGAGCGACGATCTTGCCAAAGTACTTGGAATCCTCGTCATCAATCTGACCCTCGGCAAAGTTCTCGGCACCCTGCTGCTCCACCATGGTGATGTGAAGCGTCAGGTTGTAGGC

**> MD210­_chr02:3234948-3238948**

GGGCCTGCCATTTTGAAAAAGTCCAGCGCGTCGATGACGCACGTAACGGTCAGCTTCTCAAGCTGCCTCGCCGCCAGCGCTGCCAGGGCGGCCGCCAGATCCCCTCGCTTGTGCGACTCGTAAGTCTGCTGGTTGCCCTGGGCTCCCCAGAACTGGCCGAGTCCGACCTGGCGGAGGCAGAGGCTCCTGATAGTGGCGGGCAGCTGCGCGACGGCGGCGATGTGGTCGTCGATGAGCTTGTCGTGCTTCTCCGTCGAGCAGAAGCGGGCTTTGTACTGCCAGTCGATGTATTCGATGGAGGAGAGTTGTTTGGCCAGCTTGTTGACCACGTAGGGGCTGATGTACCGGACGTTGTCATAGTTGAGCGAGATCCCAGAGATAAAATCGGCCGTCAGTGACTCGAATGTGTTGCAAGGCAGCCCGAGAGCATCCAAGTCAACCTGCCAGGTGTGGTCCCATTCTGCCTGCCTGTCAAAGTCCCTTGAGGCACTCCTGTATAAAAATGCGCGGTAATCTTCGTCTATGGTGTTGAGGTAACTGTTGTCCAGGACATCTCCGTCGTACTCCACGAATCGGCATGCCAGCTCTTGGCAGTAGCTCTTGGTCTCGGCAATCAGCTCGAGGCAGATGCCGTCGTGGGAGACCTCGGATCTTTCCCAGCGACTAGTCAAGCTTATGAACCAGTATATCATATTAGTGACCTCATAGTTGATTCGCTTTGTGTTCCATGCGGCATTGTCCTCGGCGTTTGTTGTTTGCCTGTTCAGCTCCCGATGCAGACGGGCCAGGTTTCTGTCGCAAATGCTGGCGGTCCAGACACTATTGTCCACGGACTCGACTGAGTCTATGAACAGGAACTCAAGACCAATGTACCGAATGTATCGACGGCGACGGACGTCCAAGAAGAGCTGCTCAAACGTGATCAGTTCGTCTTTGGAAAACCTGAGCTCTCTCATGGTTATCCGCTCGACGATCACCTGCCATGCTTTGCTGACTGTGGCGTAGCCTGCCACCGTCTTGTCCTCGCCATGTTCTTCCTCGACGACCTACGTCGCGATCATGCCCTGGAGCTCCCCGGGCAGGTTGCGCCACAAGGTCTCGCTGATTTGGGTATCCATGCCTAGGTATATTTATAGCATGATTAGAATGGGATGACGGAAGGGTGAGCAAGAAGTGAGAGAGACAAAAAAAGCAGATATTCTCGAGTTGAAAGAGGAAGCAATTTTGTGTGACACTCTATAGCCGTCCATTCCGGGCAACCTATCAGCCCTAACTCACCTAGATATGCAGTCGGTTGAGGTTGCTAGATGGGCTATGCCTACTGTTAGATTGATTATTCGGAAGGAGTGGGTCTTTGTAGGCACCTACTCACCACGCCACTCTCGCTCCGCTGACGCCTTCCGATATAGCGAAGCTGCCACTGTAGGGTATTTCAGCCTATCCCGGTCAATCAACAAAGTAAGGAGTATATTGATTCCGTATTTGTATGGTACAGGTGCTTCGTTGATTCAGTTGCCTGTTTGACGGGTCTCTAGTCCATTCAATACATGTAAAAGTTCTGCCGCGAGGATGTGCAGTGCAGCGCATAAATAAAGTGTACAAGAGAAGCCACAAAACCCCGGAATCCAGCCTCTTTCAAAACAGTGACAAACGGGCTAGTGTGGCGAACTTTCAGAGGGACGTGACTTGACAAAACGCGTTGCGTAGCTCGGGAAACAAACAGACTCGGCAGATGCACGCGTTGGCTGTTGAGATACGCTGAGTAAACAACCAGGTACCCTGTTCAAGCCTTCCAAAGCTCTATTAAAAAATAGCACGAATGGCATTTTTTTCAGGTGTGCAAAACTATAACTTCCTAATATGAAGCTGTTCATGGTAGTTTCCAAGGCGGCGGGTGAAGTTTCTATGCGCTGCCTGTTCTGTCCCACCATCAGGCCACTGGCGTGGACC

CGAAGAGAGCAAGCCTGCAGAGTCGTCATCCACAGATTCTTAGCTGGTTCCCTACCTACCTACCTACCTACCTACCTACCTACCTACCTAGGTACCTACTCAGTCTCATCAAGGCTTCCCAAATTTCTACCACCAGAACAATCACACCGATTGATAGAAACAGACTAGACTAGTTCTAGTCCCAGTTCAAATTCTCCTTCTATGAGAGCAACTTCAAGCTCCCCT

TGATGGGAAAAAAAGAAAGTTTGGTCTCTGGTAAACTCCCCTATTCTCCAGACAGGAGAGATTACCAAGCAACAAATACGACACATCGTCATATGCCTGATAAGAAATTTGGCCAGGAGCAATCGATTCAATGAAAATGACATTTATCCGTGTCTATTACGCAAATTTATCTAGGCTCCGATTGCACAGCCGCGTGGTTTTCGATCGCATGGTTCCGTCAACTTTGCGGCTGTATGTTCATATCCGGGAGAATATGCTTTCCCCTGTTCGGGAAGGCCCTTGGACTCTTGTGATAGGCCAGCCAAGAGCGTCAATTGTCCCGATTCAAGATGTGCATGCCGTTGACACCGGCAGTTGCTCTTTTATCCCGCTGGTAGGGGCGGACGACGAATCGGGTTGTCTTTTGTTATTAGACTTGTTCCAGTAGTCGTCATGATGGCCCCAACATATGCCAAGTCCTGTGTATGGCTGGAGCCATAAATATAGACTTTTCCGGGCTGTTTTGTTGCTCGCTTTTGTGAGGGCTGCAGCTCGTAACATTGCATTCGGCTACGCCATCCAGAAACAACCCTACAGACACCGACAGAACTGACACAATGCTTTGGCTGGGTGCTCTCAGTCTTGCCACCGCCGTGCGCGGTCAGGCCTTTAGCTTCACCATGCTACGCTTTGGATGCTCCGAGGTTGTTATTGACCGCATCGATCCTCTCGTTGACCCGGGCCAGGTCCCATCGCCGCATCTGCATCAGGTCGTCGGAGGTGTAAGTATTCTTATACACAGGTACAAGAAAAAGACAGAAGAAAAGCGGCCCACAAAGATATTAACGACCGTGTATTACACAAACAGAACGCATTCAACGCATCCATGCCGTACGAGGACATCTCTGGCATCGCCAGCTGCACGACCTGCACGTTCGACCAGGACTTTTCAAACTACTGGACCGCGAACTTGTACTTCAAGGCCCGCAATGGCAGCTACCACCGCGTGCCACAGATAGCCAACGAGGTCATCGGGGACGCCAACGGAGGCATCACCGTCTACTATACGGCTCCGGGCCCCAACACCGTGACCGCCTTTAAGCCGGTGAGTTTTTTTTATCTGCTGTGATGAAAAAGGGGGGTAAAAACTTCTGACTCGATTCATTCCACTCCGTGTTACAAGGGCTTCCGTATGCTGTCGGGAGACACCAACAGCCGCGAGTCCAAAGGCGAGGGCAAAAACATGCAGAGCTGTTTCCGCTGCTACGACGGACCCAACTTTGCTGGAAACATCTACTCGCCCTGCTTCGACCCGGTCCGAGACACCGAGGGTCTGCCTTCTAAGCCCTGCCTGGGTGGTATTAGGTCCAGTGTCATTTTCCCCATGTGAGTCTTTTTTTTTTCTTTTTTTTGGGTAATGAACTCAGCTGTTTATCCGGATGAAAATTTGGCTAACAAACAGTTTCTTTGTAGCTGCTGGGATGGAAAGAACCTCGACAGCCCCAATCACAAAGACCACGTTGCTCACCCCGTGGACGGGCCGGCTTCTTTTGCTCTTGTGGATGGCCAATGCCCCGAGTCGCACCCGGTCAAGATCCCGCAGGTGAGTATTTGTGTTTTTGGTCGAGATGCCAAAGGGAGAGAATAGTCGCAAAAGCAGTGCTTACTGTCCCTAAAATTGACTAGGTTCACTACGAGATTGTCTGGGATACGACTCAATTTAACGACGCATCTCAATGGCCCGAGGATGGCTCCCAACCTTTTGTGCTTAGTAACGGCGACAATTGAGTACACCTTTTGCTTCCATCCAATGACACGACGGAAAGAGAAAAAAAAAAAAAACTGA

**> MD211_chr02:3273446-3277446**

TATTGCGTTGATTAGATTTGAAATCTTTCTTTCTTCGATGGAACAGGAAATAGGTGCGCCAAGACGACCGCGCTTCAACCTTGAAGAACCGTGGATACTTATGGATCTTGCTCCTTAATATGCAGATCACCATGGTGTGCTTGCTGTGTGAACCCACCCAGCTTATTGTTCCGGATCTGCGATCAAGGCGCCGATAACCACGGGCAGTCTGAACGTTTTTTGTTTTTTTCCTTTTCTTTCTTTTTTTCCCCTTCTTCATTCAGACAAACCCTTGGCGTCACACCCACTGAGTCTTTTTTGGTCCTGCAGATCCATCACGATTCAACACAGCACCTCTGTCTGGTAAGTTGTCGCTTATCGAGCAGGACAGTGTTTTTTTTTCATTCGTTCCTGGTCAAGGTCTGTCAACTTTATCCACCGTCAAAGATCTTGCGGGGCCCGGGGGGGGGGGGGGGGGTGCACTACAGAGAAGGGCGAGGCTAGGCGAGCATGTGTTTGTCACAGAGAGGCGGGACCCCCCTCCCCGTCGATTTAGACAAACACATTTAGAGCCAAACTCGGAGAATAGTGATCAACACGTGGATGTTCGTTTGTTGAAGATTATACAAATCCGTTGTATGAGCAGACGTAATTTCTTACGTGCGTACCCTTTCTTTTTTTTCTTTTTTTTTCTCTCTCTCTTTACTTTTTCGTTTGAGGTGAACCTGGAATTCATGGATCCTTTGGCGATCCGGATGGCTCCATATTCTCATCGCACGGTGGTGGTCAATGCGGTTTTTTTTTTTTTTTTTTTTTTTTGGAGATATGCTCTCGACGACATGATCTAATAGACTTGAGAGAGAAAGAAAAAAAGAGAACAGGGCACCATCCGACAGGAAAAAGCAACATGGAGGATATTCTTCCGTGGCGGAAAGCCTCTCCCGGGCCGTCGAAGAGGAGGGGTGTGTGAGTTTCTTTTCTCTATATGGCCGTTGTTCTTTTCTTTTTCTTTTTTTTATCTGTCTTCTTGTCAAGTCTGTTTATTCCCTTTTTCTTTCCCATTGTCTCGTTTGTGCTGGGCAGGGCAGCTCTTACTATGACAAGTATTTCCCCATCTGGCCCATGCGCACCGGTATATTCCACCCAAAGGTACCGTGCTTGGCTGCCGTGGAGGTAGGGATGGAGGTTGTATTGGTGTGTACATCTGAAAGTGGGCCCGGCCCCTTACAAGGCCATGGACAAGGCCATGGACAAGATCGCTTTGGACCGTTACATAGTCTTTTTGTTGCCAGGGTCGCGATATTGCCTTGGATGCTCCCTCCCAGCAAGGAAATTGTGCCATCAGAAAGGGCTGGCCGGCCTTGTTGCCCAAACGTTCGGGGTGTTCCGTCATGACGCGCCTAGGATGATGGCCTCCATTCTTTTTTTTTATTTTCTTTTATCCTTTTTACTTCTTGGTCTGCTCATGGTTGTTTCGCGACGACGCTTTCCCCGAGGGTGTGGGCTTCGGTGCCAGGATACCGCCAACTGTCACCCCCTTTCCTCGTCCTTGTTGCACCCATATAGATTCTGAGAAATCACCTTGTATAAATATATATAAGGTTCCCAGTCGTCCCCTCCGAATGGCAGGTTGTGTTTACTCGGTCAAGGTTGACAAGGGTTTTCATGGGATATCACTCTACACATACTACATTACTTTATACGATACCCTCCCCAGCTCTTTTAATCCTAGGATATCTCCTCCTTTGTACACGGCTCAACCTTGCTGGGCGCCAACCGCTAGTCGGTCTCGTTCCATTGATCCCCAGGATTTCACGATAA

ACCACTACACCGCAGTACTACCTGCTCTTTACACCACCTCGTTTATTTTATCTCTTCTATACCCTGCAGGCAAGGCCAAACCATATATACATGACACAGACATAGGCGCTTGCTTGTTGATATTATTCACACCTGGATGATGCAGCATCTACATGAGACGGCAACAGTCCCCCTTTCGACCCACACTCATACTCACTCGCCCACAACCACAACCACAACCACAAC

CACAACCACAACCACAGCCGCACACAACATGTCCCAGGACTACAGAACATCAATGATGGCGCCGCAGTCGCCGGCGGTGGCGCGCAACTGCCTGCCGCTCCGGTTCAGCGACGGGCCGAGGCAGGCGGTGCCGTTCTCGACGGTGACGGCGGCGTTTTTCCACCAGGCGCGGACGCAGCCGTCGCGGCCGGCGGCGCGGGACCTGACGTCCAAGACGAGCGCGGCGGGGCGCGAGGTGACGTACGCGACGCTGGCGCGGCGGGCGCGGCTGCTGGCGGCCAAGCTGCGGCGGCTGGGCGTGCAGCCGGGCGACCGCGTGCCGCTGGTGGTGAAGCGCGGCGTCGAGATGCTGGTCGGCATCGTGGCGGTGCTGGCGTGCGGCGCGCAGTACGTGCCGCTCGACGGCGGCGTCGTCCCCGACTCGACGCTCAGGGTCGTCCTGCGCCAGGCCGGCGAGCGGCTGGCCCTGGTCCTGCCGTCCACCGAGTACCGCGTCCGCGCCGTCAGCGTCGACTGCGTCACCGTCGTGATCCCCGTCGTCGAGGGCGCCGAGGCCGAGGCCGAGGCTGAGAGGGAGAGGCTCGGCGAGTACGACAAGGCGGCGGCCGAGGAGGGGTTTGTCGACCTGGCCAGGCCGGAGCTTGGGTGTTATGTTATTTACACCTCTGGTGAGTTTTTTTTCTTGTTTCTTGTGAGATGCGTTCATGTAAGTGAGCAAGAGCTGACCTGTTGTGTGTTTTTTTTCGGGTTTTAGGCACTACTGGTGAGCCAAAGGGGGTTGATGTGATGCACAGCAACGTCACCAACCTTCTCTGTCTCGGCCCTGGAAACCTTGGCATCAAGCCCGGGATGTGCGTTGGGCAGGTCCTGAACATCTCATTTGATATGGGTAAGTACAAACTTATGAAGGGACTCATGGCTTATGAGAGTGGTTGAAAGGTTTTGTTTTGACTGACATTTTTTTTTCTTATCCATTTAGCCGCTTGGGAGATTCTTGGATGCCTGTGCAATGGAGGCACTCTTGTTCTCCGAGGGTCTGACTGGCTGCCGGCCATCAAAGAGGTGAGTTGTCTCGAGGCCAGAGCTCCTAAATACTTGATGTCTGGGGCGTTATTAACGACTTGTCTGATGTTGTTCAGATTGATGTCCTCATCTGCACTCCCAGCATTCTCGCAAAGTACAACCCTGCAGAATTCCCTAGGATCAGGACGGTGGCTACTGCCGGAGAGCCCAGCAACCAGAGGTGAGGACAAGAAACAAGCCCCTTTTTGAGAGAAAAAAAAATGCATCAGGATTCTAACATGCATCACAGGCTGGCTGATCTCTGGGCAGCCCACGGCACTTACTACAACTGCTGCGGCCCCACAGAAACGACAATCGTCAACACCATGTCCAAGCACGTACCCGGTGCTCCCCTTTCCATCGGAGCACCCACTCCCAACAACTCGGTCTACATCCTGGATGAGCAGCTCAACCCCGTGCCTTTTGGCGAGCCTGGAGTGATGTGGGCTGGAGGTGCCGGCGTCTCCCGTGGATACATTGGTCTCCCTGAAAAGACGCAGGAGAAGTACCTGCCGGATCCGTTTGCCAACGATGGGTGAGTTGTTTTCGACTTTTCGACTTTTCGACTTTTGGGCATGCTTCTGATGCAGACACACAGCTGACTCTTGGCATAGGACACATGTGTACAACACTGGCGATCTGGGCAAGTGGAACGAAGACGGAAGCATTGACATTCTGGGACGAGTGGATGATCAGATCAAGATCAAGGTGAGTGCTTCAAGTTTTGTTTTGTTTTGTTTTGTCGCTAGACCGCAACACTGACAACAACTCATCTGTAGGGATTCCGCGTCGAGCTCGACGGCGTCTCCGCCTCGCTCTCATCCGGCCCCGGCATCACAGGTGCTGTCGCTCTCTTTATCGACAATGAGATCCACGGCTTCGTGTCGCCGCGGGTTTGCGACTTGGATGCGCT

**> MD212_chr02:3862621-3866621**

CCGTCATGGACATGTACGGCTTCGGGCTCGACCTCCCCTACCCGATGATGGTGTGGACCGTCTCGTCCTTTTGCGGCCCTGCCCTGGGCCCTCTGCTCTCGGGCTTCAGTGTCCCCGCCCTGGGTTGGCGCTGGTCCCTCCTCGAGATCCTCTTCGCCTCTGCACCCGTCTTCCTTATCATGTTCGCCTTCCTTCCCGAGACTTCGAGCGACAACATCCTGCTCCGCCGCGCCCGACGCCTTCGTGCTCTCACAAAGTCGGACCGCTTCCAGACCAAGGCCGAGATCGAGCAGAAGGAAATGACGTTTGGCGCCATCCTCGCCGATGCGCTCATCAAGCCCATGGAGATCTCCATCAAGGACCCGGCCGTGGCTTTTGTCCAGATCTACACCGCCATCATCTACGGAATCTATTACTCCTTCTTCGAGGTGTTCCCGCTCGTTTTCCCCGTCTTCTACGGCATGAGCGCGGGACAGGTCGGCCTCGTCTTCCTCTGCATCCTCGTGTCGGCGGTCGTGGGGATGACCGCCTATGCCTCCTACCTGGCCTTTTACATGCACCCGCGCATCCGCGCCCGCGGTCACCCGGTGCATGAAACGCGCCTGGTACCCGCCCTGGCCGCCGCTTTCGGCCCGACCATCGGCCTCTTCATCTTTGCCTGGACGGCCCGACCAGACGTGCACTGGATCGCGCCCACCGTGGGCATTACCCTGTACGGCGCCTCGGGCTTCGTCGTCATGCAGTGCCTGTTTTGCTACATCCCGCTGTCGTACCCGCGCTACGCCGCGAGCTTGTTTGCCGCAAACGACCTCTGCCGGTCGGCCCTGGCTTTTGCCAGCGTCCTGTTTGCCAGGCCGCTCTTTGCAAACCTCGGCGTCGCCCGCGGCGTCACCCTCCTCGCCGGCCTGAGCGTACTCGGAATCGTTGGCATGTGGGCCCTGTGGAGTTTCGGCGCCCGGTTGAGGGCCATGAGCAAGTTTGCCGTGTCCGAGTCGGAAGAGATTGTCGCTGCCGAGCAGGAGAAGCAGTGAATGTTTGATGAAAAAAAGTGATTGTTTCTTGGGTTTGACTTTATGGCTGGGTTATAATGCTAAGGAGTTTAGTGCAGAGGAGTTTTTCTCATCTCCTGTTACCAGTGTCCGCGTTTGATTAGGCCCTGCGACGACATAAAGAATTCGTGCAAAAACGAATTAGACTCTAGTTCTAGACTAGCTTAGATTCGGATCTTTTGCTTCCAAATGGTATTTTTTTCAGCACAGTTCATGTGTATTTCTTGGTTCGAGTCCACGGCATCACAGCATTGATTCCGAGTATGTATGCAGGAAAAAAGACCGAGGGTGGGACGGACTTGAAATAGAAGTCGTAAGTTATCATATGCTAGGGTATTTATTCACGTACCAACGTCACATTTGTGACCCCACTTCGCAGAGCTCAAGGGGCCCCAAATACCAGAGAGAAAGATATAAAATAAACAGTGGTGAGGCACCAAGCACCCCACGTCAGAAGATGAAGTCAACAAACGGCAAAAGTGTCAGTGCCATGGTTTGAATAACCACTGACCCGACACATCAGTGTAATGGGTATCATTTGATCAGAAACTTCAATAGTAAAAAAAAAAAAAAAAACAGAACGTAATGTTCCGTGTGAAACATATGACAATCAGCGGCCGTGGCTGTTTTGCATAAGATTGTAAAGTGTGTCGTTTGCATCCATGTTTCCATAGTTATTGCCCGTCATGTTCATCGACCTGTGCTGAGAGCCGTGTGATGTTGAGCCTCCGACGTGACTAGTGTCGTAGCC

GCCGTTCACGGCTTGTTGAGGATCGACGACACCTCTGTATCCGGCTGGATTGCTGTTTGTCGCTGCGTATCCATACCATCCTTGCTGCTGCTGCGGGTGCTGGCTGGAGGTCGACTGTGGCTGCCGCCCATACGATGCATAACCCTGCTGCTGTTGCTGCTGCGACTGTTGTGTCTGTGGCTGCGAGGGACTCTGCTGGCGCTGCTGCTGCTGCTGCTGCTGCTGCTGCTGCTGCTGCTGATGGTACGATGCACGAACCGAGCTTCTCGTGGCATGTGGCGAGGTGGGCCGCACGCTGAA

ACCATGTATGGAACTCGGCTGTGACATGGGCGCTGTCTGGTCTGTACGGCTTGCTGTGGCTGTTGCCGAATTGTTGTTGTAAGTCTTTGAGTTGTGGACCTGTGGCGCGGTAGCAGACGGCCATTGGTTGTTGCTATTGCTGGCTGAAGACTTATATGCAGTCGAGGTGGGCCGTGTAACGGGATTGCGGTAAGTTTGCGTTGTTTGCGTTGTAGGCCTGTGAGGGGTGGCATTGTGAGTGCTGTAATTGTCGTATGATGGGTAAGCCGGTGCGTTATTGGAGGCAGGTTGGCTGGTATATGAGCTTTGACCGGTCCCATTGCCAGTGCTGCCGCCAGCTTGACCAGTGCTTGTTGATGCGAATGTATTATTGTCGTTGCTGCTGCTACTCCCATAGTATTGAGCGTGGGGATCCTGCGCAGCGGTACCCACGGAGGCACCGACAGAGGAGTAAGAATTAGACATGGTCGGCGGTCCGTAATCAGAAGAGTTACTGATGGCCGCAGACTGGTATTCTCGCGACACCACTGGCCGCTGGCTTTGCCTTGAGCTAGTCCTGGCCGGCTGTAATGGCTGGGTTTGTGGCGAAGCCCGACTTGTCTGTTGCATAGCGGCCTGAGACAAAGCCGCAGCTTCTCGCAAGCCTTGACTGGGTCCCTGTGAGCCAAAATCTGCCGTGTTCATGGATGTCCGGGATCTGCTCCTAGTGTGCACCTGGTACCCATGATTCGCCGTATCCTGTACCTGTACCTGTGCCTGTACCTGTACCGGCTGCGTAGGCGGAGCGGGAGCGGTGTTGGAGAAATTCGCCCATGGGGTACTATCCGGGGCCGGATGGTCTACTACTTTGGGTTGGGTGCGCTGACCTGTGGGAAGGCTCCTGCGACCCGTCCGGGAGATGCCGGCGGCCTGCTGTGCAGAGGCAGCTTCTCGACTTGGCCTTGACGGCTGGACCGGTTCTTCCATCACCGCTTCGGGCACCTGAGCTGTATGACTTGCATGGCTTGCAAAACCGGTGTGCTGGGGATATTCTAAGCTCGGTGCAGTCAAGGTATGCTCTCTAGACTCATCAACTGGTTGGGGCTGGTAGCTCGATACGTTGTCGTCATTGGTCTGTGTCTGGGTCTGAGTCTGAGTATCGGCCCAGTCCGTGGCCGTGGTTTGCTGTCCAAAACCAGGAGAAGGCAGGTCATCTGGCTCTTCCTCCTCCAGTGCCGGAGGCGGTGCTGGAGCTGACGGAGGTATTGGTGCAACGACCGGTTCAGGTTTTGACTCAATATGCTCCTCTGACTTTGCGTTGCCTCTGGGGCCCGTGGGAATTTCTTGGCGATAATGGCACTCAATGTTGGTCATGCGACATTGCTCGCAAGTGGGCTTGGTTTTGGAGCACTACGCATCCATTGTGATTTGTTGATTAGTAAAATGTTCTTCAGACGTTCAATATATGCATGTTGGTTCCTTACCAGAGTCTTGGCGTCTCGACAAGGGTCGCATTTGCCACGGGTTAGCTTCTTCTTCTTCGTCCCACTTGGAGTTGCGTCTTCGCCAGACTCAGCACAAGCCAGAGACTTCTTTGCCGACTTCTTCTTCTTCTTCTTGGTCGTGGAGCTTGCAGTTTCGGCATCTGAGGAGGCTGTGGCGGCCGAGCTATCTGGTGTAGCCGGTGTCGATGGTGTAGGAGTACTTGCTTGTGCGTCCTTATTCGGGTGTACGATCCTCAGTCTCTCGGCACCATATTTGACCAACATAGCCAGAGATTCCTGCTGAAGTTCAGGCGACTGGTTGGAAAATATGTTGAACTGCTCTTCGGACAGTGTGGCAGGATCTATTCCGCTAGTAATGAGTAACCCATTGGCATGGTCACGCCTCCTGTCCGCCTCACCAGGTGGAAGATCGAGCA

**> MD213_chr02:4127283-4131283**

TCATTGTCGTTACGCTCGCGGTGTTGTTGGCCACGGTTGCCGCCTCGCCTTTGGTTGCCACGGTCGTTGTTGTGCTGAGTGCTCGCCTCGACCAACTTGCGCTCAGCCTCCTGCTGTGCATTCGCCTCCTCCCTGCGCAGCGTCTGGAGCAGAGTGCTGACCGTCTTCTTGGTCTTCTCCAGTGAGCTTGCAAGCTGGCGCCACTGCTTCTCGCCGAACACACGGTATCTGACCGAGTGAATCAGGAAGACCTTCTTCTTCTGGGAAAGCTTGCCCTCGACCAAGCCAGCGCGTATAACGTCGATTGCCCACATCTCAACGTCCTCCGAAGGGATCTGGAGCGCCTTGGTGATGGAATTGTACGATATCTCGCGCGTCTGCATGCTTGCTGCTGCCAGCGACGCAAAAGTAAGCAGTCGCATCTTTCGGTAGAGAACCTCATTGTCAAGGTTCTCCTTCTCGATGAAACCCTCGTGCTCCTCCCTGAAGTCATCATAGTCGTCGAGGTCTTGCTCGCCGAAGATCTGCAGAAGCTGATAGTGGACAGGGTGCGACTCGCTAAGAGCGTGGACAGCGGGCAATGCCCGAATGTCCTCGAACGAGAGCCTGGTTGGTGAGGATATCGCGGAGCGGAGGGCGCGGAGAGCAAGCTGCTGGGCCTCCTCGGATGTGTAATCCTCGGCATCCTCGGAGTCGAAAGTACGTAGCGCCTTGAGGATATGTTGATATGCCTCCCTGTACCGTGCCTTTGTTAATACCATTTCGCATTACCAAAAAGTATACCTGAAATATGATTCGGTAGGTTCAAAACTCACTCTTCCTCTCCAGCAGCAGTCATGACCTCAATAACCTCTGAATACATCTTGCGCTGGTCCTCCTCATCCAGCTCCCAGCTCTGCAGCCATCTCGGCAACTGCTTCAAAGCACTCTTCAGGTGGTCTGAGATTGGGATGCTGTGGATCTTGTAGAAACGGATGATCTGGATGAAAACGTTGAACCGCACTGGGTCGTTGGGTTTCAAAAGGTTGAAGATGCTCGTGAGCTCGAAGAGTGCTAATTGGGCGCCGTGCTGGGGCGACGATGTGACGGGCCTCGTTAGGTTTTGGCAGATTGTTGGCAGGTGCTTCTTGGGCTCCTTTGATTGCAGGACCAGATGCACGAGAAGGTTGTAGGCGCCTGTGAACTCCTTCTCCGGGATCGCGTTCAGCTTCTTCGACTCGCTGACAATCTTGGTCAGCGCTTCACTCTCCTTCTCGCTGTCGAGAAGAGGTTGGATCTCGTCGCTGATCTGGAGCACGCTTGCCATGTCCTGCGCCAGCTCCGCGAAGGAGCCGTCAACGAACACCAGCTGGGGCTGGTATGAAGCCGTCATTTTGTTGACTATGTTCGCAGCGATCGTTTCGATGGCGAGAATGGGTCGATCGATGCGTTGTATACGATTGTTTGTGCAGCTGGGAGTTTTCCCTCCACCGTGATTCTGATCCTTGCCGTGGGGCCTGCTCTGTAATTTCTGAAGTCCTGGCTGGGCGTGTTCCGGAAGCGGGGTTGATATTGGGGACAATAAAATTGAATGTTTCCCAAGTGGACAGACAGCACAGGACTAGGACGCCTACACAGGTGCACCTGGAAGATACTTTTCTTGCGACAAACCGCTGGAAGATGGACAGCAGGGGGCGATGTTAGCCCGAATCAATGCAGCTTTAAAAAAGTAACAAAACCACACCTATCGTAGAAGGTCTCGCCGCCGACAACTGCACCCCTCTTCTCGCCCTCTGTCGTATTTTTTTCTGCCGTAAACGGTGGGAAAATCATGTCACGTGCATCACCGCGGTCCTCATTGAAGGGCAGTTCAGAGGGCGGTGAGTTTTGGACTCTGTCGTATGTGGGGTGCCCGCCCGCCATCGAAACACTCATTAAGAACCAGGGCAACAGGCTAGGCAGGCAGGCAGG

TAGGCAAGTAAGGTGTCTCGACCTACGAAGTAAAATACATACCTACCTGGTACCTACCACCTACCTACCACCTACCTACCACCTACCTACCACCTACCTTAGGCACCTTGGGAGCTATGAGTCAAAATGTTGGGATTAACCACAAATAGG

TACCTAGATTGATAATGAGTAGGTACGCATAATCAGGACCATGATCGACAAGTAGCAATAATACAATAACCGACCTAGTCTGATTCTAGTCGTTGTAATTAGTGTTACACATACTACTTTACAACTGGCATAGAAATGTGAGAGGATCTGCGCAAGATGTCGCGATTGAACACAAGATGGAATCGTCAGGGCGGGCCAACGAAAACTGCTAGTTTGACCAAAAGAATAAAGCAAGCGGTTAATTATAACATAGGTATTTAATGAGGGAGCTATTTCTTCTCCGTCTTGTACCATAGTTATTACCGTTGACGCAATCCCACGGTTATTGAAAGATAATAAAAGAGTATGGCCACCGTGGGAGATGTACTTGCTGTTTGCTTGCTGATGTTTAATGTCTCCTCCCTTGTCTCAGCGACAAACGGTATCATTTACGAAGGAGGTGACAATCTGCTATAGAAACTGAGCAGGCGACCGTGACATTCAGGCTTCCTACGCCGCCTGACCAGGTCGTGCGTCGGAGCTCTTGTTGGTCATGCATCTCAGTCGGCCGCCGGTGTCATCCGAACCGCATGGGCACAAGGCTTGCGCCCCAAGGCCCTTTCAGGTATAGGTTGCACAAGCCAGTCAGGAGGCCAATAGAGCTAGGCTGGAGATCGGTGACGAGATGAAGGATCATACTTGCCTTATTCTGCATACTATAGACGATCCATTTTGAATGATCAGAACAAACCAATGATAGATGTAATGATTCTGGGGGTTTGAGCTGAAACTATAGGGCAGGTGGCGGAATTTGGAGTCATCGAGCAAAGGAAGGTACGCAATCGATTGTAGGTATTGCAATAGCAATTTGTTCCTTTCCAGGCAGCCAACGAGAGGTACCTTACCTAAGGTAGGTAGTCGGTACCTACGGTGGGTGCTTGCCTACTCCAGGTACCTTGACTACCTAGCTACCCCTGCACTCTTCTCGTACTGTGAAGGTACCTAGCTGATCTCCCTAGCTCAGACATGGTTATGGGAGGAAGCCCTGCCTAGGATCGCGTTTGCTCCCCTGACAGATTGAAGTGTTGTCGCCTTTGCGCTCGTCTCACTCGCAGGTCCTCGACCCGCAAAAACCATCGTCAGAGCATACAGCCTTGCCGCCTTGTGCTGCGCTGAACTAGCTAAAACGAGTTTCTTCTAGTCTGACCCAAGGTCACCTGAGGCGAACCATCTTGTCCAGGCCAAGCAGATCGTACCATACCATACCTGCCGGGCCTACCACCTAATAATACATTGCCCATCAGCTAGGCAACATCCGTCTTAAGCAAGTAATCGTTTGCCAACGCACACAAGCAACACACGCAATCGCCTCACCTCGTTGCCTGATAATATGGTTCCACGCGCTGAGGGAAGCCAATTGAAGAGGGCTCGGGTATGTTTCTTTAACGGTGATGGTCAAGATGCCCCGGCGACGTTTGAATCCATTCCAAATTCTAACAGCCTTCTTTCAGCCCTCTACTGCCGATTCGGACGGAGAATCTTTCAAGAAACCTCGCAGGACGGAACGTGCAGCTCACAAGGCGGAGGAATCCACCGCGCAGCCAAAAACCCCGGTGGACACCAAGCAGCAACTGCCGTCCCCCATCACGCATAACCCTACCGAGGGCAGCTTCGATGCCTACAAGGAAGGGACTGTAACACCGCCTGAGGGCCGCCCGAGCCAAGTCGCACATCGCTCGCCAGTCCAGGGCTTCTCGTCACCACCACAGGATACACAAGCAACACAAGCTATTCCCTCGCAGATCGTTGATCCGAAGGCCGCGCTATCCGACGAGGTCGAAGACGAGGTGAAGGAGGGCGTATGGGGCTATCTTTTCCCTCTCGATACCAGATACGGCGGCAAATGTGTCGTTTTGCGGAA

**> MD214_chr02:4392130-4396130**

AATGCCGTTCTCAGAGCGTCTCGCGAGTGAACCAACTCCTGATTTGATGATTCTACGAACCAGCAGAAGCGGTTGTGTGCTTTCACCTCGCGTAGATTTAACCGCATCAATTTGTTTAAGCACAGCTCATTCTTGCTGGATCTTTTGGAACGAAGATTGGCTTTGATCTAGTTCTAGTCTGAATATTGTTTCTATTTCGCTGGTGTAGCAAAAAAAAAAAAAAAAAAAGAGCGAAAAGGCTAATCTTTGAACATAATCATGCTGTAACCAATGCTTCAGATGCAGAGGAAACTAGAACTGGAGAGGAACCCCCTAATTCCAACAAACTGCCATGCCGTCTATCCTTACCCGTGGGAGTTTCAAATAATAGACAAGTCATATCCGTGATATCCTGGAACGTTCGCCATTTTAGTCATCGAAAAAAAACCCCTTGACAAAACATTCATACGATGATCAGCTATCCACCGACCCTTTCCTGTAATCTCAAATCATAACCCGACCCCATTCACCATATCAAGCGCACTCTCGACGGCACTCTCTAGTTCGCCAACCCTTTGCTTCAAGGCCTTGTTCTCGTCTTTGAGCAGACCAACCGTGTCCCATGCCATGCCGGCGTCTTGGCCGCGGCAGTTTGCACAGTTGGCAGAGCCGGTACCGTTATCTACCGGCGATGCAGACGTCACGTTCCTTACGAGTTTTTGAAAAGCCGTCATGGACTGGTGCTTTTGCTGAACTGGTGCGGACCGCTGGGGCAGCATACCTTTGCTGGTCGACGCCTTCGGGGCCGTCTTGGGCGGTACTGGTGGTGGTGCCATAGTGACCGACGAAGGCGATCTCGGCAGGGTGGAGCTAGGGCCGTCAGTCGTGGTGGAACTCCGAGACCGCGAGAAGACGCTCTCGACCTCAGCCATGCTCTCGTCGTCGGTGTCGAAGCTATCAGCACCGCCACTGCTGCGCCAGTTGTTCATCCTGCGCCGGTATTGAAACTCTTCTACCGCCGCGTCGACGTCCAAGTCCTCGACGATGGATCCCTCCGATGCAGGACCGGTTCCGTGCGCCTGCTGGTCGAGACGCACCTGGGGACCCTTTGGAGAAGATACACCGCCGGCTGCCGCAAGGAGTCTCGCCGTCTTTCGACGCTCGGCAGCCAGCTCTTCGAGAAGCTCACGCATTGCACGCTCTTGGTCGGCGGCCTTGTAGGCCATGTCCTCCAGCTGCATCTTGAGGTGTGTGGCGCGGGTAGTCGCCTCCTCCATCTCTTCGAGATCGGCCTCTCGCTCGGCGCTCAGTTCGCGAAGTGCTCTGACCTGTTCCGTATAACCGCTCTGAAGACGGCGGGCGAGAGCCACAGCGTTGACCTGGAGGTTCTTGTAGGCTGCTGGGGAAAAAGTGTCCCCGGCAGCCGGAGCGCCCCCGGAAAACAGTGCCGCCTCCACGTTCAGGCTTGACAGATCATCTCGAGGCTGATTGGGCTTGGCCGACGGGCTCAGTGATGACTGTGTCAGCCTCGATGATATTGCAGACATGCCCGAGGCGATGAAGCTGAATCTTGAAGAAGCAGGTGAGGGTGTCTGCCTCTCCCCCGGAGTCAACGTCCTGTTATTCCGTATGGGCGTCCCGTCCGTTGATACAGATGTGGGTTCATCCCTAGGAACGGCGCTCTCGACGCTTCGTATGCCCAAAGGTGACAACAAGCTGTCTCCCGCAACCCCTTCATCCTGCCCGCTGCGAGATGATACGTCCGAAACCCTCTTCTTCTTCTTCCCATCGCCGGTATAAGGCATCTCGACGGCTAGGATAGGCCGTTGCTGCCTTCGCTCCGGCGAAGTAGCTCGGTTGATGAGGGCCAAAGGCAGCAAGCTCGTACTGGACTGCTGCCTGCGGTGCTGCGGTGCCAACGGTGGTGTCAAGGCCTCTCTGCAGGCGGAGGAGGCGAACCCGCTGTGGTAT

TCTTGTACCGGCTGGTGGCGGCGTTCTTCGGTAGTAGGAGTAGTAGTAGGAGTAGTAGTAGTAGTAGTAGTAGTAGTAGTAGTAGTAGTAGTAGTAGTAGTAGTAGTGCCGGTGGTGGCGATGCTCAGGTCGTCGAGCTGTCGTGGAGGCGTCAAACACGATGGCGCCGAAGCTGTTGGACTATGTGACTGATTCCTTAGACTAGAATTCAGACGTGGTTGAACGCAGCCTGGGTCAGGGCGAGATGACTGGGATATAGGAGATCCTGGTGGTGAGGAGAGAGAGGTACTAGAGGCAACG

CCCCTGGAAGTCGAGCTTAACATCATGCGTTAAGGTTTCGGGGTCAGTATTTCTAGGGCGAGAGTCGGCGCAACGCTGCATGCCTGTTGGAGCCCGCGGGGCCAACCAGGGTAGGATAAGGGCCCGGCCAATTTCGAACTGGCGGTGGGAATCAGGCAAGTGTAAAACGGAACAAAAGGGAACCTTGTCCTCGGGAGAATGTAAACTTGAGGTACTCTGGGCAACAAGGAGTACCGAGTGCTGCAGGCTAGGTTGGGTTGTTACTTAGGTACCACGGACAGCCTAGGCGGGTTTCCAAGAAGTAGTTGCCCCCTTCAGCTGGCAGCGGGAGCCGAAGTGCGCTCGGATGTCCACTCAGCAACTTGTAATGAAACGAAACAAAGCACGCCGAATTTTTTTTTTTTTTTTTTTTTTTTTGTTTCTGTCAAGAGGCGATCATAGCCCGACGGCAGGGTCCAGACGACTAATAGTTCCAATGCGCAAACCGCAGATTGCTCTTAAAGCAAAGAGGCAGGGATGAACTATTACAGTCGGGTGGGTTGTTGGAACGTTGGAATGGTTGCGAAGTAAAAACAAAGCAACTTCCCTGCACCGTGCATGGATGTGTACGTATAACCTGCTCGCAGAAAAGCGTCGGTGCCGCCCGGTACTGTGTTGTTCGTGGCTTGTGATTTGGCAGTCGAGGTAGGTATCCTGTAGGTACCGGAAACTAAAAAAGAAGGCTAGCATCCGAGGTTTTGGGGGAGGATTTTGAACTAAAAATCACAAGTTTCCATGTGTAAAGTGCTTGCAGATGATAAGTAAATCATTCGATTTGCTGCTCATGATCACAAAACGAGCGAGTTAACTCATACATTCACATGAGCAACCAAGTCTGAGTACAAATACAAACAATAACAGGGATGTTTGCTTATGGTGGTGACGCATTTCCTTTGTACACTGTTTGACTGGGTTGTTTACTTTTTTTCTTCTTCGTGCGGGAAGGAGACTGGGGGACGACTCGACTTGGGAATTCCCGTCTCTGCCCTGCAATCGATCCTGGCCTGCAGTCTTGATTTAATAATTACTTGGCCTCCCAGGTTGTCCACCAGCATCCACGCGGTCGTCATTGGCTACATACATGCAGCATTCCACAATCCACATGAACTGGAGGGCTCCGATCTCCGTGGTTCAGGTTAGACAACGCTTGATGGCTGACCAGCGGGGCGTACGGCACTTGGCTAAAGGCTTCAGCCACCCAACGTTTGCATCCAATGCCAGCCACCGACTGCAAGCGAGAGCCGATTCGTGCAACCCCACGAACTGATGCGCACCTTGGCCTGACCCTCTAGAACTAGTTCTAGACTAGAAAGTTGATGCCGCGGATGTACCTACCCAATACCCATACCGGGACGAAAAAGCTCTTCATGGTAAAAAGTTCTGCCTGTCGAGATTTGCTACAGGTTGTAAAATTACGTTAATTGAGTCAAGACGCTCCGAGTGGCCAAATGTCAGCTGCTTGCCCGGTTAGCTTTGTCTCCCGGACTGCATGATGTTGGGAAAGTCAGTCGTACAAGTGGCTATCGGGTAATAAATGACGTTGCTCTGAACTGGGTTAAGTCTGCCACGACAAAGCGCCTGCCTGAGCAAAATATGCATTAGGCACCTCTTTGTACTCCATTTGTTGAGCTCCGATATATCTCAAATTCACCAGCCAACACTATGCCCCTGGAGGTCTAGATGCGTCCGTACATTCGCTTGCTGTTACCCA

**> MD215_chr02:4734781-4738781**

TCAGCTCGCCCATCGTCAGGCCAGGCTTCTGCAGACTCAGCAACCATCCTGAAGCAGCTTAGTTCCAAGGACAAGGAAAGTATCAAGAACAAGTTCACGTCTTTCAACGCCGCCTTTGACGACATGGTAGCACGCCACAAATCTTTCAGCATGGAGCGTGAGGTGCGTCAAATGTTTGCCCGCGACATGCAGCAAATGCTAGAGCCGCTTTACGTCCGCTTCTGGGACCGATATCACGAAGTCGACAAGGGCAAGGGCAAGTATGTCAAATATGACAAGGCTGCCATTGCTGCCGTTTTCGCCAGCCTTTACTGAGCTGTATGCCTCACGCAACCTATACATTCTCCATCTTCCCCACACCACCACAACCACACCAAGCCGTCCCTAAGCTGCATTCGTCCAGCTATCAACATGCCTGTCTCCAAGAGCGGACTTTGCAGGTCAGCATACTTGTTCTAGTAATGTCGAATTTGGGAAATCCATGCATATCGCATGGTTGGGATGATACCAAAACGGTCAGCAGGAGTTTTTGGCGTTGATCAAGTTAGCTTTTTATCGATCTTCAGTTTTCTTTCCGCTGCATGAATCATCAAAGTCTATGTGACCATCAAGACGGTAACGTATGCTGAACCAGGTCAGCGCTGATTTTATTAATATTGCCCTGAGCAAGTTTAACAAGGACAGTAGCTGCACATCATCATATACGGGTGGCCTCGCAGGGACTCTTTCTTGGCTTGATACGGGTGCTTTTGAAGTAAGTAACTACCACAACCCAGTGCCAAGTGCAAAGATTACTAGTGAAATACTTGGACCAAGACGGTTCTCAAGAGCAGACTGCCTCGGCGAACACGCCCCGCACGACCTTTCTGCAGCCATTACTGTAACTCCAGGGGCTTGGTAGTGTCACATCGCCCCGACGAGCAATCAGGATTTATAAGCTGCGGCCGGCCAGGAAAGGCATCGAGAGGCGGTTGAAATTGCACTTTACTGCCCGATATGCGGCTTTCGCTTCCGCATTCTCCGACACGGTTCGGGTTGTCGTCACCCTCCGAGCTATACCTTGTACATGGCCGATGACCCCTTTCGAACGTGACCAACTAGTCTTGGGGTCGGGTGTGACATCGCGACATTTAGAGATACACTAGAGTACCGCGCATTTATCACTTCCCCCGCGTCGAGCAATGCAAGCATGGTGTGGTCGATAGTACCGGTGATTACCGGATACCGATGGGATCATGCTATTGTGTGCGAGCTGTATGAAACGTTTGGGGGAGAGCATCTGCGCCAAGTCCTCGAGACAACACCCCCCATGTGCAGCGTGGTAAGTAATCAATTCCACAATAGCACAGTAAGTAGACATGCTAGATTTCCATGCTAAAAAGAGACATCGACCTGTGACTGGCATAGCCCAAGATCAGAAATCAATCAGAAATCGAGGTCACAACCTACTGGCTGGATGCGGCTCGCCTACCTAGGTAATCACACGCTTACACCACCCCCTCACAGCCTCGCGACATGCCGTCTTAGGCACCCTTATCGGGTCCCCTTGCGATCGCCCCCACGGTGGATGTTGTATTCCGCTCTTCTTAACGTTTTGTTCCTCTTGTTATTTTATTTTTTTGCTTTTTCTCTGCCGCGGTCATGTTCTCCATCGGCACAAGATTCCTTGCCACTCTACCGGTAGAGAAGCCCGAGATGATTTTAAACGATGGCTGGCGTATGTTGATAGGAACAGCAATAGGACTGCAGAACTCGTTAGTTAGCTCTCCACGGCCTTATCTTTCGCCAACAGGGCTACTCATGTCTTCCGACCTGTCATTTCTCACCTAGGTCAAGCGCCATGGACGACTGGCCAACTGGCAAAATGATACTACAAGGGGCCCCATGGGCGGCGGATAATAAACATGAAGCTTCCTTGTGCCACGGGGTTGCATGAAAGCACCACATGGC

GAACCTGTCCTGTACCAAAGGCACAATGCCTGTAAGATACGCGCGCAACCTGGTTGGTTGGTTGGTTGGTTGGTTGGTTGGTTGGTTGGTCGGGTGTAAAGATGTTGCCCATGTCCCCAGCATTGATAGGCAGGATGGGGTATGGCTTAC

TCCGGATGACTGGGTCAGGGCCCAAGGCCCATCCCGTCCCTGCCGCTATTCCCTATTTGCTTCTTTTGTTTCCCCATCATCTCCTTCTTTTCTTCTCTGGAATTGTGGGTGTGTGAAGCGAGGGCTGAAGTCGATGCACGAAAAAAAGCCCTCCTTCCATATCTTATTCTCCTTCCCATGTTGTTATAGTTGCCTTGGCGAGGATGTCAACAATTGAACTTTTTTTGTTGGTAAACCCCTCTTTGCAAGCGAAAACGACATTGTCAGCAGCAGGGTTAAAAAACCCGAGGTGTCACTTTTCACACCCTTGAAGGCTGGGGTTCTTTTTGCTGTATACCTATGTAATATCTGCCTCATGCAAGCCTTCTCACACCCAGGCTATTTAGTGTTACCTAGGCATCGCCGATGCATTTACGAGCAGAGTGCCTTGGCTTCTTGGCCAATTGTTTTGTTGGTCCAAGGAAAGCGACTTTTCCCCCCTTCCCAAGTACATCTCATCTTGAGGCATGGGATTGGTAAACGGCAAGGGTCTATGTTCGCGTCTCGCCGTAACCCCAACAATAATTTGAAGAGAGAGAGAAAAAAGGCAAACTGGCCAAGCCTCTATTTCATTCAGCTTATGTCAGAAAAAGAGACACGCAAGACACACTACACAATTTAAACCAGCCGCCTACATGTAGCTGACGTCCGCCAAGCCACGTACAAGAGCAATTCCTGCGCAAAGCCGTCATTGACTCTGCCGCACAACACACTTTCGTCATAAAAAAATAAAAATAAAAACCGCGCCGTCTTCCGTATCGAACCAAGAGAGAAAATCAGGCAGATAAATGATATGAGGAAAACGAAAACAAAGTACATAGAAAAACTTTCGGATAATGCCCAGGCGCCTCTTGGGAGATGGTGGCCGACATAACCGTGTAAAAGGACGGCCCCCCCCCCCCCCCCCAAGCCAACGAGCTGCCTGCTGCACTGCCGAGGATTTCCAGACGGTGATATTATTATTATTATTATTTCACGCAGGATGAATTCGCTGCAAAGCTTCCCCCATCCTATACCTTGAGAGAAGCATATTGGCTCTTTGGCTTCGGAGGAGATGGCGAGTCCGTTACTGCGAACCACAGCGGGGCTTCAAACCACCTCGCGGGAGCAAATAGAAATCAAACATGTCATAGACATGGGGAACGGGGAAGGAAGGGAGAATGAGCTGGAAAATAGTGTATGTGAGACCATGAACTTCTCCGCATTTGTTGGACCAGATTCAGGTCTTGGGTATGTATGTATGCAAGGCCTTGAATCGGGTTTGCTTCGTGCTGTTGTTGACATGCATACGGCATATTGGCACGCCCTTGGCGCTGTGCAAAAGTATTCGCTGTATGCTCATGTCGGGTTTGCTTGATCCGGAAAAAAGGGGGGCTGCGGTTTGCCCCGAACTCGATATAAACCCCGCTGATGTGATACTGGGCTTGGTGCAGCCTAGAAACGGAAAAGGAACGGATGTTATGTAAGTGGCATGTTCAACCGAGCCGGTTCTACTCTGCACTGTTTCAATGACAAGCCCGCAAGGGAAACTTTTAGGACTTCCTTTGCGGCTAGACTTGACTTCTCAGATTGAGAACTAAGCGAAAGTACTAACAACAAGAATGCTCGCGCCGTCGCCATGTAAAAAAGCTAAAGACAATAGATGATGGAAAAAATGAAGTAACAAAGCTCCTGTTCCTCTGCCAAAAAAGTACGCCTGGGTATCTCCCTCGCTTAAGCGAATGTGTATGAGTCAAATGTATGGGGCGTAACGGAAAAAAAAAGAGAAAAAGAAAAAAACCAGATAACAAAAAGTCCGTGAACGACAGCAGCGAGGCACCATTGGTGAGCCTCAAAAGTTCATTGGCAAACAGGTGCCAAT

**> MD217_chr02:6252110-6256110**

CATCCGAGTCTGAAGGAAGACGGTTCAGGACAAGATCCACATAGTTCGAGAAGAGCTCGGCTCGGACCGCCGGGGGATGGTATTGTTGGAGGCCCCAGAGGTCGCCTTGGGCAAAAGTGACCAAGTCGAAGCGGGAAACAATGCCGAAGCTGGATCCTCCACCACCGCGCAAGGCCCAATAGAGGTCAGGATTCTGCTCGGGCGAGGCTTCCATAACCTCGCCAGAGACCAAGACCACCTCGTAAGTACGCACATTGTCGCATGCCCAACCACGCTTGCCTGAAAAGTATGATAATCCACCTCCCAAGATGAGGCCAGACACTCCAACGTCGCCCATGCGCCCGCCAACCACGGCAAGTCCCATCGGGTCAAGGGTTGCGGCGACTTGGACCCAGCGAGCACCGGGGCCGACGGAGGTCGTTTCCCGATCGGACGAGACTTTCACCTGGCTAAGGCGCGCGAGGTCGATCGTGACGCCACCGGGGAGGTTCGAGCCCAGGTGGGCCGTGTGACCACCGCTTTTGACGGTGAAGGGGGCCGAGATGGAGGCCAGGATCTTGACCATGACGGAAATGTCGCCGGTGCTCTCGGGGTATGCAAAACATTCCGGCGACACATCGGACTGTCGTGTAGACCAGTAGTCGTTGGACACGGCGAACGCTGACGAATTGCGGTACGAAACCTTTCCGGGGAGCGCCCAGGACAGACTCGTGCAGGCATTCAGAGAAGGGATGGGCTCGGCGCTTGATCGAACAGCCTGCGCCAGCAGAGAGGCGAAGGGGTGGATGATTATTCTAGAGACCTTCATCTTGGCTGATACCGAGGCGATCTCATCTACCCACCTGTTTTTTTGAGTTGACAAAGGGATGAGGGGCGGGCGAGAGGGAAAGGGGTCCTGACACCGGGAGAAATGAAGAGCATCGCTTCTATTATATCAACCTTGCAATATGCTTTTTTTCATCTCTTGGTAAGGTGTACCTTAATTAACTTACAAGGTAAATGGTTCGTACTAAGATTATATCGGCAACTGACCGTAAAGTATATACGACCTACCCTGCTCAGCTACTGCAATGCCACCATGGCACTCAATAGGATTGGATCTGCAACGATCAGGGCTCGAGAATCAATGGACTCATGGAGCCATTCGTCTGATGGGGCAAATAAGATGTGAAGTTTAAAAAACAAGGCATCAAATCAAGTGAATGAAAACAATCCAAGGGGTTTCGGCTCCGGGATGGCCAATATCGGCCCCGGTAACCCGAGTCGATGCAGTCCTTACTATTGGTGATATTTGATGATGTTATGCCAAAAAACTTTTATTTTTTTTTATTTTTTTTGCTCTTGGTCCGCCTTCCTACTTACAAAAGGTATATGAATATTTATCACCCCGGCCCGAGATCAATCCCGCGTAAGCTGTCTTGGCTGTATGCGGGGCCTTGGTCTCAAGCTTTGTGCAAGGGACCCCTGATGTAACAAAACCCGAACCCACACTATTCTACCGGTAGAACTAAAATCAGCCGGGTCCGAGCGTTGTAACTGCCCTTGCAAATGTCGCCTTTTGCCTTTCCATCACACCTCACGGACCCGGGGTTGCAGACTGTGGCAACAATTGACCTGTTGACAATTCATACCACTCAGCACTTTCACTGGTCGCCGAGGCTTCTGTCAATTTATTTCACGCGCCCAGAATCAAAGCCTCTCATCACACATTTGAAATTGTGTAGCAACGCGCAGTTTTGTTTTTTGACTGGATTCAGTCGTCGCCAAGATGGACTTGCTCCCTTGGGTGTCATGGCTCTT

TGGCTGGGTTTACTTCACATGCTGGAGCCTTTCATTTTACCCACAGCCAATACTGAATAACCAACGACAGTCGACAACAGGGACCACAATTGACTTTCCACTGTCCAACTGTCTGGGTAAGTTCTTAGTTACACACCCTACCACAATTGGGCCGCCAAGCATGCGTGGCTTGAGCCTTACATCACCTATAGGGCTTATGCGCCTGAGTACCTGCCTGAGTACCTGCCTGAGTACCTGCCTGAGTACCTTGACTTGGGCACCGACATTGCCTATAGGTACCTATATCTGACCATGAAGCTC

TTGGACCACAGGCTTCTTCTCTTACTTTATCTACAACCTTGCTCTGTGCTACTCGCCCATTATCCGCAGCCAGTATGCTGCGCGCAACCATGGGCTTGAGCCAACCACGCGCTTCAACGACGTCGTCTTTTCTCTCCACGGGTTCATCCTCTCTTGTGTCACGACGAGTCAGTACATTCCTGGCTTATGGACCGGGTTTGAACGCGCCCCCGTCAGCCAACGCCCGAGCCGCTGGATGCTGGGCATCATCACCGGCTGCATCGTAGGCGTCGCTGCCGTGTTCCTTTTTGTGGCCGGTAGGGAAGGCCCAGGCACGGACCCGGCGCGAGACTGGTGCTGGCTCGACGTTATGTACGCCGTGAGCTACGTCAAGCTTATTGTCACCCTCGTCAAGTACACGCCGCAGGTCATAGTGAACCATCGCAACAGGAGCACCAAGGGCTGGAGCATCTCGCAGATCGCCCTGGACTTTTCGGGCGGTCTCCTCAGCATCGCGCAGCAGAGCATAGACAGCTACCTGCAGCACGACTGGTCGGGTTTCACCGGCAACCCGGTCAAGCTGGGGCTTGCCAACGTATCTATGATCTACGACAGCATTTTCTTTGTGCAACACTACGTTCTCTACCCGTCGTCCACCTCAGCTAAGGGTCCCGAGGCCGAGGGGCTTTTGGGGCGTGACGAAGAGGAAAGGAGGCTGGACTAGACTGTATCGTAGTACATATGTACATAGGTGCCTGCTATTTGGGTTCTACCTGCATCTGTCCATAGATATCATTGCGTCGAGTCCATTTCAGGGCTTAATGCGGAGCATAGCGCATGTTCTCTGTGAGCAGCGAAACATCAACGTCAGTTGTTGGCTATCAGCAAACATGAGATACCTTTGAGACGCTCGCAAAGTCATCAGGCATGTCATGGGTGAGTTTTTAGGATGGATTTCAAACAACAGGCATTATCACGACCAGCGGAGTATAGATTTAAATTAGCCCAAATTCCTGCTCAATCCGAACTTTGTACAGTTCTGTACAGTATGGTACAAGGCCAAGATGGTGCGGTGAAGTAAGCAGATCCCACGACGCCACAGATCATCAGTCAATTCTAGATATTGATGCCTCTAAATAGCTCAGGCTGACAATGGCTTTCAACGTCGCTTCCTCTTGGATTGTTTCTCAAAGCCGATCTCGAAGATAAAAGCAATGCGAAGTAGCATCATCCCCACCAAAATACCAAGATTTATCGAGTCCACAGCTCTCCAAGCAAGGGGGTTGAGGATCACATCGACCCCCAGCTCCTTGACGGTGGCCCGGATGTGCAACATGTCGCTCACCCAAATTCCTCCCATCACAGCCTTCCAGACCCTGAGGTCATCCGTCACCCTCAACACAACGGCGCTGTTGAAAGCCATGAGGAGGTACACAGCACCAACCTGATCCAGGAGGATTCGATGTACAGGTGGATCATATGCGAAATGAGGCGTCATGGTGTCGATATAACGTGTTGGGTCGAACATGGCCAAGACAGTGCCCGATGCGCAAAACACTGGCTCTACCCAGGACAGCATTGTGCGGTACAGGGTAGGTATACGTTGTACTGAGGTCATGTTGGCTGGTAGAAAAGCGATGCCGGAACTATTTTTAGGCGCGATGGAAAAAAATAAATAAAAAAAACCAGAAGAGATCAGATCCCACCAGTAATAAAGACACTTGGCGCTTGGCTTGCCTTGTCCAGTAGTTTATTCGGCGGTGGGTAGGTAGGTAATTATGTAGATAGGTACCTACCTAGGTACGTACGTCAGCACAGAATTTTGGGGTGCCATGTACCAGTAATGTGGGGTTTGAGTAACTGCTGGTACGGCACTGCCGGGGCTCGAGATGACAGGGTACCATGCCTGAGGCATCAACAA

**> MD218_chr02:6508164-6512164**

CGAATCATGCAAGGCGTTACATGATACAATCTGGCGCAACCCCGCAAGAGAACATAGTTTTGCAACTTTCTCGTATTTAAATTCTGAGGTTGCTGGGTGCCACCACAGTGTCAATAACTGGGGGCGCGATCGAGGATGATCCTCAAAGGTCATTGCGCGCGGCTGCTCAGGGATAATGTTTCAATCCAGTAATATCTGTTAATCGGACAGTTCGGTATCCATGCAGATCCTTGCTACTATCAGCGCAAGACCAGTCGATGTAACGATCCATCACTTCGTCGTGATGGATGATTCTGGGTGCAATTCCAGCCATGGGGAAGTTTGTGCCATCTGGATCGTGAATGACAAGGCAAAGGCATATTGTCGTCTCAGAGGAGATTCACCCGACAGAAGACAGGTGCCAAGACGAATACCGGGTAGTGAGGGAAGTCGCGAGAATCAAAATAGTGAGTAAATTGGCGGAAAGACTTCATATTATAGGACGGTGTACAGCGTAGGAATCGACGCGTTCTAATGGTGTACGGAAGGGTCGAAGAAGTCTGGTAGTTGGTTAGGAGTTCATTGTAAGGGTCGTGGACAGGCGCGTCAAAGGTATCGTGAGCCCTTTTTCGAGTAGGCTTTGGTCAGGGCGACAAAATTTGCTAGCCTTCTCGTGCATCACTCGGTCGGACGGAAGCCGGTTCAGGCTCTTGCTTGATGGTTACGATCTGCGTCTGTCCAATATTAGACGGGAAATCATCATTAAAGGTTGATTGTTGCGCACCAGCTTGAAACATTTGAGACCAGTCAAGTCCACCAGATTGTTGGTTCGAGGCTTGTCCTGCAAACATAGGCATGCCGTTTTGGTTTGGCGGCATGGGGTAGTTCGCGAGCTGAGGGTTGATGTTCGCGGCCATGGTGTGCGGGACAGTTCCGTTGTAGCCCTGATCGAAGCTGCCGGGCCGCGTGTTGTTACCCATCACGGAACCTGTGACACTGCGTCGGTCGCGGTCGTTAGGGTCTTCCATACGGTTCATGCTGGGCTGTCTGGACAGGTGGTTCTGGTCGTTAGACATGTTCGGCATGCCATCCTGAGCCGGGATCAAGCCGAAAGGGTGCACCATCCCATCAGGACCCATGTTGCCCATGCCGTTAATGGGGTTGAGCTCGTTCTCGTTACCCATAGCCTTCTGCTGGGCAGCCTGGTTCTTCTTGACGTGCGCATGCGGGTGAGAGAGATGGCTTGCTCCAGTGGGGTTGCCACGGCGGATCGAGCATTTTTGGAAGTGGCGCTTGAGAATATCGCTACGGGAGAAGGTGTCGCGGCACAGGACACACATGTACGGGCGGTCGCCGGTGTCTATTAATGTGTCAGTTGAGCAGCTTTCACGTCAGAACCAGCAAAGCAGGGCAAAACTTACGTCTCAGAAGATGCCTCTTGAGATGCTTGGCGTGCAGGTACGTCTTGTTACAATGAGGGCACGGGAACTTCCCGTCGGCATCCTTCTGGGGGATCTGGTTCTTTGCCGCCTGGCTGCCAGCGACCGGGGCCTGGGGCCTACCCGGCGCGCTCGGGAGGATTCCTCGGCGACCCTGAGAGCCAACCACATGTGTCGGCGCGTCGCCTTCCTGTTGCATCATTTGAGGCTGGGGCATCAATGGGCTCTGCGCGTATGGCGACGACATGCCGCCCTGAGGGACCATGCCACCCGCGGGCATTGGACGCAATACCGGGGGAGCGCGACCACCAGGAGGCGCGGGCGCAATAGGTTGAGGGTGGGCCATTGCGGTCGATGCCTGGGTCATCATTGAGGTTGTGGTGGGATAGCCGGGGTGAGGTTGTTGGTACTGCGGCGCGGGCATCATGCCAGGGTACGGGGCTCGTGCAACATTTTG

TTGCCCCGGGGGAGGTGGATAGTTGCTCATTGTTGGAGTGCCAGACGGGTTGGGCGTGGCAGTAGTCCGAGGGGTGTTGTGGTAGCTACCGTATGGGGAAGCTTGTTGTTGTTGTTGTTGTTGCTGCTGCTGCTGCTGGTGCTGCTGCTGCTGCGCCTGTTGTTGCTGATGCTGTTGCTGCTGCTGGGGTTGTTGCATGCTATGCGTCTGCGGCTGGAGGGGAGGCAGAGTTTGCGGCTGTCCCGAATGGTTTTGCTGCTGATTCTGAAGTCCGTTTAAAGTCGCAGAGGGCGAGGGGTA

TGAAGCCGTAGTAGTCGTGTTTACCACATTAGCCGAGTATTGGGAGTTGTCCATTTCCTTCTCAAAAACAACCTTTGCGAATCTACAAAAGGCTGACCATTCGACCCGGAGATTCCAGGAAGACTGGCAGAGCGCCTAACGGTGAAGTGAGTGGTGATTGTGGTTTTCGGCAAAATCAATACCGTCTTGATACGACGCGATCTGTTGGTCGAGTTTGTGCGGGACGAAGCTAGAGCTGAGCTGTAGCTTTTGCGCTTGGTCGCGTTTGGCGGCGGATAGCTAGGGACTCGGTTGCCGTTGTCACAGAAAATAAATCTTCTGGGGATGGACGCGATGGTCGGTTGTGCTACACCAAAGCCGAACTAGTTGGGCGCGTCGTCGACCGTGCTGGGGCTAAAAGCTTGGTACGAATAGTTCGTTGATATGGTGGATAGGAAAGGTAGAAGTCTCGAGTGGAGATAAGCACACAAAAAAAAAAGGACGAAGTGGGTGATGTGAGGCTCAAGAAGGCGGTTCTTGTGGGTGGTGTGGGCGCGGGTGATTGTGGATTTTGGCTGATTGAAAAAGATCTTTGGGAGGAGGCCAAGTGGAGACCTAGGTAGGCTTGATGAATTTTTTTCTTCGAGCCCTGGACGCGGCGCCTGTGCAGGAGCTTTCTGGTTTGCGTCAAAGCTCGCGCTCTCCGTTTTCTTTCAACCCAGGCAGGGTATTATATCCACTTCTCTCTCACCGTCCAGACGTGGCAAGCACAGTGGTTCCCAGTCCCTGGTCAAAAACGCGCAAGGAGGTAGTAAAGATAGTATCGCGTTGTGGGCTGCGCTCTCCTTGCTTGCTTGGTGGGCGCAGAACGATTAAAAAAAAACCGTTTCCAAGTGTCGGGTGGAATGGTTGGATGAGTGGAGACCAAGGAGTGAGTGGACCGGCCGGGAGGTGGAGGCTTTTATGTGGATCGAAGATTGAAGACAGCAGCCAGCTAAAAAGGTGGCAGGGGCGTCCCCAAAAACCAAAATAACACCACTGCCAGGAGGCGCGTGTGCGCACGGTGGGTGAAGATTTGATATGTACGGGGGGGACGACTTTGTTTTGAAATTGAAAGGAATAAAAGCATGCGCGCTTTAGACCTCTTAACAGCTGCTTGTTCAATTAAAACAAGAACTTGAAGGGAGGCGCTGGCCTTTTTTTCGCCGTATTTCTCTCGACAACCAGGCAAAAATTCTGGGTCACGCTTCCTGTTTGTATTCCGCAGGAACGGAACCCAACGTACCAAATCAAGGCGGGATCATTTCCTGACCAGGGCCCTGTTCCGGTGCCGGTCTAGCGCGCTTCCAGCGGTCGGCTTACAGTCGCCCTCAGTCTCCGAAACACTTTTCCGCCCACGGACCAGGAAGGGTGGCTGGGGATTGCCTAATTCTGACAAGTTGACGTCGCCAAGTGCTCTTTCATGTCACATCACGCCAAAGACGTGCTTTGAATTCTACGTACGAGGACATTGAAGATCGAGATGAAAATTTTACAGATTGCTTTGTTCCTGATGTCCAGCGGAGGGGGGGGGCGATTATCTTATTTTTCAAGATGTGTGCAGTGACAACCTCAGAGTACACACCACCTTTATCCACGACTCTCAACTTTGGCCATGTATGCAGTTGATTTGTAAACATAACACATGCATAGGGACGTACTAGCATTCGAGTCCTGTCGTAAGAGACCATCAATCATACTGGCTCACGTGGCCGACCTCGGATCTAGACGCCGCCCCCTTGGAATGAGACCGTGTATGGGCTGGCTATTCATTGCGGCCATGTTACGTCAATCTGGGCTGGTGGAC

**> MD219_chr02:6764168-6768168**

TGAATAGCCTCCTCTGCCTGCCGTAGGCTTTATTTGCCGGCCGGCTATATTTGATGCGACAAGCTTGGTCCACTTCACAGCATGCATGAATACATTGGGTCAGACGTGGCAAGGTGGGTAGGCTCAGTACCTACATAGTAGGTGTCAAGCTGTACTATGATAACCTCGATGATTAAAATATCTGAAAAAAAAAAAAAAAAAAAAAAAAACCCTCATGGTTTACAATATGCATTGATAGGTCATCATGCTCAGCAAAAGAAAACATACTTTTATCAACCTTGACGGAGCATTGTCACCGGTGTGTCCCCATTTCATCCAATCCAGCAGAGTTTGATCTCAACTGTAACCTGACCGACTGAGGACGATCCCTGGACTAGCGATCCACAGGGGCAAGAACGACGGACCACAGTCTAGATTCCTTTGTCAATCAACGTCTCCGGTTTCTCTCCGTTTGGCTTGCCCCAAGCTCCAAAAGGATCATCGAAGGGTAAAATAACTACATGTGTGTATAACGGTAAAGTAAGTAGGTACCTACCTACCTACCTACCTACCTAGGTAGGCAACATCCTGCGTCTGCATCGTCTTCCATCCTGCCCGAGAGAGAACTAGAACTAGTGGTAGCACTTGCGTGTGTGTTACCTAGACCACCCTTGACTAGGTAGCTAGCGGTGTTTCGAGCCCTTATGATATTGGAGAGACCAAAACCTCGGGCTGACACAGGGCTGTACAGGGTTCAACCCCCCAACATGGCGCTGTCGGGTTTAACCCCAACCACGCTGAGGACCCTGCCGTCAACATCCATAATACGTCGTCGTATTGTAATTACCACCAGGGTGAAAATAGCTGAGAAAAAAAAAAGAAAAAAAGAAAAAAAAAAAAAAAAAAAAAAGACAGCCGCCCAAGCCAGGCAGGACGAGGACGGGTGGGCGGTGGAAAAGAAACCATCGAATGGGGCTCTTCAACGTGCCACTTCGGGAAAGACTCCCGTAGTCTGAAAACCCTGGATTAACGGATACCAGTCCAGGTTGTCAAGATCGTACCTCCGTGCTTGGTCTGGAATATACCAAGAGCACATTTACAGTTGAGACAGGCATGGCGGAGGAAATACCTAGGACGAAAAACAAATCATAAAAAAAAAGGACCCGTGACTGTCTGGAATGACTGCTTTGCTTGCCGGGAAATCTGCTGGGCTTCTATGCTACTATAGCTAGCTTCGAAAAAAGACCACCCACGAATCACCTTGCACTTAACGGGCAGCGCACTTGGCACTTGGCACGCTCAGTCAAATTGCCATTGAATTTGCCATCAAGGACGCGGGGGACAACAACCAGCAGACTAATCCAAAGCTCATTCACTCACTCGATTTGATTTGCTCGACAACCTGCACCCGTCACCACCCAACCACAATTGCGCCACTACGGAGTACACAACCTCTATCCACTCACTACCTACCTACCTGGTCAGAAAAGCAGGAATATCCTTTCATCTCGACAAAAATCACCTGCACCTACCCAGTTAACCGCCCCGTAGAGTCCACCGACATTGGAGCCCTGCAGACAACGTCAAGCTCAGAGTCGCATTTCGTATTTTTATGGTTGATTTCTTCATTCCTAATTTTTTTTTCGCCCTCGGTCTAGCCCTACCAGGCCCTCTTTTCTGACCGCCGACCGCCTCCCGCCTCCCACCTACCGTCTCCTAACCCCCGGTCCACGGTAATGTATACAGCATAGGGTAGACAACGCAACCCGCTATGTTTTGCCACTTCGCCGATCCGAGTGCTTGTTTCCCAATCTGCTCGCCTGGCTGCCTACGCAGCTCACTTCCTGTCTCGGGCAAGTAAGTGATCGGCGCATACAGTACAGTACCACCTGGCGG

GGCTACCAAACCCCCCAAATCTTCTCCCTCGACTGCGACTGGGCAACAGCAGCCATAGAGAAGGCAAAGCGGCGCCCTCTCCATTACCGCCACTGCATTGTACCCGTACCGCACCGCTGCAATACCGCACGCATCGCACGCATCGCACGCATCGCACGCATAGCATAGCACAGCACAGGACAGCAACCCAAGACTCCGAACCCGCCTGGCGACGCATTCTAGAAC

TAGACTTAGCTCCCACCTTGCTATCGCCGAGCCCCTCCTTTCTACCGACTCGATCCCCACAAACGTCCTATAGACTATAGATCGCATACGGGCTGGTCGATCGATCGTTTTTCGGTCGTCGCACACAAATCAACCGCTTCGAATCCCTTCACTATATTTGCGTTTCGCTACGACTACATCTGCTTCCTCGACACTACGCCGATCGACAAGTCACCTATTAGTACCTGGCTTGTCCCCATATGGACCTGGGTCACGAGCCCGCCGCGGGCGATGCGACCCCCGCAGCGGAGGTGCAACAATGCCCGGACATATATTCCCAGCCGGAGAGTCGCAGAAGCACCCCGGATATACTTTCAGGCGTGCCGTCCCCTACAAACTCCACCGAGACCGCGGGAATGTCTCTGATCAGCAGTCCGACGCCGACGCAAATCGACCCATTCGCTACAGCTATTTCTGGAACCGCAGGGAACGGCACTCCTAGCATATCATCATGGAGTTCAAACTCGGACAGGCAACTTGGTCACGCCTATGGAGACGACCGAGTATTTCCTATACGATCGGTGCTAACCGTCGATAACTCGGGTCACTCAGGAGAGCAATCGGATTATTTCCCGCAACGTCACAGTCATGAAGACACCCCGCAGTCACCTAGAGGAGCTCTAAGGTCGTTTATGGACCTTGCAGGTCCTGTCAGGCGTCCAGGCCCTGCAAGTGAGGAGCCCAGCTCTTCCACTGTGACATCTCCCAGCGAACGTGTCGGCCGAGTTGAGCCAGGGGGGAGACAGAGGCAGAATACCGGCACTGGATCTGTCGTGAGCATCCATGGGGATTTCAGTCGCCATACTACCTTTGTTGAGCCAATACCAATTTCCCTGGACGATGATTCATCGTCTGATGAAGGTGGAAGGCCGCCATCCATTCCGGCGGTAGCAAACACTAGTGAGCCCAACGACGGACAATCGGTGGCCGCCGACGAGCCGATAGATGAACGCTTGACGAATATGAGGTTCGAGCACGTGGTGACGGCAGAGGGCCATGCTGTAATTACGGGAAGGTCTGGAAAACTTCAACGATGCGAGGATGAACCTATTCATGCACCTGGTGCAGTTCAGGGCTTCGGAGCGCTGGTCGCCCTCGCCGAGGACGAGAACGGTTGTCACTTGGTGCGCTACGCGAGTGAGAACACGAAGCACATATTGGGATATTCTCCCCAACGGCTGTTTAGCCTGGAAAACTTCTTTGACATTCTCAGCGACGAGCAGGCCGAGAACATGCAGGACCATATGGACTTTATCAGGGACGAGGAAGCGAACCCCGAAGCCAACGGACCCGAAGTGTTCAGCATTTCCATTCGCCTACCAAAAGCAGATCAAAACACGAGGATAAAGCCCGTCAAGATCTGGTGCGCCATGCATATCAACCCGGCCCGTCCCGAAATGGTTGTGTGCGAATTCGAGCTGGATGACGACGAGGCGAACCCTTTGAGGCCACCGGGCGAGGTCACTCCTGAAATACCTACAGACACGTTGCAGGCGAACCCCACCGCGGAGGAGCTCATGGAGAGCACTGAGGTTTTGAGCAAGCCCATCAGAGTTTTGCGCAGTGCTCGTAAGCGAAGGGGTGAGGCCGGCGCCATGCAGATTTTTGATATCATGTCGCAAGTACAAGAGCAGCTCGCAACTGCCGACAACCTAGAGAAATTTCTCAAAATTCTGGTCGGTATTGTGTCCGAGTTGACCGGCTTCCACCGTGTCATGATATACCAGTTTGACTCCGAGTTCAATGGCCGAGTGGTCACAGAGCTCGTCGACACGGCCCAGACGAGGGATCTTTACAAAGGTCTCCACTTCCCTGCATCTGATATCCCGAAACAGGCTCGCGACCTATACAAGATCAACAA

**> MD220_chr02:8040406-8044406**

CAGCACGACACGGTCGCGGTAAAGTCGAGCTTCACCAGGAGCCAGCCGTCGCCGGCGTCGTTTGCATTCGTCGCCTTTTCTGTGTTTGGTTACATCGCATCAGCGCTATTCTTTCGGCTGTCGACTGGTCGAAGCCAATGGTAAATCCAAGACGGAGGAGAGAGCGAGAGAAGAAAAAAAGAAAAAGGCCTACGTTGCTTGACAACATCATTGTCGATGCAAAAGGTAAAGAGCTTGGGGTTCTTGAACGGCGATCGCCACGTCACGTTGACGACGTCGAGCGTGTTAAAGGTCTGGAACTCGGTGGGAAATATCCAAAACACGCCATTCTTGCCCTGCGTGTACGTCGTGTCTATCTCCTGCTGGACGCCGAGGCGACAGAACAAGCCAAGCGCGAGGGCGAGCACGGCAAGGAGAGCCATGTTGGCCTGCTGTCGTCGAGTGGAGACTATTACTTTGACTCGTCTCCCAGTCACGTCGCCGCTGACAAAAGAACAATGCAGGTTCCCGTCGGATCCCCGGGTTGACGACGGGCCTTGGGGCTAAAAAAGCAAAAAAAGACAAGTCCCCCAAGAGATCAAGATGCAAGAAGCCGTGGGCACCAACAGAAAAAAAAAAGGACGGGGCCGTCCCGAGGAACCCTCATGGTAAATACTCCGTACCTCTCTCGGCCTGTCGGATTGGTAAGGCTGTCTAAATGAAATGTCCACCACACTTCTTGGCATTGGCCCCCTCTGCACCGCAGTCGACGATCGAGGCCAGAACGGAACCGTGGGGACCGGGGGGTTGCAAAGAGATTTGCGTCTCATTTACTCTGGCCCACTGCTGACAGCTTATGGAGAAGAATGCAACGCTGCAAACTGATCGATTGGATGCCTGGACTTCGGATCCTCGGCAAGGTGAGAATTTCAGGCGCCAGTCTCGCATCGGCGCTTGATTGGGCGGTAAATCATGGAGCCGAGGGGGGGAAAAGGGAAAAGAAAATTTCCATGTATGCAATACCATCGCTGCTATGGAAATAATTAGATCAAGGTAAAAAAAGGTATGTACCCTTGGTACCATACTCTGTACAATACAGTACCAAGCCTTGCAAAGCCTTGCAAAGCTTTGCTTCCGTGCCACCCACGCACACGATAATAATATCTCAAATGATAAACCCTTGCCACACACACCATATGCAGCTTGGCAGCCTCTCTCGCTTGGCTGAAACTCTGCCACCCTCCAAGGCACCTTTCAAGTTTCCTTGCCACCTCGCCTTGCCACTACTACAATAGTGTGCGGTGCTGGACACAAGCCGCCCTGCCTTTTACCGGTACTCCTTTTTTTCCTCCTTTTTGTCTATCCATTTCCAATCCTAGATCTAGACCTAGACCTAGGCTAGTTTGACGGCGGACAAGCACTGCAGTGGACGGCAATGCCATAGTTCTTGCCCGGCTTGCCCTCGGCCGAGCAGTTGGTCCCGGCCGGCAGCGGGAACTTTTGCTGAATGGCCCACAGGCTGGTGGTGGGCGAGTGGTTGAACAGGATGGGTGCGTTTGGCTCCTTGTCCCGGAAGAGCTGGATCACGTCGGTGAACCTGACCGTCAGCACCGTGCTGTTGGGCCCGCGCAGCCCGTAGCCCACTCCCGTTCCCGTCCACTCCTTGACCCCGGGGACGTTGTCCATCTCTTCTGCGATGTCGCTGATGGGGTCTTCGGGGCCCACAAGAATGGCTGTTTTGGCGCTGTCAGCAAGTTGCATCATTCGCATATGGCCGACGCAAAAAAAAAAGAAGAAAAAAAAAAAGTCTCGGTACTTTACTCACCCTTTATGTTATAGCCAGCTTGCACGATTTGCTGTACATCAAACTTGATGGCAGCCTCGACCATTGCTGGGTTAAAACCTGCAGCCTGGACTGCTGGGTGGTTCCACGGTAGGCCGCTGTGCCATTGTTAATGTCAGCTCATATAT

TCGTCAATGACCCGCAAAACAAGAACAGGGGTCGTCTCCTGCAGGCATCATGTGTGTGTGTGTGTGTGTGTGTGTGTGTGTGTGTGTGTGTGTGTGTGCAGCTTTTATACGTACGTAAAGACAAAGTTCAGCTTGCCGCATTTCGAGCCGCCTCCTTTGCCCTTGCCATAGTCTTGCCTGGCATGCAATGCATTGGCATGCTGTTGGCCGTGACCTGCAGCAGCT

GCATTTGCGGCAAGTCCCAGGAGGATGAGTGGTTTGATGGCGACCATTTTTGTGATGTTTTCTCGTTTGGAGGATATTAAGATTAGAGTTACAAAGGGCAATTATTTGCTGAAGCGTGGTAGAATTGTGATCTCGTTAAAAGCTTGATGGGGAATCTTCAAACCAAAGCAGCGAAACAGCAGGCGGCGACGGTACGTATTTGTAAACTCCCGCGATTGCCCACGTCTACACACCCTGCCCGAGTCTAAGTAAAGCAAGCACTAGCTATGTCACATAGGGTCAAAGAAGGATCATCTGGAATGTTGCTAACGGCAACTCCAGCGCACTGCTTTTCGCACAGGACGAAAGCTAGTAATTGGTCCAGACCACAATTGACCCCGAACTTTGTCCCATGTTTTGCCTCCCAGGCCGGAATTCATTTTATTTCGTCACACACAAGCGTACAGACCTTTTTTTTAGAGCAAGGGCCAGCACGGCTGGAGATGGAAAGAGGCAGCATGCGCTTTACGCGTCGACCCCTTCATCTTCCGACAAGCCCCTTGGACCCCTTGGACTCACATGATTTCTTCAACGGCTAACGTGCTAGCTTCCAAACTCCGTCGGGTATTATCGAGTTCGATTCCAGCCTTATGTCCCAATGGCTGGGGGCGCCGTCCGAGCCGGCGGCTCCAATACATGTTCTAGCCGACTTTTAGCTCCCAACTTGGTTTCGGGTAACTTTCAGCGCAGCGAGGTGTGGGATCCTTAATGAATGCCATGTTTTGTTTCGGGCTTCTTTTACCTCCCGATCCAAGCGGGATCAGTATTCACCATTAATAATATGTAAACAATAAAGAAACAAGAGAACAATGCAGAAGCGCTGAGATGTCCAAGTCAAGACCGTTGTCGAGACGACGAACAACATAGTTCCGATTCCGCTAGCTGTCGAATAGAGTGACAGTACCCCCAAGGGTCCAATACTGTACAACTACAAAGGAAGCTACTCCCAGACGGTCAGCAGATGTGACGGCATCCGATATAGCTTTCTATTACGGCGGAGCATCAGCACCGCTTTTTATCTCGTGGACATGGCGACTAGAGCTCACCGTCGGCTGTCGCTCATCCCTGCCGACGTGCCCGGGCTAACCAGGCAAGCCAGGCAAAGCCATACGAGCTTGATTGTACGCGTGGGCCGACGTATGGTCGTGTTGACCCTATGCCAAGGTTTCTCCCCTATCGACGGTGAGTTGTCTAGCTGCGAAGGCGGTGACCCTTGGAATGCCAAGTGGCCATGGGGCGGAGCGGACCGGGTAGACTAAGACATTAGGGGCTAAAGCTTGCAATCATTACATAATGCCTATCCAAAGCTACGGTACCTTGTCTTTGCTTGTGCAAGCTTGTGCAAGCTACCGAATATGTGAGCCATGCTTGCCACGCTCCTCCGCATCTATACAAACCCGCCAGTCGAGAGACTCGGTGGCAGATCTCCGTCCGCCGTTCCCCAGCTAGTGCTTTCATTAGGACCCAGAGTCAATTCCAAAACTCCCCCCTTGACCCAAAAGTCGTGCGTGATCCAGTTCTTCGTATAAGGCTTCCCGTCCAGCGTCGCGCTCTGGATGTAAATGTTTGCGTACGCAGCGTCAAAATTCACGCTTCTCACCGTCGCAGTCTTGCCGCTCACTGCATTCGTCAGGTTGACCTCGCGGAAAAACGGCGGAGTTATCAGGTAGACGTCCTGACCAGCCACGGGATACAACCCCAGCATGGCCATGGTGCTAAAGCTTCCCATGGCGCCACTGTCGTCATTGCCCGGGATTCCCGCCATCGTGTCGTTGAACTGCGACGG

**Chr.3**

**> MD301_chr03:867112-871112**

GCGAAGAGATAACGGCCGGACAAGACGTCGTGTTATAAGCCTCAAAAGTTCCAAACACGGGGGCGCAGGAGGAGGCATAGTGATAGATATTTACACATGATGCATGTTTACATAGTGACAGATATCTGCATAGCGAGTCCCTCGGAGGCTTATCGGCGTTGGCCAACGGATCCTTGAAGCTCAGGCCGAAGAAACAAACGCTTGGAAGAAGCGAGCCAAGTTCGAGTCATTAAAACGTCGCATCGGATAAGGTGTGGTCTTTTCCTTTTTTGCAGGAAGAGTGGTAGAGTCGGAGATGGCCGTAGGCGATCGTCCTTTAGTTGGGATCAATACGTAGGGCCTAATACGTAGGGCCTAGGCGCGTGCCGTGGCCCCTGAATCCCCCAGGGTGGTGCCCGGCAACAAAATCGGGGGGAGCAATCGGGTTCGAGTCATTGGGTTTGCCTGAGCCTATTGGTGTAGCCACGCGGACTCGGTACCGGGGAAATTGTCTGTCGTTTGGAGGCTTATCTGCGGCGATCGACGGATCCCGTGAACCCATACATTAGAAATAGCAACGTAAAGGAAAGGAAATTGCCCGTCTCCATCCACCGGCAAACTGAGTAGCTAGTCAACTGTGGGGGGCTGGATTCGTGAGTGCTGTGCAACTAGAATGTGGGTTTTTTTTGGAGGGGAAAGGGACAGATAGATGACAGCGGATGCAGGCCATGGCTAAAAACCGGCCTCTTCTACTTTGCCTCTCTGTAGACACCGACAATCAAAACATGTCACCATGACAGTCCTCGAGCTTCCAAGCGAGCGCCTGGCGTCGCCGCCCATTGCCGTCTTGACGGCGGCACTCATCGTCCTCGCCGTGGTGTTCAAGGCCGTGTACAACATCTTCTTCCACCCCCTTCGCTCGTACCCGGGACCTCTCCTGTGGGGAGCCTCGAGATTTCCCTACGTCGTCCGCTTCATGCAGGGCAAGATCCCCTACGACCTCAAGCGGCTGCATGCCGAGTACGGGCCCGTGGTGCGCATCGCCCCCGACGAGCTGACGTACAACTGCTCCAAGGCCTGGAGGGACATCTACGGGCGCAACAAGCAGCCCAACGGCGAGACCACGCTCTTCAACAAGGATCCCATCTTGTACTCGGCCGTCTTTGCCGACCACAAGGCGCTGATCGATTCCGACGGCGACGAGCACCGGCCGCAGAAGCGGACGCTTTCCTACGCCTTCTCGCATCAGGTCTTGGTTGAGCGGGGCCATATCATCACGGGATGCATCGACCGGATGATTGAGGGCGTCAAAGCCCATCTTGGGTCGTCCCCGTCAGTTTCGGCCGTGGTCGACATTTGCCCGTGGATCGAGAATGCCCTCTTTGATCTCGGCGGACAGCTCACGGTGGGACGCGACCTCAACGCCATCATGTCTCAGGGCACCGTCCACCCCTCGCTGGCGCTCTTCAAGAGCCTCTTCGACTTGGTGCAGTGCTATTACCTGCAGCTCCGCCGGGGCCCCGGCATCCTCATCAACGCCGTCGAGAGCATGCCGGGGTTCTCGGGCTACGACTGCGTGCTGCCGGTCAAGGACGTCCACGACATGATACTCAAGCGCATGGCCGACGAGAAGTACGCAGTCGCGGACGAGAACTCGGGCGATTTCGCTTCTTACCTGATGAGCAGAGCAAAGAGCGAGGGCTTGAATGACGACAAGGTCTGGATCAACTGCGTGCACTTGATCCTCGGGACCCCAGAGGCCATGCCGTCTTCCATAATCAGCACCATCTACCACTTGCTGACGACGCCCTACGCATACGCCGAGGCCGTGAGGGAGGTCCGCGCGGCCTTCAAGTCGTCGGATGAAATCACGGCCCAGGACGTCCAGAACCTGCCCTACCTGCTCGCCTGTTTCCGCGAGACGCTGCGCAGACGTCCAACCATCCCCGGCACTCTCACTCGCCAGGTCCC

TCCAGCTGGAGCCGAAATCGCAAGTCCCCTTTCCTCCTTACCTACCTCTGTCTCTCTCTCTCTCTCTCTCTCTCT

GTCTCTTCGCACGTATATCTAACCGAAACCCTCCAGTGCGGCAAATTCGTCCAAGGAGGCACCATCGTCGGCGTCAACCAGTTCGCCACCTCTCGATCGGCCTCCAACTTCCACGACGCCGACTCTTATCGGCCCGAGCGCTGGCTCGACCCCAGACCAGCCGAGTTCGAGGGCGACGACCGCGACGCTTTCAACCCGTTCGGCTACGGCCCTCGGAAGTGCATTGCGAGAAACCTTGCCCATCTCACCACCCAGACCTTTCTCGCCAAGCTGCTCTGGGAGTTTGACATGGAGCTCGACGAGTCGTGTCGGGACTGGGACGACCAGGGCCTCTATGTTCTCGCGCTGCGGGGCCCGCTGCTGGTGAAGCTGAGCAGGGCGGCCCAATGACCTGGTGAAAATGCGATGCAAAGGCTGTGCCGCCCGAGCCTCGCGTCACTGCAACAACTCGTTTGGCCGTAGCACTCCCTTGATCTTGTTTTTGTTGTTGTCGATTGCGGGAGATCTGCGACCATTTTTATTAATTACGAAAGTTTCGTCAGTTTTGACACGAAACATGTTTGGAGTAGCGCAGCTTATTCTTGGTTTTATAGTATTAAGCATAAGTAGATAGGTATCCAATGTGTTGGCCATAGCCCTGTAACAGCAAGTAGTGCAACCCTGAATTTCGTACGGAGTAAAGGTGGTGTGGGGTTTAAAAACGTGATTGAAACAGTAAAACGGTACAGCACAGGCTTAGCTGGACGGACGCATGGGGACCAAAGAAAACCATCCGTTTTTGGACGACCTCGGATCCCAAATTCGGGAATTTTCTCTCCCACTAATATTGTGACGACCGAACTAGGTAGGTAGTACTCAGGTGGGTGACCATTGGGGAATTCTCGGTGTTGTATGCTATTGAATTTTTACCGTCTGTGCGTATCATACGGATACAAGGTGCTGGTTGACCAGAGCATTTCTGTTTTTATTATATGCCGTCCTCGGCTGCGCCCAAACTATTGAACAATGCAAGGTGCAGAATGGTATCATCCTCAATTTATTGGTTGCCTAGGCAGTCCGAACACCTTTTGTAGCCGCCCGATCTGCGCCACTGTTTATTCTCCCTCGACCATACTCGATTTCGAGTTATGGGGTCTTGTTTAGTGCACATACCAAGGGAGAAGGCTGTTGATCATCATCCGGCTGAGGGGCTGTCCTTCAAAGCATCTTCTGGTGCGCATATAACTTTACGCATGGTTTATTGTTGCCAATTTGACGATATGAGGGGCTTGAGACCGGGAGCACATGCATATCTCGGAACCGTTCTCTACCAGAAGTACAATTTTTGATGGTGGCCTGTTGCAGCGCAAACCAAATCAAATGCTGTCATGAACGCAATTCAATTATTTGAAAGAAAACAAGTCCCAAACCGGCTCGTTTTCGCGTTTAGCAACGAGGGCAATAAAATCCTAGACACAAATGCAGCCTGACAATCGCCCCAAGTATCTTTGACCCTCATGTCAAGATATCTCCAATAACACATGACACATATACTACACACCGGACCGGTCAAGTCCCTCAGGCCCAGGACCCGGGGACAGCATCACCCTTCAAAGGTGCCTTCTGACACTTTCATTGTAACCCAAGAAACCATTGCCCCCACAATCATCGAGTGTATGCTCCCTTCTAAATCCAAGTATTGCTAGGCCCAAGATGTCCTCCGAGCCCAACTACAAGACTGGAACCGCTTGCACCGAAACCCTTCTTTTTCAGTCCCTTCAGCGACCCTTCCGCATTCCCACTTGGTCTTTCGAAAGGTGATGACTTGCTGCTGGTGGAGCGATTCTTGGCGCGCGGGCTCCAAAGCTTGGCTAGGAAGTTTGTGCTGGGCTGACGCTGACGCTTCAAGCTTTCAGGGGTCAGCGGTGAGCCGCCAGCCCACTCTAACTCCGATACGCTCAAGGTGCCGAAAGATTGGATGGATGGATTGGCCCTT

**> MD302_chr03:1143485-1147485**

CTGAAAAGCGGTTATTAGTTTTGGATGGCCATGGATCACATATAACGGACGAATTTATGCTTCTTTGCTTGCAAAATAATATTCAACTCCTATATTTACCCCCTCATTCGTCACACGTTCTTCAACCATTGGATCTATCGGTTTTTGGGCCGTTAAAAGAAGCTTATCGACGTCAACTTGGATTTGTTAGCCAATTTTGCTGTTCAACAGTTATTGGAAAACGAAATTTCCTACTTTGTTATCGAAAAGCCAGATTAAAAGCATTTATAGCAAAAACCATTCAATCTGGTTGGCGTACAACGGGGTTATGGCCGGTAAACTTGGTTAAACCACTTTTAAGCCCTTTTTTGTTAGAAAATAGCAACGCCAACGTTATAAAAGATAAAAACAACGGTTTGCAAAGGGATAAAACACCGGAAAGCCCAGCCCAAAAAATTAACGACCCGTCTTTACTTATTTGGAAAACCCCTAAAACGACCCGAGATATCCGACTTCAACTGCAAAAACTTTCCCAATCCAACAAAACCAACGCTACTTCACGTCTTTTATTTGCAAAAGTCCAAAAAAGCTTCGAAGCCAAAGATACCCTTTTGGCTAGCGCCCAGCAAAAAATCAGCTTATTGGAAGCACAACTGGAGGCAATACGGCCGGTTAAAAGGAGGAGGGTGGTTCCGGATCCAAACGAGCTTTTGGTTAACAAACAGAACATTATTGGATTGCAGGAAAATGATATAGAAAATTTGGAACCTTTAGCTGATGAAGAAGAGGTTAATGAACCGGAGAAGCGTGAAAACGATTGTATTTTTGTCCGTTGATAATTTTATATTTAAATCAGTTTATAAAAGTAGGCATTTCGTTGTTAGATTTTCAGGGTGCCGAACAGGGGGGGTGCCGAACAGGGGGTACGCAACGTTACTCTATTCTTTATTTTTTTTTTACTTTTACAGGAGGAGATGGTGCATTCTCTTGAGCGGAATACTGCAGCGTCTGATCAGATCTTTTGCTGTAGTTGCGCATATATTAGATAATTTAGATGGTGACTGGGTTTTGCGAATGAATGTTTGTACTGCGTGCTCACATGCTTTGCAGCTCTTCCCGACCATGCAATTAAAAGCAGTCCAGACTTGCTACTTCGGTGGCTCGTATATTGAAACACAGTGCTCAAGAATACTTGGGTATGTTGATGATTTGCACGTGGAAGAGGGCCGGAAAGTAAGACCAAGTCCAAAGGTAGTCAAGTGATGTTGCAGCTACCCACTTGTTGACGGCTATCACACAAAACAGAGAAAACCGGTTGTAGCAACACTCCAGGAAGCGAGCTCCGCCCAAGACTCATCTTTGAGTGAAACACAGGTATAGGCCTTTGTAGTTGGAAGGTCCTAGTGTATCAGCCGCACCCCTTTGGCTCATCGTTCATGAGTCCATGTCGATCACTACCGCCATCGTATCACTATTCTGGGGTCAAAAAAGGCCTGAAACTAACGAATACGATTTTTCTGTCGGCCGACGATGGATATTGTAGCGAAAGCAAGCAAGGGTATCATATTGTCGTTCAAGGTATCTGTATTTAGAAAAGAAGGGGCATTGGCACGGTTTCTGGCCTCGGATACGTATTTACGATCAAAGCCAGGACTTTGAGCCCCAAGGCTACCTAGTGCAGAATCCAGAGCAGCTAACTCCAATTATTCAAAACCAATCTTGCCAAGACAGTGAGTGTTGTGGTGAGGGGAAAAAACCAAAGGAGGGGGAACAAATCAATGGCGACCTTGAAAGAAGACGGTTGAGAGAGTGTTGCACTGC

AGCCAAGCGACAGAGCTAGACTGTCTTGGCATCTGACTCGTCTTAATGGCAATGCGTCCCTTTCTGGACAGGACTTAATAATACCACCACTGTTATTATCCTGCGCCTGATGACCCAGTTGAATGGGCCCGGCAGGCTGGAGCCAGTTGATCCATCTACCGAAAAAACAAGAGCAAACTACCTCAACAGATAGCAGACCCTCCCACGGTCCCACGGTCCCACGGT

CCCACGGTCCCACCCCTCTGGACCACACCCATGCATCCGTACTTTCCGGCTGCCAGAGTGTACCCCGTTAATTGCATGCGCGTTTACGCTTGCAGTAGCAGTTTGGTAGTGTCGCACAGGTTTGAGGGTGGAGGAGAATGTCCCAAGGGGGATGGTACTGCCGTAAATGGCCTATGTAACGAATCCATTTTGGTGCGATATATATAGGCAAAGCTGACCGTGCCGCCCCTGACACTGATGCAGACAGCCAAGAATCTCATACGCTTCCAAAAAAGAAAGAAAAAAAAAAAGCGACTCTACAGTTTTGAACATCTATATATCAATATTTTACTCTTGGAAACATGGAAATACAACAACATATATGTGTTATTGGCGCAGGACCGGCAGGGTTTGCCCTTGCCGCAGACCTCATCAGCCAGGGAAAGCAGGTGCTGCTGTACTCTCACCCAACCCATATGCGGCAGGCCAGCGCAGTCATCAGCAATGGCGGACTTGAGATCAGCGGAGCCATAGAAGGCTTTGTACGGATCCCAATAACGACAGATATAGCCGAGGCGCTCGCATTTTCACAAGTTGCATTCCTCACCGTGCCGTCGACGGGCCAAGAAACAATCCTGCAAGAGATGGAAGGATACGACCTGCGCCACCACACCCTCATCGCCATCCCCGGCAACCTCCTGTCCTTGATCAAAAAGGCCAACGTGGAAGTCGGCAACATCCTAGAGACCAACCTGTCGCCGTACTCGTGCCGGATGGACGGCGGCCGGCTGATCGTGCTGGGCCGCAAGAAGCGCGTCATGATCGCGGCGCTGCACAACGACGGCGACCAGGGCCCCGCGGCGACGGCCCAGTTCCGCGCGGCGGTGCAGGACCTGTTCCCGGCGGGCGTGGCGCTGCAGTGGTGCCACAACGTGGTGGAGGCGTGCCTGGCCAACGTCAACGGCGTCTTCCACCCCATCATGATGCTGATGAACGCGGGCCGCATCGAGAGCACGGGCGGCGACTTTCTCCTGTACCGCGACGGCCTGACGCCCGCCGTGGCGCGCGCCATGCTGGCCATCGACGGCGTGCGCGTGGCCATCGGCGCCGCCCTGGGCCTGCTCGTCCCCTCGACGCTCGACGTGTCCAACGCCTGCTACGGGCAGGACTTTGCCGCCCTCGAGACCCTGGCCGAGCGCTCGCCGCCGCACAACCGCCTCCGCGCGCCCGCGGAGTTTGCCGCCCACCGCAACATCTCGGAGGACGTGCCGGATCTGCTGGTGCCGTGGTGCGCGCTGGCCGAGGCGCTGGGCATCGACGCCTCGCCCGTCGCTGCGGTGGTTCTCTTGGCTGAGATGTCGACGGGGGTGGATTTTATGGAGACGGGGCGGAATTTGAAAAAGCTGAAGCTAGATGCTTTGTCGCGGGGCGAGCTCGTGGCAAAGTTTAGTCCGCCGGTTGCGCGGTACGAGAGCCGGCTGTGATGGTTTTTTTTTTTTTTTTTTTTTCTTTTTTTGTTTCTTGGGAAAATGGTCAAATGCACTCATGTTTATAAAATGATATTTACTGTATATATGCTACTTAGACCTTGTTTATTAAACCTCCATCTTGTTACTCTTCATACCGCGAACCCGCTCCCCATACGAGCGCAAGACCCAGGGCAAAGGCGCAAACAAGAGCGACAGGAATCCCAGCAGACTGTTCCCCCACCCCCACCCCAGCGCGCGGTACAAATTCAGCCCACCCACCGGGATCAAAGCACCCGCGAGACCCCGCAGCACCGCGTTGGCACCCACGACGCTGGCGGCGTGGCCCTCGAACGCATCGATCAGGTACGTCTGCATGGCGACAAAAATGAGGATGGACCCGAAGCCGGTGATGGCGGTGCCGATCTGCGGCACGATCCAGTGCGCGCGCATCTCGACCGTCCACCCGTACATGAACAAGCCGAGCGGGAAGGCCAGGGCCCCCGGCACCGTGATGATGCGGGTCAGTC

**> MD303_chr03:1420947-1424947**

CTGCTCGGATATAAACCAGAAGACGGGTGGCAATATCCTTCTTGTCGAGGGCACCTGCGGCACCGGTCTCCTCTTCTTCGGATGGATGTATCGGGTTTCCATCCCAATCAATTTTGCCTTGTTTCCATAGTTCAAGCTTTTGCTGATATGAAGGAGGGTATAGCCACATGACGAGGGGCGAAGTCAAAAATGTCGTAACAAGAGCCATGACGACGAAGATTGTAAAGGTTCTGGTCGAGAGAATGCGAGCCTCCCGGCCGATGTTGAGGACAATAAGTTCGACGAGACCCTTGCACGACATCAGCGTACCGATGGTAAAGGACTCGCGCCACACGAGACCGTTCAGCCTGGCACCTATGGTACCGCCGATGAGTTTGCTGAAGAAGGCGACAAAGATGACGGCAAAGACATAAGCCCAAATCCTGCCACTATCCAAAAGGCCAAGGTTCGTGTTGATACCAGAGAGCGCAAAGAAAAGAGGGACAAAGATGACAGCAACCAGGTCTTCAATCTTTTCTGTCAGCTTGACGGCGAAACCACCCTGATGGGGGCACATGAGACCGACCATAAAGGCACCAAAGATGGAGTGCACGCCGATGATGCTGGTAAAGAAGGCAGAGGCAAGAACCATAAATATGGTCAGAGCCACCACACCTTGAGTTGGGCCATTTTCCAAACTGTGCGTCTTGTGCAGCACCCACATGAATGCCGGTCGGACTGCATAGGCCAAGAACAAGCTGAACCCGACGGCGACCAGGAGAATATATAGGGCTGTGATGCCGGCGCCAGAATTGACGAGAGTGACGCACAACGCGAGCAAGATCCATCCGACGACATCATTAATGATGCCTGACGTGAGAACAATGACGCCGACGTTGCTGTTGAGGAGCTTGAGCGAGGTCAGGATTCGACAGAGTACCGGAAATGCCGTGATAGCAATGGCGATGCCGATAAACAGTGCATAGACACCAAAAGAAATGGGCGCAATTCCCTGCTCGTCTTTGAACTCGTTGTATAGACCATAAGCGAGAGCGACGCCCAAGCCAAAGGGGATTGCCATGTCGAAGCTCGCGACGCTAACGGCAATGCGCCAGTTGCGCAGAAGGTAGCTGAGATCAATCTCGAGGCCGACCAGAAACAGATACAGCACCAGGCCGATGTTGGCTGCCAAGCGGAACGGCGCCATGGATGCAGGCGGGAATATAGTAGCTGTGAAGCCTGGGATGCGGCCCATGACTGAAGGTCCCAGTATAATTCCCTGGGGGTTATTGGCGCATTAGTTACAATAGACACACTATGGGTACTACACACAAGCTTTTTCGTTGTATATGGGAAGCAACTCGAGTTCCGGGTAGAATGCTTACCGTGATGACTTCCGCAATGACCTTTGGCTCGTGTATCTTGGCCAGAGGCCAGTACAGAAGCCGTGTCAACCCAATGACGATCGTAGCTTGGATTATGAAGAGTACAATCGGGTCTGAAGGATTGAACACTGTTGGGTTCATGCCCTCAAAGATGCCGGCCTGAGACGCTGCCGTGGCGACAGGCGCCGCAGTTATTGTTGCAGCAGACATTTTGACTACTCTCTTGTGATGCAGACCATTAACATAAATATTGAAGATGGATAGTTGCAGCTAGCTTCGTTGCTCCGTTCAAATTCGCGGTCTTGGGTATGTCAAGCGCACTTTTGCCCCGACGTGAGCGCCAGCCACTGTTGTCGCCAAAGGAGGCTAGCAATCTAGAGCAGAAGAGAGGGACAGGAAGGTCAAGGATGCGCTGGCGCAGGAGGGCAATCCTTCAACGGCGGGCAGCCTTGCCCTTGTCGGCTGGAATGTACTCTTCGTCGGTCAACACAACAGCAGCAGGTTCGCAAGC

CAGCTGCAAAGCCCAAGAATGGCAAAGCGTCTTGGCATGATCGTAAGGGTCCTGGTTTGGACTGTGTGACGGTCAGCAAAGCCACATTTCGACCCTGGTGGTAGGAGTGAAGCCGCATGCATAGATGTGTGTGTGTGTGTGTGTGTGTGTGTGTGTGTGTGTGTGTGTGTGTGGGTGGGTGGCCTTGGCATTCAGAAGCGTTTTTGAAATCGAGCCTATTGGTTGCTCGACATTGGCTGCAACGATAAGACGTTGGATTTTAGTTTTGTCGGCGTCAAACGTTGATGCAGACCGCTTGGG

GTTTACAGGACCCTTGATGCAGTTTGGATGCCGTCTGAATGATCAGATGACTTCTAACCCAAGCTCTCCACTACCGAAGGTCTTTTGCCCAGAGCCAATTGCGCTAGCAGGCAGCCCCTCCCTCACCTTCCCGATCTTTCAACCCTGGCTAGCACCTTGGCCATCTATCTGCCCAGGTAGCTATGGGGAGCTTCGGCCAATCACATCCTTCTCTCGCGTCAGCAGCTCCCGTCACCTCGGAAGCAACCATAGGTTCATCGATACCTGGACACCTTCCAGTGATCCTCCTGCATGCACGCATTCTCACCGGAGCTTCTGTCCCGGTCATCAACGCCAGGACGATAACACAGCAACCAAGGAAGTAAGTACCTATACAGGTACCTTAGGTAGCCACAACATCACCAACCCGAGCCTTTGACCGTCCGCAACACGAATGATGTTGTCTGCAGAACCTATAAATCTGTACCTTGACCCAACCTGGGCCCATAAGTTCTCCAAATTACGAGACTGATTGCCGAGCCGAGATGCTTTGAGATTAATACCCTTCAAGATATGCCGTTACTACCCATACCAGCTTGACTGGGTGCGAACTACTAGCAAAACCCCCTTTCCTGCACTGTCGGGAATACATCTCCAACAACTGTTCAACATACAGTACTACTAGGTAGCCCAGTGTTCCCAGCGTCTGGCAGCCAGACCACGAGACAGGCTGGGTTTCGAGGCTTAGAACACTGGCACTGGATTGTACTCATAGTGTCCTCCCTATTGTCCCATTGCACCGGTGCAGAACCTAGCATTTCCAAGGGGAAGGAGATGGCTGTTACGGCGATGTTACTTTTCGACCAAATGCCCCGAGCGACTGAGAGCGAGAGTCGGGATCTTGCGAGACTGTAGAAGCACGCAACAAAGACCTATGGCGAGGGAGACAGAATGGATAGAAAGCCCAAGGATTTACCTTGCTAATAAAAGAATAATTGGAAGAAGACTTGATGATTTAGTTTACAGAAGCAAACGGCGATTGCAAAATTCTCTGTCTGCATCAGCATTTCTATGCTAATGATAATCCCCTGCCAATGATAATAAAAGCTAAAACAGTCTTATGGAGTTGATGCTCTCTCGATCTCTCACCTCCCTCACCATTCCCCACCACGCTCAACACGGTACCTGGACAGGTTCTCCGTCGGGGCGGAGGATCTACTTCCGCTGTTGCTCTGCATCAAAGTCAAAGATCTTCATGAACTTGGCCGAAGTGAGGATGGCCAATCGCAGAAACTCATCTCCAAGGCATCCCGTCGTAGCAGAGCCCAGCAGCTGCAGTTGGTAAAACTCGATGGTCGGGTCGAGCGTGATGGCGCCCATCGTCCAGCGGGCAATCAGCTCGGGTTCGTGGCCGTCCAGGTTTCGATACAGCTCATTGTGGTCCTTGCCGACGCGCCACATCATCGGCGTCGACATCTTGACGGGCTCTGTATCGCGTCCAATCGGCAGCTGGAACCAGTGAGCGCGGCAGTGTTTGCGGTGCCGGCCGCAGTAGGAAGGGAGGTCGAAGCCGACGTAGGTGGGCGGGTCGATGCGCGCGCGGATCTTCTCGTAAACCCAAGCTTTCTTGTCAGGGAAATATGTAGGACGGACGCTGACCCGCACGTCCTTGTTGTTCTTGCCGCAGGCGCGCATGGTGGCGACAGGGGTGGTCATGCCGAGCTCTTGAAAGAACGGAGACTCCTCGTACAGGGCGAGTACCGAGTGGCGCTTCTAAAGCCAGCCGTGGTGCTCGTGCCGAAGGATGAAGAGAGGCTCTGCACCCATGCGTTCTCTCAGAGACCAA

**> MD304_chr03:1420968-1424968**

GACGGGTGGCAATATCCTTCTTGTCGAGGGCACCTGCGGCACCGGTCTCCTCTTCTTCGGATGGATGTATCGGGTTTCCATCCCAATCAATTTTGCCTTGTTTCCATAGTTCAAGCTTTTGCTGATATGAAGGAGGGTATAGCCACATGACGAGGGGCGAAGTCAAAAATGTCGTAACAAGAGCCATGACGACGAAGATTGTAAAGGTTCTGGTCGAGAGAATGCGAGCCTCCCGGCCGATGTTGAGGACAATAAGTTCGACGAGACCCTTGCACGACATCAGCGTACCGATGGTAAAGGACTCGCGCCACACGAGACCGTTCAGCCTGGCACCTATGGTACCGCCGATGAGTTTGCTGAAGAAGGCGACAAAGATGACGGCAAAGACATAAGCCCAAATCCTGCCACTATCCAAAAGGCCAAGGTTCGTGTTGATACCAGAGAGCGCAAAGAAAAGAGGGACAAAGATGACAGCAACCAGGTCTTCAATCTTTTCTGTCAGCTTGACGGCGAAACCACCCTGATGGGGGCACATGAGACCGACCATAAAGGCACCAAAGATGGAGTGCACGCCGATGATGCTGGTAAAGAAGGCAGAGGCAAGAACCATAAATATGGTCAGAGCCACCACACCTTGAGTTGGGCCATTTTCCAAACTGTGCGTCTTGTGCAGCACCCACATGAATGCCGGTCGGACTGCATAGGCCAAGAACAAGCTGAACCCGACGGCGACCAGGAGAATATATAGGGCTGTGATGCCGGCGCCAGAATTGACGAGAGTGACGCACAACGCGAGCAAGATCCATCCGACGACATCATTAATGATGCCTGACGTGAGAACAATGACGCCGACGTTGCTGTTGAGGAGCTTGAGCGAGGTCAGGATTCGACAGAGTACCGGAAATGCCGTGATAGCAATGGCGATGCCGATAAACAGTGCATAGACACCAAAAGAAATGGGCGCAATTCCCTGCTCGTCTTTGAACTCGTTGTATAGACCATAAGCGAGAGCGACGCCCAAGCCAAAGGGGATTGCCATGTCGAAGCTCGCGACGCTAACGGCAATGCGCCAGTTGCGCAGAAGGTAGCTGAGATCAATCTCGAGGCCGACCAGAAACAGATACAGCACCAGGCCGATGTTGGCTGCCAAGCGGAACGGCGCCATGGATGCAGGCGGGAATATAGTAGCTGTGAAGCCTGGGATGCGGCCCATGACTGAAGGTCCCAGTATAATTCCCTGGGGGTTATTGGCGCATTAGTTACAATAGACACACTATGGGTACTACACACAAGCTTTTTCGTTGTATATGGGAAGCAACTCGAGTTCCGGGTAGAATGCTTACCGTGATGACTTCCGCAATGACCTTTGGCTCGTGTATCTTGGCCAGAGGCCAGTACAGAAGCCGTGTCAACCCAATGACGATCGTAGCTTGGATTATGAAGAGTACAATCGGGTCTGAAGGATTGAACACTGTTGGGTTCATGCCCTCAAAGATGCCGGCCTGAGACGCTGCCGTGGCGACAGGCGCCGCAGTTATTGTTGCAGCAGACATTTTGACTACTCTCTTGTGATGCAGACCATTAACATAAATATTGAAGATGGATAGTTGCAGCTAGCTTCGTTGCTCCGTTCAAATTCGCGGTCTTGGGTATGTCAAGCGCACTTTTGCCCCGACGTGAGCGCCAGCCACTGTTGTCGCCAAAGGAGGCTAGCAATCTAGAGCAGAAGAGAGGGACAGGAAGGTCAAGGATGCGCTGGCGCAGGAGGGCAATCCTTCAACGGCGGGCAGCCTTGCCCTTGTCGGCTGGAATGTACTCTTCGTCGGTCAACACAACAGCAGCAGGTTCGCAAGCCAGCTGCAAAGCCCAAGAATG

GCAAAGCGTCTTGGCATGATCGTAAGGGTCCTGGTTTGGACTGTGTGACGGTCAGCAAAGCCACATTTCGACCCTGGTGGTAGGAGTGAAGCCGCATGCATAGATGTGTGTGTGTGTGTGTGTGTGTGTGTGTGTGTGTGTGTGTGTGTGTGGGTGGGTGGCCTTGGCATTCAGAAGCGTTTTTGAAATCGAGCCTATTGGTTGCTCGACATTGGCTGCAACGATAAGACGTTGGATTTTAGTTTTGTCGGCGTCAAACGTTGATGCAGACCGCTTGGGGTTTACAGGACCCTTGATGCA

GTTTGGATGCCGTCTGAATGATCAGATGACTTCTAACCCAAGCTCTCCACTACCGAAGGTCTTTTGCCCAGAGCCAATTGCGCTAGCAGGCAGCCCCTCCCTCACCTTCCCGATCTTTCAACCCTGGCTAGCACCTTGGCCATCTATCTGCCCAGGTAGCTATGGGGAGCTTCGGCCAATCACATCCTTCTCTCGCGTCAGCAGCTCCCGTCACCTCGGAAGCAACCATAGGTTCATCGATACCTGGACACCTTCCAGTGATCCTCCTGCATGCACGCATTCTCACCGGAGCTTCTGTCCCGGTCATCAACGCCAGGACGATAACACAGCAACCAAGGAAGTAAGTACCTATACAGGTACCTTAGGTAGCCACAACATCACCAACCCGAGCCTTTGACCGTCCGCAACACGAATGATGTTGTCTGCAGAACCTATAAATCTGTACCTTGACCCAACCTGGGCCCATAAGTTCTCCAAATTACGAGACTGATTGCCGAGCCGAGATGCTTTGAGATTAATACCCTTCAAGATATGCCGTTACTACCCATACCAGCTTGACTGGGTGCGAACTACTAGCAAAACCCCCTTTCCTGCACTGTCGGGAATACATCTCCAACAACTGTTCAACATACAGTACTACTAGGTAGCCCAGTGTTCCCAGCGTCTGGCAGCCAGACCACGAGACAGGCTGGGTTTCGAGGCTTAGAACACTGGCACTGGATTGTACTCATAGTGTCCTCCCTATTGTCCCATTGCACCGGTGCAGAACCTAGCATTTCCAAGGGGAAGGAGATGGCTGTTACGGCGATGTTACTTTTCGACCAAATGCCCCGAGCGACTGAGAGCGAGAGTCGGGATCTTGCGAGACTGTAGAAGCACGCAACAAAGACCTATGGCGAGGGAGACAGAATGGATAGAAAGCCCAAGGATTTACCTTGCTAATAAAAGAATAATTGGAAGAAGACTTGATGATTTAGTTTACAGAAGCAAACGGCGATTGCAAAATTCTCTGTCTGCATCAGCATTTCTATGCTAATGATAATCCCCTGCCAATGATAATAAAAGCTAAAACAGTCTTATGGAGTTGATGCTCTCTCGATCTCTCACCTCCCTCACCATTCCCCACCACGCTCAACACGGTACCTGGACAGGTTCTCCGTCGGGGCGGAGGATCTACTTCCGCTGTTGCTCTGCATCAAAGTCAAAGATCTTCATGAACTTGGCCGAAGTGAGGATGGCCAATCGCAGAAACTCATCTCCAAGGCATCCCGTCGTAGCAGAGCCCAGCAGCTGCAGTTGGTAAAACTCGATGGTCGGGTCGAGCGTGATGGCGCCCATCGTCCAGCGGGCAATCAGCTCGGGTTCGTGGCCGTCCAGGTTTCGATACAGCTCATTGTGGTCCTTGCCGACGCGCCACATCATCGGCGTCGACATCTTGACGGGCTCTGTATCGCGTCCAATCGGCAGCTGGAACCAGTGAGCGCGGCAGTGTTTGCGGTGCCGGCCGCAGTAGGAAGGGAGGTCGAAGCCGACGTAGGTGGGCGGGTCGATGCGCGCGCGGATCTTCTCGTAAACCCAAGCTTTCTTGTCAGGGAAATATGTAGGACGGACGCTGACCCGCACGTCCTTGTTGTTCTTGCCGCAGGCGCGCATGGTGGCGACAGGGGTGGTCATGCCGAGCTCTTGAAAGAACGGAGACTCCTCGTACAGGGCGAGTACCGAGTGGCGCTTCTAAAGCCAGCCGTGGTGCTCGTGCCGAAGGATGAAGAGAGGCTCTGCACCCATGCGTTCTCTCAGAGACCAAACCTCTCCCTTCTCCTGAGGC

**> MD306_chr03:1909873-1913873**

TATTTTATTTTTTCTGCTGGCACATGTCGAATCTATTTTCGCACCACCGTATTCTGGTGTGTCGATTTGACGTCGTATCGATTGACACTGTCTTGCTGAATCGTCTGCCATGGTAAAGATTGGAGATTTTTCGGTGGTGATAATCACACTTACATCCTTATAAGTAGATGCTTTGTACCCACGAAGACAAAGGAAGGGGTTTCTCTCCCAACATCAACAATCCAAAGCTTCTCAATAAAAAAAGCCATCTATCTACCTTTGCACCTTTGCTCCATTCCGGTCGAATAACCGACACCCTTGGCTCAAAATGGGTTGTAAGTTTAGTTTTGTATCTGGAAGCGACCTAACAAATACCCGACCTAGTCTATAGATGGACACGGGAGGAACGCCACCGCTAACTTGTGATCCCAAGTCTTGGAGACGTCGTTCCCCCTTAGCCACAAGCACGCCATCCCCGCGGGAGTGGACAAGGCGACGGCCATTGCGAAGCTGCACGACCACATCTACATCATCGACTGCGGACCCTACGTGACGTCTCGCACCCCACAGCCATCCCCGGCATGGGACTCGTACGACAAGCCCAAGGGCGTCACGTTCGCGTCGGAAAAGGTCGATGTCCACGAGGTCAGGAACAAGTACGAGAACCCCATCATGGGCTCCGACATCAAGAGCACCTACTACTTCATCGATACCACCGAGGGCGTCTTCATCCGCGTCCACAGCCCCATGGGCACGGTCATTGAGAGCATCTTCACGGTGAAGGAGAAGAGCGACGGCAGTCTGGAGCTCTGCCATGAGCTTCAGGGAAGGTGCAACAAGGCCCTGGCCGGTATCTGCAAGAAGGATGCGGACAAGAACTATGAAATGCTTGTCGGCATAATTGCGCAGAAAATGGTGGCTTGAGAAGTATAACAATGGGAAAGCGAGCTTGTGCATGAAAGTCGAATCGCTGTCTGTCAACATTGGGGGTTGTCAGGTAGTAGTATCTTCAATTATTTAGCACCGCTAGAAATTGTGCAGAGAGTATTCAAGTTGGGATTTATCTAAATTGTGCAAAACATCATGAACTGCATACCTACCCTGCCTAGCTACCTGACCGGATGACTGGTTTTGTAAATCCTTGTTCGTGTCTATTTACATGCCAGACTATTCTATCTCGTTTGCATCTTGACCTTATCTGCTTTTGACATCTATAATCTCCGTATCTGATTTCCACACCAGATTGGCACTCGAACTGAAGGCCTTGGCCTAACAGTGTGTCCGCAGCAAAAAGAAAAATAAAAGAAATATATAGAAAAAAAAGAGAGAAACTTTGCTGGTATTACACTATATAGGGCTTGGAGGGTCAAGGATCAGAATGGAAGAATAAAGTCAATGGCTATATGATTACGTCACATTGGTCACGGCAAGGTGAGGTGTGAACCTGTTGAAAGCTGCTATGATTGATAATCGCACCACAAATGAGATGACAGCAAGCCACGGACCACTACCTAGAATATTAAAGTTTCAAGCCGCCGACTGGATCGCAAGGTAAAAGTCTTGGTTGGTTATAGAGAGTTTTGAGATGCATACGGGCCTCCACATGAAAGAACTAAACGGGCACGATAGCATGGCAGCAAGTGCCGGAAGCACGGGACTGCTATTTTTGATATCCTATTCTTCATCATCATCAGATCATCTTCACAAGGAAAAGAAATGAGCAAAGTTTGATGGATGACCTCTCGGGTTCTTTATCAACTCCGCACTACCACTATCATCTTTTGAGGTGCTCCAGCCCATTGTTAAAATTCAATCATGGGTATGGACCTTTGAAGAGTTCGGCTGAAACGCTGTTTAGCCCAGGCAGAGCTCAGGGCGGAGCATGTCTTCCAAGAA

ATCGTCCGTTGGGAAGGAGCGGACAACCGGTGAGTAACAAGAGTGCGACGACAGTCTCTCGCTAAATATTTAGGGGAGGACGCCTGTACTGCAGTTCGGTAGCTTCAGTCAAGCACGGCAGCACCAGCAAGAAAGCAAGAAAGCAAGAAA

GCAAGAAAGCAAGCAAGCCAGCAAGCCAGCAAATATTACCCTGACACTTCAACTCAAGCACAACAGCGGACCAAAATGTTCAACTTAATTTCCAGTCTCAAAGTTGGAGCGGCCCTCGTTGCAGCCGTCTGGATATGGCAGCGAATCATGCTGTACAACCACCGGCGCAAAATCAAACGGGATTCCGGATGTCTTCCTGCTGCCAAATTTCCAGACTGGGACCCGCTTTTCGGCATTGGATTCACTTACCACATGGTGACAGAGGATATATTAGACTTCCAAATCATGAAAAACACGGCAGACTGCCTTGAAAAATACGGCAATACCTTTACGTCAAAGGCCATGGGGTTCAACTTTATTTGGACTTGCGAACCCGAAAACATCAAAGCTGTCCTTTCCACTCAGTTTGACCAGTTTAACAACCAGGGCCGCTCCGTGGGCGCCGAGGACTTTTTGGGCAAGGGGATTTTCGTGTCGGACGGCGAGCATTGGCAAAAATCACGGGCTTTGGTTCGGCCGAACTTTGCTCGGGACCAAATCACCGACCTTGTATCTGTGGAGGAGCGCTTCCAAACGCTGATTTCGCTGCTCCCAAAGGATGGATCGACCGTCGACCTTCTTCCCTTCATGTTCGACTTTACCATGGACACCTCGACCCACTTTTTGTTTGGGGACTCGGTGAAATCCCTCGAGGCCAACGCCCACGAGAGGTCCATAGGTCACCACGGCCAAGAGAACCTGGATGAGTTCTCCGCCGCCTGGGACTATGCGCAAAACGACATGCTGTCCCGCTTCGTCCTCGGCCCGCTTCGGTACGTCTACAAGGAGAAGAAGGCCGCACGCTGCATCAAGCTGGTGCAGGATCACGTCGAGAAATACGTCGACGAGGCGATACGGTACAACAGCCAGGTGCGCGCTGCGGGCGGCGACGAGAAGAAGGAGCCAGAAGAGCGCTACATCTTTCTCCGCGCGCTGGCGAAGCAGACCCAGGACCGACGGATGCTGCGGGACGAGCTGCTCAACATCCTCGTGGCCGGTCGAGACACCACGGCCTCGCTGCTGAGTAATATGCTGCATACGATGGCCAATCGACCGGATATATGGACCAAGCTGAAAAACGAGGTCGCCTTTCTTCAAGGCAGGATACCTACCTACGAGGACATTCGCAATATGAAGTATATGCGGTGGTGCATCAACGAATGTAAGACTTGACCGTGCAATGAGCCATGATCTCATCCGATACCAAAAAAAGGAAAGACAAAAAAGCTAACACCAACCCGTGTCAAAAGCCCTGCGCCTCCACACCGTGCTCGCCCTGGTGGGAAAAGAAGCATCCATAGACACGACGCTGCCCCGCGGCGGCGGCCCGGACCACAAGGCGCCCATATTCGTGGCCAAGGGGACGTTTATCGCGTACAGCTTCCTCGCGCTGCACCGGCGCAAGGACCTGTTTGGAGAAGACGCCCACGAGTTCAAGCCCGAGAGGTGGGAGACGCTGCGCCCGGGCTGGAACTACCTGCCCTTCAACGGCGGGCCGAGGATCTGCGTCGGCCAGCAGTACGCCCTGACCGAGGCCCAGTACGTCTTGATCCGCTTCCTGCAGACCTTTTCCGCCATCGAGAGCCGCGACCCCGAGCCCTGGAGCATCGGGAATCGCATGACGACGTGCCCGAAGAATGGAGTGCAGGTTTCTCTCCGGTGCTAAGAAAGGGGGGTACTTTCGCCTGGGGCGGGGGACAGGGCTTGGTATGTGTGAACTTCTGAATCTGGGCTGGATGCGACTGCTTTTACTATGCGGAGCAGCTGAGCCTACGTTGATTGCACTGGGTTCACATCTGCTTGTCAAGGTGTGCACTTTGGACAGGATCACCGTAGTCTCGGGTGGAGTTGGAAGTTTCGATAACTCCCAATGCAGGACAATTTTGTGCCGCATCCACCAGCTTTAGTCCAGCTTCTACCTTTCAGCAGGCGACAG

**> MD307_chr03:2058125-2062125**

CCAGCTTGTGACGCCAAAACCAAGCGAGCCTCAAAGCGGAGGAAGAAGCTACGGAAACGGTGCCTTTGGTACGGACTCACTGTCACCTGAATTGATCCAATTGCCAATCGTGAAACTCGCAACCCAACGATTCGACAAAAGAGAATCCTGCACTTAGCTCGTCGTCCCCTCACACAATGCGTTGACCCCCAATCGGCTCAAGCCACCAGCACACCCAGCAATGCCCGGTCCGAGAGCTTCGCTGCCGAGCCTCAGCGCCGTCGACAATGTGTGCTGGCGGTGTTGCAGCAGCAGCAGCGGCCGGCCGGTGCCCGGGCAGAGATTGAGAGGGTTCGCAATCGCAAACCAGGCCCGGCCCCTGTCGACCTCGCCCGTCCGTCCGCTCCGTTTCGGCGCCACAACAACCGTTAGGGCAGCAGCATCTCAGTCGCCGAGGCCGAATGGAGCATACTTTTTATCAAATGTGCTACTGGAGAGGTTCGGTGGCTTCCAGTTCAGCCCTCGATCTAGAGCCATAGCCGGCACATCCGGGTCAAACATATCAAATACAAAGAGAAATGCCACCGTCGCCGCCGCCGACATACCGCCCTCTACGTCGACGCCACGCCCGCAAGTCGATGAAGAGCTCCCTCCCCACCGACGACGACAAGCTGCGCGTCGCACTGCCGAGCAGGCGGCTTCTGCTTCTTCGAATGCACCCTCAGAGGCTGCACAAACTACCCCAGTAACACCACCGCCTGCTGCGCCGTCAGGCGAGATCCCTCCGGATGCATCCAGTATCCTCTCCAACGCAGCGGCTGCCCAGCCCGCACAGTCTCTCCGCCGCCGCCTGACGACACTCCTCGCGCTGTCGAAGCCCCGACTCACGATGCTTGTTGTCCTCTCGGCGATGGTTCCCTACGCCCTGTACCCGGTGCCGGACTTTTTGACCCCCGGAGTGTCGGCACCGTCCCTCTCGCCCCTGACGCTGCTCTTCCTGACGACCGGAACAACCCTCTGCTCCGCTGCCGCCAACGCGCTCAACATGATCTACGAGCCCAAGACGGACGCCCTGATGAGCAGGACGAGGACGAGGCCGCTGGTCAGGAACCTGGTGACGACGCGCGCCGCCGTCTGCTTCGCCCTCTTTTGCGCGACGACGGGCATCCTCGCCCTGCAATTTGGCGTCAACCCGACCGTCGCCTTCCTGGGCGCCGCCAACATCGTCCTCTACGCGGGCATCTACACGCCGCTCAAGCGCGTCTCGGCCCTGAACACGTGGGTCGGCGCCGTGGTGGGCGGCATCCCGCCCCTGATGGGCTGGGCCGCCGCGGCGGGCGAGTCGGCCGTGGGCGACGGGTCGTGGCGCGAGCTGCTCCTCGCCCCCGACGGCAGCAGCGCCGGCGGCTGGCTGTTCGCCGCGCTGCTCTTCACCTGGCAGTTCCCGCACTTCATGGCGCTGTCGTGGGGCGTCCGCGACGAGTACCGCGCCGCCGGCCTGCGCATGCTCGCCTGGACCAACCCGGCCCGCAACGCGCGCGTCGCCCTCCGCTACGGCCTCGTCTTCGTCCCGCTGTGCGTCGGCCTCTGCGCCGTCGGCGTCACCGAGTGGTCCTTTGCCGTCACCTCGCTGCCCGTCAACCTCTGGCTCGCCCGCGAGAGCATCCGCTTCTGGCAGACGCAGGGCGCCGCCGGCTCCGCCCGCGGTCTCTTTTGGGCCAGCGTCTGGCACCTGCCCGTCGTCATGGTCCTCGCCCTGCTGCAGAAGAAGGGCATGTGGGGCCGCGTCTGGAGGAGCGTCTTTGGCGAACCTGACCTCGA

CGACGACCTTGCCAGCGACGACGGCTGGGAGTACCTCGATGAGAGTGAGGAGCCCCCGGCTGTGACAACACGGTCGTGAGGGTTCCTATCTGCTGGATCGAGATGCCTTATTTTGCGTGTACTTGTCCCACGGAGTAACAAGACTCGGAACAAGGAATGAATCACACAGTGGCCATCTAACGAAAGAACAAAACCGCTGGGTGTGTGTGTGTGTGTGTGTGTGTG

TGTGTGTGTGTGTGTGTTCGGGGAGATAGGAGGAAGGAAGCGCAAAGGGGCACACGAGGTGGCGTCTCTTGCATTGGTTTGCTACACGCATCCGATAAAGGTCATGGTGCCACCATATTATTGGGGTTCAAGAGGCCGATCTGTGGATCAGGAGAGAGCTATTTGCTTTGTTCCCTTGTCACTTTGTTGTATTTTTTTCAAGCATATAGAGTGTAAAATTGTACAACACAATATCCCTCGTTGTTTCTTTTTTTAAATCATTTCTGCAGATGTTTAACCAGCCAGCCAGATACCTACCGTCCTGCCCCTCGTTTCGGTCGAGCAGATCACGCACTATCAGACACATATTGCATCGCTTGCCGCCGCAAGGGACAGTCTAACAATGCCCAGCGTCTAGGTCGAACCGCGTCGTTTTCATGTGTCATATGCTCCGGAAAGGCTGGGTAGCGCCTGTTCTCATGAGCGGAAATACCGCACGAGTAGCAGCGCACCAGAGGATACAGATCTGATATATAAGTACCAAAGAGAAGCCAAATCAGATTTCCACCAACTTCTAGTCTAGGCTCTATTTTTCATCGCGGTGAAAATATCAAAGCTATCGTTGCACGTTTTTCTTTCATGCCAATTTGCCACCAATTGTTACGGGCGCAGTCCTTATATGCCCAAATTTCCATGCCTTGATCCTGCCAGTCCGTGGGGGGTATCTCGAATATGCAAAAGTTCCCGCTGTCCTCATGCCGACAAATGAAAGGGGTTTCACGAGAAAGTGTCGTTTTTGAAAAGAAAATAAGAGAAGACACCCATGCTAAGAAAGAAAGGAGAAAACAGGTCGAAAGTCGTTAGCATATCAAAGTCATGAACGCCGGTCTTATGCAATGATGACAGACTTGGAGAGTCAGAAACAGACGAGATGAGATGCAATGAGATCAGAAAGAAAAATGGAATGAAGAGAGAAAAAAAAAGTTGAACAATTATAAGAGATAGAGCCACCGTAAAGTGTACTCTAGACTGCAGAGAGCGGAAGAAGAGAAACCAAGAAAAACTAGAGTAAAAAACATGAGCGTAAAGAAAATCTCAAGAGTTAACCCCCAGATACCAATATACATAAGGAGTGAGGCCAATGTAAAACCCCAAGCCGCCACCAGAAACGCCATACCACCAGAGTGAACGTGAGAAATAAAGATGTAAAAAGTGCCCGGCCTGTTACCAGTAAAATGTTGCTGGTAACCATCAAGCGAAAGAGAAAGAGGGGCTGCGCCCATCTTTCAAAAGGAGAGTAGGAAGAGGAATTAAAGAAGAAAAAAAGAAACCATAAAAGCTAAAAGAAGCGAGGCGTTAAAAAAATAAGGTGGGGATATCATGTGAAAGAAAAAGGAATGTCGTCTGGATGCGCCAAAGTGGGTGGTCATCCAAGAGTAAAAAGGGTAAGAATAAAAATCAAGAAACAAAAGTAGAAAGAGATTTGAGACAATAAGGAATTCAGGAGCCAAAAGACTCTCTCACACTTCCGTGTTGTTTCGGGTCAGGTGCGGGCCAGTAAAAGTGGCCGACTTAGACGCCGTGGTGTCCAGATCCGGAGCCCCGATGAATCAGGGAGCCAATCCCCATCTCCACACTGGGTAACCTCTGCCCATGCCCCAAATACGGGCTAGGGCTATGACTATCCTCTGCAGGGTTCTGGGTATAACCTGCAGTGTGTGTACTTGATACAGAGTTGCTGTATCCATGATGGTGTTGGTGCTGGTGTTGGTTGTGGCTAACGTAGTAGCCATTATGGTTTGCGTATGCAGTTGTGTTGGGATGATGCATGGCCGCCGTCACGGCACCGTATGCACTACCTCCAGTGCTGCCACCAGCACTGATACCTGCAGACGACGGTGAGGAATATTGGAGTTCCTGATCATCCAGGGGGGTCATTCCTGAAACTCCGCTATCGTCATCGTAAACTGCGCTCGAGTGGCCGGCGTCGGCACGC

**> MD308_chr03:2061753-2065753**

CCCATCTCCACACTGGGTAACCTCTGCCCATGCCCCAAATACGGGCTAGGGCTATGACTATCCTCTGCAGGGTTCTGGGTATAACCTGCAGTGTGTGTACTTGATACAGAGTTGCTGTATCCATGATGGTGTTGGTGCTGGTGTTGGTTGTGGCTAACGTAGTAGCCATTATGGTTTGCGTATGCAGTTGTGTTGGGATGATGCATGGCCGCCGTCACGGCACCGTATGCACTACCTCCAGTGCTGCCACCAGCACTGATACCTGCAGACGACGGTGAGGAATATTGGAGTTCCTGATCATCCAGGGGGGTCATTCCTGAAACTCCGCTATCGTCATCGTAAACTGCGCTCGAGTGGCCGGCGTCGGCACGCTCACGTCGAGCCGCATGGCGAGCAGCCGACTGCAGGTTTTTGATGCCGTTGGCCATGATCTGTGCGAGGTGTCATGCCTTTCATTAGTGTCAATGGCCCTCTGATGTTTTGAACCAGATATTTCTTGCGTACCTTTTGCTCAACCACATTCCACTTTTCGCCAGTGATGCGTGCCAGTGGCTGCCAAATCTCCTTGCGAACCTTCATGTACTCCTGGGCCAGGCGCTCCAACTTGCGCGCATCCCAATCATCGGCACCCCTGCGCTCCATCAGACGCTCATGGCGTTTGCGGCACGCGTTGGAGGTCTTGGAGGGAAAGTGAGTGGACTGGATGCTGGACCAGTTCAACCCCTGTGCGCGAGCGGCAAGGAGAGTCTGGTCCTCCTCAGCGCTCCATGCTCCGGAAGAGGCTCTGTGTGGGTGAGAAGGGGTTGGTAGTCCATGGCTGAAGTTGTTCCCAGGGATACTGATGGGCGGTGGTGGCGGCGGTGCAACAGCGTATGGGTCGAAAGAAGGCAGCGTCATGGGAGGCTGCGTGTGGGTTGCCGGCAATGGCTCGTAATATGAAGCAGCCGCAGCCATATTACTGTTACTCCTCCCGTTGTAGTATGCCATTTGTGAGGTGGTGGTGGATATGCTGGCCGGCATTGCGAGAGGATTGGCTGTGTATCGGAGGTGGTTATGCGATTGTCGCGTGATTTTGGGCATGCTAAGTAGGATTCCGTGGTTTGGCACTCTCGTCTTAACGAGTGTGACCCAGCAGGGAGAGATGCGACATATGATTTGGAGGTGTCAGACCCGAATACGAGTGAACGGTAGATAAAGAACTATGAGTGAGCGTCTTGACGGGTGTGGGTTGAGGAAAAGTCGACCGTGAATGAAACGAGGAACGGTCAGGCCAGGCTCGATTTATGGCTCTGATGGGTTAGGTGTTGGAGATATCGAATGTCTCTTGGACGGTTTGTTTTAAGAGAGAAAGTGGAACGAAAAGAAAGTTATAAACAAACCACGAGGCACATATAGAGTTTGATATATCAGTTTGTCAAGTTCATCTGGATGTCACAAGGTGAGACGGCTGGAGAAGTGATCACGGCGATGCAGTGACTTATAAGTGAGATTGGGTGACAGGGAGGAAAATCCAAAAGTAGAAACGGCATCGATCTGGCTTGTGGATCCCCCCGGCTGCAGGCACAACTGTGTAGGGCCAGAGCAAGGTGAAGACGCCGTGTAGTTGATCTGAAACAAGGATACATCTAAGCTGGGCTTGTCTGCTGAAACCCGGTAGGTGTGTCTAAACAAAGAGATTGAACGGCTTTGAGCCGCGCACATTGGCTGACGGCTTGTGTGGGCGTCTGTTGTCCAAGCCGGGCGGCTAGCAAGATGCCCAGCAGTGGGTCTGCCGAGAACGCGGAGCGGTAGGGCGGCTAG

CATTCTGCAAGTTGATGATCCTCGAGGGGGTTACCTTACCCTTGTCCTTGTGCTCATCTCAAATTCAGACGAACCATCAAGCTTTCTCAGACATTGGATGGCATTGCATTGTGCTAGGACCTCAGTATATTGATTAGCTGGATGTTTGCGATAACTCGTCATGTCGCAAACAACCAAAAACCCCAACGTAAACAAACGAAAAAAAAACAAAAAAAAAAACAAAAAAAAAAAAAACAATCAAGAGTAAAAGAGAAAAGAAACAATCAAAAATACTCGAAACCACAAGCCGCATCGTGAATA

GAAATTTGAGATTACAGCTTACTGCACTGCAATCAGCCACTCGGCACATCAAAACAAAAGAGAGAAAAAGGGGCAATAGAATCGGGGTGCAGGCGTAGACGCACGGCAAATGCAGGTACACACGACACCCAACGCTACAGGGTCAAAAATAGAGGCGGACAGGGGGGTGTGTGCTGGTTGGTGAGGGGGAGGGGAAATAGCCGCCAGTTACTGGCGCCGGGCAACCGCCAGCCTTGTACAGTGATGCCCACCCACCATGGATGGGCCAAGACCAAGCCTTGTTTGTTGTCATACCATACTCACTCTCTGAGGAACGGTTACATAATGTTCCGTCTTCTTTCCGGGCTAAATCAGGAGCACGATCTGAAAGCCAAGATAAGCAGACCGCAGCAAAAATAATAAGAACTTTATTGAGCTTGGTACCAATGTACCTACCTTTTTGTTTCTGTGTATTATTTCTTAGACGCAGGGTCATTCGGGTACCTATGACGGAATCTTTTAGTTCTAAATAAAACAAGTGCCCGCCCTGAGCCTCCAAGTACCTTGGGCTGCCAAAGTACCGTCTTCAAGTCGAGGTCAACACAACCCCAATGCAAATAAGCTCGTCAGGAAGCGGCGAAGGAGCTCGTCTCTGTCATGAAACAAAAGACAAAGTGCTCAGCCACTCAGATGCACTCGGTTCGGGAGTTGATGGTGGTTGTTGGCCTCGTAGGGAGACGGTAACCAAAAGAGAAAATTCGAATCTTCATTCCACGCCAAGCTCCCGACACATTGGGACTGGATTGCAGAAGCGCCATGATGGGGAAAAGAACCCACTGGTCGGCGTCTATCAAGCGCGAACTGACAAACGTAGGTAGTAAACGTACCAAAGAAACATGGCATGTCAAATCGGCAACAAACCAACAACATTCTAAACGCCGCGGTTAAAGAGCCGAGGCACGCGCGAGGCCGGGTTGTTGCGGGCGCTAGAGGGACATCACAAAGTGGATGTGGCATCATCACATCAAAGCCGGAGATGCATCGCAACATTACCGGCCTGGGGCTTTGTGGGTTCACACTGTCGCAGTACCCGCTTCAAGAGACAAAGGGACGATGAATGAAAAAGACTAAACAAGACTGGCTGCTTTGAATGGGGGCCGCTGGAAAAGTTGCTCCCGAAGCGAGGGGGCTCACAATCGATGAAGCACAAGTTAAGGAAAGGGAGGCTATGGTACGTACCTTGCCTTACTGTTGTGATGAACTTTGAAAGTGCTCCGCAACGTGGGGGCTGCTGAAAGTCAAAAAAGATGCATGCGATCAGAAGGTCTAGTGCTTTCCAGTTGTGGTGATGGCACAATCGTGTAGAAGGTAAACATGAAACGCTCCGACGGGCGCTTTCCCAAATCAAGCACTGCGTAAACATGACTCCAACGGAAAGTCCTCCCCGAACAGTGTAAAAAAATGAACAAAAAAAGATTCATTATAGGAAAGAGGTACCTAGGTAGGTAAGGTGACGGGATTTCCATAGTTGACAGGCGCGCGCAGGTCCCCTACGATTCAGCGCTTCCAAAAAGCGCACGTCAATGATTCAAAAATAATGAAGCAAGCGCTAGGGGAGAAGAAAGAGCCGCCGGTGAATGTGACGGGCGCGTGTTTTCTGCGGGCAAAGGTACGAGCCAGGTTCTGGACGCCCAAACAGGTTAAGCTTGACGCCCCTATTTTTGGCTGGTCTGTTCACGTGGAAGCGACCGTGGTGATTGGTGGGAGCTTGGGGGGCGGCGATCCACCATAGGCGATCTGGAGTTCTCATCATATTCCACGGAGCGGAGAGCAAAGACACCGAGCATCCTTGCCGATAACCTACAACCTGCTTGTTCGGCTGAGCTGCACAATGTCTTTCCTCGCTTTGGCGTACACAGTT

**> MD309_chr03:2118256-2122256**

AGCAAGAAATAAAAGTAGTGTTGCTGTAAATAGACACTGCATTTGGGGAGGATCGGTCGCACGCATAGATGTACAGAGCGTGTTTTTGTAAAGTGCATCATTGCAGCTTGGCTGCTGCTGGTCACGAGAAGGCGTTTGGTGTTTGGCAGTACAGTTTATACTACGAAAGAAAGAAAAAGAGGATTGGTTGGATGATTAACCGTATTCTCTTTGCAGATGTGCATCGTAGGTTGTGTTGCTTGCATTTGATTTTAATATATTTGTCCACGTTCTGCAGGTTTGAGGCCAACGTTGGAATTTCTCCAGCCTCTTCCTTGACCGCGTTATTGGCCATTATTGCGTACCTTGGATACTACAGTACTCAAGCATGATGCGTTGTCAGTCAACCACGGATACGTAAAAGTGCCTCGTGCCAGGGGTCCGGGCCTATCATATGTACGACAAGATAGGTAGACAGAATAGAACACGGCGAGCATCACACAGCAGGTGTCGAAGCTGGATTCCCTACCTACCCTACCCAGAAAAAAAAACCAAAAAAAAAAAAAAAACCAGGTAGCTTCGTTGTTCTGTCGAACTGCGATCAAATATTGCTTTTGCAGTCTTTGTACTACACAATCCTTCCCTGCCGAATCGGTCGAGGTGCAGCGGGAGAAACCTCGAGGTCGCTCTGAGCTGTCCACGGAGGGACAGGCGGGGGACCACGCACTTTGTCCAGGGTGTTGCCTGCCGGACTTTCCCACAAAGAATATTTTGTAAGGATATTTCTTGTTACAATGGTTCGTTCCCTTTTTGTCATAAAAAAAGGTCGGGACTTGGACAGACCGCCCTATTTGTGTGGCGGTGAAGAGCATGTTGCAGCACCTCATCAATTGCCCGCAATTCAAGGGTCGGCCTCCGACTCTGCCCTGGCACAACGTTGCAATTGCTACATACCAATGCTTTTCTTTTTTTTTATTTCCAGCGGTCGGAATCGCGGGAGAGATCAAGGGTTGCTGAATCTTAGATGGACTTGCCGCCCGGTGTGGTTTCTTTGCAAGCTTGCTGTCGCGTCTATCTCACCACACCCACCTCCAGAACGGAACGGGAGCATGACCGCTGTGTTGTTTTTCTGCATGTACAGTACCGCACCGGGCTGTCACACGACGAATGAGAGAAAAACCGCTTCGGTATTCCATTGGAGGGAAGTACCGGTCAGACCCTCAGATTAGGAGAACACGAATAATTAAAAAGAGCAAATACAACCAAAGTTGTAATGACAATTACAGTGGATGTGACGATTTCATAGACGCTGCCCGACGAATAATTGATGCGCTTCCAGCCAAGACCAAGGCTCCCTACGTCTTTACCTGTTATGCTATTACAATAACGAAGCATCTCATTTGTTTCCACCATCAATGAGAACAAGGCCCTAGAATGGCTTGCAGGGAATGATGCGCCCGTTTGGGGTTTGCGAAAGGGTGCAAGAATGATTTGTGGTTACGTGAGGCGAAAAAAAGGGGAGTGGAGAGAGGGTCCCGTCGTTGCGTTGGCAACTGGAGTAGCGTGGACGAAGCGCATTGGTCTGTCAAGTGAAAATGCGCAAGGGAGAAGGAGGGCAGGTGAGCGGGCAAGAGGGGGGTCGGAAAGGGGGATGCAAATGCAAGCCAGCTCATACAGGTGGCGACACTGGCGAGGTATCGTGGGCCTGACCTCGTTGTTGTACCCTAAAGGATATGAATAGCTTGGGTTTCTTATTTTTTTTTCTTTCTGCTTCTTCACGTCGTATCTTTTTGTTTATGTGCAGTGGTGTGTCTGGATGTG

GTAATTAATAGCTCAAATTGAGTGGCTTTGAGCTGGCTGGCTGCAGACCTGGTTAATTCATAGGGCCAACTGGAGACATTGGAGGCATTGGCATGCAGAAACGCATTCGATTGTTGCAAATTACCAAATTGATATGGTATCGCAGGCGATGCGTTGTCCGCAGCTTCTCTGACAGATTCTGCCGTGGTCATGTCAGCCCAGGGGGGGGGGGGGGGGGCATGCCCTCAAGCACAAAGTGGGTGTATGATGGATTTCAGACATTGACAGCACAAAAAAGCCGGGTTTGCCCTCAAAAGGCGG

TTGGAGAGGCAATCCAGTCCAGTCGACCTGAGTTAGCAAGGATGTAATTATGGAGTACAGCAACCACGAGGTGTCTGCATACTGGTTGGTACCTTGCCTGCTGTAAGTGCTTATTAAAGGTACATTAGGCCTGGGTAAAAGCAAGATAAGGTTGGCTCGCCTTTTCATTCCCATTACCGTCGGCGTGGCAGTTGCACAGTGGCTCCCGCCCGCCTTGCCGCCCCGAGCGGAGGGACAGGGCAGGGCACCTCGTGCTGGTGGGACCCCTACTGGAGCAACATGCCCGCTTGGCATTTGGCCTTGGGAAATACCCCAGTCAAAAGCCCAGCAAGCAAGCGGGAGCCCATTTTGGGCCTCGACAGGGATCAGTCCAGTCCAGTCTTGGGCCTTGCCGTCAACCCCACGAAAGTGCAGATGCTCGTCTTGAACAAGCATTTGAATTGAATGTTCACCAATTCCCTCATCCACTTATTACCTTACAAAGCTCCTAATCTGGAGCCCACCTGGACTCGACATTTCCTTGTGCATATCGTGCGTTTTTAGATCGATTTTTCTCTGGGTGATTTGGACAGCGTCTAGGCCCTGTACGGCACGCCTCTCTTTCGTCCCATTTGGTTACTCGGCCTTGTCAACGCGACTTTTTGGTCTGATGTTGTATTAGACCAAGCCCGACTCTGCGCGACACTCCTGCGTGACGATCGACCTAACTTTCCAGATTTCCTACCCGCACACCTAACCTACCTACGCTGCTGCTTGCTACCTGCCTAGCTACCTTGTAACCAACCCTGTCGTCGATCTCAAGTGTGCACCCTTCCCGGAGCTTCAGCGACGTGCTTGCGACCTGATTTGATTTCAAAGAGGTACCTACCTAACCTAACTTAGCTACTTAAAGTACCCTTACGTACCTACCTACCTACCTACCTACCTACCTTCCCGCCTCCGCTGCGTAACGGTTTCGGAGCTGCCTTACCTACCTACTCTGCATCCTCGTTCTCGTATACCCGGCCTGCCTGCTTGTGTAAGGTATTCACTTGAGCAAACAGAGAAACACGAAACGATTATTCCTACCTCGCACGCAGAGCAGTATCCACACCTTCAAGGTCAGCAGCCGTCGAGTCGAGGCTACCCAAACAAGTCCCTTCCTCGGGCTACACCAGCACGGGCGTGACGGCGCTGGTCGAGCCTGAGCCCGCCCGATGGTATTATTATTATGATTGCTATGCAGCGGTCTTTTTCGTCGCCCACATGCACTCAATCTGATCCTCGTAAGCTACCTGCTCTGCATCATTAGAATGCACAACGGACAGGAATAATCAGAAGATTTTATTCTCTCTTACCATGGCCCGCACCCGACGCGTTTGTTGCTTGCTAACGCATCCTCGCTCGCATCAAGAATCAAGCCCTTCCGAAGTTCGCTCCAGTTCACCGACCCCTTCATCCCCCGAGAATCATGTCGACCGCCCCGCCAAAGTGCGCAGCTTCAACGACCGAAGCCCTACGCGCACCTCTGGAAAGGCAAACATGTCGGCCAGGCGCAGCGAGGAGGTTGCTCGGTGGGCGCCCAAGTTGGAGCAGAGACCAGCACCCTGCGCAGCCCTGAAGCCTCTTGTGACGCAGGAAACCATTGCTTTACCGCAGCCTCTCGGCGAGGACGCGCTCCCGAGCAGTAGCAGTCCGCTCCAAGGCCACATCGACTTGGACGAGGACCACACCCAGAGTCGCGAATCGCCACCTTCCCGCACACGGGTTCCGCAGCTTTTCCTCCGCGATGTCGAGGAGTATACGAACCCGCACCTGGTTCCGGACAGCAACCACCTGACCCTGGAAGAGTTGGCACATCTCGTCCGCCTGAGCAAATATCAGGAGCGCAAGCGCGCTACTACACGAGTCCGGCTGCAAC

**> MD310_chr03:2460713-2464713**

GACCCAGCCTTTCGAGCTCGGCCTTGACAGCTCCAACAAGCTTCTCGCACTGCTCCCTGACCCGACAGAACGCAATGACGCGAACTCCTCGTAGGACGAGCTGGCAGAAAAGCCGCGCGCATTCGTACTTGGCATCGCCTCGCCCACTTGAGGGATCTCCAGGGACGCGGTAGGGCGTATTCCAGCAAAGGAACTCCTTACGGCCAGATGGAGAGCCGTCAAAATCCACCAGCCGCACATCGCTGATCCCAAATATTGTCTTGAAGTGCTCCCTGGGGTTTGCCACGGTAGCGGAGCATGATACAAACTTCACATGTCGGTTGCCAACAGCAGCGCAGATTCGGCGTAGGCGGCGCATGATGAGCGCGACATGAGAGCCCATCAGACCATTGTAATAGTGAAGCTCGTCCACCACCACGTACCGAAGATTTTTGAGAAAAGTCCGCCACCGCTCCTCTTGCGGGAGAATAGTAATATGCAGCATGTCTGGGTTCGTAAAGATGATGCGCGCCTCATCACGGATCAGGTTTCTCGCCGTCATTGGTGTGTCGCCATCAAAGGTGTCCACGATCAGGTGCTCCAGACCCGGCATGTATGCCAACATCTCCTTGAGGCTCCGCTTTTGATCCTGAGCCAGCGCTTTTGTAGGGAAGATATACATTGCCCTGGTCTCGTGGTCCTTTTCCAGCGCATGCAATACGGGAAGTTGGTAGATGAGCGACTTGCCCGAACTCGTCGAGGTGGCCACAACAACATGATGGCCATCTATCAACCCGTTGATTGCCTCTGTCTGGTGTGCATAGAACTGCGTTATCCCTTTTGCGTTGTACAGGGCATTGACGAGATCTTGGCTGAGCAGGAAATTGAGGTCTCCAAACACGGGCTCCTGCGGTTCAAAGACACGGTGGCCATCCGGAACAATTTGTCCGGTATAGAAAGGGCTCTCCTTGAGCTCCTGAACTATCTCTGGGATTGACTTGCGTTCGTTGGGTATGCTTTCTGGTATTGTACTGGTTTCCGGCGGGGGCGATGGTTGCGTGGCCGCGGTTGGCACCGGGATAAATCTCTCAGACTCACTCTTGAGCGCCATGACAGGATCGGTCGATTCTGTCACGCATCTATTTATGAAAGAATTGACGGCACTCGTGAAACGCTGGTTACGCTTCTCGATCAGGTTCGCCATCTGCTTCTGACTGTACACCGGCATCTTCAGCTCTTCGTCCCGCAGCTTCCGGGTCGGTCTTGTAGGTTCTCCCGTCTTCTTGCACTGAACCTCCCGCTTGAGGTCTCCGTCCAGAAACTCAACATACAAAACCTCCTTTCCTGTCAGGTCCACGTCGCCATTCTCAATTTGGCCGCGGGTCTGCAGCCCCTCGCGGCCGGTGAAGCCACCGACAGACGAATCTGGTGGACGACTCTGCGAAGTCAGGAAATTCCTCGAAGACCTGAATGTATCATCCCTCTCTGCGCCGCGCGCGTCCAGCTGCAGCATGACCTCGTCGACATATGCGAACCGCATGCCGTCAGGGCGGAGGGTTGCAATGGCCGCTACGTCCTCTATCAGCAGCTGCCGGCTGATGTGCGACTCCACCGTAGACTTTATGGTGTCGAAAGTGGTGGCGAGATGCTTGCGCGTGCAGCAAAATGTGACGACCAGATTGAGGGCCCTGTACGTCTTGTCCAGTATCTTGAACCAGTCGGGCCACTCGATCACGCACTCCTCCTGGACACTTGCAGGATCTGGCTGTTGAGCATTGCTTGCTTTGCTCGTCGATTTGGCTGTCCGCCGAGCCTTGCCGGCTGCTGGTTTCTCAGGTGTGTCAATACCATGGTTGCATTCGCCAGGTGCTAATGCGTCTGCGATTTGAGATCGTT

TACGCTTCCCAGACTTGTTGACTGGTTGTTTGTTAGTTTTCCCAACAGCGTCCATGTCATTCAGTCGATATCGCCAACAGAAAAGTTCAGCGGTTGGAGCGCATGCATGATGCAGATTACATGTGAAAAAAAAAAAAAAAAAAAAAAAAAAAAAAACAGAAAGAAAACTACCTAGGTACGTAAATTCAAGTGTGTTTTGTTAATGCCCGGAAAAGAAAGAAGAAG

CGGCAATTCAATTAGATTCGTTTGGTGGCAACTTGCGTCCTAGTCGGACCCAGTCACGCCAAAATGTCTCGAATTCACGTGACGACATACAAATAAGGGGCTACAACACTTTGTTCAAACCTGGAAACCAACATAAGTCATTGCTGGATGGATTCCTTTAGACAGTAATATGGAGAATTTAAATATTCTGCTAGCTACGCTAACCTACAATAAGCGAGCAGATGGGATTTCGGTCTCAACTTCACAATTATTCTGGACAAGTAGGCCAAACCACCACCAACGTCTATAAGATAGGTAGCAGTGTACGCTGAAGTGAAGTAGGCCGCACCGTAAAAGCCCATATGTACCTAGCTGCCGAGTGACTTCCCTGATACCTGAAAGTTTACACCCCAACGCCTGCTTTCCATCTTCATCCCGCAAGCCAATAATCAAGTTTGAAGAGATGCTCATCGGTCATGTGTCATTACTTAGAATGGCCCAGCCCTTTTGCTGGCTCAGGGCCTGCTGTCGGTTGTCAAAGACAGATATGTGACTGTAAAATTTGCATTTGCGAACTGTGAAACCCTCGGCCTAGCTCGAAGACTTGAGTTCTTTTTGAATAAGGTCGATGGTTTGTTGAAATATCTCGGCGTTCGCCGCAGACATCTAAAGTAGAAAATGTGAGCAAGTGCCTGCTTGGCGTTAACAGAGCTGAATACAGCACATACCTTGACTTGTAATGTGACCGGACGAGCATCTGGTCCCTCCAGGGCCGTGAACAGAAAGCCAACAGCTTTCATCATTTCTCTCCTCACAAACTCCGTCCCGGCTAACACTGCCGTGTTGATGACCACTCTAAGGGTGTGATCCTGACGCATAATCAGGCGTACGACCTTGGGCGTCTTGCTATCGCTGTCACCAGCGTCGAGCATTGAGGCATCGAAAGAAGCCGTGAGAGCAGCACCACTTTGATCGAAGTCTATGGTTGCCTCTGGGACGTTCACTTTGAGAATACCAGCACCGCGTTCCTTCCAAGCCCCTGACTCTTTATCCAGGTGATAAACTTTGGCCCTAGCCAAGAAGATGGTAGCCTCGCCGGCCTCTCCATCGTCCACGACGACTTGTTAAAGTTAGCAAACACGTGAAAACTCGAAGTTCAGCACAAGGTTTTCATCTCACCTTTTTGAAGCTTCGTCGCCTTCTTTTTGTCTTCCACGCTTACAGGCTTGGTCTGGTCTGCTTCTCCGTCTTCGTCCTTGGCCTTCTCCTCGGACTCAGACTCTTCCTGAGCCACGCCGTCTTCGCCAGTCTCTGTATCGCTTTCCCCATTTTCGGATGCTTCATTATCAGTTTCTGGGGCACCAAAGGGCCGAGCTGGCTTATCATCCTTGAGCACTCCACCAGGCTTTGCAAAGCTCGTCAGCTTGGACGCACCCAGGCCACCACCGAACCCACTGCCAAAGGCGCTTCCAAACGGTTTGGAGGCGCCGCCGTTGAGTGAAGCAAATGGAGAGGCCGAGCTCGAGCCATCGCTCGGGGGTCCAAAGGTCAACTTTGGTGGTGCTGGAATGGTACCGGAACTTGCGCCCAAAGCGCCGAATGGCGAGGGCGAGGCTGAAGAAGACAAGGCGCCGAACCCCGATGCGGTGTTGCTGGAGGATCCTAATGAACCAAATGGAGATGCACCACCAGTAGCCCCCAAAGTACCAAAGGGAGATGCGGTACCGCTCAGCTTTGCGAACCCAGACTTTGCAAAGGCGGAGGCCGAGGTAGTAGTCGGCGCAGCATCGTCCTTGCCGGCAGCGGGCTTTTGTAAGCGGGCAGATAATGAGGGGTCCTTGGGCTCGTTGGTATCGGTAGGTTCAGTGGTCTTCTTGCTGGACTCGGTTGTGGAAGCCTGTGTAAGTCGGGTTAGATGCCG

**> MD312_chr03:2489892-2493892**

CGCTTCGTTCGACAAATACCCGCTCGCCCGCTGGTATGTGCTGGCCTAGAGCCCATGTATCACGGACTGGCGAGTACCAGGCTTGGTGTCGACCCCCTTCTGCGTCATAAACGTATTCGCCAATAACTTGCCAATCATCTAGTTTGAACACCTGCTGTTCGAGGATTCTGTTTGCATGAACAAGACCGTTGAGGACGAATCTGTAAACTCAGCGTCAGTCGGCCAGTCACACGTATCCAAGGCGAGCGTGGTCAATATTAGACGTACTGATGTGTGATGCCTTTCGAGTCTGAAGAGCATTCAAAAAGACCATGTTAGTGACGTTCGTAAACAAGAAATTCCCCAGCATTATCCACTGTCCTCCGGTACTTGCCGTTGTATGCGTGACTAAGACAACGTTAGCAGTTGAACTAGAACGAGGGGCCCATCTTGTCTCGGATGAATAGGGTCATCTCACTAAACCTTGGCGGGATCGGAACAGGCATCAACCCCAATTATCATGGAATCTCCGGGACGCAGCACCTCGGAAAAGCCCCGCAGGAAGTCTGAAGCTTCGTCGCGGCGGAAATTGCCTGTAGTAGCAGTGAAGGCGTGAGTATGGATGATCTGCTCCGTGGCATACATAGGCTTCACTTTTCTGACTGACCAATGCTTGAGCCAAGCGACATGACGCATTTCTGTCGACCGAGGCTGGCGGGTTCTTTCAACCATTCGCGGCCATCGTCATATGTTCCCAGGAGACCATGGCAGTGCACATGTTTGTACTGAGGCACTTGGGCCAGTGTACGCTTTAGCTCCTCCTTGGAAAGGTCCAATGCGTAATAATCAACACTTTTGCCGGCATTGTCGATGGCTTGAAGGAGAAGATTGACCTTCCGTAGGTTACTGTGCGAAGCAATTAGTTGGCTGGCGAAGAAAGTTCGTGGAATAAAATTCCAGAGCGAGGCCCCAAGGTCTAATCATACCCACTCCCCAGCTCAACCACCATAGATCCGTCCGGAATCGCGCGAGCTATCTCAGAGGCCGAGGACTCCAAGACTGATATCTCATCATTGGTCAAGTAGTACTCTTCGAGATATGTAATCTGGCAACATTTGTTAGCTCGCTCCCACTCCGAGTCTGTGCGGTGAAGATGGGCATCATCAATATATGCTCTATGCCTGACTTGCTTAGATGCGCGAACATCTACAAGCCTGGCGACTGCATTGATAGCATAACAAGAAAGTGGAGGGGAAAAAACACCAACCTCTTCGAAAATTTGCAACCCACGCTGGTCGTATAGAAGAATGGTGGGCATAGCCCGCTGGCCCGGCGTAGAGAGAAAAGACGACAGGATCTCTTCCTTGAGATCGCTCTCAACTGCAACACCGCGAATATCGATTATGTCAGGTGGCTGGGGAGCGTAGCCGCTGACACTTTTATTTCCGAAGCTGCCGTTGATACCGTTATGTTTTCCATGGCTCAGATCCCAACCGTTGAGAGCCTTCTGCTTAGGTAGAGGATTTTCATTGCCGTAAAATCCTCCTACAAAGCTCTTCAACGGGTGTCCCACACCCCCGGCCATGAGCACTGCAGATTCCGCAGCGGGCATTGCGAAACAGGGTCCTTTCTGGGGTTAAGATGACAAAAAGAGTAGTTTTGTAGATAGATACAGGTGAAAAAAAAAAGAAAAGAGGGAAAAAAAAGAGAAATTGTGCGTAAGAAAATTGCTCAAGGAGGCGATGAGTGGTATGTCTTAAAAGCCGAGAGTTTATTGTTTGGAGATGGATGCTTATAAAATAGGAACTTCAAATTGTAAGA

AACAGGTAAAAAGAAACTTTTTGACAAGGGGGTCGACTTTGTATAAAATGTAAAGAAGATACACAAATAAATGGATGGCCTCATCTACTTCGGATGGAATACAAACGTGGTTTCGTTCAAGTTGTTATGCTTACCAGCAGTAGAAAGAAAAGGAATGGGTACATAAAGTCTAAGCCTTATAAATGCAACAATACTGCTACTAATAATAATAATAATAATAATAATAATAATAAAACTCACCACACGAGAAACTGGGCGTCATTCTTGCGTTGCGGAACCAGCGGAGAGCGCCCCCCGTCA

CAGGAACGAATGCATCCAGAGAAAGCAACCTCTTACAGAAGAAGCACCAACCTTGTCCGCGGCTGTTGGGGCCCCAAGTTACTCGTGGTCACACCTTCCAGCCTGGAGGAACCCAGCCCGGTGAAGGCCCCGGCCATGGAAATACAGTAGCAACTTTTTTTCCCCCATGGATCTGCACTGAATATGAACCTATCAAGCCGCCTGCATGCCGGGGTAGTATTTATACCTTGGTGATGTTTATCAATCAATATGTACGTAGCATCCGTAGGTAAGGTATTTGATTACTTCGTAGTGTCAAAGGGCTAGACGGTGGGTTGCATGCCCGTTCATGTCTGATGTGGTGGCTTTCTCGTGTGGATATTTCACTTGACCCCAACCAAGTAAGTACCACTTGGTAATTACACCTTGGGTTGGTTTGGTTTGATGTGTGGGTTGATGGATTTCTGTGATCAAGAGGCAAGGTACAGTAAAGGGGACCTACATAAGGTGGTGGTATCCAGCAACTTGCAGCAGTGGAAAAGTTGCGGCGCTTCGTTTCATTTGTTGCCTGCCGGGGGGTTTTTGGTGGATATGCTAACCCGATCCATGATGGTTCCGATCTTAGGTATTGTGGTACAAAAACTACCTGGTTAGGTCGCCAAGAAGCATCTAGAAGTACTTGGCTCCAGCCGTCAGGAAGGCAAGGTACGTACATACCTAACAGACCTACGGTACTGATTGATTTACCTTTTTGCTGACAGTACACTAGTTGAATAGGTATATTACATACATACCTACTCGCGTCTGACGTCGACGGAATGGACACGCGAGCGAGAAAGAGCCTGCCTAGCCTGGCCTGGCTGGCATCGGGTGATTCTCTCCAACGAACCCCAAACACTGGCTGCCCTCGAAACAATCGGTCCAGATGAACGCTGCTTAATTGCCAGTCATCGGAATTTTGGCTGGTTGCTTGCGCCTCGCGAGGTCAACTTGTCATGAAAAACAAAAACAAGATGTACTGTGCTCATTGCCAGGGTGTAGTTGTACTACCCAGTATTATGTAGCGAATGTTTAGGTTTCATGGTCGGACGAAAAATCAACGGACGGGGAGCGCGGCCGAGGGAAAGGAAGTTGGAGCAAATCGCATGTTCGGTTTCCCAGCGGGGTGGGTTTTCAGACAGGCCTAGCTTGCCTAAGCTCCACCGAACACCTTGGCAAGGAAACAAAAACCCCTCCGGCCCACTAGGCCAGAGACCAATCGCTGTGATGACAAGGCGCCAAAAAGCTCAGGTAACCCCATTTCCCACAGCTGCTTTTCCATCACAGCCCCTCCTTTCGCAGCTCCTGCCCCCACAGAAAACGAAATAAGAACTCGTACAAGTTTTGACATTGATTTTGATGAAGTCCGCACCACACTGTTTGCCACGCTCGTCCATCCGGTACAGAGCCGTTTTGCCCAACATTCAGCGTTTATTCTCTCGCTCTCTCTCCCGTCTCTCTGCACACCGGCTACGGGCTGACTGCAGCTATAGACGACACTAGCGCTCCGTTCAATGAATGGACTCTTTTATAAACTTTGGAGCCTGACGCATGCACTGGCTGGCTGGCGAAATTATAGTGTGCCGAGACTCCGCGCGGCGTCTTTCCCATTGTCGAAGGTGGTGCGGAGGTCATCAGCTGTACCCGTAGCAGCGTCATCGACCCCGTCATCTACACCAAAGAACGGGGCACATTACGGGGTAGCTGTTCCGAGGGAGCATCGTACCATGTCTTGGCAGTCACTCGAAGATCCCAGCCAAGCTCCGACCTTTTGGACTTCACCTTAGAGGTCAAGCAGGTCAAGTAATTATTGTAGGAACAAAAAAGAAACAACCTACTCCAAGCCACTCATCACATCCTTGGCCTTGCCGACCCTGTCTCG

**> MD313_chr03:2656725-2660725**

ATATCCGCGCGCTAGCTATGCCGAGTTTATGACATTTACTTCCAAGACCGTCGCCGAGCGGACCAAGGCGAGTGTGACTATACCGAGACCAGAGCCTCGAACGGATGCTGACAGCCTTGTTTCTACAGCCGGGACAACGCCAGGATGTAGAGGAGAATGGTAATGGCCTAAATTGCTTGCCGCCCTGGCATGTAGAAACACTATGACCTCGCAATGGACAGCTGACGTCAGAGATAAGAGTATACCTTTCACGCATACGGACGGACCGAAGGGATCTGCGGCATCATCATCTCAGACCACGCCTACCCGGCCCTCGTTGCGCATCAGCTACTTTTCAAAGTGGTCGACGAGTTCCTGGCCAAGCACCCGCGCTCCGCCTGGGCGACCGGCAGCCCGAGTCTACCCTTCCCCGAGCTCAAGGAGTACATCACTACGTATCAAGACCCGCACGCTGCCGACAGCATTCTCAAGATCCAAAAGGAGCTCGACGAGACCAAGATCGTGTTGCACAAGACCATCGAGTCGGTGCTACAGCGTGGAGAGAAGATTGACGACCTTGTTGCCAAGAGTGACGGGTTGAGTGCTCAGAGCAAAATGTTTTACAGTTAGTTGCGCCCAATCTTATGTGACTATGGCATAGCGGCTGACATATTTTCCCATCACCTTTTAGCGCAAGCAAAGAAGCAGAATTCGTGCTGCATTGTCATGTGAACGGTTATGGGTACCAGCAATTGTTTAGCTTAATGAGAGACGTGCGTCGGCACGTAGTCGGACATGTCCTTGCATTCTTTTCGCAGCGTTTTCGTCTCAAGGTGGCTCAATCGTGTGTTTTTTTTTTGTTTTTTTTTCTGCCCTGGATCTACGTTGTAGGACAATCTATCCCTTAATCGTTGCGTGCAAAACCCTACCGTAGAAGCTGTGCACAAATATCATCAGCGATTGGTCTGATGCATCGACGAATATCCTTTGTCTGTATGCTAGTCAGCAATGACATTGCACAATAGACTGCAGGACCACAAGCATCTCGTGAGTATCCTCGGATGATAAAAAAAAACGCTATTTGTCTGCAGGTGCTCTGGCCCGAAATTGACCTTGCATTGCATTCAAAGAAAAACCACCCCAACGCCAACGGGCAGATTACAGGATTCCAGGCACACTGCGACTACCACCGTACCGCCTTTGATGGGCGAGAAGATTGGCAAGGTTGAAAGTCAGGTAAAAATAGAGGGACTGGGCTTTTCTGGGGCGGGTTGAACTCGGCCACATTTCATCTCGAAGGTACCGGGCCATCGTTGCCAGGGTCCATCAGCGTCCTCCTTGGCATCCCGTCGTTGCAGAAGTGAATGATGAGCCAAGAGTGCTCTGCAAATCCCCCGCCATTCTTTGGCACCCACTTTGAGATTGTATCCGAATATAGAAAGACAGGGTTGGCGAGTGTCCTCAATATAGCAGCCCCCAAAGTTGTGCCTAACCTTGCTCGCCCAAGGCGAACCAAATCATTTTTTGTACTGGGCTTCCCTTGTCTCGTTGCCACCTAACTCATCCGAACACGCGATGTCAAGGGATATAGTAAGTCAACTACATCTTATGGTTGTTGTAGATCTTGAGATGTTGTCGTCTGAGTGTGAGTAGTGATCTCTTGGTGCAGTCATCCCACCTAGGCCACAATCTAGTTCTAGTTTGAATCCTGTTTATATACGTCAGACCCAACTTGGACAACAGCTCGCCAGACGCAGCGGGAACATGTAAACCCGCTTCAATCAAACCAGGTACGTACGAAGTAGGTATGTTTGGCTATTGCCAGCCCGTCCAGCTGGACTGTTTATTAAAATATGTACTATGCGGCCAGCCCTTCCACCCAAGACGTGGCTAAGGGAGCATTATTTAGTATTTACGAACAAGCGATGAGAAGAAACAGAGATAGGTCGTTCCAAAAACAAAACAAAAAAG

AGGTCGCTTCCCTGAGCGACTTTATAACGCAAAAAAAAGAAAAAAAAGAACCCCCCCCCCCCCCCCAAAAAAAAAAAATACCAATGCATCCAACGCGTGTTCATTGCCAGCATGTAAACAGCCCGGGCAAATTGAACCAGAATATCCAAC

CAGAACCTTGAGGCCAGCCGTGGTCAAAGGAATGTTTTTGTGCCCATTAATCCCAAGCGCCCCTTATAAGCCCATCAAACAAAAATCAACGCCAAAAAAAAATCGCTCCCGTTCCAAAGAAGAAAAAAGAAAAAGAAACAAAAAAGAAATCTGTATCGTTCAACCCGAGTCGGGACCAGATATCCAAAGGCGGGGGTATAGTAGAAGAGGTGCCAAAGGAGTGAAAGAAGTAAGAAGATTATCCGCCCTACGGGGCAGGATTACAAGGTCTAGGCAGCCTCCAAAATGACCTGCTCACTCTTCCGGTGCATCATCAACTGCTCGGCCGATAGATGCTGCCTGGGTGGATGAATGAGGGTCGGTGTGGACGACTTGGTCTCCTGCAGACGTTCATTCTTACCCAGCTTGACTGGGCTCACTTTTGCGGGCGAAGGCACTGGCCTGCCAGCCCTGGCGCCGGTAGCCGTCATCCCATTCCGGTCCCGCACGACGCTGGTCTGCAGAGGCCTCAGCATTTGCCGCTGCTGGCGCGAGATACTGATTTGTCTCGCGATTGAGATCTCCACCGCAGCCTTGAGCGCATTGTCGGACTCGTCATCGACAGAAGGCCTCGTAACGTTGGTGGCTACGGAGGATGTGGATGATGGAGATCGCTTGCGGGTGCTGCTTGCGGTACTCGAAGAGGTGGCGGCCGAGCTTGGCGGAGTAACCAACTGCCAGTGCGGCTCTGCTATCGTCGGCTTCAGTTGCTGCACGGTTGTTCGCTGGTCACGCTCATAGTCGGAGACCGGACTGGTGGGGGATTCCAAGTAAAGGCTGGACTGGCCCTGTCCAAAGTGCACGGCCTTGTCCCTAGCGCTCGGGTGCAGGTACCTGGACGATCCTGCCGAATGGCTGGTTTTGTGTGATGCCTGGCCTCTGCTGTGTGCCGCATTCTCAGCATCAGAGCCAGAGCCAAAACGCGGTCTTGCGGGTGAAGATACCGCCGCAGGTGACGTGTTCGAGCGGTTCCTAGGCGACGACGGGGATCGAGAAGATTCGAATCGAGAGTCAGGAGTAGGTGGGAAGAGAGTAAAGGCGGGCGACTTTGTCGGAATCGGCTGGGGGGATGTGGCGGCGCGTTGTGGTGCTGGTCTCATCCTCTCTTCCTCCTCCTTGATGATACAGTCGTTTATGCTCTTGAGTCGGTCAAGAGTTGCCTGTCGCCTTGCGAGGAGAGATGACGAGCTCTGAGGATTCAACAGACCACTAAACATTACGCTATATCTCTCCATCTTGACATCCGGAATCTCCACGTTCAAAAACGGGCCGCCTGTGTTAGTAATGGGGATAGGTGGTGGCGGGTTCACAGGCCTCGAAATTTCTGGCCTTGAAATCTCTGGCCGCAGAACCTCTTGCCTCGGAACCTGTGCCATAGTATGGGGCTGGTAAGGATGCTCTGTATCGATCGAGTTGGCGATTGGGATGGGATTGCTGATTTTTCGTCGTGGGCTCCTGGGGGTGGAATTCATGGGCTCGGTATTGGTCCTGGCGATGAGGGGCTTGTATTTTGAGGCGTTGGAACCAGCCGATGTGTTACTCCTCATTGGAGTGGACGGTGACCGCTGACTCTGCAAAGAAGAGGCAGACCTAATGTCCGAATAGGAGGAATTTTGCGAGGGCAGAGGGATCGTAGGCTCCTCTACATGCTTTCTTCCGAATAGCCCGAATAGTCTTCGCCTTCCTGTCTTGGTTCGCTTGACCGGCGCCGGTTCCGGCTCCGGCTCAGGTTGCGGTGGTGGCAGCCTCGGCTCCGGCTCTGGTCGGACAACCTCCTGCACCTGAGGTACCTGTGGCCGCCAGCTGTTGACAAAGTTGGGCGGCGAGTGTCCCTGCGTCGGGCTTCCTATAGCCATGCCTA

**> MD315_chr03:5493540-5497540**

TAGAGCACAATGAAAAAGAAAAGAAAAGATAAAATGCAAAAATAATAGACAAGAAGGGATGAAAAAAAAACAAGGAAAAAAAGACCCTAATACCTTCTCTGGAGGCTTTTGGTCGTTTTTGTCATAAGTCATTTCCCAAGATTTTTGACCAAGGGTGTTTTTCGGCCTCTGAAGTTTTATCCATTCTCCCTCCCGAATAGCAAAGACAACTTGTTTAGGATCTCTCAGATCTCAGTCCGAACAAGCTAAGTGAGCTTCACGAATACCTCCCCTTGGTGTATCTCCACAACAGACTAGCCCCCTGAAGTGCTGGAATCACTTTTGGCCTCTTCCCATCGACTAGATCAAGTTCGAGTGCCAGACCCACAGCTCGGGCGAACCGTTTCCTGAACCGCCTGTAGTCGTCGTTCGTAGGCCAAGGGTCGTCTAGGCACCAGGCGGAGGCATCCTCGCCTACGGCCATTGCAGCGTTCAGATCCCAGGTGTCGTCCTTATCCTCATCCACGTCAAGCGGATAAAATAATACTTCGTTGAGGTGCGGGCGGTGCGGGTGTTGATTTATGTTGGCCTCGATGTCGAATTTGAATCGGTATGCCTGGTGTATAACCTGTGGTTGTGATGGCGCCATCATATTTGCAGATGGCATATTATATAGAGGCGCTGTTGGTGTAGCAGCAGAATGAAGTGTTTGGGGTGGTTGCGACATCTGTGCCTGCATCGTTGGTCCCGTGGCCGCTTGTTGGTAGTGATATTGTTGCATCTGCAAATGTGGGTTATGTGGGTTATAGCCGTTGAAGTTAGAATGTCGCGCAAAGTCGAACTCGAACTGGAACTCTTGATGCACCACGAATACTAGTGCCTTGAGGCTGGGAAAGTCCGCCAGCCGAGCCACAAGCTTTCCCACGTTGCTCTTGGAGACCTTCCAGTTGAGGACCGGATGACAGATGCCAGTGTAGACACCGTACGAGGTGCCCATGGCCAGCCTGCTGAGGTTGTCGCGAACCAAGGGAATCTGGCTCATGAACTGCAGTGTTCGGTGTAACCTCTTGACTAATAAGTATGGCTCCAGCAGGAGCATATCCCGCGACAGGTCGACGAACTTGGTTGTCTTGCCTAACGTGAGAGGCTTGTACCGATGTAGTGCGATGTACCGGGATTCGCGGCAAGCAGCGGCCAGTGCCGGGGGCGGCTCCGGATGGACATTGGCGAACATCAAGCCCTCTTGAGCCGGCGTCTTCGCTTTGGCGTGTGGCGCATCCAAAACCTCGTAGACTCGTCCTTCTGGGAGAGAGTACTCCCAGACCATGGTTCGAAGTTCTGGCGGAAGCTTGGGAAAGTTCACAAATTGTGAAGGCATTCCCGCGTCTCTGCTGGCCGCAGTCGGCTCGTGCCGCAGAGGATCGTAGTGTAAACTCATTGGTGCTGTTTATATCCTAGGATGAGCCGAATAAGGAGACCGGGCTGAGTAGGTCGGCTTCTTGTACCTTGGGTGTGCAGTCGATTTCTCAAATAGCCGGGCCCAGCCTCTCGGATTGGTGACCAGAGGACCAAATTATCAAGTAAAAAATAAAAAATAAAAATAAAAAAAGGCCCTCTACGGGAGACAACACCACACCGCCCGGACAAGAAGAAGCAGACGGCGCATTCACTCGTTATAATGCGCTCAAATATAGTGCAGCAAGCGGTAAGTATGCGCGGGGTACCCGGTTCCCGTCCTGAGTACTGTCGAATGAGGTGTCTGCCACACACACTATCCAATATGGTTGACAAGTTTCCGAAACCACTGTGGAATTCAAGAGGTGGCTGCGTTGAGAAAAGGGGAGGACGAGCAGTTGGAAATTTCTAATTTCTAACTGGTGCCGAAAACATAAAAGA

TTATTATTTCTTTTGGTAGGATTATATGAGATGAGCCCAAGTCAAACGATGGAGAGACCAGAAAAGGTGTCATGTGTCGCCAATAAATTGGTGGCCAAAAAGTACAGGTGGTAAGGTACTCTGTTTATATATATATATATATATATATAT

ATATAAAGTGGAGGGGCTGAGTCAAGAGAACGAAGAAAAGCCAAGGTTGAAATAATATAGATAAAAATAAGGAACAAGAAAATAAGTGAAGAGGAACAAAAAGAAATCAGACGGAGAAGTTGGGGCGAAGAAAGGATAAGGGGGAAGGGTGTTAATTAATGAGGATTCCCTCGTCAGGAAGCGAAGTAGCTAAACATGACTTGATGCGTTAAGCTTTCGTTGGAGCTTGAGCTTTTGCTTTACGAAAGTGCTCGGGTCAGTCATTTGACGTCTCACCCTGGGCAAGGCCCAGGAGCGACGAACCGGAGCTTAACATCAGGGCGCCAGCCGGGAATGGAGGGATGCGCGTGTTCGGAAACATTGGGCTACCAAACATAGTGGTCGTAGTAGTCGCAGACGTAGTACAGAACGGAGCTTGTTTGACTGGTGCGAGGAGGGGCGCGGTAAGGGCAGAAATGTAGCGGATGGATGAATAATGGATGGATGGATGGGCCGAAAAGAAAACCTGTGATGCACTATTGACAAGGCCAGAGGGTAAAGGGTTAAAAAAAAAGACATGCAGGGAGAGCATTAGAGAGAGAGAGAGAGAGAGAGAGAGAGAGGGATAGAGTGACAGGAGAGAAAAAAAAAAGAAAAGACAGCCACACACACACACACGTATACAGAAAGTACAAAAAAAAAGAAAGAAAAAGAAATAAAAGCAAAAGCCTATGGTGTGGATGATGCGCAAGCCCAGCGTCGTTTCGAACAAGAAATCAACACACAGATGGAGAACCTTTTACAAGCGGAAACATCACATCAGACGCGGCATCGACTACCACTGCACAGCATGGGCATATGCGAGGTCGATGTATGTATGCGTGAGTGACTGTGGGTGTGTGTGTGGTCAGAGCGGCCGGATGGGGGACAGACAGACTGGAATGGAATGAGGGGCATTGCAACATCTGGCTGTTGTTTGCTGTGTGCGGTTGCTTCACCTAGACTCGGCGCTCCGTCTTGCCCGCCCCGGAACCAACGTGTCGAAAAGGACGCGGCGCCCGCTTGGGCAAGGAAGCACAGCACTGGGGCTACTGCGTATTAACTGCCTGAATGAGGCAACTCGATGGACGCAGCTTGGCAGCCCGGCGAGGCGCCGTTGATAAACTGCCTGCGGGCTAGCAGGTGGCCATTGCGCCCCAAGTCTTGGTGGAGGGGGGCCAGCTGAGTCGAAGCTGCCCTTGACGTCGCCGCAAGGAAAGGCCAGCAGGACAGGGCAGGCGGGCAAGAAAAAAGAGAGAGAGAAAAAAAAGGTCGAGATAAATCTAGTGTGGGGGGAGGTGTTTAACCACTTTGAGTAAAGCGATTCCTACAGATCAAACGAACCCGGAACCGACGACATGTCTGCTGCCGGACCGCTGCTATAATTGGGGTATAGCAACCCACATAGTATCGCCTTTGCAGTGATTTCCGACTTGCCGTGTTGCTCGCTTGCCCGGATAGCCAGAGGATAAGCAAGAAAAACAAGTCATACAAAACACATAGCAACAAGAGCAAAAGCCTGCCGAAAAATGGTACGATCGCTTACCAATCTTGGCGACCCACGTTTGCTCACATTGCAACGTTCTGCTGAAACAGATTTGCAGTTTGAAGGACTGCATTGCCCTCGCGTGAGTGACGGCTGCTCTCGGCGCCACAATCGAGCAACCTTATAGTTAGTGAGGGGGCAATTTTCAGTGGTTGCATATTGGGTGCGCTATCGACGATGGGCCGACAAAAAAAAATCACAACCGAAGCTACTCATTAATGGCCGGCGGGTTATCACCAGGCATGCAAAACGCCCCCATATGGCTTTTGTTCCATCAACACCTTCCCGCAAGCGACATCGGTATAACAAAAAATGAAAAACAAAAAAGGAGCTTGCGGAGAAGGGCGGACGAGCAAGTTTTTACAGACGGCAGAGATGCAAGCAGTTAGTTGGTGATGGTGGGCATGCTTG

**> MD316_chr03:5969371-5973371**

CGTCGGCGAATGACTGTCATTATGTGGAAAAGGGAAAGGGGGGGAATTAAAAAAAAAATGCAAGAACCCAGCACCTGCTTTAGCGCAACACAGAGCACGGGGTGGCGGACACGACACGATTGCCGCAATCTAAGGCGCGGGAAGCATAATGGCTGAGGGGCCTGGACCTAGCTAGTGCCGGGGAAAGGAGTGCCTGGTCTCAGGGGAAGCTCAAATTGCTCGCTGCGACAGGGCGCTCTTTTTACAGAATTACTGGACTACGCCGTTTCAGCTCTGCCTCAGTGTACAGACGGACAATGATTGGTCGGGTTCGGGCTTTGCGGGGCTCCCAGGGTTCCAACAGGACCGCTAGCGGAAACTTACGTAAAATAGGGGTCTGGGAAATTGGACAAAAAAAAGGTGTGCCGACTTGGCTTTGGCGGACTTTCCGTTTCTTTTTGCTCCCATGCTCAATGCCGTGCAGCGCATGGAGAAAACAGCCGACATCGGCACAAACCCGGAGGCATAAGATATTGGTCCAACGAAAGGCTTACGGACGCCAGACGCCCGCCCTCCGGCCGAACTGATCCAACAAGCCGGCCTCTCCGTGATGAATATGCCGCCTTGGTGCACATTGTTTGTCGCTTAATCGAACAGCATCGCTCTTAGACTTTAAACAGATGCCTCGCATCTTCAACCAAGCAGCATGGTGCGGTAATCTGACGTCATAGTTAATAACAAAATACTAATAACCGTCGCAGCAACACATGCCGTGTCCGCAAGATCAAATGTTTGTGAGCGTTTGTTGTTGTAGTTCTCCTTTAGCTGTCCAGAGATTGTCATCTCACATTTTTGTTCCCAGGTGATGAATCGCTCCCCATCTGCCAGCGCTGCTCCAAAGCAAGCCGCTATTGTGACCGCAGTGCGGCGCCTCCCCTCCCAGTCCTTGTGCAGCCAACAAACACACAAGACGTCTTTCCATCGCCGTCGGATCCCCGCCGGGCTCTCACCAACCCCGCGATAGCCCGCTACTTCCACCACTACATCACCTCCATCGCCCCCTGGTACGACCTCAGCGATGGGTCCCAGCACTTTGCCACCCGCCTGCCAGAACTGGCCCTCGCCAGCGCGCTCCCGTTCGCTGCTGTCGTCGCCTTGGCCGCCATCCAGATCAGCCAGACGACGGCGCCGTCGGCCCGGGCGGCGGCCGAGTACTACCATGACCACTGCGTCCGGCTGTTGTTGGCTAGGCTAAAGGACGACAATGCGGCCCGTGGCGACGGCGACGGCGACGGCGATGGCAATGGCAATGGCAATAGCAATAGCAATGGAATAACGTTGGCTTCCATCTGTCTCCTGCGCTCATACGAGATCCTAAGCGAGGAGGTCGATCCAAACCGGCATCTCCGGGGCGCGTACTCGCTTGCCGTCTCCCGCCGCCCGCTCCTCCGGGAGGGTGACCTCGGCGGACGTGGAGGGCTGCGCGGTGCCGGGTTCTGGAACTATCTGAGGGAAGACATCACCTTTAGTCTGTTCCAGCGATGCCCACTCAAAATGGGACTGGATCAGGTCGAGCTGTTTGTCTGGCGTGGCGACGATGCAGACGGCGACCACGCCTACTTGAATGCGGTGTCGCTGATCCTGGGAAGGATCATCAATGAATGCTTTGGGCGCGAGACGCTGTCACCTGAAGCCTGGGACTTTTTGTTCAGGTTGCTTCGCGATTGGTGCTCGGCTCTTCTCGGC

CGGTTTGCGCCGTTTGCACGTGAAGAGCGCCGTCTGGGGCTAGCTCTGCCTTCTGTTTGGATGTTGCACGATTGCCATGGTAGGAACATTTTCCCTTGTTTTTCTTTCCCCAAGCATCGCAGCTGGACAAAGCTCGACGGATACCAGCAGTGACAGAGTGTACATGCTGATGATTCCGTGATGCCGCCAGCGTCGGCACGCCACTACCGGCTTGTGTGCATTGCCATCCTCTGCAACCACGCATCCACCTCGGAACAAATTTCGGACCTTATTTCGCTCCTAGAGGACCTTATTTCGCTC

CTAGAGGCTTCTCAAGACGTTGATCAGTGCACCACAAGGGAAGAAATTTTAGAAAGATGTGCAGTTGAAATCTGCGGGATCGCATTCACTGCCAACTCGCCTCCTGTAGTGGTGAATTGTTTTGGTCCTATTGCATTTTGTCAGTCTTGCCCTGTTTGTCTGTCCTTGTGCTTGTCCTTGTGCATTTCGATCACAAGCATTCCTAACGCGCCAAATCGTATAGGTGGGCCATTTATCCGCGACGAAGCTGCCCAGCAGGAGCTTGTCCGTCGGCTGATGGCCTGCAAACGAACCATCGGATGGCCTGTCCAGCGGCTCGTGGCGGACTTGCAGCTTGCATGGGGAGAGGCTGCTGGACAACAGATTGCTGTGTGATGCAGCTAGACTTGGTCGGGCCCGGTGGTGCAGTTGCAGTTGAGTGCTGCGAGTCAAGGATTTCCGAGCTCGGGTTTGGCTTTGCCTAATGCGGTATCCCCGCTCCCGCCTCCTTCACGTTCAAAGCCGAGCTCGGGTTTGGAGATGAGGTCACCACGCGATGCCTCATACTCGCAAAAAAAACTATTTAGATTGCCTCGCAACGACACTTGAGATTGTGGTCCTCGTTTGACCGGCGGACACCTCGTCAAGATTTTCTACAACCAAAAAACACCTGTCATGGCATTCGCACACCAACTTGGAGCTGCGCGACTTCTGCTCCGCCAACACCGCTTCGGCCTCGCCCAATCCAGCTGCCGCCGCACCATGGCGACACTTTCTCAGAAACTCAGCAACGAGCCCGCCGTGATCAAGCCATCGGCGGCAGAGATCAAGAACAAGACCCTCTCATCACGCAACCTCGAAATGGCCGTCCGTCACCTGCACGCCGACGGCCTCGTCGTGGTCGAGGACGCCGTGCCGCATGCAGACCTCGATGCTCTGAACGCAAAGATGGTCCCCGACGCCCGGTATCTGCAGTCGCTCGGCGAAGACGGGCCCTTCAACTACAACCAAGGCAATCTGCAACAGGACCCGCCGCCGGTGGCCGAGTATTTTTTTCCCAGCATCTTTACCAGTACGTTGTGACACTGTTGCCATGGCATCACCACTCCAGACAGGACAGCATTTCAAAGGTAGAGTTGAAATTACTAGCACGAACAACAGACAAGATCGCGACGCAGATAACCTCGTCCATCCTCGGCCCCCGCCCGAAATGGACCTTTTGCTCCGCCAACTCCGCCATGCCGCCTTCGTCCCCCGACTCTCCCCCGCAGCGCCAGCCCGTGCACTCGGACGCCGACTTTGACCACCCGCACCACCCCTTTGCGCTGGTCGTCAACGTGCCGCTCGTCACCATGACGCCGCACAACGGCTCGACGGAGCTGTGGCTCGGCACGCACCACCTCTCGGACCTGTCGGCGCAGGAGGGCGCGCACGGCGAGCGGGCCAGCGGCCGCATCAGGCAGCGCCTCCTGGACGCACGCCGGAGCACAAGAGGCCCCTGCCAGCCCGTCGTCAAGAAGGGCAGCGTCGTGGTCAGGGACCTGAGGCTCTGGCACGCGGGCATGCCGAACCTGAGCCGGGACGAGGTCCGGGTCATGCTGGCGTTTATACACTTTGCGCCGTGGTATCGCAACCCCATGAGGTTGAGGCTCGGGGAGGACGTCAAGGCCAGGATCGAGGACGTCGCGGCCGAGCTGGATGTTCCGGTGGACTGGGTGGGGAGGGAGGAGGTGCTGGGTGAGTATCTGCGGCGCGGGTTTGGGAATAGCTATGATTTTGGGCAGGAGGCGTGATCTGATGGGTTGAACTTTGTGGTGGCGTGAGGGCGCAAATTTGGTGACCTTTTTGGTCGGCAGAATTCCAGGCTTGATGTTTTTCTTTTGCCGATTGTGTATCCAAAATCTCGCTTTCTTCCCATGGCGCCGCTGACCCTGTACGTTCAATAGTTGGCCAACGCCAATCTTTGCCTCCTCTTGACTCTCCGCATGCGCCTGCG

**> MD317_chr03:5969378-5973378**

GAATGACTGTCATTATGTGGAAAAGGGAAAGGGGGGGAATTAAAAAAAAAATGCAAGAACCCAGCACCTGCTTTAGCGCAACACAGAGCACGGGGTGGCGGACACGACACGATTGCCGCAATCTAAGGCGCGGGAAGCATAATGGCTGAGGGGCCTGGACCTAGCTAGTGCCGGGGAAAGGAGTGCCTGGTCTCAGGGGAAGCTCAAATTGCTCGCTGCGACAGGGCGCTCTTTTTACAGAATTACTGGACTACGCCGTTTCAGCTCTGCCTCAGTGTACAGACGGACAATGATTGGTCGGGTTCGGGCTTTGCGGGGCTCCCAGGGTTCCAACAGGACCGCTAGCGGAAACTTACGTAAAATAGGGGTCTGGGAAATTGGACAAAAAAAAGGTGTGCCGACTTGGCTTTGGCGGACTTTCCGTTTCTTTTTGCTCCCATGCTCAATGCCGTGCAGCGCATGGAGAAAACAGCCGACATCGGCACAAACCCGGAGGCATAAGATATTGGTCCAACGAAAGGCTTACGGACGCCAGACGCCCGCCCTCCGGCCGAACTGATCCAACAAGCCGGCCTCTCCGTGATGAATATGCCGCCTTGGTGCACATTGTTTGTCGCTTAATCGAACAGCATCGCTCTTAGACTTTAAACAGATGCCTCGCATCTTCAACCAAGCAGCATGGTGCGGTAATCTGACGTCATAGTTAATAACAAAATACTAATAACCGTCGCAGCAACACATGCCGTGTCCGCAAGATCAAATGTTTGTGAGCGTTTGTTGTTGTAGTTCTCCTTTAGCTGTCCAGAGATTGTCATCTCACATTTTTGTTCCCAGGTGATGAATCGCTCCCCATCTGCCAGCGCTGCTCCAAAGCAAGCCGCTATTGTGACCGCAGTGCGGCGCCTCCCCTCCCAGTCCTTGTGCAGCCAACAAACACACAAGACGTCTTTCCATCGCCGTCGGATCCCCGCCGGGCTCTCACCAACCCCGCGATAGCCCGCTACTTCCACCACTACATCACCTCCATCGCCCCCTGGTACGACCTCAGCGATGGGTCCCAGCACTTTGCCACCCGCCTGCCAGAACTGGCCCTCGCCAGCGCGCTCCCGTTCGCTGCTGTCGTCGCCTTGGCCGCCATCCAGATCAGCCAGACGACGGCGCCGTCGGCCCGGGCGGCGGCCGAGTACTACCATGACCACTGCGTCCGGCTGTTGTTGGCTAGGCTAAAGGACGACAATGCGGCCCGTGGCGACGGCGACGGCGACGGCGATGGCAATGGCAATGGCAATAGCAATAGCAATGGAATAACGTTGGCTTCCATCTGTCTCCTGCGCTCATACGAGATCCTAAGCGAGGAGGTCGATCCAAACCGGCATCTCCGGGGCGCGTACTCGCTTGCCGTCTCCCGCCGCCCGCTCCTCCGGGAGGGTGACCTCGGCGGACGTGGAGGGCTGCGCGGTGCCGGGTTCTGGAACTATCTGAGGGAAGACATCACCTTTAGTCTGTTCCAGCGATGCCCACTCAAAATGGGACTGGATCAGGTCGAGCTGTTTGTCTGGCGTGGCGACGATGCAGACGGCGACCACGCCTACTTGAATGCGGTGTCGCTGATCCTGGGAAGGATCATCAATGAATGCTTTGGGCGCGAGACGCTGTCACCTGAAGCCTGGGACTTTTTGTTCAGGTTGCTTCGCGATTGGTGCTCGGCTCTTCTCGGCCGGTTTG

CGCCGTTTGCACGTGAAGAGCGCCGTCTGGGGCTAGCTCTGCCTTCTGTTTGGATGTTGCACGATTGCCATGGTAGGAACATTTTCCCTTGTTTTTCTTTCCCCAAGCATCGCAGCTGGACAAAGCTCGACGGATACCAGCAGTGACAGAGTGTACATGCTGATGATTCCGTGATGCCGCCAGCGTCGGCACGCCACTACCGGCTTGTGTGCATTGCCATCCTCTGCAACCACGCATCCACCTCGGAACAAATTTCGGACCTTATTTCGCTCCTAGAGGACCTTATTTCGCTCCTAGAGG

CTTCTCAAGACGTTGATCAGTGCACCACAAGGGAAGAAATTTTAGAAAGATGTGCAGTTGAAATCTGCGGGATCGCATTCACTGCCAACTCGCCTCCTGTAGTGGTGAATTGTTTTGGTCCTATTGCATTTTGTCAGTCTTGCCCTGTTTGTCTGTCCTTGTGCTTGTCCTTGTGCATTTCGATCACAAGCATTCCTAACGCGCCAAATCGTATAGGTGGGCCATTTATCCGCGACGAAGCTGCCCAGCAGGAGCTTGTCCGTCGGCTGATGGCCTGCAAACGAACCATCGGATGGCCTGTCCAGCGGCTCGTGGCGGACTTGCAGCTTGCATGGGGAGAGGCTGCTGGACAACAGATTGCTGTGTGATGCAGCTAGACTTGGTCGGGCCCGGTGGTGCAGTTGCAGTTGAGTGCTGCGAGTCAAGGATTTCCGAGCTCGGGTTTGGCTTTGCCTAATGCGGTATCCCCGCTCCCGCCTCCTTCACGTTCAAAGCCGAGCTCGGGTTTGGAGATGAGGTCACCACGCGATGCCTCATACTCGCAAAAAAAACTATTTAGATTGCCTCGCAACGACACTTGAGATTGTGGTCCTCGTTTGACCGGCGGACACCTCGTCAAGATTTTCTACAACCAAAAAACACCTGTCATGGCATTCGCACACCAACTTGGAGCTGCGCGACTTCTGCTCCGCCAACACCGCTTCGGCCTCGCCCAATCCAGCTGCCGCCGCACCATGGCGACACTTTCTCAGAAACTCAGCAACGAGCCCGCCGTGATCAAGCCATCGGCGGCAGAGATCAAGAACAAGACCCTCTCATCACGCAACCTCGAAATGGCCGTCCGTCACCTGCACGCCGACGGCCTCGTCGTGGTCGAGGACGCCGTGCCGCATGCAGACCTCGATGCTCTGAACGCAAAGATGGTCCCCGACGCCCGGTATCTGCAGTCGCTCGGCGAAGACGGGCCCTTCAACTACAACCAAGGCAATCTGCAACAGGACCCGCCGCCGGTGGCCGAGTATTTTTTTCCCAGCATCTTTACCAGTACGTTGTGACACTGTTGCCATGGCATCACCACTCCAGACAGGACAGCATTTCAAAGGTAGAGTTGAAATTACTAGCACGAACAACAGACAAGATCGCGACGCAGATAACCTCGTCCATCCTCGGCCCCCGCCCGAAATGGACCTTTTGCTCCGCCAACTCCGCCATGCCGCCTTCGTCCCCCGACTCTCCCCCGCAGCGCCAGCCCGTGCACTCGGACGCCGACTTTGACCACCCGCACCACCCCTTTGCGCTGGTCGTCAACGTGCCGCTCGTCACCATGACGCCGCACAACGGCTCGACGGAGCTGTGGCTCGGCACGCACCACCTCTCGGACCTGTCGGCGCAGGAGGGCGCGCACGGCGAGCGGGCCAGCGGCCGCATCAGGCAGCGCCTCCTGGACGCACGCCGGAGCACAAGAGGCCCCTGCCAGCCCGTCGTCAAGAAGGGCAGCGTCGTGGTCAGGGACCTGAGGCTCTGGCACGCGGGCATGCCGAACCTGAGCCGGGACGAGGTCCGGGTCATGCTGGCGTTTATACACTTTGCGCCGTGGTATCGCAACCCCATGAGGTTGAGGCTCGGGGAGGACGTCAAGGCCAGGATCGAGGACGTCGCGGCCGAGCTGGATGTTCCGGTGGACTGGGTGGGGAGGGAGGAGGTGCTGGGTGAGTATCTGCGGCGCGGGTTTGGGAATAGCTATGATTTTGGGCAGGAGGCGTGATCTGATGGGTTGAACTTTGTGGTGGCGTGAGGGCGCAAATTTGGTGACCTTTTTGGTCGGCAGAATTCCAGGCTTGATGTTTTTCTTTTGCCGATTGTGTATCCAAAATCTCGCTTTCTTCCCATGGCGCCGCTGACCCTGTACGTTCAATAGTTGGCCAACGCCAATCTTTGCCTCCTCTTGACTCTCCGCATGCGCCTGCGCCGTCTT

**> MD318_chr03:6070583-6074583**

CCGCTGTGGCCCGACACGGCTTGGCCGATTGAGCCCGAGCCTGGTCCACCCTGGAGCCAAAGAGTCAGTGGGGCCTTTCGTGGGGACTTTCTGGCCTCGAAGAACATGAAGAACATGTTGCTCTCGACGGGAGAATCCTCATTTGGGGGTATGGTGATGTAGCCGCTAAAAGATCGCACCCCAGGAGTGGTCTCGCAGATGCGAACCTGTTTGCTTAGTGGTTAATGACTTTTCTTTTCGTCTCATCATCGCGGCAATCAGATCATGTACGGAGTGGTACCCTTTGGAATCTTCTAAAGTGTCCATACTGGGCTGGAACGGATCTGTAGGCATCGTCGTTGAACAGGACAAAAGAAAAGGACAAAACGATGGCTAATCCTATGGTCATCGCGAGGACCTTGTTGCCTCGCGCTGGCATTGTGCAACACGGCAGAATCAGAATTGAAGTAACGGCAATCAATGACAATCAATGACAATCAATGCCACTGTTCAGAGACCAAAACCCCAAGTAGAGTCGCCCGTCCAGACAGTCAAAATAGAAGGGCGAGAACAAAAGTCAATCGCAGCATTCCAGCCAAGCTTTTCGGGCTTCGGCTAGCTCAGTTTGCTACACGTATGCCAGAGACAAAAGTCCCATATGGATGATGATCGTGTCAGCGCAATCAGGCCATAGACCAAAGTCCAGAACTTCAGATGGTAGAAAACACCATTACAAATCATGTACGAAGCATGACTCGTCAACCCGTTTCCTGCCCGGTACTTTCTGTTCATGGTTAAAACATGGTGCGGGCAAAGCGGGCCTAGAATCCTGCCGGGCTTGGTAACCTCCGGCGCCGCTTATCCACAGAAAACGAACTGCCGAGAGATACAACACATTCGAGATCCACTGCTTGTAGTAACAGGAATGACAACAGAAATACACGAGAAACATTGGCAATACAAAGTAGTGGAAATACCAAAGCTGTTTTGAAGGCGAAATCGTTGCAATTGAAGCTGGTATTTCAAATGTTTTGGGTTGAAATCAGGTGCTGCAGGAATATTAATTACTGATAACCGTTATTAGCCACGGGGACAATATACGGTACCTTGCGTTGTAGCCGGTATAGCACGGCGAGTACAAGGTCCGTACCACAGGTAGTGACCAAAGTACCTATGATGTGCTAAGGCGTTCTACTTTGCAGACAACATACGTTTCGCATACTTACACAGCTGGAGAATGCCAAATTTGCATATTTTATATCCGACACCCCAGACCCTGAGTTGTTTATTGATGTGCAATAGTACCACGTATCTCAGCCCATTGATCTTCTGCTCTCAGGTTCTGACTCTAGCGAGCTGGGACTGCATGCCGCACCCCGCAATACAAGGGCAAGCTTTAGGTTTAGGTTTTACTAGGCGAATCCCCACAACCCCACACACTTCAGGCCCTAGCGTTTCTCCAGCTGGAACGACCCCCTGGCCCTAATCCCTATAGGGCTTGGCGACTTGAGCTTGACCGTACTCCCTTTATCCCCGCCGTACATCTCCTGGCACGTCTAAAGCATTCATCCCACCGGACCGCGCTACCGGCACTGAATCATGGTCTACTTGCGTGATGGACAGCACCTCTGCTCGCCATCGGAGAATGTGAATAGCGGATTCGCCCGGATTATTGAAGCGCCTTGAGGCACCCCCGAAGCTCGTTACGGTAACAGTCTTGACCGGGGTTCATCGACGCTATGCGATATTCCCTAAAATAGAGGCTAAGATTCTGGAATTTCAGTCACTTTGTGACTTTGGGTAGGTTTCCAACCGGTTTCAAGTCAGGTGGCGATATGGGCGAGGGGTCAAAACCAAACCAGTCCTCGCGCTCAACGTACGTTTCCCGGAGACGTG

AGAGATGCCCATCGACAGGGTGTCGAGGTACGTGAGTGGTTTCGACACTACTTCCTTCGAAACTACTTCCTAGTCCCTTGAGCATGCATTTAGTCCCCTGAACAGGCACTGAGAATGCCAAGGGCCAGCTTGCTGCAGGACAGCTTGCTGCAGGACAGCTTGCTGCAGGACAGCTTGCTGCAGGACAGCGATGATTCTCGTCGACGTGAACAAGTTCATATGATGTCCTGAGGGCTCCAGAGTCCAGACGTCGTAAGTTACCTAGGGTAGCTTACTCTACTTTTGTAGTGGATAACCATG

TCTTTGAGTGGTCAAGTGGTCGCAGCTGTTGTCTGACACACCTACTGCGCTTGGTTTACAGGTCCCTAATGAAGATGAACTTTGTCGTAGACTGCAATCTTTCAAGCTTGGAGACTTCCCTTCAACGACAGCCATAGGAGCAAGGAGCTGAACCTCGAGCCTTGGCCATACGGGAGTGTCTCGCCAGCCCCAAGCTACACAGCGTCCTCCTCAAGGAAATCTCCACCGACAAGGCATACTTCGAGACGATCCTGCGTCATGTCGGTACAATTGACCGTCTTACCAAGCTGATCACTGCGCTAGCGGAAACTTTTATTGAATGATCCCACTGCCGATTGACAGCCAGTTCGACGGCCTTTTGGAGCATTGATGCCAAGGGCTCACTTGGTGAAATATGGACCGTTGTTCGCTTACCGACACGCTGGTTTGAGGGTTCCATCGGTCTCGAAATGTGTTGCAAGAATAGATATGGGATGGATCCTCTGCATGGCAGGGTACTGCAAAAATAACAAAAAGCAAATATGCCGAATTCTACTTGTTAAGTTATTAAACACCTGACGTTCCCTGATGCCATCCCGATCGATTGATTCGACCTTTGGTTTAGAAAGCCAAGCTTTCAGGGTTCACAGGATCCCCTGCATCAAAGCTTCGCTGCAAGCCACACACCGGTGTGCCAAAAGATTGAAACAAGCACCCATGTCTCTTGATCGCATGGAGACGCCCCACGCTAATGGGACTTGTAGAACACACGCCCCTATCCACTCACAACGACACCCTTTTTTTTATAGAAATGGGCAACCTTGTTTCATTTCGACAGCCGAGAACCGGACCGCTGTCGAGACTCTCCGAGGTGTATTACACATTTAGCTCTGTATGCATATGAATATTCCGATCCATGCATGGATCCACCCCATACCTTAATTTGACAGGCTGGGTAGACGTGGTACAGGATTAGCGAGAGCTCGGTCAGGCATGCCTGGACATGTGGGATGCTGGGAGAGGGCGTGCATACAAGATTATGCCCGAGGCGATCAAAAGTTTGGGCAAAACAGCCTTGCTGGGCTCGACTCAAGTCAGGCAGTCAGAGGTAGCAATTTTTCCGATTTTTGACGTTATCAATCTTGGAGCCGGGCCCCGGTGTCGTTTTAGTTTTTTTGGTGGGCCAGGATTTTTACTTTTTTTTTTTCAGTTTGGCTATTCCGCCAGTCGGCACTATGCCCCTCCCAGATTTGTCATTCTAGGCATTTCTGTCTTTGTAATTTGGTTCATCTGGATCGCAAGTATGTCAAAATTCTCGTCTTTGACACACTATTTATTTGATGTCCTACAAGTCACATGAGGCCTCTACTCTTGAAAAGCCGTCGATCCTTCTGGTCGGAGCTACAGACTTGTACGAGGCTAACCATGGCTGACGGAGCCTCCACTTGTATCTGGAATGTGCCAACTGAAGTTCCAGAAGCTAGGCCGATCCGCAGCATCACCGTGTCAAAATGAGAAACATTAGACGCACAAGGCAGCCATGTCTTTACCACCCCAAATTTTTGCTACTTGGTCCAAGTTGTGAATGAGATTTGCCAATCTTGGTCGTCCCCCACCCCAGTAATGCGGGTTAAGATACAGCCACACCGCCATATGATCATTCCGCACAATTACCCAAAGTGGGCATTTTGGCAAGATCCGCGCATCACGAGCTCACTTTTTGATCCGCAGTCTCCTGATGACCGTCTTGAATAAATGCGATGCGCTTCACCTGCTACAAGGCAGACAACAACGCAGACAACAACGCAGAAAAGTACTTGCTGCGTGTCGCTGTATGCTTGCTGAA

**Chr.4**

**> MD401_chr04:1521192-1525192**

AAGGCTATGTGAGTTGGGCCGACTCTAAGCAAACTACAATGCTTAGATGATGGAGGCCATCGTTACATCACCCAATGTACGAGGTTCGGAGATACGGATTAGTTTCTTGTTGATTGTCCTTTTTTTATGGCTGCAATTCAGCCACTTTAGAACTTGGTATGGGAGGATTGTTTTTTTATCCCCCATATCATTTTCTTCTTGTAACATTGTTAAAGTCGCTCTTTATCACTTCTGCCTACCAAATCTTGCCCAAGTCATCACGCCACAGCCCGGCCACATAGTTCCCAGCAACCAAGTTTAACAGCATATAACGGTAACGCATTACATCCATTGTTTCTTTCATATCAAAGAAACGCATATGGCTAGCTCATACCAGCCCAAGCGAAGCATCACTCACCACAAAATCACCAAAATCAAAAGCTGTCTTTCTATGGATGTCTTTGTAGGGGGTTTCGTGTCCCCCATAAAGGCCGTGGTCCTATCATCAATTCATAATCTGGTGCGCTAGTAGAGGGCAACTTGTTTTGTTCCTCGTCCTTGTTTTTTTATTTTTTTTATCTTGTCTATTCTTTTTTTCGTCTTTTGTTTTCAGCCTTATGCTTTCATCGTGAAGCTATGAACAAAATGGGAAGAGGAAATTTATTTGTATTGGGATGCCGGGAAATGTGGATGAGATACATGTCTGTGTACATAAAGAAAAGATATACCCCTACGACGATGGGCTGACGCTCGCTATTTATTTTTTTTGACTACATATGATACAAAATGTGTTTTTCCTTGTTGCTCGCTGGTCTTTTCCCATGATATATTACTACGTATCAGACATAACAAAAGATTGGGTAATGTTGGAAGAGATATGCCCACCTACCTATTCTGGGTGCAGACCATCGCACAACCTCATCTGAAAATTTTGGGGCCACTCTGTTATAGGAGAGAAATGGCATCGGTAGTTTCTGTCAACCCAAGCCATGAGAGGGCCTTCATACTGCTCCCAGGACCGATATTTCACGCCAGAATTCGACGAGCCTGGTCTATCATCTTGCTTGACACGGAGACGTCCAACTCTTCCCACACTGCACACCACATATGTTCATCGCAGAGGACATGCGATTACTTTCTATCAGGGGCTTTGTCATGAGCATCGTCTACCCAGTCATCGGCTGGTCCAAAAGAAGGGTTCACCATAAATGGGTTACGAGAAGGACACTGGGCTCTAGGCGGAGGAGCATCTGCTAGACGACATTGCAGCCTGGTTACTTGCGGGAACGAGACTGACGGGTGCTCTTGGACTGCTGTGTAAAAGCCGACCACTTATGGCCATGGTGACCGCCAATTACATACAGCCGAGTTCCATGCTGTTCAAGTGATCCTCCTAGAGATCACACTGTAATAGGCACCGCTGACAATAGGAATGAAGGTCATCGGAGATTTTTCACCAAGATCCATGTATAGCTTCTGCCTCGAACTCCTCAAGGTACGCAAGTAGCCGAAACGCCACGCCTTCGTCACACAGCCACCTTCTTGTGACTCCTGGAGAAAAAAAATGTACACCTTGGTTATGCACTGTTCGAAATTTTTGAGAATTAGCAAAATTGGAGATTGACTAAAGGCAAACAACCGGATAAACGGATAAAGCTCTGCTGTGTCTTCTGCATTACTTTTTTCTTCCTTTTTTCGGTTAAATCTTCCCACGATCAGTGAAACAGGATATCATTGGGGAGAAGCATCCGCGAATTTCGACTTCTCTGACGGTGCGTTAAAAGTACCAGTGTATTCGGGTTTCTCGTATTCATGAGGGGTATCTCCTAGGTAGGTATATATAAAACATGGATTGTATAGCTGTTACGAATCTGCCCTTCATTCAGGCTTAGTACGTTTATGGTTATGTGGATCTCTGCTGACGAACGAAAAGAGAAAATCAACGTTATTTTCCTGCCGGTCTCATCGTTG

CACTTACATCAACAAATCTCTCAACAGCAGCAGGACTGATTACTACAGAGCCCCCCCCCCCCCCCCCCCCCAACTTCGAGGCAAGGCTTCAAACTTGTCCTGGCAACTGTAAGACAACCACGTGTTGCTGGCTCGCCTATCTCAGCTGTC

AAAGCTGGGAGCAGTGTTGCTCACAGTCACCTGGGACCTGGGTGGCTAGGTAAGGCTCTTATGTGATTTACTTTCCCCAGGGATAGCCGGGTTGACCTCTTTTTGCTACCCATTGTCGTCAGGTCTATAGGAGGCTGAGTGGGAGGCATCCAATTCCATCCTGTTCGTGTGTGGACTCGAAGATCTCAGGTTTAGATCTTTTATTGGGATCCACGTAGAACACCCGCCGAATTCACAATACTCTTAAAGGTACGTGAAGGGTCAACGGTAAATTGCTTCGTTTTCTCTTTCGTCTTTACCTCTTGTGGGGTGCCTGGACTTTCTATTTTTGGCGAGTTGGGAAGCTCTTTTTGAGTGACTGGACTAGGCGGCGGTGTTAATGGAAACTGTTGCTCAGGCTGTGGTTTCTTCTCCACTGCCTTTGGTGTCTCTGGACGTTTGATCGTTAATAAATCAGGAAGCTTTTTATCAATGGTTGGACTAGGCGGCGGTGTTAATGGAAACTGTTGCTCAGGTTGTGGTTTTTTTTCAGCTGCCTTTGGCGTTTCTGGACGTTTGATCGTTAATAAATCAGGAAGCTTCTTATCAATGGTTGGACTAGGCGGCGGTGTTAATGGAAACTGTTGCTCAGGCTGTGGTTTCTTTGGCGCTTGTGGAGTCTCTGGATGTTTGATCTTCAACAAGTCAGGAAGCTCATTATTAGCAACTGGACTGGGCGGCGAACCAGAGGCAGCCGATGAAGATGAAGATGCTATAGATGGAGAATGCTTCGCCGGCTGTGTATTCATTTGACCTTTCGACAGCCATGAATCTAAGGTTGTCTGTTTCTTCTCCGCTGCCTGTGGCGTCTTTGGGCCTTTTGAAGGTGGAGGAGGGGGCAATTGGCCAGAAGGAGATGACGAAGATTGAGATGATTTTAATGGCGACGACTGTGGGACTGGGGCAGGCTGTGAACCTATATTTGTAGCGGTCTGTGGAGCATCTGTTGGCTTCTTGGGGTTTTTTTCCTTCTCGTGCTGCGAGACTGTGGGCGGCTCATAGGCCCTGTACTGCCCTTGATATGGATCCGGGCCAAGATTCGAATACCATCCTGCGCCCCATGGTGCGCTTGACTTGAATGGCTCTTTTGACGGATTCCCAGAAGGGGGACCGGGTTTGAGTGACGCTGACACCTTAGCAGACTGAAGTGAATCGACCTTGGCGGGAACCGGTTTCCGCTTGATCTCCTCTTGTCTTGGGGGCGGCGTGAGTGGCCACGTATTAGACTTGGGAGGATACTGTGTGTTCTTCGCTGTCGCTTGCTGTTTGGGGACGCCACCATGTGTCGTTTTCTGCTGATTCTTGTTGGGGGGAATCCCTCCCGACGTTGGAACGCCCTGGGCACTTTTCTTTTGCCCCCATAGTCCCCAGCTCGACGGTGTCCACCACGATGATGCTGTGGAGGGCTGCGATTTTTTCGGGGATGGTGATGATGATGGTGATGTCGAGGAGAAGGGCCAGCCAAATTCCCACGGATACGGTGCTCGTTTGCGGATCATAGCCGTCGGTCTGGATCGGCGCCATTCTTGGTCTGGGAGGACCAGTTCTGGTGATGTCAGGTGCGGCGAGGACGAAGGAGGAGGTACGGGCAGGGCGCCTCCGAAAGTGAGCAGCAGGATAGTGAGTGAGAGAAATGTAGAGAAGAACATTGTATGTTTCTCCTACCCCAGACGAAAGCTGGTGGTAAATGTCAAATGGCAAGATATAAATCAGGGACGGGGTTGATCGTCGGTCCCAAGGAATTCGTTATTGTTATGCATAAACGGTTCTGCATGCATATCATCAGTACCTAGGCAGGTCCTTTAAGGTATGCAAGCTGCGGAAGTTCCCTTAGGGAAAGTCGAGGCGATCACCTCAGCAG

**> MD402_chr04:2234773-2238773**

TCAGGTTGGCACTCCGGCTGATTTCCTCCGCAGAGAGCGGCCCAAGTGGGTGTATTGGGTACTGAGTACTTGTGCCATTCTTCTGATGCTGGTCCGCAGCGGCCGATTCGGGCAGAGATCCCATGTTAGTGGATCGAAAATGTTTGGTAAAAAAGATCGGATGACGTTTATGTACAAGTCCGTGGGAGGCGCAATAGGGGGTTTCTCAAATCTCTCAACCAGAAAAAACCCCCAACGGTAAGCCAAGGGAGGTAGGGAGGTCATTGTGGTTATATATAAGCCTTGGAGGGGAGGCAAGAAGCATGCAAGTGAGATAGGAATAATGGAGATAGCCGGTGGTACATAGTTACATTGTTACTAACTGTCCTATGTTGGTTGATCCACCCGGGCATGATCGGCAAAGAATTTAAACTACAGCCGATTGGTTCTCTAGTCTAAATCAGGAAGCCAAGTGGCTAGCCTACAAGTCAATCATGGTTCTAAATTGTCTGTTGACACGTTTGACATGAGACCCCGCCCTGCCCGCTTGCCTTGACGCTTGTTGCTACCAAGCATGCATGGAGATTTTTAAAACATGACAAAGTTGGAACACGATACCCCATAGATTACGTTACCCCTCATTGATATATGTATAGTGTGGATAATGCGCTTGCGCTTGCAGATTGCCAGTGGCGACTCATCCAAGATACCCCCGCGAACTCGGAGGTATTTATATACATCGTATCTTGGGTACTTTGTGTACAAGCCTCGATTGCAATTACACATCAATCAATGGTCAAAAAGGGTCCCCGGTCTTTTTTTCCGGAAAAGGGTGGGCGCCGGGTTCTACTTCAATAACCTGCGATAGCCAGAAATCATCCAAAACCTCGCATACCACGTGTGCAGGTCATCCATCCACGGCGGCATGGGAGGCTTGGCCTCGTCGCCCTCTAGCTTCCTCGAGCTGGGGAACAGGGTGCTCTTGAGCCCCAGCTCGACCTCACCGCGCTGCCTGTCCACCACCGCCGAGAAGATGAAGAGGCCGTCCGAGTCGCGCGGACCCTGGTTGCGCGGCGAGTCGCCGCACCGCACCGTGATGGACGTTGGCGTCTTCTCCAGCACCTGGAAGTGGTCCGTTATGATGGTGCCCGGCTCGTACGTCGAGGCCGACAGCTGCTCCTTGGACCAGAGGTCTTGATCGGTCGAGGTCGAAGCGTTGTGGTATTTGCGTGCTAGGTATCGACGCTGGATTTCGTAGGCTGCAAGGGTTTTGTGCGAACAAAAAAACAAGGAAAGAATTCGTTTCAGAAAAAAATATAATCTGGAGGAGAATCATTGACCAAAGCTTCCGAGTGGTGAGCTGATTTGCTTTTGTGGGTTGGCACACGTACCAGGGCTAGTCCAAACACCACGGCAAAACTCCAGGACGATGTCGCCTTCTTTCTCCAACAATTCCGGTCGTATCTTGTCGAGGGGCACGCGCTTTGTCACGACATCCTGGGTCGGCGGGTTTCGGTGGATGTTGTGCTTCTTGTACAGCTTCGACTTGAATACGCTATCGTTGACGGGGAGCGGCCTGGTAAGAGTGGTGGCGCTGCCGATGTAGCCCAGGGTAGCTGCTGCGGTCACTGTGCCCGCGACCAGGGTTTTGCGAATAATGCCCATTTTTGGCGACTGCGATTGCTTGTTTCTGGTTGTTTGTTCTGTTTATTGCCGTAGGCGACGCGTCTCGGTTGACAGTAGCTAGCGCTTCTCTCCTCAGTTCCTTGGTGGAGTTGCGCGAAATCGCCTCAAGGTCGGCGTAGCGCTTCGGTGGGTGCG

ATGCCGGACCTCGGGATTGGAGTGGAGGGGTTCCAGGTACTTGCGGGTACACTAAGCTGCTACGTCACATCGACCCAATTTTAGCAAATCTCGGTCTTGAAAAGCGACCACATCAAATCAATATTCAATTTACGCATCACCACCACGAGTGTTGACTTTGATCCTTATCAACATGTGCTGATATCATCGGATTACTTTACATATATATATATATATATATATATA

TATATATATATATATATCCAAACTATAACACTGTTCCCAGCAGCAAGCTAAAACTGCCATCTTGTGCTGATTTGTAACAACGCCAAAGTGACGCTGCAATCAAATTAAATTCGGATGGTTCGGAATATCGAGATGATAACAAATAACGTTGCCTCTCTAGACTGTCTACTATGCAAAGTGGGATTTAATTTCTGCTTTTCGTCCTGCTGCAGGTTGGTCAAGGAACGTCAGAGCAGATTACTGGCGCAAGACAGCACATAACTGTGTGGATTGCATAAATTGCGAAACGATACGGTTTCTGAAAGATTAGCAAGCTCTTCAAGCACCAAGGTCAATATAGAGAGCACACCACACACATGTGACAAATGATGGAATATTTGCGGTCAATGGCAATTTCTTTATCTCTTACAGACGCGGTATCGGTCTGATTTCGTCTCTAGCCTGGTCATTAATCCGCTTCCACATTCCTTTCCTCAGTTCATGCCCATCCATGAATGCATCGAGCCACTTCTGGTCGAATTTTGGCAGCTTCCTACCCAGCCCGGGCTCACCGTCTCCGGCCAGCTCTCTCAGAGTCTCAAAATACTCTTCAAAGTACGGGTGGATGACGGTGAACTTGGGCCCATCGCCAGTTTCTGCGATGCGATTGACTTCCCACCTCTGCATCTCGTCAAGAGACGGCAGCGGCCTCGAGGTCCTGCCGGCTAGGATCCGAGCCGCCAGGACGGCCTGCCACTCGAATATCTTGAATGTCAAGCCCGCTCCGACCGCCCCGACGAACAGCAATGTCGGGTCCTGCTGCCAAACAACGTGGTGATACAAGCCCGGCACGCGGTTCTTTTTCGGTTGGATTTGCGGGAGGAACGGCAGGGTCCAGCTGTATCCCGTGCCAAATATCAAATAATCGACTCCGCTTGCCGACGTGCCGTCGACAAAGTATACAGTGGCCCCTTGGCCACCGGGTGGAACTTCGACTCTAGCTATCGAAGGCTGCATCTTTACGTTTGGGTGGCGGAAAGCCTCGTCGCCAAAGTATGGGTTGGCGGTGTGCCCCAGAAAGACTGCGTGCACGGGGGATTTTGCAACTTTGACAATGTCAAACGCAATGTCAGCGGCTGACACAGATGCGCCCACCACCACAACTCGCTGTTTTCCCAGTTGTTTTTCCTGATAAGCAATCGCCCCCTTTCACAAAATACTGGGGGAAAATCCGGGCTCGTCTCATCTGCCCCTTACCTTGTCTCTGAACTGATCCCTACCCCGGAAATATTTGCTGTGCAAAATACTCCCCGGCATAGCCCGCTGCATCTCCTCCAACCCCTCGACCTCGGGGACGTACGGCACGTTGTAGTGGCCATTCGCAACGACCACGGCATCGAACCTCTCGTCCCACCAGTGATCGCCCCCGCGCCCGTCCGCCTTGCGCAGGACCACCCGCCACTCGTCGCCGACCTTCTCGACCAGCTCGACGGTCGTGTCGTAGGACACAACCTCGCTGTGCCCAGGCGTGGCATGGATGACATTGCTGACGTACCGCTTCAGCACCTCCCAGTGACGAAACGGCGTGGAAGGGCCATACAGCGAGACGGACAGCTCGCTCCTGTCGGCCGGGATCGGCTCCTGCGAGAACTGCATGGACAGGTCCTGCACGTTGGTCTCCAGGTACGGGTAGATGGACGAGTCGGCGAAGCGCGGCTGCGTGAGGGCCGCGACGCCGTGTGCTGGGATCTGCTCCGGGATCGGAACCGGGAGGTCTGCCGTCCGGTCCGCAAGGCGCTTAAAACCAGACAGGTGCGGAGGTTCCGCCGAGTCCCCGATCCTTTATTTTGTGCAAGAGGAAGAAGAGGGGCGGCACAAAAAAAATCAGCACTTGAGCCGCGGGTTTGCGCATTTCGTCGCAATCATGTCGGCCACCCGTCTTTTCCCACTCACCAGCATCCTCCAGGGCCTTCTCTGCGCTCAAACACTCGAATGA

**> MD403_chr04:2431428-2435428**

GAAGTACTACAAGGTCGTCTTTCGTGGGCTCGGGTTCCCGGTTAGTGTCCGCGACAAGGAATTCGTTTACGAGGGCTCACCGAGCGAGAGGGCATCGGCATTCACGGACCGCATTCAGGAGCAGTACCCCTCTGCTCAGATCGTCACGAACGAGAACGTAGACGACGTTGAAGGCCAATTCCTCGTCATATCGGTCATAAGCCCCGATCGTGACCTGGCACACCTCGTGTTCCAAAGGTCGCGAGTACCACAGCCGATTCGCGAGTATCTTGTGACTCCTCACCCACAAAAGTTTGCTGTCACGTCAAAGCGCAGCACAACCGGCCCTCCTTCAGAGCACTATGCAGAGAAGACCGTTTATGTCACTGCCGACGTCTTCCCGACAATCTTGCGCCGCAGCGAGATCGTTGACAGCTACAACATTAGTCTTGATGCCAAACAGACTGCCCTCGAACGCATAGTGCGGAAAACGCAAGAAATGTCTGGCGTTGAGAAGAGGGTGCTTCAAGGGGAGGGCGGCAAGGAGTTGCTTCAGCTGCTTGTTGATGCAGTCAGCATATCAGTCAATCCGAACTCGGAGAACAGTGTTGCATGCTACCGGCAGCTGATAGCTTCGAAAAAGCCACCGCGCAAAGATGACGAAGATTCCGATGCCGATGAGTCTGATGACGATAGCGACGACGACGCGGTTCTCGACCCCAAAGAGAACGCCATCAAGATCGCGCTGATTGATCACGCCATCATGCTGAAGCGTGTCCTCGCCAACATGGCCAAGAGCTCTAATGAGATCCTTTCTCGCCACCATATGGATCTGCAACGATGTAAGCTCTGCACTTTGATTTCATGTGCCCTTATCATATACATACTGACATGGCGTTACGTTATAGACTTTGAGAGCACTTTCGCATCCGAGATAGCAACGTTCGCCCCGCCGCAGCCGCAGCAAGAAACCACGGGCTCTGTCTTGTCGCCGACATGGTCACGGACTTCTGCCGCAGATCACGCGTCTTCCCCTGTTGTACGACAGCAGGGCTTGATGTCGCCCTTCCTGACGAACAACAGTATCATGGCGCATCCAGACGAATCTGTAGTTACTGTCAGGCCTGTATCTCTACGACAAGGCAAGGGCGCGCGGTTGAGCTTCCTGGGTGGCCGCAAGAAGGAGACTCCGCCCACGCCTACTTCGCCTCTGTCACAACACCAGGCGAATTGGCAGACACAGCTTCCGCCTTTAAACACCAGTACAGAGCCAGTGAAGACGCCACGGCAGCAATCCCAATCTCAGACACCGATCGACCGCGAGGCGAACGGAGATCTGAGCCGTATCAGTAGCCGCGATACGAGCACTGCCAACAAACGTCGCAGTATTTTCAGGCCGCTTTCGGTCCTAGAAAAGCAGGATTTCTCGCTGATGACATCGACGGCCACCGTTACGACGAACGAGAATGGACCCGACGCTAAAGACGCCGGAGACTGGTTTACCGATTATTCGGCAACGGTAGGGTCGGGCGTCGGCCAGACGGGCGGATACAACAACGATGCCCCTGGTGGGCGCAGGAGCACTGACGTTAGACCGTCCGCTGAATCGCTCCCCTCAGATAAGTTCGTCTCGGGAGGAGCTGCGGGTGGACCTGCTGCCAAGCTCGGCAGTGTACGCAAAAGATTGAGCATCTTGAAACTCGGACGGAAGGGCAGCAAAAGCAGTACGGCCATGGGAAGCGTCGATGAGGAATGACGGCATTCAATGCATTGAAATTCTTCTGTCTGGATCTCTTCGTATTCTGCTCTCAGTTTTCTTATTTCCTTCTACTTATTTCTGCACCTTGCCCCTTTGGGATATATATACCGGCATTTGCTGCGCGTGATGTGTACGCC

ATACATCATGTCTCCTTTTGTCTGCTCTGGCTGGTTTCCCCCAGACATTAGCCCTGGATGTAACCATTGATTCCCTGCCATAGATTTGTGTGCTGGCTTCGTGCGAGTTCAATCTTTGTATTTTACCCCCCCCCCCCCCCCCCCCCCCTTCTGCACTTTTCAAGGTTCTATTTCTTGCACGGAAATCTTTGTCATATACCATTTTAGTGTTTTTTCTCCTCCAAC

TTTGTGTTCCATCACATAACAAAACCACCTTAGGACTTTTTCAACTGGACATACATACCCCCTTGTTTTTACGAGCGAGCGAGTATCTACTCATAACCCACCTCGTACCTTCTTTTCGTCTATTGTGTTAGTTCATAGACCTTATTTTAAATGAGTAGTTCCCGGATTGCTGGCAGGAAACTCGTCTGTTTGGCAATTTCGGCAGCAAATACTGTACGCGATTGACTATTTTACTGGGTATAAAAACGTTTTGTAACAACACGATAGATGGTTTCTTATGACTTTTACGAAAGAGAAATTTCTTTTTTCCCATCATGTTTTTGTTGTTTTTATGAGCAGACCTTGTACGGTGAGTGGATGCCGAGAGCGGGAGAAGTGCCTCCTTGGTTTCCCAATTCGACAACTACATTACGGCCCGTCAGCCGCCGGAGCGTGCTGTTTGCGTCACGTCGCAGCGACAACACGAGTTGAGAGGATGCAGCACCCGTTCCACCAAGACAGGGGCTTGGCCAAATGTGCTGGGGAGATTTTGTGTACACCATTCAATTTCAGCAGCAATATATGTAGCCACTCCAGATTGGTCGACCCAGAGACCAGGGCTGGTCCGCCTGTGAGACCAGGTGAGCCACAGACCCCTCGCAAACCGTGGTTTGGATAGCCATGGCTCGCTCCGATTGCACTGAGTTTTCCGGATCAGGCAGGCCTTTAATCACGATCGGTTGGCTCATCGCCCTCAGAGGGCTGGGTCTTCCCGGGGAAGCGGTCCTTCTATGTTCTAGTGCTCGGACGAGACCTCCGCAAGTTAGTCCTTGGTGAGCTTGGTATCCATCTGTTATAGTGCAAAATAGACTAGGGGCTCGGCCTTGGTTTGAAGGTCTAGATGCGCCAACAAAAAACGAGTCACGGTATCGCGCATGTCGGCGGTGCTCGATGCCTCTTATATAAGACTGCCAGAGCTGCCCAGGGTGGTTGGTATGCTTATACTTGGTCTTTTGCACACACCACCTTCAACAGCAGCGCGTAGTGTTCCCTTCCCCATCATGAAGTCATACGTTTTCTCAGTTATGGCAGCCTTGGCCGCGAGAGAGGTGCTGGGCCATGCCACCTTCCAGCAGCTTTGGGTCGATGGAGTTGACATGATAAGTCTCGGTCTTTCTTTATGAACGATTCAGAACCGTCCATCTCGAAACTTAAGTATCAGAGAGGTGGAAAGATGAAACCAAATCTAACAGTAATTTCGCAATCCACGGATCGCAATGCGCGCGTCTCCCGCTGTCCAACTCGCCCGTGACAAACGTCGCGTCCAACGACATTCGATGCAACGCCAACTCCGGCCCCGTCGGCCGCAAGTGTGCCGTCAACGCCGGCTCGACGGTCACGGTCGAGATGCACCAGCAGAACGGCGACCGGTCGTGCAGCAACGAGGCTATCGGCGGAGCCCACTACGGCCCCGTGGCCGTCTACCTAAGCAAGGTCGACAACGCCGCAACCAGCGACGGCTCCGGTGGGTGGTTCAAGATCTTTGAGGACTCGTGGGCCAAGAAGTCCGGCAGCGGGTCAGGCGATGATGACTACTGGGGAACCAAGGACCTCAACACGTAAGTCTTATTTCTTTTCTTCGTTTCTCTCCCCCTATCTCATTATGCGCCCCTCGGCTGTCGTAGAACAACCTTGAATGCGTCCAAAGCCCCCGGAGTGTCAAAGAGGCTATCTAACAACCCCGTCACAACAGCTGCTGCGGCAAGATGAACGTCAAGATCCCCTCCGACATCGCCTCGGGAGACTACCTGCTCCGCGCCGAGGCGCTCGCCCTCCACGCCGCCGGATCCTCGGGCGGTGGCCAGTTCTACATCACGTGCTACCAAATCACCGTCGCGGGCTCCGGGTCCGCCAGCCCTGC

**> MD404_chr04:3164700-3168700**

CAGAATGTTAAAAAGGGGGCTTAAGGATACTCATTCAAGTGACGATAAACTTGTGGATAATAGCATTCTCCATCTTCTTTTCTCCACTCGGAGACCTAGTACAGCCCCAAATCTTATTCCATAATGGAGACGCCTCTTCTTCGTGTGTCTCTCTTATAAGCCCACAAGGTGACTCAACCCACCCGTGTTCTTCTCGACAAGCTGCTATATCAGCTATCATATCCGACGCCCTTGTGTGTGTTGCTTTCTCGCCATGTAGTCACCAGATATGCAGGCGAGAGTACTCCCCTGAACTGCAATGGATGTCACCGGCTTAAATGGGAGCAGTCACTGTGATGGCACAATTACTTGCCATCGTGTCAGTGATCTCAGGGCCAAACAACTAACTATTCACTTCAGGTGTCGTTCGACATCCTTCCTTAGATGCTCCGTCTTCGATGCAAGACCTGTCCATGTTCTTCTCCATCTGGACAGAAAAAGTTAAATATCGTTGGGATCTGTGAGTTGGATCAAAGTCAGGGCTCTCCTTCGCATTGCCACTCTTGCTTCATTTCGAAAGTGACAGCTTCAAAAGCCACAGACCCCGTCGGTCTATCCACAACCCTTAATTAAGCGCCGTCAGCACAATCGAATACTCGACCAAGGACCCATTCTGGACACGGAAAGTGCAACACCTGGCATTCCATCATCAGATTACATATCACCAAGCCCTGTTCTGCTCCCTTATTTCTGGACAAGGAACCCGTGGCAGCATCCTCCGAAACCAAGGCGACGGTCCATGTCCAGAAATATCAGATGAGCCGGCCCGGCATGCTCTCCTGGAAACGTTGCCGTGGCTTCCGCCATCCGGAACCCTGTGCCCACCGGAGAAAATGTCCCCGCGGCTCCAGCTCACCGGCCTCCCGTCCCGGGCATTCCTGAGCTGAGATCTCCTGCAGTCCGATGAGGAGTGTGCAAGAACAATCTACGAGATACAGAGTGACGGAGTCGAAAACAAAACCATGTCAAGCCAGGACGCGATTGAGTTTTGTTTTCTAGACGAAGCAGCAAGCCCGAGATCTGGATCCAGAGTTGCATACGCAAAAAGGCCGTCGACTCGAGCAGCACTGGTGACGACTTGTGTCTTTATTCACCCGTCCGTTTCGGCGATTAATGCCATGCGGTGGTAGCCCCTTATCAAGCGTGAGTTTGACTTGCAATTAGCCAGCAAGCCTTCACACCTTGTACGAACAGCTTCGTAGTGAGTAAGCAGGAAAAATAACAATTGATCAACTCGGGGGGACTTGACACAATCACAGGGGCCCCTTCTTTGTTTCGATTTGCATTCCATGATGAAAAAATATTCCGAGCATTCCTTCGGGCCAGACGTCATCGCTTCCGGCCGGGCGAAGACCATTTTTGGGATGGGCCGGAGGAGGAAAAACTTTGGGCTGGAAGTTTAAAAGAATAAAGCAGCAGTCCGAAAAGCCCACCGATACAGTGCGGAAAATCTGGATTTATATAGAAGCTGTTGATCCCAAAAAACACTTTTGCCAAGTTTTTCTTCAGCACCGGAGCATGTGAAGCCCGGTCATGACGACCGGAAGCACGTAATCTTTGTTGTTTTTGTTTGTTGTGACGTCGTTTGCGTCTGTTTGTCACACTACGTTGGCGATCTCATGTCTCACGAGAATGGATGGCACAGGTAGCCTAGCCTCTCGTTCCAGCTGGAGGGTAGCAAACAGCAAGAAAAAAATGTACATCAGTCATGCACTTGACAATAATAGTTCGAAGATATGAGTTTGCATTTTCATCAATTTAGTTTTTTGATCAACGGCGGTACAACGCAAAAGGTTCACCGGTAGCATCGCGAGGCTGTTCATAGCAGGAGTTGTG

GCCCCAAGAGTCAATACTACCAAGAACTTGTCGACTCCCGGAGGCCTCACCCAAACACGGGACGAGTATGTTGTACGGAACCCCCAAGAAACCAACTCACCCTTGGGTTTTTAAGATTTTAGATGCCCCCCCCCCCCCCCCAAGGCATACTGTCGCGCGGGTGTCTGTACGCAAACAAAACTCTAAAAAAGACCAACATACGGATAACTTCCCCCGGCCCAGCCC

TGCCATGGAATTGTTCTAGCGAGGCCTAGCACGCGGCAAAAGATCAGAGTTGTTTTTTTTTTTTTTTTTTTTTTTTCTGTCTTCTTACTTCATGTGCCCTACCCGAAAATCCCGAAAACAAAAATACACCCCCTCCGGAAGAAAGGGGCGTGGTGCTCTGTTTAGTAAACAGAGGCAAGCGGGTGAGGCACAAAGGGGGCCAGCGGAGAAAAGCCCGGCGGCACGACTCGCTCCGCAGAGCTTCGGAGGGGGCTACCACTACACCGCAGCGTAGCCTCCAGGGGGCCCGGGCAAGCCGCCGGTCCAAACCTTGGGCGCCACCCCTCTTTCTAAGCTTTTTTGTTCTTCTTTTTTCTTTTTCCCGTTTTGGGATGAACGATTGTCAAAAAGGAAGAAAAAAAAAAGAAACGTAGCAAAAGGATTCTGCAGCGGGTAAATGTACGTAGTAGCAATTGCTACAAACACGAAAAAAAAGCTCGACAAGCCACGACTCTTGTCGAGACACGTGGTGAGGCAAGGTGGCCGTCCATCTAGTATCAGAGTTCAAAAAGAACGAAACAAAACACCCCCTTATTTTTTTTTTGTTTTTGATTTTGATTTTGATTTTGATTTCCTTGTTGTTCAATGTTACGGTATGATGATGCAAGCAAAAGTCAAGATGGCGGGATCGCGTCCCGTCTACCGTTGAGCTGAGGTCTCTGAATCGCAAAGCCAGTGGCAAAGACTCGTGCATGCGTTTGCAACCTGAAGGAAGGTGGTTTCGCAGAGTTGAGTCAACCCTGACAGATTCGGTGGGTGAATGAGTAGCTAGGTGGTTTTTGAGCAACGTGGATTGTTTGGATGGTCAGTATTTGCCGATCATATGCAAATCACTTGCTGAAAGATGGATGGCACAGTCAAAATACTAGTAGCAAGAATCCTTTGTATTCAATCCGCCACAGCTATCACCCTCGTCTCATCAAGACGAAAATCGTACGAATGCGTACGCCCCTGCAAAATATTTGAAAAGCAAAATAAACTCCCGAGTACCAGACCCTTCTACAAGTAGTATGCCCAAAGAATCAATCTATGACACCGTCTACCGGAGAATGCATAATGCCATGTTGTAAATCAAAAACGCCCCATATGCCTCTTGTTGATAAATAAAAAAAAGACAGAAACCGGACAATGCGTAGACCCAGGAATTAAAAGACAAGTTGCGCGCCGGGACAAAAACAAGAACAAAATAACCGAGACATGGGGGGGAAAAGAGTGAGAGTATGCAACCGTGGTATCATGTGAAAAAGTAGAGAGGGGGAATAGAGAATCAGGACTGCAGCCAGCCCATGTTCCCGTGACACACCTGGAATATGGAATAGAAAGATGGGCAAATCTAGTGTATAATATTGACACCCGACAATACATGAATCGTGGTGTAGCGAAGGGGGGGTATCATAAAAAAAATGGAGGGGGTATATTTCATTCGGTGAGTTGTGCGCAATCGGCACACAGGATGCTTTATGGTGTCAAGATGGAAACAAACGGCTCAGCCACGTAGGATGCAAGGACCAAGGGGGAAATAGGAGAAAATAAAAAAGAAGAAGCGCTAGAGATCTCTGCACGCTTGAGGGGCTTTTTCGAAAGAACCAACAGTCAGAAGCATGGTCCTGACAATTTGGCGCTTCAGGGAGAAAGACAAAAAGTCCAAACGCGCGGACCCCCCCGATGGTGCCCAAGTGGCATGTGACGGTGCGACGACGCTAGCGCAGCGCCATGCCGCAGCGACACCGCACATCTGCGTCCGCAGCGCATGCGTGTGAATCGTCGATCGTCGGAGCGGCTGCAGACGTTTCCCTCCCCCAAAGCAGTCGGACAAATTGCGGTGACTTTTGGTGCACATTGGATGGAGTGGGTGTCATTC

**> MD405_chr04:3690872-3694872**

GTCACTCCAGACCTTTTGCAGATACTGCGACGTTGGATACCGTCCTGACAGGCCATCAACTGGCACCGGCGCGTCGCCAAAGATGGGGATTTGATTGCCAAAATGCAGATCCGGATGGTTCATTACGAGGCACGTGCTGATGATATTTTGAAACGTTGTTAGTCAATTCGTATAAATATATAAAAACAATCAGGAGATAAATGGAGACAGTAAGCTGAGTATCGGCGCGTCGAATAAACAAAGTTCATACTAGGGATAGACATTGCGTGTTACAGTAGCTTGCTTACTTTCAGGCGCGGGACGGGACGCGGAAGTTAGTTGTTGTGGCTGGCTACCACAGCTGAGGAGATACACACACCCCCTTGACGCGCAGCATCAGAGAAGCGAGCAATCCAAGCAAATCCTTTCCATCCATCCACCATTAGGCAAGAGGGTATGACGAAGAAACCGACGACCAATTTGATACGCACAGTTTACCGTGATTGGGTAAAATGCAACACGAGCAGCTTTTCTGCAAACATTTGTATAAACACGAAGTACACAAGGAATACTGGGTATTTTACTCTCATGGTTGCCTGATTTATGTATGAGGCCACGGTCGCATAGGCTACGTCCTTCGGCCTACCTATTCACGCGGGGAATTAGCTTCCGACATGGCAATTGCGAAACGAAAGTGTAAATTAGCCAATTAACAAAGGGCTGAGACAGGTAGCCTAGTGCAGCTAATTGGACGAACTACGGTGAAGCTGTCTTCCGTTGCAACGGAAAATCAACCAGGGATGGATAATACTTTGCTTCTTCCTGTTGCAGTATTTCTACTTCAACTGCACGCTAGCCCAAAGGGCTGAAAACGTCGCGCTAACCATGCGAAAGGTAAAAGCAACAAACCAAAATAAAACCACCGCATCAGACATTAGAGCGTCGATAAATCAGATGAAAAGATATGTAGGACAGGGTGGTATATGAACCGTGCCCAGCCTCGTGCAATTGTCCTAACTAGACTACTCCCAGACTGGAGTCGAACTAGGGAGGGTCCCAAAGATTCATCATCCGAACGGATAGTGGTTTATCACATTGCGAGTTGAGATAGTTCAGACAGTTCAAGCGGTGAGCCAATTATTTGTCCGTTGTCAATTTGTTGTTGTTTTGTTATGCATTACCAAATTGGGTATACTGTGGAGGCGAACCCAGAAAACTATGGCGTGTGCTCGGACTCGAAAGCGATATGTCTCTTTTCGGTTGAGGGAACCAAGTGGGAACTGCGTGCTGCAGTCCGGCGATGAACCAAGATCTAGCGTGGTTAGTCATGGGAAGCGAATTTTGGAGTGTCACCTCGCTGAGAAAATGGAATTCTTTGAGAAGACTATTCGCCTCTTATAAGTTCAGCTAACAAAAATCTGGACCGGCGTTGGAGATTTAAGATGAGGGTATTCAGTTTCGCTACCAGTAGTAGAACTCATCGCACTACCCACTCGGGACTGTTTAGCTTGATCGACGCGCAAAATTTTATCTTTTGAAACTTGAGACTCTCTGGCGAACGGGGAACGTGTGCACTACTAGCTCCAAGTAGCTTGTGCGTAAAAATAAAATAAAAACCCAGAGAACCCTTATTCTCGCAAGTGGCCTTAGGCGAGTGGCTAGGCGGGGAACCAATCTGAGTACGATTGGGGCCGAATCTCGGGTGGCCTTTGCTTCTTTCGGCAAAAGGAGCGCACTTGTATTCTTGCTCTTTGAATTCGCCAGGATTATCTAGTGGCCAGCTCAACGCCTTACCTTAGGTACCTACCTAGGTGGGCAGGT

ATCAGCAAGAAATCTCACTATTAGCTACACTATCCACTCCGGATTCATGCCGGTATACGTAATGCAGAGAGCGGATTCTACTATTGGACAATGTTGTTGGTTGGTGATATGGCTTGCTGGTGAAAGCCCTCACCCCCACAGCGAACGGTTTCATATCACTTGCTGGTGGCTGAACCTACCTACCTAGGTAAGTAAGGTAAGTAAGGTAGGTAAGGTAGGTAAGGTAGGTAAGGTAGGTAAGGTAGGTAGGGTTTCGATCTTTTCGATCGCAGCAACGGTCCTTCAGGGTTGGAACGGGCT

TCGTAACAGACCAACCTCTCGAACACAAGGCTGGACTCTGCCTGCAAGACCAATTGGTGCCCTGTGCTGTTTTGGACCGCCTGGGCTTTTGAGAAATACCGTACCATGTCAGAGCCGAGACCCAGAGGTGGGCAGACAGACAGTCGGTCTGGGAGTCAAAACTGCAATACTGTCTGTGCTTTGGGGCATATGCTTGCCGCCCAGAACGCCTTGACCGTTAGCAAGCGATAATTGTAGCGCCAAAAAAAAAAAAAAAAAAGGCAATCGCCTTACAGTGCGCTTGTCAGCGCGAAGGGATGACTTATTACAGTTTACATGGCCATGGCTTACCGCTATCTCTGGACTGGGCTGGACTGAGCCTGAGCACATAAGGAAATTATATCTGACCATGTCCACAAACGTACCGGGGACCACAACATTGCTGCGGAAAAAATGCGGCTTAACTTCGATAAATGTTTTTACCAAGCAGAGAAATGCCAGAATAACAGACAGGCTCATGTGGCAATTGAGTCATGATCTATGTCAATCTACCGCGTATTTTCCGGTAATATTGAAACTGGGAAGATTTCACGGGCAAAGTGAGTGAGGTAACGACTCACAAAAGATTGAATAAATTAAACAAAGAGGCAGTGTGCGAGCAACAGCAAGGGAACCCACGCGACTCAGGGCAGATGCTACACTGGATACTAAGCCCAGCAGAGAGACAATTCTTACTTGGTACTGCCGGATCCAGGGCCTGCCCTGCTCGCCGGTCGCCGGTCGCCGACTCAGACTGACATTTGACGTACCCTTTTTTTAGTCTACCAAAAAAACTTTGAGCACCTACCTTAACTTACCTTACTTCCATAGCGAAGGTACTCGCTCCCATAAGGGCTGGGTCCCCCGCCTCGCTTAGGCTCGTGCAGGTGCGGTGCGGTGCAGCGCAGCCAGGCGCTGCAATGGGTTGGGTTCCGGGAGGGTTTTTAAACCGACTGCAATGTGTACAGTACAGTACTGTACAGTGACGCTCTCGGGGGCTTCTGGCTGGCTGTTGTGACACGGTTGGCGGCAAGTACGGCTAACTGCCTTTCTTGAGATAGATACCTAGGTAGGTAGGTACCTACGCGCGTGCCCACACACACAGACCAGACACATGCATCACGCAACGTACCCTTCCTCTACAAGCAAAGATCGTTGATTCACCCTCCGCATTCAGCTAGAGCCCTACCCGCTTGAGGCCCGATTGAGACAATTGTCGCGGCCATCCCCCCACTCTGGTGCGCCAGACCCCTTTACAGTAGGTAGCTTCGCACTTCTCTCTGAGATTGCCATGCCGCAAGGTGTTGTTGGTCCTATTCGAGAAGCCAAAAAAAAAAAAAGCCTTGCATGAGAGACAAGACTCCGCCTTGCTATTCTGAATATTTCCAAAGTACTGTGCGTGCCGCATTTGTTCAAATGTAGGAAAGTACTGAGTTTTACCAAACTCGTGTGCCCACCCCCTTCCCATCCATGGTGTCGGCTGAACAGGCCGCGATTCCAGAGCTTTGAATTCACCCGGTTCCAATGGCTGAATCCATAGGTCGAGACCAGATCACGGACTCACCATTGTCTACCAAGGAATGCCAGGAAATCCTTCATCTACTCCACAAAAGTAATATATTCTCACCGGTGCTTCGCTTACTGTTCGGGTCCGGATCTCTGGGAATTTTGCATTGGCAACAACAAATGACCTCTAGCTGTTTGGCGTCACATGAGAAGACACAATTAATTACCCAAGGCACTCGCTCGCTCTCCAACCACAGCCTCCCATATTGCTTCAAAACTAGTCGATGTATTTTATCTGCTTCATTGGTGGGTAAAGAGGCTGGCAGTCAAAACGCGCACCAAGGCGGGTGTAGAGCTTCCTTTTCGTGGACATGGA

**> MD406_chr04:3804291-3808291**

GCCTCATATTACAAGGGACTAGAAGTGCTGTCGTAAGGCGGGGAAGCGTTCTGTGGTTGTAGATTCGAGCCCGAAAGAGTGGGATTTGGATCCATTCCAAAGTCGTCAAGCTCCACGAACATAGGCGGCCTCCGGCTTTTTAATGCAGCAAAGAAACAACTCCTGGGGTAAGGTGAGTTACGGACACCTCTAGAACTACTGTACATAGTATTAGACTAGTCTAGTCGTTTTGATCCGGCGGGATCCTTTTTTTTTCGTCCGTTTTTATTCCTGCGGCCCTTGTCCGTCTCAAACAATCCCTGTTCCACAAAGATTAAGAACGGAGTAAGGCACCTACTGCAGAAAGAGTACCTAGGTAACCTTACCTACCTAGGTAGGTAGGTAAGGTAGGTGTGGTAGGTGAGTTAGCTACCAATCCCACCTAAGCAAGCTAGGTAATGGTAAGCTTGGTCAAAGGTGTGGATTTTAGTCAGCAAGCCACAATCTTGGTTCTTGTCCCAGATGCAGTGCTTCCAAGTCAATCCTTCCAAGCCTTGATGACGTTGAGGGTCTCGCACACGCCCAGATTAGACTCCCGGTCTGGGGATCAATTGAGGCTGGCGAGGGTTCATGTTTGTTTGAAGTAATGACCGACTCGTACTCCGTACTACCCTTCCCCCTTCAGCATCGACCGCATGTTACCACGTACTGGCCCCTACCTTGGTTCCTCATCTCCACTTACTTGCCCACCTACGAAGTAGGCATAGGACTAGGTAATAGGCTAAGCTACAATGAGAGATGTATTGGAGTCCCTACATACCGTAGAAGTCAGACTGATTATGTGGACTATCTTATTACCTACTATTTTCATACCGGTCGGTACCTCCAAGGGTAAGGTAGGTATTATGTACACTAACTAGCGCCGCCAGCACCTTTTTTTTTTTTTTTTTGCCAGTCAACAGCCTGCCCTACAAGACTTGACGCGTAACCCAGCCCACCACGCCAAGCAAGCACATGTCCACCCGCACTTCTGTTTTTGATGACAAAGTTGGAAAAGTGATCGCACACCTAACTATGCAAGTTCTCTTTGAAACAAGTCACTAGATACGTTATGGACCGTATGCGCAGTTGAATCCCGTGCTAGTACATTGTAGAGCACACATGCGAACGTGAGGATCTCTGGGTCCCCCTAAGAGAGGTAGTAATTTACGACTGCACCCGAAGATGATGTATTTGATTTGTGTATTAACAAGTTGCTCTTTTTTTGTGTGCCGCTGCCGATGCCAAGCAGCTTATTGCTATTGGAAGAGATAGTGAGTCCGGGGGCCTCCTTTCTTTCTCTGTGTGTGTGTGTGTGTTTTTTTGTATCTCTTCTCTTAGCACAGCTAATTTCTCTTGATGCGACATTTGCCTCCTACGAGTTATATACCTCATCCATTGGCTGATGATTCCGAGAGACGTGCAGTTGGAGAATTAAACTATCAGAACAGGCATGAAGTCCGTAGGAATCGTGACTAAGTTCATTACATAACATGCTTGGTATATGAGTAAATATATGGCAAAGTGTGACAAGCGAAAAGATAAATAAAGAAAGAGAGGGAAAGGAAACAAAGAAAAGGTTGGGTGGCTCTGTTCCATACACCGGGATTCAATCGAGGGGTTTCCGGGTCTGGTTGCGGTGACGCCAGCGGTGACTTCAGGGAGCCTCCCACCCTTAAAAGTCGGAATTCCCATTTATCACAGCAGGAATTTAGCACTCATCTCCGCAGCACCATAAAAAATTAAGAGAGAGAAAAACGACTCGCACACAAACCTGGACCA

AGCTAGAGAAGAGCTCCCGCCTCTCTCCTCCGGTCCGGCAGGTGGCCCGAGTCTTTGATGTCTCGCCCTAGAGCCAGCACCTGCTATTGTGTCGCACCAAAGGGTTAGAGATTGTCTTTTTTTTCTTTCCAATTATTTCCTTGTTTGCCCTGCAAATACAGGTTCTTTTGCAGCCCTTGAGATACCAAACATAAAGAAATAGAGAGAGAGAGAGAGAGAGAGAGA

GAGAGAGAGAGAGAGTTTAAGAGGGAAAATTAGAAAGAAGAAGACATAAACAAAGACGGAGCCTCGTTTTTTTTTTTTTTTTTTTTTTTTGCCCCGTTTCTCCCTCATCCCGTCGATTCACACAAAGAGTCGCGCGAGTCTTTGCAGCCACCCTCGATTCGCTCTCAAGCCAAGGTTAGGCCAGACCGCACATCAGCCAATTTACTTTACTCAGTTGCACTGCACACTACGTTTGCGACTGGACCGCACACCACCCCGCCGCCACTACCTAAAAAACTCCCGCCGACAACCCCCGACTCGACGACTTCCTGGCTATTGAGATTGCAACTGCCCTATTAATAATTACAGCAAACCTACCAACAGGGTTTCCTCTTGTGTCCTTTTTTGGTTCCTCTCTTTTATTTTTCCCCCCTTGGCTCTTTTTATTTTATTTATCCCCTCCTTTTTCCTTGGTCTTAATTACTTTTTTTTTTCTTTTTCTACTATTACAATCCCAACTTTGCTACTGTTATTTTTCTTCTTCTGGTCTGACCCCTACCAATTCCCTGCTCGCCCGCCATGGGGAGCACGTCGGAAGCAAGGACGTCATCTTCCGCCGGCGCATCGCAACCCTCGTCCATAAGATCAGTCGACGTCTCCGCAAAATCGTCACCAACCCTGGCGCCTCAAAATGGACACCACAACGGCGTCCCGAATTCGGCTGAACAGGTTTTAAAGCCCGTCGAGAAGACTTCGGTCAAGGAGCGCCTCACTCGCATGTTTTCGACCAAGGACACGCAACGGTCCTCGAATCTCGCTCCTCCCTCGGGCGATAAGTCCGCCCCTGTCAGCCCCGGTCTCGCTCCACCGGTAAGGAATCAACCTCCGAGTCGCAAGGGTTCCACCACCGACAAGGCATCTGAAAAGACGGCCGCCGCCAGCGCCGCCAAGGCCGCCAAGTCGAGCAATGGCACCGCACACCAGCAGCAACGATTTCTCGTCAACCCTGACGCACAGGGTGGCCATGAGCATCATCTCAAGAGTAGCCGGCGCCAGGAGAAGCTGTCAGACATGTTCCGCAGCATGCTGGGCGGCGGCAAGCGCGCCGAGCAGCAAGATCACGATCTCTCGCTCGTTTCCGGATGGGTAGACACGCTGAAGCAAGAGAAGGACAAGGACAAGGATGTCGGTCAAGCCGGCGAGAAGCGCGGAGGTCCCAACGCATCCGTGACCTTGGTGGAGAAGTACGGCAAATGCCAGGAGGTTGTGGGTCGCGGAGCGTTCGGAATCGTGAGGATATCTCACAAGAAGATGGACAACGGTGCCGGTGAAAGGTTGTTTGCCGTTAAAGAATTTCGCCGTAGGCCCGAGGAGAACGAAAAGAAGTACAGCAAGCGACTGACGGCGGAGTTTTGTATCTCGTCCAGCCTGCGCCACCCCAACGTCATCCACACGTTAGACTTGCTGCAGGATGCCAAAGGAGACTATTGTGAAGTCATGGAGTTTTGCCCTGGCGGCGATCTGTACACACTCGTGCTTGCCGCCGGGAAGCTTGAGGTCCAGGAGGCGGACTGTTTCTTCAAGCAGATGATGCGTGGCGTCGAGTACATGCACGAAATGGGCGTGGCGCACCGTGATCTAAAGCCTGAGAACTTGCTTCTGACGACCCACGGCGCCCTCAAGATTACGGATTTTGGCAACGGCGAGTGTTTCCGCATGGCCTGGGAGAACGATGCGCACATGGTTTCGGGCCTGTGCGGCTCCGGTCCTTATATCGCTCCCGAAGAGTACACAAGCAAGGAGTTCGACGCCAGGGCTGTCGATGTGTGGGCCTGTGGTGTCATCTATATGGCCATGAGAACTGGCCGCCACCTCTGGCGAATTGCCAAGAAAGATGAGGATGAGTTTTACGCCCGTTACCTCGAGGGACGTCGAGATGAGGAGGGCTATGGGCCGATAGAGTCATTACACAGGGTGAGTTTTGGGCTCTAATAAT

**> MD408_chr04:4465053-4469053**

ACTATTTGTATTTCAACCAGTGAGCGTGCTTTAACCGCAGTCTGACTTTTTCCCTTTGGCGCTGGGCAAACTATGAAAGCCTTCCGACCAATACCGAGGATCGGGAAAAATAAAAATAAAAAGAAACTCTAAAAGCAAAATTCAACTTCTTCGTCGCCACACCCATGAGCCATGAGATGAACAATGGTGGCTGCAGATATCAGATCGCCCTACCTATTTATTTTGCCGACAAGATTCTACGCATATACGAGGTTCAGAAACAAGTGTTAAAAACATGTATTTTCCAACACTCTTTAAACACTGGTTTACACCTTTATACTTCCTTGTTGAAATAATACGAGCATATGTACTTGGCTAGATATGTTTGTAAACGATAGATGGCCCATCCAGACCAGGGTTCAAAGACGGATAAGTCATTGCGGGGTTGTTTGCACAAGGAACGGCTAGTCCGGGACCGGGATCCATGTTGCATCATGCACTAGAGGGAAGAGAGTCAGCTATATAAAGAGCATTTCTATTAACTTGTCATACGCCCCGTCATTTGAAAATATGCCATTTTGAGCATTCAGCAACACTACAATTAGTCCTATACTTAATACTCCTATAAAGTTCTACCAACATCACCATCATGAATATTAAAATCATTTCGATCGTGGTCAGCTTGCTTGCCATCCAAGCCAAGGCCACCTACTACAGAGTCTCCATCGTCAAGGAACCCAACGTCAACCCAACCGTCAAGAACCCAGTCGCCTCGATCGACGGCGAGGAGGGTAAAGACTACGTCATGGTGTACGAAGGCCAAGCCTTTCGCGGGAGGGAACCGCCCAGCGTGGTTGTTCCCATCAGGATCACCGGAGGCGAGGCCATTCCCGGCGGCGGAGGTCTAGGGCGTGGGTTCAAGCTCAGAACCCAGCCCATATCGGCAGAAGAAGCGGTGCTTGCTGAGCCGGTGAACACCGAGCCGCACCCGCCTCTGAACAAGGAGCAGTACGCTCAACGGACGAGTGAGCAAGGGACAGACGGGCCTGCTCGGAGGAAGTGGTGGTAGGTGACAAACTCTACAGCAGTTCTGGAGAGGCAATCCTTAACCTGCAGCATGGGAGAAGGGCAGGTTAGCTGTTGGAATCCTTACTGGGTTGATTAGTTAACGGCGCTTAATTGTACGATGCTGCGATATGGAGATAAAAATAGTAGATTTTCACCAACTCAAGGATAACACAGTTTAATAAGAGTCAGGCGGCGCCGTCGGCCTTTCCGTAATATCAAGACTCGCATTTTTAGCGTCCTATTATAACTTACTCGTCTTTTTCTTTTCTTTTTTTTTAGAGCATCTTTGCAGTATAATGTCGTGCTAGAGGTGAAAAGCTGAGGGAATTGCTATTTCTGTGTTTTCCAGGGGTAGTAAAAGCTGTTAAACTTTTCTAATTTTTACATTGCCAACTATTTTACAAAGTCACATCAACCGTACAAATCTAGGCAATCTTCCATGTAATCAATCGCTTGTTTTTGCACTTTTCTTTTCAACTCTTCGTTTCGACTCTTCTCTTTCCGCGTAAGGATTGGGGAGTGACCAACAGATATTATTATCCAATCGTAATCCAAGACGGCAAAGCCCTGAGGTGGTGGTCCTCATCAGAGGCTGCCGGTCCCCCCGACCCCCCTTATAGGCTCGAAACTGAGCCCATACCGGAGGCACTAGCAGAGGATCTCGCCCCGACAATGCGCCATGGTCCCCCGTCGCCGGTAGCGAGGCCAAAGAAAAAGGCTGTTTGGAAACCGTCTTCCTGGTTGTGATTTTCG

TAAAAGGGCTGGGGAAAGGCAGTGGTTCGGTTTGAGAGAGAAGAGACTGACTCTCGGGATGCAGTGGGGGATTTGCCTGGTTGGGTTTGATATTCTTGGATGAAAACAGCTCGGCGTTTAGCAAGGGGATTTCTTGTTTTCTATCATGAGGTGTACGTGATTACGAATATATTTCTAGTCTAGTGGATAAATGCAGTCAAACACACACACACACACACACACACACACACACACACACACACACACAATTGGATAGACTATGAAACACATAAATGAAACTTCCTAGCAAATAGCGAAAAA

AAGACGCCGCTCACTAAACTCAATTAACCTCAACCGGGACCGAGAGCGTGTACTCAGTCTGACCCTCCAGCACCCTGGTGGTATCCTTGTACTTGAAAACGCCACTCGTCCCAGCAGGCGCGCGAATCACCACATTTACGACCTCCTCCCCCGACGCACCCGCCCCGATTTTCCAGTCCACAGCCAGGGGCCCCTTTGGCGTCTGGACCTGCGCGCTCGCCCGCTTCAGCCCGAAGCTCCCGACGCCGACCTCGGGGTCCACCACCCAGCTGGCGTAGCCAAAGCCGGCAACGCCCTCGGCCTGCCGGACGCCGGCCGCGTGGCGCGTCAGGGCGTAGGTCGCGGCGCCGCCCCAGGGGTGCGCGAGGCTGGTGAAGAGGCCGAGCCCCGGCTCGCCGGTCGCGGACAGGTACTCCCAGCTGGCGCCCGAGGCGTGGCGGTCGCCGTGCGCGCCGGGCAGCATCTCGGGCCAGAGGGAGCGGAGGAGCCGGGCGGCGGCGTCGCTTGCGTTGCGGCTGCTGCTGCTGCTGCTGCGGCGGAGCAGCCCGTCGAGGAGGAAGCCGTTTGTGTTGGGCGAGATGGTGGTGTCCGGGGCGGTCGAGTCGATCAGCGACGAGTCCTTGTACCCCGGCCCGAGCTGGAGGGCGTCGAGGGCCGCGACGGAGCGCGAGATGCGCCGCGGGTCGGACGCGACGCCCGACGACAGGCAAAAGGCGGTGGCCTGCACCGAGAGGTGGTCGCGGTCGGCCAGCGAGACCGAGTAGAAGCCGCGGGCGTCGTTCCAGAGCCGCGCGTTGACGGCGCGGGCCATCTCGTCGGCGGTCTGGTTGGCCCGGGAGTGGTAGGGGTCGTCGCCGACGGCCAGGGACAGGTCGGCGATGGCGCGGAGGGTCTGCACGGCCAGGCAGGACGTGGCGGAGCCGTCGACGGCGGGGCCGAGGAAGGCGCTTTCGAGATTGAGCAGGTTGGTGGTCTGGTTGACGCGCGCAAACAGCCAGTCGAACTGCCGGCGCCACTGGGCCCAGCCGTCGCGCGCAAAGGCCACGTCGCGCGTCTGCTGGACGTAGCTGTGCAGGGCGTTGAGGCCGAGCAGCTGGTAGTCCTCGAGCGCGTAGGGCTGGGCGCCGAAGCCGCTAAAGGGCCCGCTGGCGGCGGCGGGGTCGTAGCCCATCCGCGGGGAGATGTCAAAGATGCCGAGCGGGATCTGCGTGCCCAGGAAGAAGCGCAGCGTGCCGCGCTCCAGGTCGAAGCGCGACGTGCTGGCGCCCATGATGCGGATGGTGTGGAGAAAATCCCCCAGCCAGACCAGCCGGTCGCGCTTGGGTCCGTCCAGGCACACGCCCTCCGAGTTGCTCTGCACGCCGTACTCGGACAGGACCTCCGGCGACGTCATGGCGTTTTGGTAGACGACGCTGCCGTTCTGGGCCGTCGCCGTCACGTTGCGCACCCACGCCGCTTGGTCCTGCCACGCCCCGAACCCGAAGCTGCCAGACGGCGCGATGGGGCTGCCGCCAACGTAGTATTGCGAGAGCTGCGTGTGCAGGATCCGTGTCTTGTCGAGGGATACCGACAGGAAGCCGTCGGGCGAGAGGGTGGTTGTCAAGTGGTACCATTTGTTCTCGGCCACATCAAAGGGCACAGCGGCGAGGGCCAGATCGTACGAGGTGAGGGTGGTCTGGTTGACAAAACCAAAACCCCATCCCAGCTTGAGCGTGTTTCTCGGCAACAAGGTGGTGTTTGTGTTGGAAAACGTCGTGGCGTTGGGGAGCTCGCTCGTCAGCTGGAGCTGCACGCCGCCGAAGCGACCAAGCGGCCAGGCCACCGACCACCACACCCCGCCGCGCTCGATCATGGCGTCGAACTCGAGCGTGTAGTTGCCCAGGAGGGCACCCCGGAGCGT

**> MD409_chr04:4706378-4710378**

TTTCATACCTTTCAGAACTATTCCTGCAGCTTTAATATGCTTGTAAGAGAAATATATAGCTTCCCATGACCCTATCACAAAGAGAACAGGTATGATTAGCTGCATAGTAGCCCAAAACAATTATCCGTTGAGGTTGATTACCTTTATAAATGTAAAAGCTTTTCGAGCTGCTCAGCGAATTTGAATCGGGAGCGGGTGAAGCAATCCACTACAGATTGCTGCTTCTCAACAGGTAGACTATTTGTAATGTTGCTCAAGTCATTGTAGTGTTCTCGAGTAGATAAATAATATCCTTGGCACCTTGCTCGTGCTTAGTCCGTTTTGGATACGCCAGGGCAAAGACTTTTGATCTGAAACGAATGCCAGGTTGCAGGAAAGGAAGAGAAAGCATTGCGTTGGAAAATGTTGTGCAAATAAATCGGGTAACGGTTGCCAATGGTTTAATCGAGTTTTTTGGCCTATAAATAACGTTAACCACGTTGCGATATTTTATTGGCAATTTGGAAAACCTACCGCCTATCAGAAAATCAATATCAAAATATTGATTTTGTCGGAATATCATTTATGCGCTATCCTGACTAAGATAATTACGGGGTAAAAAGCCTGTATACATATAGGCTTTTGGACCTGTAACGGACTTACTTCTGTACCTACTTCCTTCGTTAATCAATTTTATACCTACATACGTATCCTGTTGGTTCACCACTCTGTTTTGGCCATGGCCTTAACCAATCCAATCAACAGTGGGACAAATTTGGGTGGCATTTGGCTGGAGATAAGTCCAAAACGTGCTTTTGGAATGTTGTTACTGGGTGAGGAACGGAGTGGATGTTAGTAAGTGCCCGTAGTCGGATGTAAGTGCTCGTTAGTCTAGCCCTTGAACATCTGCATGATTTTATAGTTGCTAGCTATCCTTGTGGGCATAACGAGCGATAGACGCCGATAATATATGATGATTTACCAACTTCAACCTCATGAGCTACAATGGTATTGCACAAAGCCGGTTAAATTTTATGTCAGGACAAAGAAACAAACAACGGCCAATATACATTTTGACAAACCTTTCTTTGACAGCCGCTTCTCTGTACATTCCCAGCCAAAGTCCGCGAAGAGACACTAACAAATGGCAAGCCAAGAATCTAAATCATCGCTAGATAAGAAAAGAAGTAAGGAAAGACGAGGAAGATACGAGTAGGAAAGATTGACAGTAACAAAGGAAAGGAAAGACAATACAAACGCCGGGGCTACTTAGTTCCCTTCTTCACGTTTTCTCAAAGTACACTCTCAGTGTCAACGCAGCAAACCCGCTCCAGAGCCTGCAGTCCGTTTAACTTAGGCTCTGTTGGGATTGATCCTGCAGGACTTCCCCTTGTCTTGAGGGTCATGTTACTTGGGCTCGGTGCCTACCTTGAGGTTCCAATGGGCGGCGTGCTTGAGGAAAGAGCCGTAATGGTTATACACTCGATTATCGCGTGGTAGAGGGGCCGCATCTTCTGTCCAGGTGCTTTTCTGCTTCTTGGTCTTCCTAGGCGACTTGGAGCTTTCTGTTACGGTGAAAGCGGTGCCCCTTGGCGGTTTTGTAAATGGGATTACCATCGAGGTCGACGCCTCCCCTGCCTGAATCAGGGTCGTCGTCACCACGGGCCACAAGCCTGGTGCTACGTCCGTGGCGGGCTTTATAAATGGCATTACCCTCGGCGTCGACGCCCAGCTTGCCCGAACGCGGGTAGTCACCCCGGGCGGTGATCTGGCCCTGCGGCGCCGAGTTCGACTGCCTCGGAAAGCCCTCATGTTCGCCAATACCCGGCATCATGTGGTTTGACCATCGGTTCTGGGGTAGCCCCTTTCCCTTTCTCCACTGCGGTGATCTTCCCT

TTTCATCATGGTCGTTTTCGGCGGACGCGGGAAGTTTCTCGCGGCAATCTTGGGACCGTTTGGAGCGGACCTTGGATGAAGAAGAAGAAGAGGAAGAAGAAGAAGAAGATGATGATGATGATGATGCCGAAGAAGAAGAAGAAGAAGAAGAAGAAGAAGAAGAAGACTTTGAGCCGTCGATCAGTTTCTTGAGTATGGATGGAGGAATTCGAGGAGGGTAGCTGC

CCAGCCTGCCCTTGTCGTCGGGGCAGGGGGCGGGTTCGCCCAGGTCATAGGGCAGTTCACGGGACACGAGACCGGCCTTGTCGGCGCCATGGTCGGCCAGAACTTCGGCCAGCGCCTGCGGGCCTAGGGCCGCAAGAACGGTAAACTTGAACCAGCTGGAGACGCGCATCTTAAAGTTCAAAAGCAATACGAAGAAACAAGAAACATATGAAAAGGCCGAAAGTGTACTGGCGTAAAAATCAAATAAGCAGAAGTTATAAGCAGAACAAAGGGTATTAATAGTTTATAGTTAATACGACTGCGTATGCATTATTCGTGAATAGTCCATAAAGGGCTATGTCCCTATAAAAGGGTTTCCTATTGTTAACTAACCTATTTTTGTAACCTTTACCAAGCAAATTAACAAAACATGGGAAATGCAAGCCTATTGCTTCGGTGGTTCACCGGCCCGGTTATGGGCCTATGGACACCTTGTCTTATGACTAACAATATTTAAAAGTAAATTAAAATTTCTTGCTCTTTCCAGGGTAAATACTGCAAGGTATTATTTTTGGCAACACGTACTAAAAACAAACCAGGGCATTACTCACAAATACATGACTGGCATGGTATACTATTAAAATAAATTTATGATAGAAGCCAAAATAAAGAGTGCACAATTAGAAAGAAATTCCAAGAAATATCATACAAAAGCTACTTTAGTGTGACATTCTATCCAGCCCGTTTTCAATATTGCTGTGGCCAGGGTAAACATAGCACGGAAGTCGGTTTATTAGCAACTATCGACCAAGATTTTACTCTTAGAAAATGAAAGGAAACAAACAAAGGTCCAAGCGTGACGCGACGTGGATGACTGTATCACTTCGTGGGGCTTTGGTCAGGGGTCAAGGTTAGGGTCTGGTAATCTCAGGGTCCGAATATTACACTTTTGGCTAGTTACATAGAACCATTTCACTGCAGAGGACGTCAAATGAGAAATTCAATACGCAAGTGCGATCAAAATGAAAAGCAATATCCTCACGCCACCTCAGGTTTGGAATGACAACTCGGAAAGTAAAACGCCGGCTGACTCGATGACCAGCGCCCACCGAGAAACGGTGGGACTTTTGGAAGAATCTAACATTCTACTTACAGGAATGCGCTGGCGGAACTCGTGTGAAATATTTCCCAGGTCTTTAGATTTGACATTTCAAAACAGCAGCTACATGTCCAATACTCTACTATGGTGGTAAGGGTAAAAATCAATGCCCCTGTACATTGTCCGCTGTTGGGGGCTTTGTGGGCTTTGCCGACTTTGGTAATAGTTTTGAACAAAAAGAATAATTTGGGTACTTGATGGTTCTCGCGAACGATTAATTTAATTAAGGCCCACGCATATATATACGGCGATAAGGTGTAGTTTTTTTTCGCCTGATACCGTGGTAGCAACGAGCAACAATAACCCCGGACTACTTGATTAGGCTTAACCTTGCTATAACAACTCCGAGAAACAGCTGGCGAAGGATTCTTTGTCATTTGTAAAGTCGTTTCGCTTCAGTCATATCCACCCAGTACTCACCGTGAGACAGCGAAGCTAGCCTTTGCGCGGATTGGTTGGGGTGCCGCGATTCTTCCCGCCCAGGTCGAAGACCTTCTTGGATCTGAGCTCAGATCTTGATTGCAACCCCTTTGAATCTTGTCGGCATAGGTGAAATCCACCGGGATCTGTTGTTTAAATACGCCGATGTGCATATAGACATCGTCCCTGTCAGTCAAAGAGGTCAACTGAGTATTTCTCAGTCTTTGAAGTTTAATCATGTTGTCTTTGAACTTTTCCATAGAGAGCTTTACGGTTCACCCGGCTGCACGATCTTTGACTGCTTGATCACATTTTACAGGTCATGGAAGCCCATAAAGCAAA

**> MD410_chr04:4836239-4840239**

CTCCGACCCCCCTAAAGTCCGGCCCCCTTGAAAAATGTAACGACGAAATACCCCTTTTATAAACCGATTTAAATGTAAACCTATCAACGCACAATAATACAATCATTGTCAGGCTCTCCCGGTTCGCTAACCTCTTCTCCATCGTTCAAAGCTTCTAAACAGTCCCTATTATTTTCCTGTGCTTCATAAACGTTTTTTTTGCTGACCAAAAGCTCGTTGGGATCCGGAATCACCCTTTTCCTTTTGACCGGCCGTACCGCCTCCAATTTTGCTTTTAACAAACTGATTTTTTGCTGAGCTTCAGCCAATAAGATATCCTTGGTTTCGAAGCTTTTTTGGACTTTTGCAAACAAAAGCCGTGAAGTGGCGTTACTTTTTTCGGACCGGGAAATTTCCTGTAGTTGAAGTCGAATATCTCGGGTCGTTTTAGGGGTTTTCCAAATAAGTAAAGACTGGTCGTTAATTTTTTGGGTTGGATTTTCCGGTGTTTTATCCCTTTGGAAGCCGTTATTTTTACCTTTTTCGACGTTGGCGTTGCTATTTTCTAATAAAAAAGGGTTTAAAAGTGGTTTTGCCAAGTTCACCGGCCATAACCCCGTCGTACGCCAACCAGATTGAATGGTTTTTGCTATAAATGCTTTTGATCTGGCTTTTCGATAGCAAAGTAGAAAGTTTCGTTTCCCAACAACCGTTGAACAGCAAAACTGGTTTACAAATCCCAGGTGACGTCGATAAGCTTCCTTTAACGGCCCAAAAACCGATAAATCCAACGGTTGAAGAACGTGTGACGAATGAGGGGGTAAATATAGGAGTTGAATATTGTTTTGCAAGCAAAGAAGCATAAATTCGTCCGTTATATGTGATCCATGGCCATCCAGAACTAATAACCGCTTTTCAGGGGTTAAAGGTTGGGTATACGGAATAAACACCTTTTTTAACCATTCGATACCTGTTTCATTATTTGTCCACCCGTTTTCGGTTGCATGAAATTGCCAGGTATCGAAAGGACTTAAATCAGCTGGAAACCATTGTTGCTGTACGTTTTTCCCCTTAAATATAACGAGGGGAGGTATAACAGCCCCCGTAGCTGATACACATTCGATTATGCTCGTCCAACCCCGCGTTCCAGGCTCTTTTCGCTGTAATGGCCGGATTTTATTACGCCCTAACACCAGGCCATTAGATCCTTTGCCTTCCATTATACCTGTTTCGTCCATATTCCAACGGTTGGCCGGTTTTATAGTATCGACAACGGGATTTTTCAAATAGGACCACCAAGATTTAATTACCTCGGTTGTAGCCCCATTAACCCGGGCATTTTCTATTCGCCGGGGTCTTTGGGTTTTCAAAATTGGATATCGGGCTATAAAACGGCTAACCCAATGTTTCCCAAGGCTTTTTCTTTCTCCGGCGGCCTGAAGAATTCGTTCCGCAAAATAGCGTAGTTCTTGATGCGTTGGCGGAAGGCCTAACGCGGCCTGCGCAAGTACCCAATCTGCCAAATAGGTTTCCTGCTCCTGTGAAAGCCTTTGACAAAATCTTTTCGCTTGTTGAATTGACTGGGCCCCCTTTAAACGATCGTGAAGGGTACTCCGAGGAATACCCCATTTTTGCGAGGTTTTGTAAACTGATTTGCCATTTCTTATGTCAGAAATGGCAGCAAGAAGCTGATTTTCAGTGTACTGTTTCATTGGAAGGTTTGGAGCAAGAATCTTCAAAAATCAAGTTCTAAAGTGAAAAATTCGTCTAGATATTTATAAAATAAAACAAAGGGTTGACGTGGCTGAGTAGATATTTTTAGGGGCCGGACTTTAGGGGGGTCGGAGATGTGGAGCTCAACGTTATCGCTTCTTTTTCTCCATACTTGAGGAGAAA

AGAAAACTTTTTGGACACGGAAAGACAAAAATGGAACAACTTTTGCAACCTTCTTAAGGCGCCAAGGTGGGGGCACTGTTTATTCTCTCCTTTTCCTTTTTCCTCCGGCGATTACCTGTTAAGGCTATATATATATATATATATATATATATATAATATAAACTTTCAAAATTTTACACCCGTTTGACTTTGACGCAATCGTTCAAATTGGAATTTGTTTACTTTGTCAACGCAGTTGCACACCCGCTTTACAGACTAGCTGTGGACACGAAGAATGTTAAAGGTAACCTATTTATAACA

TAAAAATAAAGATGAGCAATCCCACTCTCCCCCAACGTTTGAAAATAAACCGGAAGATAGTATCCATAACGACAAGTGAATGACCGTTCCCGCCAGGAGGATCAAAAATTGCTGCTGACAGGTGGGGGAGTTCGACAAAGCCAGAGTGGAGACAAACCCGGCGATAAAATCCAACGATGGACACCTGTGGACGATAACAAAATTCTTCTGAGATATACTTGCTCGATTGACTGCTTGGTCCGTTAGGACAAGAATTCCCCCCCAAAACGACACATAAATCACTTAGATACATTGCGAGTACCATTGGTTTGATACTGTGCAAGTTCCCTCGGAGCAGCACTTGGGTCCCGACCATTCCTGGCCGCCACATTGTCCCCATCGCGGAGAGCAGCCAGGGTTAGGAGTACCACCACCGCCGCCGCCGCCGCCACCACCACCACCACCACCACCACCATCGCCGCCGCCGCCACCTCCACCGCCTCCGCCTCCACCACCGACCGCAGCATTGAAGAAGCTCCAAGTCTCACCGGGCGCAAACTTCTGACCACCGGTGCCGCTGGGGTCTGGGACGTGACCACCGCCGTGGCCGATCCAAGTGACGGCGGCGCGGGAGCAGGTGTATGTGGTCTTGATGTGCTGCTGTGCCCCGGCCCCCGGGTCGGGAGCAGATTTGGACTGGCATCCGTTGGTGGTCAGCCACTTGTCGCGCAGCTGGCGGCCGGATCCGATGCCGAGGACGTTGTCGGCCGCGCCGTGGATGCCGAGGTAGGCGACGGGGCTGTTGCCGCCCGAGCAGCCGGAGAGGAGGGCGCCGGACAGGACGGACACGGCCTTGAAGACGTCTATAGTTTTTTGGGGGTGAGTGGTTGTTAGCTTTGAGAGTAAAAAATGGGGAGGACGGAACAGTGCAAAGAAGGCGAGGGTCGTCGTGGTGAAAGGGAGACATACTCGGCCGCGAGCAAGCGACGCTGTGGCTCATGGCACCACCATAACTCCAACCGGTGGCAAAGACCTCGGCGTCGTTGATGCACAAGTCGTTCTTGACGCTGTTGAGGATGGCATCGACAAAGGCAATGTCCTCGCCGCCGCTGTTGGCCCAGCCCTTGTTGAGCCCGTTGGGGGCGACAAAGATGGCCGTGTTGCCTGCCAGGGGCCTCAGCCCGTAGAACTGCCCGTTGGCCACGTCGTTCATGGACCCGTCGCGCCAGTGGAAGCCAAACACGAGCCTGTGCGCCGTCTGCGTGTTGTAGCTCGACGGCAGCTGCAGGATGTACTGTCGCTGGCGCCCGTTGACCGTGATGGTCTTTGTCCCCGAGGAGGGAGCGGCTTTCCCACAGCCTGCCGTCGGTGCGGCCAGGCAGGTCTGCACCGCGCCCAACAGGGCGAGACCTGTGGTTAGGAAGGAGAGCATCTTGGGGCCTGTCTTGGTCTGATTCCGGTGGAAGTTGCAGAGAAGCAGATGACAGCCTCAAATGCGAACAGCAATGACAGTCCCAAGGGGCTCTTCCTCTTATACAAAGGCAAAGGCCATTTTTCTTTTCTTCGTATTTTCCGGCTTGACATGGAGGTCTCCGTTCCGGCTCCGCAAAAGACGCACCTATCAGCACCGCCCATCTCCTGGCCTCCCTTGGGTTGCTTCTCTTTTGCAGTGGGTGGAGCAGCATTCCGACGGGAAGCCCCCCGGGGGCAGCGGAAATTAAAAGGCACATGGTGTGACAGGACGTCAAAAATACACGAAAAAGAAATGCCAAGAGCCGCAAGTCAAAGACCCCTGTTTACCCCGGATCAACAAGTGTCCAACGTCAGCATGTCGGCAAATGTTGGA

**> MD411_chr04:5098008-5102008**

GGTATGTTTTTTTTTTCTTTTTTTTTTTGTTCAGGTTCAGGTCTCCTACACGATCTTTTGCTACCCACCATTAACCAACCAAAACTAACCCTCTATAATCCCAACCAAAGGTCCGTCCTCCTCACCCCGTTCGCCCCATTCTTTGTCATCTTTTGCCAAACCATCTCGACCTCGTCGTCAGCCGACCTGGCGCTCATCCAAGCCTTTGCGGCGTCCCTGATCCCGGCCAGCGAGTACTCGGAGCCCGTCAAGCGTCTGCAGCGGCTGTGTGACGTCTTTTGCAAGGTCGCGGCCGTCTACCTCGAAGCCAAGGCCCGGGCCAGCCAGGTCGGCGACCAGGACATGATGCAGATCGGTAACGAGTTCGACATGTACTTTGGGGCACTGGGCTTGATGCAGCACCAACAGCAGCCCATGGTTCAACCGCAGTGGAATGGTTCACATCACCACCAGCAACAACAACAGCAGCAGCAGCAACACCATCAGCAGCAACAGCAGCAACAACAGTTTGCCCCGGTTCAACACCACCAGCAACATCCCCAACATCAGCAACAAGACGTTCAGAATATGGGAATGATGATGCCGGGCTTTGATATGGGATTAGGGGATTGGTATGCAGGAAGCAGGAGTATTATGGGCTTGTTGGAGGGAGACATGTCTACGTTTGGGATGCCGCCTGAGGCTGGGCATGGACATGGACATGGCCACGGCCATATTCATGGGGGGCATATGGGATGACATGTATAATGGGTTGTGACTCTGGAATAAGAAGAGCCAAGACTTGACCATTTATTTCAACTGAGGACAGGTCCAAGTCTAGTGCGTGGCAAGATATTTAATCAACTTTTTATTTTTATTTTTACTTTTTCAATCAGAAAAACATATCATGCCACGACACCCACGCAGTTTAACAACCCGAAACAAAACAAACCACGGGTGGCAAATGTGACAATAGCTCGGCTGCGTCCGTCTACATTGATTTAAATCACACTGTTCTCTTTTGGCATGCCGTCGGTTCCATGACTGTAGCCAAGAACTGGTCATCTCGACTGGGTGGAAATAAAAAAGACGAACAAAAAAACGAAACAAGCGCCCAAGCGCAAGCACATACGTGGTACTACGGTAAAGAAACTCGCGCCTTTTTCGGGGCCCGGCCATGCATGTCCGCAGCGATTCGGGTCCGCGGGGCCGCAGACTATCACTATGGTACAGTACATACCCGTGGGGGGTTCCCACCCGACGGGATCGTCAACCCATCAAACCCACAAACGTGGGTCCCGGAAACGTCCAGGGCAGATCCCACACGGAAGCTACAGTGGTAATCTAGATTCATCAGCCCCTCCCGTATGATAAGTAGAATGTTTCAAAGCCCGGGGCTCGACGACGGCTGTTGGTCTTGCCAGCCTTTTTGCCTCTTTGGGAAAAGTGCATTTTCTCTCTTTTCTTGTCTGACATATGAGCAACCTATGCACACAAGACACCTGCATTTGGCTTCCTCTTTGCCAAAACTCTACGGTCTACCTACACGCTGCACCACCCACCACACAAGCTAGGTTTGAGACATAGCAATCAAGGTAAAGCGTTTGTAATTAGGGTAGTTATTAACATTCAAAAATCAGGCCTTCTTTTTTTGTGGGAATACTTGTATGAAATTTACGACCCAACAACACTGCTCTCTGCTGTGGCAAGTGCCAACTGCCCACATACTATGTTACTTTGCGGGTCAACATGGAGCCCGAATAAGGAAGGAAACGTGGTTGACATGCACTGGCATCCATCCCATTTTAACGTGGCCCCCCCCCCCCCCCTAAACAGCCCACCCTTGTCGGGGCCGGCAAACCCCCTGCCGCGAACCTTAACGCTTGCAGTCGCGGT

GAGATCAGTGCGGACTCTTTTTTTTTTGTTCCTCTTCTCTGTCGCTGTCTCTCTTGAACCTCGCAGGGCCGGGGGTCAAACCACCCCCCGGATCGCTAGGTAGGTTCTCGCAGGTCATGAGGGGCGGGGGGGGGGGGGGGGGGCGGCTTCGGGCATCGCTTTTGCGCTTCGCGCGAAGGCCGGATTCAGAATCGGGTCAAGTTTTGACGAGGATAGCGGTGATAA

TCTTTTTTGTTTCTGGGTATGACCCGAGTAGAGACAGACAGACGTGCAAAACTACCACATAGAGCTTGGTCCCCCGGCAGTTAACGGTGCAACATGGAGCCTTAATTGACAAATGAAAAAAAAACACCCACCCATCGAGTGCCGAAGAGGAAAGGGGACCCTCAAGTTTAAAAGGGTACAAGGGGGGAAAAAAAAGACGCGAGATATTTTCCGTCTGATGTTTTTTATTAAACGCGCTGCCCCCTCGCCCGGTTTGCATCCAAAGCAAATCTCTTTGATGGGAAGCATTTTGACAGGACTGACAGACAGGTTTTGCCCAGCCTTTCGTTTCCGACTATCCTTGGTCGTTTTCTTTCTTAAAACCTAACTTTTGATTCTTTTTTTGTCTTTTCTTCTTTTTTTTCGTTTATTCTCGTTAAGTTTTTCGATGTTTCTACCATAAAACCCAAAGCAAAACCCACCTCGTCTACACCACTACAGACAGCCACCTATGCGTTTAGATCATCGTCAACCTTAATACTTTGCCTCCCTCTACCCCTCAGGGCTGTGCTAGCACGCCCAAAAAAAGTTTTTTCATCCATCTTTTTACATCTTGTTTACGCTTCTCTCATAAAACAAACCAAAAATGGGGATATTATCAGAAAAAAAGGCGGCCTCCGTGGCGCCGACGTCGTCAATGGTGACCGTCACCGTCCAGGACTCAGCAGCACAGTCGACAAAACCAGCCGAGGAAACACAGCCCGAAGGCTCAGCGTGGAAGTCGTACTGGAGAGTTTTCAGATACGCCGGGCCGGTCGAGTATGTGCTCCTGGCCATCGCCGTGCTTGCAGCCGTCGCTTCGGGCGTCGCCATGGCGTCGCAAAACATCATCTTTGGCCAGTTTGTCACCGTGTTTACCGACTTTCAGACGGGGGACGGGTCGGCAGTTCAGCCGTTTAGGGATGCAGGTTCTCAACTAGCGTGAGTTTTTTTTTTTTTTTTTTTTTTTTTTTTTTTTTTTCTCTTCTTGTTCAGCCAAGTACTGTTGCGCGAACCTTGACCGTCTTCTTCTCGACTCGCATGGCTAACCTCGAGCATGAATCCCGCAGCCTCTACTACGTCTACTTTGGCATCGGCCGGTTCGTAGTGGCCTACGTCTACAACACCCTGCTGACGTACTGCGCCTACCGCATGGTGCGCAACATCCGCCACGAGTACCTCAAGGCAGCGCTTCGCCAGGAGGTCGCGTACTTCGACATTGGCGACGGCGGCTCCATCGCCACGCAGGCCTACTCCAACGGGCGGCTGATCCAAAACGGCACGTCTGAGAAGCTCGGGCTGACCATCCAGGGCGTGTCGGCGTTTGTCAGCGCTTTCATCATTGCTTTTTTGACCAACTGGAAGCTGACGCTGATCGTGTGCGGCGTGGCCCCGCTGTGCGTCATCTTCATGGCGGTGGTGTCCTTCATCGAGGCCGGCTACGAGACCAAGGTCCTCGAGAAGCACGCGCAGGCCAACGCCTACGCGGAGGGGGTGCTGGGCAGCGTCCGCACGGTGCACGCCTTTGAGATGCGCGAGCGGCTGGTGGCCAAGTTTGACGAGTTTTTGGTGGAGGCGCACCGGTGGGGCGACAAGATCTCGTTCCTGTTTGGCGTGCTCTTCTCGGCCGAGTACACCATCGTCTACCTGGGCTACGGGCTGGCCTTTTGGCAGGGCCTGCGCATGCTGTCGACGGGCGAGGTGGCAAACAGCGGCGACGTCTTCACGGTGCTGCTGTCGGTCGTCATCGCCGCCCTCAGCCTGACCCAGATCGCCCCCTACAGCATCGAGTTCACCCGCGCCGCCTCGGCCGCCGCCCAGCTGTTTGCCCTCATCGACCGCAAGAGCGCCATCGACCCGCTGGACCCGAGCGGTGTCAGGC

**> MD412_chr04:5348263-5352263**

TCAATGCCAACAGAACTCACCGCGAGGACAGTGGTTTGTTGTCGTTCAGACTGTCACGCACAAGCACCTGGACAATTTCGAGCTCGTCCAGGTAAGCAGCAGCGCTCAGCACAGTTTCGCGAAACCCCCTGGTGGCCTCCTCGTCCCTCTCCAGCCTCTCCCGGTCTCCCTCGTCAACAGTAAACTCGGCAAAAAAGGTGTCACGGAAATGCGTATTGAACGAAGATATGTCCATGAGCGGCAATCTGGAAAGCCGAAAGATGACGTCATGCAGCTCATCCTCGCCGCCAAGTGCAGAAGCAACGGCGCCGTCGGCGGCCCGCAACCGCATCCGCAACTCGACGATGCGCTCCGCGACGCGCCTCATCATGCGCAGACGCCTGGTGGCCGGCGGGCGCCGAAGGTGGCGCAAACCCCAGTCGCATGCTCGCTCTGGCCTCGGCCCGCCGGTCAAGGGTCCCAGGGGCTCCACTTCGCCGCGGACGGCGTGGTCGATGCGCCAGGCGAGGTAGCGCTGCCAGAAAAGCTGGGCGGATCCGTCGGGTCTGTCCAGACGGCCCAGCCAGTAGGTGTTGTCGGGCAGGCCGGCGACGAAGATGACGTTGAGGATGTCGACGGACCAGATGCGGCTCACCAGGCGTAGACGCAAGGCACGCTTGAGGCCGCGCACGATGACGGCGTGGGTGAGGATTTGCTCGAGGATTTCGGTGGGGAGATGCGGGAGGGGCATTTTGGGCGGTGCGACGCTGACGATTTCATAGCGGAATGCTGCCCAAGAAAGATGGTTAAGACGAAAGGCCGCTCGCCCTCAAGGAAACTGCTGTATGCCTGACGATTGACAAATTAAACAAAGCTGCCCAATGCAAGGTACAATTAACTCGGGTGAAGTTTATCTCAAGTTCTCAACAAGCCAGCCGACTTGGGTCCGATGATCCTCCCCTGCTTGGGTTGTGGCATTCCATCCGATGGTGGAGTGAGTGGGTTGGACCCGCTAATTTGGAAACTTGAATCCCTCCAAATAGGGCTAGCCAAACAAGCTACAACGCCAAGATCAACTCCGTGCCTGCCTGCAGTCTGCAGGACTGCGGCTGATTAGCAGTTAAGTGGTGCACTAATAACGAAGGAAAGAAAGAGGGACGCCTTAGCGAGTAGAGAACTTTTCTAGCTCAGCAGTCGACTGGTGTCCGCACTAAGTATTCAATTATGGACAAGGACATATACGTACATACCTTACCTACCTTGCCTAGGTATTATGTACGTAGATTGAACGTCCTGGTCTGCATTTTTACTGAGTGGGTCCAAAGTGACTGCGGATCTACAGTAGAAAAGTTGTCTGTTCGCCAAGTGATCCCAAGACAAATCTTGTTTTTCAATCCTTGCCCAACTCTTCACAAGTTCTCTACAGTCGGTCGCATTTGAGCACATCATCCCTAGCAATGCACAAAAATCGAAGAAAAGCTTTCCAGGTTAACCTATTCTATATATTTACTCCAAAAACCGCCAATTGACTTGAGGAACAGTTGGATAATATGACCTGCGCCCGGTGGCGATAATGGTTGTAGTCCGAAAACACATAGAATAGACCATCCCTACAACTCCCCCTCCATCCTCTCACCCGCACTGTTCAACCGCTTACAAAGCCAGGACAGCGAGAGCGGCGGCGCCGATCCAGCTGGCAACCATCTTTCCGTTGGCAGAGTTGGAAGCCTTGGTAGAGGTGGCGCTGGCGGCCGAGCCGGTGCCCGAAGCAGTCGAGCTGGCGCCGGAAGCACCCGCCGATGTAGGCTTGGTGGTGCTGCCGCTGCCGCTGGCGGTGGGCGAAGCTGTGGCAGAAGAGTCAGCACCAGTGCTCGACACGGCAGTCGACGAACCAGA

TGCAGTCTTGGTGGTGGAGGATGAGGATGAGGAGGAGGAAGAAGACGAACCCCCGGTGACGTTGAACTGGTTCTACACGACAGAAGCAAATCGCAGGTTAGTAAATGTAACAAAAATTCAGGCCTGGGGGGGGGGGGGGGGGCTTGGATT

TGTCAACTTACCGACTGAGCGTAGATCTGCTCGGTCTTGGTGCCGTTGGTCAGGTTGATGGTAAAGTCGTTGCCGGCGGGGAGCTTGCCGCTGTCGACCTCGAAAGAGCCGTCGCTGGCCTTGACGTCGGAGGCGAGCAGGACGTCGGTGGGCGGGTAGGTGACCTTGTTGGAAAGGTAGATGTTGAAGGTGGGCTGGTCGCTGGAGACGGTCTCCCACTTGATGGTGTGCTTCTCACCAGAGCTCCAGTTGGTGTTGGAGTCGGGCGAGGTGACCTTGAGGGCAAGGGCGCTCGAGGCGAGCGCGAGGATGGCCAGAGAGGAAACCATTTTGAAGATTAGTTGTTGGTATGTGTTGGTTCTGAAAAAAAAAGCAAAATTAAAGATCTATTCGTAGAGCGAGTGACGGGTAGTTGGAGAAAATAAAGCACAATTAACGATCGTGGGCGTGACTTGACTGTTGGATAAAGTCTTGGCGATGAAGGTAAAGGAGAACGCGATGCCCTTGTACCGGCAGCGCCGATGCACGTCAACTTTATATCAGAGTACTCCGTATTTAGTGGATCAACAAAGACACATCCAGGCATGACGTATCCTAATTTTAACCCACATTATCGTCCATCTCTGGACGAGTCAGGGGTTGATTGGCAGGGTTCAGCTGGATTTGTGCAGATACGAAACGGTTATTCATCAAATCGCATTGACCGACTCGCTGTCAACTCGTGTTAGTGGAGTGTGTGATAGAATGGTCCACGGAGATCGTCCAGATACTTTTTTTTTTTCTGTGTGTTTTTCGTAACCCCGTGGAACATGGCAAAAAATAGCAGGCGCCGACATAACCGAGAGACAAAAAACGGTCCCCTTGTAACATTCTTCTAACTTTATCTAGTGCCATCAATCTTGACGCGTAAGCAAATTCTTCTAGATCATTTTTATTTTTTATTATTTTGCGATCGCTGTCCATTCCTAAACAACCAGTACCCAAATCATGTCGCCAAACACGCGAAAACAATGACCCCAGAGTGCACAACCGTGAAACTCAGGGATGACTGAAGGCGGGACATTAGTGGCCGAAAGCGAACCATGCCAAAACGGACCACCCCTCATGGCGGTTGAAGTCGATACGGCGTGTTAGGTCGACAGGTCCAACGAGAAAGAGCGCCGAAGACATGGATATGGCAAACAAACCCATGCCCATCAACGCGCTCCCGCAAGCCGGAATCACCCACCATGTGTCTTTTTCAGCCCCAAAATTGTAGAGTACCATGGAAAGGGAGACAATCGCCCCACCGGCAACAGTCAGCGGCAGAAGGTGCCCGGCTCTTTTCTCGCCTCCACCGACCTGGCGGCGCAGGTGGCGATGATGCGGCCCGACAGCGTCCAGCAGGCCAGCAGACCGACCAGGGTCCCGACCCCCCCCCCCCCCATAAGTATGAATACGGATCCGGCTTCTGCCGCGGAGAAGCTGTAGACCTCCTCGTATCTCGCAGGCACGTTGACGTACAGCCACACCTGCAGGCTGTAGAAGAACGACGATGGAATGACTATGCCGGGCCACGGATTGATGGAGGAGGATTGTTAAGAGGCCGCACCGATGCATCCCTCAGCAACACGACAGCATTTTTTTCGGCGCCGACAGCCGTTGCGGTGTCATGTGCAGTGCACTCTTGCGCAGGCCTGCAAATGTGAGCAACGCACTGACGACCTCCGACGCAGGCAAGCAGAACGAAGCAGCCTCTCCACCCCGGTACCGACTGCGCGATCCTCCCGCCTGCTGCGGGGCCCAATAGGGCGCCGAGACGTTGCCGACGGCGATGAAACGACAGCGCGCGGCTGCGGACCTCATTGTAGAAGAGGTCGGCGACGGTGGACGAGCCCAGGACCATGGGTGTGCCGCCCGCGCAGCCGGCGAGGAAGCGCAGGGCTACGAGTGACCAGATGTTGGGTGCGGCGGCGGCGAGGGCGGAGAATAACGC

**Chr.5**

**> MD501_chr05:281634-285634**

CAAATCCTGGCAGGACATGGCCGCTGATTGTGCCGCACAGTCGGCATCTGTTGTCGGCGCCAGCCCAGACGAGATCGCCATTATGAACACCCTGACTGCCAATCTGCATTTTATGATGGCCTCCTTCTACCGTCCCACTGAGAAGCGTCACAAGATCATTTCAGAGTGGAAGCCTTTTCCCAGCGACACTGTGAGTCAAGTTGTTGTGTTTTTTTCTTCTTTCTTCTCTTTTTCCTTTGACATTTCACGTGATCTTCACCTTTCTTCTTCTTGTATGCTCATCTGCTCATTGCTTTTTCTTTTCTCCAGTACGCCATTGCCTCCCAGATCCAATGGCATGGTTTCGACACAGCCACCTCCCTGGTTGAGCTTCACCCAGATGAGAACTACTACATCTCGACTGAAAAGATCCTCGCCACCATCGATGAGCACGCCGAAAGTACAGCCCTCCTGCTCCTGCCCGGGATCCAGTACTGGAGCGGCCAGCTCTTCGATATGCCCCTGATCACAGCCCACGCCCGCGCAAAGGGCATCGTGGTCGGGTGGGACCTTGCCCACGCCGTCGGCAATGTGCCTCTGAGCCTGCACGATTGGGACGTCGACTTTGCCATCTGGTGCACTTACAAGTACCTCAACGCCGGGCCCGGTGCCATCGCCGGCGCCTTTGTCCACGAGAGGCATGGAAAGGTCGACAGCGATGGCTTCAAGCTGCGATTGTCGGGCTGGTACGGCAACAACAAGGCCACACGTTTCAACATGGCCAAGGACTTTGACCCCACCCCTGGCGCCCAGGGTTGGGTCGTGAGCAACCCCTCGGGCATCGATCTCGCTAGCTTGGGCGCCGCCCTATCAGTCTATAACCTGACCACCCCGGCCGACCTGAGGAAAAAGTCGCTGTGGCTGACAGCTTACGCAGAGCACCTGCTCAACGGCATCCTCAAGGACGAGGCTGCATCTTCCGCGGGTGATGGGAAGAAGCCCGCATTCAGAATCATCACCCCTTCCAACAAAAACGAGCGCGGCGCTCAGCTTAGCGTGCTCCTTCGGGAAGGGTTATTGGACGTTGTGGGTGAAAAGATGGAGGCTGCCGGTGTTGTGTGCGACAGGCGCAAGCCCGACGTGATGCGCGTGGCGCCGGTACCCATGTACAACTCGTATGAGGACGTGTGGAGGTGCGTTGATGCACTTAGGAAGGCCGTCATGTCATGAAGGTGAAGCAAATGCGGTCTGTCTGTTGCTGGTCTCTCTATCTCTTTCACTCTCTCTCTCTCTCTTTTTTCTTTTGGTCCTCCCCCCCTTTTCCCCTTTTTTATCCATGTGGCTTCAAGGTTGGTAATCGTCGGGAAATGGTCGGTTGCATAGCGATTTTGCGGGAAATACATAAATACTATATATGATACACAATTTTTTGAAAATAAGCAAAAGGATGCCAAGAAGCTATTGTCTTGGCCTATCTTGGCCTGGAGGGTCTTCATCCCTTGCTTTCTTTTTTTTCGTGTGTTTGTTTGAGAGGCCCTTACCAATCTGTGGGTGTTGCTATACTACTTGACTACGTACCTAACCTATGCACGTACTAGCGAGGGAGTCTAGACCAGATCTGGTACTTTGGACTACGAAGTACCAAGACTACCTACTTAATCAACAATCAAATCACCGGTGTGATGCATGCCGCAGGGGCGCCACCCACACCGTACAGCCACCAACTTCTGCAATTAAGCATAAGACGATGTATCGCTGGAAGTCTGCCGGAGAAATTGCTACGGATGGAATGCATGGGCTGGCGGGCTGGCTGATTGTGCGCGCACGGCGGCGTGTTTCACACATGATGATAGGTTGACGTCAGTTTAGATAAGACCAATTGTGCTACTCTTTCACTGTGTCAACGACGGGTTCGAACTGATTTGACCGAACAGAGGCCATTGCAGATATATTAGGTACCTAAAAATTATA

TAATTAGAGGTACTCAAGGTAGGGAAGAAGGGCAAACTCAAGGTAGGGAAGAAGGGCAAAGGAGCATCGTGTACCTCTTACCCAAACTCATGCTCGATTGAAATGCTTGCAGTTACAGTATGTACCTTACTGTACTTGCTTGAGCCATGG

ACGTATCGTATTTACATACTATGTTGTGGAATGGAAGGGCAAGCAAGCTTCATGGGTGGGGTGACCCAGGGTTTCCGGAGCTGTTGTTCCCCGCCTGTAGCGAGCGATAACGACGGGTTGCATTCTCGATACATGGCAAAATAGGTAGGTAGGTTGCTGTATACGGTAAAGTGCCAAAAGTGCTTGCGCTCGCCTACCTATCCGGTGAACCCCGTTTTGCCCCGTGCATGATTGTCTTGATTGGAAATGCAGAGTTGCTTCTCACTGCCGCGTGCAAAACAAAAAGAAATAACTAGAACTAGGTGCTGTCGAGTTATTTCTCTTGGTACGGATGAATTTAGACCCCGCCAGTTCAGTAGCTCCAAACAGGGTCCTGCGTGGCGAACCATCCAAATACGGCACTCCCGCTGTCTACAGTTGTACTATAGTAAAAAGTCACACACACCAGCCCCAGCGAGCCCACCCACCCGGGAGAAAGTGGAGAGGGCCCAGTAGCGCGCCCCAGTTTCTTCACGCGAACTACTCTGATCATATTTACTTCAGAAATTCCGTTCTATTCTGGAGTTCTAGTCTAGCTTTTGTCACCAGCGAAACGCACTCATTCTTGTTTTATTGTTTTTTTCTGTTTGTGTTTTTCAGTTTTCGCCAAACCAACAAAAACTTGAACTCAATTCAATCGAGACGCTCCGCCAGACAGATGTTCAACGATCAGACACGACGACCCTTACACTAGGCAGGCAATGTGCGCAAACCTCTCAGAGGAGTCGGCGATCCAGATTAGTGACGGGCGCCCTCTCAGTCGCAGCCCCGAATGGGGGGATCACGGCGGCGGGCAGCCCCACAAGCAGCAGCAGCAGCACCGTCAATACCAACCGCATCACCGCCAGCATCCTCATCATCACCAGAGTAATCAAAATTACAATCACAACCTCCGCCACCAGTCGACCAAGTCCGCTGCCGCCAAAACACAAGACCGTCACCAGCAACCAGCAGTGTGGACTTGGTTGAATGCAATTGGGGCTGATGGTGCTCGCATGGTGACTCCATCCCACCCGTGTAGTCCCCCGACCCATGTGCCGCCAACAGATTCCCCCGAATTCGGCACCACCACCACCAGCAGCTTCGTTCGACCCTTGGTCCGACGGCGCTCGACTTTACCAACCAATTCACCCATGTCAACCCACGATGGCAACTCAGCCGATGCTTCAAGAATTAATTTCGCTTTCGACCCAAGGCAACAAATCAGCACCAGTGGTGTACTTTTTGGCGTCTCTGAGCTTTTTGATTGGCGACCTCCCCACGTGGATGCTTCTCATGTGTGTTATTTCTTTTCCTCTTCTTTTCTTTTCTTTTCTTTTATTTGTTTTCACCCCCTCTTTTACCCGGATATCCGGACCTTGGGTCTCAATGCCCTCGCCTCATCCCTCGCCCCCTCTCCCCCAACCTACCTACCCTGGTTAGGCAAATTGTTTGTTAAGCTGACATTGTTTTTCTTGCTTTTTTTTTCTCCGCTTCTCTCGAGAAGAAGCGCAAGGTTTCGCCTCCACAGACTTGCGTCAAATGCCATACAACCGAGACACCAGAATGGCGCAACGGTCCAGCAGGTCCCGGGACCCTGTGCAACGTCTGCGGCTTGGTGTTTGCAAAGAAACGCGCGCGCAGAGATCGAGACTCCTGGTCTCTAGATGTCAATAATGCGCGCGGGGGCTGCTAGCACCCAACGAAATTTTCTGCAGTCGCCATGCCACCAAACCAGCCATTGATCCAAGACATAGTCTGGGCCTAGGGTGTCCAGTGGCCTTCTCATCCTACCTAGGCTACCTAGGTTCTCAGTCTAGCAGAGGGACTACGGAACCTGTATTCAGGGTGGGAAACCATGCCCGATACCAAGATTTAGCCA

**> MD502_chr05:1120549-1124549**

GTCAGCGTTTAAGAGGCAGCAGGCGGCTATATGGATAGGCGACATCCTTGGCATTTAATCCTTTCCAAGAGGCCAAGACATGATCCTTTCGCGCTTTTGACTTGGAACTCAAACCGAGACTGGTACAAGTAATTCCGCGCCGGATAGACTGAAGTCCGCGGTCGAGGACTGGCCCCCGTCGATTGGAAGGAGGAATGCCAGAGCACTATATATTACTCCCTTGAGTGAACTAGTCCACCTGAATTGTCATGAGGATAGCACAGCTGCGAAAGAGCTTGGTGGGCGGGCTGACTCAACTAACAGCGCGAGAAAATGTGCGATTAATCAATTGCTAGGCTGCTCCAAGACTACTGGGGGGCCCACATGGAACAGCCCCCGAGTAGTAAGCTGTATGTGGTATAGTAACCAGAGCTTGTGAAGGCCAATATGAAAGGAGCCATGAACCTGCTTGCATTGAAGTTATAGGTGACATATTAGGCCAAAAATCCAAAACCCAAATCACGGCGCCGAGCAACCAGAAAAACCGTCAACATACAGCTCACGCAGGAGATGTCAGTTTTGTTTTTCTTTTCTTTTTTTTCCTCCTTTGTCAGTGGTTCGGATAAACCCACCATGAAGTGCTTAAAGTCTCGGGCTGGGCAACTCTAGCATTCCTCTTTCAAACTACCTATGGTCACCCCGTACCGGTGATGAAATGCGCATTATCTCGGCATTCGCCCTGCAACACGTCTCTAAGCAACGAGCCCACGTACCCAAACGATGGTGGTGAACATCCTCTGGAGTTTGACAGGGGCCAGCAACATGCCGTTTTGAAACGGCACGGTAAACATAGGTTTTGAAACGACACGGGTAAACATAGGTTACCGCAGCCTAGTCCAAAACGAAAACCTCCGTACAAGGTTACCAAGAGCCTGGGCCGGGCGCGCGTGGCTGAGAAAAAGGTGAAAGGGAAATTGGATGGTAGAAGAAAGGCCGACTATGAATTCTGCATGGCTCAGGGGGAAAAGCCTCTTGAAAGGGCTGCTTTGATGGCTGCCAAGGATCGTCAGAAAACGTCCTCGGCTTCAAAGGGGGTGACTAACCCTCCCGTACAGAACCAGGCCACTCAGACCAACCCCACCTCGTTACTTGGTCCGACAAGCAGACCGCTGCACAAGATAGACGGCCTCACCTTCTTCAGTTTTGAGTTACGATGAACTCGGTTTTTCGAATGGAGCTGCCAGGAACCCGTTTCTGGAAACAGGTCTTGACGGACTGCCTCATACACCTTGGTGTGATGCGCCACGAGTGATGGACATTCTGTGCTCAGGCAGCTGGAAGTCATTCTTGCAAACCCTACCCATACCTGACCAGGCTGAATTATTTTCTAGGTACAAGTTGTATGGGAGAAAACATTATAGTCACCGCTCAACCCTGGTGATGTTGTCATCGGTGAAAATTTCTACTCCTATTCGACAGACAGTTCTCACTGGTTCGAATAGAAAGGATATGTCACCCATGGCTGTTGTCGGCTTTGATGTAATGTTAGTCGTACGCGTACATTTGGGATTCGTAGCTCACATTTGCAAGGCATTCACAATAATTGAACAGTTAAACACTAGGCTAAAGGATTTAATGCGTCGAATTCTGCTCATGATTATCATAAAAATAACACACAATCTTGATTTTACAAGTCCGCTCTCAGTCGGCTCTTTTTGGGGGCTTTCCATCACCATTTTCCGCATCGATTCCTTGGCTCTTGGCAGCCGGGGTCGCGTCGCCAATGTCGCCCACGTCCGCCTCGTCCCATGCGTCCTCGCC

ATTCTCGTCCCAGTTGTCAACCTCCTCCTCCAAGGTCAAGCCCCTGTCTCCAACGGGTCCACTTGGTTCCGTATAGACGGGAGCATCCGTGATACCCTCTTCATGACCACCGTCGTTAGAGTAACCCAGACCCTCGCCCAGCTCAACATCCTCTTCGCCAGGGTCACCGTCCACACCGTCGACAGGCTGGTATCCCTTGCGCATGCGCTTGTGATACCATGAGCA

CAGCGCCAGTCCCGCCAGGCTACCAACGAGGTTCGCAGCGATGTCGTAAACGTCGAAGTCTCGGCCGTTGGGAAGCAGCGCCTGCAGGAACTCGGAGCCGACGCCGAGGGAGAATGTGCAGACCGTTAGGGTCAGGTTCAGAGTGCGACGACGATTCGTGTCGACAATCCAGTAGAAGACTACAGTGAGGGCGAAGAATGTAATGAGATGGAGAAGCTTGTCGTTGATAAGCTCGCCGAGTTGCAGTGACGTCAGACCGGCGTAACCTGCGATGAGGAGCAGGAAGAAGAAGACTCCTGTGAAGTTGAGATAACAGGGAGCTGTTAGTATCGTCGATAGATCATGGGGTAATCGGGCTGAAAATAAAACGAAGCTCCGCCTTACCCGCAAAAGGGAGTCGTATTCTCATGATGGGCTGGCTCTGTGGATCGTGTACAGTGTGCAGTGAAGTTTCGGTTCGCGAAGTGGCAGACCAGGCTGTCGTGATTTTGCAGTGGAAATCCTGGCCGAATACAAGATTCGGTCAGCTGAAGCTCTCGGAAGAGAAAGCTTCAGCTATGGTGGCAATCGGTCGTGGGACAAATGCTCTCAGTTTAGTAGGAATTCCTGCCGGGAAAAGGCTGTCGTGTTTGACGGAAGAGACACGACAAGTGAGCCCCCACAATGCACAGCTTCTGACGCCCACCCTCTGGAGGTTGGTTGCAGGGATGCCCGACGCCGCGAGCAGCTCTGTGGACACGACGTGCAGACAGCTTGGAGGTTACGACAGGGGTCAATGATTCTTCTGCCGGATTAGGCTTAGCGTTATGGTACGTGCTGGACCGAAACCCGCCGCATCGGCCGCAGTCTGCGGGAGTGGAATTGTGTTTGTACTGTCGAGGCTAATATAACCAACGATGGCAAGCTTGCCTCATGTCCGATTGAGACATCAGACAGGATGTCGGAAAGTCTGGGTTTGGTTTCAATTGCTTTAAGATAGCAAGGCAGTATACCTCAGCTGTGCTATATGCTACCTAGGTAGGCGAGCGGTAGGTAGTAGCTAAGGTACCTAAAGGTAGGCCCAGCTAGGTACAAAGGCATTAGAGAGTCAGGCGCGTCATTCGAGCATCTTACCCATATAGTCACATGAGATATCACGTGCGTAGATCACCTTGCTGAAACAACGTGTGGGGGAGCGACGACATCAGTTAAGAAAGCGGGTCCGGAGAACAGTGTTCGGGGGTACTATCTTGGAAGACGACGACAGGTACTGGAGCTTGCAGTGCAAGTGGGATGAAGACAAAGCAACTAAATAGATGTAATATTGTGCATCGTTAGGGTAATACAGAAAGCGCATGATTGGATTCTACGGCTTTTATCGAAAGTTTATGGCCTTCTTCTCAGCAGGGAACATGTACCTACCATCGCTCATGTGGAGACCAAAGGAAATTCAGCCAACCCTGGACTGGTGCAAGTGTACCAACCAGCGCATGAGCCAAATCCGTCAACCTCAATCGTTGATTTACCGCGAAATTGCCCTGCCTTCAATCCATCGAAACATCAGCGCAGCCATCGATATTGACATTCAAACTGGTCATTCCATCTCACACGAAGGCGGCCTTTCTTCCCATGTAACTCTCGGCGCCTGAACCTTGCTGGTTCCGCCGGGACGCGACCGCAGGATTTCCGCGCATGGCGACCGACCGACCGCGTCCTACAACTCCGATCGCATAGCACAGGGAAAGCAGCCACATTCCCCCAAGCATGTCTTTCACCAATGGCCAGCACGGCTGGTCAACCCCATCAGGCGGCCTTCCCAATGGAATCAAGAATATGCATCAATTTGATGATGGCCCGGCGAGAACACAAACTCCGATAGACACTTCCATGACATTGGTGCCTGAGGCATCCGATCTCGCCGAGGAGGAACGTCGCGCGCATTTCTCCGAGATTTATCGCAAGACCGATTCCAAAATAGCGCTATTGTTTACCGAGG

**> MD503_chr05:2125209-2129209**

GGAGTGGAAGAGGCTCGACCTGGCCGAGGCGGAGCAGCGGAAGCGCGGCGGCTGGGGCAAGCTCAGCTACGAAGAGTTTGAGGAGATTTATCGACGCGAAGAGAGAAAATCGAGCAGGCTTGACTACCTGGGCAGCTGGATCGACGTATGCATCCCATACAGCTAGGAACATCGAGGCCCGAGCCTGCAGATTATGGTGCCTTCCTCGTCCTTTGACGACGCCGATATCATCATTTGACGGAGTCGATGAAGTGTCGTTGGGCTTTATTAGACGGTACTTTTAGAGGATGGGTTAGAGTGAGCTTAGGGGGTGGTCTTCTTTGTAGGAATAGGATATTTTACGGTTAAAAGCTCTATTGCCATTCACCACCTGGCATTTCACAGGGCATGATGATACAGCAAGGGATTAGTACAGCACAGGAAAAACTTTGTGAACATTTAACGGAATGTTTGCTAGCCCAGTTTAGCGTAGTTCTCTTTATACCCACCAGCGACAATCATAAGGAACATCTCTCTCTCTCTCTTAAGTTAGCATATATGACCAAGTCCTGGGGCCAAAGTGAAGTTCACAAATGATGCAGGAGCCATCTTCCACACACGTACCTACAATAAAACCTATTTGGCAACTTCCCAAGACACCACAGTACATGACAACAGCGTCGCGACTGCTAATCGGTCGAATGGTGCTTGGTGCTTTACTCCTTATGGATTTGATTGGAAAGCTACACCTCCAAGTGAGAGAAACTCTCCCAGTCAACGATGGCAATGTGATTGTGCTTTATTATTCTTTGTCTTGGACGGCGAACAGGATCATATCCATACAGAAGCACTACTACATGCCAGCATGTTTATTGAAGCAGAAAAAGACAAGGCGGACGCTCGGTCTTGGGCGACGGAACCATGGCCGATCGGGCATTCTAGCGAATTTTACGAATACAATACCGAGGTCTTGGAGGCAACCAAAGATGAATGCTCTGACAAACTAGGGTGAGTCCTTCTGACGTAAGACTGGTTTTTCTCTGCATGTGTTCATGTCTTCGCTGACAAGAAATTACAGGCGGGGCTTGAAGTCGGCATATGAGCATCGTCGCAAAGTATGGGCTTGTCACGTATGTGGGAAGATTGTGAGCAAACGAGGCGTCGGTCAAGATGGCAAAGAGAAAGACGAAAAAGAAACCAACCAGCAGGACGGGCAGTCTCTGTGCAACGGCCTGACTCCATGCGAGCTATGTGGTTCCGCATGGTACTGCTCCGAGAAATGCCGTCGGAAAGACTGGCTGTTACACAGCCTACTGTGCACCCAGTACCGACTCTTTCAGCAATACAAGCCGTCATCGGATAGCTTCAGTTGCATCGGCATAGAGCTCCCCGTGGAGAACAAGACACCGAGACTGGTATGGGTTAGGTACGGGAAGGCCTTCAGCAGCCGACCCCAGCAGCGCCGTGCCGGGATCGCGAGGACAGCAATTGCATATCGCCACGATCCCTTTTACTACATCGGTAACATGATGGGTGGCATGGTTCAAGTAATTAAACAACCCGGTCCCTTTTGGTGCACGCGGCTCTACGTGGCACGCCGACAGACCTCGTTGCTTCATCCGGTCAACGAGTGCGTGAGGACCCTGCTGCGGCAGCTGGGTCCTAGGGCGTTTGACTCGATGAAAGGGTTCTTCGACCCCAATAAGCACCTGCAGGACGACAAACTCATCTGGAGGGGGCCGATTGTTCTGTTTCGGCGGTCGGAGATGTTCTTCACGATGAAGGCGGCGGATTTGGAAGGCCCGCTCGGCGGACGTGAGCTCCGGTCACTCGAGGTGCAGATGATGGAGGACATGACGCTGGCGGATCTCAGCTGGGCTATCGAGTACCTGGCGTGGAGGCAGATGGAGGTGGTGGAGCATCCGGAGACGGCTAACCTGGTGCCGGAGGACTGGTCACGGTGGATACGGT

CCATGCCGGATGAGGAAGAGGTGGAAGAGATAGACGAGTGAGGGGAAAGGTGTGGATGCATGTGGATGCATGTGGATGCATGTGGGATGCAGGTGGATGCATATGTGGACGTCGCTTCTGGTGTGGTAAATGCAGGGAACATCGTGCCTGCCTTTACGCTCTGGTCTTTTTTTTTGGTATTTGTCATTCAGAATTTTAACAACCAAACCCACATTCATTTTTTTT

TTTTTTTGTTTCCTGTACGGTACCTACGGAGTGCTTGGACTCTCTTTTACCCCAACCTCCGCCGCCACTCAAAGTTGTGCGGCGGCATATGAACACGGCAGGGATCTCAATGTAACTAATGTGCCTCCAAATATGACCAACCGTTTGACATGGTGCTTTTATTCGACGAGTTGCAGCCCTCTGAGATATCTTGTCAGATCGTCGGATTTTATTCGTCCGTCGACCTAAAAACGGCATACTGCTGACGCAATGATTGCAATGAAGCTCAATAGATAAAAGCATCCCAAGAGGCCCCCAATCTCGGATGCGGGGTTGCGTTTACGGAGGCCAAATATGAGTGGAGATGCTAAGATGTCCGGACTCTTTGGTTCTAGGTCTAGGTCTAAGCCGAGGGGCAAGCAAACCTTTCCCTTTCACGACCTTTGCTTCCATTCTTATCTTGTTCTCATCGCATCCTCGTCCTCTTCATTTACCTGATTAACAAAGGGCCAAATTGAGTCACCAAACGAGTTTCGGGGATCTTCTAGCAAGACATTCGCAATAAACAACCACCATGGGAGGAGAAAAGGACTTTATAGTGCCGCTCGTGATCGGCGGCAAGGAAAAGACCACCAGCGACTCTTTCCCAGTCGTTTCTCCTGCGACCGGCGAGACTGTTCATAAATGCAGCAATGCGAACGTGGCCGACGCCGAGGAGGCCGTCGAGGCCGCGGCTGCCGCTCTCAATGACTGGAAGCGCAAGACCCCGGCCGAGAGGCGTGACATATTCCTCAAGGCAGCCCAAATCCTCGTTCAGCGCACGGATGAGCTCGCGAGCTACATGGAGAGCGAGACGGCCGCATCTAGAAATTGGGCCAACTTCAACCTTGACCTCACCCGCGAGATGCTCATCGATGTCGCCGGGCGCATCTCGGCTGCCACGACAGGCGCCATCCCAGCCACCCGGGACGCCAATCTTAGTGCTATGGTGGTCAAGGAACCCTACGGTGTCGTCCTGGCCATGGCTCCCTGGAATGCCCCTTACATCCTGGGCATGCGGGCGGTCCTGTTCCCTCTTGCCGTCGGCAACACGGTCGTCTTCAAAGGGTCCGAGCTGAGCCCCCGTACCATGTGGGGAATCTGCTCCGTCCTCGCCGAGGCTGGAGTCCCCCATGGTGCGCTGAGCTTGATATTTTGCAGCAGGGAGACGGCAGCCTCGGTGACCGAGACCCTCATCGCCCACAAGCACATGAAGAAGATCAACTTTACCGGCAGCACGTCGGTCGGGCGCATCATCGGCCGCCTGTCGGGCCAGCACCTCAAGCCCGTCCTGCTCGAGCTCGGCGGCAAGGCGCCGGCCATCGTGTGGGAGGACGCGGACCTCGACAACGCGGCGGCGCAGTGCACCCTCGGTGCGTACCTTGCGGCGGGCCAGGTCTGCATGAGCACGGAGCGCATCATTGTGCACAAGGCTGTCTCGGAGCAGTTCCGCGGCAAGTTTGCGGCATGCGTCGACAAGTTCTTCCCGTCCAGCGCGGACGCGCCCATCCTGGTCCAGTCGCAGGGAGTCACGAGGAACCACGAGCTTCTCAAGGACGCTCTCTCCAAGGGCGCCGAGGTCGTGGTCGGCGACGCTGAGGCCAAGGAAGCCAACGCCCACTCCATGCGGCCCGTGGCCATCAGCGGCGTGACCCCAGACATGGACATCTACATGACCGAGAGCTTCGGGCCGACGGTGTCGCTCATCGAGGTCAGCAGCGAGGAGGAGGCACTGAGGATTGCCAACGACACCGAGTACGGCCTCTCGTCCGCCGTCTTCACCGCGGATCTGCGCCGGGCATTACGG

**> MD504_chr05:3255206-3259206**

TCGGCGAGGAAACCTCACTTGTCAGTTTGGTATCAAGTTTTGCCAATGTCTTGTCAAAGACTTTGTCGACTCAAGTGGTTTTTTGTAGCAACGTCGCTAGTTCTAGAATAGTAGCTAGCTCCCTGTAATTTTTTCTTCCTTTTCTTGTTATCTGCAGGAGAGCAAACCCTTTTTTTCCCGCTCTTACGTAGTTTCAAAAACTCACAACGAACTCCGGTCCTTCTCCAGACTGTAGTAGGGCTTGAAATACCTCATCACAGCTTGTCATAAAATATACCTTACCTAGTTGATCGAATGTTTTTTTTTTCTTTTTTTTTTTTTCTCATACGCACCTCTCAGTCTCTTTCTCATTCCACACACCTCTCGTCGCACCCCCAGCCCACCTTTTCGAATGGAAAATACCACCTCGGAACGCGCACCCACCTGGATCGACCCTCTCTCCTGGCCCTTGCAACTTCGATCCCACCTCACCTCGTCTCGCCCCCATTCCCACCTTGAGTTATACAGGATTTCTGGATACCCAAATCTTGGGTTGCCTCGATCGAAACGTGCTGTTTTATATATAACCCCTAAGCTGCCATCCGGGCACCATCCTGTCTAGTTCCGCTTTTGTGCCTCATACAGTTTTGTACAAAGAAGAGAGTAATATCTCAAATCCTCGTTGCCGTCCCCTAGCTTTTACGCTAGCTACGCCTCCGCGAAGCTCCCCCAGTTTCAAAGTAACTCTTTTCTCACATCATCAAGAAGGCACAATTTACACTACCTCATATTGGTTTTTACGCTTGGGCCCCGGCAGTGAAGGCCTCAAGATCAGCATCTGATATTTACAATACTCAACCCACTGCTCCTTCTCCCAAGATCTTCCGAATAGGAGGAAGAAGAAAAAAAAGGAACCGAGATAAATAAAACGACAAAATGTGCGACTTCACCAAGAACTACTACATATATACCTCGTGCCTCGACCCCGGTGCGCACTTTTTCAGGACCTCGGTAGACGGCAGCCACAAGCATGCATGTCCCAAGGGGCCTCACGAGAGATACATTATGCTCCCTGGCCACTGTCCTCTGTGTGATTAGCTGCCACAACCATCAAACACCATGCAGTGTCACGGGGTTTCCCTGCTAGTTACGGGAGTCGCCACGCAACAAAACAAGCCACCTTGAGAAAGCCATCAGAAAACGGACTTGGCAAACGGCAGGATCGGAGAGAAAGCCAAGTTCATCGTTTTTTTTATCTTGTCAGTATTCCCACAACAAAGTTTCCCTTGTCTTGAAAGATCATTTTTTGTATTTCTCTTGCTGCAACACAAACTGACATTCTACGACCGAAAAAAAAACAGCACCTACTAATACATTTCGTCAAAGAACGAGATAAAAGTTACACAAATGGTATCCGGAAGTCTCGGGCTTTCCCCGAAACGAGCCGAATAAAAGTCTGCACTAGAAAACAGTGTTACTCCTCGACCAAAAGTTTCGTAATAGTTTGTCGGCCTGGAGATATCTTCTTTCGTCATTCTTGGAGCTATCTGCCTTCTCTTTTTACCCCGATAATTCTACCCCAATAATTTTCCAGGAGATGCCTGTTGCCTGACTTGCCCCAAATGGGCTTCATGACTTACGAAGGCATTTTCCTCTCACCTTGTTTTTACGTCTTCATCCTCCTCTCTCTTTGGCTTCGAATGGCACGGCGTTCAGGTTGTTCTTTCTGGGAGGACCCTTTGAGTTTCGGCGATACCCAAGCTCGGTTTCTTTTTTTTTTTCCTCTCTATTTTTTGCGTATACGTCAACATGTGATGGTACTCTGCTTTTTTTTGGTATGTTTTGCAGTTTATTTCCTCAACCTGAAATTTACTTCCCCAAGGCCGGCCTTGTGTCAGTTGCAAATCAAGAATCACAGACGAACAGACGAGCAAACAAACAAACTTACAAGCGCCATCGGTGACATTAGGT

ACATAGCACTCCCCACCTATGTAACCATCGACGACATTAACAACGGGTTTTCTTCTTCTTCTTCTTCTTCTTCTT

CTTCTTCTTCTTCTTCTTCTTCATGAGAGGCTTTATTAGGCCCCCAGATTGGGTTGCAAGAGCCCACAAGGGAAATAATAGCCGTCGGCTATTTAGTTCCCGCCGTCTCCATTTGGGAATGCAAACTCAACCATACTATTTGACAGACGCGATTAGCGGGCATATCAGACCAAAGAAAGTATATTTTGAATGCAACAGACGGTGAGGCATGATGTGTGAGGGGGAGGCAACGCGGACAGCTCAGCCCTGGGCAGACTCGTCATCGGAGTCTGCAACCTCCGAAAGAGCCGCTGGGCCCCAGGCGTTCTCTCTGTTCTCTATCCTTCCATACAAGCTGAGGCCAATCAAAACAGTGGAGTAATACGAGACGTGTTGTCAATGAGAAACATGAGGAACAAAAAGGTTCATCACCGGGGAAAAAAGTCATGTGGTTCTGCTCATTCGGACCATGTCCTTTCGCCTCTCCACCTTCTCAAATATCGGCCATGCTAATTTGGGGAACAAGAGGTATATGAGCCAAGTCTTTATGACCATCCCCACCCCTCCTCCGCTTGAAGGGGGATCGTGTTCGTCTTGTTTTTTTTTTCTTCTTTATCTTGGGACGGTCCACAGAGAAAATAGAAATCTGAACACGGCAGGGAAAACATCCCGAGTGTGGCTTGGCAGTATAGGCAAATCATAATTGTTGATCAGCCGCCTTGCCACTCCGGGAAATCAGAGGGGGGGCGCCGAAAGCATTGTGGGTGCTGAATTAGGCAGGACCGGTATTAACTATAGGGCTCAGTAAAACATTATTGCCCCGAGCCGTTAAAGCGTACCAGAGTTCGGTGGCACGGTCTTATCTGTCAAAAAGACAATAAACATCCTTCCCCCCACGCCGGTTGACATTGACATTGACGTCTTGGCAAGGCGCAAATATTCTCCAAGCGCCAGCCGCCTTGATGTGATAATATACAGTGACTTGCGCGCTATTTCGAAATTGTACATGGTAAATAAACCACGCCAATCCAAGATGACCAAACACTATAGTATTCTGTCGGATCTGGGGAATGCTAATCGTGAGCGGGGTCCCATAATTTGGTAGCGTAATTGTTGGGGGGCATAGACTGCAAATCCTTCCGCGAAAGTTATGCTGCCGGCGAAGCTAGTCCTCTTTGTGCATGTAACCACAAGCCGAGATCGTGGGAACATGCACGACGGAATCTAGAACTGACGCTTGTCTGAACATAGATAGTAATAATCTCAAGGGAACTAAGTAAAATCAAACCAAAAATGGACAAAAAAAAAGTATTTGACATTCTCAAAAGAACAGGCTTAACCAAGGCTCTAGCCGCGGATTGAACGCGGGACCTCTCGCAATCGTGCTCAGGAGTTAGAACCCTAAGCGAGAATCATACCACTAGACCACCAGAGCTTGTTGAAGACTTGCTGACACCTCCTGCAAGTGTGTCTGAAGGATAGCAAATCCAGCTGCCGGTGAGAGAAAGGGAAAGACAACCGACCATCGTTCAGAATTCTTTTTGGTCGGGAACACATTCTTTGCGACCCCTTCATTATCTAATGTAAACAATAGATCGGAAGAAAGCTGCTCATCGTCGACGATCTCACCCGCGACATAATATCTATGATTTACCCATATCACGGCCCGTTGTAAGAATTGTCGAAATTTGTGCATCAGCGAGACACAAGATGGCAAGGCTCGTATGGACGGGGCAGCCCCATCGGACTGGATGAAGGATAAACTGCCACATGGCAGAGCCTAAGAGCGAGAACCCCCGTCTTCCAGGTCAAAGGAGAATACGTTCGTATCATAACTCCAGGTGCAGGTTGGCACGATGAGGGGCGAATGCAGCGCCGAGGAGGCCTGTCACCTTACCACACCGAATCCATCCATGGTTTTGTGGTTGGAAGTGCAGTTGCAGGCCTTGATATAGGTAGGGGAGGTGAGCGCTAACCGGATATTTACCTGACGTGG

**> MD505_chr05:3527911-3531911**

AGGATTTGACGCCATTACCAGGTGAACATATTTATCCACCAGCGCCCTATTCTCATCCTCAAACAGTTGCCAATCAGAGGGTTTTCGGTGCTGCATGTGCAGCGTCGACCGTTTCATATATGCCATGTTAGCACCTTGCTGGTCCTGAGCGAACAAATCCTCAATCTCCTGCATATGGGGTCCATGATCCACGAGTCCCAAACTTCTTTTCGCATGTAAGAAGATGAGAGATTGAAGTACTGGGGAATCACAACCTAAGACGTAGATCAGTTCGTTGGCAAGCGGCGGCCGCCTCCGCTCTATGATCAATTTCATCATATCATCCCAGTGATGGGCGAATCGAGTGAGCCATTGGCCTAGAGAATCCTGGCTGTTAGGTAGAAAATCCACCGGGAAGTTTATGAAGTAGCTGAACATTGGTCCAGATACAAATTGATTGGACAGCAGCAAGTCACACACTGCTCTGCAGGCCTTATCAATGGTGGACGTGTCGTATGCAGGCATAGCCCTATATACCACAGATGCATCGTAGGACCATCTACAGTAAATCTGATGAATCTGTAGATAGACATAGTCGTGTTTATGACAAGCACGTTCCAGCAAATACAGCCTTCGACGGTCAATTTGAGTTGAATTCGTATTTTGAGGGTCCATGCGGTCAAGGTAGTTTTTGATCCACGTTTCCCAAGTTGCGAATGGCATCCTGCCCCTCATCATTAGTATGAGAGTGTACGAAACTAGGCTGGGAGAGTATAGACTTACTCAAGCCTCTGTCTTTTTGCACTGTGGGGTCCGGGCTGCTGTACTGCAACGTGTGCAGACTGGGGAGACTGGACCGGAGTGTTAGGCCCGACGGGAGATTGAATGTGCAATATATTGATACCTGAGGTTACCGGAATGCCTGGCATGTTGGTAATGGGCACATTTTGAAGATATTGGAATTGCAAATCTGACGGGTTACGAATAGATGCAGCCAGCGAGGATTCATGAGAGACACGTCGAGTGTGAGTGTTGCTTTGTAGGACCCGCTGAGTGCGGCTGCTGTTAGAAGATGCAAAGCTGGTGTCTGGGCCACTGTTTGCTAGTGGTATGTGACCTGGGTATCCGGTGCCAATATTGTGTATAGGTCTCTCCATGGCCTGGGATATGGCCTGGGTATCGATTGATAGAACCGGCGCTGCTCGCGGCGGATTCGGACTGTCGGGCGGGTTCGCCGCAGAGGCTGGACTGGGTTCGTCGCTTTGCGCGGGAGAGGGAAGGGCGTTGAAGGAGGATCTAGCAACAGACACCTCGGCATGTTAGCAAAACATGATAGATTGCAGCTTCAGGAAGAGCATGTCGCGTCGCGATGCGTCCAAGCGTGTGATAAATCCCAATGGTGTCCGGGCTGAAAGGGCACCGGAGAGCATGTCTCTGCAGGGCCAAGCTAGCAGAACTTCAACCTACACATTAGGTGTCGTGAGAGATTGTAAAGGTCGCGGAAGCGAGTGCGACGAGGCTGCTGCTGCGGATGCCGTGGTGGTCCCAGGCGGGTTCAGAGGCGTGGTTGTCGGGTTCGACTGGCGGGAACGTTTCATACCAGCCCGGCGAGGGGTTGTGGAGTCGTCCGCGTTGTCGCGGTCGGTGGTCATCCATGCTGGACGTCGGCCTCCCAAGCGACTGTTCAGCGACTGGTTAGCAGCTGCGACGTCGGCCGGCCCCTGGGGGTGCCGCGTCATGCTGAATGCGATCCTCGGCGTCTCGCAAAACTAGTTGTTGAGGACGACAATCATGAAACTTTGGCAGTGTGATTTTTTTTTTAGGTTTCTTTTTCTTGGGTGGTTTTTTCTGCCTGGTGGTACCTGGCGGGACTCGTTCGCGTTCGCCGCAGCGGCTCTTTCGTTGGCGGCGTCGCGCCGCCTCCACACCGCGGCAACTCTAAAATTGAAGTGGGGGTGAAGGGTCGACGGT

TGGGTGATGGAAAGGGCTCAAGTACCGCCAGGTAAGCAGAATTGCGCTTCAATCAGCACATTCCATACCTACCTATCGTTGTGTTGGTGCCGATGAGTTCACCGTCGGTAGAGCGTGTGAGAAGTATTGGATGGAAAGAAAAAAAAAAAA

AGGTGAACAGAAGAGAGCCAAGATTTATGACAATTGTTTCGGCTCAAACGTTCGCGTGTTGGGCTGAAAAGCAACTCTGCTAGCAGATCATGTGGATCCGTCGCAAGCAAATGGAGGTCGAAAACGTCGCAGAAACCAGTCAGTCTACCTTGCCAGCAACAAAGGCAGGTAAGGCAAGACAAGCAAGGTAGCCAAACCCCAGACTGTGTCACCCACCCACGAAATGCATATAACTAACGATCCCTGCACAGTATGGAGTACCTACTACGTAGGTTGATACACATTCCATGCCGCCCTTGTATGACAGTTGAGGAAGGCACGATTTCGCTCGCAATTATCTGGGAAAGTTCTAGCTATTCCATTCGACCTAGGGGGTTGCAGAAAGAGATCAATTCCTGCTGGGTTTTCCAGATCCTACCAAAGGTGGGTTACCGTATGTACCTAGGTACCTAGGTACGTACCACCTAGATCGTTATCTAGGTGCAGTATTTTGACTGCCCACCTACCTACCTACCTACCTACCTAGTTATGAAGACCTTACCAACGTATCTCTAGCGTGATTTGTAGGCCTTCTTGGTTCAGGCGGACTGGTCAACTTTAAATTTCTCGATAAAATCACGTCCGAAGTGCAAATGTGTATACAGAGTCAGCCGCCAGGCCACCAAGGAGGTATCCAAGGTACTGAGTGAGGTACGTGGGCAACTTGAGGAGCAGTAATACCCTTGGCTGTTAACGTCCTCTCCACGTCGTGGCTTACATTCCAGAACTGGTAGTAAGCTTTTTCTGATCTCGCCGACGTACTTCCCATTCACCACTTTGTAGGAGTGTAAGTATCGTAACATCGCCTCAAATCCACTTGAGCACAAAACACCCGGCTTCCGGCTTTGCCGGGTACCGACAGCTTATCTGAGTGGATACAAGGGTTTAAAAAAAGACCCCCGTAATTACGTCAAAGCTCAGTTGTGTATACCTACTCTTGCTACTGTAAGTATGTGTAGGTGTCTTGAGAATTCTAATAGAGGTTGACCCATTATGCAAAGTTTCCGCTTTCTTCCCTTTCTCTTACAATTCCCTTGCATTGAGGGGTAAAACCAGTCGCAGTCAACCTTGGCGCCATCTATAACCGAAGAAGACCAATTCCGTTCAAGTAATTTTTGTCACTTTCGGATGATCAAGTGTACGATTTGGCCGAAAACGACAGCAAATGCGACATGCCTGACCGAGCAAAAGAACAAATAAAAAAACGTCGCGCCACTTGTAGCACAACAGTTCAAAATGACAAATGGAGGAGCCGTTGTTGTCAAGGTTTGCTGGCTGCAATGTGGCTGCATTCAGCACAGGTCAAACCAATAACAGCCGTCCAGCACGCGCCAGAAACCCGGTGTGGACACTGAGAAGCCATTATAGCTGGGAACGAGAAGCAAACAATCAAGTGAGGAATCAAGAAAACCAGGCCATTTCTGCCTGTACCGCAAACCAACAAGTCTTGGCTGTGAGTTGTCTCAATTTATATGAAAATCAGGGGTTAACTTGTTTGGTTCACAATATCAGATTATAAATAACCAACGTCGTGAGTGATCAGCAGGTTGCTAAGCAACCAGTTCGGACTAACTGTGAGGGTAGCCTCAATGTCCAAATAGAGTGCTGACTGATATGTCCATCCGTCTTGAACCTAAAGTATCAGGCTCAAAGTAACGCTGCACATGGTGCCTTGCAAGCTTGGTGATTAGAATTATAGCCAGTACAATTGAGTTCGGTTCAAGCAAGCGAGCAACATGGGAATTTATTCGCCATAGTAGCCTTTACATAGTAGAGATATCGGTTACAGGGAATAGCTCCCAACTCAGCAACTCTTGGAGCGACAAATCCGGTATCTCATGTCGACGTCGATCAACCTTGG

**> MD506_chr05:3697895-3701895**

TCTATACTTTACGCCTACTCCCCATATTTCCGAAACTAATCCAGCACTATTTACAGTATCCGCCTTCCATAAATCGATCCGCGGCTCCGACCCAGACGCAGCGCTTTACTACCTCGCCCGCATGCTGCAGTCAGGCGAGGACCCGCTGTTCATCGCGCGGCGGCTCGTGGTGGTGGCTTCTGAGGACGTAGGCTTGGCGGACAACACGCTCCTGCCCCTGGCCACGGCGGCCTACACGGCAGCGCAGCAGATCGGGCTGCCCGAGGCGCGGATTCCGCTCGCGCACGCGACGGTGGCGCTGTGCCTGGCGCCCAAGAGCACGAGGGCGTACCGCGGCCTCAACAACGCCTTTACCGCGTTGAGGGAGCCGGGAGTCGCGGCGCTTCCCGTCCCGATACACCTGCGCAACGCGCCGACGAGGTTGATGAGGGAGATGGGCTACGGTGCAGAGTACAAGTATAATCCAAACTACAAGGATGGCAAGGTCAAGCAGCAGTATCTGCCCGACGACCTGGTCGGGAGGAGGTTCCTCGAGGAACGGGACCTCGGAACAGAGATTGATCCCGACCTGGGCGAGGATGGAGGGTGATTCGGCAAGTGTCCTCCTCGTACGATGTCCTGTGGTTTTGTTTACTGGTACAATGGCGGAACTGTTGTATTTTAGGATATGCACAGCGCGGCTTGGCTATGCGTCAACATAGACCCGGGCCGAAGTCCGGTGTATTTACAAGGGGCTCTCTCTTGTTGTATACATCTGTTTAATAAGAACCCGGTAGATTCAAAGGATTAAAACCCGATGAGCAGAAATATCTTGCCCGGTGGTAGGCATGAATAAGTCACATGTTGCAAGTCGCGGCCATCACGTGATTGTTCAAAAAGCTCGGGAGTTCCCACCAATAATTTTGCTTCTCTTCCCCGCCAAATATGAGGTGATCAATCTGCTCGGTATTCTCCATGCTCGACACACGGCAGTTGTTATCCGTTGGAACAAATACCTGGAACGGCCATTCAACATGTGCATTGCCACGAGAGACAACCAATGCCAAGCGAACGGAATTCGCATCAGTTTTTTGAGTTGGTTACACATCCGTGTTGTTCATGAATCGGGTACCTTAGCAGTGCCACTGTGATGCGGGCGCGAATTTGAGTTTGTCTTGGCATTAGATACTATGGAACGACTGACTGGCTGCGTTCGTCGTCAATCCAAGAAACATCAAAGGTATCAATCAGCGCCATGCAACTACGATTACCAAGTGAAAACAAGCCAGTCAAGCCAGTCTTGCTAGGTCTGGGCAATTGCTTTCTCGTCTTTGGCATGCTGTCCAAGCTGTCTCCAGTTCTAGACCAACTTGGCTCCCGAACGCCCAGTTTCGTGTAGGGTAAACATTTGGCGGATATTGACTTGGCAGCTGTGGTTGGTCCTAATCAACACACCGAAGTAGTGCTCTGTAAGAGGAGCTTTTTTCCCGTTCCAAGGCTTACTATGCGATCCGCAAAGAACTTGCTGACGACAGGCACGCCGAGCGTTGGCGCCGCTGAGCCAAGCAGCTTGGCTCATATACGCAGTCTAATGCCAAGACGACCCGCCTCGGAAGTGGATCAGGATGAGAAGGAAAACGGTAAAAGTGAGAAGCGCCTTTGAGACTGTTTCGAAGGAAAGGGACCGACTCGCGGTGAAAAAGGCAAAAAGGATTCCAGAGAAAAACATATAAGAAAACGGCACAAACGGCAAGAATTCGATGGTTCGAAAAGGGGAAACAAACAAACACCATGCTTATCGCTCGTTCGGCAAACTCGAAACTGAACATTGAATGAACCGATAGGAGCTAAAAACAATTGTAATGAGGCGCTCCGCATATGGAGCCCAGACTGGCCCGTAACACGCCCAGCCTCTTGGCTCGCCCGCAACCTCTTGGCAAGAACGCGCGGAACCAAAGCAAAGCATTGGCA

GCGACCACCGTGGTCAACAGCACCGGGGTCCAGCAAGGATGGAGTTACATGTCTTGGCATTTGGTCTTGGCGATGCAACCGCCATTTTTTTCTCGCAATTAACTGAAAGCCCAGGGCTCCGTCTTTCTGAAATGGGGTTAAAGAGAAAAAAAAAAAGGGCAGAGGTTCGTCGAAAGGCCCGCTCGGTAATTCTCATCATTAGTTTGAACCTGGCTAATTTTTTTC

TGTATGGGTTTGGAGGTTCGTTTGCCAAAACATCGCGAGCTAACGAAAGGAAAAGAAAAGATACTTTCAACAATCAAGGCATTACGGCACAGAAAAAAGGGAACTGGGATAATTGTCGCTCAATTGCGACAACAACTGCAACCAAACAACACGACGGCGGCTACTTCCGCGTCGTTGTCGGCAACCTCAGCAAAATCCACTGGCTGCTGGAGGCGTATCGAATTTGCTGGGTTGAATCGTGCGCAGTCCCGGTTGAGGTGCGCACGCTGAGTCGGACAGGAAACCGCCTGGCTAAGGGAAACCAGGCCACAACCACGATTTGAGAAACCAGGGATTCTGGAGGAACAGGGAAAGGGTCCGGCTGGCTTGGGCGCTGATATACTTGGTCACGGGTATCGCGCCAGAAGTCAGGGACGGTTGAAATCTGGAAACGACCAACCACCAGCACCAACCAGAAACAACAGCCACATACAACCGCAAAAATGCGGCAATCGTCAGCATCATCCCCATGGTCATGGGCATCACAACAATCACAACAAGCCCCCGGAAGGCCGCTGGCCAGGTTATTACCACTACTGCTACTGCAGCTGGTGCTGTTCACGACGGCGGCTCTCGGCTTCGACCCGGTCAAGGATGCCGAGTGCGACTCGGTCGACAGCATCTGCCTGACTTCCTTCAGGTGGTGCGCTCGCAATGGGCGCAGCTGCCACTACCCAGAGGGCGTCGACGCCTACATCCCGTGGTCGACCTCGAGTCCCTACGCGATGCTGTACCACGGCAACGTCTACGACGTCACGTGGAAGCAGGCCGTGCCTGGGGTGCCCGTCAAGATCGAGTGGTTCTTCTCGGAGAAGCTCTATACTACTGTCAATGACGGTTGGGGCACTCCCCAGGTCATGTGGAGTATGAGTAAGTGGATGAATCTGTGTGTTTTTTGTTAGCAGTGGGAATGCTGCCAGGCGCGTGGAGAGGCAGCGGATCCGGATGGAAGACGGCTATTGCACGCTGTCCAGACAAACGATAAACACAATGCTAATTCCACGTGCCCTACCCACAGACACCACAGACAGCTCATTCACGTTCGACCCGCTCGCCATCCTGATGAGCTTCCCGACGCAGTACAGCAACATCAGCTCGGGCGAGGCCATCGCGGCCACGCACGACGTGAGCAACTACCTGCGCATCAGCCAACCCAACGCCACATCCGACTCGCAGCGCGCGCCCGCGCTGACGGATCAGTTCCTCGTCCACACGGGCTGGACCGAGGAGTTTGTGCGCAACGTCCAGGCCTTTGACGCCAACCTGTACGGCCAGCAGCAGCGAAACTGGCAAATTGGCGTCGGCGCCGGCATCGGCATCGGCCTGCCGCTCATCGTCGTCCTGAGCTGGTTCATGGGCCACCGCCTCGGCACAAAGGCCGGGTACGAGCTCGCCGCAAAGGTGGCCAGGAGGAGGGAGGTCAGGGAGATGAGCTAGAAGCAAAGACACAACGACCGGGAGGGTGTTTTGCTGTCGAGCCTGTTAAGTTGCATTGTACAAACTGATGAAGCGTTGTTCTTTTTTTGTTATTTCCTGTAATGTTCTCGATGAACTCATGGGCTTGTTTTGCGCCGGCGTTTCGGATGTCTCTTGTTGGAGGGCATAACTCATGATTTGCGGCTGGGTGGATCTGCAGCCTTCATTTCCTAGATACCCTCATAGTAATGTTACCCACTCGTTTTATTTTCCACACATATACCGAGTGCCTCTTTGTATGTTGAAAAGGTACGTACGGGCTTCGGGACGTCGGGAGGTTTTCTCATCTAGGGCTAACCAGCCCCTAGCG

**> MD507_chr05:3771379-3775379**

GGGGTGTATGGCGCGGGCTGGTCGCCGTGGAGCTCTCCGTCGGCCGGGTAGTCATAGGCATCCTCCGGGGGAGGTAAGACTGGGACCGCTGATGCCAAGATGCTTGTCGAGATGGCGCCGGTTGTGGTCGGTGAAGGGCCGGTGTAGGGGCCATGGCCGGTCACGTTGGTGTGAGGCACCACGGGTGGGGTTATGGCCGCCTCCGAGACAACGGTCGTCTGAGCCTCGGAAACATAAGGCATGGCCAAAGAGCCGGAAGCCAAGGCGAGAAGCCCGGCATAGTGAAGTGTGCTTCTGGGAGCCATTATTTATTCGGTCCCTTGTCGAGGTCTCTTAGGGTCCGATGGATATGCTTCGAACTGGACTGCTGAGGCTTGCTTGGTTTTCTGGATAAACGAGCAGAGATGCATGCTTTATACCTTTTGTGTTCCTACCCCGACTTCTCTGGGTTTCCAAATCATGACAAACTATGCCAGATGCCGGATTTGCCGATGAGGCTCCGTTGATTGAAGCAATATGGTGCGGGTCATTGTGATTCCTGGTTGTATAGATTTACTTACCGCAAAAAGTCAGATTCAATTACAGGATCATGATCATCACCATTTCGCCATGACGGGTTAATTGGTTGGAGCCAAGCCTGGCATGCGGCTTCGGCGGTCGGCTCCATGTCTACACCGTCGACTTCCCCAGTTGTTGGGCGTCATGTCTCCGTCATCGGCTGGCATGATCTACCAAATGTGCGTGCCGGTAGGATATATGTAGCTGATTGATCCAGGCAAAGCCAGGGCAGCTAAAGCTCTGGTAACATCAAAAGACATGGTTATTGTTGAGCTCGCCGGGATAGGAAAGTCGGTCCGAGTGTGGTGGCAGTCAGGTAGGTAGTATTCTAGCTACCCATAGCTATACCCGATCGTCAGTTAGACGTCAACTTGACCAGATAGACTTTTACAGAGTAAGAGAGCTGTCCCTACGGTCGACCCAAAAGGGAGAGGCAACATGGGGACTTTCATTGGTGGATCTGACCGGGATACCGATGACGCTTACATTGGAAGGTACAGCTTTCTTTTTTTCTTTCTTTTTCAACGCGGGTATCTCACCGTGATGGACCATGAAAACCACGTTCCGACTCGATGTGCCGTTTTTTGATCGAGCAAACAAACACGTGGGGCGTGGTGATGGTGTGCGCAAGTGGTAACACGAACCCCAGCCTTTTTGCCTTGCGGATGTGAAGCTCCGGAATGATCCCGCATCCGTAGCGTACCCTAATTACCAGCAGCCCTTGCTAATTAGCGTTTTTTGTGCATACATACATATTTCTTGTTCCTGGATTTCTTTTTTACTGTAACCCAAATCTTTCTGTTCTCATCGATGAATGGTGGACCATTTATCCGAGCCAATGCTAAGATGGCATTGGATCAAATTTGCACCAGAACCAGGAAAATCCGCACCACCGTTGATAATGGTGGGTTTGCCACATACTACTATTGGGGTTGGTTCGTCTGAATAATACTTTTGTCATAAGAAAACCAGGTCCCCGAAAATAGTGTTGTTTTTTTTCTTGCTCTAATGCACGATCTGACCCGCCGCAACTTTATTAATCCAACTGCCGCATTTCCAGGTACGGGTTCCTCCCAAAAGGCCCTTTTTGGGCTGACCGTTGGGGGTCGGGTGCCACTCCACTATCAATCGACTTCGTCACAACTGCGTCTTCTGTCCGAGTCGATGACAATTACACACAAAGTCCACAGAATTCTCCGCACATCCTCCGCACAACCATGTCCAATAGGTAAATTTACAAAC

AGCAAATTGTAGATCCAGGGCGAGTCTAATACTGCGGCGAGAAAGCTCTCATCTGCATTCCAAGAAAGAAATCGGTTTTGACCCATCTACTGTCGATATACGGTTGTTCCACAAAGATCAAGCAGATTTTCTACCACCGATATTATTCGATTCGATCCCCAAGAGTGTGGCTTTTGTCCAGATCGGCCATTTTTCTTATTTCTCTCTCTCTCTCTCTCTCTCTCTCTCTCTCTCTCTCTCTCTCTCTCTCTCTCTCTCGTATCACGCTGCAATATCCGTGCCGACTAACCGATCACAGAC

ATGTCTCAACCTGGAATAGGCCGTTTATTCTCGGCTGCAAGCAGTACAAGTTTCACATCGTTTACTTAATTCTAGATAAAATGCCGCGAGTACATGTTAGGTACCCCTGACTGGCAACTTCTATGCCCTCCTGGCCTTGCCTCAGATGAGCTCCATCTTGATACCACGCTTGCCGGTCCTCTCATCCTTCTCATCCGTGATCCTGACCTTGACCTTTTGGAATAGCTCTGCCGTTATCTCGCGGCTTCCAGAGGTCCGCAGGACGTAGTTGTCAGCATCAAACTCCGCCTCAGGTTCGGGCTCCGCAAGGTCTCGCAGCCTGATTAGGCCCTCGACTCCGAACCGCGGCACGTAGACTACGAAGCCGTTGCTGAAGATCTTCATGATGAAACCATCCTCTTCGGCCACCTTGCCCTTGAGTGACTGTCCCACATAGTAGGCTACGCTGGCACGCCCGGCGAGCTGAGCGTTGCGGTGCCTGACGTTGATGTTGCGGCAAACAGCCTCCAGCCTGCCCCGGCTACGTACGCTTGGATGGACCGCCTCGTAGCCAATTGCGGCCGCTAGCTGTCTGTGCGCTACAAGGTCTGCATATCGGCGAATCGGGGACGTGAAGTGGGTGTATATCTCGGAAGCAAGACCGTAGTGGCGGAACTCGGGATAAGCTTGGGTTCCGGAGCAAAAGTACTCGGCCGACATCATGCAACGAGTCGCCATGATACGAACCAATGTGTTGAAGAATGGGTTGTTGGGGTCTACACATGTGTCCAGGCTGTCCGCAAGTGCGCGAGAAGAGTCGACCCGCAGCTCGAAACCCTTTTTGACCTTGAGCTGGTTGGCAAGTTCGTCAAAGTTGGTCTTGGGCGGCGCGCCGTGTCTTCGGAGGATAGCCGTTTGGGGAGAGGCTTCGTAGATCTTTCGTGCAACGCTAATGTTGGCGAGCAACATGAACTCTTCGACCAGTGACATGGTCTCGAGATGGACCTTGGTCTTGATGTCGATGGGATCAGAAGTTTCCGACTCCATCTGAACCTTGACTTCCGGCGAGGACAGACTGAGAGCACCGGCATCCATACGCTTCTGCTTGAGCTTCTTGGAAAGAGCAAGTAGAGTGCGTATGCCTGTGGTGAGCTCGTCCTGCTGCGATGCATCATCCACCCTGATCTGTGCTGCCTCGTAGCTGAATGCCTCTCGTGACTTGATGACTGACTTTGTGAAGCGCGCATTGACAATGTCGGCGTCAGGTGTCAGCTCCCAAAGTACCGAGAAAGCAAACCGCTCAACATAAGGCTTCAGAGAGCACAGGTCTGTGCCCAGGAGCATGGGCAACATGTCGATACGCTTGTCCACCAGATAGACCGTTGTGCCGCGAATGCTGGCCTCGGTGTCCATTGCGTTACTCGGCTTGACGAAATGTGACACATCAGCAATGTGGACGCCGACCTCAAAGTTGCCGTTAGGTAATGGCCTGGCATGCAGAGCATCGTCGATATCTTGACAGCCGATCGGGTCGATACTGCAGATGAGGAGGCCTCGTAAATCCTCGCGGTCCCTCCAGCCCGGGTCATCTGTGCTGGCTGGGACCTTCCAGTCATGGCCCTCCTTAGGTAGACAGTCTAGCACAGTCTTGGGAAACGGCCTGTACTGGACGTCGTACTCGAGCAGCAAGGCTTCCGTCTCTGCAGCTTTTGTCTCTAGCTCTCCCAAAGACCGGACAAAGTGACCGACTGGGTGCCGAGAGTCCCTGTCCCATGCATCAATCGTCACGAGAATGCGCTTTCCCAGTAGCTCGCCAACCTGTCTTGTGCGAAGTCTAATTTTGGGGATCTTCTTGTCCATCGGAATCAAGAATACCACCTCCTGCTTCCTTCCTTGTGTAGCAGATTTGCTGACGGAGCTC

**> MD508_chr05:3809891-3813891**

CGCACTTCCTTTTTTTTCTATGAATTTTTATACTGTATGTACTTCTTCGCTGATGGTGTACACAACTATCCAACCAGACAAAAGCGCGCGTAACTCACTTTGTTTGTACTTGGTTAGTGATTTTCACCTAAGCAATCTGTAGCAACTTTGCGCCGTGATGCTGTCATCCGCGGTGGGTGGCCAGGGGGAGCCTGCACCCGAACGGGCTGCCCACATCCCCCCAAATGGTTCATCGGCCGTACGACCGTACCGTGGTTCATCCGGGTGTCATTCGTCCCGACTCGCGTCAAAGGGTCCAGAGTATATTATATCACCGTCATTATAGACTGGGATGACACGTCCCGTCGCTTGAAGCGGATGTCGACCGTTGGACGGGCCTCGGGACTCCTCCGAATGGGGCCAAGCGGCAGATGGGACGATAACTGAACCTTTTTTGGGGGCTGCTTTTTTGTTTTGTTTGATTTTGTTTCGATTTGCGATGTCATGAATCTGGGTGGTATTCCGAGTATCTTGGTAAATCTCAAAACTTTGCCGGTGAATTTGGTGTGATTAGGTCTGTGTCAACGTTGTTGTTAAAGGTAGGGGTTTTGACTTTTGGAAAGAAAAGAAAAGAAAGTCGGCCCCATAAAATACAGGCAGCACGTTTAACAAAAGCGTCCACACATGAGCACGCAAGCAAACGACCGCATGTACTCTGTACCGCGAAAGTACTATACTGTCTGAATACCCTCGAGGCAACCCTTGCGGGAGGTGGTTGGAAGGAATGCCCGAGAGACAGACCTGAGGCGAAGCGGGACCCAATGCTACACAGCCCACACTCACGACTCTGCCTTTGCAGATGGGACCCATCAATGCGCGGGGCTCGCAAGGCAGCCCAAGCCCGCCAGTCCACCTGTGGCTAGTGCGAATATGATAGGGATGGAGGGGGGAAACTAAAAGAAAAAGAGAAAGAAAAAAAAAGTCATCACAGTTTACCGCGCAAAGATACTTCGTACATCCCCATAAATAGCCCTTTTTTTCTACCCCATGCAAAATGCCGTGTAACAGCAACGAGGATGTTGTACGAGATGAGGGAACGTAGGACAAAGTATAAGCACAACAATTCTAGGATCGGCCCACTGCTTGTTCGTCTTACTGGAATTAGTCTAATTACTGTGCACTACTCTGTCAGCAGTAAGAAAGCAACAGGCAGAGAGCAGTAAAATACTCGGCGACCAATCATGCTGGATGCGGGTTGTCGAATATTCCATTAGTTGTGTTACCATCCTGCCGTGTGTGGTACCTACATATGCGTCTCCATATGCATCCCCATTATCAAGCGATTGGACGGTGCTTTTCTACAGGCCTTTATAACAGATGACAAAGCTGCCTGCCGGTCGGCGCATGAAATGCCCCCATCAGGAGCACGTCCTGCGCTTTGGGCCCTGGACTAAGGTAGGTAAGGTACGAAGTAGGTACCTTGTTAGTAAACACTTGTAGGCTGGCAAAGTACAACGTGGTAGTTGTTGGCTACGGGGAAAGGGTGCAAAATGCCGAGTCGGCGTTCCCTTGCCCACATGGCGGAGAAGTCTTAAAACTGAAAGAACGTCCGGATGCTGCGCTGCACACAGTAGCTTTGACAGACCGGCAGAGTGGTAAACCAGAATTACGGGACTTCATCTACAAGTAAACCTTCGGCGACATGATAGGCGGTACGCACGCCTGGCATCAACCAAAGCACGGCGC

TGGGTCCGGTCCCAGGGCCGGCAGTTACTTCTAGCGTCTCGTGGCCAAGTTACATGGTCGTCTTGTCCTGCAAAGAGACGCCATAATCTCGGGGCCAAGCTTGGTGATTGCGAATCAAACGGACGGATAAGCGAGATGGGCCCCCCTTCAAGTTGCATATGCGGGTGGTCTGAGCCAGCGGGAGGGATGAGAGGTTCCACAGCCAGGGAAGGCTGGCAGGGCGGGCAAGAGAAAGGTCAGCCGCTGCCGTCCAAGAGGCGGAGGGAAGGGAAGAGAAAAAAAAAAACGAAAAAAAAAAAC

GAAAAAAAAAAAAAAAAAAAAAAACGAAGACTAATGCGGCTGACGGGTAGGCGACCGGGAGGATTGTACTTGGCTTGTCTGAAATTGCGATGATGATGATGATGATGATGATGATGATGATACTGGGGATGATGAATTACGATGCGCATCGTGTGGTCAATGCTGACGAAAAAGGGATGTCGTATGCCATACAGTGGCTGTATCTCATTATTGTTATTTTCCTCTTGGGCGGCTATGATAATAATCACGACGATAATGCAAAGAGCGGCGCCTCGCACGGCAGGACATGAAAACGAGGGCCGTGCGGTATGACGACTCCTGCGCTCTCGGCAGATCTCCCCGGAAGTGTATTGTAGATTGATGAACTGCGATATTACCTACCGGCTTGGGGCCCTCGTGTAGTTGCGGCGTGCCGAATGATTGATGCACATTTGGTCCCCGCTGACTTCATGCCCCCAGAACAAAGTTGGGCGATCAACATAGGGCCCCTATTAACGAAAACGCAAGTAACCCGTGTTTTGATCCCTTGAATTTGTTGACCGAATCGGTTTGACTTAGTTCCATGATCCCATCAATCTCCAAAAGCCCGGACGGCAGACAAGCAGAGATAAGCAAGGGCAAGGATGCTGCGGTCACGGTACCTTTATGGTTTTGGTACCACACGGGGAGGTAATCTTGCCGGTGAAGCAAACAATCCATGGCAAGACAAGGTACCACCAAGAAAATGGTTACTACCATGGGAAGGGTTGCGGCCTGTCGGGGCGAAGACGACGACAACAACAACAACAACAAAAACAACAAAAAAGAAGACAAAGCACGGACGGGGAGCCAGTAAAGTGTCGTGATCCGGGATCCCTGTCTCCATCCAATACCCACCTACTATATACGTAGGGGGTCCACTGACTGCGGGTGGCTTCACAGTACTAACTAAATTACATACCTGCCGAACACTGTATCTAAGCTCCTTCCAATTCCGTGATAGCCTCTTGCTTTGCGCGCCCGACTTGCCGTTCCTACTGCGGCAGAAGGTGTGAAGCATGGATACAGTACGTCTTGGGTGCAACCAATTTGGAAGGCACCTGCGTATTGTACTACAGGTAGCTACGGTACCTACAGTATACCTTCCAGTTTCCGCAAATTGACAACAACGCCTTCTCCAAGCCGTCCCTTTTTTTCCCCTTCTGTTCGGTGTGGAGAGATTGATGCAAGGACGACATTTTGAAAGTAGTGGCCGTCTCCGCCTCCTGTAGAGTGGCCTGCCCAGTACCAACACTCATTACAAGCCGAACCTCTACAATCGGCCCATTCCACCCGTCTGTCGACATATTCCAGAGAGTCGGGATTACTTAGTTCGAGTGGCTGCTCTTCTACCCGCTGCGGATCTAAGGCGCTCAGCCAAAAAAGATGCAAAAGCAAAGCGACCAGGCACAAACATGCAAACATGACAGTTGGCTGATTGACTTGCATGATCAGGGTTACCGGCCTGCCCTCGTGACATTTTGTTAATCAGGTACACAATCAAGTTGGGTCGCGGACATTTGCAGCTTATGCTGAACTGTACCGCCTTAGTTCTCGAGCTTAGCCTGGCAGGTCTCTCTCCATGTACCAACTTTAGTCTAGTACCGTTAACGGGTGTGTTTGTGCCTGGAACTTGTCGCGGGCTAATGCTAACAGGCACACCCCGGACTCATGTTGACCTCGAAGCACATCAACGGGTGGCCGCTTTAATGCTAGCCAGGGACGGTCTGCAGTACCTTTCGTGATGATCAGCCCAGTTTCCCGGCTGGCTTCATTGTTGTGACAGACCAAGACATCGTTCTCTACGAACTTGCAACATCCCTATACACGGACCCCCAAGGCCAGAAAAAGACTTTTTGATTTTAGTTCTGGAGATAAGCTCACAACGAGAGAGGGGCTTTTCTTTTTATCACTTTTTCATCCGTCAGCATCAACCGTACCCCAATGCGCTTACTCC

**> MD509_chr05:3887001-3891001**

ACTCCCTCGCCAGCCTGTGGAGGGGTGTGATCAACTTGCACTCGCGGTACATCATCACGTCCCCTTACTGCCACAGCTTCCAGCCACCCGCCGAGTCCGGGATCCCACCGACGCGCAACGGGGCCTTTGAGCACAACAACCCGCAGCCGGGCTACGACCCCCGGCTTGTGTACGACTGCAAGTGGGAGCTGGACTCGCTGGCGTCGTTCCTACAGATCTCGGTGGGCTACCACCAGCGCACGGGCGACCTGGCCTTCTTTGGCCGGTACCAGTGGGCCCAGGCCGTGCGGGCCGCGGTCGACGCCGCCGCCGCCATGACGCTGGGGACGTACGCTGCCGACGGCAAGACGGAGAAGTCGGCGTGGACCTTTATGGGCTGGACGAACCGCGGCACCGAGACTCTGACCAACGACGGGATGGGCAACCCGACGCGGGCCAACGGCATGGTGCGCACCGCGTTCCGGCCCAGCGACGACGCCACCATCTTCCAGCTCCTGGTCCCGGCAAACATGATGTTTGCAAAGTACCTCGAGGAGGCCTCGCTCATCCCCGAGGCGCTGTCGTCGACCACCGACCGCGCCGCCGCCGCCGGCACAAAGCGGGACGAGGGGCTGGTGCGGCTTGCGGCCGACATGCGGGCGCTGGCAAAGGGGATCCGTGAGGGCATCGCGCGGGACGCCGTGGTGAGGCACCGCCGGTTCGGCGACATGTTCGCCTACGAAGTGGACGGGTTCGGCGGGGCAAACCTCATGGACGACGCCAACGTGCCGTCGCTGCTGGCCATGCCGCTGTGGAACTTTTCCCAAAGGGGCTGGGGCGGCGGCGGCGGCGGCGGCAGCGTCGTCGGGGCCGCCAACGCGACGTGGGATCCGCTGGCGGAGGAGCTCGCGATCAACAGGATCTACCTGAACACGCGGCGGTTCGTGCTCAGCGATGCGAACCCGTACTACGCCAAGGGACCCGTGATCTCGGCGGTCGGGGGCCCGCATATCGGGCCCGGTAGGGCTTGGCCCATGGCGGCCATCGTGGCGGGCATGACGGCCTACGAGTAGTAGTAGTAGTAGTAGTAGTATTATTTAACGCCGGGCTCGGCCCGGCTCATTAGCCGGCCGTTAGCAACCTCCCGAGGGGTATCTCGGCGCGCTTTCTATTTTCTCTATTTACACAGCGGAAAACAAGGGCATAGAAGCGAATCGAGCCTGACCGGGTTCGTGCCCGGTCCTGCTTACAGGTTTGTTCACTGACGCGACCCCGTGCCTTCAGGCGCACGGAGGATTCGTCTGACGGCAGTTGACGGCTCTTCATCGAGAGGGGCCTGTGTCCAAACAGCGGAGCTGTCGGTCGTTTGTAGCCATGGAGACGAAGTTCCCAACAAGTGTGGGCTCGACGGCACAGGCGGGTGGTTGTTGTCGATAGCCAGCCGGGTTATCCTTGCCACGGGTAAGCGGGGAGGAGGTGGAGGGAGCAGGATTCGAACCTACGTTACTCAAGTAACAGCCATGGCGCTTAACCACTGCGCCATTCCCCCCGTGACGGCCTACGACGTGGCGGCGATGAGGAGGTTGGGCTTTTGGAATGCTACGGGGATCAGTAGGAAAACCAAGGATGATGAGGTGGAGGCGTGGCTGCAGGACGAGGTGGCGGGGCAGCTGCGGATGGTGCTCGACTCAACGAGCGGCACGGGAGTGGTTCACGAGAGCGTCAATTCGTGGGACGAGGGCCTGTGGACGAGGTCCTGGTTCGGATGGGCGAGCGGGCTGCTTGGAGAGTTGATTCTGAGGATAGAGGGGTATGAGAAGCGATTACCGCCGAACGGGAAGGGGCTGTTGTCTAGAAGTTGGCAGTGATCAGGCCACATGTCTCATGGGTCTTTGTTGTTTGTTTTTGATTCCGACATATCAAATGTGTAAACCAAATAGGGTAGAACCGGCCGAGAGATTCGGAGAAATG

TCGAAAACATGGACAGCTTGATTTCGGGGGATGATGAATGGCTGTGCTGGTCGTGGGCAGATCGTCGGCAGATGGACTAAGATCAATCGGGCGGAGATGGGGAATGGGACACCACACACCACTGGAGAATCGGGGCTGAGGCGCAATCAT

AATAGGCAACACTGGAACTGTGCCGCAGTCTCCAAGAAGCACCTCAAAGTTGAAGTGCGCGAAGCCACGATGAAAACGTATGCACCTCTAGTAAAGTACCTACCTACCTAGGTAGGTAAGTATGAACTCTTTTGATTTCGCATTGTTCGTAGAGGCTTGATTATTCATGTAACAAATAGCCAGGGCGTAAGTTTGGGTTAGTTTGGGGGACAACTAAAAAAGAAAAACCACGTAAATTCAGCAACACTCGATTTGAGCCACTCTTTGGCTTCAAGTCCAATCATCGAGAAATCCAAGAGTGCGTAGCAAAATCTTGGTTAAAATAGCCAAATATATTTTTACATATGTTGGGTTCAAATCATACGTTCTGAGCGGCACTTATTGACGCCGGTCCCCACTGTTTCCAGGCGCCTTAGTTTCAATGTCGCCCTTCTCCAGCGACTGTTGCTCCCCAAAGACTGTATCCACCAAGCCACCCATTTCCTCCCGAGCCATAGAATCCAATCCTCGCTTCCTCACCTGCACGATCCCAAACTTGGCCCATCGCACTATTCCTGCCTGCTTTTGAGGCGCCGTGTGAATGTCTCCGACTCTACCTCCACCACGCCGCTCAGACTAAAAGGCACTGCTCTTTTTTTTTTCTTCTCGCAAGTAATGTCAGCAATCGATCGCCCACCTAGTCCAGTAAGCTTGGCATTGCTCTGCTCTCCAGTGAAGTACGTGGTGGCTTGATCCAAAAGCCATAGGCTCAGCTGATATCTTCCGACCCAAACGTCAGAAAGAGTGGGAGCAGCGGGGACAGCGCCTTGATCTCGTCGATGAGATAGATGCACCCAAAAGCTTGGTTCTGGTTGTTTATGGTTTGCCATGTAATTACCAAAACATTCGATCCATGGTGTTTCCACGTGGGCGCTTACATGCGAAAGAAAGTAAGCTGGGCTTTGCTGGGGTTTTGTCTGGGTCCAGTTTACGAGCGGGAGAGCCCAGTGCACCATTCAGTGGGACGGTGAACAAGCGCCCCATTTGTACCCCCTTGGCGTGTCTCTAAGTAGAGATACAGCAGCCAACGGTTTCGATAATCGAGTCTCCGTGCCACCAAACCAACAATCAGTGAGTCCATTTTGCGACAATGCCACGGAAATGCGTCGAGGGGCAATTGGATCAGGTTGGGGTTTGTTTGTCGCCTTATTCCAAGGCTTCGACGTCCCGAGGACGAGTGACAAGTCAAGGTGACTCTAGGCCAGGCAATGCAATGCAATACACATACCCTCACTCACCCTCCTGCTTTTGCTCGTCTTACTATGCCACATAAATTCACAACCTGCACACTGCCACAGTCCCTTGCCTACCATAGGACAGCTCGCATGCTTGGTTCCTATTGAGATGGCATATTAGCTACCTACCCATGTGTCTTTTCGGTGCCGAACACCCTCTCGGTCTTGGTGGGATAAAGCCGCATTACATCTTACTATTGTTGCAACACCCATTTCGATTCGAAGTAAGACCTTCGCCGCCCTGTCCATCTACTTTAAGAGTGTGCATATCCCCGCACAGTCTTGAATGATCGAGGATATAGCATCCATCTCCCCAACAACAACAACAACAACAACAACAAGACAAGATTCTCAACATCGCCTTACATACACACGCTTGCTATACAACCGAAAGCATGCATATCAGCTTAGCTATCCTGACAGCGGCTGGACTCGCCAGCGCCGGCCAGTACGTCGAAAAGGACTACTACCACCGCAAGAATTTCTTCGACGCCTTCGACTTCGTCTCGATCGACGACCCGACCAAAGGCAAGGTCAACTACCTGGACAAGAGGCAGGCCAACGACAAGAAGCTGGCCGGGTTCCCCAAGGGCGAGGACGCCATCTACCTGGCCGTGGACGACAAG

**> MD510_chr05:3966102-3970102**

GAATCATCTTGGGCCGGCTACCCTTACCTGATCCGCCTGAATGGGAATGGAATCTCTGGTGGGTTGACAAGGTCTTTAAGACCCAATTCGGCAACCATCCGGAGATCAAAACGCGGGCTTCCCGAAGTGGCCGTGGTCAGAGACCTTTGTTCCCTTTTTTCGATATACAAGGCCATTCCGTAAAAAGATCCAAGGTTCTCTAATTCATGTAGGCATGCGTCAGGGGCGGAATGCCCGTCATCCTGGGCTGCGTCTGGATGCGCGAGGCTTAAGCGCCGGCTTTGAGATGGGGTTCTTTTCGGGGGCCAACCATACATCACCCTTCCATCTGTTGAGAATTGAATTGGCCCTATTCGCTTTGAGCGATGATGATCATCGCCACGAAACTGTGCCTCTTGAGCTGAGCAGTAGTCAGTAGACTTGGCGTCAGGATGCGGCGTTGGGCGCGGTTGTCGCGGTAGGCAGACAAAAGTATGCAGCCATGCGAGAGACCTCCTCTCAGCCCTGAATTGAGTGAATTTAACGGATCTCGATTACTTTCAAAAGAGGTCCCGCGATTGCCTACCTAGGAGCGACTATGGCGGGCCATCGCATTAAGCCATATAACCATGCCAGAGTCTATATATAGGCAGATCTATTTTCCAAATCCGAATACGTAGTAATTACACACGTATGCTCCGCTCCACTGAGATACCTATGTATTCCCTTCCGTCCGGGATATGATTGATGCACTCAAACAAACGTCATGCTTCAGCCATTGCGATTCGCATACTAATTACAGCAGAACTAGGTACTGTCTGCTCTCGGGATGCAGAGGCTTTTGGAGTCCCGACTATGGAATTTGCACTGTAGAAATTGCAAGTTTGTTGCATCGGAATAAGCAAGCCACCATGCAACTACTCTGCTAGTCGTGTCTGAGAACAACTCCTCCTCGGATCGCATAATGGTTCGTGTTTTCTTTGGTTTGCGCTCTTCAATGGTTTCCGGGCTGACAGGCTTCATTGCTAGGTAGACAGCAACGCAAAAGCCTAGATAGGTTAGCAGGTCGCTATTGTCCTGTGGTTGTGTGGTTGTGTGGTTACACAATCGAGTGCTGGTAGAGACTGGCTCGCTGGTAGAGACGCCTTTCGCCCAATCCAGCTAGCCACCTGAAGCTACTTTTGCGTGCTACCAACATGGTGGGAAGTTGACGTATGTTTTTGTCTTAGTTTGATCGAGCACAAATAGTGTCACACAGTTCCGGATGGGCAGATGCCAGCACCGAGGACACGGTGATTAATTTAGCGTAGAATACTACTATCTAGACCCAGAACCAATCCTTGTAGGTGCTCTTGGTATATGAGAAACACGTTGATCGAATTTTGGGGTTGCTGCCGTTGATTGTACCAGATCGGCGTCTTTCCAATTTTCAAACACTCACCTGACACATTTCACTGTTTACACAACACACGGCTCGCAATCACATCGAGTGTTGGTTGCAGACCCCCGGCTGGTAGGTAATAAATAGTTACGATATAAGGCAGGCTATGCTCGCTCGCTCACGCAAAGTAAAACCTCGGCAACTACCTGTTCGAGGTGTTGTTCCCGCCAATATTTACTCTGCGTCGCAAAGAGAGTGGGTATCACTTATCAACAATATGACAAAAACAGCTGTGCGCGACATGCATTGCATTTTTTGTAGCCTGTGGGATTCTTGACGATTAGATGCAAGTCTCAATGTTTAGATGGAAATGGGTTCCCCGTCCTAACCTGTCAACGACATACGGTACATCTTCAACCGAGGCTCAATGGGCGGGCAGTCTGTGGTTTTTATTTCTCCGTCGCCAACCAGCCAGCCTTCGCCCCCAACAAACAAACAGCCAGCCAGCCTTCTGG

TCCAAAATTCGGGGAATCGCGTTGACCTCCAATTGCCCCGCATGCTTGTCAGTCCCTAGTGGGGCGACAGGAGTTCAGACGACGTCCTACTCCAACCACATTTCATCATTCCGGGCAGCCGCAAACGCAAGGCACGAGACACTCGTCAAT

CCCTGAACCAGACGCAAGACCCAGCCAGACCCAGATTCAGACAAGCCAGACCCTATTGCAGTTGCTATTACTGTACGTACTCCATTTGGTAGGACCCTCCTGCCCTTGACCGCTGCATTCAATATCGCAAAAAAAGAGACTGTAAGAAAAAAAGAAAAAAAGAAAAAGAGAGAGAGAAAAAAAACAGAAACCGAACGGCCGAGTCGGGTACCTATTTTTGGTGGGTCTACTTTTTGTGGCCTAGGTTGTATTTGAACCTCCACAGTCCATCGTCTCCAAGTCAGAGTGCCAGTCACTTCTGCCTACCTGGTTTGCGGGAGCCAGCAGGAGAGTCTTGTCTGCTGTGTTTGCTTCGAGCGAAAGTACCACCTGTCCGGCCTGCTGGGACTCAACTCTGCTGTGTTCCGGCAGCAGCTTCCCGACACGGGATAGCATCCTGCCCAGCGGTTTTTGGATCTCGGCAAGGCGCATGCCTGCCTTATCTAGGTAGCAAACGCCTACTCATAAGACGCCCGGTTTTATACTTCCCTCTCCGCCCAAGGGTCTTTTTGCCCGCACGCTCGCCGCCTTACTCGTGGACTCTTCACGCACAAATACATACCATGACGAGCTCTTCCTCCTCGCCCCCACCTACGAGCTTCCTGGAGCAGACCGTGTCGGTCATCTCCAGCGTAGCGGGCTACATGCGGCTTCCGGCCATGGCCTCCACGGGCGTCGCCGCCGTGCTGACATCCCTGCTGTATTTCAAGCAAAAGTGAGCAAAAACAAAACAACCAGACCCGAGATAGGTGATGATGGAAGACTGGCCCTTCCCACTAGAGTACTGATATAGTCCCCGCCGTGGGGTTCGCCATCACAGATCCTTGATATACTCCTCCAACATGCCGCCGAATTCTCGAACCCAAGTCTCACGGCCCAGCCAATTCAACATTAAAGATTTCGAAGAGCTCATGATCCCGACGCCCGACGGCGAGAAACTTTCGGCCTTTTACATCCGCGGTTCTCGAAACGGACGCAACTCGAACGTCACCATTATCATGTTCCACGGCAATGCGGGCAACATTGGCCATAGACTACCCATCGCAAGACATTTGGTGGAGCTTATGGGCTGCAACGTTTTCATGCTCGAGTACCGCGGCTACGGCTTGTCAACCGGCACTGCGGACGAGTCGGGGCTTATGATTGATGCTCAGACCGGGCTGGACTACCTCAGGGATCGTCCCGAGACGAGGAAGCACCGGCTGGTGGTTTACGGGCAAAGCCTGGGTGGTTCCGTGGCCATTCGGCTGGTGAGCAAGAACCAGGCTGCGGGGGATATTGTTGGTCTGATTCTGGAAAACACATTCCTCAGCATGCGCAAACTCATCCCCTCTGTCATCCCGCCGACCAAGTATTTTGCCTTCCTCTGCCACCAGGTTTGGCCTTCCGATGTAGCGATACCAAACATCACCAAGGTGCCCATCCTGTTTCTGAGTGGACAGCAGGATGAGATTGTTCCGTGAGTGCGGCCTGCTAGCGATATCTATTTCAACCACCACCTTGATTTTTATGCTAATTCTTGTTACGAATCACACAGTCCATCGCACATGCGCCAGCTATATGAGCTCTCTGCGGCCCCGAATAAGATATGGAAACCATTACCCAACGGGGACCACAACTCGAGCGTCTTGGAGGACGGGTATTTTGACGCCATCTCGGACTTTGTTGCCTCGGTTGCATCGGAACCACCCCGTCTGAGTGAGAAGATATGACAGCGCATTTCCAATTGATTACGTCTACAACCCGGACCGAAACGTCTGCAGAGTCTTTTGGCCTCATTGGTGTTCAAACTTGCGAAAATCGACCCCTCCCCGGCCCATGATACAACTTGGATCGGTTTGGTACCATTGTCAAAGGATTTTCCGGTTTGCCTCCTCACAAATAATAAGAGATTTGGTCTTTACAAGAAATAGATTGATTGCAAAGGCCCGTCGGC

**> MD511_chr05:4002261-4006261**

CTCTGCATACTATGGCACATGCATTCGTTACATTGTACCTGAAAGATTTGTGGATCGAAACTGCTCCCTTGGATGGACCACTCTTTTACTGCGTAGTCTTGAGCCCCGCGATCATTAGATTCAAGGTCCGTGACATAGATCGACTGCGTGTCATCCGTTGATGAGCTTTCTCCTACCCACCCCCCTCGGAGAAGCGAATCCGGTTCACTTCTGGACGAAATAGCAATGTCACTTTTTTAATTTCTCCAATCCCATATAAAACTGTCTTTTTTTTTTTTTTTTTTTTGTCTGGTTTTTCCGCCCCTGAAAAGTTTCAGCGACAGTTTCAGTGCAGTGTTGGACCGAATATACATAAAATACTAAGATACATGGAAAGGAAACGTTGGATGCCACGACGTCGAGGTAAAATATGCCCAAACTTTATTGTTGTTGCCCGCCCCAAATCCCCCATAGCGCCTTGGAAAACTTTGTATTGTCGACTTTTAGACACAACCCAAGATTGGGATTGCCGTGGCTGCCCCCGTTGATGCGAGTTGAAGGCGGTTAGGGTTTCTTGCAACGTATTAGCAGGTATCTTAGTGTTGTGCGGGTGTAATTAAACAAGGACGGTGCCATTGACGAGGATATTCCTGGGACTTTGCCGGTGTATTTTGGTAGTAAAAGTTACAGAGGGTTCCGTCTCTTGTCAGTGTTGCTGTATATAGCTCAGGTTCGTACGTCAAGATGTGGTATTTATTCATCGGTTCAAAGGCTTATGTTTGGTGTGTGTGTGTGTGTGTGTGTGTGTGTGTGTGTGTGTGTGTGTGTAGATATCCATATTTATACTAAGTTGTTCTAGAACTATTTCTAGGTCTAGTTCTAGTCTAATTAGTATGTTGGTTTTGTCTCAATTCAATGATTACCAAGCATCAATGTGAGTCAAAATTCACTAGCAAGCTGATAAATCTGCTGGTTTTTGTCAACCCATAAAATGCGAATAATAATATGCGATCATTCCAAATTAGAGAGAAGTCACATACAAGTTTGTTTCAACATTTCCCAACACAGTCCAAGGTTTACGCGCATGTCCTTGAATAATTAATTATACACCACACTCCATTGTTGCATCAACGCTATTTCGGCACGAGCATCATCCTAAAAGCTTCAATTAAGCGATATTACTGGCGATATAGGACGCCATATATCGGTGCAACAAATACCACCTGATCATCGAAACCCCTCCAATCCCTAAACCCAACCAGCCGCGCATCAGAAAAAAGACTGCCCCCCATATTAGCCCGGCCGTCCGTGGGATATCGGCTGCAACGATGCGCCCGGCGGTGAGCACCCCAACGGCGATACTCCTCGTAGACATCCAAGAGGGGTTCAAGCACCCCACCCACTGGGGCGAGTCCAGGAGCACTCCTTCCTTCGAAAGCAACGTCGAGGCGATCCTGTCCGCGGCGAGGGCCTACAATACTCGCTTGGAAACCAACCATGCAACAGCAACAGACCCAGCACCAGCACCAGCAGACGCAGGTAGCACTCCCAGTCAGCACGCGGTCCAGATAATCCACATCCACCACCACTCCCTATCCCCGACGTCGGCGCTGCACCCAACCCACCGCATCGCCGGCTCGTCGACGCCGTCCGTGGCCGCCAACCCCTGCGCTGCACCGCGGCCCCACGAAGCAGTGCTCGTCAAGCACCACAACAGCTCCTTTGTGGGCACGGACTTGGAGGCGCGCCTGCGGGCCGCGGGCGTGCGGCAGCTGGTAGTCCTCGGCCTGACCACCGACCACTGCGTCAGCACGACGGTGCGCTTCGCCGCCAACCTGCAGGTGCTGGGCGGCGAGGGCGGCCCCGACGGCAACGGGGAGGGCGTGCACGGCGTCGTGGTCGTGCGCGACGCCACGGCCACGTTTGCCAGGGGAGGCTTCGACGCCGAGACGGTCCATGCCGTCAGCCTGG

CCAGCTTGGATGGTGAGTTTGCCCAGGTCGTGGAGACCAAGGACGTCTTGGGGGCACTTGGGGCACTTGGGGCACTTGGGGCGTGAGGTGGGTGAGCAGGGAAGAAGGGGGGATGGAGGGGGATCTGGTACATAGTAAACTCTGGTCGTA

TAGGGGGTACATGAAAAGCAGGAGGTAGGGGCAGCGGGGATCACTACAAAAGATCCAGGTGTGGTATTCTACAGGGTCATGGGAAAGATACCATTAGAGCAGGCGATGCATGGGCTTGAAGCCCTGGACATGCACATCGATCGTGCAAGTCATACAACCCAATTTTTGCTACTTATGTACTTTCTTTGTGTGACACAGAAAAAAAGAGAAAGAAAAAAGAAAGAAGAAAAGAAAAAATCTCCATGACTTGACGGCCATCACAGTGTTTGCACATCAAACGAAACGAACCTCGGCAGGGGTCTCCCAACGCAAAGAACGGTTTGGTTGGCCAGCCTGAACTCTCGTGACATACATGTTCGTCACTAATATTTGTTGGCATGAGTGAAAAGGAAAGTGCGCTGGAGCAAAATTATAAGCAGAAAACAGTTCATGAATACGATGAATGCGCCTGTCCCATGGCTCCCCAACAACCCAACCATCCTAGTTCGAGGTTCAAAGTTTCGAGCAAGAGTACGGTTGACGTTTGGGAATGAGTTTCCCCCCAATCTTCATAACTGTTTCTGCCCCCCGAATGTCATGTTTCAATCACATAAGACATACCAAAGTGGACATGTGACGAGACGTTACGATTGGATCGATTCTTCGACCAGTACCCCAATATACCTTGGTCTTTCTTAGAACACAGTAGCTAGGTACAGGTAGGGAGGGACCTAGGTATGCAAAAAAACGCATTACTAAGTGGTACCTGTTTTTATATACTACTATTACTACTACTAGTACTACGGTACCTGCCTAGGCATTTCGACCAACCCTGAAATGCCTCGTGCCAATCGTCGATCATTCAATGCAACTTCACATGCCGAAAGCAAGTTTTCCTAGGGGACCAAGGGTTCCCCAGAAATGCCAAATCAGGAAACCCGTCGGGCTTTGGTTTTTTTTTTTCTTCTTTCTACTCTCAATTCCTTTTTCTCAAGCCCTCAACATATATTTTATTATTCTTATTATTATTTTACCGCCACCATTCTTTTCCTTCCCCGGGGGTTCCTGCCAAAAAGAGGGGTTCAAGACCTCGGGAGATAAAGACCCTGCATTTCACCGAAATGGATAAACCAGCCCCTAGCGCGCGGGGAAGGAGAGAGGGAGAGTCGGATTGATTATCGGGAAAACGCTCCTATTTTGGCAGGACTTGCGGTGTCTTTTACTAGTGTACCATGAATTTTGGGTTGGGCATTTTGATGTCTATCTGTTGTATCTGTTGGAGGGTATGCCCCGTCTGAAGAAAAAAAAAAAAGAAAAAAAAAACCCTCGTTCGGATTATCGGACTCATGAACGCTCTGGGGTTGGTTGCATTAGCCCGGTAGATAGATACGTATGCCTACCTACCTTATAATTATCCCAAACTTGATTTGTTCTATCCCCAGTGCTACGATAGCGTCACTTGTTTTAGTCCGGTTTAGGTATGCAGTATGCGGGTGGCGGACGCTGATCATGGAGAGAGGTTTCTCGATGAAGAAGGAGGCTATTATATGATCCACGTGGCGTACATGCATCGCAGAAGCTAAATCTTTTAAGGTTGATTGGGTGGGAGCAGGAGGGCCAAGTCTGCGCGGTGGTGGTGTGACCAGTGAACACACCTACCTAAATAAATGGCGTTTAGAATTCAGATGGATCAAGTTCCGGTCCGCGACGGGCCGTTGGATTTGTTTTACCTTGGTTTACGTCCTTTTGTTCCATACTAAAACAAGACGGACCACCTAGTACCACACATGCAAAGTACCGTATACCACGTACTAGCAGCAGTGTAGTACATTACTGTAGGTACCTACCTACCTACCTAGCTAGGAAAGGTAGCTAACTAAATAAATAGTACTTGGGGGCTGTATGATATGGGCCCCCTCAA

**> MD512_chr05:4178095-4182095**

GACGGTGGAGTCGGCGGCCTTGGTGAAGCGCTCGCCGTAGTGGGCGTAGCGCTCCGGGTCGTAGACGTACATCTTGACCAGGTACTCGTACGCCGAGTCGTTGCCCGACGTCCACCCGCCGTACCGGTCCAGGATCTCGCCCGTGTCGCAGTTGAACTGCCCGCCCGTCAGCCCCGGCCACACCTCGGACGACGGCTTGAGGAAGTAGCTCTCGGCCTTTTGCGCCAGCCGCCCGTACTGCGCGTCGCCCGTCAGGTCCGACAGCCGCTGCCACTCCAGCACCAGCGTGCCCAGCTCCGCCAGCCCGGCCGACTGCGCCCCGCTGTCCAGCTTGTTGTCGTCGCTGAACGTCTGGTTCTCGATGAAGAGCTGCCCCACGGGGATTCCGCTCTTGGTGTTGAACGCGAACTTGAGCGTGTCGGCCAGGCTCTTGGCCTGCCTGAGCAGCACGTCCACGCTTTCCGCCTTGACCTGCAGGTGGCTAAAGGGGCCCTTTAGCAGGTCGTAGGCCGAAAGCAGACCGCCGAGGTAGCGGATGTTGGTCTCGAAGAGGCTAATCTTTGAAGGCTTTGGCGTGTTGGTCTTTGTGAAATTGATGGTGCGCACGTGCTCCAGGATGGTGTTGACAATGTCCGTCTGCTCCATGATGATGGCCGTGTCGAGCCCGTCCACCGCCGTCACGCCCCAGCCGCCACGGTCGTCCGAGTAGTTGCCATACAGCGGCCGCAGCGAGTCGTGCGGGAACGCGTGCACGTGGTACGCATTCCAGGCGTACCGGAACATCTCCACCACGGCCTGCGCCCGCGCCGTCTGGTTGCTTTCGAATCCGGTGTAGTCATAGTTGCTGCCTTTCTGCTTGGGGTATGCCAGTTCCTGAGCAGAGGCCCCGTTTGCGGAGCCAGACAGGGCGCCCAGGAAGAGGCCCCCGAGCACAAAGATGTACGACAGTAGGCGTGCCATTCTCTTTTACTCGCTCACTCTCTGGTTTCCTTATGACTACAGTTCGTGGCCACAGAGCGTCGAGAAAACCTGAAGCAATCAAGGAGCAGGCGAGGGATTACTATATAAAGGCAATCATAACTGCAAGGAAATGGCGGGGCAACACGTCCGTCTGGATATAAATGGTTGTTGATCATCTGGTCGAACAAGCCGAGTGCCTGTAAATTGAGTTTTGGTTTTTTTTGTGCCTAGAAAACTCTGTCCGACTCGATCCAGCGGCGCGCTCAAGAAATTAATTTAGAAAGCCATTAATCATTTCCCAATCCGGTACGAGTCGCCGTCTATGTGGACTACCTTTGGACAGCAAACAAGGCACACTTGTCACAACCTTGCATATGGTAATATAGGGCTGGCCAAACGTCCTGTTTTCTCCGCTACTGTAACACCCACTGTCCGCTCGGGCTCCGGCAACACATCTTTTACCCGTTGCGGCTGTCCGATCTCTGTGCATAACGTCCAACATTCCAGTTCTAGACAAGTCCCTTGATCTCTAATCAGATCAGACTGACTCGGAAGTCGGTCCCGAGAAAGCGAGCAAGCCCTTTTTTCTTTCTGTTTTCTCACTTGTGAATGATTCAGAAGTGCCTGCACACATAAGATGAAGAGCATAAATGTCTTGACATAAACTATCGTTAAAGAGTGTGTTCCTAGAGTTGAAGATCATAAAAGACAAAAAGAAAAAAGCTTTGACCTCAACAAAGCAGTCGGAAAGACGAGAGTCCATGCTATCGCAGCCAAACGAAAAACCAGAATCCCAGATGCTGTAGAAAAAAAAAAGACAAACACCAGGGCCTCAGAAAG

AAATCCCTACCAGGAGACAGCGCCGACCATGCAGACGAGACCAGAAAACAGACGAAACAAGAAACAAAGAAGAGACGCAAAAGGAAAAGAAAACGACAAGCACAGAAGAGGGGTGCAGCGTTGCTGCAATGAAAAGACGGACGCAAATGCCTGCAGCATCAACCGCTCACAAACGTGACAATGTGAGCAGCCGTAGCCGAAGAGAGAGAGAGAGAGAGAGAGAGA

GAGAGAGAGAAAAGCCAACACAGTCGGCCACCCAAGGGGCACATCTCAGCACCTTAGAACATGGCGGGCAGCCTGACGGGCAGCGAGCGCTTGCCCTTGGGGGTGGCGGGCTTGGGCTTGGGCATGGGCATGGTGCTGTTCTTGCCGGGGCGCTTGGGCCCGTTGTTCTTCTTGCCGCCCTTCCTGTCCTTGCGCGGCTTCTTGCCGTCCTTTTTCTTGTCGCAGTCCTTTTTGGCCTCGGCCTCGACGTCGCGCTTCTGGTTGGCGGCGGGCTCGGCGGGCGCGGCGGGCGCGGTGCCGTTCTTGGGCTTGCCAAACTTGTGCCTGAGCTTCTCGAGCTCCTTCTTGATGTCCTTGCCAATGTCGCCCAGGTTCAGGGGGTCGCGCTCCTCGAGGGTGACGCTGCGCTTGGGCGTGGCCTTTTTGGGCTCGCTCTTCTTGGGCGGCTTGCCCGGCTTGGTGTTGTTCTTGCCCACCGGCACGCCGACCTTGGGAGGCGACTTCTTCTTGGCGTCCTCGGCCTTCTTCTTTGCGTCTTCGGCCTTCTTCTTGGCGTCCTCTCCCTTCTTCTTGACGGCGACGGGGACCTGGGGGTCGCTAAACATGTCCTTGGCGGCGGCGCGCTTCACCTGCTGCCTCGGCGGCATGCCCGGCTTGGTGATGTTGAGGTGGGGGATCTTGGGCAGGCGCTTCTTGGCCTCCTCGAGCTTCTTGCGGGCCTTTTCCTTGTCCGCCTCGATCTTCTTCTTGAGGTCCTCCTTGACCTCGCCGGGGCTCTTGGGGTCGCGCTTGGCAAAGTGCGCGGGCATGGGCCCGTTTCCGGGCGCCTCGCTCCGGGCCTGCAGGGCCGCGCTGGCTCCGTCTCCGTTCATGGGGACGGCAACGGCGGCCGAGGCCAGGAGGCTGACGATCAGAGTCTCGAATCTCATCTTGACGTGTTGATGGGGTATAAGATGAGGGATAAAGAGATAAGGACAAGACAACTGCAACGATAGCAAATGGAAGAGAAAAAAGGAGAAAAGGAAAAAAGGAACAGAGGACAAAGGGAATAAGGAAAAAGAGACGTAGGAAAGATAGGAAAGGAAAAGAGAGACCAAAGAGGAGGTGGTGGTGAGAAGGAAGCAAAAAGATCCAGAAAGGGAGGACAACGCCCTTCTTTTATCTTTGCTCGCTTGCCATGTTCACCACCATCTGGTCACGTCTCGGAAGGTGAGTGATACTCCCCAACATCGTACACGCACAACCAGTGATGGCATCAAAGCGGTCGAGTTTCTATTTCGGAATCCAACTACACTACGGCATCCGCTCTGGTATCAAGATTAGTGGAAACGCCAAAGGCTTCTCCTTGACGAATAAGGGGGCCACTCCACATCCAGGTGGTGTACCATTGGAATCTACCAAAGTATCAGTCGCCTGTCGACGCATACTCTTACCCAGCTGCGCTGCAACTCTGCCGCTGCCGTAGGGCACGAGACCGGATCGAGAAGGTCCAAAACTCCGGGATCGTGGTAGTGCGCTCCGAATCCTACTGCATTGGGTAGGTATGGCAGCTGTTAACCTTAGCGCTTGGCGCATTGAACTGGTGGAGAGGGCAGGACCGACCAACAGCAACCCCCCCAACCATCGGGGTCGGGGTTATCCACAATCTCTCAGAACGGATAATACCTTGTACCATGGCAGCATGTTGGCTTGCCACCGGCGTGCGCATGCATCCTGCATCCTTGTGTCAATCAAGCTTGTCGGCTTGTTGTCAGCCCCCCTAGCTACCCGTGCCCGCCCCGACTCGTGCATCTGTTTCGGCTGGGGGGATCCGCCGCCCCGAGAATAGCAAAAAACAGCCACCCCCCCCTTGAATGGGAAAGAAAGCTCGTGGAGACAAACGCAAACTGGACAATGCGTTCAATTCGGAGGCGAGGCGAACCCACCTCGTAGGCGAGAAATTTCTTGGAGGAGGCAAAGACTTTTTGCGGTGCTG

**Chr.6**

**> MD603_chr06:333494-337494**

CGGTTTGCGTTGTTTATATCTGATGATCCCTTGCCGTTTCAGTTGTCCGGTTTGTAGCATTGGAGTTGTTTCCGGACGATTGGTGTGTTGCATGTCTGGGTGTTTTCATTTGTACTGGACGAGAGAACCGTAGTAACATGAAGAGACGCTATCTATCTCTAATCCATGATTGACTTGTGTTGCTGCCGATGCCACGCTGAATAGTCTTTCTTTACATCTCTCACAATTTACCCCGTTGGCCCCGGCTGGTCAATGGCCCCTTACTCAGCTTCACAACGCTATTTTGCCAAAGCCCCAAAATATTCAACCGCATGTGCCATAGTCAGCCTGGCAGTATAGTGGTGTGCAATAGTCGGGCCCGTCTCCACACCAACCATAGTCGGAGCAGCATGGCCCGTAGTTTGTCCCAGTACTGCGCCATGAATCGTTAGCTTTTGTGGTTATGAAAATTGCAACCAATCGTATGCGTTTGTGCCGTTCAAGGTCAATAACATTCAGGACAGCAAGCTCACCAAGTAAATCTGCCCGCGTCGACACCACACCTCCCGTTGGTGCTGGCGTTGGATGGCCGTGGCACGGTGGTGCTCGTGCTGGTGCTGGTGCTGGTGCTCGAGGACGGCACGGTCGAGGTAGACGTGCTCGGCCCAGGCGGCGGCAGCGGCACGGAGCCGTCCCCGCACGTGCCGAACGAGCCCCTGCATCCCACGCCGCAGTATTCGGCGCCGCTGCCGCACCAGTAGTACTCTGAGCAGCAGCTGCCAAACGGCGACCCGGCGCAGGTGGTCGAGTTTCCGCACTGCCCGTTGATGCTGGCGACGAGGCTGCCGGTCGGCACGGGCGGCGAGGTCGCAGTGGTTGTTCTCGTGGCGGTTGTGGTGCTGGTCGTGGTGGTCGTGCCGGATGTCGTCGTCGTCGTCGGCGGAGGAGGTGGTGGTGGCGCGTCGCCGCACCGCCCAAACTGCACCTGGCACCAGCGCACGTCGCAGTAGTCTGGCTTCTCGCCGCAGTACCCGTAGTCGGAGCAGCAGAGGTTGTTGGGGCAGCGGCGGTTGCCGCCTGCTGGGCCGCACTGCGGGGAGACGGCTTGCCGGCGGGAGAGTTGTGCTGCGTCTGGATGGGGGTGGGCTGGGGAACGTTGGATGATGCCTTCAGCGTTGTTGGCGTGTGTGTTCCCGGCCACGGCAAGCTGTAGGGCCGAGAAGAGCAGTGGGAGAATGCTGGCTGTGGGTAGCATCTTGAAGGGTCGTTTTTTTCCGCCGATGGAGACGGTCTGTCAGGGGTTTCTTGGAGGACGCTGCCTGGGAGATGACAGTGAGGGGTCAGCAAATATTAAAGATGATATCAACCGAGCGAATAAATAGCGGAAGTCTCGGGCTATACATACATGTGTGGCTCGCTAGCCTCCACTATACCAGCTTGAGGTCGGCTGGTTATTGCCGATGTGATGCGCATTTTCTCTTCTTGGAGGGTTCTCCGCGGGGGGGGAAGAGCGGTGCCGGAAGTGTCATGAATGGGCAGAATGGAACGCAACCAAATATTCTACAAAAGATAACGAGCGAGATTCCAATGTAACCTGTCTTTGTGGGTGGGAGTGCGAAGAAAATCGTTCGATGCAGGTACCGGCTTAGGATTTGTTTAGGAGCAATTTGCGGTATATGCACCGGCTAAATTGCCCGCCGGGACTTTTCCCACATAGGTAGCTACGGTTGAAGCCGTTTTGCCCGATATCTGACCAATAGTTTCGATCACGGGAGCAAAAAAGCCATTCTCTTTGCCCGCGGCACTAAATACCACGGATGCATGCTTTCACGTCATTCTTCCTGCAAACAAATCAAGATTTGGCTCTCTTCTCGCTTTTACTTGATGCCTAGCTAC

TAGTTCTAGACTAGGAATCAAACAAAAAGCCTTGTGTGGACCTACATCGTTTCGAATCTTCTAGTATCCCAGACAGCAGGCACGCAATTAAACTGTTAGTGATTTGGCACAGGTTCACGTTCGCTGGGCCTGATAGTCGATGGTGGTTTG

AATTAATTTTAGGAAAACCACAGCTCAAGAGCGCCACAATGGAGAGTGCCTAGGATAATTCTAACACTACAATACCGAAAGAGGTATTTATCTAGACTAGTAACATAACTGGGATGGTAGCCGTCATGATCCGCACAGAATCATGCCACAAACCTTGGGAGCTCCTCCAACGTCCAAGAACTCATCGATCGGCGATGGTCCTCATCACTTGTTCCTTGTTCTCAACCTTTTCTAAGCACTTGATAATGAATGCCAGTGTCTTGACTCTGTTGTCCAGACTCGCCTTTTTCCAGTACTCGTTAACCTTTCCTCTCTCACCGGTGACCAGTGCCTTGGCCACCTCCATAGCGAGGGCCTCAGAGTCTTCTGGCATGCCATGATTGCTTGTCGATGTTTGACGTTAATGGGGTGGATTCTGATGACGGCCTCAACGAGAAATTAAGCGTAGGATAGAAAACATGGGAGGAAGTCAGGGAAAAGAAGTGATTCTGGGTGGTGTTGGAAACCCGTCCACGAGAAGATTCGTGAGGTAGCTGTAGTGGTTCAGTGAGAAGTTTTGAAGATTCATAAATATGGCGGACAACCTTCGCATCCTGATCCTCGTAAACGCTACTGGAGATGGTATGTCTTTTGATGCCCCCAGTCTTGTGATATGTCAATTTACCATAGAGACATTACACCGGCTAAGATAGAAGATTCTGGTATGAGCAACCAAGCTGGATCATATAGTTTTCTCGTCTTTAAAGACGACTCTAGCCGTCTCGAGTGTTGGAAAAAGCACGAAGCTGTCCGAAATATTCTAGAAAGAGTGTCTTCGAGTCCGATGAACCGTGACAACCAATGAATAGATTTGACGGTAGAGGGCTTTGTATAAGGGAATACATGCGAACCTAAGCAATTTAGTGTACGTAAAGCTCGTTGGTTAACGTTGCGTACCCCCTGTTCGGCACCCCCCCTGTTCGGCACCCAAAAATAACAACACTTTTATTTTTATCCTCCAACTTCTATTATAAATATCTCGATGAATCTGTCACTTTTGGATTTACTTTTTTCTGATTTTAATTCTCCATTTTCCAATGAAGCAATATACTGAAAAACAGCTTATATCTGCAATTAACGACGTCAATAATGGCAATCCAATTGCAAAAACCTCCCGAAAATGGGGAATACCTAGGTCTACACTTCAAAGTCGACTTAAAGGTTCTCAACCTTATAAAAAAGCACAAAGCCCTTTTCAAAGGCTTTCCACGGAACAGGAAAAGCATTTGGCTGATTGGGTACTTACCCAAACAGCTTTAGGGCTTCCGCCAACGCATCAAGAATTACGCTTTTTTGCCGAACGAATTCTTCAAGCCGCCGGAGAGACAAAAGGCCTTGGAAAACGTTGGATAACTCGTTTTCTGGCTCGTTATCCAATCCTTAAAACCCAAAGGCCCCGTCGAATAGATAACGCCCGGGTTAATGGCGCTACTACGGAGGTAATTAAATCTTGGTGGCTTTATATTACGAACCCGGTTATTAACGCTATTAAACCGGAAAACCGTTGGAATATGGACGAAACCGGTATAATGGAAGGCAAAGGATCTAATGGCCTAGTATTAGGGCTTAACGGGATCCGGCCGTTGCAACGAAAAGAGCCCGGAACGCGTGGTTGGACGACTATAATCGAATGTATATCGGCTACGGGCGTTGCCCTCCCTCCCCTCGTTATATTTAAGGGAAAAAACGTACAACAACAATGGTTTCCCACGGATTTAAGCCCTTTCGATAATTGGCAATTTCATGCAACCGAAAACGGGTGGACAAATAACCAAACGGCTATCGAATGGTTAAAAAAGGTGTTTATTCCGTATACCCAACCTTTAACCCCTGAAAAGCGGTTATTAGTTTTGGATGGCCATGGATTACATATAACGGACGAATTTATGCTTCTTTGCTTGCAAAATAATATTCAACTCCTATATTTACCCCCTCA

**> MD604_chr06:706168-710168**

GCCAAAAATAATAAAATAAAATGTAAAACTTATCGCCACGGATACCGCAAATATAAATTAATATAAATGGAAAGATTTTGTTTTTAACCGCAATTCCGTTTTAACCAATTAATTTATTAAATTTGCATTTTATAACTGTTATTTTTACAATGGTTCGCAAAAATAATTATACCAAAATATATTACCAATTATTTATTATTAATTACGTCCGTTTTGTAATTAATTTTCCGCAATTGCAATAAAATTTCCGTAATGGAATAATCGGTATCGCGGCGGCGAAAATTAACATTTCTTCGCTAAGTCGGGCCGAAACTTGGGTAATTTTATTACCGGCGAAAGTTTAAAACGCGTATTTTAAAAAGAAATTCGGAAAAAAGGTATTTTTTGCAATTTAATAGTTATAACGTTAAAATCTCAAAATTCTTCCTATTTTTTCAGACGGTTTAGGTATTTTCCGGCGGGATTTTAGCAGGGCGTTTTCGGTTACTTTTCTAAATTATAATACTTTTTTGCGGCGCAATAATATAAAATACGCCGCAATTCGTCCCATATTTTAATTTTTAACAATTTCCAAAAAGAAAATTTATTTTTCAAAAAAGAAATTCCTTTTTATATTATATATAATTAATATATATACTAAAAGTAATAAATGCACGGTATATTTAATAATTATATAATAACCGTATTAATTGCGCGGTACTTGTAACAAATATTTCGCGATTTTGGATATTTGAATTGGGTAAATTTTATTTTATTTTTTATATTTTTAATACGTTATATAAATTTAATAATTTTCGAAAATGTTTAAATTATTATTATATAAACGTTTTCGCTAATCCGTCCGCAAATATAAGCTTCGTTAATAAATATTTTACCGCAATTTATCCATTTTAAATTAATTGCCGTAATAATATATTTTATATATTAATATATTTAAATTTCGTTAAAAGATTTGTATTTTATTCTATTACTAACCGAAGTATTTATATATTATTACAAATATTTTACTTGCGCCGAAAATATTTGAATTTAAATTATTTAACAATTTTTCAAACGCAAAAATTATTTTTGCTGTTAAATTTAAAACCAGCAATTTAAACTTAATAAATAAAATGGAAACTATTGTTTGCTTTGAAAGTTTCCAATGGATTAAACCATTGTTAATTTTAAATACAAAACCCTATAAATTGCATTTAATTTTAATATAATTGGCAAATAATATATTGGACGCAACCATTAAAAATCCGTAAGTTTCCTATAATCCGAAATAAATTGCTAGGAAACGCGTCGAAAAAAATAATATATAATTTAATTTGCCGCATTTATATATTTTTGTAATAAATTATTTAAATATTTGGATAATTTTAAAACGGCGAAAATAATACTGAGGCGGATCGTTACGGTTATATATATTACACCTCTTATTTCTTCCTAATATATCCGGATAAACGATTTATTTCCGGGTTCGCCCTGGTTTAGTAATAATTTCGTAGCAAAATAAAATATTGCGGAAAAAAACCTGTTTACATTATATAATTTATATGCAAGGTAAATATACCGTTATTTTTCGTTTTTAATAATTTTTATAGTTAAAAACCATTGCAAATTTTTCATTGGCGGGGAACTATATTTTTCCAAATAAATGCAAAGATTCTTTTCCCCTATTTTTTTTTGTAATATATTACCAGAGAATTATTAATATAAAAAACAATAGTAATTTTGTAA

TTTTTAGAATAAAACAAATATGGTTTTTTTGCGATGGGTGTTACGATCAAGGTATCGGGTAACCTGTTCTTAGGGTAAAGTACAGTGGGTTGAAGCAACTGAGCGTCTGGCTGGGTAACTGGGGTTCTCAATGACTGGGGGTGAAGTTATGATCGTTCTCAAGTAAGTATTGAGGTTAACTAGAGTTTGATTGCTGCTAAACAATTCTAAAATCGAATCTATCTACATGGTAATGAGCGTGCCTTATATAGACTATGTTCCGAATGTGATCATGGTGATCACTTCGTGATCACAATGATC

ACTTTTGTTTAGATCTTCCTGATCATTTAACGATCCAAATGATCACCTCCTCTTGGCGTCACGTGCAGTCACGTGACCCATTGCTTAGGTAATCTTGGTCCTGCCGGTCGGTGTTTCCTGGGCAAGTGTCGTCAGGAGGGGCGTGACCCCACCTGGCCTTCCTGGTACCGGCCCAACCCTTTGGTCGTAACAATGGGTTTTAGGCCAAAATTGTAAAAAATGTTGGAAAAACGTTAATATTATAGCAAAACTAACTTTTTTATACCGTATAAAATTATTAGCAAAAATATAAATATATTTGTTTTTTTATATCTTGGGGGTAAATAATAATATATTGGTTTTTAATCTCAAACAAATTTGGTATTAAAATAAAAAAAAATTATATTTGTTTATTTTATTTCCATAATTTGTTCCGCAATTATTACTAATACCGTGCGAAATAATGTAACAGCAAAAATTATTACACATATATTTCCAATTGTAATTTTTTGCAAATCGTCTTTAACGCAAATTCGTATTTTGCATTTAATTACGTAACTGTCCTGGTTAAATTTATATAAAAAACCCCATTTAAATAATATAAATTTAATTTTTTCTGGGAATTTTGTAAATGGATTTTTTTGTAAAACTTTTTAACGTGGAAATTGCCCATTTTTTTCGTTTATTGTCACGGCCAGGCATACATTGGAGAACCTCAGTGTATTAGGTGCTATCGACGAAAATTCTAAACTGAAGAGAAGAGAGAAATTACAATCGACGACGCGCTCAAGAGACGCGCTTAAATCCGGAGGTAGTGGATCCTTGTCCGATCCCTGGCTCGGTGTGGAGCCGAGTCGTGATTCTGAGGGTAGGTCTAGGGGCCTGATCCTCACATTTATCGCGTATTTAAAATTGTTTTTTAATAAATATTTAAATATGGTTTTGTAGTTATCGGGTATTGGCGGAAAATTGTTCGAATATTTGAAATTTTCCGCGATATATATATTAAAACCTGTTTTTTTACTTTTTGGAATTAATTGGTTATTTTTAAAAAAATTGGTGGTAACCGCGAAAATATTATATATTGTAATTTGTATATTTTCGAATTTCGGATTATAATTTATAAGTTTCGCAAAGTTTTACCAATTCCGTTCCGCAATTATTTTATTTATATGCGCGAATATTGCAAATATATTTTATATTTTATCGTTATATCGATTTTGTTTTATTAACGGACCCTATTATTTGTTTGGAAATTTTATTAATTTACCGCGGCGTTTTAATACGGTTTATAATAGGAATTTTATATTTATTTAAATTTTGGATGGATAATTTTATTATAATAATAAAAATGGATTTTCCACCTTTTTAACGGTATAATAATCTATATTACGGTATTAATATCTGGAAATATTTTCGATTAAAATTTCTTTTTCCGGGCCTTTAAAAACTTAGGTATAATTTTTGGGGTTTTAAAACCGATATCCATTTTTCGGTTTTTTTTGGGTATTTGTTCCGCGATTATAAAGACAATTATATATTTTTTTTCGACAAATAATCCAACGAAATTTGCAACGTTTTAAATATATTTAATTTCTTTTTTTAATTTATCGACGGTAAATTAAATTATTCTGGAAAAGAAAATTGTTTTTCCGTTTATAAAATTAAAGAATAATGTTAATTATATGCATTATTTTGCATGTAATAAGCCCACTTTTATTTGTATATAGCCAATTTTATTTAGTGCCGAAGTGGAGTGGGGAATGGTGGAATTCCGTTACTTTGTTAATGCAGGAATGCAATTTAAAGGTCAGCGAGTGGGGTTAGCTACCCTGTACCGGGTTTAATACCCACCCTGCACCTGCGAGTCAGCTACCCTGTACCGGGTTGAAAATTTCACCAAGTTAACGTTTAAATGCAAACCCAGAGATGGTTTGTATGCAAATGAGGGTGTTTTTTCAAAAACCCATACAAATTAGTTTTTCGGAAATTCATT

**> MD605_chr06:1305584-1309584**

AACGGTTTGCAAAGGGATAAAACACCGGAAAGCCCAGCCCAAAAAATTAACGACCCGTCTTTACTTATTTGGAAAACCCCTAAAACGACCCGAGATATCCGACTTCAACTGCAAAAACTTTCCCAATCCAACAAAACCAACGCTACTTCACGTCTTTTATTTGCAAAAGTCCAAAAAAGCTTCGAAGCCAAAGATACCCTTTTGGCTAGCGCCCAGCAAAAAATCAGCTTATTGGAAGCACAACTGGAGGCAATACGGCCGGTTAAAAGGAGGAGGGTGGTTCCGGATCCAAACGAGCTTTTGGTTAACAAACAGAACATTATTGGATTGCAGGAAAATGATATAGAAAATTTGGAACCTTTAGCTGATGAAGAAGAGGTTAATGAACCGGAGAAGCGTGAAAACGATTGTATTTTTGTCCGTTGATAATTTTATATTTAAATCAGTTTATAAAAGTAGGCATTTCGTTGTTAGATTTTCAGGGTGCCGAACAGGGGGGGGGGTGCCGAACAGGGGGTACGCAACGTTATTGGCAAGCATGAATTGGTCAATCACATAATCAACACTGAAAGGATGAAGGCAAGGAGCGCCTTGCGGCTCGTTGACGTTTTGTTTCCGCTTATTCTGATCTAATGATCTTTTGGGGTTTTACTTGCACCTTTCCCACATTGCTTTTTTACACTTTTTTCGGTAGTTGGTAGTGTCTCGAATATTTTGCAAAACTTGAACGCCGATACGTATTTGATAATCGATACGCTGTTTAGAGAACAATTAGAGCACTTTGCTCGAGTTTGTTTTAAGCATTGCCAGGTTTGAATTTCATCAGATGTCGTTGGTATTTTCTGTTCGTCATCTTTTGCTTTGGCCACCAAAACTACCTTATCTACCTTGGTAAATAAAGTCAGCCAATCACCATTTTGAATGGACAGAATATTAGCCTACTAACTTAGCAAATCCTTCAACCCTTATTTGGAAACCTCTGTTAGAATGATAATAACGAAAATGGACCCATCAAACCATGTATTACGCCTCGAAAAGGACAAATAAATGTATAAGAACCACGTTGCAAGTCGACGAGCTTCCATTGACTGGATTGCATCCGAAAGCCCCGCCGGTGTGTTATGGGTGGCTAAGGCGAAAATCGAACGACACACGCAGTGGCCTGCTCGCCTTTCCGCGGCCGGCTTTCCTCCCAATGAACTTTATACAAGGCATGCATCACGGGCGAGTGACTGGGGCGCCTAGCTGATAGCGACTTAAAACACATAACATAACGTTGCAACTGAGAGAAAAACTCCTTGAGTGTTTTAGAGTGCACGAGCTAGAATACAGAAACTGACTCTGAAAGTTCGCGCTAAAGCAGGATCAGAATAGGCCATGATGATGGGTGCAAGGCACAAAATAGGCTGTAGTTAAATCGTCTCATCGTCAAAAGCTGTTCCAAACCAAGAACCCCTAGTCTCACTTGCAGTGCCCTTAATTCCCTCCGACTCCTCTTTGTCAAGGACAATTCCAAAATATAACCGAATAGCCTCAACACGTTCCGCTTCGGTGCGGAACTCAAGGCTCAGTTTCAAATCCCCTTCCCTCCGCCATTTCAACCTATTCTGATATAGCACAATAGAGCCGTCCAGCTCGCCATCCATGACATCCTTCATATTCGTTCTCCCAGGTGCAGTTAGGGGCTCATTTGATGTTGTGAATCGCTGAAGCAACAATTCACGTGATGCGAAACATGTGCGGCTCATGCCATGCGTGTTTAGGATCTCGATATCCTCGGGCAGGAACTCAAAGTCCGAGTTGAAACAGCACATTGGCTGCCACTCCTCACCAGGGCCACGTCGGAACTGATATACCCATAGCCGCCTAGTTTGA

TCGACCTGCTGTGCAATCGCCTGTTTCACTACTCGCATCTCCGCTTGAGGTCCTATGTGCTGTAGACCGTTCGGTTCAATGCCTCCCGTTCCCTCGAGCAATGGTACAGGCTGGGGTGGACCGTATCCACCAAAGCCCACATCGAGCAAATAGTGTACGCCACCGATCTTGACAATATTGACACAATGCACAAGTCCGCCGAATCTGCCGTTGCAGGGCTCAAAG

ACTCGCGAACCTGCCAAAATAACGTCGAATCCCAGCGACAGGAGGACCGTATTAAGCAATTGGTTCGTTTCAAAGCACCAACCACCGCGGCGGTGGCGGACAATCTTCTCATAGAGACGCGTCGGCGAAACACCGAGCCCTTTGTGCCAGGAGTAATGAGCCTGAAGATTCTCATAAGGCACGCTGACAAGATGGTGCTTCTTGAGAAGGGTAAGGTAGGCTAGCTGTTGAACAGATGTAAGGTTGCTGACGGAGAAGATTCGCTGTGTAGGAGAAAGGTGAATTCGATTGAAATATTCAGTTATTTCCAAGTTGGCGTATCTTGGCCGCTTGCATTGCTGAGCTTGAGCTGTGCTGATGAAGCGAGCCGATAATAATTGGGGCGACAGGCCTCGGAAACAGACTGACGTTTTAGGGATGATCATTATTGTAGCTTGCTTGCTACAGAGGGATTTGATGTAAAGATTTTTTTTCATTCTAGAGTCTTCAAAAAAACTGCAGTTGGTGCAGTCAGTTGAAATATGTGACTGCGGAATCAGTAGCCTTTATGTCATCTCGACTGCTTGCGTTAAAACACTTTCCATAGGCAGAACCTCAAGACACCTAGACCACTATGCGTTCCGTAGAGCAAATGAAAGAATTTGATAATACTCTAGACCCTTGACATATTGCGCGGAACGTGTACGCGTTCAGCACCATATGGGATTTACAGCCCCGGGATTTTACCGTTAGCGGAATGCTGGGGCGTCACATTGCAACCTCTCCATACGTTAAGCATGTTGACGATTTAATGCAAATTTTTGCTTATGCGTGACCAGAACAGGGACAAGATGTTGGTGATGTGATGTAAATTTGTGCGGCGAGTGATGTTGTAAAATCACAACCCTGGACGTCTTTTCGTCTCTGTGATGAGGTTGAAATGGTGCTATCTTAATGCCGGCGATGGGCATTCGGCCCAGAGCCTCGGGGTTGGCGTGAAATTGGTGACGCAGTACAAATCTTATACGGTTATGCCGAAGCAGTTACCGTGTTGGGCAGGAAAAGAAGTGTTGGTGACATGGTTCGAAAAAGTGGAGTTGTGGCTGATATCCGTCCAAATGCTACTTCGCCGCAGGGGATTAAACGTTGCAAAGTTGGTGATAAGAACCGAAAGGCTTGGGACCAACGAAAAGACGTTGCGACCCTAGCGTTCCCACCTTTCTTCGACGTGACGGGGAATTGTTGCAAAAAGATAATAGGGCCAAAACATTAGGAGTTGTGCCTAGACCGCTTGCGAAAGAGGAAAGGACAAAATGTGGGACGTACATTCTTCATTCAAAAGTCGGCTGTTTGCTCCACACTGGGTCGGCCACCGATGTCACTATCCGGTTTTGCAGCCGCGGATGTGGTTCATCTGAAAACTAATAGGTTCGCCCATTGGTCAGGAGTTGTGTAAAGTGCACCTTGCATCCCTGTAAAAATGCCGCGTGGGTTATTTTCGCACATCACCAACATCAACCAGAAGCAACAAGGTTGTTGTCACAGAGTAGTAGTAGTAGTAGTAGTATTAGTTAACGCCGGGCTCGGCCCGGCTTGTTAGCCGGCCGTTAGCAACCTCCCGAGGGGTATCTCGGCGCGCGTTCTATCTTCTTTATTTACACAGGGGGAAAACAAGGAGGGCAGAGGCGGATCGTGCCTGACCGGGTTCGGGCCCGGTCCTGCTTAGGGGGTTAGTTCACTGACGCGACCCCGTGCCTAAGGTGCACGGAGGGTTTGTCTGACGGCTTGTGCCGTGAAGTGTGGGTGAAAAGGCAGCAAGTCTATTCCTCGTCAGAGTTCGAGTCGTTTACGATCGGTGTCCCTAGGCGTAAAGTGCGTTCGTGGCGCGCGGGGCGCTGGCGGGCCGCTCTGGGGCGGGCGC

**> MD606_chr06:2013625-2017625**

TGTTTTCGGTGATCTGTGCCGACACCTTACTGTTTTTCTCTCTCCTCCAAGATTTCGGATTGCGTTCCCATGGTTCCCACATTTCCCATTGACGATTAGCGGCGCAGATGAAAGGGTTTAATGCTTTTGTATTTGGATTTACTTTGGCCTTTGGCGTTGGGTCCGTATATACTTAATTACCATTCATCATTCAGTCACGAAAAGAACTTCGGGACATCATCTACCTGCACATACCTAAACCAAAAATTGTTAGACGGTTGCCTTATCTAAAGGTCGAGTGGACTAACTATACTATTTAGGAAAAGTACCAGCTAAACACTCCAGGAGCTGGATTAGCTCAAAACTGAGACTATCAAGCAATTCGACGGCAATCGGCATCTGGCGCGCGATACCTTGGACGAAATTCTCTCGACGGGGTTCATTGCGGCGAAACATGCAGTCCGACCCTGTCCAATAGTCGTATTGCTTACATATAGGAACGGTCACCTTCCAAGATGAGAAATTGCTGGGGGATGGTTGGCTTGGCTAAGATACCTCGACCAGGGTTCATTTGGTAGCAATAAAAGACTGCCGACTCCTCGTCAATAAGGTTGGCGTTTGACTCGATTATCTATCGCCCTCCCAGGCCAGCGCATCGCTTCCATATTAGCCAGATCTGGCCCGAAGACATATGTTGTACAATACTGGTCAAACTCATACTAAATTCAGGACATATCGCAGCCACAATGCGCGTGACTATCAGCACCTTCTGGATCATTTGCAGCTCCTTGCTACATCTCAGTAACGGAGCTGCCGTCCGAAGGGCTCAAGGCACATGGGACGAGCTCACGTCGACGCCAATCCTACCACGCCAGGAAAACACCGCAGTTGGGATTAATTCAACCTCACTCGCCGTTCTGGGTGGCATCGTAACCAATGGCACACAAGTGCGCAGCTCGGCTCTGGCCTCTATCTACCATGTGCCAACCAACCAGTGGCAGCAGATTGCGGATTTGCCCATCCCACTGAACCACCCGAATGCGGCCGTCATTAATGGCAAGATTTACCTGCTAGGCGGCCTGGCCGATATCGATGGAATCTGGAAAGGCGTACCCAACGCTTGGGTTTGGGATCCAGAGACGGATACCTGGAACGACCTTGAGCCGTTCCCAACCGACACTGAGCGTGGTAGTGCGGCGGTTGCCGTAGTGGGCGACACGGTCTACCTCGCTGGTGGACAACGTTCCCTCGCGCTGGTTCCTGGCGGACTGCACGATACGGTGGACACGGTGTCAGCATTTGACACCCTGTCGGGAAAATGGAAACTACTCCCAAACTTGGCGCTACCTGAGCCACGAGACCATGCCGGTGCCGCCGTGGTGGGGACGAAGTTATTTGTCACAGGAGGTCGGCACCTACGTGAGGGCCTGATCGTGAGCGGCAAGGTCTACTCAGTTGATTTTGCTGCCGAGGAACCTCGATGGACGCTAGAGGATGAGCAAATGCCCACCGCGAGGGGTGGACACATGGCTGCTGCGATTGGAGACCAAGTGTACACATTTGGCGGCGAGGGCTCGGGCAAATCGGACTCAGGCGTCTTTGACGAAGTTGAGGTTCTTGATACCACCACGGGCATTTGGAGTCGGAGGGAGCCAATGAGGCTGCCCCGGCACGGGACATATGCTGTTGCCATCGGCGGGGCCATTTACATACCTGGCGGTGGCTTGCAGCAGGGCGCTGGGGCGGCGACGGATGGTTTTGATGTTTTTAGACCTTGATGGGCCGGCAGGAGAAGCATGGCGGTCACGAAGGCTGTTGCACACAAAGTATGTTTGTCGTCACAGCCCTAGCTTGTGCGCTCACATTTCATGTGGGCATTGTGTCGAAAGCTTGCGA

CAGTCACATTCTTAGGGATGCATATACAAAGTCAATATCAAGACTTGCATGATCGTGGAGGGAAGGAGTAATGAAATTAGGGCACACCATCAATCGACCATCTATAAAACCCCATTGCTTTCAGACATCAACATCATCAACATCATCAAC

ATCATCAACATCATCAACATCATCAACATCATCAAAATTCCAAAGACCTTTTATCCAGCAAGCTGCCAATACACCATGTCGGCATACGGAACCCTCGGCACATATGAAGACGACATCGAGCTCGAACAAATGGCCCCCGACGAAAGGGAACGTGTCGTCTTAAGGATGCCACTCGAGCAGCACGAGTTCTCAATTCCGGCAATCATCGTGATGTTTATCGCCACGTTTGCCGTCATCTTCTGGACAATCTGGTTCGTCGCATCGCCCCCGACTAAACATCACCACGCAGGAGGCGGCTCGGACGGTGACTCGGGCTGGTTTTGAGGATGGTCTTTGTCTTGACCTGTTCTTCTGCGGACCGGCCGTGGAAGGAAACCAGGACTGGCCAGCGTTGTAGCCTGTGTTGGTTTTCTGTGCGAGACTGGGACCTGGCGGTCACGAGTTCTTCCCCGTCCAGCACGACAACGACCTCAGGAATCAGTGGCGTGTCGAGACCCATGCGTTATGTGAATTACATTGGATTTGCGCAAAGACAGTCCCGCTTGCCTAAATGGCTGCCTAGCTGGCGCGTGGAGGCCTATCCCGTAATAAAACTAGGTGCGAAGGATGGGGAAAAGTCAAGCAAGGCTGTACGTGCTTCAGGGCCTAGGGAGTCGGAGAATTAAACTTGCCCTCACCGGCTAATAGAGGACCCATCAGTACCTATAAATCATTTACAAGTGGCCATAATGTGAAAGCTGAATGGGGCTTTGGTGTTGAGGCAACCCAACCAGCCTGAATTAGGCCCAGACTCAGACATGGTAAAACTTGTAATTGATTGGCAAACTCCAAGGTACTGTACTGTAGCTTGGAGTGAGCTTTTTAGCCGTAGGAGTCACAAAGCATCCGATTTGGGGGCGCAGCACAAAATTGGGACCAAGGGACTTTATGACCAAAAGTACGTCAGACCGCTAGTACAGGCAATCACAGTAAAAGACAAGGTGCCAAAACAGAGCCGAGCACTGCATGCACAACCAACAGGCAACTTGACGCCTGTCTGTAGATTGCCCTCAATCATAATTGCAAGGAGCTGATCACGTCCCGAATACTCTCACCATTAATGGCTGATGCCCAGTGCGTCGAGGCCTCCACAAACTTATTCGTAAGGTAGGTGTGTAATCCTAGGTGGTTCCCGTCTCGCCGAATGGACTCATCAGCAGATAGTAAACGCGCCAGACCTGTTCAGGTAGGATACCTAACCTATCCCACTGGCAAAACAAGGGTTATTTGACCCTCTTTTTTCAGCTAATGCATAGCCCGCTGACTGCAGCCGGTCTGATCCGCTTGACGCTTCCAGGCTGCGGGGGCTTGGCGCCTGGGTTTGGGGTGCGCTATGCTGCTCCATCGTCTTCTGATTTGATAGTGCGTACGAGGCATTCGTGTTTGGGAAAAGGAGTCCACCGCTCCGCACATTTAAACGGCTCTAGTCTCACCAGAGATTGACAAATGAGCATTCTTTCAGACCACGCACTCTTTCATGCTCTCTCTCGCATTCAAAAGGGACCGGATTTCTTTTAGACAAGAACCCACCATCATACCATTATTTTCACATATACAGCCTCAGTCGACGCAACAAGAGAAACATCACCATGTCGGATAAGCTGGCTGCAGACGAAGAGACTTCTTCGGTGGTCATAGTGACCCCCGTCGAGGGCTCCGAGAAGGGCGATGCCGCAGATTCCCAGTCCAAATCTCCCGAACAGGAGGATGGTGAAAAGAAGAGCGAAGAGTCGGAAGAGAAGGTCATCGTCGGCATGGTGTGCGGAACCAAGGATCTCTACCAGAAATGGGACCAGAACCAACCCAACAAGTTCACATGGACCGAGAAGTACCCAGAGGACCTAGAGGAGGCGGCCGAGAACGCAGAGACGATGAAGTTTGCCATTCTTGTGCGAAACAGTAGGTCATTCCGAGTACTGCCCAGCGATTTGAGTTG

**> MD607_chr06:2778767-2782767**

CGAGGATAGACAACGCCGCGCGTGCCGCCCGCGCCGGCGTCAGCCCCAGCAGCCTGGCCTACGACGGCATCAGCCTACCAGGCTCGCCCGGAGGCATGGGCATGAGCGGCGCCAAGACGGTGCCGCCGGTCAAGATCGTGGCGGGGAACAAGTGTGACTTGCAGGAGAGCAGGCAGGTGCCGGCGGCGCAGGGCCTCGAGTGGGCGCGCCGTCGCGGCTGTGGCTTCATGGAGACGTCGGCACGGCTCGAAGTCAACATCGAGGAGACGTTTGCGCTCATCGTCCGCCGCGTCGTTGAGAGCCGCCGCCTGGCCGAGATGGGTGTTATGGACCACGACGGCGGCAGGATGGACAAGCGAGGGATGACGAAGCCCTTGAGCCCGCTGCCATCACGCGATACGGAGAAGTCGATCGCCGAGGAGCGGCACGGACATCGGGGCCCCGGGGTTAGAGGCCCTAACATCAACGGCGCCAGGATTGGGGCCCGAAGCAAGGGGTTTTGGAGGATGTTGAGATGTTGGTGACCGGGTGGTACAACATTGTGGGCTCCGAGAGCGCCCTTGAGATGAGAATCACGACAAACGGCTTTTCTTTTTTTTCTCCTTCCTGCGAATTCGACTATTGGGCCACTTGTCGTGTTTTTTTTTTTTTTTTTTTTTTTTTGGCGAAAGGGATATCCAGACTTCACAGTGCATATTTCGCACTTTGTGGTCTCTACATGTGCAGCGTTCTTTGCTTGACGAATTTTCTTTCCCCTGTTATATTTTCAGCATGATTCATTTGCTGGGGTCTGTTGTCTTTGTTTTCCTTTGTTTCTTTCGTCTAATATTTCTCCCTCATGAGATTCTCGGGAGTACACCAAAGTAGGGCGTTTTGGAGTTTGGGCTGCTTTTACTCGTCTCCCAGGCGGCATGGTCGGTATACACAAATGTACATGGAGCAGAAGAGCAATACTTCACGTGTTTGTACGGCCAGGCGTTTTCGACATGGCATCAATCAAAGTCTTTGGACATATCAATGCTATAGAATTGTTGGCAAGCTGCGATTATGTGCGGATAAAATCTTGATGCTCGATTTAGGATTTAGATGGCCGGGTATTAAATCCGAGCTTGCTCTATCTCCATTGCAGCGCAGATCTCGTTCGAAAACAAAAATCATGATACTCACCTATCACTGACCAAGCTACTTTCGTCAGAAATAAAAGCCTCTGTCTAGATGGTCTAGCCCGTAAAACCGTAAAACCGTAAACTCCCTCACTCCCTAATGTCCGATAGCCCTACCCTCTAATCTCTGATAAACAAGATAGTAGGGGAAAGAAGAGAAGAAAAAAAAGCAAGAAAGAAAACGTGAATAAAAAAAACGAGACCCAAACTACAGTGCGCCCATAAATTTCACAAAATAGCTTCCAGCCCAAGGTGAGCTACACTTTGATTACAAATGCCGCCACAGGACTCACGCCGCAGTCTCCGCGGGGTTCTCGTCCGCCCAGTCCCTCCAGGCCTTGTAGGCAGCGTACTCCAAGCTGACCTTCCTGCCCTGGTACCTGACGACCTTGAGGTCAACGTCGCGCCACTTGTCCTTACGGCCCATCCAGCCGGCCTTGCGCATGAGGCTGACGAGCGTTTCCTCCTTTGTCCAGCCCTGCTCGGGGGCGACATCTGGGAGGTAGGTTGCGCCGTAACGGCGCCCGGCGTGGTAGAAGCTGATGCGCAGGCCGTGGGTGCCGAGGACCCAGTCGTCGGCATCGTCGGCATCCTCAAAGTCAGTCAGCAGCGTGACGGCGACCTCGAGCGAGGGCAGCTCGGCGGCCCGCACGGGGTCGAAGCGCGTGTCGTGCAGCGCCGACGTCAGCGCGTACGACGCCAGGCCCTCCTC

GAGCGGCTGCGGCTCAAACGTTCCGATGCATCCACGCAGCTGAGACCCGCGACGCGGGTGGATTGTGTTCAGTGTCACGAACAGCGGGGACTCCTTTGGTGCCGCAGCGGCTGATGACGAGGCCAGCGAGAGCTCGCTGGGGCCGGCGGGCGTGGTGGCCGCCGTGTCCGCGGCCAGAGACGTGGTGCTGCTGGCGGCCGAGGACCCCGAGGAGTGGTCTGCCGAGAGGTCTGCGATGCGGCGCACCGCGGGGGGCTGCTTGGTTGCGGCGCTGTTGGAGGCGACCAGATATGCGGCCCA

CGACTTTTCGACCTCGGCGAGCGGGAGGGGTTCGCGGTCTTCAAAGTGCGCCGTAAGAGCCTCGAAGCAGAAAACACAGTGTTCAGTGGCTGCCATGGCTTCCAGTTTGGGCTGGCAGCGGGTTGCGGTGGTGGTTTTACGGGAAAGTGAGTGCACTCTCGTTGTGACCGAGCTGGTGTGTGTGAATGAGTGAAGTGAGATGGGTGTGGCCGGGGGCAAGGCGGGGCGCGAGAGACAAAGGAAATAAAGATTTGAATGGCTGACGTCGGTGCTGATCAGGGAAGAGATGGGCAGGCAGGTGGCTTGAATTGCAGAAGCTGTCTGCAAGTGACGAGAGAGCTTCGATGTTGTTTGATAACGTAAAAGGACTTGACGCAGGTTGGAAGGTGGGGTTGGAGCTTGCTTAGCTGATTGTTCATCCTAAAGCATGAGGAAAATGAGGGGAAGGTTGCAGCTTGCGAACTGCTTGTTGGTTGGTTGGTGTTGAGATGCAAGCCCATGTTGCAAGCTTGAACTTGACATGCCAGTCAGGTCGAACATTTATCAAAGCACTGGCTTTGACAGCGTTGCATCAAAGGCCATACTGAATAAGAGTCTTGGAGTTGTCGGTTTACGTGGTGTCGATTGATTTGATAACAAAGTGCCGGGCGAATTGAATCGACGTTTGTGACAGCAGCTGCTCAATACCTTGCCAAGCAAGAAATCGCTGAGCAAGTCATGAGAAAGAAAAGCTGGGATTGTTTCAGGTCAGTGCAAGTCGTTGGTCAAAAAAGTCAGCTGAAGCTAATAAAGGCGTGAAATGGCTTTAGCTCTTTCAGGGCGGTGTCGAATGATTACCTATAAATACAGGGTAGGTACTAGGCACATTACAAAGAGTCATGATAGATGACCCTGCCTGGATAAGAGTTTTTCAGATGCAATCTGGGTACCCGGAGAATATGTTTTTAACAATTTGGGTGAGTTTGTAAATAGGTAGGCATGTAACAGTAGCAGCAGACCAGACCAGGTCTCTCAAGTCTCGGCGTAGCATGTCCATCATTGCAACGCTACAAACCCCACCGATCTCGGCACTCAACTGCCATTCACTCTGCCGGGTATACAACCTGACCAAATGTCTTGCTATTAGTCGGGTGAGACCGGCCAATAGAAATAAAGTCCATCACCAACCAAGAGACGTAACCATTAGATTCAAATCGGATCTGCTTTAACGAGGGGCGAGCCACAGAGAAGACGGATTTTTAATATTGCATGCCGGGTCATCTACCTATCAAAGCTCTCACGGCTATTTCAACCCCCAAATTGCACCCGTACTGATTATCCCGGTCACTAATCGTAAAATTCCCATAAATGGTGCCTTGGGATACTGCCCAGCCCGATCCCAGAATGGCAACCCGGATCTTGGTACAATGCCGACCAAGAGTGCCGGCTACTACATCCACGGAGGAACCAAACCCTGAGCCCATGACGGGCGATTGCATCAGGGCAAACCCACTTGGGTGGCCAATATTGTGATAAATAGGACAACATGTTCCCCCTGACAATAGGCGCTGAAACTGGCCAAGTCCCTAACTTGCGCCAGTTATCCGTTGCGTCTGTAAATATCTGAATCGGCAATGGCTTCCATCATCCGTATACTCGCCGCCGCGGCCGTCCTAGCAGGGAGCGCAGCCGCCTCTCGCAGCGCCAAGGTAGAGTCCCGCCAGGCGCCATTCACTCCACGCCCAGGCTACTTCCCCGCGACGGGCGACGACACCATAGCCGGCGGCATATTCTGCATCCCGCTCAACGAGCTCGGCACCGCCGTGGCCAACCTCAACCTCCCCGG

**Chr. 7**

**> MD701_chr07:40844-44844**

GTGCAACATGGCAATCTTCAGGACGCTAGAAGCAAGGAACCGCCCAGGACACGCCAGCTTCCCGTGGGAAAACCCCATGTGGTCTGGACTCGTCGAAACAAATGCCCATCGGTTGGCGTATTTTGGATTTTTCCTCCGCTCAATGAAGCGGTAAGGGTCGAAATGCTCGGGATTCGGGTAGTTTGACTCTTGCCACATCTGGTGCGCAGAAATGGCCAAGTGCTCGCCCTTCCGGATGGTGACGTCCGGGCCGAGCTGAACATCTTCCATAGCGCGACGATGCATGCTGGCCAGCCCCTCGGGCTTGAGCCGCTGCGCTTCTTTGAGGAAGCTGTCCATGAAGGTCAGGCTGTGCATGTTGGTGACGGCCTTTTCCAAGCCGTCCCTTTGTAGGACCTCGATGGCTTCTTGCCGAAGGGGCTCGAAATATTGTCGATTCGCGCAAAGATAGAGAATCGACTGACACAACTGGTTGCTCGTGGTCTGCATCGAGACTTGATTGAGCCCCATCTGGATCCAGATCTCGGGATAGTTGAAACCGTCGGCAGCCTGGCGGAACCATTCGATCGAGTCATCCGGCAGGTCCGGCTTCTGACCTTTCATTTCGGCCACCTTGATCTGCATGTGGCGCTCCTGTATCACGGGTCCCAGAATCGAAGCGATGACTTCCTGGTATTTTCGCACCTTGCGGTTGAGGGGCGAGAACCTGTCCACCAGCGGCCTCAACAGGGGATGAAAGGATCTCGTGATAGCCATGGCCATGAACATGTTCTCGACAAATGAAGTTGTGACGTCGATCCACTGCTGGCTGTGGGCAAAGCTTTCTCCTAGGAAAACCAAGGTGGTCATGTGAGCGACCCATCCTCTGGCTTCGTTGATGAAGTTGACCTCGTGCCACCCTTGCTGGTAACCCAGGCTTTTATCCAAGCATGCGGACAGCTCGGAGGACATGCCCTGGGTAAACTTGGCTGTTGCGATTGATCGTCAGCTCAGCCCGAGCAAGCTAGCAGATGGAACAACTTACGCAGGCGGCGGTTTATGCCATTTTGTATGGCCTTGCTGAACATCCCGGGCGGCATTGGGCGGCAGGTTCGGAAGGTGTCAAAATCCGACAGGAACTCCTCCCGGACATACTCGAGGAAACTAAGCTTTCTCTCGGCATTGATGGCATCGATGTACTCTGGAGGCAGGATTATCAACGGCTTGGAGAGAGTGATGACTTTGAAAGGCCCCGAGAACTGATTTGGTCAGACCGTCAGCCCGGGTTCGCAAATTCACTGCCATGGTGAGGAATAGATCATGTTTACCTTTTGGGATCCTTCAACCAGCAACTGACGAGCATTATGCACAAACTGTCTTCTGGCAGCAGCATAAGTCCAACTGCGCTTCTGGTAGTTGATGATTGGGTAGTTGCTGGTCGTTTGAAAGGAACGACGAAGAGACACCGCATTCCATGCCAGTAATATGCCGAAAGTGGCCAAAAGAGGACCCCATGTCCAAACGAAATCAGGTAACTCCATGTTGAGCCGCTGGTATGGGAGGAAGGCTGATTGCGAATTTAGAACACAGAATCAAGTCAGCTCAACGTCCTGTATAGGTTTTGTTATTCAACCGAGGGGACAATGTGTGGTTGGGGCAGGCGGAGCTGCG

AAGATATGCCCACCACATACGATAAAGGCTGTGGTTTTATACAGTTATGCTGTACAAATCGCTAAATAACCCGTAAATGGACAGCGTTACACTGCTATTATCCCAATTACCTACAGCCACTTGCACGCGCGGGCTGCAATGTTTGCATTTATACCATTCGCACCACGTGATTTTTGGCATGCGGCGAGAATTTAGGCATTTACACCATGTGCACCCCCTTTATAAATCCATACCGTTTGCAACGCAGAAAACCACAACGCGATTACGAGGTTCGATCGAAAATGCGTATATTTACCCAATGAATTGGAATCCTTTCTATTTTACGTGCGCATTTATTCTTTGCTTTGCAAGTGGTGTAGGTGGTGTAGGTGGTGCAGGTGGTGCAGGTGGTGCAGGTGGTGCAGGTGGTGCAGGTGGTGCAGGTGGTGCAGGTGGTGCAGGTGGTGCAGG

TGGTGCAGGTGGTGTAAGCTTGCTGATTCGTAAATTTGTTTGGAATCTTGGTTGCATCATGTTTTTACTTTTGATCGTAGCACTGTGAGCTTATCTATTCATCTGATATAAGTTAAGCTTCTACAGGGCTTTTTCTAAACATGGCAGCTTTCGTCTCGTCTCATAAACTCAAAATGCCCGCAGTTCTAGACCCCAGCTCAATTTTAATCCCCTTATGCTTATCCAATCTTGAAACCTCCTCCACAATGACGCGGCCTTGACAAGTATTGCAACATTAGGCCGACCGGCTGCGAAGCTCGTGATATTTGGCTGTGATACAAACGCTACTACACAGAGTAACTCTACAGGGGTCGGTACCTGTGACGAATGCCTGTGGTCGAGATATAGTACATTTATTTCTACCTCGCAAGATGGCGTACGACTCAAACAATGGGATTATAGAACCGAAAGCGGTTAAGGGGATCATATCGAGCCTTGAGATCACGCAGTTTGGCAAGGCGCCATGGCTGGTGCCCATACCAGTCTTCCTTGGTCTCGCGACCGTTGGCATAGTTGACATAGGCGCTGGGCGTGCGTTGCGGCTGGCCAGCATTCCACTGGTTGCGAACCTCGTTCGCCCACTCCCACATGTCTGCTTCTAGGTTTGACTCTTCAATTGTCCCCTGGAACTGCATCAGATGTCGGTCACCACGGAACGGGTAAGCCGAGGACGCGCGGTCGATGCTTTCCACGCCCTCGGTCGCGTAGCCTTCGTGTATGATGATGGTCTTGTGAGTGAGCTTTGGGTTCTTCGACAATCGCCTCTTGAAGCCGTCCCAGATCTGATGCTCCGCGGTCAGGTTGTAGGTCTGGAGCCCCGCGGTGGTTATGATCCGCTGGGCAGCAGTGCAGATCCAGGGGGTGTCGGCAAGCTGCGGGTAGGTGGATTCTCTGGCGTCCACCGCGACGGCCTCGATGGAGTCGAAAGGGGCCAGCAGCGGTTTTGCTTGCTCGGCGGTGCCGCGGTACTCAAAGAGCCAAAAAATGACGGGCTCCTCCTTTGTTATGTTAGGCATCATGAGAAAGGTTCCTGTGTTTACCCCCATGCCCACCGGGGTTGTGCCGTTGCCGTGAAATTGATTCAAGGCGTTGAAAACAGCATCCAACTTGTCGCCGCGCCAGGTGTAAGCTTTGTGATACCACTTGTCTGGGCCGCGAGGATAAATCTTCACTTGGGCACTTGTCACGATGCCAAAGTTGTGGCCGGCCCCCTTCATCGCCCAATAAAGGTCACTGTGGTCTGTTTTGTTCACCCGGATAGCGGTTCCGTTGGCAGTGACAAGGTTGAACTGCAAGATATTATCGCTCACCATACCGTAAAGCCCCTCAAGTCGCCCATGGCCGCCTCCGAGTGCCAGGCCCATGTAGCCAACACAGGCCGCGGCGCCGGTTGGAGTTACATAGCCATGGTCCCAGAGATGGTTTATGACGCTCCCACCGGTCGATCCGCCTTGTACCCATGCGGTCTTGCCTTTTGGGTCAATGGTTACATTCCTAAGCCTGGCCATGCTGATCTGTATGCCGTCGAAAGACCCGAGTGTGGTTGTCGATCCGTGACCGCCACTGTACGCCAAGAAGTCGATGTCATGCTTGTTGCAGTATTTAACCTAGACAACATTTGCCCAGAGGCTAGTAAACATCATGGTACGCCTACGGTGGAGCTCGAGGATATTTTCATACAATCTTTGGAATGTCATCTTCATGACCTGGTTCCACGACCATCTCAATGTTGGGTGCCAGATAGGTATCCCACCTCTCTGTCGCTTCCCTAAAGTCGGATGTGTTGGGTCCCACAACTTTGGTGCTATTAGGTAGCGGGCCGCCAAGTGCGCTGGGAACTTGCAGGTCGCTCAAGCTCCAT

**> MD702_chr07:297103-301103**

TCGGCCCTGTCAAAAGAAATGGAGCCCAAGGGGACGACCCTCACGTCGGATTCAGGGGTAAAAAGAGAAGTAACCAAGCAAACAAGGCGTTGTGTGTGTGGATCGCGTGGTTCGTGAGGGGATCCGAATTTCCTTTGCTTTTTCTTTCTTGTCTTTTTTTTTTCTTTGGGGACCCGAACTGGGGACCCGAACCGGGGTGGATGCCGTGGGTGGGTGAACCCCCCCTGCTGCATAACGTTTGCCAACAATGTGGGACATGTTGCTTGGTGTGGTAATTCCAAGGGAAATGGCGTCTTGATATGCAAACACAGGGGAAACCCGAGAGGCATGCTGCCACGCAATCAGGAAGCCGGGAAAAAAAAAGAAAAGAAAATAAAACAAAAACGGGTCAATCCCGGTACATCCGGCATGGTCTTCCGAAAGGTGTGCATGACTTGTTTACTCTGTACGTTGTATAACTGTTGATGGAAAGGAATCATTGACTTTTGGAAACTACGGTACACTCAAAGCATCCAATGCCAGAGAGAAGCCCTTGACCTAGGTACAGCAGTGCTATTATCTATCTACTACCTGATTACTCCAAGATACAAGATCAAGATAGATTTACCGTAAGCAGTGGCGGCCCCCACCCCCCAAAGTAATGATAAAATTCCAGAAGAGGAAAGCAGCGGCGCTAAATTTCATTACTACGTCGTACTTTAAGTACTTGCATTGTAGATCTCATATCAACAGCCAAGGTGGGTTCATTATCCGGTAGCACTCGGCCTAGGTAGTAAAGACAGCCGAGATCAACGAAATGCATGGCAAAGATGGGAAGGAAGCCCGCTTGCCCGCAAACCTCGGGATGCACCACGCACACGCACACGCATGCACACAGACACACGACAAAAGTCAGGAATTAATTGTTCTTGATCGTATTTACTATGTTAAACCCCAGGCGCAGTGATAAGGGATACTTTTTAACTATTGCCTACTCTAATTAATCTTCCACTCGGAAAGAAGACTTCAGATGAAAGAGGGTCAATTCGCCAGCCGCCCTGGCAGCAGCTTTATGCCGATGGTGGATATCAGGGGGGGAAAAATCATATGACAGGTGATAAATACAAGGTAGCGACTGGGAGCTGGCAAAGCCGGCACGCTGGGTAAGGTCGTGAATCAATAAAGCCGGAGCAGTATAGGTAGGTAGCTATTACCTACCGTACATACAGTTTAACATAGCTGCCCAATCTCGATCAACACTCCACCCCTTACATGCAGCAGCACTCTACGTGGTACTCCATTCTTGTGGGGACGCAAATCCTCCCTGATGGTGCCAGTGCCCATTGCCAAAAGCCCCCAAATGCAACCAACGTATGTAGTACTGTATCCGGATAACCTCGCAGCGTTCGGCAACCACATCTTTTTACGTGATTGCAACCCTCTATACGAAAAGCCAAAGTCAGCCTTGAGGTTATTTTTTTTTTTTTTCAGCACAAAACAAAACCCTTTGCCTAGTTTTCTTCCGGTAGTCCCCTAGCCTAGTTTTTACTCCCAAAAGGGCTTTTTCTTCGAACAAGTCCAACCCAGAATGTTCCAACAGTGGAGGCGAGCGCATCGGCATCCCGGGAGGAGAGGCCGTTTACCCTGGCAAGCGAACCTGGTCGCCTCCAAGTAGCCGATGTTTCGTCTGTGGCGTTTCCGCCTCCTGAAATAAAAAAGAAGAAGCAAATCCTTTCCGTACGAACCCTTTTTTTTTCGGCGGCCTTGGCCATGTGTTTTTTTATTCTTCCGGCTTGCACCGCCAAGACCGATTTGCTTTTTGTGTTCTGTACCTTGTGTACGTACTTTGTACAACGCCCAAACCACCAAGATCACAAGTGTACTTCTTTTTTATTG

TTATTATTTTTTTATTCTTGGTCTTGGGTCTCGTTTCTTTGATCCGAGCTTCCCCTTGGCGCTCCGGGCCACGCTGACTACCGTGCGTGAACCAGTGCTGCCCTATTGGCTCGGATGGGGGAAGGAGGCGATGGGGGCAGGCGTTTGGAT

GGGGATAATAATAAAGATTTCCTAGCAGTTACAATTGGCTGCATATCAGCGCTCGCACTAGGACTGTTTCCCTTTTAGAACCAGTTTTGACCTGATAGTCCAAGTTTCCATCTACAGTACCTTTTTGACTGCGTCATTCTCTTCACAATCCATAACTCCAAGTATAATAAAACCGGCCTCACAGAACAACATAAGAGAAACACAGATATAACTTACACATACACTTACACTTACATCACCCCCCTCCCTTTATCGTCCCCCACTTCAACCACCAGCCTCTCCTCCATCACTCCCTCTTCCTCCGCCGGATCCTCACCGTGGCCTCCATGTCCGGCACCCTGCCGATGCCATACCACTTGTTGCGCGGCCTCTGCCTGGGCTCAATGTCGTAGCCCAGCAGCATGTGCGCCAGCAGGAGCTTGAGCTCCGTGGCGGCAAAGAACCTCCCGGGGCAGGCGTGGCGCCCGTGGCCAAAGGCCAGGTAGTCGGGGCTGGTGGTGGCAAAGGCGTTGCGCGCGCGCTGGACGTAGTCGGCGGACTCGTCGCGCCTGGCCTCGGCGAAGCGGAACGGCTTGAACGTCGCCGCGTCCGGGTAGATGCCCTCGTCGTGCAGCACGGGGTACGCCTGCAGCATGATGCCGACGCCGCGCGGCAGCGTCTGGCCGTTGGGCAGCGTCAGCCCCTGCGGCGCCGTGACGAGCCGGTTGCTGCCAAAGGTCACAAACGAGTTGAGCCGCGCCGACTCCCGCAGCGTCGAGTCCAGCTTCTCCATCTTGTTCAGCGCCCGCTTGCTCCACTCGCCGCCGTGCGCCGCCAGCACCGACGTGATCTCGTCGCGCAGCTCCGCGACCACGGCCCCGTCGCACGACGCGAGGTCCATGACCACGTGGGCGAGGCTGAAGGCCGAGGTGTGGATCGAGACAAAGTTGAGCACCAGCAGGCGGCCGGCCAGGACGTCCTCCTTCCAGAAGTAAGGGTCGCCCAGCTCCTTGGCCTGCCTGATGGCCCACTGCAGGTAGTCGTTGGGTTGGTCTGTCGACGCCTCCTGCTGCTTCTCCAGGTCCGCGCGGCGACCGTCCCAGTCGGCCAGCCGTCGCCTGATCTCTGGCGCCACGAGCTGCGGAACTTGTGGATCGTGACGTTGATGGGTATGGTGGCCAGGGGAGTGAGCATGGGCCGCAGGATGCCGTAGGTCAGATGCAAGGCGACTCCGCACATGAGCGCCCGGTGCACAAACTGCATGGACAGCTGGAACAGCCTGGGCTCGCGGCAGAGGTCCCTCCCGTAGGCGACGCGGGCCGACACCATTCCCAGCATGGTTTGGACCGTATCGTAGACCCCGACGTCGGTCCAGTCGTCCGGCTTGCCCCACTCGTGGTCGAGGGCCAGCCTTGTCTCCTCTTCCATCTCCGTCACTAGGTTTCCAATCTGCTTGGTCAGATCCCTGGCGATGAGATCCACATGCACCGGCGGGTGGTGCAGCTTGCTGTCCAGGAACGTGTGGTCGAGCTGGAAGAACTCGCTGGCTTGGTCGTACAGGCTGAGGACGTTGTCGGGCTGCGAGTTGATGAAGCCCGTCCACTCCTTGGGCAGCACCACCGTCTCGCCCATGCCGCCCGCCAGTGGGAAGCGATACGGTTGTTCTTGGTCCTTGAACTGTCTATACGCAGACTTGAGGGCATTCTTGCTGTCCATCGTGTTACGCCACTTGGCCTGCCAGTAAGGGAACCAGTCTCCCTCGCGCGCACCCGCAATGGGGAGGTCCGGCAGGTTGTTTGGCTTGAGAAAGAAGTTGTAAACGATGTAGCTGGTCAACAGCACCAATGGCGCTGCGACAGCTGGTCTGAACAAGATTTCAAGCATGTTTAGCGTAAGCATAACCCAAGGGTGATTTGATCAGTAAATATTGCAATGTTTGGCTCCACTAGCATAGCCGACAACTGGTATTGATCACGGAACTCGAGCATTGTTTTAT

**> MD703_chr07:991765-995765**

AAGTGGAATTTCCAAGTTCAGCTCCTCGGTATCGCTTCCGCTGCATGATTGGGATCCCTGACCAGAGACTTCCGGTTGCTGTTCAGGTCCAGTTTCACCATTGGGGTCTCCCGTGACCTTCTCAGTAAGTTGATTGGCACCATCTGTGGACTCATCGGCGTTCTTGAGGACAGTGGGCACAACATCACCCTCACTCTTAATGGCCTCGAGCTTCCTGTCAAGCTTACGGAAAAAGATCTCGTCATGGGCAGGCTGGTTGCGAATACCAAGAAATCCCTTCAGAGGTTGATCGCCCTCAGGTGGCGTCAAGGTACCCATATCGATTGCCTCACCGCCGGCTGCGTCTGCCCCAACAGTGAGGGTTGAATCTGAGGACTCCTCGACATGCTTGTTCTTGCCGGCACTTCCGCGGCTGAGGCATCCGTCAGGTGCTGTGGTAGTAGGTGTTACCTCACCCGAGCTACTTGCGGTAGTGTGTGCTACAGTGGTTGTTGCTGAGGGGAGAATTGACAGAGAGTGATTTGGCGTATGTCCTGCATTGATGACAAGACGCTGGGGTGCTGGTGCTGCGAGGACCTTCAATACGTCCTTCTCCTGCGGCCTGCTCCTTACTCCGTCGGGGCTAGAACTTTGGGGAGGGGGCGAGGATCTGGCTGAGCCGGACTTGGACGTAGTACGGCTAGTTTCGCTGTCGGTAAAAGTTGACTTCTCGACGGCAGTCTTCTTGATGGGAATGCCTTCGAGGTCGGGATGGATAATCTGCACATCAATGGTAGGCACAGGGGTCCCCGACCTGGATTCGTGCGGAGGCATTGCATCCAAAGGCTCCTGGGGAGGACTGCTGACATGTGATCGAGGCTGGGATTGCTGCAAAGGACTGATTCGTGCCCCGAGTGGCGGGCTAAGGCCAGACGCACCGTTTGCCAGCCGAGCCAGAACCTGCGAGGCTTTTGAATGACATGACGTCGCCTGGGTCAAGGCCTGTTCCTGTTCCGACGACACTGAAGACACCGGCACACTGTCCGAGCCAGCCGGGTGTCCACAGCTGTCGCCGCCACCTCTAAGGCCAATGACATCCTCACCGCACTCGGGTATTCCGGAGATGCGTGCTCGTGTAACCTCTAACCTGAGAAGAGCAATCTCTTCCAATAGAATGGCTCGTTCAGCCTCCCAAATGGTGCGTTCTTCTTGATAGACCTCCTCAGCGCGTGCCCGATTGGCTTCCAAATACTTTCGTTCGTTCTCCCAAGCCTGACGGATTGATGCATTCTGCTTCTCGAGTATTGCTATCCTCTGTGCATATAGGTTGCCCGCCGATGCAGTAGAATCAGCAGAGGCCGCGCGGGTCTGCATTCCATACGAATGTGCCGATGTTGGTATTGAACTTGAAAGTGAGGGTGGTACTGAGTGCGGCGGCGACAGCATGGTGTCGCCAGTAGGTAGTGAATGGGTTAAGAGGGCACGCGATGTCCCAATAAGTTCACTGACTGTCGGGACGGAGGTGCCTCTGAATGTTGGCGACAGACTGGCCGAACGATGCTGCGTCTGTTTCTGTTGGCGCTGCTGCAAGTAAAGTTGATGGCTCATCTGTGTTGAAGTCGGAGCTAGCCTGGGAGAGAAGAAATGATTCGATTGTCTACCCCTTGCACGGTACGAGTTGCTGGTCGGGAGTGCAGGGCGGGCCTGAGGCTCAAAGTAATGTGTCGCATTCCTCGGAAGGGACAGAGGAGAGATTACAGAAGTGTCACCTCCCTGCGAGAGGGAGAAATGATGCATGTCGTGTGCACCTGAAGGTAGGAA

AGAGTCGGACGAGGCTAGCCTCGCCATGTTGTTTGGCTGCACTGTCGCTGGTGTGGCACATGAGCTGATAAGCCGTAGTTATGATCCAGACGAGGAGGACCGGTGACAAGTCCGAGGCGAGTGCTTTTTGTCGAAAACCGAGGGAGGGGCGAGCAGAAGAAAAAGAAGTCGAGACGATCGGGCGAGATGACAAAATGTGGAGGGCGTCTTTTGGCTCTTTGGATCGGTCGGGGGCGAGTTACGAAGAATGTTGTACTAGTCTAAGGTAGTGAGGTCCAAAGGTAGCTCCTCCTGACCTTG

TGCGTATTTTGTGTCCCTAGACAAACTGCAGGTGGTCCCTTTTAGACTTGGGTCAAGTCGAGCGAGATCAAGTTTGTCGTGGGTCTGCTTGTCCCTACCGGCAGATGTCGGACTGAATCGGGCTGCAGCTGGAGGACTAAGATTAAGTGCGTTCTGGTGTTCTTGTGCCTGACTCAATGGTGCGGAGCGTGCCCCAAGCAGCAACAAGGCGTGGGTGAGTGGGCCACCAGTGACGTCTGCGTTCTTTCTTTGGCCTGGTTTGGTTTGGGTTCGGTTGATTGTCGAGAAGAAAAAGTGGAGTCGGGCAGCGGGATGCACCTTTCAAAGTTTCGGTCGTTTCGCGCAGAGAAAACAAGCAGTGATTCGCACAACGAATAGGAAAGCTGGGATGACACAGCGCCGGTCCGTCCTGCCTTTATAGTTAGAATTAGCACACGCGACAGTTTGCGTGGTTGGACCATGTTGTACGAGGTACCGAATAGAATTTGTGAAGATGTGCGCACATGTGAGTTGTGAATACAGTACGAGTAAACGGTGCTGACAAGTCTGTTTGAGAATTTGGAAAAAGCAAAGCAAAAGAAAGGGGGTAGGCCGGAAAACAAAAAAAGAAAGAAAAAGAAAAAAAACTGTACACTTTCTCACCCGCACCTTGGCTGGTCTGACTTACTTTGGGTGGCGCAGAAGGAACTCAGCTTCGTTGTTGGCTTTTCGGTTTGAGTACTCCGTAGATTTGGGAGACGGGAACTTGATGGCGTACAAGTTCAACCTCTTTCAGCTGGAAACCGATACATACATGGGTAGAGAGAGAAGCTAAAGCATCCAAACATCCCCCTTTTTTTTTGTTCCGGCCAGCGGTGCGTTTTCAACTTAATGGTGGTGGTGGTGTTTGGCAAGTGGTGGGAAAGGTGTGTTCCAAAGAATTAAGTTTTCCTAGACGGACTCCAGTGTGTGCGTCTGTGTGAGTGCGTGTTGTGTCTGTCCAATCTCCAACTGAAGAAGACTGAATTCTCCAAGTACCAGACCCACTTAGATTCGGTGGGAAAGGGGTTTCCCTCTATCGTGCCGCTTCCGCGTTAGGTCGCTCTTTTGGTCGCGTTCCAAAATTCAATCAATCTCCCTATGGACGGAGGGAGGAATGTGGAAAAGAAGCGCGCGATGCACCGGATGGAAGTGCCGGATCGCAAGGGTACCTGCTGACGCCACCGACCGGTCCAGGTGTCAATGTGGACTAAGAGGGTCTGGTTGTTGTGGTGGCGGTCAGGGCAAAAGTTTACTTTGTAAACGCAAAGGAAAGAAGAAAAGAAAGGGCTAGGCTGGCTTGCTCCCGAGACTGGACCTGGAATGCTATAGAAGCAGTGGGTTTGAAATGCGAACAAAAGATAAAAGGACGCGGCGGTGAGACCCAAGGCCAGCACAACAAGTGTTGATTGATGAACGTTGTGTTTTGGTAGTTGTTGTTTGCTTCATTTGTATGCGGTAGGTACGTGTCTCGCGACCAGGCAAAGTTGGGGGTAAAAACAGTGGAGGCCAGATTAGAGCACACACTCTCAGACAGTGCCATACAGAAGGGTCCACGTCAGCTGCACAAGGTATTACAACCTAAGCTGAAGATGAAATAATGATTGCCAATTCGATAGGCTCGGATCTGCTGGTGTTGAGTGAGCGAAACCAATCTAATTTGAGAAGGAGAAAAAAAAAAGACAGAAAAGGCAGAAAAAAGAACAGACGGATCTCAATTATGCTGTAAGACCAGGGAAAATCCAGGGTCCTCATGCGATCCTAATTTAGGTAGTAATTTTCCAAACCAGGCACGAGAAGGAGTTGATCGGCGGGGGAGGGGACTCCGCCGTGGTGGCAGTGGAGGGAACAACACACACAATACAACACTCAGGTTCTCGGA

**> MD704_chr07:1071944-1075944**

GGGAGGGATTCAATTGTCCAGGAGCAGAAGAGGGTTGTTGGCCGGCGCATCCCAAAGGCAGGGATTTCAATCATCGCCGAGGGCTCGACTCGCAAGGTACTTGCTGACCGTCTCGGCCGTCCATGATCGTCTTTTTGATTTCCCACGGGCTATATAATGAGATGGTTGTGAAGACAGACCATATGTTATCCGGAACGTTGAGCGATATTGGTTCCGCTTTATCGTTTGGTCAAACAGGTGAACTCCAAGCTGCAAAAAAGCTTTTAGTAGCCTGCAACTCCTGCAACTAACGGGCTGGATAGCACAAAGGGCATCATCGCCAGCCAGTCGTAGAGCCCATGGGCTATACAAACAAGGAAGCCTGTTCTCGAGTATATAGATTGAGGAACTCGTATTTTGCCAGTGGGCCACGGTGGGATCTTCCTGCCACAAAAGATTATACATCAAATGCAAAGTGACGAACAGAGTCTGTATTCCGGCGTGGTAGGAGGGATCAAATCTGTGATCCAACCTACGCAAACTGCGGCATGTACGGCACCGCGGGTTGTAGTCGTCATGGCGTTGACGGAAAGACGAGACGCCGAAGGTAGTTGGAAAATTCCGCGTCTCCATATGCTCTGAATCCCATGTCCTTGGCTGTAAAGTCTTTGTCACCAACCATTAGTGCTGGATCCTTGGTCTCAATGACGCATCGCCAAAGATGTCTGAGGAGTTCGAAATGCACTTTGCCTGGACCACACTGAGCCTCCTGCAGCCGTGTAACCGTAAACGCGATTTCAGGCCATGAAGAAGAGGCGGCTTGGCCCGAGCTGGCTACTCGCCACTGGCAAGATTGAGCGAGATGGGGCTCAACGATTTCCCAAGTGGCTGGTATCATGACGGACGATCACCCGACCCGACATTTGAACGTAGCTAGCCTTGCTGCCACGACTGCTGTCTCGGAGTAGAAACTGTCAGAGTCTTCGACATCTGAGTCGTTCTCGGTGCCTCCTCGGTGCCGAGCCCCAGGTGCCCGCCTGCGGCCGCAATAGTCAGGTCGCGTACTATCCGAGCCTGCTCTTCAGATAACCTTCCCGGCTGCATGGCTGGGATTAAGAGTCAGCAACTGCACTACCGGGGACTGTAGACGTTGAGGTGAGTCAAACTGTATTCTTTGACCATGGCATTAGTAGTGCGGTAGATGCGCACAGCACAGTCGAAAAACTTGACAAGGTCATCAGGAAGGTGTGAACAGAATCCGAGTCGATAAAGCATTCAGTCGTGCATTGTGGGCTTGTTTGTTCCGCAACCCCTCGAGAGCCATCCAGAAAGGAGCCTCATCCGCCCCTGGCTGGCGCATCGGGATATCCAGCTCCATCGTGTTCTTAAACTTACGGTACTGCACCTGGGGTTTGGATCTTCTGGACAGCGACTGGCTAGACAAAAAGAGGTCATTGCTGCGCGGGAGGCAACTGCCAAAAGTCACCCAGAGTGACAATGTTCCAGCCAGCGACGGGCAGTGGTTTGTGTCCAGTGTGTCGAGGTAATGCGCTGGCTCTAACGCTATGACGGCTGCGATGGCGGAGCCATCACGGTGCATGCCCGCTGCCCCCGCGGCGGCCTGCTCTTGATTCTCGTCAGCACACACCCATGTACGCGGCGTGAGCGTGGTGGAGGCGTTGATCATATGTCGATTCTTGCTGGCTTCACTACGGTATTGTTCTCCTTCACACGCGTCACCGCCTGGTGGTGATGTGACGGCCGTCAACAATCGTCTGTTGAGGTTTGTGAAGGCGATGGCATAGACCGGGTCCTTTGCGTGTAAAGCCTCGCCATGGACAGAGCTTCAGGAATGGGTGAAAGGTTATCATGTTTGTCCATTTACTGCCAGTCAAGTTGCAAAATCCTTCGAGGGTTTGCTCCGCAGTGGCTCGACTGGATGCGCTCGATAGGCCAGTTTTTCGCATCGTT

TCTCAACACGTTGAAAACGAGGTTCACCTCTTTTGCTTGAGCTCGAGTTGATATATATATATATATATATATATATATATATATATATATATATATAGCGGATGGTAAAGTGAGTCTTGTTCGTAGTGGCCTTCGAATTCAGGCTGATCC

ATTCGTTGACAACCACCCCCAGGATAGTGAGGACCATGTGCGTGCATCCAGTCATCGAATGTGGGAAGGATGAGTTTTGATACATCCTTATGCCTGCGCGCGGTTTTACAGTCTTCTTTTGCCCTTGCGATGACGCCTGCCTGCCGATTGTATGGTGTTGGAAAAATTCCAGTCTTCCTGCAACTGGATGCCGTGGTGACATTGCACTGCAGATGCAAGTTGATCCCACTGGAAGCCACGGACATGGATGTAAGAAATACATCCACTTCTGACACCTTCTGGCTTCCTCTGCTCCATTTTTCATGGAGAGTTGGACTTAGGTTGGGATCTTCCAAAAATAAGATTCAAGGAGATATATATAGATGGATAATGGAAGCCCTATGCTTGAGGTAGAGCAAGGGATAAATCGGTGCAGGAAGCAGATCACCCATCCTGTGGCCTCCCGTCTCTGCGGATGATTGTACGTCTTGGGCGGAGAAAAGAAGTCCGAAGGTGATCCAGGCAATCGAGTCAAGCAGGGTATAGCTTGTGTAGGTACAAGGTGTTATGGGTGTAAAATCCACACATGGATCCAACAAAATATAGCCGGAGTGATTCTCGTGGCTGCGGATCCAATGAGACTCATGGGCTGTCTTGTTGTCATTGTGACTTATAGTTCAAGGCGATTTATGATCCTGTGGGAAGCGAATGAATAGCTACCAAGGAATGAGGTCAGATGAAATAGTGTATGAGCTGATATCGACGGCACTCACCAGCCGATACTTGCTGAACATCGAAAAGGGGAATAGACCCTAACCCTAACGGGTACAGTACCACCCCTGACTTGCCCTGCCAAGCCTGCATACCTTATCCTGGAAAACACGGATGTTTTCCGAGTGCCATACGCAGTACCATGGCGGACACGAGAGTGGAGTGGGCTTGAGGAGCCGGTTAGTCGCCTACAAAGGCAATAAACAGGTGGGGGGAGAGGCCATATCTGCAATGCGAGGCTCTGTCCCGGCATTGTCCCACCGACTAATTACCCTATCGCCGTACCGATGATGAAAGCGAGAGCGAGTGAGGAACTTGCAAATGAAATGTTGATGTGGGAAGAGGACTCCAACGCTAAGGCTGCATGTGGATAGCCAAGCCACCAGGCTGACTGGGTTTGTGGGGCAGAGGAAGCCCCCCACATAAAGCTAAGATGAAGCTGGTACTGTTGTGTACCATATCGTGTCCGGTCGAGTAGCGATGAGATTCTTTGTTGGACTTCATTCTTTTTGGATTAGGAGGGCTTGAGAATGCAAAAGAGAATTGTTCTGACGGCAGAAGTTGGTGTTAATATCGATGTTTTGGGCGTGGTACGCATGGGGACAGACATGGAATATACTCTTGGTACATCGACAGGATCACCACCATGTCCCTAGTTCCACCACATCCCCCGCACGCAACCCTGGATGCTCCAATGCTCGTTTGCAAACATGCCGGTGACTCACTGGTGTGAGGTCTGATGTATCTGATGTTGCATTGGCATTTGGGGTTGGTCGAGGCGCAGCTTAGCTCGACCTGTTAGGGGCTGGGCTTACTTGGGACATGCATCAGGGTTGGTCGACGTGATGATTGCTGCGGAAAGATAATCCCCCAGGCTCCCACTCGAGGGCATTGCTGAAATCTCAGGCTTCTTACTCCTCTTACCATCATTGTTAATCATCATTCATCATTGTGTTGAGACCAACTCGCAGTCTCAGTATATCGTAAAGAATACGCACGAGCTTTTGACACCTCCCTGTCAATTAAGCCCAAAGCTACTGTGGGAACAAAGCCAAGCAGTTCCCAATTGAGCTTGAAACCACTCCAGAGATTGGTGGAATCGGTGTGGTAGCTCAGCATCCAGGTTGTCTGCTTCTTGAGTCGACTCGGA
